# Supplementary material for: Homo-BacPROTAC-induced degradation of ClpC1 as a strategy against drug-resistant mycobacteria
Source: Nat Commun. 2024 Mar 5;15:2005. doi: 10.1038/s41467-024-46218-7 (PMC10914731; doi:10.1038/s41467-024-46218-7)
Supplement: Supplementary file 1 — Supplementary Information [file 41467_2024_46218_MOESM1_ESM.pdf]

Supplementary Information

Homo-BacPROTAC-induced degradation of ClpC1 as a strategy against drug-resistant mycobacteria

Lukas Junk*$[1], Volker M. Schmiedel$[2], Somraj Guha[1], Katharina Fischel[2], Peter Greb[2], Kristin Vill[3], Violetta Krisilia[3], Lasse van Geelen[3], Klaus Rumpel[2], Parvinder Kaur[4], Ramya V. Krishnamurthy[4], Shridhar Narayanan[4], Radha Krishan Shandil[4], Mayas Singh[4], Christiane Kofink[2], Andreas Mantoulidis[2], Philipp Biber[2], Gerhard Gmaschitz[2], Uli Kazmaier[1], Anton Meinhart[5], Julia Leodolter[5], David Hoi[5], Sabryna Junker[5], Francesca Ester Morreale[5], Tim Clausen[5], Rainer Kalscheuer[3], Harald Weinstabl*[2], Guido Boehmelt*[2]

[1] Organic Chemistry I, Saarland University, Campus Building C4.2, 66123 Saarbrücken, Germany

[2] Boehringer Ingelheim RCV GmbH & Co. KG, Dr. Boehringer-Gasse 5-11, 1121 Vienna, Austria

[3] Heinrich Heine University Düsseldorf, Faculty of Mathematics and Natural Sciences, Institute of Pharmaceutical Biology and Biotechnology, 40225 Düsseldorf, Germany

[4] Foundation for Neglected Disease Research, Plot No. 20A, KIADB Industrial Area, Veerapura Village, Doddaballapur, Bengaluru 561203, Karnataka, India

[5] Research Institute of Molecular Pathology, Vienna BioCenter, Vienna, Austria

Contents

Supplementary Tables 1-11 3

Supplementary Fig. 1. MIC on Mtb Beijing strain HN878. 12

Supplementary Fig. 2. MIC on replicating versus starvation-induced, dormant *Mtb* H37Rv cells. 13

Supplementary Fig. 3. BacPROTAC mediated degradation of ClpC1-NTD over time. 14

Investigation of Intramolecular Hydrogen Bonds (IMHB) 15

Supplementary Tables 14-17. Pharmacokinetic data after oral administration 18

General Information (Chemistry) 22

General Synthetic Procedures 23

Synthesis of the compounds 25

- Synthesis of exit vector 6 Homo-BacPROTACs (6 – 9) 25
- Synthesis of enantiomeric exit vector 6 Homo-BacPROTAC (8a) 30
- Synthesis of exit vector 7 triazol-based Homo-BacPROTACs (11 + 12) 42
- Synthesis of enantiomeric exit vector 7 Homo-BacPROTAC (12a) 45
- Synthesis of exit vector 3 triazole-based Homo-BacPROTACs (SI-39, SI-40) 49
- Synthesis of exit vector 6 Homo-BacPROTACs via olefin metathesis (15, 16) 61
- Synthesis of exit vector 3 Homo-BacPROTAC via olefin metathesis (17) 66
- Synthesis of Trp-Nα-methylated exit vector 7 Homo-BacPROTACs (21, 22) 72
- Synthesis of Trp-Nα-methylated dCymC (23) 77
- Synthesis of Trp-Nα-methylated exit vector 6 Homo-BacPROTACs (SI-57, 27, 28, SI-61) 80
- Synthesis of Trp-Nα methylated exit vector 3 Homo-BacPROTACs (SI-65, SI-66) 89
- Synthesis of a Phenylalanine-containing dCym derivative (SI-72) 93
- Synthesis of Glutamine-containing dCym derivatives (SI-74, SI-76) 97

Supplementary Fig. 7: SPR Sensorgrams 102

Supplementary Fig. 8 – 209: Copies of NMR spectra 104

Supplementary Fig. 210: SPR stacking experiment 217

References 218

Supplementary Tables 1-11

Supplementary Table 1. Structure-Activity Relationships part 1 (SPR, Caco-2)

compound[1]	Compound descriptor	K_D ClpC1-NTD[2] [nM] Mean ± SD	K_D correlation[3]	k_{off} ClpC1-NTD[4] [s^{-1}] Mean ± SD	Caco-2 permeability[5]				Permeability Correlation[6]	
					P (A-B)	P (B-A)	Efflux ratio	Int. Perm	P (A-B)	Ratio
SI-38	[3-OPra]	5.6 ± 0.6$_a$	2	(5 ± 1)E-2	<0.2	18	-	9.1	**>75**	**>31**
SI-64	[6-Me][3-OPra]	11 ± 1$_a$		(2 ± 1)E-1	15	43	2.9	29.0		
**SI-40**	**[3,3-Tri][5]**	0.66 ± 0.04$_a$	11	9E-4 ± 8E-5	<2.5	<0.7	-	1.60	-	-
**SI-66**	**[6-Me][3,3-Tri][5]**	7 ± 2$_a$		1E-2 ± 6E-3	<4.8	<2.5	-	3.7		
**SI-39**	**[3,3-Tri][14-O3,6,9,12]**	0.45 ± 0.04$_a$	4.5	(9 ± 3)E-4	<4.1	<1.7	-	2.9	-	-
**SI-65**	**[6-Me][3,3-Tri][14-O3,6,9,12]**	1.8 ± 0.7$_a$		1E-2 ± 6E-3	<8.6	<5.1	-	6.9		
14	[3-Allyl]	10 ± 1$_a$		(6 ± 6)E-2	3.3	40	12.1	21.7		
**17**	**[3,3][4-E2]**	23 ± 5$_a$		4E-3 ± 7E-4	<2.5	0.9	-	1.7		
10	[7-Pra]	4.0 ± 0.7$_b$	3.5	(4 ± 3)E-2	<0.1	14	-	7.1	**>28**	
20	[6-Me][7-Pra]	13.9 ± 0.3$_a$		(3 ± 3)E-1	2.8	28.5	11.8	15.7		
24	[7-Tri][5]	4.6 ± 0.2$_a$	5.7	(4 ± 3)E-2	<0.3	11	-	5.6	**>3**	**>1.5**
25	[6-Me][7-Tri][5]	26.1 ± 0.1$_a$		1E-1 ± 2E-2	0.9	22	25.1	11.4		
SI-74	[7-NMe2Glu]	4 ± 2$_a$	5.9	(6 ± 6)E-2	<0.5	3	>6	1.7	-	-
SI-76	[6-Me][7-NMe2Glu]	26 ± 6$_a$		6E-2 ± 5E-3	<0.1	8.4	>84	4.3		
**12**	**[7,7-Tri][5]**	0.28 ± 0.08$_c$	3	(7 ± 2)E-4	0.7	0.2	0.3	0.5	0.7	1
**22**	**[6-Me][7,7-Tri][5]**	0.9 ± 0.3$_a$		1E-2 ± 5E-3	0.5	0.2	0.3	0.3		
**11**	**[7,7-Tri][8-O3,6]**	0.4 ± 0.2$_d$	9	(7 ± 2)E-4	1.6	4.2	2.6	2.9	0.3	**4.33**
**21**	**[6-Me][7,7-Tri][8-O3,6]**	3.6 ± 0.9$_a$		(8 ± 4)E-3	<0.5	<0.3	-	0.4		
SI-72	[3-Phe][6-propargyl]	14 ± 6$_a$		(6 ± 1)E-2	0.2	12.5	61.4	6.4		
5	[6-propargyl]	3.5 ± 0.1$_a$	4	(3 ± 2)E-2	1.1	23	20.4	12.1	**13.6**	**9.3**
26	[6-Me][6-propargyl]	15 ± 3$_a$		4E-2 ± 4E-3	15	33	2.2	24.0		
13	[6-allyl]	3.6 ± 0.2$_a$	2.2	(9 ± 2)E-2	2.4	19	7.9	10.7	**14.6**	**3.2**
SI-60	[6-Me][6-allyl]	8 ± 3$_a$		(3 ± 1)E-2	35	88	2.5	61.5		
**15**	**[6,6][4-E2]**	1.2 ± 0.8$_a$	1.5	(8 ± 5)E-4	2.2	1.8	0.8	2.0	0.4	
**28**	**[6-Me][6,6][4-E2]**	1.8 ± 0.1$_a$		1E-3 ± 6E-5	0.9	<0.4	-	0.66		
**16**	**[6,6][4]**	0.5 ± 0.2$_a$	**1**	(4 ± 2)E-4	<1.4	<1.1	-	1.3	-	-
**SI-61**	**[6-Me][6,6][4]**	0.54 ± 0.06$_a$		(6 ± 2)E-4	<4.2	<1.3	-	2.75		
**9**	**[6,6-Tri][5]**	0.6 ± 0.2$_e$	2.7	5E-4 ± 8E-5	<0.2	0	-	0.1	-	-
**SI-57**	**[6-Me][6,6-Tri][5]**	1.6 ± 0.8$_a$		1E-3 ± 7E-4	<2.2	<0.7	-	1.5		
**6**	**[6,6-Tri][5-O3]**	0.9 ± 0.4$_b$		1E-3 ± 4E-4	<1.7	2.4	-	2.1		
**7**	**[6,6-Tri][11-O3,6,9]**	0.46 ± 0.05$_b$		1E-3 ± 3E-4	not determined					
**8**	**[6,6-Tri][14-O3,6,9,12]**	0.4 ± 0.1$_b$	**1**	1E-3 ± 4E-4	<0.6	<0.3	-	0.4	-	-
**27**	**[6-Me][6,6-Tri][14-O3,6,9,12]**	0.4 ± 0.2$_a$		2E-3 ± 5E-4	<6.4	<1.7	-	4.1		
dCymC	dCymC	1.1 ± 0.4$_f$	10	(1 ± 3)E-2	1.3	3.1	3	2.2	**3**	**1.57**
23	[6-Me]-dCymC	12.1 ± 0.3$_a$		(7 ± 8)E-2	3.9	7.3	1.9	5.6		

[1] bold compounds/descriptors: Homo-BacPROTACs; [2] Dissociation constant K_D of compound binding to ClpC1-NTD determined by SPR. Mean values ± standard deviation. Footnotes indicate the number of measurements performed for the respective compound: a) n = 2, b) n = 4 c), n = 10, d) n = 7, e) n = 3, f) n = 145. For the calculation of all reported SPR values, molar compound concentrations were used throughout, regardless of the number of binding moieties of the molecule. [3] Ratio of K_D values (ClpC1-NTD) of Trp-N^{α}-methylated and non-Trp-N^{α}-methylated compounds. Bold: no measurable influence; [4] Dissociation rate constant k_{off} of compound binding to ClpC1-NTD determined by SPR. Mean values ± standard deviation. The association rate constant k_{on} can be calculated using the formula $k_{on} = k_{off}/K_D$ [5] Permeability across Caco-2 monolayers in *P(A–B)*: apical to basolateral and *P(B–A)*: basolateral to apical direction (values reported in 10^{-6} cm s^{-1}); efflux ratio = P(B–A)/P(A–B); Int. perm: mean value of P(A–B) and P(B–A). [6] Influence of Trp-N^{α} methylation on P(A-B) and efflux ratio. Bold: positive influence.

Supplementary Table 2. Structure-Activity Relationships part 2 (MetStab, MIC). For MIC values, mean ± SD are given with footnotes indicating the number of measurements performed: a) n = 1, b) n = 3, c) n = 5, d) n = 2, e) n = 4, f) n = 6, g) n = 7, h) n = 9

**Compound**[1]	**Compound descriptor**	**Microsomal Stability [%Qh]**[2]			**Hepatocyte [%Qh]**[3]			**MIC [µM]**[4]		
		Mice	Rat	Human	Mice	Rat	Human	*Mtb*	*Msm* 607	*Msm* 700084
SI-38	[3-OPra]	73	59	84	15	37	19	1.6$_a$	25.0$_a$	12.5$_a$
SI-64	[6-Me][3-Opra]		n.d.		34	50	22		n.d.	
**SI-40**	**[3,3-Tri][5]**		n.d.		15	12	n.d.	0.2$_a$	>50$_a$	>50$_a$
**SI-66**	**[6-Me][3,3-Tri][5]**		n.d.		15	10	12		n.d.	
**SI-39**	**[3,3-Tri][14-O3,6,9,12]**		n.d.		15	6	12	0.2$_a$	12.5$_a$	12.5$_a$
**SI-65**	**[6-Me][3,3-Tri][14-O3,6,9,12]**		n.d.			n.d.			n.d.	
14	[3-Allyl]	>88	84	>88	29	57	41	3.1$_a$	1.6$_a$	12.5$_a$
**17**	[3,3][4-E2]	<24	<23	<24	21	12	n.d.		n.d.	
10	[7-Pra]	>88	85	>88	64	70	12	3.1 ± 0.0$_b$	3.1 ± 0.0$_b$	25.0 ± 0.0$_c$
20	[6-Me][7-Pra]	>88	>88	>88	59	63	34	50.0 ± 0.0$_d$	25.0 ± 0.0$_d$	>50$_d$
24	[7-Tri][5]		n.d.		35	33	n.d.		n.d.	
25	[6-Me][7-Tri][5]	82	62	>88	21	16	30	6.3$_a$	12.5$_a$	12.5$_a$
SI-74	[7-Nme2Glu]	84	85	61	38	49	12	3.1 ± 0.0$_d$	0.8 ± 0.0$_d$	9.4 ± 4.4$_d$
SI-76	[6-Me][7-Nme2Glu]	>88	78	>88	23	52	n.d.	6.3$_a$	12.5$_a$	25.0$_a$
**12**	**[7,7-Tri][5]**	<24	<23	<24	15	6	12	0.1 ± 0.0$_d$	0.9 ± 0.3$_c$	>50$_c$
**22**	**[6-Me][7,7-Tri][5]**		n.d.		15	10	12	3.1 ± 0.0$_d$	2.3 ± 1.1$_d$	>50$_d$
**11**	**[7,7-Tri][8-O3,6]**	<24	<23	<24	15	6	12	0.1 ± 0.0$_d$	1.6 ± 0.0$_d$	1.6 ± 0.0$_e$
**21**	**[6-Me][7,7-Tri][8-O3,6]**		n.d.		15	6	n.d.		n.d.	
SI-72	[3-Phe][6-propargyl]	>88	87	>88	71	82	12		n.d.	
5	[6-Pra]	>88	>88	>88	58	51	12	1.6$_a$	25.0$_a$	6.3$_a$
26	[6-Me][6-Pra]	>88	>88	>88	67	68	n.d.	6.3$_a$	6.3$_a$	12.5$_a$
13	[6-allyl]	>88	>88	>88	59	65	19		n.d.	
SI-60	[6-Me][6-allyl]		>88	>88	61	57	50		n.d.	
**15**	**[6,6][4-E2]**	<24	<23	<24	15	6	12	0.2 ± 0.1$_e$	2.3 ± 0.9$_e$	50.0 ± 0.0$_e$
**28**	**[6-Me][6,6][4-E2]**		n.d.		15	6	n.d.		n.d.	
**16**	**[6,6][4]**		n.d.			n.d.			n.d.	
**SI-61**	**[6-Me][6,6][4]**		n.d.		15	6	n.d.		n.d.	
**9**	**[6,6-Tri][5]**	25	<23	43	15	6	12	0.3 ± 0.1$_d$	0.8 ± 0.0$_d$	>50$_e$
**SI-57**	**[6-Me][6,6-Tri][5]**		n.d.		15	8	12	n.d.	n.d.	n.d.
**6**	**[6,6-Tri][5-O3]**	43	<23	70	15	6	12	0.4 ± 0.3$_e$	2.3 ± 0.9$_e$	20.8 ± 15.1$_f$
**7**	**[6,6-Tri][11-O3,6,9]**	53	40	88	n.d.	n.d.	n.d.	0.1 ± 0.0$_e$	1.4 ± 0.2$_e$	n.d.
**8**	**[6,6-Tri][14-O3,6,9,12]**	72	40	>88	32	6	12	0.4 ± 0.4$_e$	1.9 ± 0.9$_g$	8.3 ± 5.1$_h$
**27**	**[6-Me][6,6-Tri][14-O3,6,9,12]**	77	41	>88	15	6	12	<0.1$_a$	1.6$_a$	0.8$_a$
dCymC		56	38	66	46	55	36	0.4 ± 0.1$_e$	2.8 ± 0.4$_e$	n.d.
23	[6-Me] dCymC		n.d.		15	57	13		n.d.	

[1] bold compounds/descriptors: Homo-BacPROTACs; [2] Clearance in microsomes [3] Clearance in hepatocytes; [4] Minimum inhibitory concentrations against *Mtb* H37Rv, *Msm* 607 and *Msm* 700084 determined as described above; [%Qh] = clearance stated as percent related to hepatic blood flow.

Supplementary Table 3. Summary of IV PK studies in BALB/cAnNCrl mice with 1 mg kg^{-1} bolus administration

Compound	C_{Max}[1]	AUC[2]	$T_{1/2}$[3]	VSS[4]	Cl[5]
**5**	415	164	1.03	4.54	98.8
**6**	1820	4490	3.33	0.95	3.73
**8** (UdsBI-0545)	6710	8580	6.74	0.48	1.90
**12** (UdSBI-4377)	3270	3870	1.37	0.42	4.14

[1]dose-normalized maximum plasma concentration (nM/(nmol kg^{-1})); [2] Dose-normalized area under plasma concentration curve (h nM/(nmol kg^{-1})); [3] Half-life in plasma (h); [4] Intravenous volume of distribution at steady state (l kg^{-1}); [5] Plasma clearance (ml min^{-1} kg^{-1})).

Supplementary Table 4. Individual and mean plasma concentrations (nmol l^{-1}) and pharmacokinetic parameters of compound **5** after single i.v. (bolus) administration to mice.

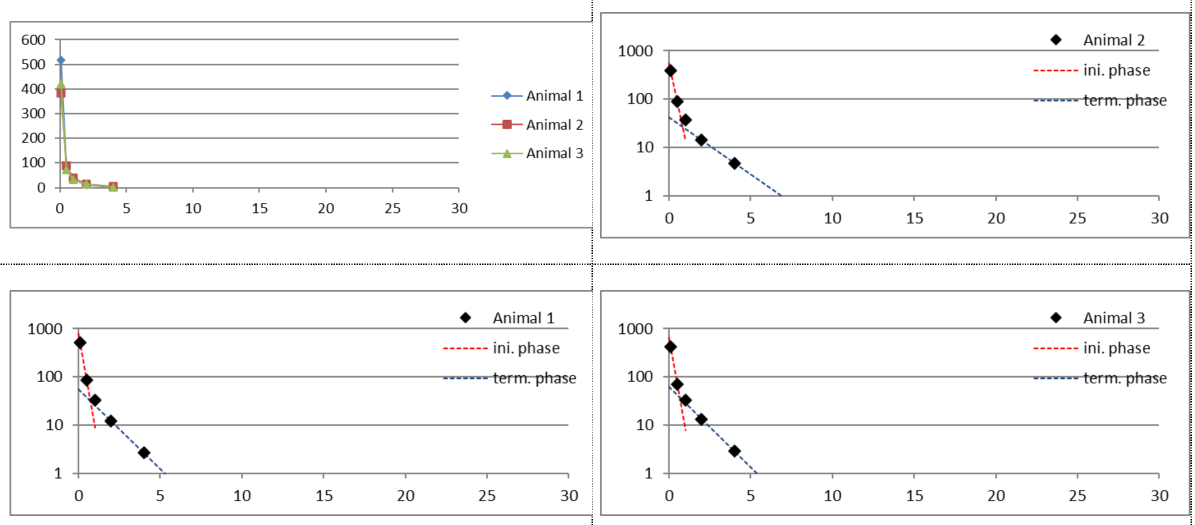

Administration		iv (bolus)				
Nominal Dose	µmol kg^{-1}	1.062				
Matrix		Plasma				
**Time/Parameter**	**Unit**	**Concentration (nmol l^{-1})**				
		**Animal 1**	**Animal 2**	**Animal 3**	**Mean**	**SD**
0.1	h	518	384	421	441	69
0.5	h	85.1	88.1	71.5	81.6	8.9
1	h	33.6	37.9	32.6	34.7	2.8
2	h	12.3	14.2	13.4	13.3	1.0
4	h	2.7	4.8	2.9	3.5	1.2
6	h	BLOQ	BLOQ	BLOQ	BLOQ	NC
24	h	BLOQ	BLOQ	BLOQ	BLOQ	NC
C_{max}/Dose	nM/(nmol kg^{-1})	488	361	396	415	65
AUC_{0-t}/Dose	h nM/(nmol kg^{-1})	177	161	154	164	12
AUC_{0-inf}/Dose	h nM/(nmol kg^{-1})	180	169	158	169	11
$t_{1/2}$	h	0.9	1.3	0.9	1.0	0.2
MRT	h	0.6	0.9	0.7	0.8	0.2
V_{ss}	l kg^{-1}	3.5	5.6	4.6	4.5	1.0
CL	ml min^{-1} kg^{-1}	92.3	98.5	106.	98.8	6.7
CL PL QH	%	103.0	109.0	117.0	110.0	7.4

Supplementary Table 5. Individual and mean plasma concentrations (nmol l^{-1}) and pharmacokinetic parameters of compound **6** after single i.v. (bolus) administration to mice.

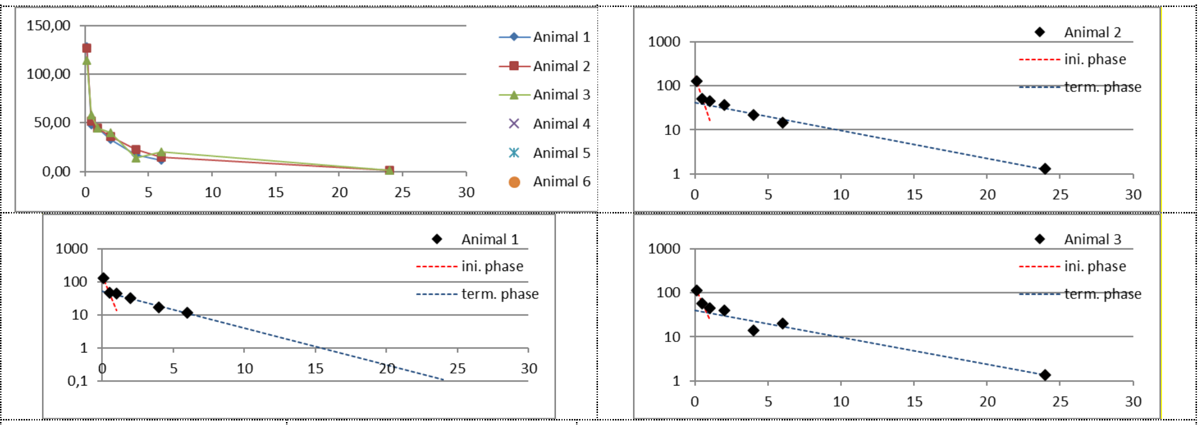

Administration		iv (bolus)				
Actual Dose	µmol kg^{-1}	0.0677				
Matrix		Plasma				
**Time/Parameter**	**Unit**	**Concentration (nmol l^{-1})**				
		**Animal 1**	**Animal 2**	**Animal 3**	**Mean**	**SD**
0.1	h	128	127	114	123	8
0.5	h	47.9	51.6	58	52.5	5.1
1	h	44.8	45.0	44.5	44.8	0.3
2	h	32.5	36.4	39.6	36.2	3.6
4	h	17.3	22.2	13.8	17.8	4.2
6	h	11.6	14.7	20.6	15.6	4.6
24	h	BLOQ	1.3	1.4	1.3	NC
t_{max}	h	0.1	0.1	0.1	0.1	0
C_{max}	nmol l^{-1}	128	127	114	123	8
AUC_{0-t}/Dose	h nM/(nmol kg^{-1})	2600	5020	5730	4450	1640
AUC_{0-inf}/Dose	h nM/(nmol kg^{-1})	3260	5150	5870	4760	1350
$t_{1/2}$	h	2.7	4.8	4.9	4.1	1.3
MRT	h	3.6	5.1	5.3	4.7	0.9
V_{ss}	l kg^{-1}	1.1	1.0	0.9	1.0	0.1
CL	ml min^{-1} kg^{-1}	5.1	3.2	2.8	3.7	1.2
CL PL QH	%	5.7	3.6	3.2	4.1	1.4

Supplementary Table 6. Individual and mean plasma concentrations (nmol l^{-1}) and pharmacokinetic parameters of compound **8** (UdSBI-0545) after single i.v. (bolus) administration to mice.

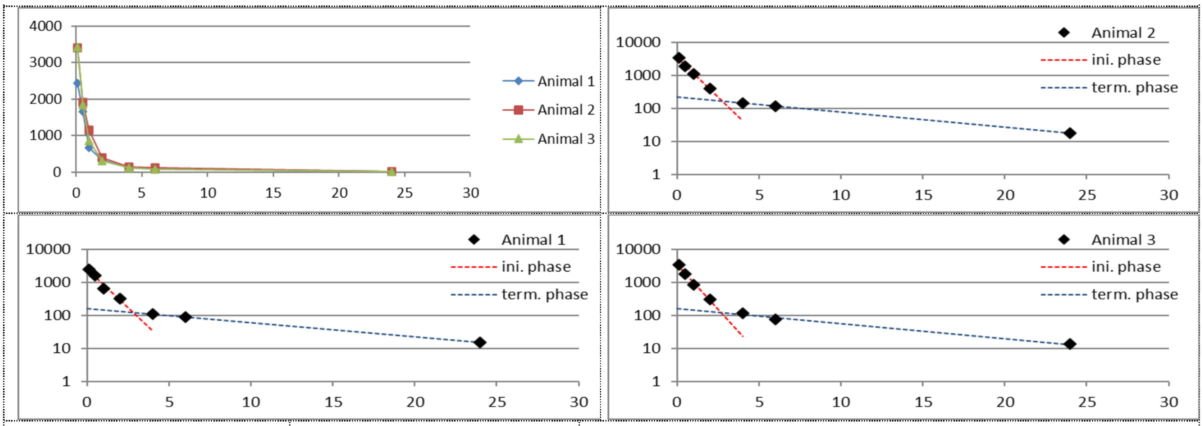

Administration		iv (bolus)				
Nominal dose	µmol kg^{-1}	0.461				
Matrix		Plasma				
**Time/Parameter**	**Unit**	**Concentration (nmol l^{-1})**				
		**Animal 1**	**Animal 2**	**Animal 3**	**Mean**	**SD**
0.1	h	2440	3410	3420	3090	563
0.5	h	1650	1900	1850	1800	132
1	h	655	1140	855	883	244
2	h	328	402	307	346	50
4	h	110	144	117	124	18
6	h	90.8	118	77.8	95.5	20.5
24	h	14.8	18.2	13.3	15.4	2.5
C_{max}/Dose	nM/(nmol kg^{-1})	5300	7400	7420	6710	1220
AUC_{0-t}/Dose	h nM/(nmol kg^{-1})	7540	10000	8140	8580	1310
AUC_{0-inf}/Dose	h nM/(nmol kg^{-1})	7860	10400	8420	8900	1350
$t_{1/2}$	h	6.9	6.7	6.6	6.7	0.1
MRT	h	4.5	4.3	3.8	4.2	0.4
V_{ss}	l kg^{-1}	0.6	0.4	0.5	0.5	0.1
CL	ml min^{-1} kg^{-1}	2.1	1.6	2.0	1.9	0.3
CL PL QH	%	2.4	1.8	2.2	2.1	0.3

Supplementary Table 7. Individual and mean plasma concentrations (nmol l^{-1}) and pharmacokinetic parameters of compound **12** (UdSBI-4377) after single i.v. (bolus) administration to mice.

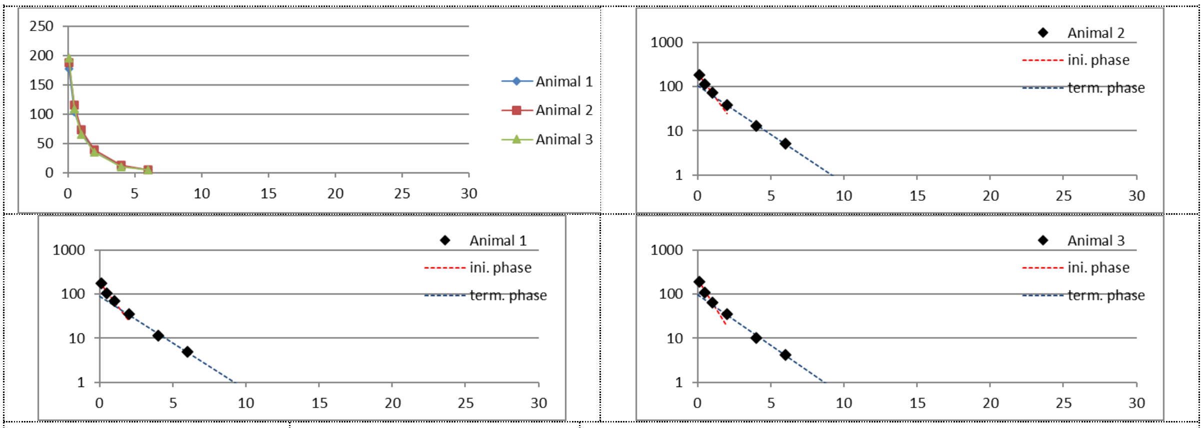

**Administration**		**iv (bolus)**				
Actual Dose	µmol kg^{-1}	0.0571				
Matrix		Plasma				
**Time/Parameter**	**Unit**	**Concentration (nmol l^{-1})**				
		**Animal 1**	**Animal 2**	**Animal 3**	**Mean**	**SD%**
0.1	h	177	187	196	187	9
0.5	h	104	115	109	109	6
1	h	70.4	72.8	64.7	69.3	4.1
2	h	35.2	38.5	35.3	36.3	1.9
4	h	11.4	12.9	10.3	11.5	1.3
6	h	5.0	5.1	4.3	4.8	0.4
24	h	BLOQ	BLOQ	BLOQ	BLOQ	NC
C_{max}/Dose	nM/(nmol kg^{-1})	3100	3280	3440	3270	167
AUC_{0-t}/Dose	h nM/(nmol kg^{-1})	3780	4070	3760	3870	175
AUC_{0-inf}/Dose	h nM/(nmol kg^{-1})	3960	4250	3900	4040	185
$t_{1/2}$	h	1.4	1.4	1.3	1.4	0.1
MRT	h	1.7	1.7	1.6	1.7	0.1
V_{ss}	l kg^{-1}	0.4	0.4	0.4	0.4	0.1
CL	ml min^{-1} kg^{-1}	4.2	3.9	4.3	4.1	0.2
CL PL QH	%	4.7	4.4	4.8	4.6	0.2

Note: Mean PK parameters were calculated by mean plasma concentrations.

BLOQ: Below lower limit of quantification (<1.00 nmol/l).

NC: Not calculated.

Supplementary Table 8. *M. smegmatis* and *M. tuberculosis* ClpC1P1P2 in cell-free degradation assays. Footnotes indicate the number of independent experiments performed for the respective compound: a) n = 4, b) n = 5, c) n = 3, d) n = 6, e) n = 2

Compound	Value	*M.smegmatis* ClpC1P1P2		*M.tuberculosis* ClpC1P1P2	
		Mean	*SD*	*Mean*	*SD*
**dCymC**	DC_{50} [μM]	> 100.0_a		> 100.0_e	
**5**	DC_{50} [μM]	> 100.0_a		> 100.0_e	
**6**	DC_{50} [μM]	7.5_a	4.6	5.8_a	2.9
	D_{Max}	78%	9%	54%	10%
**8** (UdSBI-0545)	DC_{50} [μM]	8.0_b	1.2	8.3_c	3.9
	D_{Max}	83%	6%	54%	4%
**8a** (UdSBI-0966)	DC_{50} [μM]	> 100.0_a		> 100.0_e	
**9**	DC_{50} [μM]	8.0_c	1.2	6.8_c	2.2
	D_{Max}	54%	2%	26%	5%
**10**	DC_{50} [μM]	> 100.0_b		> 100.0_e	
**11**	DC_{50} [μM]	5.6_a	1.5	5.1_e	0.5
	D_{Max}	87%	3%	74%	6%
**12** (UdSBI-4377)	DC_{50} [μM]	8.4_d	1.8	7.1_c	2.6
	D_{Max}	81%	7%	52%	5%
**12a** (UdsBI-0117)	DC_{50} [μM]	> 100.0_b		> 100.0_e	
**16**	DC_{50} [μM]	7.0_a	0.9	10.7_c	4.6
	D_{Max}	57%	5%	47%	10%
**17**	DC_{50} [μM]	> 100.0_a		> 100.0_e	
**21**	DC_{50} [μM]	5.5_a	1.1	8.6_c	1.2
	D_{Max}	92%	4%	84%	6%
**27**	DC_{50} [μM]	12.5_c	5.0	24.7_a	14.6
	D_{Max}	91%	2%	74%	4%
**SI-39**	DC_{50} [μM]	5.8_c	0.6	7.7_c	1.6
	D_{Max}	88%	8%	73%	7%
**SI-57**	DC_{50} [μM]	23.0_a	9.7	23.7_c	6.8
	D_{Max}	67%	7%	55%	12%
**SI-61**	DC_{50} [μM]	5.3_a	0.5	7.2_e	0.2
	D_{Max}	42%	3%	42%	7%
**SI-65**	DC_{50} [μM]	5.2_c	0.8	8.0_e	1.4
	D_{Max}	92%	5%	84%	1%

Supplementary Table 9. MICs of Homo-BacPROTACs and monomers against Gram positive and Gram negative bacteria (ESKAPES panel)

Compound	Gram positive		Gram negative				
	Enterococcus faecium	*Staphylococcus aureus*	*Klebsiella pneumoniae*	*Acinetobacter baumanii*	*Pseudomonas aeruginosa*	*Enterobacter cloacae*	*Salmonella typhimurium*
	**µM**	**µM**	**µM**	**µM**	**µM**	**µM**	**µM**
**5**	>50.0	>50.0	>50.0	>50.0	>50.0	>50.0	>50.0
**8** (UdSBI-0545)	>50.0	>50.0	>50.0	>50.0	>50.0	>50.0	>50.0
**10**	>50.0	>50.0	>50.0	>50.0	>50.0	>50.0	>50.0
**12** (UdSBI-4377)	>50.0	>50.0	>50.0	>50.0	>50.0	>50.0	>50.0
Moxifloxacin	79.7 ± 0.0	0.3 ± 0.0	1.3 ± 0.0	0.3 ± 0.0	2.5 ± 0.0	0.3 ± 0.0	0.6 ± 0.0

Values represent means and standard deviations of n = 3 well replicates.

Supplementary Table 10. MICs of Homo-BacPROTACs and monomers against non-tuberculous Mycobacteria.

Means and standard deviations of n = 2 independent experiments (one in triplicate, one as unicate) are shown. Values marked with * represent means and standard deviations of n = 3 well replicates from a single experiment.

Compound	***Mycobacterium avium***	***Mycobacterium abscessus***	***Mycobacterium fortuitum***	***Mycobacterium intracellulare***
	**µM**	**µM**	**µM**	**µM**
**10**	12.5 ± 0.0	12.5 ± 0.0	12.5 ± 0.0	12.5 ± 0.0
**12** (UdSBI-4377)	>50.0	>50.0	>50.0	>50.0
**5**	6.3 ± 0.0	5.5 ± 1.6	6.3 ± 0.0	6.3 ± 0.0
**8** (UdSBI-0545)	>50.0	>50.0	>50.0	>50.0
Moxifloxacin	2.5 ± 0.0	2.5 ± 0.0	0.1 ± 0.0	1.2 ± 0.0
Rifampicin	0.3 ± 0.0*	9.7 ± 0.0*	1.2 ± 0.0*	0.2 ± 0.0*
Amikacin	1.7 ± 0.0*	3.4 ± 0.0*	0.4 ± 0.0*	0.9 ± 0.0*
Clarithromycin	0.3 ± 0.0*	0.7 ± 0.0*	0.7 ± 0.0*	0.2 ± 0.0*

Supplementary Table 11: Homo-BacPROTACs are bactericidal on Mtb, but not Msm cells**.**

Homo-BacPROTACs **8** (UdSBI-0545) and **12** (UdSBI-4377) display bactericidal activity in *Mtb* H37Rv, but not in *Msm* #607, as demonstrated by replating all respective cultures from one of the triplicate set of wells showing growth inhibition in the MIC assay plate. MBC plating was conducted onto compound-free plates to monitor any colony formation indicative of survivors. An individual example is shown. See Source Data file for details.

	Msm #607				***Mtb* H37Rv**			
**Compound**	**MIC*** ***Msm* #607**		**MBC**** ***Msm* #607**		**MIC*** ***Mtb* H37Rv**		**MBC**** ***Mtb* H37Rv**	
	**µM**	**µg/ml**	**µM**	**µg/ml**	**µM**	**µg/ml**	**µM**	**µg/ml**
**10**	6.3	5.7	>50.0	>45.7	3.1	2.9	12.5	11.4
**12** (UdSBI-4377)	0.8	1.5	>50.0	>99.0	0.05	0.1	0.1	0.2
**5**	25.0	23.5	>50.0	>47.1	1.6	1.5	6.3	5.9
**8** (UdSBI-0545)	1.6	3.4	>50.0	>108.5	0.1	0.2	0.1	0.2
Rifampicin	9.7	8.0	ND		0.01	0.01	ND	
Moxifloxacin	0.1	0.1	0.3	0.1	0.1	0.03	0.3	0.1

MIC*: Minimum Inhibitory Concentration

MBC**: Minimum Bactericidal Concentration

Supplementary Fig. 1. MIC on Mtb Beijing strain HN878.

The MIC for the hypervirulent *Mtb* Beijing strain HN878 was determined using the Resazurin assay described above. Homo-BacPROTACs **12** (UdSBI-4377) and **8** (UdSBI-0545) show more efficient inhibition of bacterial growth as compared to the matching monomers **10** and **5** , respectively. Error bars indicate mean ± SD of n = 3 well replicates.

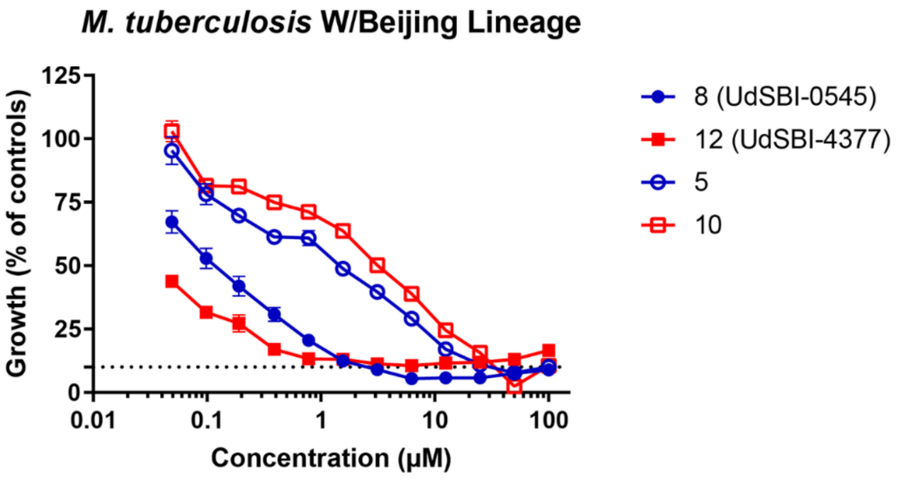

Supplementary Fig. 2. MIC on replicating versus starvation-induced, dormant *Mtb* H37Rv cells.

Starvation-induced non-replicating cells of *Mtb* are known to become highly tolerant to a multitude of clinical drugs including the TB antibiotics isoniazid, rifampicin, streptomycin and moxifloxacin[1]. In the used model, we observed strongly reduced susceptibility of all compounds tested, with bedaquiline showing at least partial remaining activity. Error bars indicate mean ± SD of n = 3 well replicates.

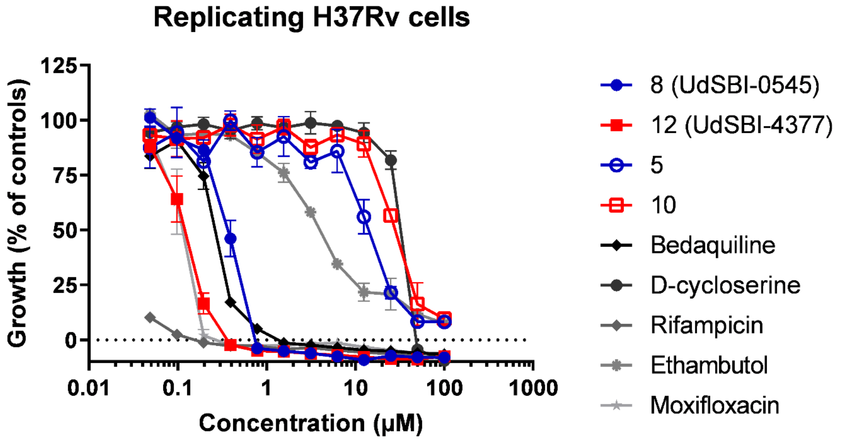

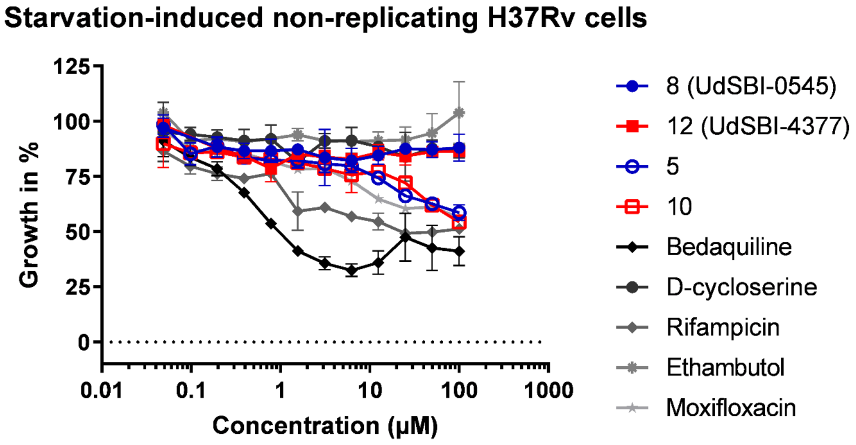

Supplementary Fig. 3. BacPROTAC mediated degradation of ClpC1-NTD over time.

Homo-BacPROTAC-mediated cell-free degradation of ClpC1 NTD by the *M. smegmatis* ClpC1P1P2 complex monitored over different incubation times (quantified by WES capillary Western platform). Degradation induced by compound **8** was monitored over six hours at two concentrations (8 µM, 33 µM) at experimental conditions otherwise kept constant and described in the Methods section “Cell-free degradation assay”. Means ± SDs of n = 2 well replicates are shown for one representative experiment. The experiment was independently performed twice with similar results. For comparative profiling of compounds in this assay, a 90 min incubation time was chosen.

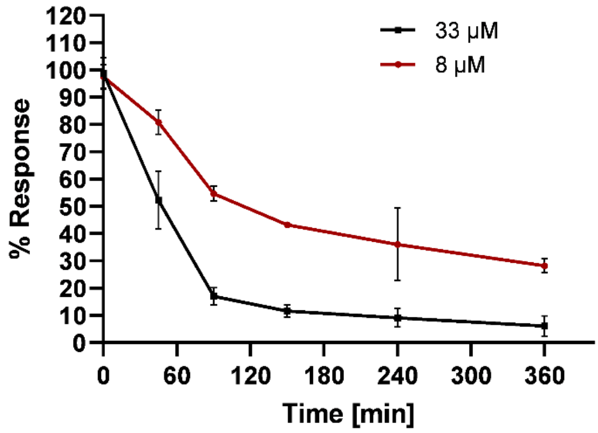

Investigation of Intramolecular Hydrogen Bonds (IMHB)

The NMR assignment of N-H protons in **10** was accomplished using ^{1}H-^{1}H-COSY, ^{1}H-^{13}C HSQC and HMBC experiments (for copies of NMR spectra, see below).

N-H acidity coefficient

The difference in ^{1}H NMR chemical shifts in $CDCl_3$ and DMSO-d_6 can be used to approximate the acidity of N-bonded protons.[2] The acidity A_{NMR} can be calculated by the following equation:

$A_{NMR} = 0.0065 + 0.133\ \Delta\delta;\ \Delta\delta = \delta(\text{DMSO-d}_6) - \delta(CDCl_3)$

For $A_{NMR} > 0.15$, no IMHB is present, for $A_{NMR} < 0.05$, a strong IMHB can be assumed.

Supplementary Table 12. Chemical shifts and acidity coefficients for N-H protons in **10**

	position	δ(DMSO-d$_6$) (ppm)	δ($CDCl_3$) (ppm)	Δδ (ppm)	A_{NMR}	IMHB
Val-NH	**2**	9.13	8.08	1.05	0.146	none
β-OMe-Phe-NH	**3**	7.50	7.27	0.23	0.037	strong
Ala-NH	**4**	8.27	8.46	-0.19	-0.019	strong
Trp-NH	**6**	9.37	7.8	1.57	0.215	none
Pra-NH	**7**	8.13	8.23	-0.1	-0.007	strong

It can be assumed that Val-NH and Trp-NH are not involved in IMHB ($A_{NMR} \geq 0.15$). β-OMe-Phe-NH, Ala-NH and Pra-NH all show values <0.05, indicating that these groups are involved in a strong IMBH network.

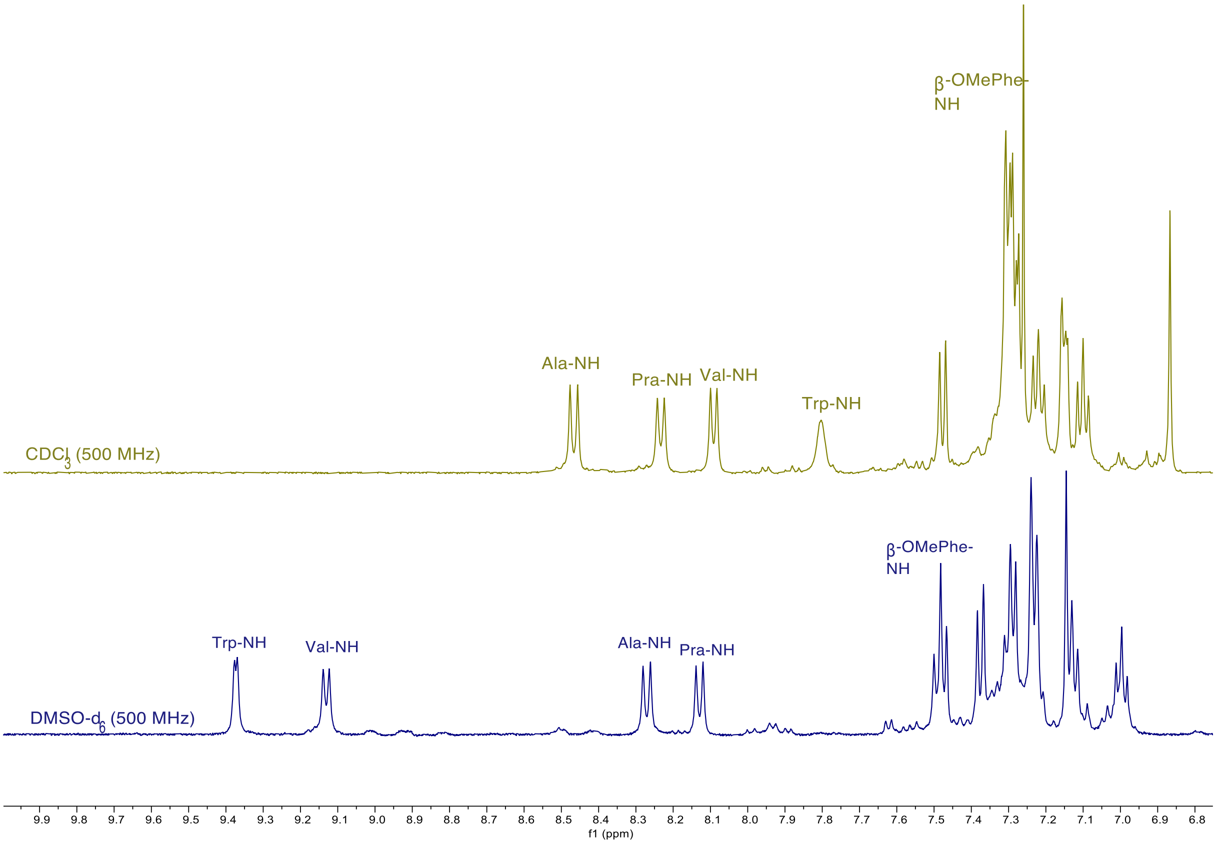

Supplementary Fig. 4. Excerpt from the ^{1}H spectra of **10** in $CDCl_3$ (top, yellow) and DMSO-d_6 (bottom, blue).

NMR Temperature Coefficients

In VT-NMR experiments in DMSO-d_6, the chemical shifts of the N-H protons were determined at different temperatures from 298 K to 338 K in 10 K steps. Linear regression affords the slope of the chemical shifts (Δδ (ppb K^{-1})). These slopes indicate wether the proton is solvent-exposed or part of the IMHB network. Empirically, coefficients < –4.6 ppb K^{-1} indicate a solvent exposed NH, whereas values ≥ –4.6 ppb K^{-1} indicate IMHB interactions.[3] In **10**, the coefficients can be classified in two groups (Table S7; Fig. S2). One group of protons show Δδ values between -2.31 and -1.01 ppb K^{-1} and the other group between -6.76 and -4.59 ppb K^{-1}. It is therefore likely that the first group (β-OMe-Phe, Ala, Pra) is involved in IMHB, whereas the latter group (Val, Trp) is not. This is in accordance with the IMHB network observed by A_{NMR} coefficients as well as the IMHB network in the crystal structure of ClpC1-NTD in complex with Cym A (Fig. S3).

Supplementary Table 13. ^{1}H NMR Chemical shifts (in ppm) of N-H protons in **10** at different temperatures.

AA	position	298 K	308 K	318 K	328 K	338 K	Δδ (ppb K^{-1})	IMHB?
Val-NH	**AA2**	9.13	9.053	8.99	8.925	8.856	-6.76	solvent exposed
β-OMe-Phe-NH	**AA3**	7.483	7.464	7.438	7.415	7.392	-2.31	IMHB
Ala-NH	**AA4**	8.263	8.245	8.225	8.204	8.182	-2.03	IMHB
Trp-NH	**AA6**	9.359	9.321	9.276	9.228	9.176	-4.59	solvent exposed
Pra-NH	**AA7**	8.125	8.117	8.107	8.096	8.085	-1.01	IMHB

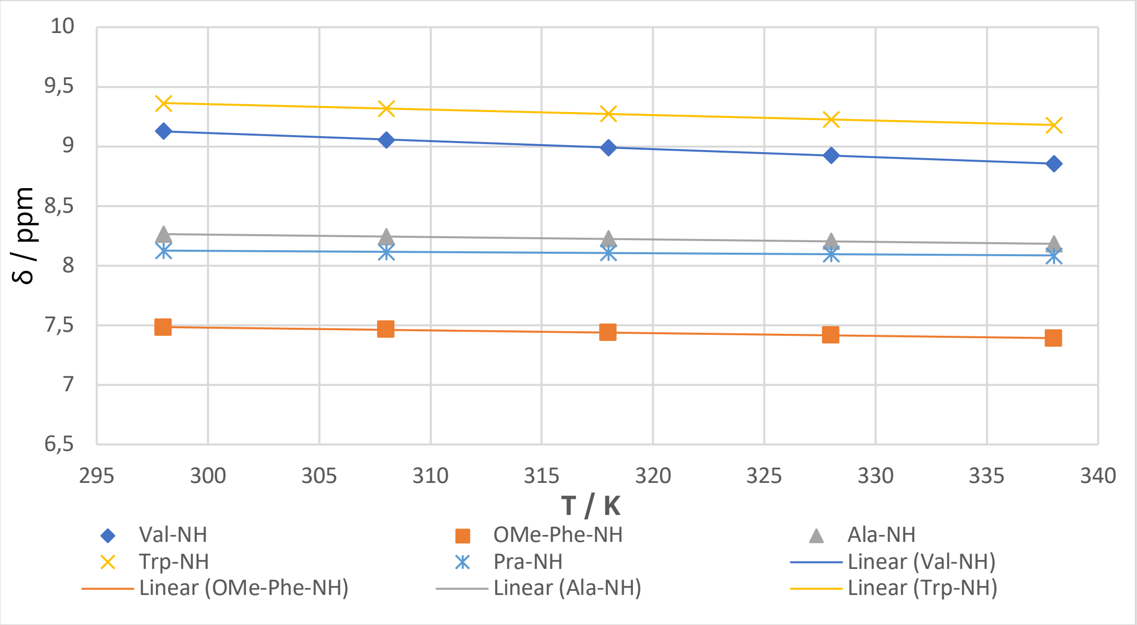

Supplementary Fig. 5. Linear regression of δ(NH)/ppm vs. T/K; *Val-NH*: y = -0.00676x + 11.14; R^2 = 0.9989; *β-OMe-Phe-NH*: y = -0.00231x + 8.173; R^2 = 0.9983; *Ala-NH*: y = -0.00203x + 8.8693; R^2 = 0.9986; *Trp-NH*: y = -0.00459x + 10.732; R^2 = 0.9967; *Pra-NH*: y = -0.00101x + 8.4272; R^2 = 0.9962.

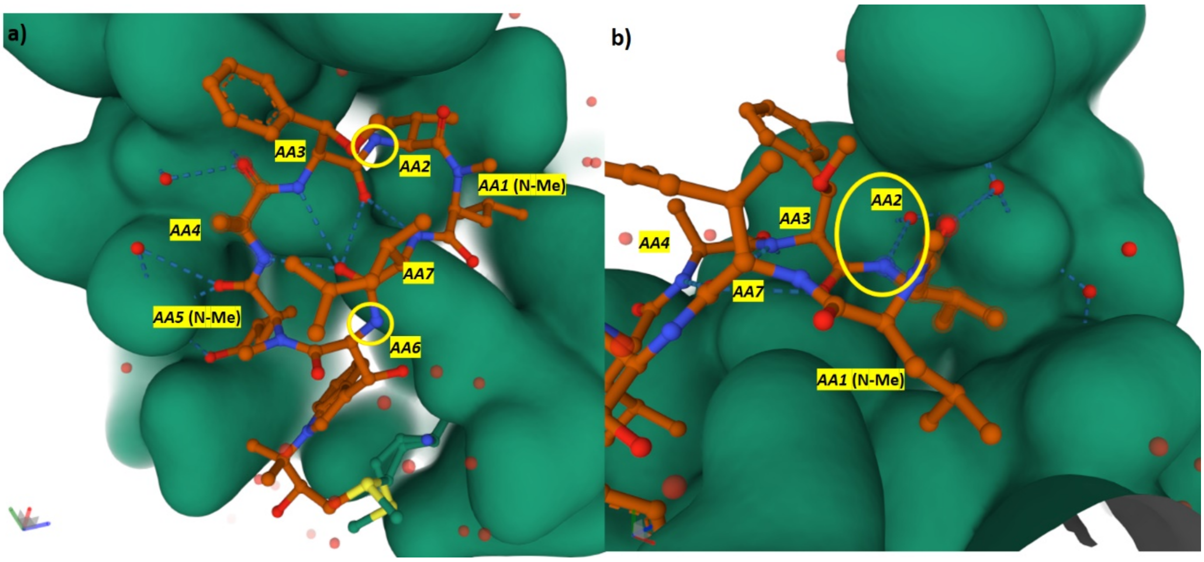

Supplementary Fig. 6. a) IMHB interactions in the crystal structure of CymA bound to ClpC1 NTD (https://www.rcsb.org/structure/3WDC). Yellow circles indicate the two N-H atoms which were determined to be solvent exposed. **b)** The yellow circle indicates a hydrogen bonding network between Val(*AA2*)-N-H, an H_2O-molecule and Gln17/Ile18.

Supplementary Tables 14-17. Pharmacokinetic data after oral administration

Supplementary Table 14. Individual and mean plasma concentrations (nmol l^{-1}) and pharmacokinetic parameters of compound **8** (UdSBI-0545) after single oral gavage administration to mice.

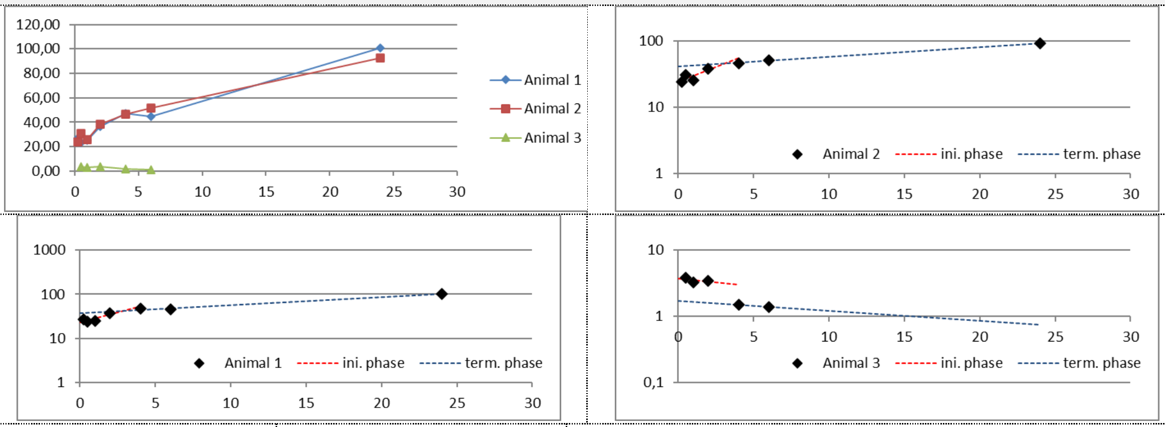

Administration		oral (gavage)				
Dose	µmol/kg	9.30				
Matrix		Plasma				
**Time/Parameter**	**Unit**	**Concentration (nmol/L)**				
		**Animal 1**	**Animal 2**	**Animal 3**	**Mean**	**SD**
0.25	h	27.2	24.1	BLOQ	25.7	2.19
0.5	h	23.4	31.1	3.80	19.4	14.1
1	h	25.2	25.8	3.24	18.1	12.9
2	h	36.5	38.2	3.41	26.0	19.6
4	h	47.4	46.6	1.48	31.8	26.3
6	h	44.5	51.7	1.38	32.5	27.2
24	h	101	92.4	NR	96.7	6.08
t_{max}	h	24.0	24.0	0.500 1)	24.0	0.00
C_{max}/Dose	nM/nmol/kg	10.9	9.95	0.409 1)	10.4	0.655
AUC_{0-t}/Dose	h*nM/nmol/kg	165	165	1.38 1)	165	0.114
AUC_{0-inf}/Dose	h*nM/nmol/kg	NC	NC	NC	NC	NC
$t_{1/2}$	h	NC	NC	NC	NC	NC
MRT	h	NC	NC	NC	NC	NC
BA	%				NC	

BLOQ: Below lower limit of quantification (<1.00 nmol/L).

NC: Not calculated.

NR: No results, due to missing sample.

1) Value excluded from mean and SD

Supplementary Table 15. Individual and mean plasma concentrations (nmol l^{-1}) and pharmacokinetic parameters of compound **12** (UdSBI-4377) after single oral gavage administration to mice.

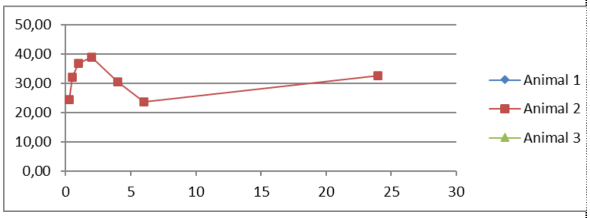

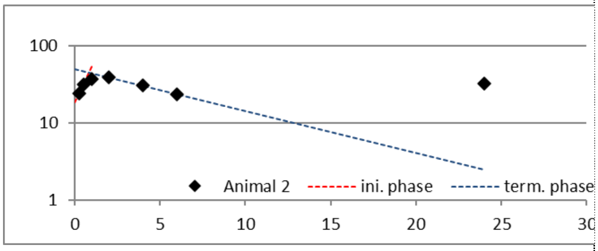

**Administration**		**oral (gavage)**				
Dose	µmol/kg	15.1				
Matrix		Plasma				
**Time/Parameter**	**Unit**	**Concentration (nmol/L)**				
		**Animal 1**	**Animal 2**	**Animal 3**	**Mean**	**SD**
0.25	h	BLOQ	24.5	BLOQ	NC	NC
0.5	h	BLOQ	32.0	BLOQ	NC	NC
1	h	BLOQ	36.8	BLOQ	NC	NC
2	h	BLOQ	38.8	BLOQ	NC	NC
4	h	BLOQ	30.5	BLOQ	NC	NC
6	h	BLOQ	23.5	BLOQ	NC	NC
24	h	BLOQ	32.5	BLOQ	NC	NC
t_{max}	h	NC	2.00	NC	NC	NC
C_{max}/Dose	nM/nmol/kg	NC	2.56	NC	NC	NC
AUC_{0-t}/Dose	h*nM/nmol/kg	NC	45.5	NC	NC	NC
AUC_{0-inf}/Dose	h*nM/nmol/kg	NC	62.6	NC	NC	NC
$t_{1/2}$	h	NC	5.53	NC	NC	NC
MRT	h	NC	18.0	NC	NC	NC
BA	%		0.7			

BLOQ: Below lower limit of quantification (<1.00 nmol/L).

NC: Not calculated.

Supplementary Table 16. Individual and mean plasma concentrations (nmol l^{-1}) and pharmacokinetic parameters of compound **5** after single oral gavage administration to mice.

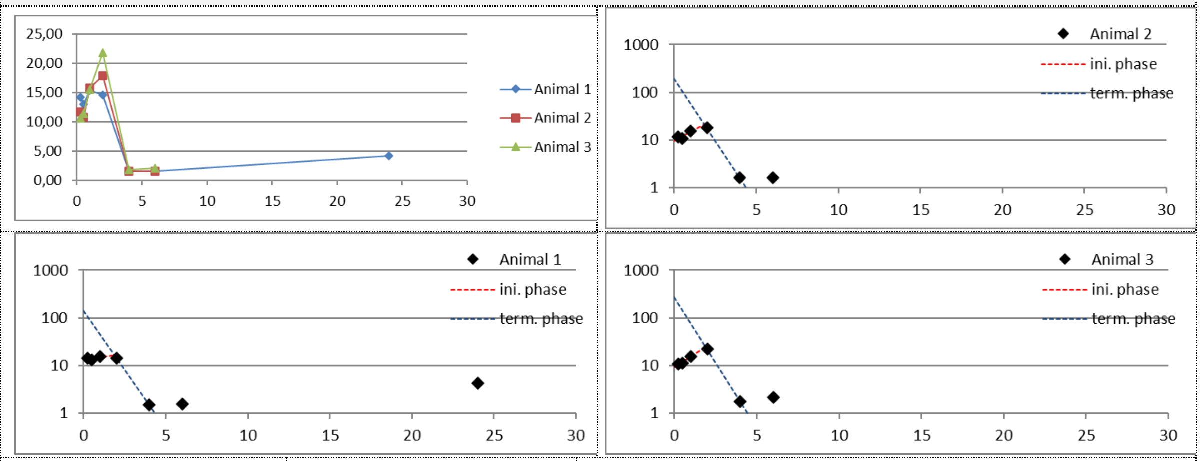

Administration		oral (gavage)				
Dose	µmol/kg	31.9				
Matrix		Plasma				
**Time/Parameter**	**Unit**	**Concentration (nmol/L)**				
		**Animal 1**	**Animal 2**	**Animal 3**	**Mean**	**SD**
0.25	h	14.2	11.7	10.6	12.2	1.85
0.5	h	13.0	10.7	11.4	11.7	1.18
1	h	15.3	15.7	15.5	15.5	0.200
2	h	14.5	17.8	21.8	18.0	3.66
4	h	1.49	1.61	1.76	1.62	0.135
6	h	1.54	1.59	2.14	1.76	0.333
24	h	4.22	BLOQ	BLOQ	4.22	
t_{max}	h	1.00	2.00	2.00	1.67	0.577
C_{max}/Dose	nM/nmol/kg	0.480	0.558	0.684	0.574	0.103
AUC_{0-t}/Dose	h*nM/nmol/kg	3.02	1.53	1.74	2.10	0.805
AUC_{0-inf}/Dose	h*nM/nmol/kg	3.14	1.57	1.80	2.17	0.845
$t_{1/2}$	h	0.609	0.577	0.551	0.579	0.0293
MRT	h	11.7	1.98	2.06	5.23	5.57
BA	%				1.2	

BLOQ: Below lower limit of quantification (<1.00 nmol/L).

NC: Not calculated.

Supplementary Table 17. Individual and mean plasma concentrations (nmol l^{-1}) and pharmacokinetic parameters of compound **6** after single oral gavage administration to mice were not available due to bad solubility of the compound (see data below).

Concentration time plots N/A

**Administration**		**oral (gavage)**				
Dose	µmol/kg	14.7				
Matrix		Plasma				
**Time/Parameter**	**Unit**	**Concentration (nmol/L)**				
		**Animal 1**	**Animal 2**	**Animal 3**	**Mean**	**SD**
0.25	h	BLOQ	BLOQ	BLOQ	BLOQ	NC
0.5	h	BLOQ	BLOQ	BLOQ	BLOQ	NC
1	h	BLOQ	BLOQ	BLOQ	BLOQ	NC
2	h	BLOQ	BLOQ	BLOQ	BLOQ	NC
4	h	BLOQ	BLOQ	BLOQ	BLOQ	NC
6	h	BLOQ	BLOQ	BLOQ	BLOQ	NC
24	h	BLOQ	BLOQ	BLOQ	BLOQ	NC
t_{max}	h	NC	NC	NC	NC	NC
C_{max}/Dose	nM/nmol/kg	NC	NC	NC	NC	NC
$AUC_{0\text{-}t}$/Dose	h*nM/nmol/kg	NC	NC	NC	<1.61[1)]	NC
$AUC_{0\text{-}inf}$/Dose	h*nM/nmol/kg	NC	NC	NC	NC	NC
$t_{1/2}$	h	NC	NC	NC	NC	NC
MRT	h	NC	NC	NC	NC	NC
BA	%				<0.04[1)]	

BLOQ: Below lower limit of quantification (<1.00 nmol/L).

NC: Not calculated.

1) Maximum potential value calculated considering 1 nM concentration (BLOQ) for each time point.

General Information (Chemistry)

Compounds **SI-1**–**SI-15**, **2a**, **SI-21**–**SI-34**, **SI-44**–**SI-49**, **SI-67** and **SI-68** were synthesized by Aragen Life Sciences.

All other compounds were synthesized at Saarland University by the authors. For these, the following applies:

All experiments were carried out in oven-dried glassware (round-bottom flasks or glass vials) under air if not stated otherwise. Anhydrous Dichloromethane (DCM), *N,N*-dimethylformamide (DMF), 1,4-dioxane and acetonitrile (MeCN) were purchased from Acros Organics and used without further purification. Cyclohexane (p.a. grade), Acetonitrile (HPLC grade) were purchased from Fisher Scientific. EtOAc and petroleum ether (PE) were distilled prior to use. Reactions were monitored by LC/MS (Shimadzu Prominence-*i* LC-2030, column: Phenomenex Onyx C18, 50 x 4.6 mm, Shimadzu LCMS-2020, ESI ionization) or by analytical TLC (Polygram SIL G/UV_{254} plates by Macherey-Nagel, visualization with UV-light (254 nm), $KMnO_4$, ninhydrin or cerium molybdate stains). Rotary evaporation was conducted at 40 to 50 °C.

The compounds were purified by automated flash chromatography (normal phase: Grace Reveleris, Teledyne Isco RediSep R_f cartridges, CyH/EtOAc gradient; reversed phase (RP): Büchi Reveleris PREP, Büchi Flashpure Select C18 cartridges, H_2O/MeCN gradient). Final compounds were additionally purified by preparative HPLC (Büchi Releveris PREP, column: Phenonemenex Luna C18, 5 µm, 21.2 x 250 mm, H_2O/MeCN gradient). The compounds were dried by lyophilization from MeCN/H_2O overnight.

NMR spectra were recorded in $CDCl_3$ (δH 7.26 ppm; δC 77.16 ppm) or DMSO-d_6 (δH 2.50 ppm; δC 39.52 ppm) on a Bruker Avance II 400 MHz spectrometer (5 mm BBO Probe, ^{1}H 400 MHz, ^{13}C 101 MHz, 298 K, standard pulse programs from TOPSPIN 3.2 software), a Bruker Avance I 500 MHz spectrometer (5 mm TCI Probe, ^{1}H 500 MHz, ^{13}C 126 MHz, 295 K, standard pulse programs from TOPSPIN 2.4 software), or a Bruker Avance Neo 500 MHz spectrometer (5 mm TCI Prodigy CryoProbe, ^{1}H 500 MHz, ^{13}C 126 MHz, 298 K, standard pulse programs from TOPSPIN 4 software). Chemical shifts (δ) are reported in parts per million (ppm) relative to $Si(CH_3)_4$. Multiplicities are reported as bs (broad signal), s (singlet), d (doublet), t (triplet), q (quartet) and m (multiplet). For mixtures of rotamers in which the signals could not be attributed to either rotamer, the observed integrations for each signal are reported.

High resolution mass spectra were recorded on a *Bruker MAXIS 4G* UHR-TOF (ESI) or a *Finnigan MAT 95* (CI).

Specific optical rotation was measured on a Jasco P-2000 polarimeter in a thermostated (20 °C ± 1 °C) cuvette (path length: 50 mm, λ = 589 nm). The concentrations are given in g/100 ml.

The purity of final compounds was determined via analytical HPLC (column: Luna 3 µm C18(2), 50x4.6 mm; flow: 1 ml min^{-1}; MeCN/H_2O gradient).

Starting materials prepared according to literature procedures:

2 (Cbz-protected pentapeptide);[4]
N^{α}-((allyloxy)carbonyl)-1-(prop-2-yn-1-yl)-L-tryptophan;[5]
methyl N-(((2S,3R)-2-((S)-2-((2S,4R)-2-((S)-2-(((allyloxy)carbonyl)amino)-N-methyl-3-(1-methyl-1H-indol-3-yl)propanamido)-5-((tert-butyldimethylsilyl)oxy)-4-methylpentanamido)propanamido)-3-methoxy-3-phenylpropanoyl)-L-valyl)-N-methyl-L-leucinate (Alloc-protected hexapeptide);[6]
*N*α-((allyloxy)carbonyl)-1-methyl-L-tryptophan;[5]
Allyloxycarbonyl-D-valine;[7]
(2S,4R)-2-(((benzyloxy)carbonyl)(methyl)amino)-5-((tert-butyldimethylsilyl)oxy)-4-methylpentanoic acid;[4]
*N*²-(((9*H*-fluoren-9-yl)methoxy)carbonyl)-*N*⁵,*N*⁵-dimethyl-L-glutamine;[8]
*N*α-((allyloxy)carbonyl)-1-(2-methylbut-3-en-2-yl)-L-tryptophan;[4]
(2*S*,3*R*)-2-(((allyloxy)carbonyl)amino)-3,5-dimethylhex-4-enoic acid.[4]

General Synthetic Procedures

The exact reagent amounts and reaction times used are provided in the following chapter.

***GP 1**: N-Cbz-deprotection*

To a solution of the Cbz-protected peptide (1.0 equiv) in MeOH (0.1 M) was added 10 wt% Pd/C (8 mol%). The flask was evacuated (water aspirator) and refilled with H_2 (from a balloon) five times. The reaction mixture was then stirred under H_2 (balloon pressure) until TLC indicated complete conversion (typically 1–2 h). Subsequently, the mixture was filtered over Celite, the filtrate was evaporated *in vacuo* and dried in high vacuum.

***GP 2**: N-Alloc-deprotection*

To a solution of the Alloc-protected peptide (1.0 equiv) in DCM (0.1 M) was added *N*,*N*-dimethylbarbituric acid (DMBA, 3.0 equiv) followed by $Pd(PPh_3)_4$ (0.03 equiv) (under N_2) and stirred until TLC indicated full conversion (typically after 1 h). The reaction mixture was then diluted with EtOAc and washed with sat. $NaHCO_3$ soln. (3x). The combined aqueous phases were back-extractred with EtOAc (1x), the combined organic phases were dried (Na_2SO_4) and evaporated *in vacuo*.

***GP 3:** Peptide coupling with BEP*

The *N*-deprotected peptide was dissolved in DCM (0.1 M) and the acid component (1.2 equiv) and 2-bromo-1-ethylpyridinium tetrafluoroborate (BEP) (1.2 equiv) were added. The mixture was cooled to –20 °C (ice/NaCl) and 4-methylmorpholine (NMM) (2.5 equiv) was added dropwise at this temperature. The reaction mixture was slowly warmed to room temperature overnight and then quenched by the addition of 1 M $KHSO_4$. The mixture was diluted with EtOAc (ca. 20 ml per mmol substrate), transferred to a separatory funnel and shaken vigorously. The phases were separated and the organic phase was subsequently washed with H_2O, sat. $NaHCO_3$ soln. and brine. The organic phase was dried (Na_2SO_4) and evaporated. The residue was purified by flash chromatography.

***GP 4:** Peptide coupling with HOBt/EDC*

The *N*-deprotected peptide was dissolved in DCM (0.1 M) and the acid component (typically 1.1 to 1.3 equiv) was added. The mixture was cooled to 0 °C and 4-methyl morpholine (NMM) (2.0 equiv), HOBt·H_2O (1.1 equiv) and EDC (1.1 equiv) were added. The mixture was slowly warmed to room temperature and then diluted with EtOAc and washed with 1 M $KHSO_4$ soln., H_2O, sat. $NaHCO_3$ soln. and brine. The organic phase was dried (Na_2SO_4) and evaporated *in vacuo*. The residue was purified by flash chromatography.

***GP 5**: Peptide coupling with NMI/HCl*[9]

The acid component (1.3 equiv) was dissolved in anhydrous 1,4-dioxane (0.3 M) under N_2-atmosphere. DIPEA (1.3 equiv) and $BnNMe_2$ (0.1 equiv) were added at rt. The mixture was heated to 60 °C and 1 M isopropyl chloroformate solution (1 M in toluene) (1.3 equiv) was added dropwise. After 5 min at 60 °C, a solution of the *N*-deprotected peptide (1.0 equiv), HCl (4 M in 1,4-dioxane) (0.2 equiv) and *N*-methylimidazole (0.1 equiv) in 1,4-dioxane/MeCN 1:1 (0.12 M) was added. The flask was rinsed with additional 1,4-dioxane (final reaction concentration 0.06 M). The reaction mixture was stirred at 60 °C for the specified time. The solvent was then evaporated *in vacuo*, the residue was taken up in EtOAc and washed with 1 M $KHSO_4$ soln., H_2O, sat. $NaHCO_3$ soln. and brine. The organic phase was dried (Na_2SO_4) and evaporated *in vacuo*. The residue was purified by flash chromatography.

***GP 6a:** Global deprotection and cyclization of Alloc-protected heptapeptides*

C-deprotection: The protected heptapeptide (1.0 equiv) was dissolved in 1,4-dioxane (0.1 M) and 1 M aq. LiOH solution (typically 1.2 to 1.8 equiv) was added at rt. The mixture was stirred until full conversion was indicated by LC/MS or TLC. The mixture was then evaporated *in vacuo* and used without purification in the next step.

N-deprotection: The crude *C*-deprotected peptide was dissolved in MeCN/H_2O (1:1, 0.05 M), Et_2NH (5 equiv), triphenylphosphine-3,3',3''-trisulfonic acid trisodium salt (TPPTS) (typically 0.1 equiv) and $Pd(OAc)_2$ (typically 0.05 equiv) were added at rt. The mixture was stirred until LC/MS indicated full conversion and was subsequently evaporated in vacuo. The residue was azeotroped with DCM two times to remove residual H_2O.

Cyclization: The crude *N*- and *C*-deprotected peptide was dissolved in DMF (0.1 M) and the resulting solution was added dropwise to a solution of HATU (typically 3.5 equiv) and DIPEA (typically 5.0 equiv) in DCM (1 mM) over the course of 2–6 hours at 2rt. The mixture was stirred until LC/MS indicated full conversion (typically 16 to 24 h). The solvent was then removed *in vacuo* and the residue was redissolved in EtOAc, washed with 1 M $KHSO_4$, 1 M LiCl, sat. $NaHCO_3$ and sat. NaCl solutions, dried (Na_2SO_4) and evaporated.

TBDMS cleavage: The crude cyclic peptide was dissolved in MeOH (0.1 M), NH_4F (typically 10–20 equiv) was added and the mixture was heated to 45 °C until LC/MS indicated full conversion (typically 18 – 24 h). The reaction mixture was then diluted with EtOAc and washed with $NaHCO_3$ soln. (3x). The combined aqueous phases were back-extracted with EtOAc once. The combined organic phases were dried (Na_2SO_4) and evaporated *in vacuo*. The residue was purified by RP flash chromatography.

***GP6b**: Global deprotection and cyclization of Fmoc-protected heptapeptides*

N-deprotection: The protected heptapeptide (1.0 equiv) was dissolved in DCM (0.1 M) and tris(2-aminoethyl)amine (tris(2-aminoethyl)amine) (10 equiv) was added at rt. The mixture was stirred until full conversion was indicated by LCMS or TLC (typically 30 min) and was then diluted with EtOAc. It was then washed with H_2O (2x) and phosphate buffer (pH = 5.5, 66.7 mM) (2x). The aqueous phases were back-extracted with EtOAc (1x), the combined organic phases were dried (Na_2SO_4) and evaporated *in vacuo*.

C-deprotection: The crude deprotected heptapeptide was dissolved in 1,4-dioxane (0.1 M) and 1 M aq. LiOH solution (typically 2.2 equiv) was added at rt. The mixture was stirred at this temperature until full conversion was indicated by LC/MS and then evaporated *in vacuo*.

Cyclization and *TBDMS cleavage* were perfromed as described in *GP6a*

***GP6c:** Global deprotection and cyclization of 6-N-methylated heptapeptides*

Depending on the N-protecting group, *N-deprotection* and *C-deprotection* were perfomed as described in *GP6a* or *GP6b*.

Cyclization: The crude *N*- and *C*-deprotected peptide was dissolved in DCE (0.1 M) and the resulting solution was added dropwise to a solution of HATU (typically 3.5 equiv) and DIPEA (typically 5.0 equiv) in DCE (1 mM) over the course of 2 – 4 hours at 60 °C. The mixture was then stirred for 16 – 24 h or until LC/MS indicated full conversion. The solvent was then removed *in vacuo*, the residue was redissolved in EtOAc, washed with 1 M $KHSO_4$, 1 M LiCl, sat. $NaHCO_3$ and sat. NaCl solutions, dried (Na_2SO_4) and evaporated.

TBDMS cleavage was performed as described in *GP6a*.

***GP7**: Dimerization of cyclic peptides by CuAAC*

A vial was charged with the alkyne-bearing cyclic peptide (2.0 equiv). A solution of the diazide in *t*-BuOH (typically ca. 20 mg ml^{-1}; 1.0 equiv) was added to the peptide followed by additional *t*-BuOH and H_2O to reach a final concentration of 0.025 M and a ratio *t*-BuOH to H_2O of 1:1. 1 M $CuSO_4$ (aq.) (typically 0.8 equiv) and 1 M sodium ascorbate (aq.) (typically 1.3 equiv) were added, which resulted in a brown coloration of the solution that dissipated after a few seconds. The vial was immediately flushed with Argon and sealed. After full conversion was reached (indicated by LC/MS), the mixture was evaporated *in vacuo* and the residue was purified by RP flash chromatography (followed by prep HPLC in most cases).

Synthesis of the compounds

Synthesis of exit vector 6 Homo-BacPROTACs (6 – 9)

methyl *N*-(((2*S*,3*R*)-2-((*S*)-2-((2*S*,4*R*)-2-((*S*)-2-(((allyloxy)carbonyl)amino)-*N*-methyl-3-(1-(prop-2-yn-1-yl)-1*H*-indol-3-yl)propanamido)-5-((*tert*-butyldimethylsilyl)oxy)-4-methylpentanamido)propanamido)-3-methoxy-3-phenylpropanoyl)-L-valyl)-*N*-methyl-L-leucinate (3)

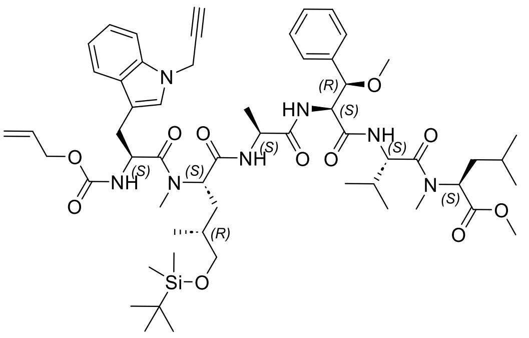

Prepared according to *GP1* and *GP3*: Pentapeptide **2**[4] (803 mg, 894 µmol), Pd/C (80.3 mg) (2 h); Alloc-Trp(propargyl)-OH[5] (344 mg, 1.05 mmol), BEP (289 mg, 1.05 mmol), NMM (0.246 ml, 2.24 mmol) (16 h). Flash chromatography (DCM/EtOAc 100:0 – 60:40) followed by lyophilization afforded **3** (82.0 mg, 76.5 µmol, 69%) as a white amorphous solid. R_f = 0.37 (DCM/EtOAc 1:1).

$[\alpha]_{20}^{D}$ = –58.2 (c 0.5, $CHCl_3$).

¹H NMR (500 MHz, $CDCl_3$) *(mixture of rotamers, ratio 3:2)* δ -0.27 – -0.19 (m, 0.3H), -0.05 (s, 0.8H), -0.03 (s, 0.8H), 0.01 – 0.03 (m, 2.5H), 0.03 (s, 2.2H), 0.40 (d, *J* = 6.6 Hz, 0.8H), 0.83 (s, 2.4H), 0.88 (s, 7H), 0.89 – 0.98 (m, 12.9H), 0.99 (d, *J* = 6.9 Hz, 2.4H), 1.19 (d, *J* = 7.0 Hz, 2.3H), 1.22 – 1.28 (m, 1.4H), 1.44 – 1.54 (m, 2.4H), 1.66 – 1.80 (m, 3.1H), 1.86 – 1.92 (m, 0.6H), 1.94 – 2.04 (m, 1.0H), 2.07 – 2.21 (m, 1.2H), 2.36 – 2.43 (m, 1.0H), 2.73 (s, 2.0H), 2.75 (s, 0.9H), 2.97 (s, 1.0H), 3.01 (s, 2.1H), 3.06 (dd, *J* = 9.6, 5.3 Hz, 0.4H), 3.09 – 3.17 (m, 1.1H), 3.21 (dd, *J* = 14.5, 8.1 Hz, 0.9H), 3.28 (s, 1.3H), 3.33 (s, 2.4H), 3.40 (dd, *J* = 9.8, 5.4 Hz, 0.7H), 3.45 (dd, *J* = 9.8, 4.7 Hz, 0.8H), 3.67 – 3.71 (m, 3.0H), 4.17 – 4.23 (m, 0.6H), 4.24 – 4.31 (m, 0.4H), 4.46 – 4.59 (m, 2.2H), 4.63 (dd, *J* = 7.6, 3.4 Hz, 0.6H), 4.70 (dd, *J* = 7.7, 3.6 Hz, 0.9H), 4.75 – 4.91 (m, 5.3H), 4.94 – 5.02 (m, 0.9H), 5.15 – 5.32 (m, 2.3H), 5.35 (dd, *J* = 10.6, 5.3 Hz, 1.0H), 5.59 (d, *J* = 7.6 Hz, 0.3H), 5.80 – 5.92 (m, 1.5H), 6.36 (d, *J* = 6.7 Hz, 0.6H), 6.75 (d, *J* = 7.5 Hz, 0.4H), 6.78 (d, *J* = 7.6 Hz, 0.6H), 7.04 (s, 1.0H), 7.11 – 7.25 (m, 5.5H), 7.27 – 7.32 (m, 2.0H), 7.33 – 7.38 (m, 1.5H), 7.43 (d, *J* = 8.7 Hz, 0.5H), 7.59 (d, *J* = 7.9 Hz, 0.3H), 7.67 (d, *J* = 7.9 Hz, 0.6H), 7.83 (d, *J* = 6.7 Hz, 0.3H).

¹³C-NMR (126 MHz, $CDCl_3$) *(mixture of rotamers)* δ -5.3, -5.25, -5.21, 15.7, 16.3, 17.4, 17.5, 17.55, 17.59, 17.9, 18.4, 18.5, 19.6, 19.7, 21.53, 21.54, 23.42, 23.44, 24.9, 25.0, 26.07, 26.10, 28.5, 29.0, 29.3, 31.28, 31.35, 31.4, 31.5, 31.7, 32.0, 32.2, 35.75, 35.81, 35.9, 37.1, 49.8, 49.9, 50.9, 51.6, 52.26, 52.28, 54.2, 54.4, 54.60, 54.7, 56.3, 57.6, 57.7, 57.9, 58.0, 65.9, 66.4, 67.4, 68.5, 73.8, 73.8, 77.7, 78.0, 81.3, 81.5, 109.57, 109.65, 109.8, 109.9, 117.8, 118.4, 118.97, 119.04, 120.1, 120.2, 122.4, 122.7, 126.3, 126.4, 127.02, 127.04, 128.18, 128.24, 128.3, 128.4, 128.51, 128.54, 132.2, 132.9, 136.0, 136.2, 137.0, 137.1, 156.0, 156.8, 168.6, 168.7, 168.9, 170.5, 171.68, 171.72, 172.1, 172.2, 172.3, 172.5, 173.4.

Selected diagnostic peaks:

Major rotamer: **¹H NMR** (500 MHz, $CDCl_3$) δ 0.02 (s, 3H), 0.03 (s, 3H), 0.88 (s, 9H), 2.39 (t, *J* = 2.5 Hz, 1H), 2.73 (s, 3H), 3.01 (s, 3H), 3.14 (dd, *J* = 13.5, 5.6 Hz, 1H), 3.33 (s, 3H), 3.40 (dd, *J* = 9.8, 5.4 Hz, 1H), 3.45 (dd, *J* = 9.8, 4.7 Hz, 1H), 3.69 (s, 3H), 4.20 (t, *J* = 6.9 Hz, 1H), 4.70 (dd, *J* = 7.7, 3.6 Hz, 1H), 5.35 (dd, *J* = 10.6, 5.3 Hz, 1H), 6.36 (d, *J* = 6.7 Hz, 1H), 6.78 (d, *J* = 7.6 Hz, 1H), 7.04 (s, 1H), 7.67 (d, *J* = 7.9 Hz, 1H). **¹³C-NMR** (126 MHz, $CDCl_3$) δ 26.10, 32.2, 52.28, 54.7, 57.7, 67.4, 73.78, 156.0.

Minor rotamer: **¹H NMR** (500 MHz, $CDCl_3$) δ -0.27 – -0.19 (m, 1H), -0.05 (s, 3H), -0.03 (s, 3H), 0.40 (d, *J* = 6.6 Hz, 3H), 0.83 (s, 9H), 2.39 (t, *J* = 2.6 Hz, 1H), 2.75 (s, 3H), 2.97 (s, 3H), 3.28 (s, 3H), 3.69 (s, 3H), 4.28 (t, *J* = 6.9 Hz, 1H), 5.59 (d, *J* = 7.6 Hz, 1H), 6.75 (d, *J* = 7.5 Hz, 1H), 7.59 (d, *J* = 7.9 Hz, 1H), 7.83 (d, *J* = 6.7 Hz, 1H). δ 26.07, 52.26, 57.6, 73.82. **¹³C-NMR** (126 MHz, $CDCl_3$) δ 26.07, 52.26, 54.60, 57.6, 68.5, 73.8, 156.8.

HRMS (ESI): calcd for $C_{57}H_{86}N_7O_{11}Si^+$ $(M+H)^+$: 1072.6149; found: 1072.6121.

methyl *N*-(((2*S*,3*R*)-2-((*S*)-2-((2*S*,4*R*)-2-((*S*)-2-((*S*)-2-(((allyloxy)carbonyl)amino)-3-methylbutanamido)-*N*-methyl-3-(1-(prop-2-yn-1-yl)-1*H*-indol-3-yl)propanamido)-5-((*tert*-butyldimethylsilyl)oxy)-4-methylpentanamido)propanamido)-3-methoxy-3-phenylpropanoyl)-L-valyl)-*N*-methyl-L-leucinate (4)

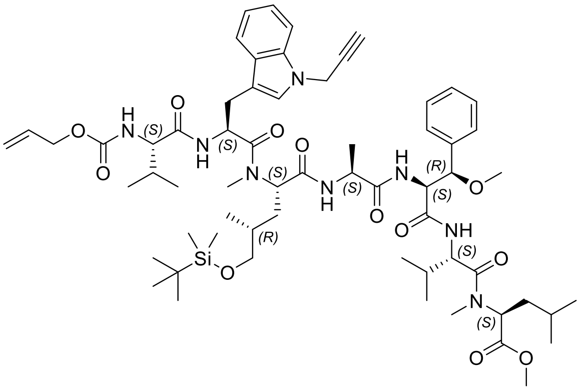

Prepared according to *GP2* and *GP4*: **3** (635 mg, 592 µmol), DMBA (277 mg, 1.78 mmol), $Pd(PPh_3)_4$ (20.5 mg, 17.8 µmol) (1 h); Alloc-L-Val-OH (131 mg, 651 µmol), HOBt (109 mg, 710 µmol), EDC (136 mg, 710 µmol), NMM (0.130 ml, 1.18 mmol) in DMF (6 ml) (18 h). Column chromatography (DCM/EtOAc 100:0 – 60:40) followed by lyohpilization afforded **4** (525 mg, 0.448 mmol, 76% yield) as a white amorphous solid. R_f = 0.30 (DCM/EtOAc 1:1).

$[\alpha]_{20}^{D}$ = –64.2 (c 0.5, $CHCl_3$)

^{1}H-NMR (500 MHz, $CDCl_3$) *(mixture of rotamers, ratio ~2:1)* δ -0.26 – -0.17 (m, 0.3H), -0.05 (s, 1.0H), -0.03 (s, 1.1H), 0.02 (s, 2.0H), 0.03 (s, 2.2H), 0.42 (d, *J* = 6.6 Hz, 0.8H), 0.83 (s, 4.4H), 0.88 (s, 6.0H), 0.88 – 0.91 (m, 19.1H), 0.99 (d, *J* = 6.8 Hz, 2.7H), 1.18 – 1.24 (m, 2.9H), 1.40 – 1.53 (m, 2.8H), 1.66 – 1.80 (m, 5.2H), 1.93 – 2.03 (m, 1.4H), 2.04 – 2.17 (m, 2.1H), 2.36 – 2.43 (m, 1.0H), 2.71 (s, 1.0H), 2.75 (m, 1.8H), 2.95 – 3.00 (m, 3.3H), 3.07 – 3.18 (m, 1.5H), 3.23 (dd, *J* = 14.5, 8.0 Hz, 0.9H), 3.29 (s, 1.1H), 3.33 (s, 2.4H), 3.40 – 3.44 (m, 1.4H), 3.69 (s, 3.2H), 3.98 – 4.08 (m, 0.9H), 4.22 – 4.30 (m, 0.7H), 4.31 – 4.38 (m, 0.4H), 4.51 – 4.68 (m, 2.3H), 4.66 – 4.73 (m, 1.5H), 4.75 – 4.86 (m, 4.6H), 4.89 – 5.02 (m, 1.2H), 5.16 – 5.41 (m, 5.0H), 5.65 (d, *J* = 9.5 Hz, 0.3H), 5.86 – 6.00 (m, 1.0H), 6.34 (d, *J* = 6.9 Hz, 0.6H), 6.74 – 6.83 (m, 1.7H), 7.04 (s, 1.0H), 7.11 – 7.18 (m, 2.0H), 7.19 – 7.24 (m, 4.0H), 7.27 – 7.40 (m, 4.7H), 7.56 – 7.58 (m, 0.4H), 7.67 – 7.69 (m, 0.5H), 8.10 (d, *J* = 7.0 Hz, 0.3H).

^{13}C-NMR (126 MHz, $CDCl_3$) *(mixture of rotamers)* δ -5.3, -5.25, -5.22, 15.9, 17.3, 17.4, 17.5, 17.7, 18.0, 18.4, 18.5, 19.2, 19.3, 19.6, 19.7, 21.5, 21.6, 23.4, 23.5, 24.95, 24.97, 26.07, 26.10, 28.2, 28.8, 29.3, 31.1, 31.3, 31.4, 31.50, 31.55, 31.8, 32.3, 35.76, 35.80, 37.1, 49.4, 49.9, 50.0, 52.3, 54.2, 54.6, 54.7, 56.0, 57.6, 57.66, 57.71, 58.1, 58.2, 60.1, 60.2, 66.0, 66.2, 67.1, 68.6, 73.8, 73.9, 77.7, 78.1, 81.2, 81.4, 109.5, 109.7, 109.75, 109.80, 117.9, 118.1, 118.96, 119.05, 120.1, 120.3, 122.4, 122.7, 126.3, 127.09, 127.14, 128.3, 128.36, 128.41, 128.5, 128.6, 128.7, 132.06, 132.08, 132.2, 132.3, 132.8, 136.1, 136.2, 136.9, 156.2, 156.4, 168.5, 168.7, 170.3, 170.9, 171.6, 171.8, 172.08, 172.11, 172.20, 172.24, 172.5, 172.8.

Selected diagnostic peaks:

Major rotamer: **^{1}H NMR** (500 MHz, $CDCl_3$) δ 0.02 (s, 3H), 0.03 (s, 3H), 0.88 (s, 9H), 2.39 (t, *J* = 2.3 Hz, 1H), 2.75 (s, 3H), 2.98 (s, 3H), 3.23 (dd, *J* = 14.5, 8.0 Hz, 1H), 3.33 (s, 3H), 3.69 (s, 3H), 3.99 – 4.08 (m, 1H), 4.22 – 4.31 (m, 1H), 5.87 – 5.97 (m, 1H), 6.34 (d, *J* = 6.9 Hz, 1H), 7.04 (s, 1H). **^{13}C-NMR** (126 MHz, $CDCl_3$) δ 26.10, 28.8, 31.5, 49.4, 117.9, 156.2.

Minor rotamer: **^{1}H NMR** (500 MHz, $CDCl_3$) δ -0.26 – -0.17 (m, 1H), -0.05 (s, 3H), -0.03 (s, 3H), 0.42 (d, *J* = 6.6 Hz, 3H), 0.83 (s, 9H), 3.29 (s, 3H), 4.32 – 4.37 (m, 1H), 5.65 (d, *J* = 9.1 Hz, 1H), 8.10 (d, *J* = 7.2 Hz, 1H). δ **^{13}C-NMR** (126 MHz, $CDCl_3$) 28.2, 49.9, 118.1, 156.4.

HRMS (ESI): calcd for $C_{62}H_{95}N_8O_{12}Si^+$ $(M+H)^+$: 1171.6833; found: 1171.6830.

(3*S*,6*S*,9*S*,12*S*,15*S*,18*S*,21*S*)-21-((*R*)-3-hydroxy-2-methylpropyl)-9-isobutyl-6,12-diisopropyl-15-((*R*)-methoxy(phenyl)methyl)-1,10,18-trimethyl-3-((1-(prop-2-yn-1-yl)-1*H*-indol-3-yl)methyl)-1,4,7,10,13,16,19-heptaazacyclohenicosane-2,5,8,11,14,17,20-heptaone (5)

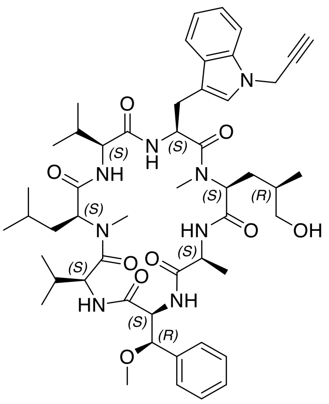

Prepared according to *GP6a*: **4** (509 mg, 434 μmol), 1 M LiOH (521 μl, 0.521 μmol) (3.5 h); $Pd(OAc)_2$ (2.9 mg, 13.0 μmol), TPPTS (14.8 mg, 26.0 μmol), Et_2NH (0.22 ml, 2.17 mmol) (1 h); HATU (576 mg, 1.52 mmol), DIPEA (0.303 ml, 1.74 mmol) (addition over 1.5 h, additional 16 h); NH_4F (322 mg, 8.68 mmol) (17 h). RP flash chromatography (H_2O/MeCN 90:10 - 5:95) followed by lyophilizaiton afforded **5** (247 mg, 262 μmol, 60%) as an off-white, amorphous solid.

$[\alpha]^{D}_{20}$ = -118.5 (c 0.5, $CHCl_3$).

^{1}H-NMR (500 MHz, $CDCl_3$): δ -0.62 – -0.52 (m, 1H), 0.23 (d, *J* = 6.8 Hz, 3H), 0.60 (d, *J* = 6.3 Hz, 3H), 0.64 (d, *J* = 6.6 Hz, 3H), 0.70 – 0.79 (m, 2H), 0.96 (d, *J* = 6.9 Hz, 3H), 0.97 – 1.01 (m, 6H), 1.08 (d, *J* = 6.6 Hz, 3H), 1.10 – 1.16 (m, 1H), 1.17 (d, *J* = 7.3 Hz, 3H), 1.52 – 1.63 (m, 1H), 1.80 – 1.90 (m, 1H), 2.19 – 2.29 (m, 1H), 2.33 (ddd, *J* = 13.4, 10.4, 4.5 Hz, 1H), 2.41 (t, *J* = 2.6 Hz, 1H), 2.51 (bs, 1H), 2.59 (s, 3H), 2.84 (s, 3H), 2.89 – 2.97 (m, 1H), 2.98 – 3.04 (m, 1H), 3.12 (dd, *J* = 13.4, 4.7 Hz, 1H), 3.32 (dd, *J* = 13.7, 10.9 Hz, 1H), 3.36 (s, 3H), 4.05 (t, *J* = 9.5 Hz, 1H), 4.32 (dd, *J* = 11.0, 2.7 Hz, 1H), 4.48 (t, *J* = 8.6 Hz, 1H), 4.73 – 4.78 (m, 2H), 4.80 (dd, *J* = 10.2, 2.5 Hz, 2H), 4.83 – 4.88 (m, 1H), 4.91 (t, *J* = 5.1 Hz, 1H), 5.07 (d, *J* = 5.5 Hz, 1H), 6.92 (d, *J* = 5.4 Hz, 1H), 7.00 (s, 1H), 7.09 – 7.15 (m, 1H), 7.16 – 7.22 (m, 3H), 7.22 – 7.26 (m, 4H), 7.35 (d, *J* = 8.4 Hz, 1H), 7.48 (d, *J* = 8.0 Hz, 1H), 8.12 (d, *J* = 8.0 Hz, 1H), 8.23 (d, *J* = 9.5 Hz, 1H), 8.48 (d, *J* = 10.4 Hz, 1H).

^{13}C-NMR (126 MHz, $CDCl_3$): δ 17.3, 18.7, 19.5, 20.1, 20.2, 21.0, 22.7, 23.7, 25.4, 28.4, 29.3, 29.7, 30.9, 31.7, 31.9, 33.2, 35.8, 39.1, 50.5, 51.2, 55.4, 56.1, 57.9, 59.1, 59.3, 66.0, 74.1, 77.6, 80.0, 108.8, 109.9, 118.9, 120.4, 122.9, 126.4, 128.0, 128.2, 128.3, 128.8, 135.1, 136.0, 168.8, 169.2, 170.1, 170.7, 171.3, 171.6, 172.2.

HRMS (ESI): calcd for $C_{51}H_{73}N_8O_9^+$ $(M+H)^+$: 941.5495; found: 941.5483.

(3*S*,3'*S*,6*S*,6'*S*,9*S*,9'*S*,12*S*,12'*S*,15*S*,15'*S*,18*S*,18'*S*,21*S*,21'*S*)-15,15'-(((((oxybis(ethane-2,1-diyl))bis(1*H*-1,2,3-triazole-1,4-diyl))bis(methylene))bis(1*H*-indole-1,3-diyl))bis(methylene))bis(12-((*R*)-3-hydroxy-2-methylpropyl)-21-isobutyl-3,18-diisopropyl-6-((*R*)-methoxy(phenyl)methyl)-1,9,13-trimethyl-1,4,7,10,13,16,19-heptaazacyclohenicosane-2,5,8,11,14,17,20-heptaone) (6)

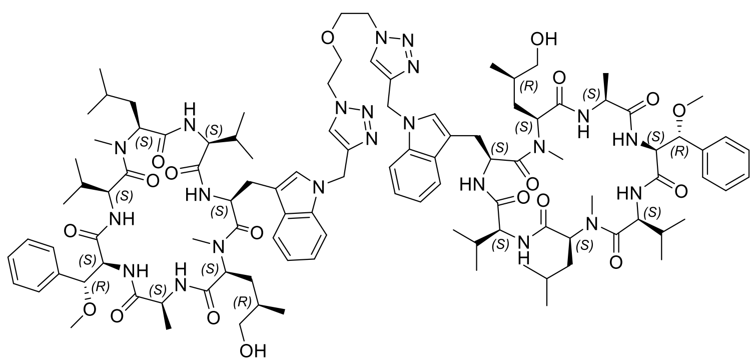

Prepared according to *GP7*: **5** (40.7 mg, 43.2 μmol), 1-azido-2-(2-azidoethoxy)ethane (3.39 mg, 21.6 μmol), 1 M $CuSO_4$ (17.3 μl, 17.3 μmol), 1 M sodium ascorbate (30.3 μl, 30.3 μmol) (5 h). RP flash chromatography (H_2O/MeCN 70:30 – 5:95) followed by lyophilization afforded **6** (34.1 mg, 16.7 μmol, 77%) as a white, amorphous solid.

$[\alpha]^{D}_{20} = -102.5$ (c 0.5, $CHCl_3$)

^{1}H-NMR (500 MHz, $CDCl_3$): δ -0.60 (t, *J* = 9.1 Hz, 2H), 0.20 (d, *J* = 6.8 Hz, 6H), 0.62 (d, *J* = 6.6 Hz, 6H), 0.65 (d, *J* = 6.8 Hz, 6H), 0.86 (q, *J* = 7.0 Hz, 2H), 0.96 – 1.04 (m, 18H), 1.02 – 1.09 (m, 2H), 1.13 (d, *J* = 6.6 Hz, 6H), 1.18 (d, *J* = 7.1 Hz, 6H), 1.60 (ddd, *J* = 13.4, 11.2, 5.4 Hz, 2H), 1.76 (ddd, *J* = 13.2, 11.0, 5.8 Hz, 2H), 2.23 – 2.36 (m, 4H), 2.55 (s, 6H), 2.86 (s, 10H), 3.06 (d, *J* = 4.3 Hz, 2H), 3.14 (dd, *J* = 13.6, 5.1 Hz, 2H), 3.29 – 3.38 (m, 2H), 3.40 (s, 6H), 3.71 – 3.79 (m, 4H), 4.15 (t, *J* = 9.5 Hz, 2H), 4.35 – 4.45 (m, 6H), 4.50 (t, *J* = 8.4 Hz, 2H), 4.70 – 4.81 (m, 4H), 4.89 (dd, *J* = 10.1, 3.9 Hz, 2H), 4.93 (t, *J* = 5.1 Hz, 2H), 5.11 (d, *J* = 5.5 Hz, 2H), 5.28 (d, *J* = 15.6 Hz, 2H), 5.38 (d, *J* = 15.6 Hz, 2H), 7.01 (s, 2H), 7.11 (t, *J* = 7.3 Hz, 2H), 7.17 (d, *J* = 4.9 Hz, 2H), 7.19 – 7.29 (m, 14H), 7.36 (s, 2H), 7.43 (d, *J* = 8.2 Hz, 4H), 7.50 (d, *J* = 8.0 Hz, 2H), 8.11 (d, *J* = 7.9 Hz, 2H), 8.25 (d, *J* = 9.5 Hz, 2H), 8.39 (d, *J* = 10.4 Hz, 2H).

^{13}C-NMR (126 MHz, $CDCl_3$): δ 16.4, 18.7, 19.4, 20.06, 20.14, 21.0, 22.7, 23.7, 25.3, 28.1, 29.2, 29.7, 30.9, 31.6, 31.9, 32.4, 39.0, 41.3, 50.0, 50.4, 51.1, 55.3, 56.0, 57.9, 58.7, 58.8, 59.0, 66.6, 69.1, 80.0, 108.8, 110.0, 118.8, 120.0, 122.6, 123.4, 126.8, 127.8, 128.18, 128.23, 128.8, 135.1, 136.0, 143.7, 168.6, 168.7, 170.0, 170.7, 171.56, 171.60, 172.2.

HRMS (ESI): calcd for $C_{106}H_{153}N_{22}O_{19}^{+}$ $(M+H)^{+}$: 2038.1677; found: 2038.1714.

(3*S*,3'*S*,6*S*,6'*S*,9*S*,9'*S*,12*S*,12'*S*,15*S*,15'*S*,18*S*,18'*S*,21*S*,21'*S*)-15,15'-(((((((oxybis(ethane-2,1-diyl))bis(oxy))bis(ethane-2,1-diyl))bis(1*H*-1,2,3-triazole-1,4-diyl))bis(methylene))bis(1*H*-indole-1,3-diyl))bis(methylene))bis(12-((*R*)-3-hydroxy-2-methylpropyl)-21-isobutyl-3,18-diisopropyl-6-((*R*)-methoxy(phenyl)methyl)-1,9,13-trimethyl-1,4,7,10,13,16,19-heptaazacyclohenicosane-2,5,8,11,14,17,20-heptaone) (7)

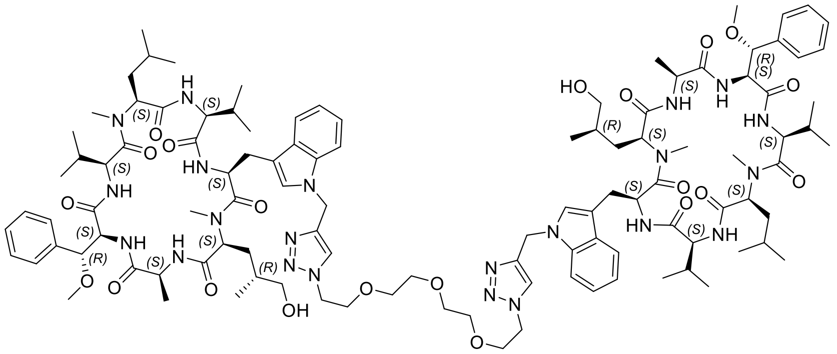

Prepared according to *GP7*: **5** (17.0 mg, 18.1 µmol), 1-azido-2-(2-(2-(2-azidoethoxy)ethoxy)ethoxy)ethane (2.21 mg, 9.03 µmol), 1 M $CuSO_4$ (9.03 µl, 9.03 µmol), 1 M sodium ascorbate (9.03 µl, 9.03 µmol) (4.5 h). RP flash chromatography (H_2O/MeCN 90:10 – 95:5) followed by prep HPLC (H_2O/MeCN 90:10 – 95:5) and lyophilization afforded **7** (14.0 mg, 6.48 µmol, 71%) as a white, amorphous solid.

$[\alpha]_{20}^{D}$ = −114.8 (c 0.5, $CHCl_3$).

^{1}H-NMR (500 MHz, $CDCl_3$): δ -0.80 (dd, *J* = 10.8, 7.5 Hz, 2H), 0.10 (d, *J* = 6.8 Hz, 6H), 0.57 (d, *J* = 6.4 Hz, 6H), 0.61 (d, *J* = 6.6 Hz, 6H), 0.72 (d, *J* = 9.3 Hz, 2H), 0.84 (q, *J* = 6.1 Hz, 2H), 0.93 – 1.00 (m, 18H), 1.08 (d, *J* = 6.6 Hz, 6H), 1.10 – 1.19 (m, 8H), 1.52 – 1.61 (m, 2H), 1.62 – 1.72 (m, 2H), 2.18 – 2.27 (m, 2H), 2.29 (ddd, *J* = 14.7, 10.0, 4.7 Hz, 2H), 2.52 (s, 6H), 2.75 – 2.83 (m, 2H), 2.82 (s, 6H), 2.93 – 3.02 (m, 4H), 3.06 (dd, *J* = 13.5, 4.7 Hz, 2H), 3.21 – 3.30 (m, 2H), 3.30 – 3.36 (m, 4H), 3.35 (s, 6H), 3.40 – 3.46 (m, 4H), 3.75 (t, *J* = 5.2 Hz, 4H), 4.13 (t, *J* = 9.5 Hz, 2H), 4.32 (dd, *J* = 11.2, 2.8 Hz, 2H), 4.42 (td, *J* = 5.0, 2.8 Hz, 4H), 4.48 (t, *J* = 8.5 Hz, 2H), 4.67 – 4.78 (m, 4H), 4.85 (dd, *J* = 10.1, 3.9 Hz, 2H), 4.89 (t, *J* = 5.1 Hz, 2H), 5.07 (d, *J* = 5.5 Hz, 2H), 5.24 (d, *J* = 15.7 Hz, 2H), 5.34 (d, *J* = 15.6 Hz, 2H), 6.98 (s, 2H), 7.08 (t, *J* = 7.4 Hz, 2H), 7.15 – 7.19 (m, 8H), 7.21 – 7.24 (m, 6H), 7.40 (d, *J* = 8.2 Hz, 2H), 7.45 (d, *J* = 7.9 Hz, 2H), 7.52 (s, 2H), 7.63 (d, *J* = 4.9 Hz, 2H), 8.11 (d, *J* = 7.9 Hz, 2H), 8.28 (d, *J* = 9.5 Hz, 2H), 8.45 (d, *J* = 10.3 Hz, 2H).

^{13}C-NMR (126 MHz, $CDCl_3$): δ 16.7, 18.7, 19.4, 20.1, 20.2, 21.0, 22.8, 23.7, 25.3, 28.2, 29.3, 29.7, 30.9, 31.6, 32.0, 32.6, 39.0, 41.4, 50.37, 50.45, 51.1, 55.3, 56.0, 57.9, 58.8, 59.0, 59.1, 66.4, 69.3, 70.3, 70.5, 80.0, 108.7, 110.1, 118.7, 120.0, 122.6, 123.4, 126.8, 127.9, 128.2, 128.3, 128.8, 135.1, 136.1, 143.7, 168.7, 168.9, 170.0, 170.7, 171.57, 171.63, 172.3.

HRMS (ESI): calcd for $C_{110}H_{161}N_{22}O_{21}^{+}$ $(M+H)^{+}$: 2126.2201; found: 2126.2183.

(3*S*,3'*S*,6*S*,6'*S*,9*S*,9'*S*,12*S*,12'*S*,15*S*,15'*S*,18*S*,18'*S*,21*S*,21'*S*)-15,15'-(((((3,6,9,12-tetraoxatetradecane-1,14-diyl)bis(1*H*-1,2,3-triazole-1,4-diyl))bis(methylene))bis(1*H*-indole-1,3-diyl))bis(methylene))bis(12-((*R*)-3-

hydroxy-2-methylpropyl)-21-isobutyl-3,18-diisopropyl-6-((*R*)-methoxy(phenyl)methyl)-1,9,13-trimethyl-1,4,7,10,13,16,19-heptaazacyclohenicosane-2,5,8,11,14,17,20-heptaone) (8) (UdSBI-0545)

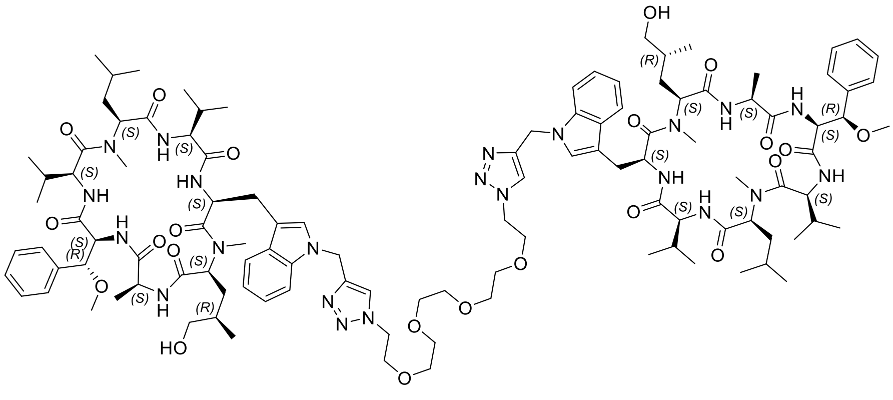

Prepared according to *GP7*: **5** (17.0 mg, 18.1 µmol), 1,14-diazido-3,6,9,12-tetraoxatetradecane (2.60 mg, 9.03 µmol), 1 M $CuSO_4$ (9.03 µl, 9.03 µmol), 1 M sodium ascorbate (9.03 µl, 9.03 µmol) (18 h). RP flash chromatography (H_2O/MeCN 90:10 – 95:5) followed by prep HPLC (H_2O/MeCN 90:10 – 95:5) and lyophilization afforded **8** (12.3 mg, 5.67 µmol, 63%) as a white, amorphous solid.

$[\alpha]_{20}^{D}$ = –98.3 (c 0.5, $CHCl_3$).

^{1}H-NMR (500 MHz, $CDCl_3$): δ -0.81 – -0.74 (m, 2H), 0.09 (d, *J* = 6.8 Hz, 6H), 0.56 (d, *J* = 6.5 Hz, 6H), 0.62 (d, *J* = 6.6 Hz, 6H), 0.67 – 0.78 (m, 2H), 0.93 – 1.01 (m, 20H), 1.09 (d, *J* = 6.8 Hz, 6H), 1.12 – 1.19 (m, 8H), 1.53 – 1.62 (m, 2H), 1.70 (s, 2H), 2.20 – 2.28 (m, 2H), 2.30 (ddd, *J* = 14.2, 10.1, 4.8 Hz, 2H), 2.57 (s, 6H), 2.83 (s, 10H), 3.00 (d, *J* = 10.6 Hz, 2H), 3.06 (dd, *J* = 13.3, 4.7 Hz, 2H), 3.14 (d, *J* = 6.9 Hz, 2H), 3.21 – 3.30 (m, 4H), 3.28 – 3.35 (m, 2H), 3.36 (s, 6H), 3.41 – 3.49 (m, 4H), 3.73 – 3.79 (m, 4H), 4.14 (t, *J* = 9.3 Hz, 2H), 4.31 (dd, *J* = 11.1, 2.9 Hz, 2H), 4.35 – 4.44 (m, 2H), 4.43 – 4.54 (m, 4H), 4.77 (dd, *J* = 10.6, 7.2 Hz, 4H), 4.85 (dd, *J* = 10.1, 3.9 Hz, 2H), 4.91 (t, *J* = 5.1 Hz, 2H), 5.08 (d, *J* = 5.5 Hz, 2H), 5.27 (d, *J* = 15.6 Hz, 2H), 5.45 (d, *J* = 15.6 Hz, 2H), 7.07 (s, 2H), 7.10 (d, *J* = 7.6 Hz, 2H), 7.15 – 7.22 (m, 6H), 7.20 – 7.26 (m, 6H), 7.43 (d, *J* = 8.4 Hz, 2H), 7.46 (d, *J* = 7.6 Hz, 2H), 7.58 (s, 2H), 7.79 (bs, 2H), 8.14 (d, *J* = 7.7 Hz, 2H), 8.31 (d, *J* = 9.5 Hz, 2H), 8.45 (d, *J* = 10.1 Hz, 2H).

^{13}C-NMR (126 MHz, $CDCl_3$): δ 16.9, 18.9, 19.4, 20.1, 20.2, 21.1, 22.8, 23.7, 25.2, 28.2, 29.3, 29.7, 30.9, 31.6, 32.1, 32.7, 39.1, 41.4, 50.4, 50.5, 51.2, 55.3, 56.1, 57.9, 58.8, 59.0, 59.2, 66.1, 69.4, 70.3, 70.4, 70.7, 80.0, 108.7, 110.2, 118.7, 120.1, 122.6, 123.5, 127.0, 128.0, 128.2, 128.3, 128.8, 135.1, 136.1, 143.7, 168.6, 169.0, 170.0, 170.7, 171.5, 171.6, 172.4.

HRMS (ESI): calcd for $C_{112}H_{165}N_{22}O_{22}^+$ (M+H)$^+$: 2170.2463; found: 2170.2490.

(3*S*,3'*S*,6*S*,6'*S*,9*S*,9'*S*,12*S*,12'*S*,15*S*,15'*S*,18*S*,18'*S*,21*S*,21'*S*)-15,15'-((((pentane-1,5-diylbis(1*H*-1,2,3-triazole-1,4-diyl))bis(methylene))bis(1H-indole-1,3-diyl))bis(methylene))bis(12-((*R*)-3-hydroxy-2-methylpropyl)-21-isobutyl-3,18-diisopropyl-6-((*R*)-methoxy(phenyl)methyl)-1,9,13-trimethyl-1,4,7,10,13,16,19-heptaazacyclohenicosane-2,5,8,11,14,17,20-heptaone) (9)

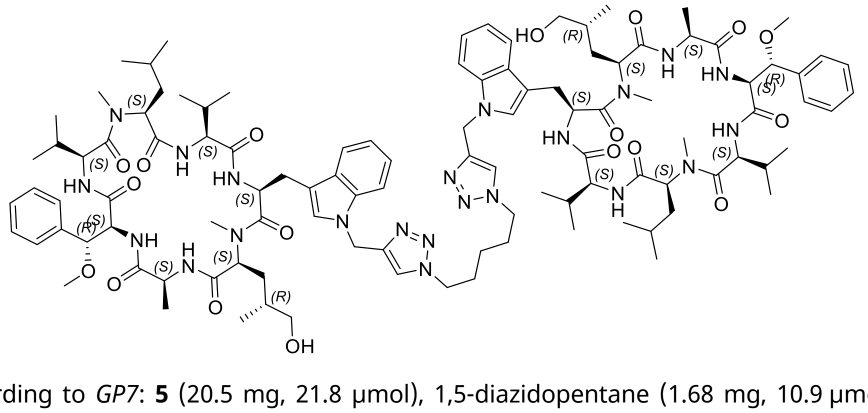

Prepared according to *GP7*: **5** (20.5 mg, 21.8 µmol), 1,5-diazidopentane (1.68 mg, 10.9 µmol), 1 M $CuSO_4$ (8.71 µl, 8.71 µmol), 1 M sodium ascorbate (14.2 µl, 14.2 µmol) (17 h). RP flash chromatography (H_2O/MeCN 70:30 – 5:95) followed by lyophilization afforded **9** (18.7 mg, 9.18 µmol, 84%) as a white amorphous solid.

$[\alpha]_{20}^{D}$ = −107.3 (c 0.5, $CHCl_3$)

^{1}H NMR (500 MHz, $CDCl_3$): δ -0.88 – -0.73 (m, 2H), 0.08 (d, *J* = 6.9 Hz, 6H), 0.57 (d, *J* = 6.4 Hz, 6H), 0.60 (d, *J* = 6.7 Hz, 6H), 0.69 – 0.76 (m, 2H), 0.85 – 0.93 (m, 2H), 0.93 – 1.00 (m, 18H), 1.08 (d, *J* = 6.6 Hz, 6H), 1.12 (d, *J* = 7.2 Hz, 6H), 1.15 (d, *J* = 4.6 Hz, 2H), 1.20 – 1.28 (m, 2H), 1.56 (q, *J* = 6.6 Hz, 2H), 1.61 – 1.70 (m, 2H), 1.80 – 1.90 (m, 6H), 2.19 – 2.26 (m, 2H), 2.26 – 2.33 (m, 2H), 2.48 (s, 6H), 2.71 – 2.78 (m, 2H), 2.82 (s, 6H), 3.00 (dd, *J* = 11.4, 4.5 Hz, 2H), 3.10 (dd, *J* = 13.5, 4.8 Hz, 2H), 3.24 – 3.32 (m, 2H), 3.35 (s, 6H), 4.12 (t, *J* = 9.5 Hz, 2H), 4.20 – 4.29 (m, 4H), 4.30 – 4.35 (m, 2H), 4.47 (t, *J* = 8.5 Hz, 2H), 4.67 – 4.76 (m, 4H), 4.84 (fdd, *J* = 10.1, 4.0 Hz, 2H), 4.89 (t, *J* = 5.1 Hz, 2H), 5.06 (d, *J* = 5.5 Hz, 2H), 5.24 (d, *J* = 15.6 Hz, 2H), 5.37 (d, *J* = 15.6 Hz, 2H), 6.92 (s, 2H), 7.09 (t, *J* = 7.5 Hz, 2H), 7.14 – 7.25 (m, 14H), 7.38 – 7.49 (m, 8H), 8.11 (d, *J* = 7.9 Hz, 2H), 8.27 (d, *J* = 9.6 Hz, 2H), 8.42 (d, *J* = 10.4 Hz, 2H).

^{13}C NMR (126 MHz, $CDCl_3$): δ 16.5, 18.7, 19.4, 20.1, 20.2, 20.9, 22.7, 23.4, 23.7, 25.3, 28.1, 29.2, 29.4, 29.7, 30.9, 31.7, 31.9, 32.5, 38.7, 39.0, 41.3, 50.0, 50.4, 51.1, 55.3, 56.0, 57.9, 58.8, 59.0, 59.1, 66.5, 80.1, 108.7, 110.0, 118.7, 120.1, 122.4, 122.7, 126.7, 127.8, 128.2, 128.3, 128.8, 135.1, 136.2, 143.8, 168.7, 168.8, 170.0, 170.8, 171.5, 171.6, 172.3.

HRMS (ESI): calcd for $C_{107}H_{155}N_{22}O_{18}^+$ (M+H)$^+$: 2036.1884; found: 2036.1908.

Synthesis of enantiomeric exit vector 6 Homo-BacPROTAC (8a)

***tert*-butyl *N*-[(1*S*,2*S*)-1,3-dihydroxy-1-(4-nitrophenyl)propan-2-yl]carbamate (SI-1)**

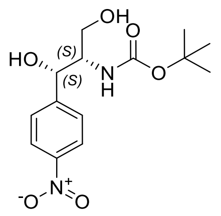

To a stirred solution of (1*S*,2*S*)-2-Amino-1-(4-nitro-phenyl)-propane-1,3-diol (45.0 g, 212 mmol) in MeOH (500 ml, 0.42 M) was added Boc-anhydride (50.9 g, 233 mmol) slowly at 0°C. The mixture was allowed to reach rt. After 12 h, TLC indicated complete conversion. The reaction was quenched with water, extracted with EtOAc, dried (Na_2SO_4) and concentrated in vacuo. The residue was triturated with petroleum ether and diethyl ether to yield crude **SI-1** (60.0 g, 192 mmol, 91%) which was used in the next step without further purification.

^{1}H NMR (400 MHz, DMSO-d_6) δ 1.05 (br s, 1.35H, rotamer), 1.21 (s, 7.65H, rotamer), 3.32 – 3.26 (m, 1H), 3.53 (dt, *J* = 10.5, 6.7 Hz, 1H), 3.73 – 3.61 (m, 1H), 4.76 (t, *J* = 5.2 Hz, 1H), 4.94 (br s, 1H), 5.58 (br s, 1H), 5.76 (d, *J* = 9.4 Hz, 0.15H, rotamer), 6.15 (d, *J* = 9.4 Hz, 0.85H, rotamer), 7.56 (d, J = 8.6 Hz, 2H), 8.17 (d, *J* = 8.6 Hz, 2H).

^{13}C NMR (101 MHz, DMSO-d_6) δ 27.6, 28.0, 57.5, 58.8, 60.7, 61.0, 69.9, 77.6, 122.8, 127.4, 146.3, 152.0, 152.4, 155.1.

MS calcd for $C_{10}H_{13}N_2O_6^+$ (M − C_4H_9 + 2H)$^+$: 257.08; found: 257.09.

The spectroscopic data are in agreement with previously published results.[10]

***tert*-butyl *N*-[(1*S*,2*S*)-3-[(*tert*-butyldimethylsilyl)oxy]-1-hydroxy-1-(4-nitrophenyl)propan-2-yl]carbamate (SI-2)**

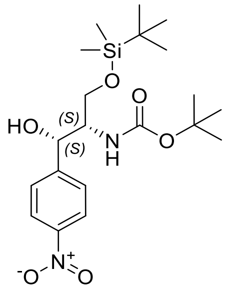

To a stirred solution of crude **SI-1** (60.0 g, 192 mmol) in DMF (300 ml, 0.64 M) were added imidazole (32.7 g, 480 mmol) and TBDMS-Cl (63.4 g, 423 mmol) at 0°C. The mixture was stirred at rt. After 6 h, TLC indicated

complete conversion. The mixture was quenched with cold water and extracted with EtOAc (2x). The combined organic layers were washed with cold brine, dried (Na_2SO_4) and concentrated in vacuo. Silica gel column chromatography (10% EtOAc in petroleum ether) yielded **SI-2** (65.0 g, 152 mmol, 79%).

¹H NMR (400 MHz, DMSO-d$_6$) δ 0.05 (s, 6H), 0.87 (s, 9H), 1.05 (br s, 1.8H, rotamer), 1.22 (s, 7.2H, rotamer), 3.57 – 3.40 (m, 1H), 3.81 – 3.59 (m, 2H), 4.90 (dd, *J* = 5.2, 2.2 Hz, 1H), 5.63 (d, *J* = 5.3 Hz, 1H), 5.88 (br d, *J* = 9.4 Hz, 0.2H, rotamer), 6.23 (br d, *J* = 8.9 Hz, 0.8H, rotamer), 7.56 (br d, *J* = 8.6 Hz, 2H), 8.18 (d, *J* = 8.6 Hz, 2H).

¹³C NMR (101 MHz, DMSO-d$_6$) δ -5.4, 17.9, 25.8, 27.6, 28.0, 57.5, 58.9, 62.3, 62.9, 69.9, 77.6, 122.8, 127.4, 146.4, 151.7, 155.1.

MS calcd for $C_{20}H_{35}N_2O_6Si^+$ $(M+H)^+$: 427.23; found 427.24.

The spectroscopic data are in agreement with previously published results.[11]

tert-butyl _N_-[(1_S_,2_S_)-3-[(_tert_-butyldimethylsilyl)oxy]-1-methoxy-1-(4-nitrophenyl)propan-2-yl]carbamate (SI-3)

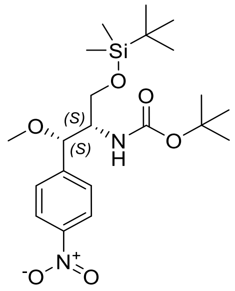

To a stirred solution of **SI-2** (55.0 g, 129 mmol) in DMF (300 ml, 0.43 M) was added LiHMDS (1 M in THF) (129 ml, 129 mmol) at -15°C. After 10 minutes, iodomethane (27.5 g, 193 mmol) was added. The mixture was stirred at –15°C. After 3 h, TLC indicated complete conversion. The reaction was quenched with cold water and extracted with EtOAc (2x). The combined organic layers were washed with brine, dried (Na_2SO_4) and concentrated in vacuo. Silica gel column chromatography (10% EtOAc in petroleum ether) yielded **SI-3** (52.0 g, 118 mmol, 91%).

¹H NMR (400 MHz, DMSO-d$_6$) δ 0.03 (s, 6H), 0.87 (s, 9H), 1.03 (br s, 1.8H, rotamer), 1.23 (s, 7.2H, rotamer), 3.17 (s, 3H), 3.54 – 3.36 (m, 1H), 3.77 – 3.56 (m, 2H), 4.51 (d, *J* = 3.3 Hz, 1H), 6.24 (br d, *J* = 7.9 Hz, 0.2H, rotamer), 6.60 (br d, *J* = 8.4 Hz, 0.8H, rotamer), 7.53 (d, J = 8.4 Hz, 2H), 8.21 (d, *J* = 8.6 Hz, 2H).

¹³C NMR (101 MHz, DMSO-d$_6$) δ -5.5, -5.4, 17.9, 25.7, 27.6, 28.0, 57.0, 61.9, 77.6, 80.5, 123.2, 128.3, 146.9, 147.6, 155.1.

MS calcd for $C_{17}H_{29}N_2O_6Si^+$ $(M – C_4H_9 + 2H)^+$: 385.18; found: 385.36.

The spectroscopic data are in agreement with previously published results.[12]

tert-butyl _N_-[(1_S_,2_S_)-1-(4-aminophenyl)-3-[(_tert_-butyldimethylsilyl)oxy]-1-methoxypropan-2-yl]carbamate (SI-4)

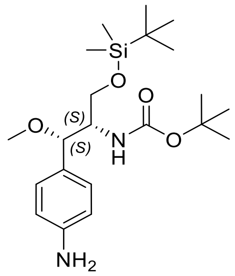

To a solution of **SI-3** (30.0 g, 68.1 mmol) in THF (300 ml, 0.23 M) was added 10 wt% Pd/C (36.4 g, 34.0 mmol). The mixture was stirred at rt under H_2 (50 psi). After 5 h, TLC indicated complete conversion. Subsequently, the mixture was filtered over Celite, rinsed with EtOAc and the filtrate was evaporated in vacuo. Silica gel column chromatography (20% EtOAc in petroleum ether) yielded **SI-4** (22.0 g, 53.6 mmol, 79%).

^{1}H NMR (400 MHz, $CDCl_3$) δ 0.03 (s, 3H), 0.05 (s, 3H), 0.91 (s, 9H), 1.38 (s, 9H), 3.20 (s, 3H), 3.42 (dd, *J* = 9.4, 3.8 Hz, 1H), 3.55 – 3.73 (m, 3H), 4.30 (d, *J* = 4.6 Hz, 1H), 4.89 (d, *J* = 8.8 Hz, 1H), 6.65 (d, *J* = 8.4 Hz, 2H), 7.07 (d, *J* = 8.4 Hz, 2H).

MS calcd for $C_{16}H_{27}N_2O_3Si^+$ (M – C_4H_9 – OCH_3 + H)$^+$: 323.18; found: 323.23.

The spectroscopic data are in agreement with previously published results.[12]

***tert*-butyl *N*-[(1*S*,2*S*)-3-[(*tert*-butyldimethylsilyl)oxy]-1-methoxy-1-phenylpropan-2-yl]carbamate (SI-5)**

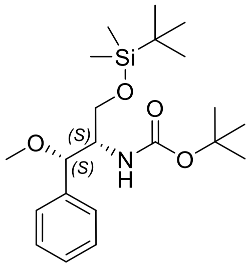

To a stirred solution of **SI-4** (20.0 g, 48.7 mmol) in $CHCl_3$ (200 ml) and water (200 ml) (0.12 M) was added acetic acid (58.4 g, 974 mmol) and sodium nitrite (16.8 g, 244 mmol). The mixture was stirred at rt. After 16 h, TLC indicated complete conversion. The reaction was diluted with water and extracted with DCM (2x). The combined organic layers were dried (Na_2SO_4) and concentrated in vacuo. Silica gel column chromatography (7% EtOAc in petroleum ether) yielded **SI-5** (10.4 g, 26.3 mmol, 54%).

^{1}H NMR (400 MHz, $CDCl_3$) δ 0.06 (s, 3H), 0.07 (s, 3H), 0.92 (s, 9H), 1.35 (s, 9H), 3.25 (s, 3H), 3.47 (dd, *J* = 9.7, 4.2 Hz, 1H), 3.65 (dd, *J* = 9.7, 7.4 Hz, 1H), 3.71 – 3.79 (m, 1H), 4.46 (d, *J* = 4.2 Hz, 1H), 4.88 (d, *J* = 9.1 Hz, 1H), 7.24 – 7.38 (m, 5H).

MS calcd for $C_{21}H_{38}NO_4Si^+$ (M+H)$^+$: 396.26; found: 396.34.

The spectroscopic data are in agreement with previously published results.[4]

***tert*-butyl *N*-[(1S,2S)-3-hydroxy-1-methoxy-1-phenylpropan-2-yl]carbamate (SI-6)**

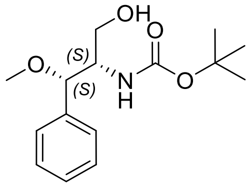

To a stirred solution of **SI-5** (16.0 g, 40.4 mmol) in THF (160 ml, 0.25 M) was added TBAF (1M in THF) (44.5 ml, 44.5 mmol) at 0°C. The mixture was stirred at rt. After 3 h, TLC indicated complete conversion. The reaction was quenched with cold water and extracted with EtOAc (2x). The combined organic layers were washed with brine, dried (Na_2SO_4) and concentrated in vacuo. Silica gel column chromatography (7% EtOAc in petroleum ether) yielded **SI-6** (11.0 g, 39.1 mmol, 97%).

^{1}H NMR (400 MHz, $CDCl_3$) δ 1.34 (s, 9H), 2.66 – 2.83 (m, 1H), 3.26 (s, 3H), 3.62 – 3.73 (m, 2H), 3.77 (dt, *J* = 9.0, 4.7 Hz, 1H), 4.42 (d, *J* = 4.2 Hz, 1H), 5.10 (bs, 1H), 7.25 – 7.40 (m, 5H).

MS calcd for $C_{15}H_{24}NO_4^+$ (M+H)$^+$: 282.17; found: 282.15.

The spectroscopic data are in agreement with previously published results.[12]

(2*R*,3*S*)-2-{[(*tert*-butoxy)carbonyl]amino}-3-methoxy-3-phenylpropanoic acid (SI-7)

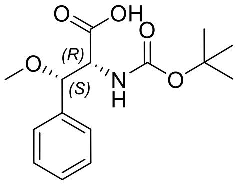

A stirred solution of **SI-6** (11.0 g, 39.1 mmol) in MeCN (88.0 ml) and water (88.0 ml) (0.22 M) was cooled to 0°C and NaH_2PO_4 (1.22 g, 7.82 mmol), (diacetoxyiodo)benzene (1.26 g, 3.91 mmol), TEMPO (1.22 g, 7.82 mmol) and $NaClO_2$ (12.3 g, 137 mmol) were added. The mixture was stirred at rt. After 2 h, TLC indicated complete conversion. The reaction was quenched with 2 M aq. Na_2CO_3 and washed with diethyl ether. The aqueous layer was acidified with 1 N aq. HCl and extracted with EtOAc (3x). The combined EtOAc layers were dried (Na_2SO_4) and concentrated in vacuo to yield crude **SI-7** (9.70 g, 31.4 mmol, 80%) which was used in the next step without further purification.

Major rotamer: ^{1}**H NMR** (500 MHz, $CDCl_3$) δ 1.33 (s, 9H), 3.32 (s, 3H), 4.56 (dd, *J* = 9.4, 2.9 Hz, 1H), 4.88 (d, *J* = 2.8 Hz, 1H), 5.33 (d, *J* = 9.4 Hz, 1H), 7.29 – 7.42 (m, 5H), 9.67 (bs, 1H).

^{13}**C NMR** (126 MHz, CDCl3) δ 28.3, 57.7, 59.1, 80.2, 82.4, 127.0, 128.4, 128.6, 136.9, 155.7, 175.4.

Minor rotamer (selected signals, ratio ~5:1): ^{1}**H NMR** (500 MHz, $CDCl_3$) δ 1.15 (s, 9H), 3.29 (s, 3H), 4.40 (dd, *J* = 9.3, 2.9 Hz, 1H), 4.78 – 4.83 (m, 1Hf), 5.88 (d, *J* = 9.3 Hz, 1H).

^{13}**C NMR** (126 MHz, CDCl3) δ 27.9, 57.6, 60.6, 81.0, 82.8, 127.2, 155.5.

MS calcd for $C_{11}H_{13}NO_5^+$ (M – C_4H_9 + 2H)$^+$: 239.08; found: 239.87.

The spectroscopic data are in agreement with previously published results.[4]

methyl *N*-(((benzyloxy)carbonyl)-D-valyl)-*N*-methyl-D-leucinate (SI-8)

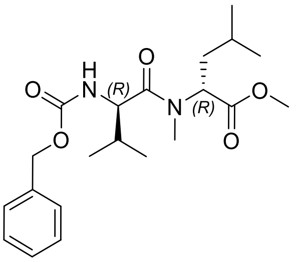

To a solution of methyl N-(*tert*-butoxycarbonyl)-N-methyl-D-leucinate (50.0 g, 193 mmol) in DCM (250 ml, 0.77 M) was added HCl (4 M in dioxane) (240.0 ml) dropwise at 0°C, the mixture was allowed to reach rt and stirred for 3 h. The solvents were evaporated in vacuo and the crude amine was used in the peptide coupling without further purification.

To a solution of Cbz-D-Valine (20.3 g, 80.7 mmol) in DMF (180 ml, 0.45 M) was added DIPEA (56.4 ml, 323 mmol) dropwise at 0 °C followed by portionwise addition of HATU (46.1 g, 121 mmol). After 10 minutes, the deprotected amino acid prepared as described above (15.8 g, 80.7 mmol) was added. The mixture was allowed to reach rt and stirred for 16 . The reaction was quenched with water and extracted with EtOAc. The organic layer was washed with brine, dried (Na_2SO_4) and concentrated in vacuo. Silica gel column chromatography (50% EtOAc in petroleum ether) yielded **SI-8** (25.5 g, 65.0 mmol, 81%).

Major rotamer: ^{1}**H NMR** (500 MHz, $CDCl_3$) δ 0.88 (d, *J* = 6.5 Hz, 3H), 0.92 (dd, *J* = 6.7, 2.4 Hz, 6H), 1.02 (d, *J* = 6.8 Hz, 3H), 1.40 – 1.52 (m, 1H), 1.64 – 1.79 (m, 2H), 2.01 – 2.10 (m, 1H), 3.00 (s, 3H), 3.68 (s, 3H), 4.54 (dd, *J* = 9.2, 6.0 Hz, 1H), 5.09 (s, 2H), 5.33 (dd, *J* = 10.6, 5.2 Hz, 1H), 5.52 (d, *J* = 9.2 Hz, 1H), 7.28 – 7.39 (m, 5H).

^{13}**C NMR** (126 MHz, $CDCl_3$) δ 172.8, 172.0, 156.4, 136.3, 128.5, 128.0, 127.9, 77.3, 77.0, 76.7, 66.8, 55.8, 54.4, 52.1, 36.8, 31.2, 31.1, 24.7, 23.2, 21.3, 19.4, 17.1.

Minor rotamer (selected signals, ratio ca. 16:1): ^{1}**H NMR** (500 MHz, $CDCl_3$) δ 2.83 (s, 3H), 3.61 (s, 3H), 5.42 (d, *J* = 9.5 Hz, 1H).

HRMS (ESI) calcd for $C_{21}H_{33}N_2O_5^+$ (M+H)$^+$: 393.2384; found: 393.2400.

The spectroscopic data are in agreement with previously published results.[4]

methyl *N*-(((2*R*,3*S*)-2-((*tert*-butoxycarbonyl)amino)-3-methoxy-3-phenylpropanoyl)-D-valyl)-*N*-methyl-D-leucinate (SI-9)

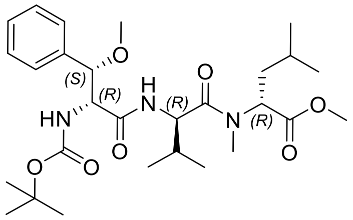

To a solution of methyl **SI-8** (17.0 g, 43.3 mmol) in DCM (85.0 ml, 0.51 M) was added HBr (33% in AcOH) (14.6 g, 91.0 mmol) slowly at 0°C. The mixture was allowed to reach rt and stirred for 2 h. It was degassed with nitrogen to remove excess HBr. The solvents were evaporated in vacuo and the residue was triturated with diethyl ether. The crude amine was used in the peptide coupling without further purification.

To a solution of **SI-7** (17.1 g, 58.1 mmol) in DMF (120 ml, 0.48 M) was added DIPEA (32.8 ml, 194 mmol) dropwise at 0 °C followed by portionwise addition of HATU (22.1 g, 58.1 mmol). After 10 minutes, the deprotected dipeptide prepared as described above (10.0 g, 38.7 mmol) was added. The mixture was allowed to reach rt and stirred for 16. TLC indicated complete conversion. The reaction was quenched with cold water and extracted with EtOAc. The organic layer was washed with brine, dried (Na_2SO_4) and concentrated in vacuo. Silica gel column chromatography (20% EtOAc in petroleum ether) yielded **SI-9** (12.0 g, 22.4 mmol, 58%) as a white solid.

^{1}H NMR (500 MHz, $CDCl_3$) δ 0.91 (d, *J* = 6.5 Hz, 3H), 0.92 – 0.97 (m, 6H), 1.01 (d, *J* = 6.8 Hz, 3H), 1.32 (s, 9H), 1.42 – 1.52 (m, 1H), 1.66 – 1.80 (m, 2H), 2.05 – 2.18 (m, 1H), 3.02 (s, 3H), 3.31 (s, 3H), 3.70 (s, 3H), 4.41 (dd, *J* = 8.5, 3.0 Hz, 1H), 4.87 (dd, *J* = 9.0, 6.2 Hz, 1H), 4.91 (d, *J* = 3.0 Hz, 1H), 5.26 (d, *J* = 8.4 Hz, 1H), 5.33 (dd, *J* = 10.5, 5.2 Hz, 1H), 7.26 – 7.36 (m, 6H).

^{13}C NMR (126 MHz, $CDCl_3$) δ 172.3, 172.2, 169.5, 155.5, 137.4, 128.5, 128.1, 126.8, 81.9, 80.2, 60.0, 57.6, 54.6, 54.1, 52.3, 37.1, 31.7, 31.5, 28.3, 24.9, 23.4, 21.5, 19.6, 17.3.

Minor rotamer (selected signals, ratio ~11:1) **^{1}H NMR** (500 MHz, $CDCl_3$) δ 1.29 (s, 9H), 2.81 (s, 3H), 3.62 (s, 3H), 4.32 – 4.35 (m, 1H), 4.72 (t, *J* = 7.1 Hz, 1H), 5.20 (d, *J* = 9.3 Hz, 1H).

HRMS (ESI) calcd for $C_{28}H_{46}N_3O_7^+$ $(M+H)^+$: 536.3330; found: 536.3347.

The spectroscopic data are in agreement with previously published results.[4]

Methyl *N*-(((2*R*,3*S*)-2-((*R*)-2-((*tert*-butoxycarbonyl)amino)propanamido)-3-methoxy-3-phenylpropanoyl)-D-valyl)-*N*-methyl-D-leucinate (SI-10)

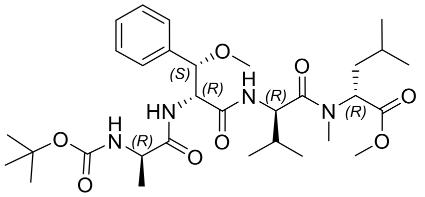

To a solution of **SI-9** (3.00 g, 5.60 mmol) in DCM (15.0 ml, 0.37 M) was added HCl (4 M in dioxane) (5.60 ml, 22.4 mmol) slowly at 0 °C and the mixture was allowed to reach rt and stirred for 4 h. The solvents were evaporated in vacuo and the crude amine was used in the peptide coupling without further purification.

To a solution of Boc-D-Ala-OH (868 mg, 4.59 mmol) in DMF (10.0 ml, 0.46 M) was added DIPEA (3.99 ml, 23.0 mmol) at 0 °C followed by addition of HATU (2.62 g, 6.89 mmol). After 10 minutes, the deprotected tripeptide prepared as described above (2.00 g, 4.59 mmol) was added. The mixture was allowed to reach rt and stirred for 16 . TLC indicated complete conversion. The reaction was quenched with cold water and extracted with EtOAc. The organic layer was washed with brine, dried (Na_2SO_4) and concentrated in vacuo. Silica gel column chromatography (20% EtOAc in petroleum ether) yielded **SI-10** (1.80 g, 2.97 mmol, 60%).

Major rotamer: **^{1}H NMR** (500 MHz, $CDCl_3$) δ 0.91 – 0.94 (m, 6H), 0.96 (d, *J* = 6.7 Hz, 3H), 0.99 (d, *J* = 6.8 Hz, 3H), 1.29 (d, *J* = 7.1 Hz, 3H), 1.45 (s, 9H), 1.70 – 1.77 (m, 3H), 2.09 – 2.18 (m, 1H), 2.99 (s, 3H), 3.33 (s, 3H), 3.69 (s, 3H), 4.08 – 4.18 (m, 1H), 4.68 (dd, *J* = 7.5, 3.6 Hz, 1H), 4.82 (dd, *J* = 8.8, 6.0 Hz, 1H), 4.85 (d, *J* = 3.5 Hz, 1H), 4.88 – 4.95 (m, 1H), 5.36 (dd, *J* = 10.5, 5.3 Hz, 1H), 6.78 (d, *J* = 7.4 Hz, 1H), 7.18 – 7.24 (m, 2H), 7.25 – 7.31 (m, 3H), 7.36 (d, *J*

= 8.8 Hz, 1H). **^{13}C NMR** (126 MHz, $CDCl_3$) δ 17.3, 18.5, 19.7, 21.5, 23.4, 24.9, 28.4, 31.3, 31.5, 37.0, 50.4, 52.3, 54.2, 54.6, 57.6, 57.8, 80.3, 81.3, 127.0, 128.3, 128.4, 136.9, 155.5, 168.5, 172.0, 172.2, 172.5.

Minor rotamer (selected signals, ratio ~15:1): **^{1}H NMR** (500 MHz, $CDCl_3$) δ 1.23 – 1.26 (m, 3H), 1.49 (s, 9H), 2.83 (s, 3H), 3.63 (s, 3H), 4.58 – 4.63 (m, 1H), 6.85 (d, *J* = 8.0 Hz, 1H).

HRMS (ESI) calcd for $C_{31}H_{51}N_4O_8^+$ $(M+H)^+$: 607.3701; found: 607.3726.

The spectroscopic data are in agreement with previously published results.[4]

Methyl (2*R*)-3-[(*tert*-butyldimethylsilyl)oxy]-2-methylpropanoate (SI-11)

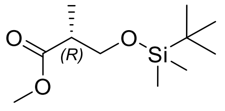

To a stirred solution of (*R*)-3-hydroxy-2-methyl-propionic acid methyl ester (10.0 g, 84.7 mmol) in DCM (100 ml, 0.85 M) were added imidazole (7.48 g, 110 mmol) and TBDMS-Cl (14.1 g, 93.1 mmol) at 0°C. The mixture was stirred at rt. After 3 h, TLC indicated complete conversion. The reaction was quenched with water and extracted with DCM (2x). The combined organic layers were washed with brine, dried (Na_2SO_4) and concentrated in vacuo. Silica gel column chromatography (10% EtOAc in petroleum ether) yielded **SI-11** (19.0 g, 81.8 mmol, 97%).

^{1}H NMR (400 MHz, $CDCl_3$) δ 0.03 (s, 3H), 0.03 (s, 3H), 0.87 (s, 9H), 1.13 (d, *J* = 7.0 Hz, 3H), 2.59-2.70 (m, 1H), 3.62-3.67 (m, 1H), 3.67 (s, 3H), 3.77 (dd, *J* = 9.7, 6.9 Hz, 1H).

For this compound, no mass spectrometric data could be obtained.

The spectroscopic data are in agreement with previously published results.[13]

(2*R*)-3-[(*tert*-butyldimethylsilyl)oxy]-*N*-methoxy-*N*,2-dimethylpropanamide (SI-12)

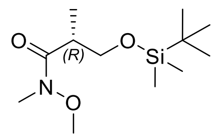

To a stirred solution of **SI-11** (18.0 g, 77.5 mmol) in THF (180 ml, 0.43 M) was added *N,O*-dimethylhydroxylamine hydrochloride (12.1 g, 124 mmol) and it was stirred for 10 minutes at rt, before the mixture was cooled to −30 °C. Then, iPrMgCl (2 M in THF) (116 ml, 232 mmol) was added. The mixture was stirred at −30 °C. After 1 h, TLC indicated complete conversion. The reaction was quenched with aq. NH_4Cl and extracted with EtOAc (2x). The combined organic layers were washed with brine, dried (Na_2SO_4) and concentrated in vacuo. Silica gel column chromatography (5-10% EtOAc in petroleum ether) yielded **SI-12** (20.0 g, 76.5 mmol, 99%) as a colorless liquid.

^{1}H NMR (400 MHz, $CDCl_3$) δ 0.04 (s, 3H), 0.05 (s, 3H), 0.87 (s, 9H), 1.07 (d, *J* = 7.0 Hz, 3H), 3.11-3.19 (m, 1H), 3.19 (s, 3H), 3.53 (dd, *J* = 9.5, 6.1 Hz, 1H), 3.71 (s, 3H), 3.84 (dd, *J* = 9.4, 8.3 Hz, 1H).

MS calcd for $C_{12}H_{28}NO_3Si^+$ $(M+H)^+$: 262.18; found: 262.38.

The spectroscopic data are in agreement with previously published results.[14]

methyl (*S*)-2-(((benzyloxy)carbonyl)amino)-5-((*tert*-butyldimethylsilyl)oxy)-4-methylpent-2-enoate (SI-13)

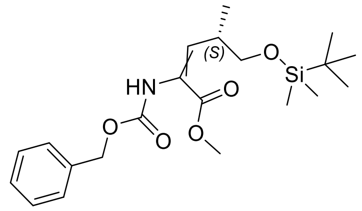

To a stirred solution of **SI-12** (15.0 g, 57.4 mmol) in THF (150 ml, 0.38 M) at 78 °C was added DIBALH (1 M in hexane) (115 ml, 115 mmol) and the mixture was stirred at -78 °C. After 1 h, TLC indicated complete conversion. The reaction was quenched with sat. aq. K-Na-tartrate solution at 0°C and stirred for 2 h at rt. It was extracted with EtOAc (2x). The combined organic layers were washed with brine, dried (Na_2SO_4) and concentrated in vacuo. The crude aldehyde was directly used in the next step without further purification.

To a stirred solution of the crude aldehyde (12 g, 59.3 mmol) in THF (450 ml, 0.13 M) at −78 °C was added 1,1,3,3-tetramethyl guanidine (7.16 g, 62.3 mmol) and stirred for 5 minutes. Then a solution of *N*-Cbz-α-phosphonoglycine trimethyl ester (21.6 g, 65.2 mmol) in THF (450 ml) was added dropwise. The mixture was allowed to reach rt and stirred for 16 h, until TLC indicated complete conversion. It was quenched with water and extracted with EtOAc (2x). The combined organic layers were washed with brine, dried (Na_2SO_4) and concentrated in vacuo. Silica gel column chromatography (10% EtOAc in petroleum ether) yielded **SI-13** (12.0 g, 29.4 mmol, 50%).

^{1}H NMR (400 MHz, $CDCl_3$) δ 0.03 (s, 3H), 0.05 (s, 3H), 0.88 (s, 9H), 1.01 (d, *J* = 6.8 Hz, 3H), 2.73-2.88 (m, 1H), 3.41 (t, *J* = 9.2 Hz, 1H), 3.64 (dd, *J* = 9.6, 4.8 Hz, 1H), 3.67-3.85 (m, 3H), 5.08-5.20 (m, 2H), 6.20 (d, *J* = 9.4 Hz, 1H), 6.97-7.08 (m, 1H), 7.29-7.38 (m, 5H).

MS calcd for $C_{21}H_{34}NO_5Si^+$ $(M+H)^+$: 408.22; found: 408.35.

The spectroscopic data are in agreement with previously published results.[4]

methyl (2*R*,4*S*)-2-{[(benzyloxy)carbonyl]amino}-5-[(*tert*-butyldimethylsilyl)oxy]-4-methylpentanoate (SI-14)

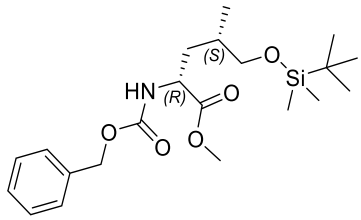

To a stirred solution of **SI-13** (10.0 g, 24.5 mmol) in DCM (350 ml, 0.07 M) under Argon atmosphere was added (*S*)-MonoPhos (705 mg, 1.96 mmol) and [Rh(cod)$_2$BF$_4$] (398 mg, 981 µmol). The resulting mixture was stirred under H_2 (350 psi) in a steel reactor. After 48 h, TLC indicated complete conversion. The mixture was filtered over Celite and the filtrate was concentrated in vacuo. Silica gel column chromatography (7% EtOAc in petroleum ether) yielded **SI-14** (6.40 g, 15.6 mmol, 64%) as a brownish liquid.

^{1}H NMR (400 MHz, $CDCl_3$) δ 0.00 (s, 3H), 0.01 (s, 3H), 0.85 (s, 9H), 0.88 (d, *J* = 6.9 Hz, 3H), 1.49-1.58 (m, 1H), 1.62-1.76 (m, 1H), 1.82-1.95 (m, 1H), 3.35 (dd, *J* = 10.0, 6.1 Hz, 1H), 3.51 (dd, *J* = 9.9, 4.9 Hz, 1H), 4.30-4.45 (m, 1H), 3.70 (s, 3H), 4.98-5.13 (m, 2H), 5.59 (d, *J* = 7.8 Hz, 1H), 7.24-7.36 (m, 5H).

MS found for $C_{21}H_{36}NO_5Si^+$ $(M+H)^+$: 410.24; found: 410.22.

The spectroscopic data are in agreement with previously published results.[4]

(2*R*,4*S*)-2-(((benzyloxy)carbonyl)(methyl)amino)-5-((*tert*-butyldimethylsilyl)oxy)-4-methylpentanoic acid (SI-15)

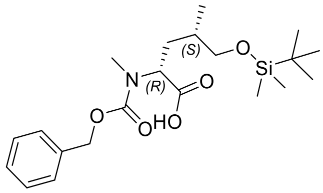

To a stirred solution of **SI-14** (5.00 g, 12.2 mmol) in MeOH (25.0 ml, 0.49 M) was added 1 M aq. NaOH (13.4 ml, 13.4 mmol) at 0°C. The resulting mixture was stirred at rt. After 16 h, TLC and LCMS indicated complete conversion. The mixture was diluted with water and acidified to pH 2-3 with aq. $KHSO_4$ solution. It was extracted with EtOAc (2x). The combined organic layers were washed with brine, dried (Na_2SO_4) and

concentrated in vacuo. The obtained crude Cbz-amino acid was used in the next step without further purification.

To a stirred solution of crude Cbz-amino acid (2.30 g, 5.81 mmol) in THF (50.0 ml, 0.12 M) at −4 °C was added Iodomethane (5.78 g, 40.7 mmol). Then sodium hydride (60%) (558 mg, 14.0 mmol) was added in portions over 10 minutes at -4 °C. The mixture was stirred at the same temperature. After 76 h, TLC and LCMS indicated complete conversion. The reaction was quenched with water and acidified to pH 2-3 with diluted $KHSO_4$ solution. It was extracted with EtOAc (2x), washed with brine, dried (Na_2SO_4) and concentrated in vacuo. Silica gel column chromatography (8% EtOAc in petroleum ether) yielded **SI-15** (1.29 g, 3.15 mmol, 54%).

Major rotamer: **^{1}H NMR** (500 MHz, $CDCl_3$) δ 0.04 (s, 3H), 0.05 (s, 3H), 0.87 – 0.93 (m, 11H), 1.48 – 1.72 (m, 2H), 2.01 – 2.16 (m, 1H), 2.90 (s, 3H), 3.40 (dd, *J* = 10.0, 4.6 Hz, 1H), 3.45 (dd, *J* = 10.1, 5.0 Hz, 1H), 3.50 (d, *J* = 4.9 Hz, 2H), 4.92 (dd, *J* = 10.5, 5.1 Hz, 1H), 5.11 – 5.22 (m, 2H), 7.34 (dd, *J* = 20.9, 3.7 Hz, 5H). **^{13}C NMR** (126 MHz, CDCl3) δ -5.4, -5.3, 17.7, 18.4, 26.0, 30.7, 32.0, 32.5, 56.9, 66.5, 67.7, 127.9, 128.0, 128.6, 136.6, 157.2, 177.7.

Minor rotamer (selected signals, ratio ~3:2): **^{1}H NMR** (500 MHz, $CDCl_3$) δ -0.03 (s, 3H), -0.01 (s, 3H), 0.86 (s, 9H), 0.96 (d, *J* = 6.7 Hz, 3H), 2.92 (s, 3H), 4.78 (dd, *J* = 10.4, 5.0 Hz, 1H). **^{13}C NMR** (126 MHz, $CDCl_3$) δ -5.4, 17.8, 18.4, 26.0, 31.1, 32.2, 32.4, 66.4, 67.8, 128.0, 128.2, 136.4, 156.5.

MS found for $C_{21}H_{36}NO_5Si^+$ $(M+H)^+$: 410.24; found: 410.32

The spectroscopic data are in agreement with previously published results.[4]

Methyl *N*-(((2*R*,3*S*)-2-((*R*)-2-((2*R*,4*S*)-2-(((benzyloxy)carbonyl)(methyl)amino)-5-((*tert*-butyldimethylsilyl)oxy)-4-methylpentanamido)propanamido)-3-methoxy-3-phenylpropanoyl)-D-valyl)-*N*-methyl-D-leucinate (2a)

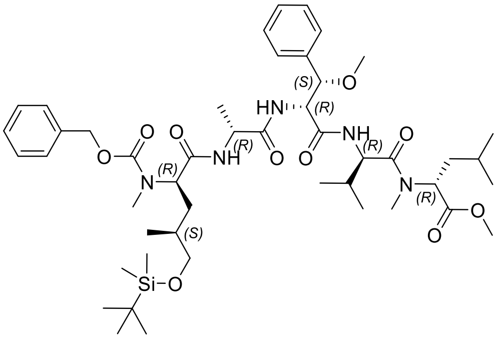

To a solution of **SI-10** (2.00 g, 3.30 mmol) in DCM (10.0 ml, 0.33 M) was added HCl (4 M in dioxane) (4.12 ml, 16.5 mmol) at 0 °C and the mixture was allowed to reach rt and stirred for 3 h. The solvents were evaporated in vacuo, the residue was triturated with petroleum ether and the crude amine was used in the peptide coupling without further purification.

To a solution of deprotected tetrapeptide as described above (1.50 g, 2.96 mmol) in DMF (15.0 ml, 0.20 M) were added DIPEA (2.57 ml, 14.8 mmol), HATU (1.69 g, 4.44 mmol) and **SI-15** (1.21 g, 2.96 mmol). The mixture was stirred for 16 . TLC and LCMS indicated complete conversion. The reaction was quenched with cold water and extracted with EtOAc. The organic layer was washed with brine, dried (Na_2SO_4) and concentrated in vacuo. Silica gel column chromatography (10-100% EtOAc in petroleum ether) yielded **2a** (2.00 g, 2.23 mmol, 75%) as a light brownish solid.

$[\alpha]_{20}^{D}$ = +58.0 (c 1.0, $CHCl_3$).

Major rotamer: **^{1}H NMR** (500 MHz, $CDCl_3$) δ -0.02 – 0.08 (m, 6H), 0.88 (s, 9H), 0.90 – 0.97 (m, 12H), 1.00 (d, *J* = 6.8 Hz, 3H), 1.23 (d, *J* = 7.0 Hz, 3H), 1.46 – 1.52 (m, 1H), 1.53 – 1.63 (m, 2H), 1.66 – 1.79 (m, 2H), 1.92 – 2.02 (m, 1H), 2.08 – 2.16 (m, 1H), 2.84 (s, 3H), 2.98 (s, 3H), 3.32 (s, 3H), 3.39 – 3.45 (m, 1H), 3.49 – 3.55 (m, 1H), 3.69 (s, 3H), 4.30 – 4.38 (m, 1H), 4.67 (dd, *J* = 7.5, 3.6 Hz, 1H), 4.69 – 4.75 (m, 1H), 4.78 – 4.84 (m, 2H), 5.11 – 5.21 (m, 2H), 5.35 (dd, *J* = 10.6, 5.1 Hz, 1H), 6.43 – 6.51 (m, 1H), 6.66 (d, *J* = 7.2 Hz, 1H), 7.11 – 7.21 (m, 2H), 7.29 – 7.38 (m, 9H). **^{13}C NMR** (126 MHz, $CDCl_3$) δ -5.3, 17.3, 17.6, 18.1, 18.5, 19.7, 21.5, 23.4, 24.9, 26.1, 30.0, 31.2, 31.4, 31.6,

32.3, 37.0, 49.3, 52.3, 54.2, 54.6, 56.9, 57.7, 57.9, 67.2, 67.8, 81.3, 126.9, 127.9, 128.3, 128.4, 128.5, 128.7, 136.6, 136.8, 157.4, 168.4, 170.9, 171.6, 172.1, 172.2.

Minor rotamer (ratio ~2:1, selected signals) **^{1}H NMR** (500 MHz, $CDCl_3$) δ 4.59 (s, 1H), 6.16 – 6.21 (m, 1H), 6.55 – 6.61 (m, 1H).

HRMS (ESI): calcd for $C_{47}H_{76}N_5O_{10}Si^+$ $(M+H)^+$: 898.5356; found: 898.5321.

((Allyloxy)carbonyl)-D-tryptophan (SI-16)

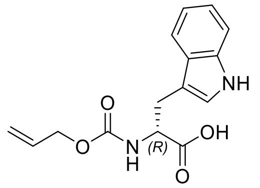

D-Tryptophan (10.0 g, 49.1 mmol) and Na_2CO_3 (10.3 g, 123 mmol) were dissolve in a mixture of THF (100 ml) and water (123 ml). The mixture was cooled to 0 °C and a solution of allyl chloroformate (5.23 ml, 49.1 mmol) in THF (22 ml) was added dropwise. The resulting solution was slowly warmed to rt over the course of 18 h. The solvent was concentrated in vacuo, the residue was dissolved in EtOAc and washed with 1 M HCl. The aqueous phase was extracted with EtOAc (3x). The combined organic phases were washed with brine, dried (Na_2SO_4) and evaporated. Recrystallization from pentane/Et_2O yielded **SI-16** (11.5 g, 39.7 mmol, 81%) as a white solid.
$[\alpha]_{20}^{D}$ = –40.6 (c 0.5, $CHCl_3$).

Melting point: 122 – 124 °C

^{1}H NMR (400 MHz, DMSO) δ 2.98 (dd, *J* = 14.6, 9.7 Hz, 1H), 3.17 (dd, *J* = 14.6, 4.6 Hz, 1H), 4.20 (ddd, *J* = 9.7, 8.1, 4.6 Hz, 1H), 4.34 – 4.48 (m, 2H), 5.14 (dd, *J* = 10.5, 1.6 Hz, 1H), 5.24 (dd, *J* = 17.2, 1.8 Hz, 1H), 5.85 (ddt, *J* = 17.3, 10.5, 5.3 Hz, 1H), 6.95 – 7.01 (m, 1H), 7.04 – 7.09 (m, 1H), 7.15 (d, *J* = 2.3 Hz, 1H), 7.34 (d, *J* = 8.0 Hz, 1H), 7.50 (d, *J* = 8.2 Hz, 1H), 7.53 (d, *J* = 7.9 Hz, 1H), 10.83 (s, 1H), 12.65 (bs, 1H).

^{13}C NMR (101 MHz, DMSO) δ 26.9, 54.9, 64.4, 110.1, 111.4, 117.0, 118.1, 118.4, 120.9, 123.7, 127.1, 133.5, 136.1, 155.8, 173.7.

HRMS (CI): calcd for $C_{15}H_{17}N_2O_4^+$ $(M+H)^+$: 289.1183; found: 289.1196.

***N*$^{\alpha}$-((allyloxy)carbonyl)-1-(prop-2-yn-1-yl)-D-tryptophan (SI-17)**

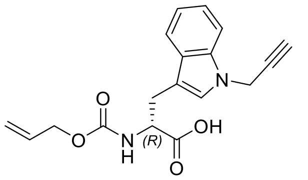

SI-16 (1.44 g, 5.00 mmol) was dissolved in DMF (10.0 ml) under N_2. KO*t*-Bu (1.18 g, 10.5 mmol) was added at rt and the mixture was stirred until all solids were dissolved, resulting ina a pale yellow color. After cooling to 0 °C, propargyl bromide (0.754 ml, 7.00 mmol) was added in one portion. TLC control indicated full conversion after 15 min. The reaction mixture was quenched after 23 min by addition of 1 M HCl and then extracted with EtOAc. The organic phase was subsequently washed with 1 M HCl (2x), H_2O (2x) and brine (1x), dried (Na_2SO_4) and evaporated. The residue was purified by flash chromatography (CyH/[EtOAc+2%HOAc] 100:0 - 6:4), which yielded **SI-17** (1.15 g, 3.53 mmol, 71%) as an orange-red resin. R_f = 0.28 (PE/EtOAc/HOAc 70:30:1).

$[\alpha]_{20}^{D}$ = -38.1 (c 1.0, $CHCl_3$).

^{1}H NMR (400 MHz, DMSO) δ 3.00 (dd, *J* = 14.7, 9.7 Hz, 1H), 3.18 (dd, *J* = 14.7, 4.6 Hz, 1H), 3.37 (t, *J* = 2.5 Hz, 1H), 4.22 (ddd, *J* = 9.7, 8.1, 4.5 Hz, 1H), 4.36 – 4.50 (m, 2H), 5.03 (d, *J* = 2.5 Hz, 2H), 5.15 (dd, *J* = 10.5, 1.6 Hz, 1H), 5.25 (dd, *J* = 17.2, 1.7 Hz, 1H), 5.86 (ddt, *J* = 17.3, 10.5, 5.3 Hz, 1H), 7.07 (t, *J* = 6.9 Hz, 1H), 7.13 – 7.22 (m, 1H), 7.23 (s, 1H), 7.47 (d, *J* = 8.2 Hz, 1H), 7.54 – 7.62 (m, 2H), 12.61 (bs, 1H).

^{13}**C NMR** (101 MHz, DMSO) δ 21.1, 26.7, 35.0, 54.8, 64.4, 75.5, 79.2, 79.3, 110.0, 110.6, 117.0, 118.6, 119.1, 121.4, 126.6, 127.9, 133.5, 135.7, 155.9, 173.6.

HRMS (CI): calcd for $C_{18}H_{18}N_2O_4^+$ (M)$^+$ 326.1261; found: 326.1267.

methyl *N*-(((2*R*,3*R*)-2-((*R*)-2-((2*R*,4*S*)-2-((*R*)-2-(((allyloxy)carbonyl)amino)-*N*-methyl-3-(1-(prop-2-yn-1-yl)-1*H*-indol-3-yl)propanamido)-5-((*tert*-butyldimethylsilyl)oxy)-4-methylpentanamido)propanamido)-3-methoxy-3-phenylpropanoyl)-D-valyl)-*N*-methyl-D-leucinate (3a)

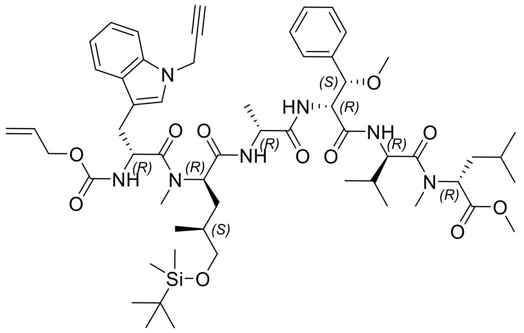

Prepared according to *GP1* and *GP5*: **2a** (371 mg, 413 µmol), Pd/C (22.0 mg) (2 h); **SI-17** (189 mg, 0.578 mmol), DIPEA (101 µl, 578 µmol), $BnNMe_2$ (9.20 µl, 61.9 µmol), 1 M isopropyl chloroformate (578 µl, 578 µmol), NMI (3.29 µl, 41.3 µmol) and 4 M HCl (10.3 µl, 41.2 µmol) (2.5 h). Flash chromatography (CyH/EtOAc 100:0 – 40:60) followed by lyophilization afforded **3a** (353 mg, 329 µmol, 80%) as a white amorphous solid. R_f = 0.25 (PE/EtOAc 4:6).

$[\alpha]_{20}^{D}$ = +52.1 (c 1.0, $CHCl_3$).

^{1}**H NMR** (500 MHz, $CDCl_3$) *(mixture of rotamers, ratio ~3:2)* δ -0.28 – -0.18 (m, 0.3H), -0.05 (s, 0.9H), -0.03 (s, 0.9H), 0.02 (s, 2.2H), 0.03 (s, 2.3H), 0.40 (d, *J* = 6.6 Hz, 0.8H), 0.83 (s, 2.4H), 0.88 (s, 6.2H), 0.87 – 0.98 (m, 14.4H), 0.99 (d, *J* = 6.8 Hz, 2.7H), 1.19 (d, *J* = 7.1 Hz, 1.8H), 1.22 – 1.27 (m, 1.9H), 1.43 – 1.53 (m, 2.6H), 1.65 – 1.81 (m, 2.4H), 1.85 – 1.92 (m, 0.4H), 1.94 – 2.04 (m, 1.0H), 2.08 – 2.21 (m, 1.3H), 2.36 – 2.42 (m, 1.0H), 2.73 (s, 1.9H), 2.75 (s, 1.2H), 2.97 (s, 1.0H), 3.01 (s, 2.0H), 3.14 (dd, *J* = 13.9, 5.8 Hz, 1.0H), 3.21 (dd, *J* = 14.4, 8.1 Hz, 0.8H), 3.28 (s, 1.2H), 3.33 (s, 2.8H), 3.40 (dd, *J* = 9.9, 5.4 Hz, 0.8H), 3.45 (dd, *J* = 9.8, 4.7 Hz, 0.8H), 3.67 – 3.71 (m, 3.4H), 4.16 – 4.23 (m, 0.6H), 4.25 – 4.32 (m, 0.4H), 4.47 – 4.59 (m, 2.2H), 4.63 (dd, *J* = 7.6, 3.5 Hz, 0.7H), 4.69 – 4.72 (m, 1H), 4.75 – 4.91 (m, 5.6H), 4.95 – 5.02 (m, 0.8H), 5.15 – 5.32 (m, 2.2H), 5.32 – 5.41 (m, 1.1H), 5.61 (d, *J* = 7.6 Hz, 0.3H), 5.81 – 5.92 (m, 1.5H), 6.34 (d, *J* = 6.8 Hz, 0.6H), 6.74 (d, *J* = 7.6 Hz, 0.3H), 6.78 (d, *J* = 7.7 Hz, 0.6H), 7.04 (s, 1.0H), 7.11 – 7.25 (m, 5.7H), 7.27 – 7.32 (m, 2.5H), 7.36 (d, *J* = 8.4 Hz, 1.6H), 7.44 (d, *J* = 8.7 Hz, 0.7H), 7.58 (d, *J* = 7.9 Hz, 0.4H), 7.67 (d, *J* = 7.9 Hz, 0.7H), 7.83 (d, *J* = 6.8 Hz, 0.3H).

^{13}**C-NMR** (126 MHz, $CDCl_3$) *(mixture of rotamers)* δ -5.0, 16.0, 17.6, 17.8, 18.2, 18.7, 19.9, 21.8, 23.7, 25.2, 26.3, 26.4, 29.2, 31.5, 31.7, 32.5, 36.1, 37.3, 50.1, 50.2, 51.8, 52.5, 54.5, 54.6, 54.9, 56.6, 58.0, 58.3, 66.1, 66.7, 67.6, 68.7, 74.0, 81.5, 81.8, 109.9, 110.0, 110.2, 118.0, 118.7, 119.3, 120.3, 122.6, 122.9, 126.5, 127.3, 128.57, 128.63, 128.8, 133.1, 136.3, 136.5, 137.3, 156.3, 157.0, 168.9, 169.1, 170.7, 171.9, 172.46, 172.52, 173.7.

Selected diagnostic peaks:

Major rotamer: ^{1}**H NMR** (500 MHz, $CDCl_3$) δ 0.02 (s, 3H), 0.03 (s, 3H), 0.88 (s, 9H), 1.85 – 1.92 (m, 0H), 2.39 (t, *J* = 2.1 Hz, 1H), 2.73 (s, 3H), 3.01 (s, 3H), 3.14 (dd, *J* = 13.9, 5.8 Hz, 1H), 3.21 (dd, *J* = 14.4, 8.1 Hz, 1H), 3.33 (s, 3H), 3.69 (s, 3H), 4.16 – 4.23 (m, 1H), 6.34 (d, *J* = 6.8 Hz, 1H), 6.78 (d, *J* = 7.7 Hz, 1H), 7.04 (s, 1H), 7.67 (d, *J* = 7.9 Hz, 1H). ^{13}**C-NMR** (126 MHz, $CDCl_3$) δ 26.4, 31.7, 37.3, 52.5, 81.8.

Minor rotamer: ^{1}**H NMR** (500 MHz, $CDCl_3$) δ -0.28 – -0.18 (m, 1H), -0.05 (s, 3H), -0.03 (s, 3H), 0.40 (d, *J* = 6.6 Hz, 3H), 0.83 (s, 9H), 2.38 (t, *J* = 2.1 Hz, 1H), 2.75 (s, 3H), 2.97 (s, 3H), 3.28 (s, 3H), 3.68 (s, 3H), 4.26 – 4.30 (m, 1H), 5.61 (d, *J* = 7.6 Hz, 1H), 6.74 (d, *J* = 7.6 Hz, 1H). ^{13}**C-NMR** (126 MHz, $CDCl_3$) δ 16.0, 26.3, 29.2.

HRMS (ESI): calcd for $C_{62}H_{95}N_8O_{12}Si^+$ (M+H)$^+$ 1171.6833; found: 1171.6830.

methyl *N*-(((2*R*,3*S*)-2-((*R*)-2-((2*R*,4*S*)-2-((*R*)-2-((*R*)-2-(((allyloxy)carbonyl)amino)-3-methylbutanamido)-*N*-methyl-3-(1-(prop-2-yn-1-yl)-1*H*-indol-3-yl)propanamido)-5-((*tert*-butyldimethylsilyl)oxy)-4-methylpentanamido)propanamido)-3-methoxy-3-phenylpropanoyl)-D-valyl)-*N*-methyl-D-leucinate (4a)

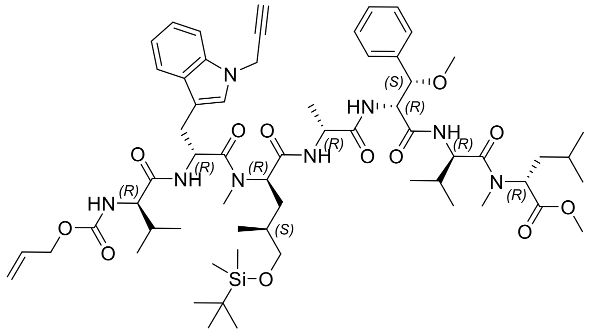

Prepared according to *GP2* and *GP4*: **3a** (326 mg, 304 μmol), DMBA (142 mg, 912 μmol), $Pd(PPh_3)_4$ (10.5 mg, 9.12 μmol) (1 h); ((allyloxy)carbonyl)-D-valine (84.0 mg, 395 mmol), HOBt (65.2 mg, 426 μmol), EDC (82.0 mg, 426 μmol), NMM (84.0 μl, 760 μmol) (15 h). Flash chromatography (CyH/EtOAc 100:0 – 20:80) followed by lyohpilization afforded **4a** (305 mg, 260 μmol, 86%) as a white amorphous solid. R_f = 0.23 (PE/EtOAc 3:7).

$[\alpha]_{20}^{D}$ = +68.3 (c 0.5, $CHCl_3$)

^{1}H-NMR (500 MHz, $CDCl_3$) *(mixture of rotamers, ratio ~2:1)* δ -0.05 (s, 1.1H), -0.04 (s, 1.0H), 0.01 (s, 2.1H), 0.02 (s, 2.0H), 0.37 (d, *J* = 6.6 Hz, 0.8H), -0.82 (s, 4.9H), 0.85 – 0.88 (m, 8.1H), 0.88 – 0.97 (m, 18.2 H), 0.99 (d, *J* = 6.9 Hz, 2.6H), 1.20 (d, *J* = 7.3 Hz, 2.8H), 1.26 (d, *J* = 6.3 Hz, 0.9H), 1.38 – 1.53 (m, 2.6H), 1.65 – 1.80 (m, 2.4H), 2.02 – 2.15 (m, 2.1H), 2.38 – 2.41 (m, 1.0H), 2.71 (s, 1.0 H), 2.75 (s, 2.1 H), 2.97 (s, 1.2 H), 2.98 – 2.99 (m, 2.3 H), 3.03 – 3.07 (m, 0.4H), 3.09 – 3.16 (m, 1.1H), 3.18 – 3.25 (m, 0.9H), 3.28 (s, 1.2H), 3.32 (s, 2.6H), 3.41 (d, *J* = 5.0 Hz, 1.4H), 3.66 – 3.70 (m, 3.5H), 4.06 – 4.14 (m, 0.9H), 4.24 – 4.31 (m, 0.7H), 4.33 – 4.39 (m, 0.5H), 4.51 – 4.65 (m, 2.4H), 4.67 – 4.73 (m, 1.6H), 4.76 – 4.86 (m, 4.3H), 4.90 – 5.00 (m, 1.1H), 5.17 – 5.39 (m, 4.5H), 5.70 (d, *J* = 9.5 Hz, 0.2H), 5.85 – 5.98 (m, 1.0H), 6.47 (d, *J* = 6.0 Hz, 0.6H), 6.79 (d, *J* = 7.4 Hz, 0.5H), 6.82 (d, *J* = 7.3 Hz, 0.4H), 6.97 (d, *J* = 7.4 Hz, 0.6H), 7.01 – 7.05 (m, 1.0H), 7.10 – 7.18 (m, 2.0H), 7.19 – 7.25 (m, 4.1H), 7.27 – 7.29 (m, 1.5H), 7.32 – 7.37 (m, 1.8H), 8.13 (d, *J* = 7.3 Hz, 0.2H).

^{13}C NMR (126 MHz, $CDCl_3$) *(mixture of rotamers)* δ -5.31, -5.28, -5.2, 15.7, 17.40, 17.42, 17.8, 18.0, 18.2, 18.40, 18.45, 19.2, 19.6, 21.5, 21.6, 23.4, 24.9, 26.1, 28.1, 28.7, 29.3, 31.3, 31.4, 31.5, 31.7, 32.3, 35.8, 37.1, 49.4, 49.9, 52.2, 54.2, 54.6, 54.7, 57.55, 57.61, 57.7, 58.0, 58.2, 60.1, 65.9, 66.1, 67.2, 68.5, 73.7, 73.8, 77.8, 78.1, 81.2, 81.5, 109.5, 109.6, 109.8, 117.9, 118.0, 119.0, 120.0, 120.2, 122.4, 122.6, 126.3, 127.1, 128.2, 128.3, 128.5, 128.6, 128.7, 132.0, 132.2, 132.3, 132.8, 136.0, 136.2, 136.9, 156.2, 156.4, 168.5, 170.4, 170.9, 171.6, 171.7, 171.9, 172.1, 172.2, 172.6, 172.8.

Selected diagnostic peaks:

Major rotamer: **^{1}H NMR** (500 MHz, $CDCl_3$) δ 0.01 (s, 3H), 0.02 (s, 3H), 0.86 (s, 9H), 1.20 (d, *J* = 7.3 Hz, 3H), 2.39 (t, *J* = 2.5 Hz, 1H), 2.75 (s, 3H), 2.98 (s, 3H), 3.32 (s, 3H), 3.69 (s, 3H), 4.28 (t, *J* = 7.0 Hz, 1H). **^{13}C NMR** (126 MHz, $CDCl_3$) δ 26.1, 32.3, 49.4, 57.61, 156.2.

Minor rotamer: **^{1}H NMR** (500 MHz, $CDCl_3$) δ -0.05 (s, 3H), -0.04 (s, 3H), 0.37 (d, *J* = 6.6 Hz, 3H), 0.82 (s, 9H), 1.26 (d, *J* = 6.3 Hz, 3H), 2.39 (t, *J* = 2.5 Hz, 1H), 2.71 (s, 3H), 2.97 (s, 3H), 3.28 (s, 3H). **^{13}C NMR** (126 MHz, $CDCl_3$) δ 29.3, 49.9, 156.4.

HRMS (ESI): calcd for $C_{62}H_{95}N_8O_{12}Si^+$ $(M+H)^+$: 1171.6833; found: 1171.6847.

(3*R*,6*R*,9*R*,12*R*,15*R*,18*R*,21*R*)-21-((*S*)-3-hydroxy-2-methylpropyl)-9-isobutyl-6,12-diisopropyl-15-((*S*)-methoxy(phenyl)methyl)-1,10,18-trimethyl-3-((1-(prop-2-yn-1-yl)-1*H*-indol-3-yl)methyl)-1,4,7,10,13,16,19-heptaazacyclohenicosane-2,5,8,11,14,17,20-heptaone (5a)

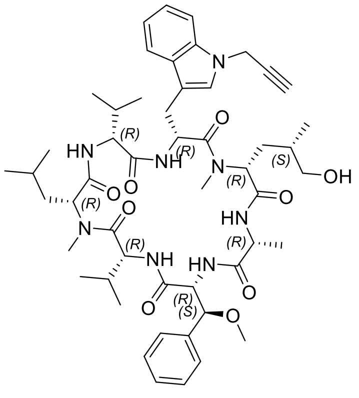

Prepared according to *GP6a*: **4a** (200 mg, 171 µmol), 1 M LiOH (222 µl, 222 µmol) (4.5 h); $Pd(OAc)_2$ (1.9 mg, 8.6 µmol), TPPTS (9.7 mg, 17 µmol), Et_2NH (179 µl, 1.71 mmol) (1 h); HATU (293 mg, 770 µmol), DIPEA (179 µl, 1.03 mmol) (addition over 3 h, additional 17 h); NH_4F (127 mg, 3.42 mmol) (16 h). RP flash chromatography (H_2O/MeCN 90:10 - 5:95) followed by prep HPLC (H_2O/MeCN 80:20 – 5:95) and lyophilizaiton afforded **5a** (46.1 mg, 49.0 µmol, 29%) as a white, amorphous solid.

$[\boldsymbol{\alpha}]_{\mathbf{20}}^{\mathbf{D}}$ = +107.2 (c 0.5, $CHCl_3$).

^{1}H NMR (500 MHz, $CDCl_3$) δ -0.61 (dd, *J* = 11.0, 6.6 Hz, 1H), 0.21 (d, *J* = 6.8 Hz, 3H), 0.59 (d, *J* = 6.5 Hz, 3H), 0.62 (d, *J* = 6.6 Hz, 3H), 0.68 – 0.78 (m, 2H), 0.97 (dq, *J* = 10.7, 3.9 Hz, 10H), 1.07 (d, *J* = 6.6 Hz, 3H), 1.09 – 1.19 (m, 4H), 1.53 – 1.62 (m, 0H), 1.83 (ddd, *J* = 13.6, 11.0, 7.2 Hz, 1H), 2.17 – 2.28 (m, 1H), 2.27 – 2.37 (m, 1H), 2.41 (t, *J* = 2.6 Hz, 1H), 2.59 (s, 3H), 2.59 – 2.66 (m, 1H), 2.83 (s, 3H), 2.89 – 2.95 (m, 1H), 2.97 – 3.02 (m, 1H), 3.12 (dd, *J* = 13.5, 4.7 Hz, 1H), 3.27 – 3.33 (m, 1H), 3.35 (s, 3H), 4.09 (t, *J* = 9.5 Hz, 1H), 4.32 (d, *J* = 8.7 Hz, 1H), 4.48 (t, *J* = 8.6 Hz, 1H), 4.72 – 4.83 (m, 4H), 4.85 (dd, *J* = 9.6, 3.2 Hz, 1H), 4.91 (t, *J* = 5.1 Hz, 1H), 5.07 (d, *J* = 5.5 Hz, 1H), 7.00 (s, 1H), 7.10 – 7.14 (m, 1H), 7.15 – 7.18 (m, 2H), 7.23 (d, *J* = 4.9 Hz, 5H), 7.34 (d, *J* = 8.4 Hz, 2H), 7.47 (d, *J* = 7.9 Hz, 1H), 8.13 (d, *J* = 8.0 Hz, 1H), 8.27 (d, *J* = 9.5 Hz, 1H), 8.51 (d, *J* = 10.4 Hz, 1H).

^{13}C NMR (126 MHz, $CDCl_3$) δ 17.0, 18.5, 19.2, 19.9, 20.6, 22.5, 23.4, 25.1, 28.1, 29.0, 29.4, 30.7, 31.6, 32.9, 35.5, 38.8, 50.3, 50.9, 55.1, 55.8, 57.7, 58.8, 58.9, 59.1, 65.9, 73.8, 77.4, 79.9, 108.7, 109.6, 118.6, 120.1, 122.6, 126.2, 127.8, 128.0, 128.0, 128.5, 134.9, 135.8, 168.5, 168.9, 169.8, 170.5, 171.2, 171.4, 172.0.

HRMS (ESI): calcd for $C_{51}H_{73}N_8O_9^+$ $(M+H)^+$: 941.5495; found: 941.5504.

(3*R*,3'*R*,6*R*,6'*R*,9*R*,9'*R*,12*R*,12'*R*,15*R*,15'*R*,18*R*,18'*R*,21*R*,21'*R*)-15,15'-(((((3,6,9,12-tetraoxatetradecane-1,14-diyl)bis(1H-1,2,3-triazole-1,4-diyl))bis(methylene))bis(1*H*-indole-1,3-diyl))bis(methylene))bis(12-((S)-3-hydroxy-2-methylpropyl)-21-isobutyl-3,18-diisopropyl-6-((S)-methoxy(phenyl)methyl)-1,9,13-trimethyl-1,4,7,10,13,16,19-heptaazacyclohenicosane-2,5,8,11,14,17,20-heptaone) (8a) (UdSBI-0966)

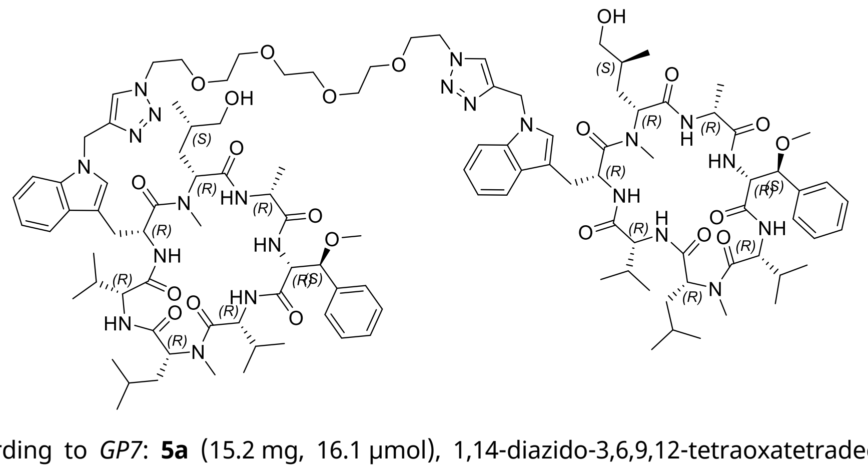

Prepared according to *GP7*: **5a** (15.2 mg, 16.1 µmol), 1,14-diazido-3,6,9,12-tetraoxatetradecane (2.33 mg, 8.07 µmol), 1 M $CuSO_4$ (6.46 µl, 6.46 µmol), 1 M sodium ascorbate (10.5 µl, 10.5 µmol) (16 h). RP flash chromatography (H_2O/MeCN 70:30 – 90:10) followed by prep HPLC (H_2O/MeCN 70:30 – 90:10) and lyophilization afforded **8a** (9.0 mg, 4.15 µmol, 51%) as a white, amorphous solid.

$[\alpha]_{20}^{D}$ = +95.6 (c 0.25, $CHCl_3$).

^{1}H NMR (500 MHz, $CDCl_3$) δ -0.70 – -0.53 (m, 2H), 0.15 (d, *J* = 6.8 Hz, 6H), 0.58 (d, *J* = 6.5 Hz, 6H), 0.63 (d, *J* = 6.6 Hz, 6H), 0.72 – 0.82 (m, 2H), 0.94 – 1.01 (m, 18H), 1.10 (d, *J* = 6.6 Hz, 6H), 1.12 – 1.19 (m, 8H), 1.54 – 1.61 (m, 2H), 1.71 (d, *J* = 5.4 Hz, 2H), 2.19 – 2.27 (m, 2H), 2.27 – 2.35 (m, 2H), 2.56 (s, 6H), 2.79 – 2.88 (m, 8H), 3.02 (dd, *J* = 11.5, 4.4 Hz, 2H), 3.07 (dd, *J* = 13.8, 5.0 Hz, 2H), 3.25 – 3.30 (m, 2H), 3.35 – 3.39 (m, 10H), 3.43 (dd, *J* = 11.0, 4.4 Hz, 2H), 3.47 – 3.52 (m, 4H), 3.79 (t, *J* = 5.4 Hz, 4H), 4.11 (t, *J* = 9.3 Hz, 2H), 4.36 (d, *J* = 12.5 Hz, 2H), 4.39 – 4.52 (m, 8H), 4.69 – 4.81 (m, 4H), 4.86 (dd, *J* = 10.1, 3.8 Hz, 2H), 4.90 (t, *J* = 5.0 Hz, 2H), 5.09 (d, *J* = 5.5 Hz, 2H), 5.27 (d, *J* = 15.4 Hz, 2H), 5.38 (d, *J* = 15.6 Hz, 2H), 7.02 (s, 2H), 7.09 (t, *J* = 7.5 Hz, 2H), 7.15 (d, *J* = 5.0 Hz, 2H), 7.17 – 7.26 (m, 16H), 7.32 (s, 2H), 7.42 (d, *J* = 8.2 Hz, 2H), 7.46 (d, *J* = 8.0 Hz, 2H), 7.56 (s, 2H), 8.09 (d, *J* = 8.4 Hz, 2H), 8.24 (d, *J* = 9.5 Hz, 2H), 8.37 (d, *J* = 10.4 Hz, 2H).

^{13}C NMR (126 MHz, $CDCl_3$) δ 16.9, 18.9, 19.4, 20.0, 20.2, 20.8, 22.7, 23.7, 25.5, 28.6, 29.3, 29.6, 31.1, 32.0, 32.9, 39.2, 41.7, 50.5, 50.6, 51.2, 55.5, 56.2, 57.9, 59.1, 59.3, 66.8, 69.5, 70.7, 80.4, 108.9, 110.2, 118.9, 120.1, 122.7, 123.4, 127.1, 128.1, 128.4 , 128.8, 135.4, 136.5, 143.8, 168.8, 169.0, 170.1, 170.9, 171.6, 171.8, 172.4.

HRMS (ESI): calcd for $C_{112}H_{165}N_{22}O_{22}^+$ (M+H)$^+$: 2170.2463; found: 2170.2477.

Synthesis of exit vector 7 triazol-based Homo-BacPROTACs (11 + 12)

Methyl *N*-(((2*S*,3*R*)-2-((*S*)-2-((2*S*,4*R*)-2-((*S*)-2-((*S*)-2-((((9*H*-fluoren-9-yl)methoxy)carbonyl)amino)pent-4-ynamido)-*N*-methyl-3-(1-methyl-1*H*-indol-3-yl)propanamido)-5-((*tert*-butyldimethylsilyl)oxy)-4-methylpentanamido)propanamido)-3-methoxy-3-phenylpropanoyl)-L-valyl)-*N*-methyl-L-leucinate (SI-18)

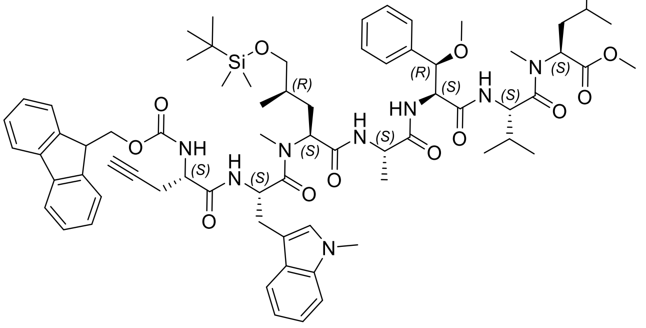

Prepared according to *GP2* and *GP4*: methyl N-(((2S,3R)-2-((S)-2-((2S,4R)-2-((S)-2-(((allyloxy)carbonyl)amino)-N-methyl-3-(1-methyl-1H-indol-3-yl)propanamido)-5-((tert-butyldimethylsilyl)oxy)-4-methylpentanamido)propanamido)-3-methoxy-3-phenylpropanoyl)-L-valyl)-N-methyl-L-leucinate (377 mg, 360 µmol), DMBA (169 mg, 1.08 mmol), $Pd(PPh_3)_4$ (12.5 mg, 10.8 µmol) (1.5 h); Fmoc-Pra-OH (157 mg, 468 µmol), HOBt (60.6 mg, 396 µmol), EDC (75.9 mg, 396 µmol), NMM (79.2 µl, 720 µmol) (17 h). Flash chromatography (CyH/EtOAc 100:0 – 20:8) followed by lyophilization yielded **SI-18** (424 mg, 329 µmol, 91%) as a white amorphous solid. R_f = 0.21 (PE/EtOAc 4:6).

$[\alpha]_{20}^{D}$ = –58.5 (c 0.5, $CHCl_3$)

^{1}H NMR (500 MHz, $CDCl_3$) *(mixture of rotamers)* δ -0.04 (s, 0.7H), -0.02 (s, 0.7H), 0.00 – 0.05 (m, 4.5H), 0.49 (d, *J* = 6.7 Hz, 0.5H), 0.84 (s, 2.1H), 0.86 – 0.89 (m, 9.6H), 0.89 – 0.96 (m, 9.5H), 0.99 (d, *J* = 6.8 Hz, 2.4H), 1.19 – 1.36 (m, 4.6H), 1.42 – 1.52 (m, 2.2H), 1.63 – 1.80 (m, 2.1H), 1.91 – 1.97 (m, 0.5H), 1.99 – 2.09 (m, 1.8H), 2.09 – 2.18 (m, 1.0H), 2.49 – 2.71 (m, 1.8H), 2.74 (s, 0.7H), 2.76 – 2.86 (m, 2.2H), 2.93 – 3.01 (m, 3.0H), 3.06 – 3.11 (m, 0.3H), 3.11 – 3.20 (m, 1.0H), 3.24 – 3.36 (m, 4.2H), 3.38 – 3.45 (m, 1.3H), m, 1.3H), 3.64 – 3.71 (m, 5.8H), 4.19 – 4.25 (m, 1.0H), 4.25 – 4.30 (m, 0.7H), 4.30 – 4.49 (m, 3.0H), 4.65 – 4.86 (m, 3.7H), 4.99 – 5.07 (m, 0.3H), 5.21 – 5.29 (m, 0.7H), 5.29 – 5.39 (m, 1.0H), 5.62 (d, *J* = 8.3 Hz, 0.5H), 5.70 (d, *J* = 8.5 Hz, 0.2H), 6.42 – 6.50 (m, 0.5H), 6.72 – 6.81 (m, 0.3H), 6.85 – 6.91 (m, 1.0H), 6.94 – 7.00 (m, 0.4H), 7.07 – 7.12 (m, 1.0H), 7.14 – 7.25 (m, 5.0H), 7.26 – 7.35 (m, 4.5H), 7.37 – 7.44 (m, 2.6H), 7.44 – 7.49 (m, 1.0H), 7.52 – 7.57 (m, 0.8H), 7.57 – 7.63 (m, 1.7H), 7.64 – 7.70 (m, 1.4H), 7.74 – 7.80 (m, 1.9 H), 7.90 (d, *J* = 6.7 Hz, 0.2H).

^{13}C NMR (126 MHz, $CDCl_3$) *(mixture of rotamers)* δ -5.3, 17.4, 17.5, 18.0, 18.4, 18.5, 19.6, 19.7, 21.5, 21.6, 23.1, 23.4, 24.9, 26.0, 26.1, 28.6, 29.3, 31.4, 31.5, 32.3, 32.8, 37.0, 47.2, 49.6, 50.5, 52.3, 53.1, 54.2, 54.6, 57.6, 58.3, 67.3, 67.4, 68.4, 72.0, 79.2, 81.5, 108.5, 109.5, 118.8, 119.4, 120.1, 121.9, 125.3, 127.2, 127.9, 128.1, 128.4, 128.5, 128.6, 128.7, 132.1, 132.2, 132.3, 133.0, 136.9, 137.0, 141.4, 143.8, 143.9, 155.8, 168.5, 168.6, 168.7, 169.5, 170.3, 170.8, 171.6, 171.8, 172.06, 172.14, 172.4.

Selected diagnostic peaks:

Major rotamer: **^{1}H NMR** (500 MHz, $CDCl_3$) δ 0.01 – 0.04 (m, 6H), 0.88 (s, 9H), 2.80 (s, 3H), 2.96 (s, 3H), 3.32 (s, 3H), 3.68 (s, 3H), 5.62 (d, *J* = 8.3 Hz, 1H), 6.88 (s, 1H), 7.77 (d, *J* = 7.5 Hz, 2H). **^{13}C NMR** (126 MHz, $CDCl_3$) δ 26.1, 31.5, 81.5, 155.8.

Minor rotamer: **^{1}H NMR** (500 MHz, $CDCl_3$) δ -0.04 (s, 3H), -0.02 (s, 3H), 0.49 (d, *J* = 6.7 Hz, 3H), 0.84 (s, 9H), 2.74 (s, 3H), 3.66 (s, 3H), 5.70 (d, *J* = 8.5 Hz, 1H), 6.86 (s, 1H), 7.90 (d, *J* = 6.7 Hz, 1H). **^{13}C NMR** (126 MHz, $CDCl_3$) δ 26.0, 32.8, 79.2.

HRMS (ESI) calcd for $C_{71}H_{97}N_8O_{12}Si^+$ $(M+H)^+$: 1281.6990, found: 1281.7024.

(3*S*,6*S*,9*S*,12*S*,15*S*,18*S*,21*S*)-21-((*R*)-3-hydroxy-2-methylpropyl)-9-isobutyl-12-isopropyl-15-((*R*)-methoxy(phenyl)methyl)-1,10,18-trimethyl-3-((1-methyl-1*H*-indol-3-yl)methyl)-6-(prop-2-yn-1-yl)-1,4,7,10,13,16,19-heptaazacyclohenicosane-2,5,8,11,14,17,20-heptaone (10)

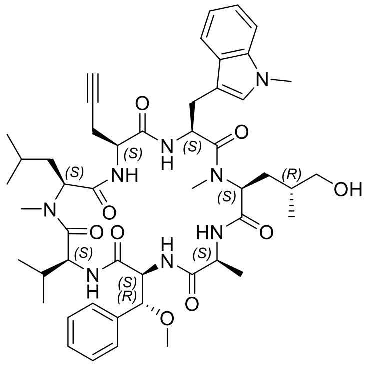

Prepared according to *GP6b*: **SI-18** (422 mg, 329 µmol), tris(2-aminoethyl)amine (0.49 ml, 3.29 mmol) (0.5 h); 1 M LiOH (658 µl, 658 µmol) (3 h); HATU (438 mg, 1.15 mmol), NMM (145 µl, 1.32 mmol) (addition over 1.5 h, additional 18 h); NH_4F (122 mg, 3.29 mmol). RP flash chromatography (MeCN/H_2O 10:90 – 95:5) followed by lyophilization yielded **10** (190 mg, 208 µmol, 63%) as a white amorphous solid.

$[\alpha]^{D}_{20}$ = –119.9 (c 0.5, $CHCl_3$).

^{1}H NMR (500 MHz, $CDCl_3$) δ -0.46 (dd, *J* = 12.2, 6.2 Hz, 1H), 0.29 (d, *J* = 6.8 Hz, 3H), 0.70 – 0.78 (m, 1H), 0.90 – 0.98 (m, 9H), 1.06 (d, *J* = 6.5 Hz, 3H), 1.06 – 1.14 (m, 1H), 1.26 (d, *J* = 7.2 Hz, 3H), 1.28 – 1.34 (m, 1H), 1.50 – 1.57 (m, 1H), 1.70 (d, *J* = 16.0 Hz, 1H), 1.89 (s, 1H), 2.19 – 2.31 (m, 1H), 2.33 (s, 1H), 2.51 – 2.58 (m, 1H), 2.59 (s, 3H), 2.92 (s, 3H), 2.94 – 2.99 (m, 1H), 3.00 – 3.07 (m, 1H), 3.20 (dd, *J* = 13.6, 4.8 Hz, 1H), 3.34 – 3.38 (m, 4H), 3.73 (s, 3H), 4.31 – 4.37 (m, 1H), 4.51 (t, *J* = 9.1 Hz, 1H), 4.70 – 4.81 (m, 3H), 4.87 – 4.96 (m, 2H), 5.06 (d, *J* = 5.4 Hz, 1H), 6.87 (s, 1H), 7.07 – 7.12 (m, 1H), 7.12 – 7.17 (m, 2H), 7.19 – 7.25 (m, 2H), 7.29 (dd, *J* = 10.3, 6.8 Hz, 5H), 7.48 (d, *J* = 8.0 Hz, 1H), 7.80 (bs, 1H), 8.09 (d, *J* = 8.6 Hz, 1H), 8.23 (d, *J* = 9.3 Hz, 1H), 8.47 (d, *J* = 10.2 Hz, 1H).

^{13}C NMR (126 MHz, $CDCl_3$) δ 17.4, 19.4, 20.0, 21.1, 22.3, 22.7, 23.7, 25.3, 28.7, 29.3, 29.4, 30.8, 31.8, 32.9, 33.3, 39.0, 50.8, 51.3, 51.9, 55.1, 56.2, 57.9, 59.0, 59.4, 65.9, 72.2, 80.1, 80.2, 107.7, 109.8, 118.7, 119.8, 122.4, 127.7, 127.99, 128.01, 128.6, 129.3, 135.1, 137.0, 168.6, 169.4, 170.2, 170.6, 170.7, 171.2, 171.8.

HRMS (ESI) calcd for $C_{49}H_{69}N_8O_9^+$ $(M+H)^+$: 913.5182; found: 913.5177.

(3*S*,3'*S*,6*S*,6'*S*,9*S*,9'*S*,12*S*,12'*S*,15*S*,15'*S*,18*S*,18'*S*,21*S*,21'*S*)-18,18'-((((ethane-1,2-diylbis(oxy))bis(ethane-2,1-diyl))bis(1*H*-1,2,3-triazole-1,4-diyl))bis(methylene))bis(12-((*R*)-3-hydroxy-2-methylpropyl)-21-isobutyl-3-isopropyl-6-((*R*)-methoxy(phenyl)methyl)-1,9,13-trimethyl-15-((1-methyl-1*H*-indol-3-yl)methyl)-1,4,7,10,13,16,19-heptaazacyclohenicosane-2,5,8,11,14,17,20-heptaone) (11)

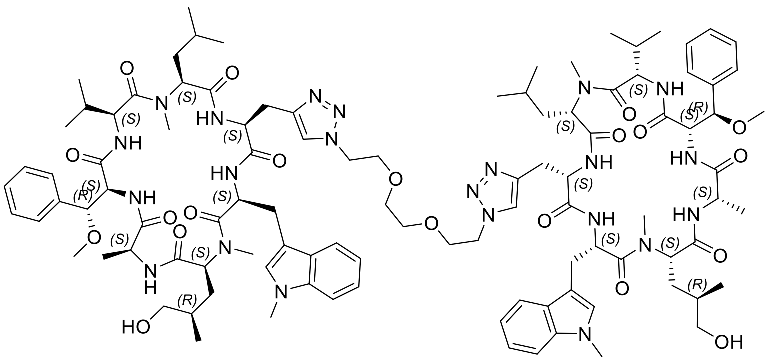

Prepared according to *GP7*: **10** (23.3 mg, 25.4 µmol), 1,2-bis(2-azidoethox)ethane (2.55 mg, 12.7 µmol), 1 M $CuSO_4$ (10.2 µl, 10.2 µmol), 1 M sodium ascorbate (28.1 µl, 28.1 µmol) (18 h). Flash chromatography (H_2O/MeCN 90:10 – 5:95) followed by prep HPLC (H_2O/MeCN 80:0 – 5:95) and lyophilization yielded **11** (16.4 mg, 8.09 µmol, 63%) as a white amorphous solid.

$[\alpha]_{20}^{D}$ = –118.2 (c 0.5, $CHCl_3$).

^{1}H NMR (500 MHz, $CDCl_3$) δ -0.72 (dd, *J* = 12.8, 6.4 Hz, 2H), 0.15 (d, *J* = 6.7 Hz, 6H), 0.56 – 0.62 (m, 2H), 0.90 (d, *J* = 6.7 Hz, 6H), 0.91 – 0.98 (m, 2H), 0.97 – 1.02 (m, 12H), 1.04 (d, *J* = 6.6 Hz, 6H), 1.12 (t, *J* = 8.4 Hz, 2H), 1.40 (d, *J* = 7.2 Hz, 8H), 1.50 (d, *J* = 11.2 Hz, 2H), 1.74 – 1.83 (m, 2H), 2.17 – 2.28 (m, 4H), 2.55 (s, 6H), 2.57 (s, 6H), 2.80 – 2.87 (m, 2H), 2.98 (dd, *J* = 11.3, 3.6 Hz, 2H), 3.24 – 3.29 (m, 4H), 3.31 (s, 6H), 3.67 (t, *J* = 6.6 Hz, 2H), 3.70 (s, 6H), 3.92 (dq, *J* = 7.5, 3.6 Hz, 2H), 4.12 (dd, *J* = 9.1, 4.9 Hz, 2H), 4.48 (d, *J* = 11.1 Hz, 2H), 4.50 – 4.62 (m, 4H), 4.68 – 4.75 (m, 2H), 4.78 (dt, *J* = 10.3, 5.4 Hz, 6H), 4.86 (dd, *J* = 9.9, 7.0 Hz, 2H), 4.98 (t, *J* = 5.0 Hz, 2H), 5.06 (d, *J* = 5.3 Hz, 2H), 6.78 (s, 2H), 7.03 (t, *J* = 7.6 Hz, 6H), 7.08 – 7.12 (m, 8H), 7.17 – 7.22 (m, 2H), 7.23 – 7.29 (m, 4H), 7.40 (s, 2H), 7.51 (d, *J* = 4.9 Hz, 2H), 7.57 (d, *J* = 7.9 Hz, 2H), 8.10 (d, *J* = 8.6 Hz, 2H), 8.43 (d, *J* = 9.3 Hz, 2H), 8.62 (d, *J* = 10.2 Hz, 2H), 8.99 (d, *J* = 5.4 Hz, 2H).

^{13}C NMR (126 MHz, $CDCl_3$) δ 17.4, 19.4, 20.0, 21.3, 22.8, 23.9, 24.8, 27.9, 28.9, 29.3, 29.4, 30.8, 31.9, 32.8, 33.1, 39.3, 50.4, 50.6, 51.2, 52.1, 54.9, 56.3, 57.9, 58.7, 59.3, 65.6, 69.5, 70.7, 80.1, 108.3, 109.8, 119.0, 119.5, 122.4, 123.7, 127.57, 127.64, 128.1, 128.6, 129.1, 135.3, 137.0, 141.6, 167.7, 169.9, 170.0, 170.6, 171.3, 171.4, 171.9.

HRMS (ESI) calcd for $C_{104}H_{149}N_{22}O_{20}^+$ $(M+H)^+$: 2026.1313; found: 2026.1266.

(3*S*,3'*S*,6*S*,6'*S*,9*S*,9'*S*,12*S*,12'*S*,15*S*,15'*S*,18*S*,18'*S*,21*S*,21'*S*)-18,18'-((pentane-1,5-diylbis(1*H*-1,2,3-triazole-1,4-diyl))bis(methylene))bis(12-((*R*)-3-hydroxy-2-methylpropyl)-21-isobutyl-3-isopropyl-6-((*R*)-methoxy(phenyl)methyl)-1,9,13-trimethyl-15-((1-methyl-1*H*-indol-3-yl)methyl)-1,4,7,10,13,16,19-heptaazacyclohenicosane-2,5,8,11,14,17,20-heptaone) (12) (UdSBI-4377)

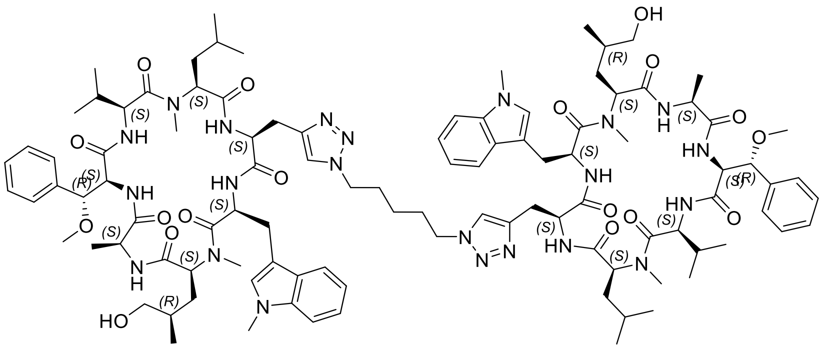

Prepared according to *GP7*: **10** (31.7 mg, 34.7 µmol), 1,5-diazidopentane (2.68 mg, 17.4 µmol), 1 M $CuSO_4$ (13.9 µl, 13.9 µmol), 1 M sodium ascorbate (22.6 µl, 22.6 µmol) (18 h). Flash chromatography (H_2O/MeCN 70:30 – 20:80) followed by prep HPLC (H_2O/MeCN 60:40 – 5:95) and lyophilization yielded **12** (24.7 mg, 11.8 µmol, 64%, purity 95%) as a white amorphous solid.

$[\alpha]_{20}^{D}$ = –98.3 (c 0.3, $CHCl_3$).

^{1}H NMR (500 MHz, $CDCl_3$) δ -0.51 (ddd, J = 13.8, 6.6, 2.5 Hz, 2H), 0.24 (dd, J = 6.8, 1.1 Hz, 6H), 0.77 (q, J = 5.8 Hz, 2H), 0.95 (d, J = 6.6 Hz, 8H), 1.03 – 1.08 (m, 18H), 1.26 – 1.31 (m, 8H), 1.50 – 1.60 (m, 4H), 1.59 – 1.70 (m, 2H),

1.85 (ddd, J = 13.4, 10.6, 7.3 Hz, 2H), 1.89 – 1.98 (m, 4H), 1.99 – 2.05 (m, 2H), 2.22 – 2.28 (m, 2H), 2.58 (s, 6H), 2.83 (s, 6H), 2.86 – 2.94 (m, 4H), 3.04 (dd, J = 11.2, 4.0 Hz, 2H), 3.09 – 3.15 (m, 2H), 3.22 – 3.27 (m, 2H), 3.30 (s, 6H), 3.71 (s, 6H), 4.36 – 4.47 (m, 4H), 4.55 (dd, J = 10.7, 2.6 Hz, 2H), 4.62 (t, J = 9.1 Hz, 2H), 4.73 – 4.78 (m, 2H), 4.79 – 4.86 (m, 4H), 4.90 – 4.97 (m, 4H), 5.10 (d, J = 5.4 Hz, 2H), 6.85 (d, J = 1.9 Hz, 2H), 6.96 – 7.00 (m, 4H), 7.07 – 7.09 (m, 6H), 7.18 – 7.21 (m, 2H), 7.23 – 7.28 (m, 4H), 7.34 – 7.38 (m, 2H), 7.45 – 7.52 (m, 4H), 8.15 (d, J = 8.8 Hz, 2H), 8.32 (d, J = 9.2 Hz, 2H), 8.75 (d, J = 10.0 Hz, 2H), 8.83 – 8.90 (m, 2H).

^{13}C NMR (126 MHz, $CDCl_3$) δ 17.4, 19.4, 20.0, 20.1, 21.1, 22.8, 22.9, 23.5, 23.7, 25.1, 28.0, 28.8, 28.9, 29.0, 29.5, 31.0, 32.0, 32.8, 33.2, 39.2, 50.2, 50.4, 51.0, 51.8, 54.9, 56.3, 57.9, 58.9, 59.5, 66.0, 80.0, 108.7, 109.7, 118.9, 119.5, 122.3, 127.7, 128.2, 128.4, 129.1, 135.5, 137.0, 142.1, 167.6, 169.7, 170.1, 170.8, 171.2, 171.5, 172.2.

HRMS (ESI) calcd for $C_{103}H_{147}N_{22}O_{18}^+$ (M+H)$^+$: 1980.1258; found: 1980.1278.

(3*S*,6*S*,9*S*,12*S*,15*S*,18*S*,21*S*)-21-((*R*)-3-hydroxy-2-methylpropyl)-9-isobutyl-12-isopropyl-15-((*R*)-methoxy(phenyl)methyl)-1,10,18-trimethyl-3-((1-methyl-1*H*-indol-3-yl)methyl)-6-((1-pentyl-1*H*-1,2,3-triazol-4-yl)methyl)-1,4,7,10,13,16,19-heptaazacyclohenicosane-2,5,8,11,14,17,20-heptaone (24)

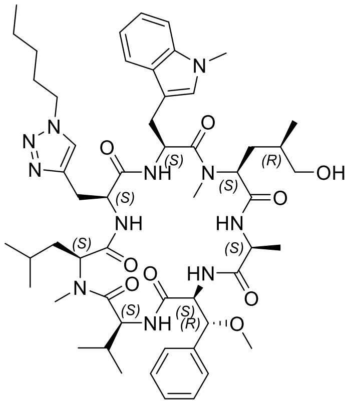

A 1.5 ml vial was charged with **10** (15.6 mg, 17.1 µmol), *t*-BuOH (248 µl) and H_2O (285 µl). To this solution, 1-azidopentane (37.2 µl of a 13.0 mg ml^{-1} stock solution, 4.83 mg, 42.7 µmol) followed by 1 M $CuSO_4$ (6.83 µl, 6.83 µmol) and 1 M sodium ascorbate (10.3 µl, 10.3 µmol) were added, the vial was flushed with Argon and sealed. After 5 h, 76% conversion was observed by LC/MS. The reaction mixture was subsequently evaporated in vacuo and the residue was purified by RP flash chromatography (H_2O/MeCN 90:10 – 5:95). After lyophilization, **24** (11.5 mg, 11.2 µmol, 66%) was obtained as a white amorphous solid.

$[\alpha]_{20}^{D}$ = –90.6 (c 0.5, $CHCl_3$).

^{1}H NMR (500 MHz, $CDCl_3$) δ -0.34 – -0.15 (m, 1H), 0.34 (d, *J* = 6.9 Hz, 3H), 0.86 – 0.91 (m, 1H), 0.93 (t, *J* = 7.2 Hz, 3H), 0.94 – 0.98 (m, 9H), 1.08 (d, *J* = 6.7 Hz, 3H), 1.17 (ddd, *J* = 13.0, 8.4, 4.2 Hz, 1H), 1.27 – 1.34 (m, 5H), 1.36 – 1.43 (m, 2H), 1.47 – 1.58 (m, 1H), 1.62 (dd, *J* = 14.6, 10.9 Hz, 1H), 1.90 – 1.98 (m, 2H), 2.17 – 2.30 (m, 3H), 2.48 – 2.56 (m, 1H), 2.63 (s, 3H), 2.86 (s, 3H), 2.95 – 3.00 (m, 1H), 3.02 – 3.06 (m, 1H), 3.07 – 3.13 (m, 1H), 3.19 – 3.28 (m, 1H), 3.35 (s, 3H), 3.71 (s, 3H), 4.36 (t, *J* = 7.0 Hz, 2H), 4.45 – 4.59 (m, 3H), 4.67 – 4.77 (m, 2H), 4.77 – 4.86 (m, 1H), 4.93 (t, *J* = 5.0 Hz, 1H), 5.07 (d, *J* = 5.3 Hz, 1H), 6.82 (s, 1H), 6.86 (d, *J* = 5.0 Hz, 1H), 7.04 – 7.17 (m, 7H), 7.17 – 7.24 (m, 2H), 7.24 – 7.30 (m, 1H), 7.37 (d, *J* = 4.9 Hz, 1H), 7.45 (d, *J* = 7.9 Hz, 1H), 8.08 (d, *J* = 8.5 Hz, 1H), 8.14 (d, *J* = 9.3 Hz, 1H), 8.59 (d, *J* = 9.9 Hz, 1H).

^{13}C NMR (126 MHz, $CDCl_3$) δ 14.1, 17.4, 19.4, 20.0, 20.8, 22.3, 22.7, 23.6, 25.2, 28.2, 28.7, 28.8, 29.2, 29.6, 29.9, 31.0, 32.3, 32.8, 33.3, 38.9, 50.4, 50.6, 51.1, 52.4, 54.9, 56.3, 57.9, 59.0, 59.5, 66.3, 80.1, 107.9, 109.7, 118.7, 119.6, 121.8, 122.4, 127.6, 127.9, 128.1, 128.4, 129.2, 135.4, 137.0, 141.9, 168.1, 169.87, 169.89, 170.9, 171.1, 171.2, 172.1.

HRMS (ESI) calcd for $C_{54}H_{80}N_{11}O_9^+$ (M+H)$^+$: 1026.6135; found: 1026.6085.

Synthesis of enantiomeric exit vector 7 Homo-BacPROTAC (12a)

N^{α}-((allyloxy)carbonyl)-1-methyl-D-tryptophan (SI-19)

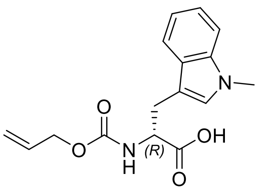

((Allyloxy)carbonyl)-D-tryptophan (**SI-16**) (500 mg, 1.73 mmol) was dissolved in DMF (5.8 ml) and KO*t*-Bu (448 mg, 3.99 mmol) was added at rt and stirred until all solids were dissovled. The mixture was cooled to 0 °C and methyl iodide (152 µl, 2.43 mmol) was added in one portion. TLC control after 10 min indicated full conversion, the reaction was quenched by addition of 1 M HCl after 20 min. The mixture was extracted with EtOAc (3x). The combined organic phases were washed with 1 M LiCl (2x), brine and dried (Na_2SO_4). Flash chromatography (CyH/[EtOAc+2%HOAc] 100:0 – 70:30 – 50:50) yielded **SI-19** (283 mg, 936 µmol, 54%) as a brownish resin. R_f = 0.31 (PE/EtOAc/HOAc 40:60:1).

$[\alpha]_{20}^{D}$ = –40.6 (c 0.5, $CHCl_3$).

Major rotamer:

^{1}H NMR (500 MHz, DMSO-d_6) δ 2.98 (dd, *J* = 14.6, 9.7 Hz, 1H), 3.16 (dd, *J* = 14.6, 4.6 Hz, 1H), 3.72 (s, 3H), 4.19 (ddd, *J* = 9.7, 8.1, 4.6 Hz, 1H), 4.36 – 4.46 (m, 2H), 5.14 (dd, *J* = 10.5, 1.6 Hz, 1H), 5.23 (dd, *J* = 17.2, 1.7 Hz, 1H), 5.85 (ddt, *J* = 17.2, 10.5, 5.3 Hz, 1H), 7.02 (ddd, *J* = 7.9, 7.0, 1.0 Hz, 1H), 7.09 – 7.18 (m, 2H), 7.38 (d, *J* = 8.2 Hz, 1H), 7.55 (d, *J* = 8.0 Hz, 2H), 12.71 (bs, 1H). **^{13}C NMR** (126 MHz, DMSO) δ 26.8, 32.3, 55.0, 64.4, 109.5, 109.7, 117.0, 118.4, 118.6, 121.1, 127.5, 128.1, 133.6, 136.6, 155.9, 173.8.

Minor rotamer (selected signals, ratio ~8:1):

^{1}H NMR (500 MHz, DMSO-d_6) δ 4.30 – 4.38 (m, 2H), 5.04 – 5.11 (m, 2H), 5.75 (ddt, *J* = 15.7, 10.0, 4.8 Hz, 1H).

HRMS (ESI) calcd for $C_{16}H_{19}N_2O_4^+$ $(M+H)^+$: 303.1339; found: 303.1348.

methyl *N*-(((2*R*,3*S*)-2-((*R*)-2-((2*R*,4*S*)-2-((*R*)-2-(((allyloxy)carbonyl)amino)-*N*-methyl-3-(1-methyl-1*H*-indol-3-yl)propanamido)-5-((*tert*-butyldimethylsilyl)oxy)-4-methylpentanamido)propanamido)-3-methoxy-3-phenylpropanoyl)-D-valyl)-*N*-methyl-D-leucinate (SI-20)

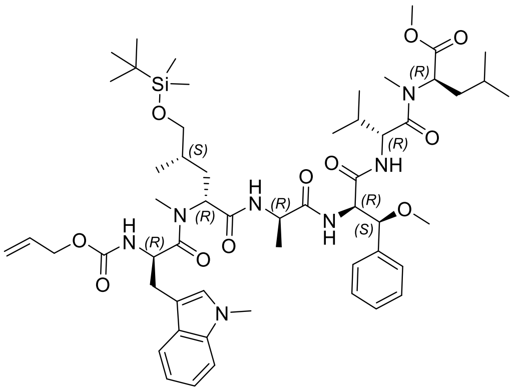

Prepared according to *GP1* and *GP5*: **2a** (522 mg, 581 µmol), Pd/C (49.5 mg) (2 h); **SI-19** (243 mg, 802 µmol), DIPEA (140 µl, 802 µmol), $BnNMe_2$ (8.51 µl, 57.3 µmol), 1 M isopropyl chloroformate (802 µl, 802 µmol), NMI (4.57 µl, 57.2 µmol) and HCl (14.3 µl, 57.3 µmol) (2.5 h). Flash chromatography (CyH/EtOAc 100:0 – 25:75) followed by lyophilization afforded **SI-20** (547 mg, 522 µmol, 91%) as a white amorphous solid. R_f = 0.28 (PE/EtOAc 3:7).

$[\alpha]_{20}^{D}$ = +59.9 (c 0.5, $CHCl_3$).

^{1}H NMR (500 MHz, $CDCl_3$) *(mixture of rotamers, ratio ~3:2)* δ -0.19 – -0.11 (m, 0.3H), -0.04 (s, 0.9H), -0.02 (s, 0.9H), 0.00 – 0.05 (m, 5.0H), 0.45 (d, *J* = 6.7 Hz, 0.8H), 0.83 (s, 2.8H), 0.87 (s, 7.2H), 0.89 – 0.93 (m, 6.8H), 0.93 – 0.98 (m,, 7.1H), 0.99 (d, *J* = 6.8 Hz, 2.5H), 1.18 (d, *J* = 7.1 Hz, 1.8H), 1.22 – 1.27 (m, 1.5H), 1.43 – 1.53 (m, 2.5H), 1.66 – 1.80 (m, 2.2H), 1.87 – 2.05 (m, 2.0H), 2.06 – 2.22 (m, 1.2H), 2.72 – 2.76 (m, 2.5H), 2.85 – 2.93 (m, 0.6H), 2.95 – 3.02 (m, 3.3H), 3.07 (dd, *J* = 9.5, 5.5 Hz, 0.3H), 3.13 (dd, *J* = 14.2, 5.6 Hz, 1.0H), 3.21 (dd, *J* = 14.5, 8.0 Hz,

0.7H), 3.28 (s, 1.2H), 3.33 (s, 2.5H), 3.40 (dd, J = 9.9, 5.4 Hz, 0.7H), 3.45 (dd, J = 9.9, 4.5 Hz, 0.7H), 3.67 – 3.70 (m, 5.0H), 4.13 – 4.23 (m, 0.6H), 4.23 – 4.31 (m, 0.4H), 4.42 – 4.57 (m, 2.0H), 4.59 – 4.66 (m, 0.8H), 4.66 – 4.73 (m, 0.9H), 4.76 – 4.91 (m, 3.2H), 4.93 – 5.00 (m, 0.6H), 5.13 – 5.32 (m, 2.2H), 5.36 (dd, J = 10.5, 5.2 Hz, 1.0H), 5.62 (d, J = 7.4 Hz, 0.2H), 5.80 – 5.92 (m, 1.4H), 6.36 (d, J = 6.9 Hz, 0.5H), 6.76 (d, J = 7.5 Hz, 0.3H), 6.80 (d, J = 7.8 Hz, 0.5H), 6.86 – 6.91 (m, 0.9H), 7.06 – 7.14 (m, 1.2H), 7.16 – 7.25 (m, 4.5H), 7.25 – 7.32 (m, 4.0H), 7.35 (d, J = 8.8 Hz, 0.4H), 7.45 (d, J = 8.7 Hz, 0.5H), 7.54 – 7.58 (m, 0.3H), 7.65 (d, J = 7.9 Hz, 0.6H), 7.89 (d, J = 6.7 Hz, 0.2H).

^{13}C NMR (126 MHz, $CDCl_3$) *(mixture of rotamers)* δ -5.30, -5.27, -5.25, 15.7, 17.4, 17.5, 17.6, 17.8, 18.4, 18.5, 19.6, 19.7, 21.5, 23.41, 23.44, 24.9, 26.0, 26.1, 28.6, 28.8, 29.3, 31.3, 31.4, 31.5, 32.2, 32.8, 37.0, 49.8, 50.0, 51.0, 51.7, 52.2, 52.3, 54.2, 54.4, 54.5, 54.6, 56.4, 57.6, 57.7, 57.9, 58.0, 65.8, 66.4, 67.3, 68.4, 81.3, 81.5, 108.4, 108.6, 109.5, 117.7, 118.4, 118.7, 118.8, 119.4, 119.6, 121.9, 122.2, 127.0, 127.8, 127.8, 128.0, 128.1, 128.3, 128.4, 128.5, 132.2, 132.8, 136.9, 136.9, 137.0, 156.1, 156.8, 168.6, 168.9, 170.5, 171.7, 171.7, 172.1, 172.2, 172.3, 172.6, 173.4.

Selected diagnostic peaks:

Major rotamer: **^{1}H NMR** (500 MHz, $CDCl_3$) δ 0.02 (s, 3H), 0.03 (s, 3H), 0.87 (s, 9H), 3.13 (dd, J = 14.2, 5.6 Hz, 1H), 3.21 (dd, J = 14.5, 8.0 Hz, 1H), 3.33 (s, 3H), 3.69 (s, 3H), 4.17 (t, J = 7.1 Hz, 1H), 4.70 (dd, J = 7.7, 3.6 Hz, 1H), 5.36 (dd, J = 10.5, 5.2 Hz, 1H), 6.80 (d, J = 7.8 Hz, 1H), 6.89 (s, 1H), 7.65 (d, J = 7.9 Hz, 1H). **^{13}C NMR** (126 MHz, $CDCl_3$) δ 26.1, 52.3, 81.5, 156.1.

Minor rotamer: **^{1}H NMR** (500 MHz, $CDCl_3$) δ -0.19 – -0.11 (m, 1H), -0.04 (s, 3H), -0.02 (s, 3H), 0.45 (d, J = 6.7 Hz, 3H), 0.83 (s, 9H), 2.97 (s, 3H), 3.28 (s, 3H), 3.68 (s, 4H), 4.25 – 4.30 (m, 1H), 5.62 (d, J = 7.4 Hz, 1H), 6.76 (d, J = 7.5 Hz, 1H), 6.89 (s, 1H), 7.87 (d, J = 6.7 Hz, 1H).). **^{13}C NMR** (126 MHz, $CDCl_3$) δ 26.0, 52.2, 81.3, 156.8.

HRMS (ESI) calcd for $C_{55}H_{86}N_7O_{11}Si^+$ $(M+H)^+$: 1048.6149; found: 1048.6149.

methyl *N*-(((2*R*,3*S*)-2-((*R*)-2-((2*R*,4*S*)-2-((*R*)-2-((*R*)-2-((((9*H*-fluoren-9-yl)methoxy)carbonyl)amino)pent-4-ynamido)-*N*-methyl-3-(1-methyl-1*H*-indol-3-yl)propanamido)-5-((*tert*-butyldimethylsilyl)oxy)-4-methylpentanamido)propanamido)-3-methoxy-3-phenylpropanoyl)-D-valyl)-*N*-methyl-D-leucinate (SI-18a)

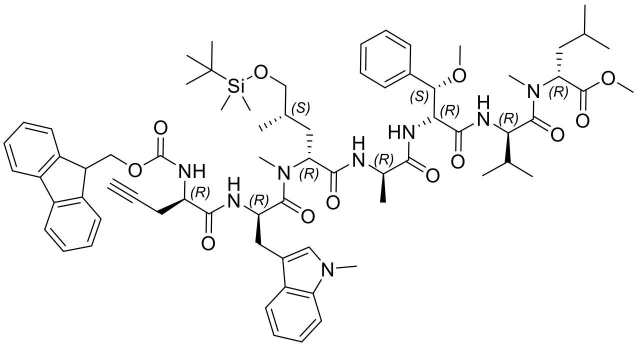

Prepared according to *GP2* and *GP4*: **SI-20** (511 mg, 487 µmol), DMBA (228 mg, 1.46 mmol), $Pd(PPh_3)_4$ (16.9 mg, 14.6 µmol) (1 h); Fmoc-D-Pra-OH (212 mg, 633 µmol), NMM (134 µl, 1.22 mmol), HOBt (104 mg, 682 µmol), EDC (131 mg, 682 µmol) (16 h). Flash chromatography (CyH/EtOAc 100:0 – 20:80) followed by lyophilization yielded **SI-18a** (403 mg, 310 µmol, 64%, 99% purity) as a yellow foam. R_f = 0.23 (PE/EtOAc 3:7).

$[\alpha]_{20}^{D}$ = +49.4 (c 0.5, $CHCl_3$).

^{1}H NMR ($CDCl_3$, 500 MHz) *(mixture of rotamers, ratio ~2:1)* δ -0.03 (s, 0.6H), -0.01 (s, 0.6H), 0.01 – 0.07 (m, 4.7H), 0.52 (d, J = 6.7 Hz, 0.5H), 0.85 (s, 2.0H), 0.88 (s, 6.6H), 0.89 – 0.97 (m, 11.2H), 0.99 (d, J = 6.8 Hz, 2.7H), 1.21 – 1.30 (m, 3.7H), 1.33 (d, J = 7.1 Hz, 0.5H), 1.43 – 1.53 (m, 2.2H), 1.62 – 1.81 (m, 2.3H), 1.93 – 2.00 (m, 0.7H), 2.02 (t, J = 2.3 Hz, 0.8H), 2.08 – 2.18 (m, 1.3H), 2.21 – 2.33 (m, 1.0H), 2.53 – 2.69 (m, 1.5H), 2.74 (s, 0.6H), 2.80 (s, 1.7H), 2.94 – 2.98 (m, 2.3H), 3.00 (s, 0.7H), 3.10 – 3.18 (m, 0.9H), 3.28 (m, 0.9H), 3.31 – 3.35 (m, 2.6H), 3.39 – 3.45 (m, 1.3H), 3.65 – 3.71 (m, 5.2H), 4.19 – 4.25 (m, 0.9H), 4.27 (t, J = 7.1 Hz, 1H), 4.31 – 4.47 (m, 2.7H), 4.66 – 4.72 (m, 1.1H), 4.74 – 4.84 (m, 1.7H), 4.85 (d, J = 4.3 Hz, 0.7H), 5.00 – 5.07 (m, 0.2H), 5.21 – 5.29 (m, 0.6H), 5.29 – 5.38 (m, 0.9H), 5.52 – 5.60 (m, 0.4H), 5.63 (d, J = 8.4 Hz, 0.2H), 6.38 (d, J = 7.1 Hz, 0.5H), 6.75 (d, J = 7.3 Hz, 0.2H), 6.86 – 6.90 (m, 1.0H), 7.06 – 7.13 (m, 1.0H), 7.16 – 7.22 (m, 2.0H), 7.22 – 7.26 (m, 2.9H), 7.27 – 7.34 (m, 4.5H), 7.36 – 7.42 (m,

1.8H), 7.43 – 7.49 (m, 1.1H), 7.51 – 7.56 (m, 0.8H), 7.56 – 7.63 (m, 1.7H), 7.65 – 7.71 (m, 1.6H), 7.74 – 7.78 (m, 1.7H).

^{13}C NMR (126 MHz, $CDCl_3$) *(mixture of rotamers)* δ -5.3, 14.3, 16.0, 17.4, 17.5, 18.0, 18.5, 19.6, 21.5, 21.6, 23.1, 23.4, 24.9, 26.05, 26.10, 28.6, 31.4, 31.5, 32.3, 32.8, 37.0, 47.2, 49.6, 50.5, 52.3, 53.1, 54.2, 54.6, 57.5, 57.7, 58.3, 60.5, 67.3, 67.4, 72.0, 79.2, 81.5, 108.5, 109.5, 118.8, 119.4, 120.1, 121.9, 125.3, 127.2, 127.9, 128.1, 128.4, 128.5, 128.6, 128.7, 132.1, 132.2, 132.3, 133.0, 136.9, 137.0, 141.4, 143.9, 144.0, 155.8, 168.5, 168.6, 169.5, 170.3, 171.8, 172.1, 172.1, 172.4.

Selected diagnostic peaks:

Major rotamer: **^{1}H NMR** (500 MHz, $CDCl_3$) δ 0.03 (s, 3H), 0.03 (s, 3H), 0.88 (s, 9H), 2.01 – 2.03 (m, 1H), 2.80 (s, 3H), 2.97 (s, 3H), 3.33 (s, 3H), 3.68 (s, 3H), 4.22 (t, *J* = 7.1 Hz, 1H), 4.70 (dd, *J* = 7.6, 4.4 Hz, 1H), 5.56 (bs, 1H), 6.89 (s, 1H). **^{13}C NMR** (126 MHz, $CDCl_3$) δ 26.05, 81.5, 155.8.

Minor rotamer: **^{1}H NMR** (500 MHz, $CDCl_3$) δ -0.03 (s, 3H), -0.01 (s, 3H), 0.52 (d, *J* = 6.7 Hz, 3H), 1.33 (d, *J* = 7.1 Hz, 3H), 1.93 – 1.95 (m, 1H), 2.74 (s, 3H), 2.95 (s, 3H), 3.28 (s, 3H), 3.66 (s, 3H), 4.27 (t, *J* = 7.1 Hz, 1H), 5.63 (d, *J* = 8.4 Hz, 1H), 6.75 (d, *J* = 7.3 Hz, 1H), 6.87 (s, 1H). **^{13}C NMR** (126 MHz, $CDCl_3$) δ 26.05, 79.2.

HRMS (ESI) calcd for $C_{71}H_{97}N_8O_{12}Si^+$ $(M+H)^+$: 1281.6990; found: 1281.6965.

(3*R*,6*R*,9*R*,12*R*,15*R*,18*R*,21*R*)-21-((*S*)-3-hydroxy-2-methylpropyl)-9-isobutyl-12-isopropyl-15-((*S*)-methoxy(phenyl)methyl)-1,10,18-trimethyl-3-((1-methyl-1*H*-indol-3-yl)methyl)-6-(prop-2-yn-1-yl)-1,4,7,10,13,16,19-heptaazacyclohenicosane-2,5,8,11,14,17,20-heptaone (10a)

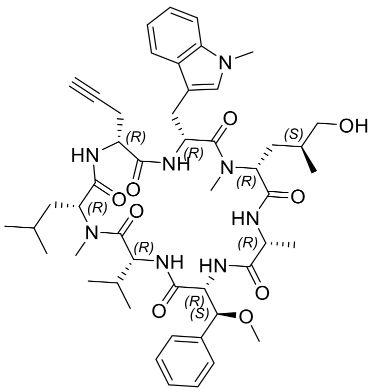

Prepared according to modified *GP6b*: **SI-18a** (200 mg, 156 µmol), tris(2-aminoethyl)amine (234 µl, 1.56 mmol) (2 h); 1 M LiOH (328 µl, 328 µmol) (3 h); PyAOP (285 mg, 546 µmol), DIPEA (123 µl, 702 µmol) (addition over 4 h, additional 18 h); Deprotection: Crude cyclic peptide was dissolved in THF (3.0 ml)/H_2O (0.15 ml), pTosOH (35.6 mg, 187 µmol) was added at 0 °C. After 16 h, the mixture was quenched with sat. $NaHCO_3$ soln., extracted with EtOAc (3x). The combined org phases were dried (Na_2SO_4) and evaporated. RP flash chromtography (H_2O/MeCN 90:10 – 5:95) followed by prep HPLC (H_2O/MeCN 80:20 – 5:95) and lyophilization afforded **10a** (65.4 mg, 71.6 µmol, 46%) as a white amorphous solid.

$[\alpha]_{20}^{D}$ = +105.8 (c 0.5, $CHCl_3$).

^{1}H NMR (500 MHz, $CDCl_3$) δ -0.54 – -0.45 (m, 1H), 0.28 (d, *J* = 6.7 Hz, 3H), 0.70 – 0.77 (m, 1H), 0.92 (d, *J* = 6.7 Hz, 3H), 0.94 – 0.98 (m, 6H), 1.05 (d, *J* = 6.7 Hz, 3H), 1.07 – 1.12 (m, 1H), 1.25 (d, *J* = 7.3 Hz, 3H), 1.28 – 1.38 (m, 2H), 1.50 – 1.58 (m, 1H), 1.70 – 1.78 (m, 1H), 1.84 – 1.91 (m, 1H), 2.20 – 2.31 (m, 2H), 2.36 – 2.39 (m, 1H), 2.58 (s, 3H), 2.90 – 2.98 (m, 4H), 2.99 – 3.06 (m, 1H), 3.21 (dd, *J* = 13.6, 4.9 Hz, 1H), 3.32 – 3.37 (m, 4H), 3.73 (s, 3H), 4.33 (dd, *J* = 10.5, 2.8 Hz, 1H), 4.51 (t, *J* = 9.2 Hz, 1H), 4.71 – 4.80 (m, 3H), 4.91 – 4.99 (m, 2H), 5.06 (d, *J* = 5.3 Hz, 1H), 6.87 (s, 1H), 7.10 (ddd, *J* = 8.1, 6.9, 1.1 Hz, 1H), 7.12 – 7.18 (m, 2H), 7.18 – 7.25 (m, 1H), 7.26 – 7.33 (m, 5H), 7.48 (d, *J* = 7.9 Hz, 1H), 7.88 (d, *J* = 11.0 Hz, 1H), 8.09 (d, *J* = 8.5 Hz, 1H), 8.23 – 8.29 (m, 1H), 8.48 (d, *J* = 10.2 Hz, 1H).

^{13}C NMR (126 MHz, $CDCl_3$) δ 17.3, 19.4, 20.0, 21.1, 22.3, 22.7, 23.7, 25.4, 28.7, 29.2, 29.4, 30.8, 31.8, 32.9, 33.3, 39.0, 50.8, 51.3, 52.0, 55.1, 56.2, 57.9, 59.0, 59.4, 65.9, 72.3, 80.2, 107.7, 109.8, 118.7, 119.8, 122.4, 127.7, 128.0, 128.4, 128.6, 129.3, 135.1, 137.0, 168.6, 169.4, 170.2, 170.5, 170.7, 171.2, 171.8.

HRMS (ESI) calcd for $C_{49}H_{69}N_8O_9$ $(M+H)^+$: 913.5182; found: 913.5141.

(3*R*,3'*R*,6*R*,6'*R*,9*R*,9'*R*,12*R*,12'*R*,15*R*,15'*R*,18*R*,18'*R*,21*R*,21'*R*)-18,18'-((pentane-1,5-diylbis(1*H*-1,2,3-triazole-1,4-diyl))bis(methylene))bis(12-((*S*)-3-hydroxy-2-methylpropyl)-21-isobutyl-3-isopropyl-6-((*S*)-methoxy(phenyl)methyl)-1,9,13-trimethyl-15-((1-methyl-1*H*-indol-3-yl)methyl)-1,4,7,10,13,16,19-heptaazacyclohenicosane-2,5,8,11,14,17,20-heptaone) (12a) (UdSBI-0117)

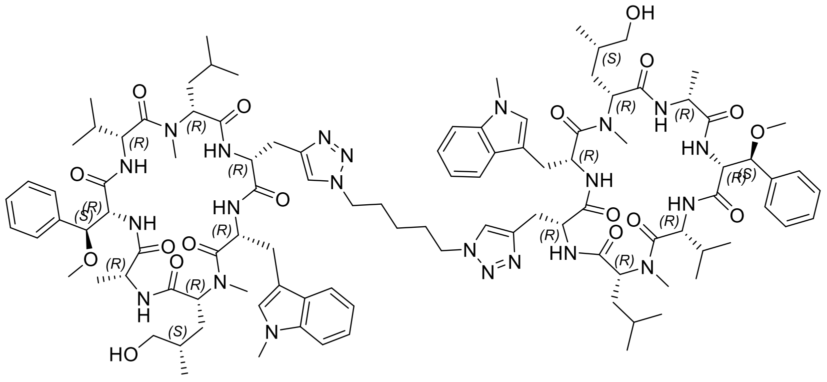

Prepared according to *GP7*: **10a** (20.6 mg, 22.6 µmol), 1,5-diazidopentane (1.74 mg, 11.3 µmol), 1 M $CuSO_4$ (9.02 µl, 9.02 µmol), 1 M sodium ascorbate (15.8 µl, 15.8 µmol) (16 h). RP flash chromatography (H_2O/MeCN 90:10 – 5:95) followed by prep HPLC (H_2O/MeCN 75:25 – 5:95) and lyophilization afforded **12a** (15.7 mg, 6.34 µmol, 56%, 80% purity) as a white amorphous solid.

$[\alpha]_{20}^{D}$ = +99.1 (c 0.5, $CHCl_3$).

^{1}H NMR (500 MHz, $CDCl_3$) δ -0.40 – -0.32 (m, 2H), 0.28 (d, *J* = 6.8 Hz, 6H), 0.87 – 0.93 (m, 2H), 0.96 – 0.98 (m, 8H), 1.04 – 1.10 (m, 18H), 1.28 – 1.32 (m, 8H), 1.51 – 1.64 (m, 4H), 1.88 – 1.99 (m, 4H), 2.00 – 2.09 (m, 2H), 2.09 – 2.18 (m, 2H), 2.19 – 2.29 (m, 4H), 2.61 (s, 6H), 2.68 – 2.74 (m, 2H), 2.86 (s, 6H), 2.91 – 2.98 (m, 2H), 3.03 – 3.08 (m, 2H), 3.08 – 3.13 (m, 2H), 3.26 – 3.29 (m, 2H), 3.32 (s, 6H), 3.71 (s, 6H), 4.36 – 4.46 (m, 4H), 4.59 – 4.66 (m, 4H), 4.73 – 4.78 (m, 2H), 4.79 – 4.87 (m, 4H), 4.89 – 4.94 (m, 2H), 4.94 – 5.02 (m, 2H), 5.13 (d, *J* = 5.4 Hz, 2H), 6.86 (s, 2H), 6.91 – 7.03 (m, 6H), 7.05 – 7.12 (m, 8H), 7.17 – 7.22 (m, 2H), 7.33 (s, 2H), 7.44 – 7.51 (m, 4H), 8.12 (d, *J* = 8.7 Hz, 2H), 8.24 (d, *J* = 9.3 Hz, 2H), 8.62 (d, *J* = 5.4 Hz, 2H), 8.67 (d, *J* = 9.9 Hz, 2H).

^{13}C NMR (126 MHz, $CDCl_3$) δ 17.3, 19.4, 20.0, 21.1, 22.9, 23.5, 23.7, 25.1, 28.0, 28.8, 28.97, 29.02, 29.5, 31.0, 32.1, 32.8, 33.2, 39.1, 50.1, 51.0, 51.1, 51.9, 54.9, 56.3, 57.9, 58.9, 59.5, 66.0, 80.0, 108.7, 109.7, 118.8, 119.5, 122.3, 122.8, 127.7, 128.2, 128.35, 128.44, 129.1, 135.5, 137.0, 142.2, 167.6, 169.7, 170.1, 170.8, 171.3, 171.5, 172.3.

HRMS (ESI) calcd for $C_{103}H_{147}N_{22}O_{18}$ $(M+H)^+$: 1980.1258; found: 1980.1195.

Synthesis of exit vector 3 triazole-based Homo-BacPROTACs (SI-39, SI-40)

(2*S*,4*R*)-5-[(*tert*-butyldimethylsilyl)oxy]-2-({[(9H-fluoren-9-yl)methoxy]carbonyl}(methyl)amino)-4-methylpentanoic acid (SI-21)

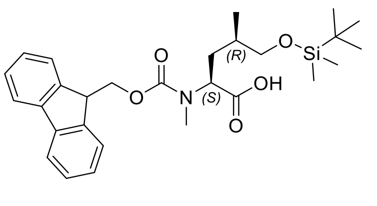

The Cbz-amino acid was deprotected according to modified *GP1*: (2*S*,4*R*)-2-(((benzyloxy)carbonyl)(methyl)amino)-5-((tert-butyldimethylsilyl)oxy)-4-methylpentanoic acid (5.00 g, 12.2 mmol), Pd/C (8.80 g), toluene (125 ml, 0.1 M) (6 h); The resulting free amino acid (4.00 g, 14.5 mmol) was dissolved in a mixture of 1,4-dioxane (15.0 ml) and water (5.0 ml) (0.73 M) and cooled to 0°C. Then sodium bicarbonate (1.46 g, 17.4 mmol) and Fmoc-Cl (3.94 g, 15.2 mmol) were added and the mixture was stirred at 0 °C for 10 minutes before it was allowed to reach rt. After 16 h, TLC indicated full conversion. The reaction mixture was diluted with EtOAc and water, the layers were separated and the aqueous layer was extracted with EtOAc (3x). The combined organic layers were washed with brine, dried ($MgSO_4$) and concentrated in vacuo. Reverse phase column chromatography (40-100% MeCN in water) yielded **SI-21** (3.50 g, 7.03 mmol, 48%) as a colorless gum, which was stored at 4 °C.

$[\alpha]_{20}^{D}$ = −12.1 (c 1.0, $CHCl_3$).

Major rotamer: **^{1}H NMR** (500 MHz, DMSO-d_6) δ -0.01 (s, 6H), 0.81 – 0.89 (m, 9H), 0.88 (d, *J* = 6.8 Hz, 3H), 1.36 – 1.46 (m, 1H), 1.46 – 1.56 (m, 1H), 1.89 (ddd, *J* = 14.1, 9.1, 4.7 Hz, 1H), 2.71 (s, 3H), 3.42 – 3.46 (m, 1H), 4.26 – 4.41 (m, 2H), 4.61 (dd, *J* = 11.1, 4.7 Hz, 1H), 7.27 – 7.36 (m, 2H), 7.39 – 7.43 (m, 2H), 7.58 – 7.67 (m, 2H), 7.86 – 7.92 (m, 2H).

^{13}C NMR (126 MHz, DMSO-d_6) δ -5.5, -5.4, 17.6, 18.0, 25.8, 25.9, 30.0, 31.9, 46.7, 46.7, 56.1, 65.7, 66.8, 120.2, 125.0, 127.1, 127.7, 140.8, 143.7, 143.9, 156.1, 172.9.

Minor rotamer (selected signals): **^{1}H NMR** (500 MHz, DMSO-d_6) δ 0.00 (s, 6H), 2.76 (s, 3H), 4.19 – 4.24 (m, 1H), 4.53 (dd, *J* = 9.9, 5.1 Hz, 1H). **^{13}C NMR** (126 MHz, DMSO-d_6) δ 17.2, 31.1, 56.4, 66.1, 155.7.

HRMS (ESI) calcd for $C_{28}H_{40}NO_5Si^+$ $(M+H)^+$: 498.2670; found: 498.2650.

***tert*-butyl *N*-[(1*R*,2*R*)-1,3-dihydroxy-1-(4-nitrophenyl)propan-2-yl]carbamate (SI-22)**

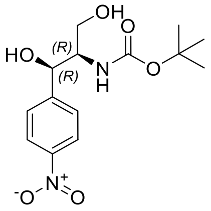

To a stirred solution of (1*R*,2*R*)-2-Amino-1-(4-nitro-phenyl)-propane-1,3-diol (75.0 g, 353 mmol) in MeOH (200 ml, 1.8 M) was added Boc-anhydride (89.3 ml, 389 mmol) slowly at 0° C. The mixture was stirred at 0-5 °C. After 4 h, TLC indicated complete conversion. The solvent was evaporated in vacuo, the crude was dried in high vacuum to yield **SI-22** (110 g, 352 mmol, quant.) which was used in the next step without further purification.

^{1}H NMR (400 MHz, DMSO-d_6) δ 1.05 (br s, 1.35H, rotamer), 1.21 (s, 7.65H, rotamer), 3.32 – 3.26 (m, 1H), 3.53 (dt, *J* = 10.5, 6.7 Hz, 1H), 3.73 – 3.61 (m, 1H), 4.76 (br t, *J* = 5.2 Hz, 1H), 4.94 (br s, 1H), 5.58 (br s, 1H), 5.76 (br d, *J* = 9.4 Hz, 0.15H, rotamer), 6.15 (d, *J* = 9.4 Hz, 0.85H, rotamer), 7.56 (d, *J* = 8.6 Hz, 2H), 8.17 (d, *J* = 8.6 Hz, 2H).

^{13}C NMR (101 MHz, DMSO-d_6) δ 27.6, 28.0, 57.5, 58.8, 60.7, 61.0, 69.9, 77.6, 122.8, 127.4, 146.3, 152.0, 152.4, 155.1.

MS calcd for $C_{10}H_{13}N_2O_6$ $(M - C_4H_9 + 2H)^+$: 257.08; found: 257.03.

The spectroscopic data are in agreement with previously published results.[15]

***tert*-butyl *N*-[(1*R*,2*R*)-3-[(*tert*-butyldimethylsilyl)oxy]-1-hydroxy-1-(4-nitrophenyl)propan-2-yl]carbamate (SI-23)**

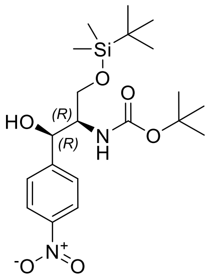

To a stirred solution of crude *tert*-butyl ((1*R*,2*R*)-1,3-dihydroxy-1-(4-nitrophenyl)propan-2-yl)carbamate (100 g, 320 mmol) in DMF (120 ml, 2.7 M) were added imidazole (54.5 g, 800 mmol) and TBDMS-Cl (106 g, 704 mmol) at 0°C. The mixture was stirred at rt. After 4 h, TLC indicated complete conversion. The mixture was diluted with EtOAc and water, the aqueous layer was extracted with EtOAc (3x). The combined organic layers were washed with brine, dried (Na_2SO_4) and concentrated in vacuo. Silica gel column chromatography (20% EtOAc in petroleum ether) yielded **SI-23** (107 g, 251 mmol, 78%).

^{1}H NMR (400 MHz, DMSO-d$_6$) δ 0.05 (s, 6H), 0.87 (s, 9H), 1.05 (br s, 1.8H, rotamer), 1.22 (s, 7.2H, rotamer), 3.57 – 3.40 (m, 1H), 3.81 – 3.59 (m, 2H), 4.90 (dd, *J* = 5.2, 2.2 Hz, 1H), 5.63 (d, *J* = 5.3 Hz, 1H), 5.88 (br d, *J* = 9.4 Hz, 0.2H, rotamer), 6.23 (br d, J = 8.9 Hz, 0.8H, rotamer), 7.56 (br d, *J* = 8.6 Hz, 2H), 8.18 (d, *J* = 8.6 Hz, 2H).

^{13}C NMR (101 MHz, DMSO-d$_6$) δ -5.4, 17.9, 25.8, 27.6, 28.0, 57.5, 58.9, 62.3, 62.9, 69.9, 77.6, 122.8, 127.4, 146.4, 151.7, 155.1.

MS calcd for $C_{20}H_{35}N_2O_6Si$ $(M+H)^+$: 427.23; found: 427.40.

The spectroscopic data are in agreement with previously published results.[11]

***tert*-Butyl *N*-[(1*R*,2*R*)-3-[(*tert*-butyldimethylsilyl)oxy]-1-methoxy-1-(4-nitrophenyl)propan-2-yl]carbamate (SI-24)**

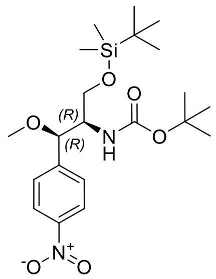

To a stirred solution of **SI-23** (50.0 g, 117 mmol) in DMF (500 ml, 0.23 M) was added LiHMDS (1 M in THF) (129 ml, 129 mmol) at −15 °C. After 10 minutes, iodomethane (7.30 ml, 117 mmol) was added. The mixture was stirred at −15 °C. After 45 minutes, TLC indicated complete conversion. The reaction was quenched with water and extracted with EtOAc (3x). The combined organic layers were washed 1N $KHSO_4$, sat. $NaHCO_3$ and brine, dried (Na_2SO_4) and concentrated in vacuo. Silica gel column chromatography (20% EtOAc in petroleum ether) yielded **SI-24** (40.0 g, 90.8 mmol, 78%) as a colorless oil.

^{1}H NMR (400 MHz, DMSO-d$_6$) δ 0.03 (s, 6H), 0.87 (s, 9H), 1.03 (br s, 1.8H, rotamer), 1.23 (s, 7.2H, rotamer), 3.17 (s, 3H), 3.54 – 3.36 (m, 1H), 3.77 – 3.56 (m, 2H), 4.51 (d, *J* = 3.3 Hz, 1H), 6.24 (br d, *J* = 7.9 Hz, 0.2H, rotamer), 6.60 (br d, *J* = 8.4 Hz, 0.8H, rotamer), 7.53 (d, *J* = 8.4 Hz, 2H), 8.21 (d, *J* = 8.6 Hz, 2H).

^{13}C NMR (101 MHz, DMSO-d$_6$) δ -5.5, -5.4, 17.9, 25.7, 27.6, 28.0, 57.0, 61.9, 77.6, 80.5, 123.2, 128.3, 146.9, 147.6, 155.1.

MS calcd for $C_{17}H_{29}N_2O_6Si^+$ $(M - C_4H_9 + 2H)^+$: 385.18; found: 385.36

The spectroscopic data are in agreement with previously published results.[12]

***tert*-butyl *N*-[(1*R*,2*R*)-3-hydroxy-1-methoxy-1-(4-nitrophenyl)propan-2-yl]carbamate (SI-25)**

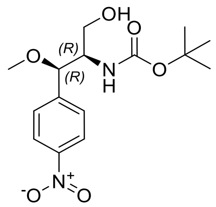

To a stirred solution of **SI-24** (50.0 g, 113 mmol) in THF (300 ml, 0.38 M) was added TBAF (32.6 g, 125 mmol) at 0 °C. The mixture was stirred at rt. After 2 h, TLC indicated complete conversion. The reaction was quenched with cold water and extracted with EtOAc (2x). The combined organic layers were dried (Na_2SO_4) and concentrated in vacuo. Silica gel column chromatography (35% EtOAc in petroleum ether) yielded **SI-25** (31.0 g, 95.0 mmol, 84%).

^{1}H NMR (400 MHz, DMSO-d$_6$) δ 1.05 (br s, 1.2H, rotamer), 1.24 (s, 7.2H, rotamer), 3.18 (s, 3H), 3.23 (br dd, J = 10.9, 5.3 Hz, 1H), 3.54 – 3.43 (m, 1H), 3.69 – 3.55 (m, 1H), 4.55 (d, *J* = 3.8 Hz, 1H), 4.77 (t, *J* = 5.1 Hz, 0.8H, rotamer), 4.83 (br s, 0.2H, rotamer), 6.08 (d, *J* = 8.4 Hz, 0.2H, rotamer), 6.45 (d, *J* = 9.1 Hz, 0.8H, rotamer), 7.53 (d, *J* = 8.4 Hz, 2H), 8.20 (d, *J* = 8.6 Hz, 2H).

^{13}C NMR (101 MHz, DMSO-d$_6$) δ 27.6, 28.0, 57.1, 58.4, 60.4, 60.6, 77.6, 80.4, 123.1, 128.2, 146.8, 148.1, 155.1.

MS calcd for $C_{15}H_{23}N_2O_6^+$ $(M+H)^+$: 327.16; found: 327.17.

The spectroscopic data are in agreement with previously published results.[12]

(2*S*,3*R*)-2-{[(*tert*-butoxy)carbonyl]amino}-3-methoxy-3-(4-nitrophenyl)propanoic acid (SI-26)

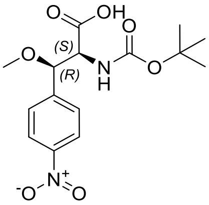

A stirred solution of **SI-25** (30.0 g, 91.9 mmol) in MeCN (30.0 ml) and 2N NaH_2PO_4 buffer (30.0 ml) (1.5 M) was cooled to 0 °C and (diacetoxyiodo)benzene (2.96 g, 9.2 mmol), TEMPO (2.87 g, 18.3 mmol) and $NaClO_2$ (29.0 g, 320 mmol) were added. The mixture was stirred at rt. After 2 h, TLC indicated complete conversion. The reaction was quenched with 2 M aq. Na_2CO_3 and washed with diethyl ether. The aqueous layer was acidified with 1 N aq. HCl and extracted with EtOAc (3x). The combined EtOAc layers were dried (Na_2SO_4) and concentrated in vacuo to yield crude **SI-26** (25.0 g, 73.5 mmol, 80%) which was used in the next step without further purification.

^{1}H NMR (500 MHz, DMSO-d$_6$) δ 0.95 (br s, 1.8H, rotamer), 1.20 (s, 7.2H, rotamer), 3.14 (s, 3H), 3.79 (d, *J* = 7.3 Hz, 1H), 3.85 (dd, *J* = 8.7, 2.0 Hz, 1H), 4.91 (d, *J* = 2.5 Hz, 1H), 5.40 (d, *J* = 7.3 Hz, 0.2H, rotamer), 5.79 (d, *J* = 8.5 Hz, 0.8H, rotamer), 7.54 (d, *J* = 8.5 Hz, 2H), 8.15 (d, *J* = 8.5 Hz, 1.6H, rotamer), 8.21 (d, *J* = 7.6 Hz, 0.4H, rotamer).

^{13}C NMR (126 MHz, DMSO-d$_6$) δ 27.5, 28.0, 57.2, 59.9, 61.1, 77.6, 83.0, 83.2, 122.9, 123.0, 128.1, 146.6, 148.4, 148.9, 154.0, 154.8, 170.7, 170.9.

MS calcd for $C_{11}H_{13}N_2O_7^+$ $(M - C_4H_9 + 2H)^+$: 285.07; found: 285.04.

The spectroscopic data are in agreement with previously published results.[12]

Methyl (2*S*,3*R*)-2-{[(*tert*-butoxy)carbonyl]amino}-3-methoxy-3-(4-nitrophenyl)propanoate (SI-27)

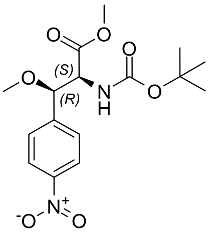

To a stirred solution of **SI-26** (35.0 g, 103 mmol) in DMF (300 ml, 0.34 M) were added potassium carbonate (28.4 g, 206 mmol) and iodomethane (9.61 ml, 154 mmol) at 0°C. The mixture was stirred at rt. After 2 h, TLC indicated complete conversion. The reaction was quenched with cold water and extracted with EtOAc (2x). The combined organic layers were dried (Na_2SO_4) and concentrated in vacuo. Silica gel column chromatography (15% EtOAc in petroleum ether) yielded **SI-27** (30.0 g, 84.7 mmol, 82%).

^{1}H NMR (500 MHz, DMSO-d$_6$) δ 1.11 (br s, 1H), 1.23 (s, 8H), 3.17 (s, 3H), 3.61 (s, 3H), 4.34 – 4.27 (m, 0.15H, rotamer), 4.40 (dd, *J* = 9.1, 4.4 Hz, 0.85H, rotamer), 4.82 (d, *J* = 3.2 Hz, 0.15H, rotamer), 4.88 (d, *J* = 4.4 Hz, 0.85H, rotamer), 6.80 (d, *J* = 8.5 Hz, 0.15H, rotamer), 7.19 (d, *J* = 9.1 Hz, 0.85H, rotamer), 7.63 (d, *J* = 8.5 Hz, 1H), 8.22 (d, *J* = 8.5 Hz, 2H).

^{13}C NMR (126 MHz, DMSO-d$_6$) δ 27.5, 27.9, 52.1, 57.2, 58.9, 60.2, 78.4, 81.3, 123.2, 128.5, 145.6, 147.2, 147.3, 153.6, 155.3, 170.1.

MS calcd for $C_{12}H_{15}N_2O_7^+$ $(M - C_4H_9 + 2H)^+$: 299.09; found: 299.05.

Methyl (2*S*,3*R*)-3-(4-aminophenyl)-2-{[(*tert*-butoxy)carbonyl]amino}-3-methoxypropanoate (SI-28)

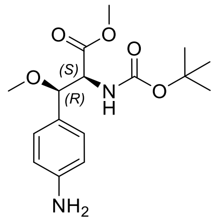

To a solution of **SI-27** (30.0 g, 84.7 mmol) in THF (300 ml, 0.28 M) was added 10 wt% Pd/C (9.0 g, 8.5 mmol). The mixture was stirred at rt under H_2 atmosphere (balloon pressure). After 5 h, TLC indicated complete conversion. Subsequently, the mixture was filtered over Celite and the filtrate was evaporated in vacuo. Silica gel column chromatography (5% MeOH in DCM) yielded **SI-28** (22.0 g, 67.8 mmol, 80%) as a colorless oil.

^{1}H NMR (500 MHz, DMSO-d_6) δ 1.23 (br s, 1.35H, rotamer), 1.34 (s, 7.65H, rotamer), 3.04 (s, 3H), 3.45 (s, 3H), 4.03 (br d, J = 6.0 Hz, 0.15H, rotamer), 4.13 – 4.06 (m, 0.85H, rotamer), 4.27 (br d, J = 5.4 Hz, 0.15H, rotamer), 4.31 (d, J = 6.3 Hz, 0.85H, rotamer), 5.07 (s, 2H), 6.50 (d, J = 8.2 Hz, 2H), 6.61 (br d, J = 6.9 Hz, 0.15H, rotamer), 6.91 (d, J = 8.5 Hz, 2H), 7.00 (d, J = 8.2 Hz, 0.85H, rotamer).

^{13}C NMR (126 MHz, DMSO-d_6) δ 27.7, 28.1, 51.6, 55.9, 60.0, 61.3, 78.3, 78.5, 81.8, 81.9, 113.5, 123.6, 123.8, 127.9, 148.6, 154.1, 155.4, 170.9.

MS calcd for $C_{11}H_{13}N_2O_4^+$ (M – C_4H_9 – OCH_3 + H)$^+$: 237.09; found: 237.28.

Methyl (2*S*,3*R*)-3-(4-bromophenyl)-2-{[(*tert*-butoxy)carbonyl]amino}-3-methoxypropanoate (SI-29)

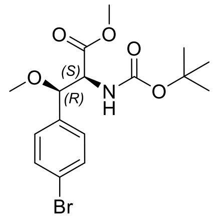

To a stirred solution of **SI-28** (15.0 g, 46.2 mmol) in DCM (75.0 ml, 0.28 M) was added bromotrichloromethan (18.3 g, 92.4 mmol) at 0 °C. After 15 minutes, sodium nitrite (16.0 g, 231 mmol), dissolved in water (75.0 ml), was added. After 10 minutes, acetic acid (55.5 g, 925 mmol) was added at 0°C. The mixture was stirred at rt. After 2 h, TLC indicated complete conversion. The reaction was quenched with water and extracted with DCM (2x). The combined organic layers were dried (Na_2SO_4) and concentrated in vacuo. Silica gel column chromatography (20% EtOAc in petroleum ether) yielded **SI-29** (15.0 g, 38.6 mmol, 84%).

^{1}H NMR (500 MHz, DMSO-d_6) δ 1.15 (br s, 1.35H, rotamer), 1.28 (s, 7.65H, rotamer), 3.12 (s, 3H), 3.56 (s, 3H), 4.20 (d, *J* = 4.7 Hz, 0.15H, rotamer), 4.27 (dd, *J* = 8.8, 5.0 Hz, 0.85H, rotamer), 4.60 (br d, *J* = 4.1 Hz, 0.15H, rotamer), 4.64 (d, *J* = 5.0 Hz, 0.85H, rotamer), 6.71 (d, *J* = 8.2 Hz, 0.15H, rotamer), 7.12 (d, *J* = 8.8 Hz, 0.85H, rotamer), 7.28 (d, *J* = 8.2 Hz, 1H), 7.54 (d, *J* = 8.5 Hz, 1H).

^{13}C NMR (126 MHz, DMSO-d_6) δ 27.6, 28.0, 51.9, 56.8, 59.3, 60.5, 78.4, 81.3, 121.1, 129.4, 131.0, 137.0, 153.7, 155.3, 170.4.

MS calcd for $C_{12}H_{15}BrNO_5^+$ (M – C_4H_9 +2H)$^{+:}$ 332.01, found: 331.98.

Methyl (2*S*,3*R*)-2-{[(*tert*-butoxy)carbonyl]amino}-3-methoxy-3-[4-(4,4,5,5-tetramethyl-1,3,2-dioxaborolan-2-yl)phenyl]propanoate (SI-29a)

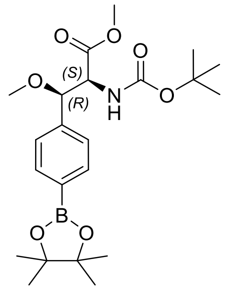

A stirred solution of **SI-29** (10.0 g, 25.8 mmol) in 1,4-Dioxane (100 ml, 0.26 M) was degassed with Argon and bis(pinacolato)diboron (7.85 g, 30.9 mmol), potassium acetate (7.57 g, 77.2 mmol) and tetrakis(triphenylphosphine)palladium (1.49 g, 1.29 mmol) were added. The mixture was stirred at 110°C. After 12 h, TLC indicated complete conversion. The reaction was quenched with water and extracted with EtOAc (2x). The combined organic layers were dried (Na_2SO_4) and concentrated in vacuo. Silica gel column chromatography (20% EtOAc in petroleum ether) yielded **SI-29a** (8.00 g, 18.4 mmol, 71%).

^{1}H NMR (500 MHz, $CDCl_3$) δ 1.32 (s, 9H), 1.33 (s, 12H), 3.25 (s, 3H), 3.76 (s, 3H), 4.46 (dd, *J* = 9.4, 3.0 Hz, 1H), 4.77 (d, *J* = 2.9 Hz, 1H), 5.27 (d, *J* = 9.3 Hz, 1H), 7.32 (d, *J* = 7.9 Hz, 2H), 7.80 (d, *J* = 7.9 Hz, 2H).

MS calcd for $C_{22}H_{35}BNO_7^+$ $(M+H)^+$: 436.25; found: 436.27.

No ^{13}C NMR data was obtained for this compound.

Methyl (2*S*,3*R*)-2-{[(*tert*-butoxy)carbonyl]amino}-3-(4-hydroxyphenyl)-3-methoxypropanoate (SI-30)

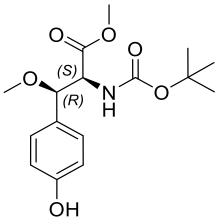

To a stirred solution of **SI-29a** (10.0 g, 23.0 mmol) in THF (100 ml, 0.23 M) was added 30% H_2O_2 (30 ml, 265 mmol) at 0°C and the mixture was stirred at rt. After 12 h, TLC indicated complete conversion. The reaction was quenched with water and extracted with EtOAc (2x). The combined organic layers were dried (Na_2SO_4) and concentrated in vacuo. Silica gel column chromatography (20% EtOAc in petroleum ether) yielded **SI-30** (6.00 g, 18.4 mmol, 80%).

^{1}H NMR (500 MHz, DMSO-d_6) δ 1.20 (br s, 1.35H, rotamer), 1.32 (s, 7.65H, rotamer), 3.07 (s, 1H), 3.46 (s, 3H), 4.07 (t, *J* = 6.1 Hz, 0.15H, rotamer), 4.14 (dd, *J* = 8.2, 6.3 Hz, 0.85H, rotamer), 4.38 (d, *J* = 5.0 Hz, 0.15H, rotamer), 4.43 (d, *J* = 5.7 Hz, 0.85H, rotamer), 6.65 (d, *J* = 7.3 Hz, 0.15H, rotamer), 6.71 (d, *J* = 8.2 Hz, 2H), 7.05 (d, *J* = 8.5 Hz, 0.85H, rotamer), 7.08 (d, *J* = 8.5 Hz, 2H), 9.40 (br s, 1H).

^{13}C NMR (126 MHz, DMSO-d_6) δ 27.7, 28.1, 51.7, 56.2, 59.9, 61.2, 78.3, 78.5, 81.6, 81.7, 114.9, 127.3, 128.4, 153.9, 155.4, 157.2, 170.8.

MS calcd for $C_{16}H_{24}NO_6^+$ $(M+H)^+$: 326.16; found: 326.14.

Methyl (2*S*,3*R*)-2-{[(*tert*-butoxy)carbonyl]amino}-3-methoxy-3-[4-(prop-2-yn-1-yloxy)phenyl]propanoate (SI-31)

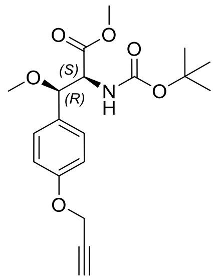

To a stirred solution of **SI-30** (5.00 g, 15.4 mmol) in DMF (8.0 ml, 1.9 M) were added potassium carbonate (2.76 g, 20.0 mmol) and propargyl bromide (2.00 g, 16.9 mmol). The mixture was stirred at rt. After 12 h, TLC indicated complete conversion. The reaction was quenched with water and extracted with EtOAc (2x). The combined organic layers were dried (Na_2SO_4) and concentrated in vacuo. Silica gel column chromatography (20% EtOAc in petroleum ether) yielded **SI-31** (4.00 g, 11.0 mmol, 72%).

^{1}H NMR (400 MHz, DMSO-d_6) δ 1.17 (br s, 1.8H, rotamer), 1.30 (s, 7.2H, rotamer), 3.09 (s, 3H), 3.49 (s, 3H), 3.53 (t, *J* = 2.4 Hz, 1H), 4.15 – 4.06 (m, 0.2H, rotamer), 4.18 (dd, *J* = 8.5, 5.7 Hz, 0.8H, rotamer), 4.49 (d, *J* = 4.6 Hz, 0.2H,

rotamer), 4.53 (d, *J* = 5.6 Hz, 0.8H, rotamer), 4.77 (d, *J* = 2.3 Hz, 2H), 6.66 (d, *J* = 7.6 Hz, 0.2H, rotamer), 6.95 (d, *J* = 8.6 Hz, 1H), 7.08 (d, *J* = 8.4 Hz, 0.8H, rotamer), 7.23 (d, *J* = 8.6 Hz, 2H).

^{13}C NMR (101 MHz, DMSO-d$_6$) δ 27.7, 28.2, 51.8, 55.4, 56.5, 59.8, 61.1, 78.2, 78.5, 79.3, 81.5, 114.5, 128.4, 130.0, 155.4, 157.0, 170.8.

MS cald for $C_{19}H_{26}NO_6^+$ (M+H)$^+$: 364.18; found: 364.45.

(2*S*,3*R*)-2-{[(*tert*-butoxy)carbonyl]amino}-3-methoxy-3-[4-(prop-2-yn-1-yloxy)phenyl]propanoic acid (SI-32)

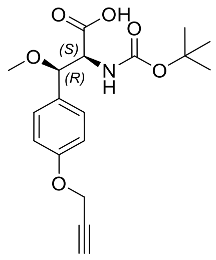

To a stirred solution of **SI-31** (5.00 g, 13.8 mmol) in THF (10.0 ml) and water (10.0 ml) (0.69 M) was added LiOH·H_2O (2.31 g, 55.2 mmol) and the mixture was stirred at rt. After 12 h, TLC indicated complete conversion. The reaction was diluted with water and washed with diethyl ether. The aqueous layer was acidified with 1 M HCl (aq.) and extracted with EtOAc (2x). The combined EtOAc layers were dried (Na_2SO_4) and concentrated in vacuo to yield **SI-32** (3.50 g, 10.0 mmol, 73%) which was used in the next step without further purification.

$[\alpha]_{20}^{D}$ = –28.1 (c 1.0, $CHCl_3$).

Major rotamer: **^{1}H NMR** (500 MHz, $CDCl_3$) δ 1.34 (s, 9H), 2.52 (t, *J* = 2.4 Hz, 1H), 3.28 (s, 3H), 4.50 (dd, *J* = 9.2, 3.0 Hz, 1H), 4.68 (d, *J* = 2.5 Hz, 2H), 4.82 (d, *J* = 3.0 Hz, 1H), 5.31 (d, *J* = 9.0 Hz, 1H), 6.94 – 7.02 (m, 2H), 7.23 – 7.28 (m, 2H). **^{13}C NMR** (126 MHz, $CDCl_3$) δ 20.9, 28.4, 55.9, 57.6, 59.1, 75.7, 78.6, 80.2, 82.0, 115.0, 128.3, 128.4, 129.9, 155.7, 157.7, 175.2.

Minor rotamer (ratio ~4:1, selected signals): **^{1}H NMR** (500 MHz, $CDCl_3$) δ 1.18 (s, 9H), 3.25 (s, 3H), 4.34 (m, 1H), 4.73 – 4.76 (m, 1H). **^{13}C NMR** (126 MHz, $CDCl_3$) δ 28.0, 60.7, 81.0, 82.4, 128.4, 177.2.

HRMS (ESI) calcd for $C_{18}H_{24}NO_6^+$ (M+H)$^+$: 350.1598; found: 350.1593.

Methyl (2*S*)-2-[(2*S*)-2-[(2*S*,3*R*)-2-{[(*tert*-butoxy)carbonyl]amino}-3-methoxy-3-[4-(prop-2-yn-1-yloxy)phenyl]propanamido]-*N*,3-dimethylbutanamido]-4-methylpentanoate (SI-33)

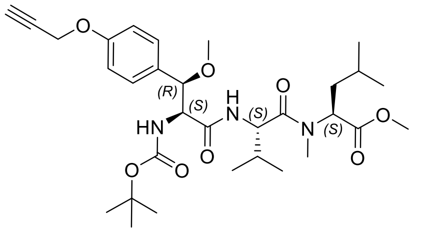

To a stirred solution of methyl *N*-(L-valyl)-*N*-methyl-L-leucinate[4] (3.00 g, 11.6 mmol) and **SI-32** (4.06 g, 11.6 mmol) in DMF (25.0 ml, 0.46 M) was added DIPEA (4.06 ml, 23.2 mmol) at 0 °C. After 10 minutes, HATU (4.86 g, 12.8 mmol) was added and the mixture was stirred at rt. After 12 h, TLC indicated complete conversion. The reaction was quenched with water and extracted with EtOAc (2x). The combined EtOAc layers were washed with brine, dried (Na_2SO_4) and concentrated in vacuo. Silica gel column chromatography (50% EtOAc in petroleum ether) yielded **SI-33** (2.90 g, 4.92 mmol, 42%).

Major rotamer: **^{1}H NMR** (400 MHz, $CDCl_3$) δ 0.91 (d, *J* = 6.5 Hz, 3H), 0.92 – 0.95 (m, 6H), 1.00 (d, *J* = 6.8 Hz, 3H), 1.34 (s, 9H), 1.41 – 1.53 (m, 1H), 1.64 – 1.82 (m, 2H), 2.05 – 2.17 (m, 1H), 2.50 (t, *J* = 2.4 Hz, 1H), 3.01 (s, 3H), 3.28 (s, 3H), 3.69 (s, 3H), 4.38 (dd, *J* = 8.3, 3.0 Hz, 1H), 4.65 (d, *J* = 2.4 Hz, 2H), 4.80 – 4.90 (m, 2H), 5.25 (d, *J* = 8.3 Hz, 1H), 5.32 (dd, *J* = 10.4, 5.4 Hz, 1H), 6.85 – 6.96 (m, 2H), 7.19 (d, *J* = 8.3 Hz, 2H), 7.29 (d, *J* = 8.9 Hz, 1H). **^{13}C NMR**

(101 MHz, $CDCl_3$) δ 17.3, 19.6, 21.5, 23.4, 25.0, 28.4, 31.5, 31.7, 37.1, 52.3, 54.1, 54.7, 55.9, 57.5, 59.8, 75.7, 78.7, 80.2, 81.5, 114.8, 128.1, 130.3, 155.5, 157.5, 169.4, 172.2, 172.3.

Minor rotamer (selected signals, ratio ~12:1): **^{1}H NMR** (400 MHz, $CDCl_3$) δ 1.31 (s, 9H), 2.81 (s, 3H), 3.24 (s, 3H), 3.61 (s, 3H), 4.27 – 4.32 (m, 1H), 5.20 (d, *J* = 9.1 Hz, 1H).

HRMS calcd for $C_{31}H_{48}N_3O_8^+$ $(M+H)^+$: 590.3436; found: .590.3450.

methyl (2S)-2-[(2S)-2-[(2S,3R)-2-[(2S)-2-{[(tert-butoxy)carbonyl]amino}propanamido]-3-methoxy-3-[4-(prop-2-yn-1-yloxy)phenyl]propanamido]-N,3-dimethylbutanamido]-4-methylpentanoate (SI-34)

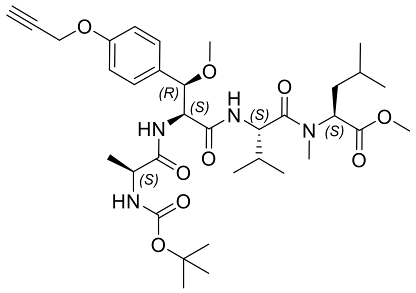

To a solution of **SI-33** (2.80 g, 4.75 mmol) in DCM (20.0 ml, 0.24 M) was added HCl (4N in dioxane) (10.0 ml) dropwise at 0°C and the mixture was allowed to reach rt and stirred for 3 h. The solvents were evaporated in vacuo and the crude amine was used in the peptide coupling without further purification.

To a solution of the deprotected tripeptide (5.00 g, 10.2 mmol) in DMF (50.0 ml, 0.20 M) was added DIPEA (1.55 ml, 8.87 mmol) at 0°C followed by addition of Boc-Ala-OH (1.93 g, 10.2 mmol) and HATU (4.27 g, 11.2 mmol). The mixture was allowed to reach rt and stirred for 16 . The reaction was quenched with water and extracted with EtOAc. The organic layer was washed with water and brine, dried (Na_2SO_4) and concentrated in vacuo. Silica gel column chromatography (50% EtOAc in petroleum ether) followed by prep. HPLC yielded **SI-34** (3.00 g, 4.54 mmol, 45%) as a white solid.

$[\alpha]_{20}^{D}$ = –47.9 (c 1.0, $CHCl_3$).

Major rotamer: **^{1}H NMR** (500 MHz, $CDCl_3$) δ 0.90 – 0.94 (m, 6H), 0.96 (d, *J* = 6.7 Hz, 3H), 0.98 (d, *J* = 6.8 Hz, 3H), 1.30 (d, *J* = 7.0 Hz, 3H), 1.45 (s, 9H), 1.49 – 1.53 (m, 1H), 1.66 – 1.79 (m, 2H), 2.06 – 2.17 (m, 1H), 2.51 (t, *J* = 2.4 Hz, 1H), 2.99 (s, 3H), 3.30 (s, 3H), 3.69 (s, 3H), 4.08 – 4.16 (m, 1H), 4.64 – 4.67 (m, 3H), 4.77 – 4.83 (m, 2H), 4.89 – 4.96 (m, 1H), 5.35 (dd, *J* = 10.5, 5.3 Hz, 1H), 6.75 (d, *J* = 7.3 Hz, 1H), 6.85 – 6.90 (m, 2H), 7.14 (d, *J* = 8.4 Hz, 2H), 7.38 (d, *J* = 8.7 Hz, 1H). **^{13}C NMR** (126 MHz, $CDCl_3$) δ 17.2, 18.4, 19.6, 21.4, 23.3, 24.9, 28.3, 31.3, 31.4, 36.9, 50.4, 52.2, 54.1, 54.5, 55.7, 57.4, 57.6, 75.6, 78.5, 80.2, 80.8, 114.7, 128.2, 129.7, 155.4, 157.5, 168.4, 172.0, 172.1, 172.4.

Minor rotamer (ratio ~10:1, selected signals) **^{1}H NMR** (500 MHz, $CDCl_3$) δ 1.48 (s, 9H), 2.84 (s, 3H), 3.61 (s, 3H).

HRMS (ESI) calcd for $C_{34}H_{53}N_4O_9^+$ $(M+H)^+$: 661.3807; found: 661.3778.

Methyl *N*-(((2*S*,3*R*)-3-(allyloxy)-2-((2*S*)-2-((4*R*)-5-((tert-butyldimethylsilyl)oxy)-4-methyl-2-(methylamino)pentanamido)propanamido)-3-phenylpropanoyl)-L-valyl)-*N*-methyl-L-leucinate (SI-35)

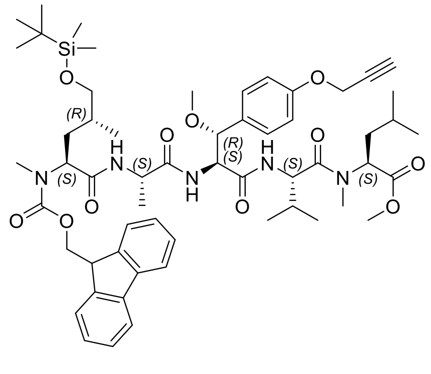

A 25 ml round-bottom flask was charged with **SI-34** (661 mg, 1.00 mmol), 4 N HCl in 1,4-dioxane (2.50 ml, 10.0 equiv) was added and the reaction mixture was stirred at 25 °C. After 2 h, the solvent was evaporated and

the residue was reacted according to *GP4*: **SI-21** (484 mg, 1.00 mmol), HOBt (166 mg, 1.10 mmol), EDC (211 mg, 1.10 mmol), NMM (220 µl, 2.00 mmol) (18 h). Flash chromatography (CyH/EtOAc 100:0 – 60:40) followed by lyophilization afforded **SI-35** (850 mg, 820 µmol, 82% yield) as a white amorphous solid. R_f = 0.43 (PE/EtOAc 1:1).

$[\boldsymbol{\alpha}]_{20}^{\mathbf{D}}$ = –53.9 (c 0.5, $CHCl_3$).

^{1}H NMR (500 MHz, $CDCl_3$) δ -0.31 – 0.32 (m, 6H), 0.88 (s, 9H), 0.91 – 0.97 (m, 12H), 0.99 (d, *J* = 6.8 Hz, 3H), 1.29 (d, *J* = 7.0 Hz, 3H), 1.45 – 1.61 (m, 3H), 1.67 – 1.81 (m, 2H), 1.93 – 2.05 (m, 1H), 2.08 – 2.17 (m, 1H), 2.36 – 2.59 (m, 1H), 2.82 (s, 3H), 2.98 (s, 3H), 3.29 (s, 3H), 3.36 – 3.56 (m, 2H), 3.69 (s, 3H), 4.26 (t, *J* = 6.7 Hz, 1H), 4.29 – 4.38 (m, 1H), 4.42 – 4.54 (m, 2H), 4.60 – 4.69 (m, 3H), 4.71 – 4.78 (m, 2H), 4.80 (dd, *J* = 8.7, 5.8 Hz, 1H), 5.35 (dd, *J* = 10.5, 5.2 Hz, 1H), 6.36 – 6.74 (m 2H), 6.88 (d, *J* = 8.4 Hz, 2H), 7.12 (d, *J* = 8.4 Hz, 2H), 7.28 – 7.35 (m, 2H), 7.36 – 7.49 (m, 3H), 7.52 – 7.63 (m, 2H), 7.76 (d, *J* = 7.6 Hz, 2H).

^{13}C NMR (126 MHz, $CDCl_3$) δ -5.3, -3.5, 17.3, 17.6, 18.4, 19.7, 21.5, 23.4, 24.9, 26.1, 31.4, 31.5, 37.0, 47.4, 49.3, 52.3, 54.2, 54.7, 55.8, 56.8, 57.5, 57.7, 67.2, 67.9, 75.7, 80.9, 114.8, 120.1, 125.0, 127.2, 127.9, 128.2, 129.6, 141.4, 143.9, 157.3, 157.7, 168.4, 170.9, 171.6, 172.07, 172.10, 172.14.

HRMS (ESI): calcd for $C_{57}H_{82}N_5O_{11}Si^+$ (M+H)$^+$: 1040.5775; found: 1040.5730.

Methyl *N*-(((2*S*,3*R*)-2-((2*S*)-2-((2*S*)-2-(2-(((allyloxy)carbonyl)amino)-*N*-methyl-3-(1-methyl-1*H*-indol-3-yl)propanamido)-5-((tert-butyldimethylsilyl)oxy)-4-methylpentanamido)propanamido)-3-methoxy-3-(4-(prop-2-yn-1-yloxy)phenyl)propanoyl)-L-valyl)-*N*-methyl-L-leucinate (SI-36)

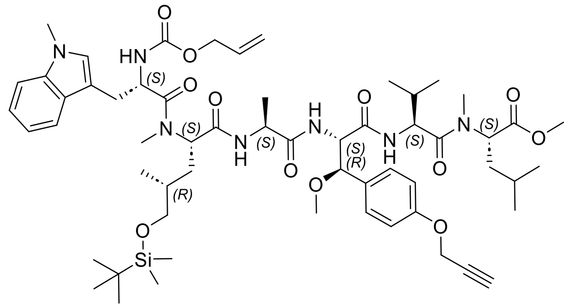

SI-35 (400 mg, 384 µmol) was dissolved in DCM (4.0 ml, 0.1 M), tris(2-aminoethyl)amine (574 µl, 3.84 mmol) was added and the reaction mixture was stirred vigorously. After completion of the reaction (TLC-monitoring; 30 minutes) the reaction mixture was washed with saturated NaCl solution (20 ml ×3) and phosphate buffer (67.0 mM, pH 5.5) (30 ml ×3). The aqueous phase was back-extracted with DCM (1x20 ml). The organic phase was dried (Na_2SO_4) and evaporated in vacuo. The resulting crude peptide was reacted according to *GP*3: Alloc-Trp(Me)-OH (141 mg, 468 µmol), BEP (128 mg, 468 µmol), NMM (107 µl, 975 µmol) (18 h). Flash chromatography (CyH/EtOAc 100:0 – 45:55) followed by lyophilization afforded **SI-36** (309 mg, 280 µmol, 72%) as a white amorphous solid. R_f = 0.38 (PE/EtOAc 2:3).

$[\boldsymbol{\alpha}]_{20}^{\mathbf{D}}$ = –41.3 (c 0.4, $CHCl_3$).

^{1}H NMR (500 MHz, $CDCl_3$) *(mixture of rotamers, ratio ~2:1)* δ -0.04 (d, *J* = 10.4 Hz, 2.3H), -0.02 (d, *J* = 5.7 Hz, 6.1H), 0.09 (s, 0.7H), 0.32 (d, *J* = 6.8 Hz, 0.1 H), 0.36 (s, 0.2H), 0.46 (d, *J* = 6.6 Hz, 1.H), 0.82 (s, 3.6H), 0.87 (s, 10.2H), 0.89 – 1.01 (m, 23.9H), 1.19 (d, *J* = 7.0 Hz, 3H), 1.22 – 1.28 (m, 2.3H), 1.38 (td, *J* = 7.2, 1.8 Hz, 0.9), 1.42– 1.54 (m, 3.9H), 1.66 – 1.81 (m, 3.1H), 1.88 – 2.44 (m, 5.7H), 2.47–2.55 (m, 1.7H), 2.64–2.69 (m, 0.3H), 2.70–2.78 (m, 3.7H), 2.78 – 2.94 (m, 1.5H), 2.93–2.99 (m, 2.0H), 3.01 (s, 2.5H), 3.03 – 3.24 (m, 3.5H), 3.25 (s, 1.3H) 3.30 (s, 3.0H), 3.35 – 3.49 (m, 2.2H), 3.56 – 3.65 (m, 0.4H), 3.66 – 3.70 (m, 6.8H), 3.72 (s, 1.9 H), 4.03 (td, *J* = 7.3, 2.6 Hz, 0.5H), 4.12–4.35 (m, 1.47H), 4.40–4.58 (m, 3.1H), 4.59 (d, *J* = 2.4 Hz, 0.7H), 4.60 – 4.63 (m, 0.2H), 4.64 – 4.66 (m, 2.3H), 4.67–4.79 (m, 1.1H), 4.74 – 4.84 (m, 3.1H), 4.87 (q, *J* = 6.5, 6.0 Hz, 1.2H), 4.93–5.00 (m, 0.87H), 5.13 – 5.31 (m, 3.2H), 5.33–5.41 (m, 1.5H), 5.58–5.67 (m, 0.3H), 5.77 – 5.97 (m, 2.0H), 6.31 – 6.48 (m, 0.8H), 6.65 – 6.99 (m, 6.3H), 7.06 – 7.24 (m, 6.4H), 7.24 – 7.30 (m, 2.3H), 7.33 – 7.44 (m, 1.0H), 7.49 (d, *J* = 8.7 Hz, 0.8H), 7.54 – 7.59 (m, 0.5 H), 7.64 (d, *J* = 7.9 Hz, 0.9H), 7.87 (d, *J* = 6.6 Hz, 0.3H).

^{13}C NMR (126 MHz, $CDCl_3$) *(mixture of rotamers)* δ -5.29, -5.27, -3.4, 14.8, 15.7, 17.3, 17.4, 17.6, 17.9, 18.5, 19.6, 19.7, 21.5, 23.42, 23.45, 24.94, 24.97, 25.7, 26.0, 26.1, 28.8, 31.36, 31.40, 31.44, 32.2, 32.8, 37.0, 49.8, 50.0, 51.1,

51.7, 52.2, 52.3, 54.2, 54.4, 54.6, 54.7, 55.79, 55.82, 57.46, 57.54, 57.8, 57.9, 58.1, 65.8, 66.4, 67.4, 68.4, 75.7, 78.6, 81.1, 108.4, 108.6, 109.49, 109.54, 114.7, 114.8, 117.7, 118.4, 118.7, 118.8, 119.4, 119.6, 121.9, 122.2, 127.8, 128.0, 128.26, 128.30, 129.7, 132.2, 132.8, 136.9, 137.0, 156.1, 156.8, 157.5, 157.6, 168.7, 169.0, 170.5, 171.7, 172.1, 172.18, 172.24, 172.3, 172.6, 173.4.

Selected diagnostic peaks:

Major rotamer: **^{1}H NMR** (500 MHz, $CDCl_3$) *δ* -0.02 (s, 3H), -0.01 (s, 3H), 0.87 (s, 9H), 1.19 (d, *J* = 7.0 Hz, 3H), 2.50 – 2.52 (m, 1H), 2.75 (s, 3H), 3.00 (s, 3H), 3.30 (s, 3H), 3.69 (s, 3H), 4.13 – 4.20 (m, 1H), 6.36 – 6.42 (m, 1H), 7.49 (d, *J* = 8.7 Hz, 1H), 7.64 (d, *J* = 7.9 Hz, 1H). **^{13}C-NMR** (126 MHz, $CDCl_3$) *δ* 23.42, 24.97, 26.1, 37.0, 55.82, 157.6.

Minor rotamer: **^{1}H NMR** (500 MHz, $CDCl_3$) *δ* -0.05 (s, 3H), -0.04 (s, 3H), 0.82 (s, 9H), 2.48 – 2.49 (m, 1H), 2.73 (s, 3H), 2.97 (s, 3H), 3.25 (s, 3H), 3.68 (s, 3H), 4.01 – 4.07 (m, 1H), 6.29 – 6.34 (m, 1H) **^{13}C-NMR** (126 MHz, $CDCl_3$) *δ* 23.45, 24.94, 26.0, 55.79, 157.5.

HRMS (ESI): calcd for $C_{58}H_{88}N_7O_{12}Si^+$ $(M+H)^+$: 1102.6255; found: 1102.6209.

Methyl *N*-(((2*S*,3*R*)-2-((2*S*)-2-((2*S*)-2-(2-(2-(((allyloxy)carbonyl)amino)-3-methylbutanamido)-*N*-methyl-3-(1-methyl-1*H*-indol-3-yl)propanamido)-5-((tert-butyldimethylsilyl)oxy)-4methylpent anamido)propanamido)-3-methoxy-3-(4-(prop-2-yn-1-yloxy)phenyl)propanoyl)-L-valyl)-*N*-methyl-L-leucinate (SI-37)

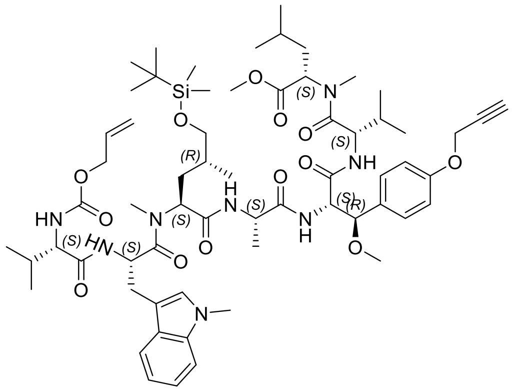

Prepared according to *GP2* and *GP4*: **SI-36** (150 mg, 136 µmol), $Pd(PPh_3)_4$ (4.72 mg, 4.10 µmol), DMBA (64.0 mg, 408 µmol); Alloc-Val-OH (35.6 mg, 177 µmol), NMM (30.0 µl, 272 µmol), HOBt (22.6 mg, 150 µmol), EDC (28.7 mg, 150 µmol) (18 h). Flash chromatography (CyH/EtOAc 100:0 – 60:40) followed by lyohpilization afforded **SI-37** (77.0 mg, 64.0 µmol, 47% yield) as a white amorphous solid. R_f = 0.42 (PE/EtOAc 2:3).

$[\alpha]^{D}_{20}$ = –53.5 (c 0.5, $CHCl_3$).

^{1}H NMR (500 MHz, $CDCl_3$) *(mixture of rotamers)* δ -0.04 (d, *J* = 7.8 Hz, 2.0H), 0.02 (d, *J* = 5.6 Hz, 4.1H), 0.09 (s, 0.7H), 0.36 (s, 0.4H), 0.45 (d, *J* = 6.7 Hz, 0.7H), 0.83 (s, 5.7H), 0.87 (s, 7.8H), 0.88 – 1.05 (m, 23.2H), 1.15 – 1.26 (m, 3.0H), 1.37 – 1.49 (m, 1.9H), 1.61 – 1.86 (m, 2.0H), 1.89 – 2.24 (m, 3.2H), 2.41 – 2.58 (m, 1.1H), 2.70 (s, 0.7H), 2.77 (s, 1.1H), 2.81 – 2.94 (m, 1.6H), 2.94 – 3.02 (m, 3.2H), 3.04 – 3.24 (m, 2.3H), 3.24 – 3.36 (m, 4.0H), 3.41 – 3.46 (m, 1.1H), 3.62 – 3.78 (m, 7.0H), 4.04 (t, *J* = 8.2 Hz, 0.8H), 4.11 – 4.39 (m, 0.5H), 4.48 – 4.91 (m, 8.3H), 5.13 – 5.44 (m, 4.4H), 5.79 – 6.00 (m, 1.0H), 6.39 (d, *J* = 7.1 Hz, 0.4H), 6.72 – 7.03 (m, 4.8H), 7.07 – 7.25 (m, 4.9H), 7.35 – 7.77 (m, 3.2H).

^{13}C NMR (126 MHz, $CDCl_3$) *(mixture of rotamers)* δ -5.3, -5.25, -5.23, 1.1, 14.3, 16.0, 17.3, 17.6, 17.7, 18.1, 18.4, 18.5, 19.2, 19.3, 19.66, 19.69, 21.5, 23.4, 25.0, 26.06, 26.1, 28.3, 28.7, 29.3, 29.8, 31.2, 31.37, 31.44, 31.5, 31.8, 32.2, 32.3, 32.8, 37.1, 49.4, 50.0, 50.1, 50.2, 52.27, 52.29, 54.20, 54.23, 54.6, 54.7, 55.83, 55.85, 57.4, 57.5, 57.6, 57.8, 58.3, 60.1, 60.1, 65.9, 66.1, 67.0, 68.5, 75.8, 78.6, 80.8, 81.0, 108.40, 108.44, 109.5, 109.6, 114.7, 114.88, 117.91, 118.1, 118.7, 119.5, 122.0, 127.8, 127.9, 128.0, 128.4, 128.6, 128.7, 129.7, 129.8, 132.1, 132.1, 132.2, 132.3, 132.84, 133.1, 137.0, 137.1, 156.2, 156.4, 157.6, 157.7, 168.4, 168.5, 168.8, 170.3, 170.9, 171.5, 171.7, 171.9, 172.1, 172.2, 172.2, 172.4, 172.8.

Selected diagnostic peaks:

Major rotamer: ^{1}H NMR (500 MHz, $CDCl_3$) δ 0.01 (s, 3H), 0.02 (s, 3H), 0.87 (s, 9H), 1.15 – 1.24 (m, 3H), 2.50 – 2.54 (m, 1H), 2.77 (s, 3H), 2.98, (s, 3H), 3.30 (s, 3H), 3.69 (s, 3H), 4.21 – 4.27 (m, 1H), 5.30 – 5.40 (m, 1H), 5.82 – 6.00 (m, 1H), 6.36 – 6.43 (m, 1H), 7.62 – 7.72 (m, 2H). **^{13}C-NMR** (126 MHz, $CDCl_3$) δ 19.66, 26.09, 37.1, 60.14, 81.0, 109.6, 137.1, 157.7.

Minor rotamer: **^{1}H NMR** (500 MHz, $CDCl_3$) δ -0.05 (s, 3H), -0.03 (s, 3H), 0.83 (s, 9H), 2.48 – 2.50 (m, 1H), 2.70 (s, 3H), 2.97 (s, 3H), 3.27 (s, 3H), 3. 68 (s, 3H), 5.74 – 5.79 (m, 1H). 8.08 – 8.27 (m, 1H). **^{13}C-NMR** (126 MHz, $CDCl_3$) δ 19.69, 26.06, 60.07, 80.8, 109.5, 136.0, 157.6.

HRMS (ESI): calcd for $C_{63}H_{97}N_8O_{13}Si^+$(M+H)$^+$: 1201.6939; found: 1201.6901.

(3*S*,6*S*,9*S*,12*S*,15*S*,18*S*,21*S*)-21-((*R*)-3-Hydroxy-2-methylpropyl)-9-isobutyl-6,12-diisopropyl-15-((*R*)-methoxy(4-(prop-2-yn-1-yloxy)phenyl)methyl)-1,10,18-trimethyl-3-((1-methyl-1*H*-indol-3-yl)methyl)-1,4,7,10,13,16,19-heptaazacyclohenicosane-2,5,8,11,14,17,20-heptaone (SI-38)

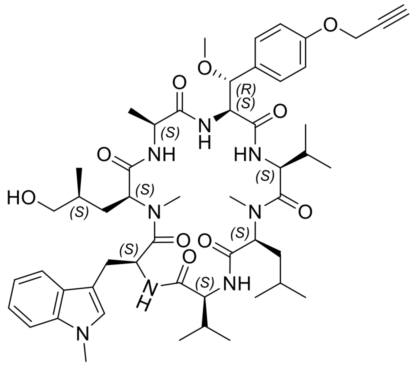

Prepared according to *GP6a*: **SI-37** (75.0 mg, 62.0 µmol), 1 M LiOH (75.0 µl, 75.0 µmol) (3.5 h); $Pd(OAc)_2$ (420 µg, 2.00 µmol), TPPTS (2.14 mg, 3.72 µmol), Et_2NH (32.0 µl, 310 µmol) (1 h); HATU (83.0 mg, 0.22 mmol), DIPEA (42.0 µl, 250 µmol) (addition over 1.5 h, additional 16 h); NH_4F (46.0 mg, 1.23 mmol) (17 h). RP flash chromatography (H_2O/MeCN 90:10 - 5:95) followed by lyophilizaiton afforded **SI-38** (22.0 mg, 23.0 µmol, 37%) as an off-white, amorphous solid.

$[\alpha]_{20}^{D}$ = –65.3 (c 0.5, $CHCl_3$)

^{1}H NMR (500 MHz, $CDCl_3$) δ -0.61 – -0.49 (m, 1H), 0.31 (d, *J* = 6.7 Hz, 3H), 0.52–0.77(m, 6H), 0.71 – 0.83 (m, 1H), 0.90–1.07 (m, 10H), 1.07 (d, *J* = 6.6 Hz, 3H), 1.17 (d, *J* = 7.2 Hz, 3H), 1.49 – 1.65 (m, 1H), 1.78–1.93 (m, 7.4 Hz, 1H), 1.93–2.17 (m, 2H), 2.20 – 2.28 (m, 1H), 2.29 – 2.41 (m, 1H), 2.50 (t, *J* = 2.4 Hz, 1H), 2.57 (s, 3H), 2.61 – 2.69 (m, 1H), 2.84 (s, 3H), 2.91–2.98 (m, 1H), 2.99–3.07 (m, 1H), 3.09–3.17 (m, 1H), 3.26–3.31 (m, 1H), 3.72 (s, 3H), 4.06 (t, *J* = 9.4 Hz, 1H), 4.33 (dd, *J* = 10.8, 2.5 Hz, 1H), 4.48 (t, *J* = 8.6 Hz, 1H), 4.61 (d, *J* = 2.4 Hz, 2H), 4.72 – 4.81 (m, 2H), 4.85 (dd, *J* = 10.4, 3.5 Hz, 1H), 4.90 (t, *J* = 5.1 Hz, 1H), 5.02 (d, *J* = 5.4 Hz, 1H), 6.67 – 6.96 (m, 3H), 6.86 – 7.02 (m, 1H), 7.01–7.17 (m, 3H), 7.17 – 7.33 (m, 5H), 7.46 (d, *J* = 7.9 Hz, 1H), 8.13 (d, *J* = 7.9 Hz, 1H), 8.29 (d, *J* = 9.5 Hz, 1H), 8.54 (d, *J* = 10.3 Hz, 1H).

^{13}C NMR (126 MHz, $CDCl_3$) δ 17.4, 18.7, 19.4, 19.5, 20.1, 21.0, 22.7, 23.7, 25.4, 28.5, 29.3, 29.8, 30.9, 31.5, 32.1, 32.8, 33.2, 39.1, 50.6, 51.3, 55.4, 55.8, 56.2, 57.8, 59.1, 59.4, 65.9, 75.9, 76.9, 78.4, 79.6, 107.8, 109.8, 114.5, 118.7, 119.7, 122.4, 127.6, 127.9, 128.0, 129.5, 136.9, 158.0, 168.7, 169.2, 170.2, 170.7, 171.4, 171.6, 172.2.

HRMS (ESI): calcd for $C_{52}H_{75}N_8O_{10}^+$ (M+H)$^+$: 971.5601; found: 971.5561.

(3*S*,6*S*,9*S*,12*S*,15*S*,18*S*,21*S*)-21-((*R*)-3-*H*ydroxy-2-methylpropyl)-15-((*R*)-(4-((1-(14-(4-((4-((*S*)-((2*R*,5*R*,8*R*,11*R*,14*R*,17*R*,20*R*)-17-((*S*)-3-hydroxy-2-methylpropyl)-8-isobutyl-5,11-diisopropyl-7,16,20-trimethyl-14-((1-methyl-1*H*-indol-3-yl)methyl)-3,6,9,12,15,18,21-heptaoxo-1,4,7,10,13,16,19-heptaazacyclohenicosan-2-yl)(methoxy)methyl)phenoxy)methyl)-1*H*-1,2,3-triazol-1-yl)-3,6,9,12-tetraoxatetradecyl)-1*H*-1,2,3-triazol-4-yl)methoxy)phenyl)(methoxy)methyl)-9-isobutyl-6,12-diisopropyl-1,10,18-trimethyl-3-((1-methyl-1*H*-indol-3-yl)methyl)-1,4,7,10,13,16,19-heptaazacyclohenicosane-2,5,8,11,14,17,20-heptaone (SI-39)

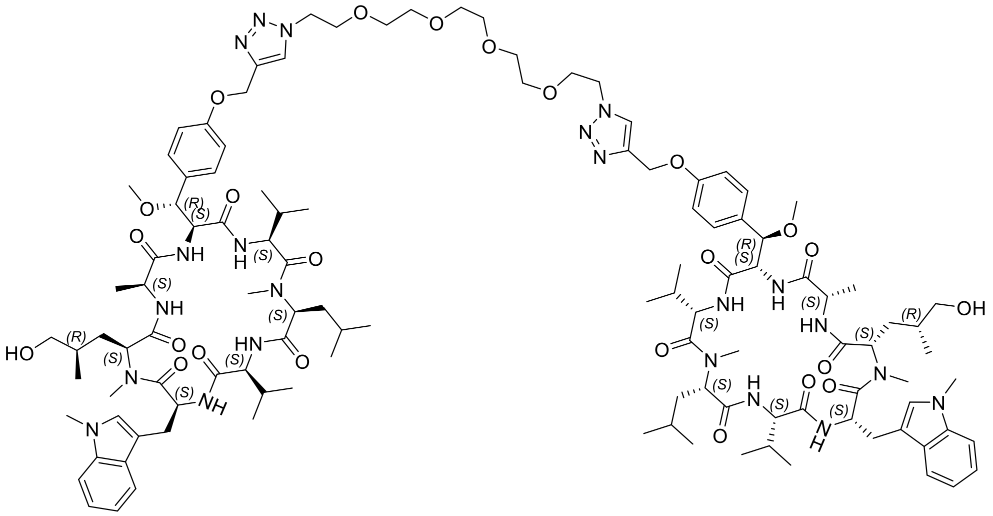

Prepared according to *GP7*: **SI-38** (22.0 mg, 23.0 µmol), 1,14-diazido-3,6,9,12-tetraoxatetradecane (3.27 mg, 11.0 µmol), 1 M $CuSO_4$ (11.3 µl, 11.0 µmol), 1 M sodium ascorbate (13.6 µl, 14.0 µmol) (17 h). RP flash chromatography (H_2O/MeCN 70:30 – 5:95) followed by lyophilization afforded **SI-39** (12.3 mg, 5.51 µmol, 48%) as a white amorphous solid.

$[\alpha]_{20}^{D}$ = –58.1 (c 0.2, $CHCl_3$).

^{1}H NMR (500 MHz, $CDCl_3$) δ -0.63 – -0.45 (m, 2H), 0.30 (d, *J* = 6.8 Hz, 6H), 0.53–0.70 (m, 11H), 0.70 – 0.89 (m, 3H), 0.91–1.03 (m, 22H), 1.07 (d, *J* = 6.6 Hz, 6H), 1.18 (d, *J* = 7.1 Hz, 6H), 1.47 – 1.73 (m, 2H), 1.79–1.92 (m, 2H), 2.14 – 2.47 (m, 24H), 2.58 (s, 6H), 2.83 (s, 6H), 3.00–3.10 (m, 2H), 3.32 (s, 6H), 3.57 (s, 6H), 3.71 (s, 6H), 3.80–4.02 (m, 4H), 4.10 (t, *J* = 9.5 Hz, 2H), 4.34 (dd, *J* = 10.7, 2.6 Hz, 2H), 4.48 (t, *J* = 8.7 Hz, 2H), 4.52–4.66 (m, 4H), 4.74–4.83 (m, 2H), 4.84 – 4.93 (m, 4H), 5.01 (d, *J* = 5.3 Hz, 2H), 5.05–5.27 (m, 3H), 6.79 – 6.95 (m, 6H), 6.99 – 7.17 (m, 7H), 7.16 – 7.23 (m, 2H), 7.27 (s, 2H), 7.46 (d, *J* = 7.8 Hz, 2H), 7.83 (s, 2H), 8.12 (d, *J* = 8.0 Hz, 2H), 8.27 (d, *J* = 9.5 Hz, 2H), 8.54 (d, *J* = 10.3 Hz, 2H).

^{13}C NMR (126 MHz, $CDCl_3$) δ 17.4, 18.8, 19.4, 19.5, 20.1, 21.1, 22.7, 23.7, 25.4, 28.5, 29.3, 29.8, 30.9, 31.5, 32.0, 32.8, 33.2, 39.1, 50.7, 51.3, 55.4, 56.2, 57.8, 59.1, 59.4, 61.8, 65.9, 69.5, 70.57, 70.65, 79.8, 107.9, 109.8, 114.4, 118.7, 119.7, 122.4, 124.4, 127.7, 127.9, 129.5, 137.0, 143.4, 158.6, 168.7, 169.3, 170.2, 170.8, 171.5, 171.6, 172.2.

HRMS (ESI): calcd for $C_{114}H_{169}N_{22}O_{24}^+$ (M+H)$^+$: 1116.1391; found: 1116.1374.

(3*S*,3'*S*,6*S*,6'*S*,9*S*,9'*S*,12*S*,12'*S*,15*S*,15'*S*,18*S*,18'*S*,21*S*,21'*S*)-15,15'-((1*R*,1'*R*)-((((Pentane-1,5-diylbis(1*H*-1,2,3-triazole-1,4-diyl))bis(methylene))bis(oxy))bis(4,1-phenylene))bis(methoxymethylene))bis(21-((*R*)-3-hydroxy-2-methylpropyl)-9-isobutyl-6,12-diisopropyl-1,10,18-trimethyl-3-((1-methyl-1*H*-indol-3-yl)methyl)-1,4,7,10,13,16,19-heptaazacyclohenicosane-2,5,8,11,14,17,20-heptaone) (SI-40)

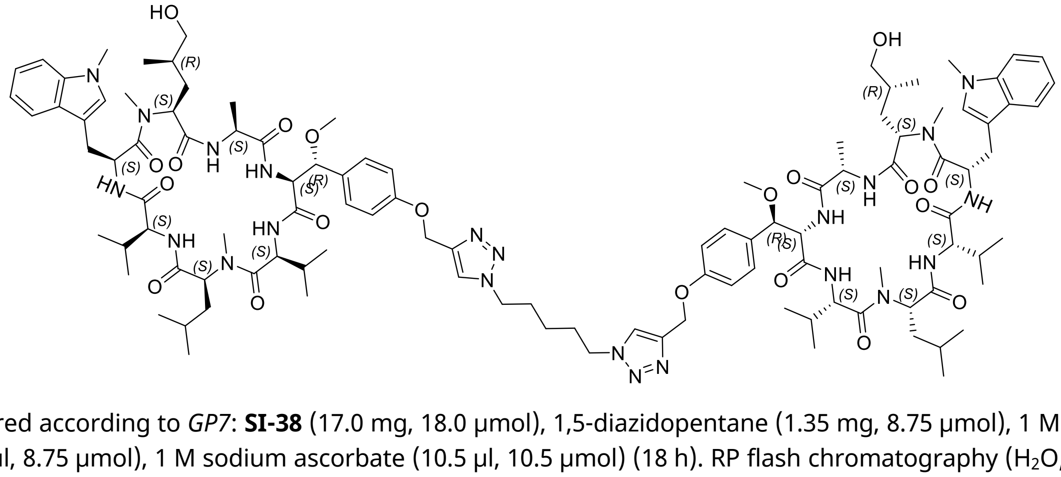

Prepared according to *GP7*: **SI-38** (17.0 mg, 18.0 µmol), 1,5-diazidopentane (1.35 mg, 8.75 µmol), 1 M $CuSO_4$ (8.75 µl, 8.75 µmol), 1 M sodium ascorbate (10.5 µl, 10.5 µmol) (18 h). RP flash chromatography (H_2O/MeCN 70:30 – 5:95) followed by lyophilization afforded **SI-40** (7.40 mg, 3.53 µmol, 40%) as a white amorphous solid.

$[\alpha]_{20}^{D}$= –65.5 (c 0.2, $CHCl_3$).

^{1}H NMR (500 MHz, $CDCl_3$) δ -0.66 – -0.47 (m, 2H), 0.30 (d, *J* = 6.7 Hz, 6H), 0.45–0.72 (m, 10H), 0.91–1.00 (m, 22H), 1.07 (d, *J* = 6.7 Hz, 6H), 1.17 (d, *J* = 7.1 Hz, 6H), 1.52–1.60 (m, 2H), 1.91–2.02 (m, 22H), 2.19 – 2.37 (m, 6H), 2.57 (s, 6H), 2.83 (s, 3H), 2.89 – 3.07 (m, 4H), 3.09 – 3.28 (m, 2H), 3.33 (s, 6H), 3.72 (s, 6H), 4.11 (t, *J* = 9.4 Hz, 2H), 4.30 – 4.42 (m, 10H), 4.48 (t, *J* = 8.6 Hz, 2H), 4.69 – 4.95 (m, 6H), 5.02 (d, *J* = 5.3 Hz, 2H), 5.13 (s, 3H), 6.83 (s, 2H), 6.85 – 6.91 (m, 4H), 7.01–7.10 (m, 1H), 7.12 (d, *J* = 8.4 Hz, 4H), 7.17 – 7.25 (m, 2H), 7.46 (d, *J* = 7.9 Hz, 2H), 8.12 (d, *J* = 8.0 Hz, 2H), 8.30 (d, *J* = 9.4 Hz, 2H), 8.53 (d, *J* = 10.3 Hz, 2H).

^{13}C NMR (126 MHz, $CDCl_3$) 17.4, 18.8, 19.5, 20.1, 21.1, 22.7, 23.5, 23.8, 25.4, 28.6, 29.3, 29.71, 29.74, 30.9, 31.5, 32.1, 32.9, 33.3, 39.1, 50.1, 50.7, 51.3, 55.4, 56.2, 57.8, 59.1, 59.4, 62.1, 65.9, 79.7, 107.8, 109.8, 114.5, 118.7, 119.7, 122.4, 122.8, 127.7, 127.8, 127.9, 129.6, 136.9, 144.1, 158.7, 168.7, 169.3, 170.2, 170.8, 171.4, 171.6, 172.2.

HRMS (ESI): calcd for $C_{109}H_{159}N_{22}O_{20}^+$ (M+H)$^+$: 1049.1101; found: 1049.1048.

Synthesis of exit vector 6 Homo-BacPROTACs via olefin metathesis (15, 16)

1-Allyl-N$^{\alpha}$-((allyloxy)carbonyl)-L-tryptophan (SI-41)

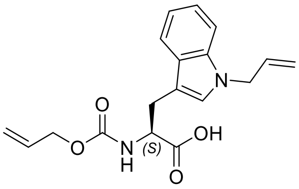

((Allyloxy)carbonyl)-L-tryptophan (400 mg, 1.39 mmol) was dissolved in DMF (2.8 ml) under N_2 and KO*t*-Bu (327 mg, 2.91 mmol) was added. After all solids were dissolved, the mixture was cooled to 0 °C and allyl bromide (168 µl, 1.94 mmol) was added in one portion. Complete conversion was observed after 5 min (TLC), the reaction was quenched by addition of 1 M HCl after 10 min. The mixture was extracted with EtOAc (2x). The combined organic phases were washed with 1 M LiCl and sat. NaCl solution, dried (Na_2SO_4) and evaporated. Flash chromatography (CyH/[EtOAc+2%HOAc] 100:0 – 70:30) followed by co-distillation with toluene and lyophilization afforded **SI-41** (336 mg, 1.02 mmol, 74%) as a yellow honey-like resin. R_f (PE/EtOAc/HOAc 70:30:1) = 0.28.

$[\alpha]_{20}^{D}$ = +33.6 (c 1.0, $CHCl_3$).

^{1}H NMR (500 MHz, DMSO-d_6) δ 2.98 (dd, *J* = 14.6, 9.7 Hz, 1H), 3.17 (dd, *J* = 14.6, 4.7 Hz, 1H), 4.20 (ddd, *J* = 9.6, 8.2, 4.7 Hz, 1H), 4.35 – 4.46 (m, 2H), 4.76 (d, *J* = 5.4 Hz, 2H), 4.93 – 5.00 (m, 1H), 5.09 – 5.16 (m, 2H), 5.23 (dq, *J* = 17.2, 1.7 Hz, 1H), 5.84 (ddt, *J* = 17.2, 10.4, 5.3 Hz, 1H), 5.96 (ddt, *J* = 17.0, 10.4, 5.3 Hz, 1H), 6.98 – 7.06 (m, 1H), 7.08 – 7.15 (m, 1H), 7.15 (s, 1H), 7.37 (d, *J* = 8.2 Hz, 1H), 7.53 (d, *J* = 8.2 Hz, 1H), 7.55 (d, *J* = 7.9 Hz, 1H), 12.69 (bs, 1H).

^{13}C NMR (126 MHz, DMSO-d_6) δ 26.80, 47.85, 54.86, 64.38, 109.96, 110.00, 116.53, 116.97, 118.49, 118.68, 121.15, 127.13, 127.59, 133.55, 134.50, 135.96, 155.84, 173.67.

HRMS (CI) calcd for $C_{18}H_{20}N_2O_4^+$ (M)$^+$: 328.1418; found: 328.1431.

methyl *N*-(((2*S*,3*R*)-2-((*S*)-2-((2*S*,4*R*)-2-((*S*)-3-(1-allyl-1*H*-indol-3-yl)-2-(((allyloxy)carbonyl)amino)-*N*-methylpropanamido)-5-((*tert*-butyldimethylsilyl)oxy)-4-methylpentanamido)propanamido)-3-methoxy-3-phenylpropanoyl)-L-valyl)-*N*-methyl-L-leucinate (SI-42)

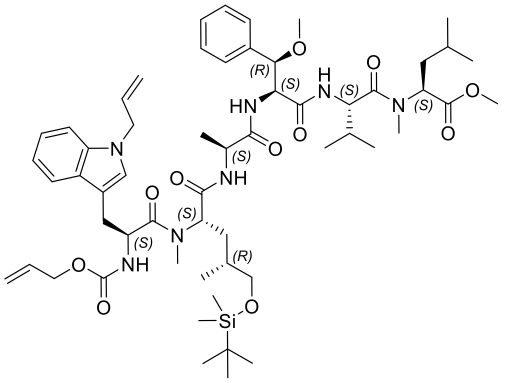

Prepared according to *GP1* and *GP5*: Pentapeptide **2** (607 mg, 676 µmol), Pd/C (71.9 mg) (1 h); **SI-41** (307 mg, 916 µmol), DIPEA (153 µl, 878 µmol), $BnNMe_2$ (10.0 µl, 6.75 µmol), 1 M iPrOCOCl (878 µl, 878 µmol), 4 M HCl (16.9 µl, 6.75 µmol), NMI (10.8 µl, 135 µmol) (16 h). Flash chromatography (CyH/EtOAc 100:0 – 70:30 – 35:65) followed by lyophilization yielded **SI-42** (606 mg, 564 µmol, 84%) as a white amorphous solid. R_f = 0.16 (PE/EtOAc 1:1).

$[\alpha]_{20}^{D}$ = −56.9 (c 1.0, $CHCl_3$).

^{1}H NMR (500 MHz, $CDCl_3$) *(mixture of rotamers, ratio ~3:1)* δ -0.33 – -0.26 (m, 0.2H), -0.05 (s, 0.7H), -0.03 (s, 0.7H), 0.00 – 0.05 (m, 4.5H), 0.41 (d, *J* = 6.6 Hz, 0.6H), 0.83 (s, 2.0H), 0.86 – 0.88 (m, 4.9H), 0.88 – 0.96 (m, 13.2H), 0.96 – 1.02 (m, 3.0H), 1.16 – 1.21 (m, 1.4H), 1.21 – 1.27 (m, 1.7H), 1.32 (d, *J* = 7.3 Hz, 0.7H), 1.38 – 1.53 (m, 2.4H), 1.66 – 1.81 (m, 2.1H), 1.84 – 1.91 (m, 0.3H), 1.92 – 2.23 (m, 2.5H), 2.25 – 2.29 (m, 0.5H), 2.73 (s, 0.6H), 2.78 (s, 1.5H), 2.81 – 2.85 (m, 0.4H), 2.97 (s, 0.8H), 2.98 – 3.05 (m, 2.3H), 3.08 – 3.24 (m, 1.4H), 3.27 (s, 0.8H), 3.32 (s, 2.1H), 3.38 – 3.47 (m, 1.3H), 3.63 – 3.71 (m, 3.0H), 4.13 – 4.21 (m, 0.4H), 4.24 – 4.31 (m, 0.5H), 4.44 – 4.58 (m, 1.6H), 4.58 – 4.74 (m, 3.2H), 4.75 – 4.90 (m, 2.7H), 4.91 – 5.06 (m, 1.2H), 5.06 – 5.30 (m, 2.8H), 5.31 – 5.38 (m, 1.0H), 5.67 (d, *J* = 7.6 Hz, 0.2H), 5.77 – 5.99 (m, 1.9H), 6.41 (d, *J* = 6.8 Hz, 0.4H), 6.75 (d, *J* = 7.6 Hz, 0.3H), 6.80 (t, *J* = 7.6 Hz, 0.5H), 6.90 – 6.97 (m, 0.8H), 7.06 – 7.14 (m, 1.0H), 7.15 – 7.31 (m, 7.2H), 7.35 (d, *J* = 8.8 Hz, 0.3H), 7.46 (d, *J* = 8.8 Hz, 0.4H), 7.57 (d, *J* = 7.9 Hz, 0.3H), 7.64 (d, *J* = 7.9 Hz, 0.4H), 7.87 (d, *J* = 6.6 Hz, 0.2H).

^{13}C NMR (126 MHz, $CDCl_3$) *(mixture of rotamers)* δ -5.29, -5.26, 14.3, 15.6, 17.2, 17.3, 17.5, 17.6, 17.9, 18.4, 19.57, 19.65, 21.5, 23.4, 24.9, 26.0, 26.1, 28.5, 28.8, 31.3, 31.4, 31.6, 32.1, 32.2, 37.0, 39.7, 48.8, 48.9, 49.6, 49.8, 49.9, 50.9, 51.7, 52.3, 54.2, 54.3, 54.5, 54.6, 56.4, 57.6, 57.7, 57.86, 57.94, 65.1, 65.8, 66.4, 67.4, 68.4, 81.2, 81.5, 108.8, 109.1, 109.86, 109.93, 117.3, 117.7, 117.8, 118.4, 118.8, 119.5, 119.7, 122.0, 122.2, 126.77, 126.84, 126.94, 127.01, 128.2, 128.26, 128.34, 128.4, 128.6, 132.2, 132.8, 133.4, 133.5, 136.3, 136.4, 136.9, 156.0, 156.7, 168.6, 168.8, 170.5, 171.7, 172.0, 172.18, 172.23, 172.6, 173.4.

Selected diagnostic peaks:

Major rotamer: **^{1}H NMR** (500 MHz, $CDCl_3$) δ 0.01 (s, 3H), 0.03 (s, 3H), 0.87 (s, 9H), 2.78 (s, 3H), 3.00 (s, 3H), 3.32 (s, 3H), 3.68 (s, 3H), 5.36 (ddd, *J* = 10.7, 5.3, 2.3 Hz, 2H), 6.93 (s, 1H), 7.46 (d, *J* = 8.8 Hz, 1H), 7.64 (d, *J* = 7.9 Hz, 1H). **^{13}C NMR** (126 MHz, $CDCl_3$) δ 26.1, 31.4, 81.2, 156.0

Minor rotamer: **^{1}H NMR** (500 MHz, $CDCl_3$) δ 0.41 (d, *J* = 6.6 Hz, 3H), 0.83 (s, 9H), 1.32 (d, *J* = 7.3 Hz, 3H), 2.73 (s, 3H), 2.97 (s, 3H), 3.27 (s, 3H), 5.67 (d, *J* = 7.6 Hz, 1H), 6.75 (d, *J* = 7.6 Hz, 1H), 6.92 (s, 1H), 7.35 (d, *J* = 8.8 Hz, 1H), 7.57 (d, *J* = 7.9 Hz, 1H), 7.87 (d, *J* = 6.6 Hz, 1H). **^{13}C NMR** (126 MHz, $CDCl_3$) δ 26.0, 31.3, 81.5, 156.7.

HRMS (ESI) calcd for $C_{57}H_{88}N_7O_{11}Si^+$ $(M+H)^+$: 1074.6306; found: 1074.6287.

methyl *N*-(((2*S*,3*R*)-2-((*S*)-2-((2*S*,4*R*)-2-((*S*)-3-(1-allyl-1*H*-indol-3-yl)-2-((*S*)-2-(((allyloxy)carbonyl)amino)-3-methylbutanamido)-*N*-methylpropanamido)-5-((*tert*-butyldimethylsilyl)oxy)-4-methylpentanamido)propanamido)-3-methoxy-3-phenylpropanoyl)-L-valyl)-*N*-methyl-L-leucinate (SI-43)

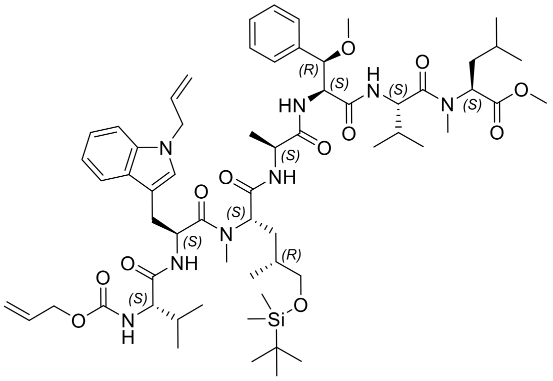

Prepared according to *GP2* and *GP4*: **SI-42** (571 mg, 531 µmol), DMBA (249 mg, 1.59 mmol), $Pd(PPh_3)_4$ (18.4 mg, 15.9 µmol) (1.5 h); Alloc-L-valine (123 mg, 611 µmol), HOBt (89.0 mg, 584 µmol), EDC (112 mg, 584 µmol), NMM (117 µl, 1.06 mmol) (16 h). Flash chromatography (CyH/EtOAc 100:0 – 30:70) followed by lyophilization yielded **SI-43** (525 mg, 447 mmol, 84%) as a white amorphous solid. R_f = 0.26 (PE/EtOAc 3:7).

$[\alpha]^{D}_{20}$ = −66.6 (c 1.0, $CHCl_3$).

^{1}H NMR (500 MHz, $CDCl_3$) *(mixture of rotamers, ratio ~2:1)* δ −0.45 – -0.29 (m, 0.1H), -0.05 (s, 0.9H), -0.04 (s, 1.0H), -0.01 – 0.06 (m, 3.8H), 0.38 (d, *J* = 6.8 Hz, 0.6H), 0.79 – 0.83 (m, 4.7H), 0.84 – 0.87 (m, 6.1H), 0.87 – 0.96 (m, 15.1H), 0.96 – 1.01 (m, 3.1H), 1.18 – 1.22 (m, 2.0H), 1.25 (d, *J* = 6.3 Hz, 0.6H), 1.30 – 1.35 (m, 0.7H), 1.40 – 1.53 (m, 2.1H), 1.65 – 1.80 (m, 2.2H), 1.80 – 1.88 (m, 0.3H), 1.89 – 2.25 (m, 4.0H), 2.67 – 2.72 (m, 0.9H), 2.73 – 2.78 (m, 0.4H), 2.80 (m, 1.4H), 2.95 – 3.00 (m, 3.0H), 3.00 – 3.05 (m, 0.3H), 3.06 – 3.17 (m, 0.9H), 3.18 – 3.24 (m, 0.6H), 3.28 (s, 1.0H), 3.30 (s, 0.5H), 3.32 (s, 1.8H), 3.39 – 3.43 (m, 0.9H), 3.65 – 3.72 (s, 3.0H), 4.02 – 4.13 (m, 0.7H), 4.22 – 4.29 (m, 0.5H), 4.31 – 4.39 (m, 0.4H), 4.45 – 4.76 (m, 5.3H), 4.77 – 4.87 (m, 2.1H), 4.89 – 4.98 (m, 0.8H), 498 – 5.05 (m, 0.6H), 5.09 – 5.26 (m, 2.7H), 5.26 – 5.38 (m, 2.2H), 5.59 (d, *J* = 9.0 Hz, 0.1H), 5.71 (d, *J* = 9.5 Hz, 0.2H), 5.83 – 5.98 (m, 1.7H), 6.53 (d, *J* = 6.1 Hz, 0.3H), 6.74 – 6.86 (m, 1.0H), 6.89 – 6.96 (m, 0.9H), 6.97 – 7.04 (m, 0.4H), 7.06 – 7.13 (m, 1.0H), 7.14 – 7.32 (m, 7.8H), 7.33 – 7.42 (m, 0.8H), 7.42 – 7.48 (m, 0.5H), 7.51 – 7.57 (m, 0.5H), 7.62 – 7.69 (m, 0.8H), 8.14 (d, *J* = 7.1 Hz, 0.2H).

^{13}C NMR (126 MHz, $CDCl_3$) *(mixture of rotamers)* δ 15.7, 17.37, 17.44, 17.7, 18.0, 18.1, 18.4, 18.5, 19.18, 19.24, 19.60, 19.64, 21.48, 21.52, 23.4, 24.9, 26.0, 26.1, 28.1, 28.7, 29.2, 31.3, 31.4, 31.5, 32.3, 37.0, 48.8, 48.9, 49.4, 49.8, 50.1, 52.3, 54.2, 54.5, 54.6, 57.5, 57.6, 58.0, 58.1, 60.0, 65.9, 66.1, 67.1, 68.5, 81.2, 81.4, 108.7, 109.0, 109.9, 110.0, 117.3, 117.9, 118.0, 118.8, 119.6, 122.0, 122.2, 126.8, 127.0, 127.1, 127.9, 128.2, 128.3, 128.4, 128.5, 128.6, 128.7, 132.1, 132.17, 132.25, 132.8, 133.4, 133.6, 136.3, 136.4, 136.9, 156.2, 156.4, 168.5, 170.4, 170.9, 171.6, 171.8, 171.9, 172.1, 172.2, 172.5, 172.9.

Selected diagnostic peaks:

Major rotamer: **^{1}H NMR** (500 MHz, $CDCl_3$) δ 0.00 (s, 3H), 0.01 (s, 3H), 0.86 (s, 9H), 2.80 (s, 3H), 2.97 (s, 3H), 3.32 (s, 3H), 3.68 (s, 3H), 5.71 (d, *J* = 9.5 Hz, 0H), 6.53 (d, *J* = 6.1 Hz, 1H), 6.93 (s, 1H). **^{13}C NMR** (126 MHz, $CDCl_3$) δ 26.1, 57.6, 81.4, 156.2.

Minor rotamer: **^{1}H NMR** (500 MHz, $CDCl_3$) δ -0.05 (s, 3H), -0.04 (s, 3H), 0.38 (d, *J* = 6.8 Hz, 3H), 0.83 (s, 9H), 2.69 (s, 3H), 3.28 (s, 3H), 3.69 (s, 3H), 4.35 (t, *J* = 7.3 Hz, 1H), 5.59 (d, *J* = 9.0 Hz, 1H), 6.91 (s, 1H), 8.14 (d, *J* = 7.1 Hz, 1H). **^{13}C NMR** (126 MHz, $CDCl_3$) δ 26.0, 57.5, 81.2, 156.4.

HRMS (ESI) calcd for $C_{62}H_{97}N_8O_{12}Si^+$ $(M+H)^+$: 1173.6990; found: 1173.6974.

(3*S*,6*S*,9*S*,12*S*,15*S*,18*S*,21*S*)-3-((1-allyl-1*H*-indol-3-yl)methyl)-21-((*R*)-3-hydroxy-2-methylpropyl)-9-isobutyl-6,12-diisopropyl-15-((*R*)-methoxy(phenyl)methyl)-1,10,18-trimethyl-1,4,7,10,13,16,19-heptaazacyclohenicosane-2,5,8,11,14,17,20-heptaone (13)

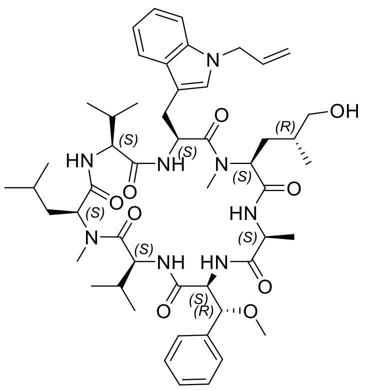

Prepared according to *GP6a*: **SI-43** (134 mg, 114 µmol), 1 M LiOH (160 µl, 160 µmol) (17 h); $Pd(OAc)_2$ (1.3 mg, 5.7 µmol), TPPTS (6.5 mg, 11.4 µmol), Et_2NH (56.3 µl, 570 µmol) (2.5 h); HATU (130 mg, 342 µmol), DIPEA (80.7 µl, 456 µmol) (addition over 1.5 h, additional 16 h); NH_4F (84.5 mg, 2.28 mmol) (16 h). Reversed phase flash chromatography (H_2O/MeCN 90:10 – 5:95) followed by lyophilization yielded **13** (68.6 mg, 72.7 µmol, 64%) as a white amorphous solid.

$[\alpha]_{24}^{D}$ = −82.2 (c 0.5, $CHCl_3$).

^{1}H NMR (500 MHz, $CDCl_3$) δ -0.60 – -0.52 (m, 1H), 0.24 (d, *J* = 6.8 Hz, 3H), 0.58 (d, *J* = 6.5 Hz, 3H), 0.61 (d, *J* = 6.6 Hz, 3H), 0.73 (d, *J* = 6.8 Hz, 1H), 0.81 (q, *J* = 5.2 Hz, 1H), 0.93 – 0.99 (m, 9H), 1.07 (d, *J* = 6.6 Hz, 3H), 1.09 – 1.12 (m, 1H), 1.14 (d, *J* = 7.3 Hz, 3H), 1.52 – 1.61 (m, 1H), 1.84 (ddd, *J* = 13.6, 11.0, 7.1 Hz, 1H), 2.18 – 2.35 (m, 3H), 2.57 (s, 3H), 2.82 (s, 3H), 2.92 (dd, *J* = 11.2, 5.5 Hz, 1H), 3.01 (dd, *J* = 11.2, 4.3 Hz, 1H), 3.11 (dd, *J* = 13.6, 4.9 Hz, 1H), 3.25 – 3.32 (m, 1H), 3.34 (s, 3H), 4.10 (t, *J* = 9.5 Hz, 1H), 4.36 (dd, *J* = 11.0, 2.6 Hz, 1H), 4.47 (t, *J* = 8.7 Hz, 1H), 4.58 – 4.66 (m, 2H), 4.72 – 4.78 (m, 2H), 4.85 (dd, *J* = 10.4, 3.8 Hz, 1H), 4.90 (t, *J* = 5.1 Hz, 1H), 5.06 (d, *J* = 5.5 Hz, 1H), 5.10 – 5.17 (m, 1H), 5.17 – 5.22 (m, 1H), 5.90 (ddt, *J* = 17.2, 10.2, 5.8 Hz, 1H), 6.86 (s, 1H), 7.04 – 7.11 (m, 1H), 7.14 – 7.18 (m, 3H), 7.19 – 7.24 (m, 4H), 7.26 – 7.28 (m, 1H), 7.42 (d, *J* = 5.2 Hz, 1H), 7.46 (d, *J* = 7.9 Hz, 1H), 8.11 (d, *J* = 7.9 Hz, 1H), 8.25 (d, *J* = 9.5 Hz, 1H), 8.51 (d, *J* = 10.4 Hz, 1H).

^{13}C NMR (126 MHz, $CDCl_3$) δ 17.2, 18.7, 19.4, 20.10, 20.13, 20.9, 22.7, 23.7, 25.3, 28.3, 29.3, 29.7, 30.9, 31.6, 31.9, 33.1, 39.0, 48.9, 50.5, 51.1, 55.3, 56.0, 57.9, 58.9, 59.0, 59.2, 66.0, 80.0, 108.2, 110.1, 118.1, 118.6, 119.8, 122.4, 126.8, 127.8, 128.1, 128.2, 128.8, 133.2, 135.1, 136.3, 168.6, 169.1, 170.0, 170.7, 171.5, 171.6, 172.2.

HRMS (ESI) calcd for $C_{51}H_{75}N_8O_9^+$ (M+H)$^+$: 943.5652; found: 943.5617.

(3*S*,3'*S*,6*S*,6'*S*,9*S*,9'*S*,12*S*,12'*S*,15*S*,15'*S*,18*S*,18'*S*,21*S*,21'*S*)-15,15'-((((*E*)-but-2-ene-1,4-diyl)bis(1*H*-indole-1,3-diyl))bis(methylene))bis(12-((*R*)-3-hydroxy-2-methylpropyl)-21-isobutyl-3,18-diisopropyl-6-((*R*)-methoxy(phenyl)methyl)-1,9,13-trimethyl-1',4',7',10',13',16',19'-heptaazacyclohenicosane-2,5,8,11,14,17,20-heptaone) (15)

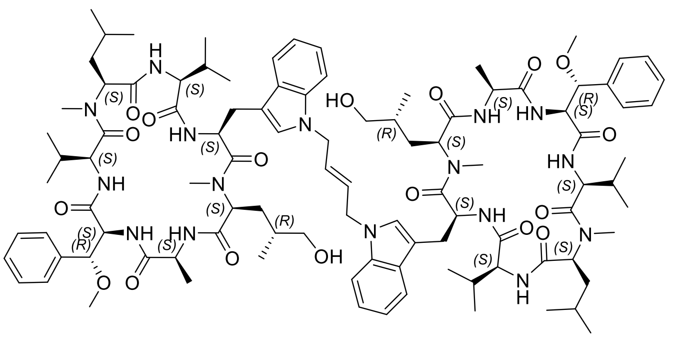

A 4 ml vial was charged with **13** (23.6 mg, 25.0 µmol) and Grubbs catalyst 2nd Gen (2.1 mg, 2.5 µmol), freshly degassed (3x freeze-pump-thaw) DCM (250 µl) was added, the vial was flushed with Argon and sealed. The mixture was heated to 40 °C. After 16 h, additional Grubbs catalyst 2nd Gen (2.1 mg, 2.5 µmol) was added. After 41 h, LC/MS indicated ca. 50% conversion (and formation of side products). The reaction mixture was evaporated in vacuo. RP flash chromatography (H_2O/MeCN 90:10 – 5:95) followed by prep HPLC (H_2O/MeCN 70:30 – 0:100) and lyophilization yielded **15** (6.7 mg, 3.6 µmol, 29%) as a white amorphous solid.

$[\alpha]_{20}^{\mathrm{D}}$ = −121.7 (c 0.2, $CHCl_3$).

^{1}H NMR (500 MHz, $CDCl_3$) δ -0.37 – -0.23 (m, 2H), 0.36 (d, *J* = 6.8 Hz, 6H), 0.53 (d, *J* = 6.1 Hz, 6H), 0.62 (d, *J* = 6.3 Hz, 6H), 0.67 (s, 2H), 0.91 – 0.97 (m, 18H), 1.01 – 1.08 (m, 8H), 1.15 – 1.23 (m, 8H), 1.48 – 1.55 (m, 2H), 1.98 – 2.08 (m, 2H), 2.16 – 2.26 (m, 2H), 2.27 – 2.34 (m, 2H), 2.47 (s, 6H), 2.76 – 2.84 (m, 8H), 2.91 – 2.98 (m, 2H), 3.03 (dd, *J* = 14.1, 6.7 Hz, 2H), 3.08 – 3.16 (m, 4H), 3.33 (s, 6H), 4.03 (t, *J* = 9.3 Hz, 2H), 4.40 (t, *J* = 8.7 Hz, 2H), 4.49 (dd, *J* = 11.2, 3.6 Hz, 2H), 4.54 – 4.62 (m, 4H), 4.68 (d, *J* = 13.2 Hz, 2H), 4.82 (dd, *J* = 10.6, 3.4 Hz, 2H), 4.85 – 4.90 (m, 4H), 5.04 (d, *J* = 5.5 Hz, 2H), 5.72 (t, *J* = 3.2 Hz, 2H), 6.89 (s, 2H), 7.04 – 7.10 (m, 2H), 7.11 (d, *J* = 4.7 Hz, 2H), 7.14 – 7.17 (m, 4H), 7.18 (d, *J* = 7.7 Hz, 2H), 7.20 – 7.22 (m, 6H), 7.26 (d, *J* = 8.2 Hz, 2H), 7.32 (d, *J* = 5.0 Hz, 2H), 7.42 (d, *J* = 7.9 Hz, 2H), 8.04 (d, *J* = 7.9 Hz, 2H), 8.25 (d, *J* = 9.5 Hz, 2H), 8.35 (d, *J* = 10.4 Hz, 2H).

^{13}C NMR (126 MHz, $CDCl_3$) δ 17.0, 18.8, 19.5, 20.1, 21.4, 22.7, 23.7, 25.5, 27.6, 28.9, 29.7, 31.0, 32.0, 32.2, 33.1, 39.0, 47.4, 50.6, 51.1, 55.5, 56.0, 57.9, 59.0, 59.1, 66.8, 80.0, 108.7, 109.9, 118.4, 119.8, 122.4, 126.9, 127.9, 128.2, 128.3, 128.5, 128.8, 135.1, 135.7, 168.8, 169.0, 169.9, 170.7, 171.5, 171.6, 172.5.

HRMS (ESI) calcd for $C_{100}H_{145}N_{16}O_{18}^+$ (M+H)$^+$: 1858.0917; found: 1858.0987.

(3*S*,3'*S*,6*S*,6'*S*,9*S*,9'*S*,12*S*,12'*S*,15*S*,15'*S*,18*S*,18'*S*,21*S*,21'*S*)-15,15'-((butane-1,4-diylbis(1*H*-indole-1,3-diyl))bis(methylene))bis(12-((*R*)-3-hydroxy-2-methylpropyl)-21-isobutyl-3,18-diisopropyl-6-((*R*)-methoxy(phenyl)methyl)-1,9,13-trimethyl-1',4',7',10',13',16',19'-heptaazacyclohenicosane-2,5,8,11,14,17,20-heptaone) (16)

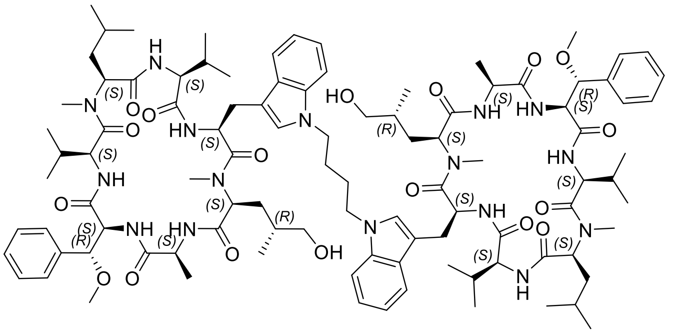

Under Ar, **13** (21.1 mg, 22.4 µmol) was dissolved in freshly degassed (3x freeze-pump-thaw) DCE (2 ml) and the mixture was heated to 60 °C. A solution of Grubbs catalyst 2nd Gen (5.7 mg, 6.7 mg) in DCE (1 ml) was added over the course of 12 h. After 18 h, LC/MS indicated 80% conversion. The mixture was evaporated in vacuo and the residue was dissolved in MeOH (4 ml). Pd/C (5 mg) was added and the mixture was shaken under H_2 (4 bar) for 16 h. RP flash chromatography (H_2O/MeCN 90:10 – 5:95) followed by prep HPLC (H_2O/MeCN 90:10 – 0:100) and lyophilization yielded **16** (4.8 mg, 2.58 µmol, 23%) as a white amorphous solid.

$[\alpha]_{20}^{\mathrm{D}}$ = −155.6 (c 0.2, $CHCl_3$).

^{1}H NMR (500 MHz, $CDCl_3$) δ -0.37 – -0.27 (m, 2H), 0.34 (d, *J* = 6.8 Hz, 6H), 0.56 (d, *J* = 6.5 Hz, 6H), 0.63 (d, *J* = 6.5 Hz, 6H), 0.67 – 0.72 (m, 2H), 0.93 – 0.98 (m, 18H), 1.05 – 1.11 (m, 10H), 1.21 (d, *J* = 7.3 Hz, 6H), 1.50 – 1.57 (m, 2H), 1.69 – 1.82 (m, 6H), 1.99 – 2.08 (m, 2H), 2.21 – 2.27 (m, 2H), 2.28 – 2.35 (m, 2H), 2.52 (s, 6H), 2.78 – 2.86 (m, 8H), 3.05 (dd, *J* = 14.0, 6.5 Hz, 2H), 3.10 (dd, *J* = 11.2, 4.3 Hz, 2H), 3.14 – 3.20 (m, 2H), 3.36 (s, 6H), 3.88 – 3.96 (m, 2H), 4.02 – 4.14 (m, 4H), 4.42 (t, *J* = 8.7 Hz, 2H), 4.45 – 4.50 (m, 2H), 4.60 – 4.66 (m, 2H), 4.80 – 4.88 (m, 4H), 4.90 (t, *J* = 5.0 Hz, 2H), 5.06 (d, *J* = 5.5 Hz, 2H), 6.85 (s, 2H), 7.06 – 7.11 (m, 2H), 7.14 (d, *J* = 4.9 Hz, 2H), 7.17 – 7.21 (m, 8H), 7.21 – 7.25 (m, 8H), 7.44 (d, *J* = 7.9 Hz, 2H), 8.06 (d, *J* = 7.9 Hz, 2H), 8.24 (d, *J* = 9.5 Hz, 2H), 8.38 (d, *J* = 10.1 Hz, 2H).

^{13}C NMR (126 MHz, $CDCl_3$) δ 17.0, 18.7, 19.5, 20.1, 21.2, 22.7, 23.7, 25.5, 27.8, 28.3, 29.1, 29.7, 30.9, 32.0, 32.3, 33.0, 39.0, 46.1, 50.6, 51.1, 55.4, 56.0, 57.9, 59.05, 59.06, 66.6, 80.0, 108.3, 109.8, 118.5, 119.7, 122.4, 127.0, 127.8, 128.2, 128.3, 128.8, 135.1, 135.8, 168.8, 169.0, 170.0, 170.7, 171.5, 171.6, 172.5.

HRMS (ESI) calcd for $C_{100}H_{147}N_{16}O_{18}^+$ (M+H)$^+$: 1860.1074; found: 1860.1062.

Synthesis of exit vector 3 Homo-BacPROTAC via olefin metathesis (17)

tert-butyl *N*-[(1*R*,2*R*)-1-(4-aminophenyl)-3-[(*tert*-butyldimethylsilyl)oxy]-1-hydroxypropan-2-yl]carbamate (SI-44)

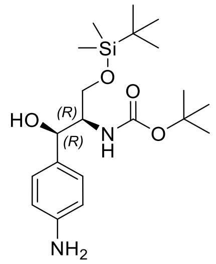

To a solution of *tert*-butyl ((1*R*,2*R*)-3-((*tert*-butyldimethylsilyl)oxy)-1-hydroxy-1-(4-nitrophenyl)propan-2-yl)carbamate[12] (25.0 g, 58.6 mmol) in THF (150 ml, 0.39 M) was added 10 wt% Pd/C (6.21 g, 5.86 mmol). The mixture was stirred at rt under H_2 atmosphere (balloon pressure). After 4 h, TLC indicated complete conversion. Subsequently, the mixture was filtered over Celite and the filtrate was evaporated in vacuo. Silica gel column chromatography (20% EtOAc in petroleum ether) yielded **SI-44** (18.0 g, 45.4 mmol, 77%).

^{1}H NMR (500 MHz, DMSO-d_6) δ 0.01 (s, 6H), 0.86 (s, 9H), 1.21 (br s, 1.35H, rotamer), 1.32 (s, 7.65H, rotamer), 3.52 – 3.46 (m, 1H), 3.55 (dd, *J* = 9.5, 6.6 Hz, 1H), 4.50 (t, *J* = 4.7 Hz, 1H), 4.90 (s, 2H), 4.94 (d, *J* = 5.4 Hz, 1H), 5.54 (d, *J* = 8.5 Hz, 0.15H, rotamer), 5.96 (d, *J* = 8.5 Hz, 0.85H, rotamer), 6.49 (d, *J* = 8.5 Hz, 2H), 6.92 (d, *J* = 8.5 Hz, 2H).

^{13}C NMR (126 MHz, DMSO-d_6) δ -5.4, -5.3, 17.9, 25.8, 27.9, 28.2, 57.9, 62.3, 62.9, 70.0, 77.6, 113.4, 126.6, 126.8, 130.4, 147.5, 155.3.

MS calcd for $C_{16}H_{27}N_2O_3Si^+$ (M – C_4H_9 – OH + H)$^+$: 323.18; found: 323.45.

tert-butyl *N*-[(1*R*,2*R*)-1-(4-aminophenyl)-3-[(tert-butyldimethylsilyl)oxy]-1-(prop-2-en-1-yloxy)propan-2-yl]carbamate (SI-45)

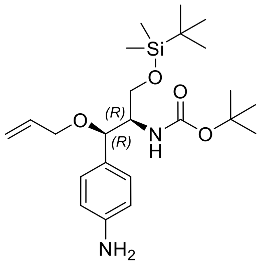

To a stirred solution of **SI-44** (15.0 g, 37.8 mmol) in DMF (120 ml, 0.32 M) was added LiHMDS (1 M in THF) (37.8 ml, 37.8 mmol) at –20 °C. Then, allyl bromide (5.03 g, 41.6 mmol) was added and the mixture was stirred at rt. After 2 h, TLC indicated complete conversion. The reaction was quenched with water and extracted with EtOAc (2x). The combined organic layers were washed with brine, dried (Na_2SO_4) and concentrated in vacuo. Silica gel column chromatography (20% EtOAc in petroleum ether) yielded **SI-45** (10.0 g, 22.9 mmol, 61%).

^{1}H NMR (500 MHz, DMSO-d_6) δ -0.03 (d, *J* = 4.1 Hz, 6H), 0.84 (s, 9H), 1.23 (br s, 1.8H, rotamer), 1.35 (s, 7.2H, rotamer), 3.26 (dd, *J* = 9.6, 5.8 Hz, 1H), 3.56 – 3.44 (m, 2H), 3.70 – 3.61 (m, 1H), 3.76 (dd, *J* = 13.6, 4.4 Hz, 1H), 4.21 (d, *J* = 6.3 Hz, 1H), 5.01 (s, 2H), 5.06 (d, *J* = 10.4 Hz, 1H), 5.28 – 5.17 (m, 1H), 5.85 – 5.75 (m, 1H), 5.87 (d, *J* = 7.3 Hz, 0.2H, rotamer), 6.25 (d, *J* = 8.8 Hz, 0.8H, rotamer), 6.51 (d, *J* = 8.5 Hz, 2H), 6.88 (d, *J* = 8.2 Hz, 2H).

^{13}C NMR (126 MHz, DMSO-d_6) δ -5.4, 25.8, 27.9, 28.3, 57.5, 58.9, 62.1, 62.5, 68.4, 77.4, 78.9, 113.6, 115.6, 126.1, 127.6, 127.8, 135.5, 148.2, 155.3**.**

MS calcd for $C_{23}H_{41}N_2O_4Si^+$ (M+H)$^+$: 437.28; found: 437.27.

tert-butyl *N*-[(1*R*,2*R*)-3-hydroxy-1-phenyl-1-(prop-2-en-1-yloxy)propan-2-yl]carbamate (SI-46)

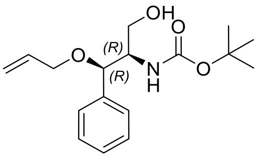

SI-45 (8.00 g, 18.3 mmol) was taken up in water (80.0 ml, 0.23 M) and cooled to 0°C. Then 15 wt% hypophosphorous acid solution (120 ml, 275 mmol) and sodium nitrite (12.6 g, 183 mmol), dissolved in water, were added. The mixture was stirred at rt. After 3 h, TLC indicated complete conversion. The reaction was basified with aq. Na_2CO_3 and extracted with EtOAc (2x). The combined organic layers were dried (Na_2SO_4) and concentrated in vacuo. Silica gel column chromatography (50% EtOAc in petroleum ether) yielded **SI-46** (4.00 g, 13.0 mmol, 71%).

^{1}H NMR (500 MHz, DMSO-d$_6$) δ 1.13 (br s, 1.8H, rotamer), 1.31 (s, 7.2H, rotamer), 3.17 (dt, *J* = 10.6, 5.4 Hz, 1H), 3.79 – 3.72 (m, 1H), 3.67 – 3.56 (m, 1H), 3.89 – 3.79 (m, 1H), 4.49 (d, *J* = 4.7 Hz, 1H), 4.64 (t, *J* = 5.2 Hz, 0.8H, rotamer), 4.71 (br s, 0.2H, rotamer), 5.17 – 5.03 (m, 1H), 5.27 (dd, *J* = 17.3, 1.3 Hz, 1H), 5.94 – 5.75 (m, 1H, 0.2H rotamer), 6.25 (d, *J* = 9.1 Hz, 0.8H, rotamer), 7.29 – 7.22 (m, 3H), 7.36 – 7.30 (m, 2H).

^{13}C NMR (126 MHz, DMSO-d$_6$) δ 27.8, 28.2, 57.4, 60.5, 69.2, 77.5, 78.8, 79.0, 115.9, 126.9, 127.4, 128.1, 135.2, 139.9, 155.3.

MS calcd for $C_{17}H_{26}NO_4^+$ (M+H)$^+$: 308.19; found: 308.24.

(2*S*,3*R*)-2-{[(*tert*-butoxy)carbonyl]amino}-3-phenyl-3-(prop-2-en-1-yloxy)propanoic acid (SI-47)

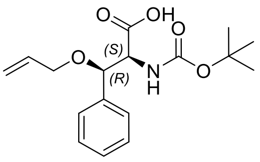

A stirred solution of **SI-46** (5.00 g, 16.3 mmol) in MeCN (20.0 ml) and 2 M NaH_2PO_4 buffer (20.0 ml) (0.40 M) was cooled to 0 °C and (diacetoxyiodo)benzene (524 mg, 1.63 mmol), TEMPO (514 mg, 3.25 mmol) and $NaClO_2$ (5.86 g, 65.1 mmol) were added. The mixture was stirred at rt for 12 h. The reaction was quenched with 2 M aq. Na_2CO_3 and washed with diethyl ether. The aqueous layer was acidified with 1 M aq. HCl and extracted with EtOAc (3x). The combined EtOAc layers were dried (Na_2SO_4) and concentrated in vacuo to yield crude **SI-47** (3.50 g, 10.9 mmol, 67%) which was used in the next step without further purification.

$[\alpha]_{20}^{D}$ = –12.7 (c 1.0, $CHCl_3$).

Major rotamer **^{1}H NMR** (500 MHz, $CDCl_3$) δ 1.33 (s, 9H), 3.84 (dd, *J* = 12.7, 6.1 Hz, 1H), 4.05 (dd, *J* = 12.7, 5.0 Hz, 1H), 4.58 (dd, *J* = 9.4, 2.9 Hz, 1H), 5.05 (d, *J* = 2.8 Hz, 1H), 5.15 – 5.25 (m, 2H), 5.34 (d, *J* = 9.4 Hz, 1H), 5.80 – 5.90 (m, 1H), 7.29 – 7.39 (m, 5H), 9.89 (bs, 1H). **^{13}C NMR** (126 MHz, $CDCl_3$) δ 28.3, 59.2, 70.4, 79.9, 80.2, 117.8, 127.0, 128.4, 128.6, 133.9, 137.2, 155.7, 175.2.

Minor rotamer (selected signals, ratio ~4:1) **^{1}H NMR** (500 MHz, $CDCl_3$) δ 1.17 (s, 9H), 4.37 – 4.43 (m, 1H), 4.94 – 4.98 (m, 1H), 5.71 – 5.79 (m, 1H). **^{13}C NMR** (126 MHz, $CDCl_3$) δ 28.0, 60.8, 70.1, 80.9, 117.6, 127.2, 137.5, 155.4.

HRMS (ESI) calcd for $C_{17}H_{23}NO_5Na^+$ (M+Na)$^+$: 344.1468; found: 344.1457.

Methyl (2*S*)-2-[(2*S*)-2-[(2*S*,3*R*)-2-{[(*tert*-butoxy)carbonyl]amino}-3-phenyl-3-(prop-2-en-1-yloxy)propanamido]-N,3-dimethylbutanamido]-4-methylpentanoate (SI-48)

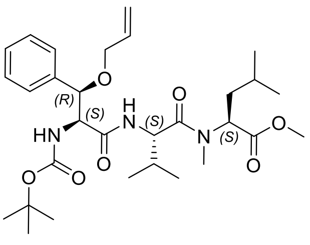

To a stirred solution of methyl N-(L-valyl)-N-methyl-L-leucinate[4] (3.00 g, 11.6 mmol) and **SI-47** (5.60 g, 17.4 mmol) in DMF (9.0 ml, 1.9 M) was added DIPEA (4.06 ml, 23.2 mmol) at 0°C. After 10 minutes, HATU (4.86 g, 12.8 mmol) was added and the mixture was stirred at rt. After complete conversion, the reaction was quenched with water and extracted with EtOAc (2x). The combined EtOAc layers were washed with brine, dried (Na_2SO_4) and concentrated in vacuo. Silica gel column chromatography (50% EtOAc in petroleum ether) yielded **SI-48** (3.00 g, 5.34 mmol, 46%).

Major rotamer: **^{1}H NMR** (500 MHz, $CDCl_3$) δ 0.89 (d, *J* = 6.5 Hz, 3H), 0.92 – 0.95 (m, 6H), 1.01 (d, *J* = 6.8 Hz, 3H), 1.31 (s, 9H), 1.41 – 1.51 (m, 1H), 1.65 – 1.80 (m, 2H), 2.07 – 2.16 (m, 1H), 3.01 (s, 3H), 3.69 (s, 3H), 3.86 (dd, *J* = 12.4, 6.1 Hz, 1H), 4.01 (dd, *J* = 12.3, 5.4 Hz, 1H), 4.42 (dd, *J* = 8.6, 2.9 Hz, 1H), 4.86 (dd, *J* = 8.9, 6.4 Hz, 1H), 5.07 – 5.12 (m, 1H), 5.13 – 5.17 (m, 1H), 5.19 – 5.23 (m, 1H), 5.26 (d, *J* = 8.6 Hz, 1H), 5.32 (dd, *J* = 10.5, 5.2 Hz, 1H), 5.81 – 5.92 (m, 1H), 7.25 – 7.33 (m, 6H). **^{13}C NMR** (126 MHz, $CDCl_3$) δ 17.5, 19.6, 21.5, 23.3, 25.0, 28.3, 31.5, 31.8, 37.1, 52.3, 54.1, 54.6, 60.1, 70.7, 79.6, 80.3, 117.5, 126.8, 128.1, 128.5, 134.2, 137.7, 155.5, 169.4, 172.2, 172.3.

Minor rotamer (selected signals, ratio ~ 12:1): **^{1}H NMR** (500 MHz, $CDCl_3$) δ 1.28 (s, 9H), 2.80 (s, 3H), 3.62 (s, 3H), 4.35 (dd, *J* = 9.3, 2.4 Hz, 1H), 4.72 (t, *J* = 7.1 Hz, 1H), 4.81 (dd, *J* = 9.7, 7.6 Hz, 1H).

HRMS (ESI) calcd for $C_{30}H_{48}N_3O_7^+$ (M+H)$^+$: 562.3487; found: 562.3494.

methyl (2*S*)-2-[(2*S*)-2-[(2*S*,3*R*)-2-[(2*S*)-2-{[(*tert*-butoxy)carbonyl]amino}propanamido]-3-phenyl-3-(prop-2-en-1-yloxy)propanamido]-*N*,3-dimethylbutanamido]-4-methylpentanoate (SI-49)

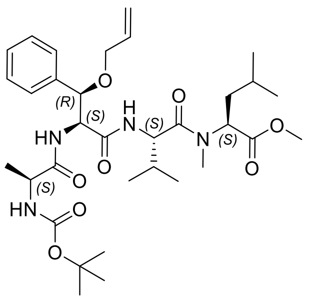

To a solution of **SI-48** (3.00 g, 5.34 mmol) in DCM (40.0 ml, 0.13 M) was added HCl (4N in dioxane) (10.0 ml) dropwise at 0°C and the mixture was allowed to reach rt and stirred for 3 h. The solvents were evaporated in vacuo and the crude amine was used in the peptide coupling without further purification.

To a solution of the deprotected tripeptide (3.00 g, 6.50 mmol) in DMF (30.0 ml, 0.22 M) was added DIPEA (2.27 ml, 13.0 mmol) at 0°C, followed by addition of HOAt (442 mg, 3.25 mmol), Boc-Ala-OH (1.23 g, 6.50 mmol) and HATU (2.72 g, 7.15 mmol). The mixture was allowed to reach rt and stirred for 16 . The reaction was quenched with water and extracted with EtOAc. The organic layer was washed with brine, dried (Na_2SO_4)and concentrated in vacuo. Silica gel column chromatography (50% EtOAc in petroleum ether) yielded **SI-49** (2.00 g, 3.16 mmol, 49%) as a white solid.

$[\alpha]_{20}^{D}$ = –53.6 (c 1.0, $CHCl_3$).

Major rotamer: **^{1}H NMR** (500 MHz, $CDCl_3$) δ 0.90 – 0.94 (m, 6H), 0.95 (d, *J* = 6.7 Hz, 3H), 0.99 (d, *J* = 6.7 Hz, 3H), 1.29 (d, *J* = 7.0 Hz, 3H), 1.45 (s, 9H), 1.47 – 1.53 (m, 1H), 1.67 – 1.78 (m, 2H), 2.06 – 2.17 (m, 1H), 2.98 (s, 3H), 3.69 (s, 3H), 3.89 (ddt, *J* = 12.5, 6.1, 1.4 Hz, 1H), 4.04 (ddt, *J* = 12.5, 5.1, 1.5 Hz, 1H), 4.09 – 4.15 (m, 1H), 4.69 (dd, *J* = 7.5, 3.4 Hz, 1H), 4.81 (dd, *J* = 8.8, 6.1 Hz, 1H), 4.85 – 4.92 (m, 1H), 5.03 (d, *J* = 3.4 Hz, 1H), 5.17 (dq, *J* = 10.5, 1.4 Hz, 1H), 5.25 (dq, *J* = 17.3, 1.6 Hz, 1H), 5.36 (dd, *J* = 10.6, 5.2 Hz, 1H), 5.90 (dddd, *J* = 16.8, 10.8, 6.1, 5.1 Hz, 1H), 6.80 (d, *J* = 7.5 Hz, 1H), 7.20 – 7.31 (m, 5H), 7.38 (d, *J* = 8.8 Hz, 1H). **^{13}C NMR** (126 MHz, $CDCl_3$) δ 17.4, 18.4, 19.6, 21.5, 23.3, 24.8, 28.3, 31.2, 31.4, 37.0, 50.4, 52.1, 54.1, 54.5, 57.9, 70.5, 78.8, 80.2, 117.4, 126.9, 128.2, 128.3, 133.9, 137.1, 155.4, 168.4, 171.9, 172.1, 172.4.

Minor rotamer (selected signals) **^{1}H NMR** (500 MHz, $CDCl_3$) δ 1.01 (d, *J* = 6.7 Hz, 4H), 1.24 (d, *J* = 7.1 Hz, 5H), 1.48 (s, 9H), 2.83 (s, 3H), 3.62 (s, 3H), 4.59 – 4.65 (m, 1H), 6.88 (d, *J* = 8.2 Hz, 1H).

HRMS (ESI) calcd for $C_{33}H_{53}N_4O_8^+$ (M+H)$^+$: 633.3858; found: 688.3866.

Methyl N-(((2*S*,3*R*)-2-((2*S*)-2-((4*R*)-2-((((9*H*-fluoren-9-yl)methoxy)carbonyl)(methyl)amino)-5-((*tert*-butyldimethylsilyl)oxy)-4-methylpentanamido)propanamido)-3-(allyloxy)-3-phenylpropanoyl)-L-valyl)-N-methyl-L-leucinate (SI-50)

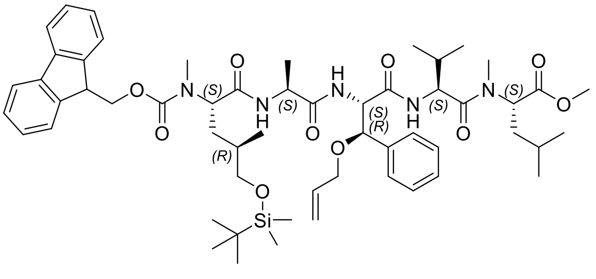

A 25 ml round-bottom flask was charged with **SI-49** (750 mg, 1.20 mmol) and 4 N HCl in 1, 4-dioxane (3.00 ml, 10 equiv) was added at rt. After completion of the reaction, the solvent was evaporated and the crude peptide was reacted according to *GP4*: **SI-21** (590 mg, 1.20 mmol), HOBt (197 mg, 1.30 mmol), EDC (250 mg, 1.30 mmol), NMM (260 µl, 2.40 mmol) (18 h). Flash chromatography (CyH/EtOAc 100:0 – 60:40) followed by lyophilization afforded **SI-50** (868 mg, 860 µmol, 72% yield) as a white amorphous solid. R_f = 0.43 (PE/EtOAc 1:1).

$[\alpha]_{20}^{D}$ = −49.1 (c 0.5, $CHCl_3$).

^{1}H NMR (500 MHz, $CDCl_3$) δ 0.00 – 0.04 (m, 6H), 0.88 (s, 9H), 0.90 –0.98 (m, 12H), 1.00 (d, *J* = 6.8 Hz, 3H), 1.29 (d, *J* = 7.0 Hz, 3H), 1.40 – 1.63 (m, 3H), 1.67 – 1.83 (m, 2H), 1.85 – 2.03 (m, 2H), 2.08 – 2.19 (m, 1H), 2.82 (s, 3H), 2.98 (s, 3H), 3.38 – 3.61 (m, 2H), 3.69 (s, 3H), 3.82 – 3.90 (m, 1H), 3.97 – 4.09 (m, 1H), 4.26 (t, *J* = 6.7 Hz, 1H), 4.31 – 4.40 (m, 1H), 4.43 – 4.54 (m, 2H), 4.67 – 4.77 (m, 2H), 4.81 (dd, *J* = 8.7, 5.8 Hz, 1H), 4.94 – 5.05 (m, 1H), 5.12 – 5.20 (m, 1H), 5.22 – 5.29 (m, 1H), 5.36 (dd, *J* = 10.6, 5.1 Hz, 1H), 5.84 – 5.97 (m, 1H), 6.41 – 6.73 (m, 2H), 7.17 – 7.23(m, 2H), 7.27 – 7.33 (m, 3H), 7.36 – 7.44 (m, 3H), 7.52 – 7.63 (m, 3H), 7.76 (d, *J* = 7.6 Hz, 2H).

^{13}C NMR (126 MHz, $CDCl_3$) δ -5.3, -5.2, 17.4, 17.7, 18.3, 18.5, 19.7, 21.5, 23.4, 25.0, 26.1, 27.0, 29.9, 31.2, 31.3, 31.6, 32.3, 37.1, 47.4, 49.3, 52.3, 54.2, 54.6, 56.8, 57.9, 67.2, 67.9, 70.6, 78.9, 117.6, 120.2, 125.1, 127.0, 127.2, 127.9, 128.4, 128.5, 134.0, 137.0, 141.5, 144.0, 157.3, 168.3, 170.9, 171.6, 172.1, 172.2.

HRMS (ESI): calcd for $C_{56}H_{82}N_5O_{10}Si^+$ (M+H)$^+$: 1012.5825; found: 1012.5807.

Methyl N-(((2*S*,3*R*)-3-(allyloxy)-2-((*S*)-2-((2*S*,4*R*)-2-((*S*)-2-(((allyloxy)carbonyl)amino)-N-methyl-3-(1-methyl-1*H*-indol-3-yl)propanamido)-5-((tert-butyldimethylsilyl)oxy)-4-methylpentanamido)propanamido)-3-phenylpropanoyl)-L-valyl)-*N*-methyl-L-leucinate (SI-51)

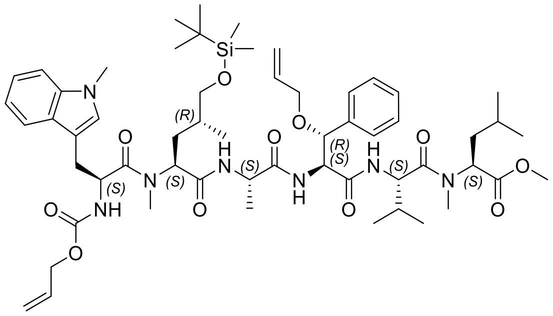

SI-50 (400 mg, 395 µmol) was dissolved in DCM (4.0 ml, 0.1 M), tris(2-aminoethyl)amine (592 µl, 3.95 mmol) was added and the reaction mixture was stirred vigorously. After completion of the reaction (TLC-monitoring; 30 minutes) the reaction mixture was washed with saturated NaCl solution (20 ml ×3) and phosphate buffer (67.0 mM, pH 5.5) (30 ml ×3). The aqueous phase was back-extracted with DCM (1x20 ml). The organic phase was dried (Na_2SO_4) and evaporated in vacuo. The resulting crude peptide was reacted according to *GP3*: *N*α-((allyloxy)carbonyl)-1-methyl-L-tryptophan (143 mg, 474 µmol), BEP (130 mg, 468 µmol), NMM (109 µl, 987 µmol) (18 h). Flash chromatography (CyH/EtOAc 100:0 – 45:55) followed by lyophilization afforded **SI-51** (189 mg, 176 µmol, 44%) as a white amorphous solid. R_f = 0.36 (PE/EtOAc 2:3).

$[\alpha]_{20}^{D}$ = −38.4 (c 1.0, $CHCl_3$).

^{1}H NMR (500 MHz, $CDCl_3$) *(mixture of rotamers, ratio ~5:3:1)* δ -0.01 – -0.02 (m, 1.5H), 0.02 – 0.03 (m, 4.7H), 0.09 (s, 0.5H), 0.16 – 0.19 (m, 0.3H), 0.49 (d, *J* = 6.6 Hz, 0.6H), 0.85 (s, 2.8H), 0.86 – 0.93 (m, 16.2H), 0.94 – 1.01 (m, 3.2H), 1.15 – 1.33 (m, 6.2H), 1.42 (s, 0.6H) 1.44 – 1.58 (m, 3.3H), 1.65 – 1.83 (m, 3.2H), 1.90 – 2.03 (m, 1.8H), 2.02 – 2.25 (m, 2.8H), 2.25 – 2.39 (m, 0.9H), 2.74 – 2.79 (m, 2.8H), 2.81 (s, 3.0H), 2.83 – 2.90 (m, 0.7H), 2.95 – 3.04 (m, 4.3H), 3.05 – 3.32 (m, 3.3H), 3.33 – 3.50 (m, 0.5H), 3.62 – 3.76 (m, 7.9H), 3.80 – 3.95 (m, 1.6H), 3.97 – 4.17 (m, 2.1H), 4.40 – 4.84 (m, 6.7H), 4.85 – 5.42 (m, 9.7H), 5.76 – 6.00 (m, 3.1H), 6.81 – 6.97 (m, 2.0H), 7.06 – 7.16 (m, 1.5H), 7.18 – 7.25 (m, 4.2H), 7.27 – 7.33 (m, 3.2H), 7.50 – 7.57 (m, 0.9H), 7.57 – 7.61 (m, 0.3H), 7.64 (d, *J* = 7.9 Hz, 0.7H).

^{13}C NMR (126 MHz, $CDCl_3$) *(mixture of rotamers)* δ -5.28, -5.26, -5.2, -3.4, 15.9, 17.7, 17.8, 18.5, 19.5, 19.6, 21.5, 23.4, 25.0, 25.3, 25.8, 26.1, 27.0, 28.6, 29.3, 29.8, 30.3, 31.3, 31.5, 31.6, 32.2, 32.8, 37.1, 38.8, 50.1, 50.3, 51.0, 51.7, 52.28, 52.30, 54.3, 54.4, 54.6, 54.7, 54.8, 57.9, 58.2, 58.3, 59.0, 65.9, 66.0, 66.4, 67.3, 67.6, 68.4, 70.6, 70.7, 70.8, 70.9, 78.7, 79.1, 79.3, 108.4, 108.6, 109.5, 109.58, 109.65, 117.4, 117.68, 117.74, 118.2, 118.37, 118.41, 118.7, 119.4, 119.5, 122.0, 122.1, 126.99, 127.04, 127.1, 127.87, 127.92, 128.0, 128.3, 128.4, 128.5, 132.2, 132.3, 132.9, 133.8, 134.1, 137.0, 137.1, 137.3, 156.4, 156.7, 168.5, 168.7, 168.8, 170.7, 171.8, 172.0, 172.20, 172.25, 172.5, 172.7, 173.6.

Selected diagnostic peaks:

Major rotamer: **^{1}H NMR** (500 MHz, $CDCl_3$) *δ* 0.02 (s, 3H), 0.03 (s, 3H), 0.87 (s, 9H), 1.65 – 1.83 (m, 3 H), 2.80 (s, 3H), 3.00 (s, 3H), 3.69 (s, 3H), 6.90 – 6.93 (m, 1H), 7.64 (d, *J* = 7.9 Hz, 1H). **^{13}C-NMR** (126 MHz, $CDCl_3$) *δ* 26.1, 37.1, 79.1, 108.6, 156.4.

Minor rotamer: **^{1}H NMR** (500 MHz, $CDCl_3$) *δ* -0.02 (s, 3H), -0.01 (s, 3H), 0.84 (s, 9H), 2.77 (s, 3H), 2.97 (s, 3H), 3.68 (s, 3H), 6.84 – 6.88 (m, 1H). **^{13}C-NMR** (126 MHz, $CDCl_3$) *δ* 26.0, 79.3, 108.4, 156.7.

HRMS (ESI): calcd for $C_{57}H_{88}N_7O_{11}Si^+$ $(M+H)^+$: 1074.6306; found: 1074.6298.

Methyl *N*-(((2*S*,3*R*)-3-(allyloxy)-2-((*S*)-2-((2*S*,4*R*)-2-((*S*)-2-((*S*)-2-(((allyloxy) (methyl)amino)-3-carbonyl)methylbutanamido)-N-methyl-3-(1-methyl-1*H*-indol-3-yl)propanamido)-5-((tert-butyldimethylsilyl)oxy)-4-methylpentanamido)propanamido)-3-phenylpropanoyl)-L-valyl)-*N*-methyl-L-leucinate (SI-52)

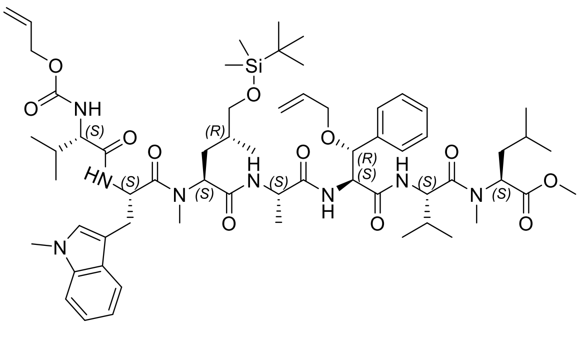

Prepared according to *GP2* and *GP4*: **SI-51** (170 mg, 158 µmol), $Pd(PPh_3)_4$ (5.49 mg, 4.75 µmol), DMBA (74.0 mg, 475 µmol); Alloc-Val-OH (41.5 mg, 206 µmol), NMM (34.9 µl, 317 µmol), HOBt (26.4 mg, 170 µmol), EDC (33.4 mg, 170 µmol) (18 h). Flash chromatography (CyH/EtOAc 100:0 – 60:40) followed by lyohpilization afforded **SI-52** (107 mg, 91.0 µmol, 57% yield) as a white amorphous solid. R_f = 0.45 (PE/EtOAc 2:3).

$[\alpha]_{20}^{D}$ = –58.7 (c 0.5, $CHCl_3$).

^{1}H NMR (500 MHz, $CDCl_3$) *(mixture of rotamers, ratio ~2:1)* δ -0.07 – 0.00 (m, 3.1H), 0.02 (d, *J* = 5.3 Hz, 7.3H), 0.05 – 0.12 (m, 1.2H), 0.45 (d, *J* = 6.7 Hz, 1.0H), 0.83 (m, 9.9H), 0.84 – 0.89 (m, 12.1H), 0.89 – 1.02 (m, 23.8H), 1.12 – 1.32 (m, 5.8H), 1.33 – 1.57 (m, 4.2H), 1.64 – 1.89 (m, 3.0H), 1.89 – 2.23 (m, 3.6H), 2.70 (s, 1.5H), 2.76 (s, 3.0H), 2.79 – 2.94 (m, 1.1H), 3.03 – 3.18 (m, 2.4H), 3.22 (dd, *J* = 14.5, 7.7 Hz, 1.0H), 3.28 – 3.36 (m, 0.4H), 3.41 (d, *J* = 4.6 Hz, 2.4H), 3.62 – 3.72 (m, 9.1H), 3.73 (s, 1.6H), 3.83 – 3.94 (m, 1.8H), 3.99 – 4.07 (m, 2.7H), 4.24 (t, *J* = 7.0 Hz, 0.8H), 4.35 – 4.41 (m, 0.2H), 4.48 – 4.63(m, 3.9H), 4.76 – 4.86 (m, 1.6H), 4.88 – 5.05 (m, 3.4H), 5.05 – 5.46 (m, 10.7H), 5.65 (d, *J* = 9.1 Hz, 0.4H), 5.80 – 6.04 (m, 4.5H), 6.30 (d, *J* = 6.9 Hz, 1.0H), 6.51 (d, *J* = 6.9 Hz, 0.2H), 6.52 –

6.61 (m, 0.1H), 6.68 (d, *J* = 7.5 Hz, 0.2H), 6.74 – 6.83 (m, 2.5H), 6.85 – 6.89 (m, 1.7H), 7.10 (m, 1.7H), 7.17 – 7.25 (m, 5.5H), 7.27 – 7.33 (m, 4.4H), 7.35 – 7.43 (m, 1.0H), 7.43 – 7.50 (m, 2.1H), 7.51 – 7.58 (m, 1.5H), 7.60 – 7.73 (m, 2.8H), 8.15 (d, *J* = 7.3 Hz, 0.4H).

^{13}C NMR (126 MHz, $CDCl_3$) *(mixture of rotamers)* δ -5.3, -5.2, -5.2, 16.1, 17.4, 17.6, 17.7, 18.0, 18.1, 18.4, 18.5, 19.18, 19.25, 19.5, 19.67, 19.68, 21.5, 21.58, 21.60, 23.40, 23.43, 25.0, 26.07, 26.09, 28.2, 28.7, 29.3, 29.8, 31.2, 31.3, 31.37, 31.40, 31.5, 31.61, 31.65, 31.8, 32.3, 32.8, 37.1, 49.4, 50.0, 50.1, 52.27, 52.28, 54.17, 54.22, 54.58, 54.65, 55.9, 57.6, 58.0, 58.3, 60.0, 60.1, 65.9, 66.1, 67.0, 68.5, 70.58, 70.60, 70.7, 78.7, 79.1, 108.42, 108.44, 109.5, 109.6, 117.6, 117.9, 118.1, 118.7, 118.8, 119.5, 119.6, 122.0, 122.2, 127.1, 127.3, 127.8, 128.0, 128.27, 128.31, 128.4, 128.5, 128.6, 128.7, 132.08, 132.10, 132.2, 132.3, 132.8, 134.08, 134.15, 136.99, 137.03, 137.1, 156.2, 156.4, 168.3, 168.4, 168.6, 170.3, 170.9, 171.6, 171.9, 172.0, 172.07, 172.09, 172.2, 172.2, 172.8.

Selected diagnostic peaks:

Major rotamer: **^{1}H NMR** (500 MHz, $CDCl_3$) *δ* 0.01 (s, 3H), 0.02 (s, 3H), 0.87 (s, 9H), 1.64 – 1.89 (m, 3 H), 2.76 (s, 3H), 2.98 (s, 3H), 3.69 (s, 3H), 6.87 – 6.92 (m, 1H). **^{13}C-NMR** (126 MHz, $CDCl_3$) *δ* 23.40, 26.09, 37.1, 54.65, 79.1, 156.2.

Minor rotamer: **^{1}H NMR** (500 MHz, $CDCl_3$) *δ* -0.04 (s, 3H), -0.03 (s, 3H), 0.83 (s, 9H), 2.70 (s, 3H), 2.97 (s, 3H), 3.68 (s, 3H), 6.74 – 6.79 (m, 1H), 8.15 (d, *J* = 7.3 Hz, 1H). **^{13}C-NMR** (126 MHz, $CDCl_3$) *δ* 23.43, 26.07, 54.58, 78.7, 156.4.

HRMS (ESI): calcd for $C_{63}H_{99}N_8O_{12}Si^+$ $(M+H)^+$: 1173.6990; found: 1173.6992.

(3*S*,6*S*,9*S*,12*S*,15*S*,18*S*,21*S*)-15-((*R*)-(Allyloxy)(phenyl)methyl)-21-((*R*)-3-hydroxy-2-methylpropyl)-9-isobutyl-6,12-diisopropyl-1,10,18-trimethyl-3-((1-methyl-1*H*-indol-3-yl)methyl)-1,4,7,10,13,16,19-heptaazacyclohenicosane-2,5,8,11,14,17,20-heptaone (14)

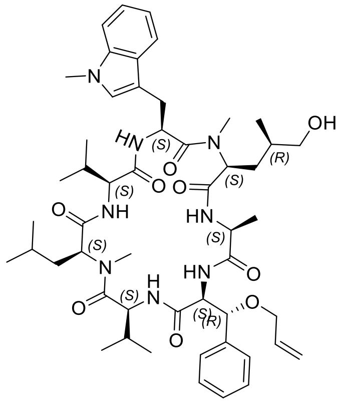

Prepared according to *GP6a*: **SI-52** (81.5 mg, 69.0 µmol), 1 M LiOH (83.0 µl, 83.0 µmol) (3.5 h); $Pd(OAc)_2$ (460 µg, 2.00 µmol), TPPTS (2.35 mg, 4.14 µmol), Et_2NH (36.0 µl, 345 µmol) (1 h); HATU (92.0 mg, 240 µmol), DIPEA (47.0 µl, 280 µmol) (addition over 1.5 h, additional 16 h); NH_4F (51.0 mg, 1.40 mmol) (17 h) RP flash chromatography (H_2O/MeCN 90:10 - 5:95) followed by lyophilizaiton afforded **14** (32.8 mg, 35.0 µmol, 50%) as an off-white, amorphous solid.

$[\boldsymbol{\alpha}]_{20}^{\mathbf{D}}$ = –89.8 (c 0.2, $CHCl_3$).

^{1}H NMR (500 MHz, $CDCl_3$) δ -0.56 – -0.43 (m, 1H), 0.33 (d, *J* = 6.7 Hz, 3H), 0.58 – 0.65 (m, 6H), 0.95 – 1.00 (m, 9H), 1.08 (d, *J* = 6.6 Hz, 3H), 1.18 (d, *J* = 7.2 Hz, 3H), 1.25 (s, 3H), 1.52 – 1.62 (m, 2H), 1.90 – 2.00 (m, 6H), 2.15 – 2.39 (m, 2H), 2.57 (s, 3H), 2.83 (s, 3H), 2.97 – 3.01 (m, 1H), 3.00 – 3.18 (m, 1H), 3.32 (dd, *J* = 13.6, 10.2 Hz, 1H), 3.73 (s, 3H), 3.86 – 4.13 (m, 2H), 4.33 (d, *J* = 10.6 Hz, 1H), 4.47 (t, *J* = 8.5 Hz, 1H), 4.72 – 4.81 (m, 1H), 4.84 (dd, *J* = 10.4, 3.7 Hz, 1H), 4.93 (t, *J* = 5.2 Hz, 1H), 5.21 – 5.29 (m, 2H), 5.30 – 5.44 (m, 1H), 5.85 – 6.06 (m, 1H), 6.74 – 6.92 (m, 2H), 7.09 (t, *J* = 7.4 Hz, 1 H), 7.15 – 7.26 (m, 6H), 7.56 (d, *J* = 7.8 Hz, 1H), 8.21 (d, *J* = 9.4 Hz, 1H), 8.32 (d, *J* = 7.8 Hz, 1H), 8.51 (d, *J* = 10.3 Hz, 1H).

^{13}C NMR (126 MHz, $CDCl_3$) δ 17.4, 18.7, 19.4, 20.1, 20.2, 20.9, 22.7, 23.7, 25.4, 28.5, 29.4, 29.7, 29.8, 31.0, 31.6, 31.8, 32.9, 33.3, 39.1, 50.6, 51.2, 55.4, 56.1, 59.1, 59.4, 65.9, 70.6, 77.8, 107.8, 109.8, 118.1, 118.7, 119.7, 122.4, 127.6, 127.9, 128.2, 128.3, 128.8, 133.5, 135.4, 137.0, 168.8, 169.2, 170.1, 170.7, 171.4, 171.7, 172.2.

HRMS (ESI): calcd for $C_{51}H_{75}N_8O_9{}^+$ (M+H)$^+$: 943.5652; found: 943.5608.

(3*S*,3'*S*,6*S*,6'*S*,9*S*,9'*S*,12*S*,12'*S*,15*S*,15'*S*,18*S*,18'*S*,21*S*,21'*S*)-15,15'-((1*R*,1'*R*)-(((*E*)-But-2-ene-1,4-diyl)bis(oxy))bis(phenylmethylene))bis(21-((*R*)-3-hydroxy-2-methylpropyl)-9-isobutyl-6,12-diisopropyl-1,10,18-trimethyl-3-((1-methyl-1*H*-indol-3-yl)methyl)-1,4,7,10,13,16,19-heptaazacyclohenicosane-2,5,8,11,14,17,20-heptaone) (17)

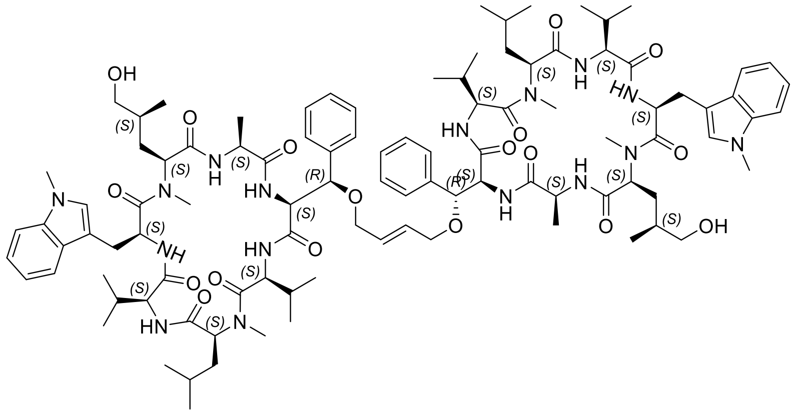

A 4 ml vial was charged with **14** (9.00 mg, 9.65 µmol) and Grubbs catalyst 1st Gen (793 µg, 0.96 µmol, 10 mol%). Freshly degassed (3x freeze-pump-thaw) DCM (48.2 µl) was added, and the vial was flushed with Argon and sealed. The mixture was heated to 45 °C. After 16 h, the reaction mixture was evaporated in vacuo. RP flash chromatography (H_2O/MeCN 90:10 – 5:95) followed by prep HPLC (H_2O/MeCN 70:30 – 0:100) and lyophilization yielded **17** (1.20 mg, 0.70 µmol, 13%) as a white amorphous solid.

$[\alpha]_{20}^{D}$ = –106.3 (c 0.2, $CHCl_3$).

^{1}**H NMR** (500 MHz, $CDCl_3$) δ -0.45 (d, *J* = 14.2 Hz, 2H), 0.30 – 0.38 (m, 6H), 0.57 – 0.68 (m, 12H), 0.85 – 0.97 (m, 18H), 1.02 – 1.11 (m, 6H), 1.11 – 1.19 (m, 6H), 1.21 – 1.49 (m, 20H), 1.51 – 1.76 (m, 4H), 1.77 – 2.38 (m, 6H), 2.57 (s, 6H), 2.81 (s, 6H), 2.87 – 3.01 (m, 2H), 3.01 – 3.23 (m, 2H), 3.26 – 3.40 (m, 2H), 3.71 – 3.75 (s, 6H), 3.91 – 4.12 (m, 4H), 4.13 – 4.28 (m, 2H), 4.29 – 4.41 (m, 2H), 4.46 (t, *J* = 8.4 Hz, 2H), 4.63 – 4.93 (m, 6H), 5.25 (d, *J* = 5.5 Hz, 2H), 5.86 –5.94 (m, 2H), 6.74 – 6.95 (m, 2H), 7.07 – 7.13 (m, 2H), 7.14 – 7.24 (m, 8H), 7.49 – 7.64 (m, 1H), 7.67 – 7.79 (m, 1H), 8.07 – 8.35 (m, 4H), 8.52 (d, *J* = 10.1 Hz, 2H).

^{13}**C NMR** (126 MHz, $CDCl_3$) δ 17.4, 18.7, 19.4, 20.0, 20.2, 20.7, 22.7, 23.7, 25.4, 28.5, 29.4, 29.7, 29.9, 30.9, 31.6, 32.8, 33.2, 39.0, 50.7, 51.2, 55.3, 56.2, 59.1, 59.4, 66.1, 68.3, 69.6, 77.9, 107.8, 109.8, 118.7, 119.7, 122.4, 127.7, 127.9, 128.2, 128.3, 128.9, 131.0, 135.3, 137.0, 168.9, 169.2, 170.0, 170.7, 171.5, 171.7, 172.2.

HRMS (ESI): calcd for $C_{100}H_{145}N_{16}O_{18}{}^+$ (M+H)$^+$: 1858.0917; found: 1858.0905.

Synthesis of Trp-Nα-methylated exit vector 7 Homo-BacPROTACs (21, 22)

Methyl *N*-(((2*S*,3*R*)-2-((*S*)-2-((2*S*,4*R*)-2-((*S*)-2-(((allyloxy)carbonyl)(methyl)amino)-*N*-methyl-3-(1-methyl-1*H*-indol-3-yl)propanamido)-5-((*tert*-butyldimethylsilyl)oxy)-4-methylpentanamido)propanamido)-3-methoxy-3-phenylpropanoyl)-L-valyl)-*N*-methyl-L-leucinate (18)

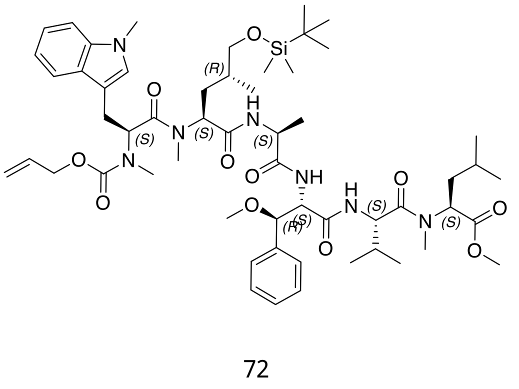

*N*α-((allyloxy)carbonyl)-1-methyl-L-tryptophan (100 mg, 331 µmol) and methyl iodide (124 µl, 1.99 mmol) were dissolved in THF (3.5 ml) and cooled to 0 °C. NaH (60%) (39.7 mg, 992 µmol) was added in one portion and the reaction mixture was slowly warmed to rt overnight. After 22 h, 1 M HCl was added carefully (gas evolution), the resulting mixture was extracted with EtOAc (3x). The combined org phases were washed with 1 M Na_2SO_3 and sat. NaCl soln., dried (Na_2SO_4) and evaporated in vacuo. The crude methylated Trp-derivative was used in the peptide coupling without further purification.

Pentapeptide **2** (270 mg, 301 µmol) was deprotected according to *GP1* and the resulting amine was coupled to crude methylated Trp derivatives (as described above) according to *GP3:* BEP (90.6 mg, 331 µmol), NMM (66.2 µl, 602 µmol) (16 h). Flash chromatography (DCM/EtOAc 100:0 – 50:50) followed by lyophilization afforded **18** (242 mg, 228 µmol, 76%) as a white, solid foam. R_f = 0.48 (DCM/EtOAc 1:1).

$[\alpha]_{20}^{D}$ = −87.5 (c 0.5, $CHCl_3$).

^{1}H NMR (500 MHz, $CDCl_3$) *(mixture of rotamers, ratio ~2:2:1)* δ -0.05 (s, 0.5H), -0.03 (s, 0.6H), -0.01 – 0.06 (m, 4.9H), 0.43 (d, *J* = 6.7 Hz, 0.4H), 0.83 (s, 1.6H), 0.87 – 0.90 (m, 7.6H), 0.90 – 0.97 (m, 11.6H), 0.98 – 1.01 (m, 2.8H), 1.22 – 1.28 (m, 1.7H), 1.28 – 1.36 (m, 1.0H), 1.44 – 1.55 (m, 2.1H), 1.66 – 1.73 (m, 1.0H), 1.73 – 1.81 (m, 1.6H), 1.87 – 1.97 (m, 0.8H), 2.07 – 2.17 (m, 1.0H), 2.75 – 2.81 (m, 2.5H), 2.88 – 2.95 (m, 1.9H), 2.96 – 3.02 (m, 3.0H), 3.02 – 3.05 (m, 0.4H), 3.11 – 3.17 (m, 0.6H), 3.29 (s, 0.6H), 3.33 (s, 2.4H), 3.41 – 3.56 (m, 1.9H), 3.60 – 3.73 (m, 5.3H), 4.10 – 4.23 (m, 0.6H), 4.23 – 4.34 (m, 0.5H), 4.34 – 4.51 (m, 0.2H), 4.46 (dd, *J* = 12.8, 6.1 Hz, 0.3H), 4.50 – 4.74 (m, 2.4H), 4.75 – 4.87 (m, 1.9H), 4.88 – 5.01 (m, 0.2H), 5.03 – 5.30 (m, 2.4H), 5.30 – 5.41 (m, 1.1H), 5.47 – 5.56 (m, 0.3H), 5.59 – 5.72 (m, 0.2H), 5.82 – 6.00 (m, 0.5H), 6.17 (d, *J* = 7.0 Hz, 0.3H), 6.33 (d, *J* = 6.9 Hz, 0.2H), 6.58 – 6.69 (m, 0.8H), 6.82 – 6.96 (m, 0.8H), 7.05 – 7.13 (m, 0.8H), 7.15 – 7.35 (m, 7.9H), 7.52 – 7.63 (m, 0.6H), 7.71 (d, *J* = 8.0 Hz, 0.3H).

^{13}C NMR (126 MHz, $CDCl_3$) *(mixture of rotamers)* δ -5.32, -5.28, 17.3, 17.6, 18.4, 19.7, 21.5, 22.3, 23.4, 24.9, 25.3, 26.0, 29.1, 29.6, 30.0, 30.4, 30.6, 30.8, 31.3, 31.5, 32.2, 32.3, 32.7, 32.8, 37.0, 49.0, 49.2, 49.7, 52.3, 54.2, 54.6, 55.6, 56.7, 57.6, 57.7, 57.9, 66.5, 66.8, 67.1, 81.3, 109.3, 109.4, 117.3, 117.8, 118.6, 118.7, 118.8, 119.1, 119.2, 121.7, 127.0, 127.8, 127.88, 127.94, 128.2, 128.37, 128.42, 128.5, 132.4, 132.9, 136.87, 136.94, 137.0, 155.6, 156.3, 168.4, 170.5, 171.4, 171.9, 172.1, 172.2.

Selected diagnostic peaks:

Major rotamer 1: **^{1}H NMR** (500 MHz, $CDCl_3$) δ 0.03 (s, 3H), 0.04 (s, 3H), 0.89 (s, 9H), 2.78 (s, 3H), 2.99 (s, 3H), 3.33 (s, 3H), 3.69 (s, 4H), 5.32 – 5.35 (m, 1H), 6.92 (s, 1H). **^{13}C NMR** (126 MHz, $CDCl_3$) δ 26.1, 156.3.

Major rotamer 2: **^{1}H NMR** (500 MHz, $CDCl_3$) δ 0.04 (s, 3H), 0.05 (s, 3H), 0.89 (s, 9H), 5.35 – 5.38 (m, 1H), 6.85 (s, 1H).

Minor rotamer: **^{1}H NMR** (500 MHz, $CDCl_3$) δ -0.05 (s, 3H), -0.03 (s, 3H), 0.43 (d, *J* = 6.7 Hz, 3H), 0.83 (s, 9H), 3.29 (s, 3H), 3.65 (s, 3H), 4.46 (dd, *J* = 12.8, 6.1 Hz, 1H), 5.52 (dd, *J* = 9.1, 5.7 Hz, 1H), 6.33 (d, *J* = 6.9 Hz, 1H), 6.88 (s, 1H), 7.71 (d, *J* = 8.0 Hz, 2H). **^{13}C NMR** (126 MHz, $CDCl_3$) δ 156.6.

HRMS (ESI) calcd for $C_{56}H_{88}N_7O_{11}Si^+$ $(M+H)^+$: calcd 1062.6323; found 1062.6306.

methyl *N*-(((2*S*,3*R*)-2-((*S*)-2-((2*S*,4*R*)-2-((*S*)-2-((*S*)-2-((((9*H*-fluoren-9-yl)methoxy)carbonyl)amino)-*N*-methylpent-4-ynamido)-*N*-methyl-3-(1-methyl-1*H*-indol-3-yl)propanamido)-5-((*tert*-butyldimethylsilyl)oxy)-4-methylpentanamido)propanamido)-3-methoxy-3-phenylpropanoyl)-L-valyl)-*N*-methyl-L-leucinate (19)

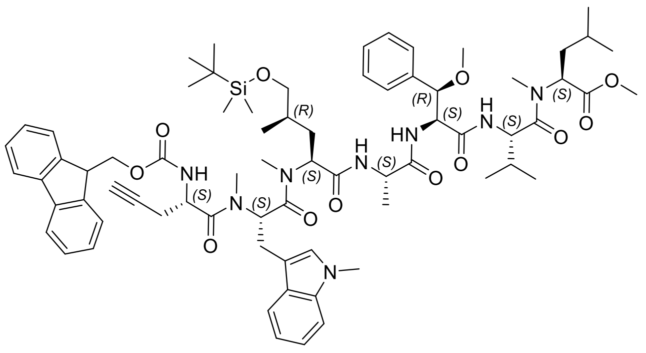

Prepared according to *GP2* and *GP5*: **18** (272 mg, 256 µmol), DMBA (120 mg, 768 µmol), $Pd(PPh_3)_4$ (8.9 mg, 7.68 µmol) (2 h); Fmoc-Pra-OH (120 mg, 358 µmol), $BnNMe_2$ (3.80 µl, 25.6 µmol), DIPEA (58.1 µl, 333 µmol), 1 M iPrOCOCl (358 µl, 358 µmol), NMI (2.04 µl, 25.6 µmol), 4 M HCl (6.4 µl, 25.6 µmol) (4.5 h). Flash chromatography (CyH/EtOAc 100:0 – 30:70) followed by lyophilization yielded **19** (293 mg, 226 µmol, 88%) as a white amorphous solid. R_f = 0.34 (PE/EtOAc 3:7).

$[\boldsymbol{\alpha}]_{20}^{D}$ = −76.9 (c 0.5, $CHCl_3$).

^{1}H NMR (500 MHz, $CDCl_3$) *(mixture of rotamers, ratio ~5:2)* δ -0.06 – 0.00 (m, 1.6H), 0.02 – 0.07 (m, 3.7H), 0.43 (d, *J* = 6.7 Hz, 0.5H), 0.81 – 0.86 (m, 3.1H), 0.89 (s, 6.8H), 0.90 – 1.02 (m, 14.3H), 1.23 – 1.27 (m, 1.1H), 1.30 – 1.37 (m, 1.2H), 1.39 – 1.59 (m, 2.8H), 1.66 – 1.80 (m, 2.0H), 1.84 – 1.88 (m, 0.6H), 1.95 – 2.02 (m, 0.6H), 2.05 (t, *J* = 2.6 Hz, 0.6H), 2.07 – 2.17 (m, 0.9H), 2.54 – 2.63 (m, 0.9H), 2.68 – 2.75 (m, 1.4H), 2.77 (s, 1.4H), 2.80 (s, 1.5H), 2.92 – 2.98 (m, 1.6H), 2.99 (m, 1.9H), 3.09 (s, 1.7H), 3.25 (s, 0.5H), 3.33 (s, 2.5H), 3.34 – 3.61 (m, 0.6H), 3.52 (dd, *J* = 9.7, 4.2 Hz, 1.0H), 3.58 – 3.63 (m, 1.7H), 3.66 – 3.72 (m, 3.4H), 4f.14 (t, *J* = 7.0 Hz, 0.5H), 4.18 – 4.30 (m, 1.1H), 4.33 – 4.48 (m, 1.9H), 4.67 (dd, *J* = 7.5, 3.7 Hz, 0.7H), 4.71 (dd, *J* = 7.0, 3.9 Hz, 0.3H), 4.74 – 4.88 (m, 2.5H), 4.99 – 5.06 (m, 0.6H), 5.30 – 5.39 (m, 0.9H), 5.73 (d, *J* = 8.4 Hz, 0.5H), 5.89 – 5.95 (m, 0.5H), 5.99 (d, *J* = 9.0 Hz, 0.1H), 6.21 (d, *J* = 7.0 Hz, 0.5H), 6.60 (d, *J* = 7.1 Hz, 0.1H), 6.66 (d, *J* = 7.5 Hz, 0.6H), 6.84 (s, 0.2H), 6.91 (s, 0.5H), 7.07 – 7.16 (m, 1.6H), 7.16 – 7.25 (m, 3.9H), 7.27 – 7.37 (m, 4.7H), 7.37 – 7.43 (m, 2.0H), 7.44 – 7.49 m, 2.2H), 7.52 – 7.59 (m, 1.9H), 7.59 – 7.64 (m, 1.5H), 7.64 – 7.70 (m, 2.5H), 7.72 – 7.79 (m, 2.3H).

^{13}C NMR (126 MHz, $CDCl_3$) *(mixture of rotamers)* δ 17.3, 17.9, 18.5, 19.7, 21.5, 23.4, 24.0, 25.0, 25.1, 26.1, 30.9, 31.4, 31.5, 32.2, 32.7, 37.0, 38.7, 47.3, 49.0, 49.7, 52.3, 53.9, 54.2, 54.7, 57.7, 57.9, 67.2, 71.8, 78.6, 81.3, 109.1, 109.4, 118.9, 119.3, 120.2, 121.8, 125.3, 127.0, 127.2, 127.9, 128.4, 128.5, 128.6, 128.7, 132.1, 132.1, 132.2, 132.3, 133.0, 136.8, 137.0, 141.5, 143.9, 155.5, 168.4, 170.2, 171.1, 171.5, 172.1, 172.2.

Selected diagnostic peaks:

Major rotamer: **^{1}H NMR** (500 MHz, $CDCl_3$) δ 0.04 (s, 3H), 0.05 (s, 3H), 0.89 (s, 9H), 2.05 (t, *J* = 2.6 Hz, 1H), 2.80 (s, 3H), 2.98 (d, *J* = 13.2 Hz, 4H), 3.09 (s, 2H), 3.33 (s, 3H), 3.52 (dd, *J* = 9.7, 4.2 Hz, 1H), 3.69 (s, 3H), 4.20 – 4.27 (m, 1H), 4.67 (dd, *J* = 7.5, 3.7 Hz, 1H), 5.35 (dd, *J* = 10.6, 5.2 Hz, 1H), 5.73 (d, *J* = 8.4 Hz, 1H), 6.21 (d, *J* = 7.0 Hz, 1H), 6.66 (d, *J* = 7.5 Hz, 1H), 6.91 (s, 1H). **^{13}C NMR** (126 MHz, $CDCl_3$) δ 26.1, 52.3, 81.3, 155.6.

Minor rotamer: **^{1}H NMR** (500 MHz, $CDCl_3$) δ -0.04 (s, 3H), -0.02 (s, 3H), 0.43 (d, *J* = 6.7 Hz, 3H), 0.84 (s, 9H), 2.77 (s, 3H), 2.96 (s, 3H), 4.14 (t, *J* = 7.0 Hz, 1H), 4.71 (dd, *J* = 7.0, 3.9 Hz, 1H), 5.99 (d, *J* = 9.0 Hz, 1H), 6.60 (d, *J* = 7.1 Hz, 1H), 6.84 (s, 1H).

HRMS (ESI) calcd for $C_{72}H_{99}N_8O_{12}Si^+$ $(M+H)^+$: calcd 1295.7146; found 1295.7156.

(3*S*,6*S*,9*S*,12*S*,15*S*,18*S*,21*S*)-21-((*R*)-3-hydroxy-2-methylpropyl)-9-isobutyl-12-isopropyl-15-((*R*)-methoxy(phenyl)methyl)-1,4,10,18-tetramethyl-3-((1-methyl-1*H*-indol-3-yl)methyl)-6-(prop-2-yn-1-yl)-1,4,7,10,13,16,19-heptaazacyclohenicosane-2,5,8,11,14,17,20-heptaone (20)

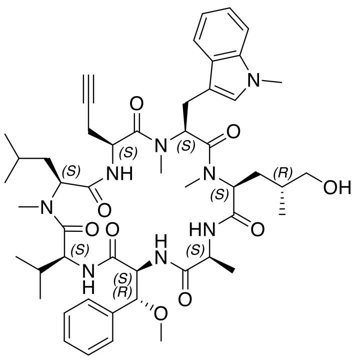

Prepared according to *GP6c*: **19** (50.7 mg, 39.1 µmol), tris(2-aminoethyl)amine (58.6 µl, 391 µmol) (0.5 h); 1 M LiOH (98.0 µl, 98.0 µmol) (4 h); HATU (66.9 mg, 176 µmol), DIPEA (37.6 µl, 215 µmol) (addition over 2.5 h; additional 16 h); NH_4F (14.5 mg, 391 µmol) (16 h). RP flash chromatography (H_2O/MeCN 90:10 – 5:95) followed by lyophilization afforded **20** (16.7 mg, 18.0 mmol, 46%) as a white amorphous solid.

$[\boldsymbol{\alpha}]_{\mathbf{20}}^{\mathbf{D}}$ = −99.5 (c 0.5, $CHCl_3$).

^{1}H NMR (500 MHz, $CDCl_3$) δ -0.33 – -0.19 (m, 1H), 0.37 (d, *J* = 6.8 Hz, 3H), 0.83 – 0.88 (m, 1H), 0.91 – 0.94 (m, 6H), 0.96 (d, *J* = 6.8 Hz, 3H), 0.98 – 1.03 (m, 1H), 1.07 (d, *J* = 6.6 Hz, 3H), 1.16 – 1.21 (m, 1H), 1.27 (d, *J* = 7.3 Hz, 3H), 1.40 – 1.47 (m, 1H), 1.48 – 1.55 (m, 1H), 1.88 (t, *J* = 2.7 Hz, 1H), 1.89 – 1.94 (m, 1H), 2.17 – 2.24 (m, 1H), 2.29 (dt, *J* = 9.8, 6.7 Hz, 1H), 2.44 (bs, 1H), 2.58 (s, 3H), 2.92 (s, 3H), 2.95 – 3.01 (m, 1H), 3.06 – 3.11 (m, 1H), 3.14 (dd, *J* = 14.3, 6.0 Hz, 1H), 3.37 (s, 3H), 3.39 (s, 3H), 3.47 (dd, *J* = 14.0, 9.3 Hz, 1H), 3.71 (s, 3H), 4.53 (dd, *J* = 10.5, 3.1 Hz, 1H), 4.63 (t, *J* = 9.3 Hz, 1H), 4.71 (dd, *J* = 10.0, 4.5 Hz, 1H), 4.80 (dd, *J* = 10.2, 7.3 Hz, 1H), 4.87 – 4.90 (m, 1H), 5.09 (td, *J* = 9.7, 6.1 Hz, 1H), 5.24 (d, *J* = 5.5 Hz, 1H), 5.38 (dd, *J* = 9.4, 6.1 Hz, 1H), 6.82 (s, 1H), 7.05 – 7.12 (m, 1H), 7.12 – 7.18 (m, 2H), 7.18 – 7.25 (m, 2H), 7.27 – 7.31 (m, 5H), 7.46 (d, *J* = 8.0 Hz, 1H), 8.07 (d, *J* = 10.2 Hz, 1H), 8.31 (d, *J* = 9.3 Hz, 1H).

^{13}C NMR (126 MHz, $CDCl_3$) δ 17.4, 19.4, 19.9, 21.1, 22.1, 22.6, 23.5, 25.1, 26.6, 29.08, 29.09, 30.9, 31.9, 32.5, 32.9, 33.2, 39.0, 47.0, 51.1, 53.5, 55.0, 56.3, 58.0, 58.7, 59.3, 66.0, 71.5, 78.2, 79.7, 108.4, 109.7, 118.8, 119.6, 122.4, 127.3, 127.6, 128.2, 128.7, 129.2, 135.3, 137.0, 167.7, 169.6, 169.9, 170.6, 171.3, 172.1, 173.1.

HRMS (ESI) calcd for $C_{50}H_{71}N_8O_9^+$ $(M+H)^+$: calcd 927.5339; found 927.5337.

(3*S*,3'*S*,6*S*,6'*S*,9*S*,9'*S*,12*S*,12'*S*,15*S*,15'*S*,18*S*,18'*S*,21*S*,21'*S*)-6,6'-((((ethane-1,2-diylbis(oxy))bis(ethane-2,1-diyl))bis(1*H*-1,2,3-triazole-1,4-diyl))bis(methylene))bis(21-((*R*)-3-hydroxy-2-methylpropyl)-9-isobutyl-12-isopropyl-15-((*R*)-methoxy(phenyl)methyl)-1,4,10,18-tetramethyl-3-((1-methyl-1*H*-indol-3-yl)methyl)-1,4,7,10,13,16,19-heptaazacyclohenicosane-2,5,8,11,14,17,20-heptaone) (21)

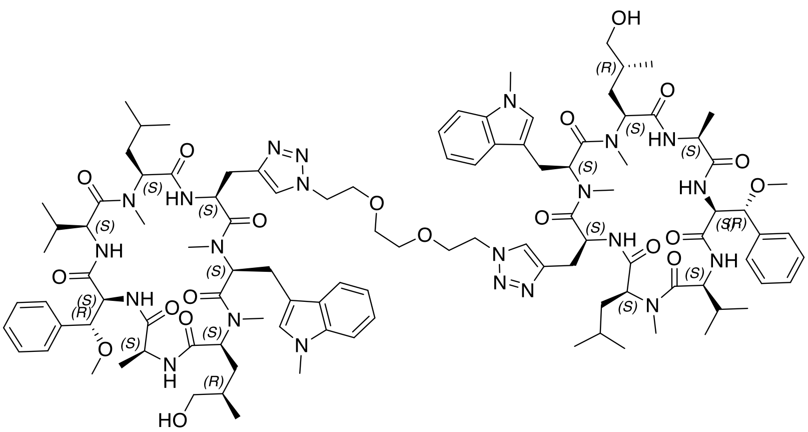

Prepared according to *GP7*: **20** (18.7 mg, 20.2 µmol), 1,2-bis(2-azidoethoxy)ethane (2.02 mg, 10.1 µmol), 1 M $CuSO_4$ (8.08 µl, 8.08 µmol), 1 M sodium ascorbate (13.1 µl, 13.1 µmol) (17 h). RP flash chromatography (H_2O/MeCN 90:10 – 5:95) followed by prep HPLC (H_2O/MeCN 70:30 – 5:95) and lyophilization afforded **21** (12.0 mg, 5.84 µmol, 58%) as a white amorphous solid.

$[\alpha]_{\mathbf{20}}^{\mathbf{D}}$ = −43.2 (c 0.5, $CHCl_3$).

^{1}H NMR (500 MHz, $CDCl_3$) δ 0.02 – 0.11 (m, 2H), 0.36 (d, *J* = 6.7 Hz, 6H), 0.93 – 1.01 (m, 18H), 1.09 (d, *J* = 6.6 Hz, 6H), 1.13 – 1.15 (m, 2H), 1.24 – 1.33 (m, 2H), 1.35 (d, *J* = 7.5 Hz, 6H), 1.45 – 1.57 (m, 4H), 1.94 – 2.08 (m, 4H), 2.15 – 2.22 (m, 2H), 2.26 (dt, *J* = 9.5, 6.6 Hz, 2H), 2.54 (s, 6H), 2.73 (s, 6H), 2.89 (t, *J* = 5.3 Hz, 2H), 3.01 (d, *J* = 1.7 Hz, 10H), 3.13 – 3.28 (m, 4H), 3.35 (s, 6H), 3.62 – 3.70 (m, 10H), 3.82 – 3.95 (m, 4H), 4.48 – 4.53 (m, 2H), 4.52 – 4.59 (m, 2H), 4.69 (dd, *J* = 9.8, 4.6 Hz, 2H), 4.73 – 4.89 (m, 6H), 4.93 – 5.01 (m, 2H), 5.23 – 5.28 (m, 4H), 5.49 – 5.56 (m, 2H), 6.71 (s, 2H), 6.90 – 6.94 (m, 4H), 6.98 – 7.07 (m, 8H), 7.15 – 7.21 (m, 2H), 7.24 (d, *J* = 8.1 Hz, 2H), 7.30 (s, 2H), 7.41 – 7.46 (m, 4H), 8.18 (d, *J* = 9.3 Hz, 2H), 8.48 (d, *J* = 9.2 Hz, 2H), 8.74 (d, *J* = 8.9 Hz, 2H).

^{13}C NMR (126 MHz, $CDCl_3$) δ 17.5, 19.3, 19.8, 19.9, 22.8, 23.7, 25.1, 25.7, 28.8, 29.0, 30.2, 30.3, 31.2, 32.7, 33.3, 33.9, 39.1, 48.1, 50.4, 51.1, 51.5, 54.5, 56.4, 58.0, 58.9, 59.7, 66.6, 69.8, 70.5, 79.3, 108.9, 109.6, 118.8, 119.4, 122.2, 123.0, 127.0, 127.6, 128.2, 128.4, 135.9, 137.0, 141.4, 167.6, 169.2, 170.4, 171.1, 171.3, 171.7, 173.2.

HRMS (ESI) calcd for $C_{106}H_{153}N_{22}O_{20}^+$ $(M+H)^+$: calcd 2054.1626; found 2054.1671.

(3*S*,3'*S*,6*S*,6'*S*,9*S*,9'*S*,12*S*,12'*S*,15*S*,15'*S*,18*S*,18'*S*,21*S*,21'*S*)-6,6'-((pentane-1,5-diylbis(1*H*-1,2,3-triazole-1,4-diyl))bis(methylene))bis(21-((*R*)-3-hydroxy-2-methylpropyl)-9-isobutyl-12-isopropyl-15-((*R*)-methoxy(phenyl)methyl)-1,4,10,18-tetramethyl-3-((1-methyl-1*H*-indol-3-yl)methyl)-1,4,7,10,13,16,19-heptaazacyclohenicosane-2,5,8,11,14,17,20-heptaone) (22)

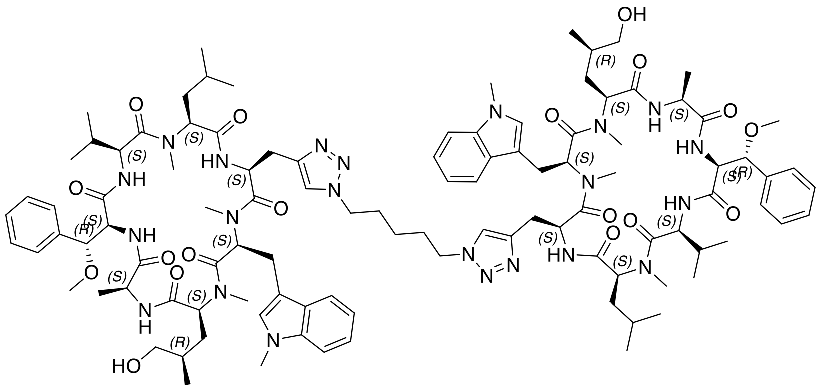

Prepared according to *GP7*: **20** (16.0 mg, 17.3 µmol), 1,5-diazidopentane (1.33 mg, 8.63 µmol), 1 M $CuSO_4$ (6.90 µl, 6.90 µmol), 1 M sodium ascorbate (11.2 µl, 11.2 µmol) (17 h). RP flash chromatography (H_2O/MeCN 90:10 – 5:95) followed by prep HPLC (H_2O/MeCN 75:25 – 5:95) and lyophilization afforded **22** (10.6 mg, 5.28 µmol, 61%) as a white amorphous solid.

$[\alpha]^{D}_{20}$ = −66.5 (c 0.2, $CHCl_3$).

^{1}H NMR (500 MHz, $CDCl_3$) δ 0.06 – 0.13 (m, 2H), 0.36 (d, *J* = 6.7 Hz, 6H), 0.81 – 0.87 (m, 2H), 0.92 (d, *J* = 6.7 Hz, 6H), 0.94 – 0.96 (m, 12H), 1.04 – 1.09 (m, 8H), 1.24 – 1.29 (m, 2H), 1.31 (d, *J* = 7.4 Hz, 6H), 1.44 – 1.51 (m, 4H), 1.52 – 1.58 (m, 2H), 1.95 – 2.05 (m, 8H), 2.17 (ddd, *J* = 14.3, 9.6, 5.2 Hz, 2H), 2.21 – 2.28 (m, 2H), 2.59 (s, 6H), 2.72 (s, 6H), 2.96 – 3.04 (m, 10H), 3.16 (dd, *J* = 11.1, 4.3 Hz, 2H), 3.21 – 3.27 (m, 2H), 3.33 (s, 6H), 3.64 (s, 6H), 4.32 – 4.42 (m, 4H), 4.67 (dd, *J* = 9.7, 4.4 Hz, 2H), 4.70 – 4.80 (m, 4H), 4.81 – 4.86 (m, 2H), 4.90 – 4.99 (m, 2H), 5.15 (dd, *J* = 9.6, 3.4 Hz, 2H), 5.23 (d, *J* = 5.4 Hz, 2H), 5.50 (t, *J* = 7.6 Hz, 2H), 6.70 (s, 2H), 6.93 (t, *J* = 7.6 Hz, 4H), 7.00 – 7.06 (m, 8H), 7.15 – 7.20 (m, 4H), 7.22 (d, *J* = 8.3 Hz, 2H), 7.37 – 7.46 (m, 4H), 8.18 (d, *J* = 9.0 Hz, 2H), 8.44 (d, *J* = 9.1 Hz, 2H), 8.67 (d, *J* = 9.0 Hz, 2H).

^{13}C NMR (126 MHz, $CDCl_3$) δ 17.5, 19.3, 19.9, 20.0, 22.8, 23.61, 23.64, 25.1, 25.8, 28.9, 29.0, 29.9, 30.1, 30.5, 31.2, 32.8, 33.3, 33.7, 39.1, 48.2, 50.1, 51.2, 52.0, 54.6, 56.4, 58.0, 58.9, 59.7, 66.6, 79.4, 108.8, 109.6, 118.8, 119.4, 121.7, 122.2, 127.0, 127.6, 128.19, 128.23, 128.5, 135.8, 137.0, 141.8, 167.5, 169.3, 170.7, 171.0, 171.4, 171.7, 173.0.

HRMS (ESI) calcd for $C_{105}H_{151}N_{22}O_{18}^+$ $(M+H)^+$: calcd 2008.1571; found 2008.1597.

(3*S*,6*S*,9*S*,12*S*,15*S*,18*S*,21*S*)-21-((*R*)-3-hydroxy-2-methylpropyl)-9-isobutyl-12-isopropyl-15-((*R*)-methoxy(phenyl)methyl)-1,4,10,18-tetramethyl-3-((1-methyl-1*H*-indol-3-yl)methyl)-6-((1-pentyl-1*H*-1,2,3-triazol-4-yl)methyl)-1,4,7,10,13,16,19-heptaazacyclohenicosane-2,5,8,11,14,17,20-heptaone (25)

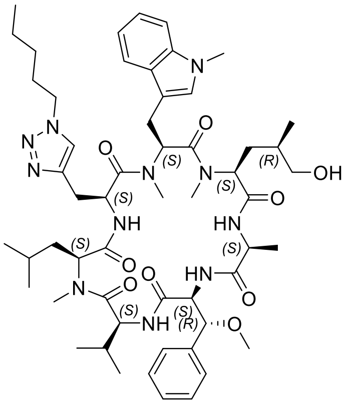

A 1.5 ml vial was charged with **20** (13.1 mg, 14.1 µmol), *t*-BuOH (235 µl) and H_2O (235 µl) were added followed by 1-azidopentane (24.6 µl of a 13.0 mg ml^{-1} stock solution, 3.20 mg, 28.3 µmol). 1 M $CuSO_4$ (5.65 µl, 5.65 µmol) and 1 M sodium ascorbate (8.48 µl, 8.48 µmol) were added, the vial was flushed with Argon and sealed. After 4 h, incomplete conversion was observed (LC/MS) and more 1-azidopentane (15.4 µl of a 13.0 mg ml^{-1} stock solution, 2.00 mg, 17.7 µmol) was added. After 5 h, full conversion was observed by LC/MS. The reaction mixture was evaporated in vacuo and the residue was purified by RP flash chromatography (H_2O/MeCN 75:25 – 5:95). After lyophilization, **25** (11.5 mg, 11.1 µmol, 78%) was obtained as a white amorphous solid.

$[\alpha]_{20}^{D}$ = –60.8 (c 0.5, $CHCl_3$).

^{1}H NMR (500 MHz, $CDCl_3$) δ 0.05 – 0.13 (m, 1H), 0.38 (d, *J* = 6.7 Hz, 3H), 0.85 – 0.91 (m, 1H), 0.90 – 1.00 (m, 12H), 1.06 – 1.14 (m, 4H), 1.28 – 1.34 (m, 5H), 1.35 – 1.44 (m, 2H), 1.47 – 1.60 (m, 2H), 1.84 – 1.96 (m, 3H), 1.99 (dd, *J* = 14.2, 5.8 Hz, 1H), 2.02 – 2.08 (m, 1H), 2.15 – 2.23 (m, 1H), 2.23 – 2.30 (m, 1H), 2.59 (s, 3H), 2.73 (s, 3H), 2.97 – 3.05 (m, 5H), 3.18 (dd, *J* = 11.1, 4.4 Hz, 1H), 3.22 – 3.28 (m, 1H), 3.35 (s, 3H), 3.67 (s, 3H), 4.30 – 4.36 (m, 2H), 4.70 (dd, *J* = 9.9, 4.5 Hz, 1H), 4.73 – 4.81 (m, 2H), 4.85 (dd, *J* = 5.4, 3.8 Hz, 1H), 4.93 – 5.00 (m, 1H), 5.17 (dd, *J* = 9.6, 3.5 Hz, 1H), 5.25 (d, *J* = 5.3 Hz, 1H), 5.49 – 5.55 (m, 1H), 6.74 (s, 1H), 6.90 – 6.98 (m, 2H), 7.03 – 7.12 (m, 5H), 7.18 – 7.22 (m, 1H), 7.22 – 7.25 (m, 1H), 7.40 (d, *J* = 3.9 Hz, 1H), 7.45 (d, *J* = 8.0 Hz, 1H), 8.20 (d, *J* = 9.2 Hz, 1H), 8.45 (d, *J* = 9.0 Hz, 1H), 8.72 (d, *J* = 8.9 Hz, 1H.

^{13}C NMR (126 MHz, $CDCl_3$) δ 14.1, 17.6, 19.3, 19.88, 19.91, 22.2, 22.8, 23.6, 25.1, 25.7, 28.7, 28.9, 29.0, 29.8, 30.1, 30.4, 31.2, 32.8, 33.4, 33.7, 39.1, 48.2, 50.5, 51.2, 52.0, 54.6, 56.3, 58.0, 58.9, 59.7, 66.5, 79.4, 108.9, 109.6, 118.9, 119.4, 121.5, 122.2, 127.0, 127.7, 128.19, 128.22, 128.5, 135.8, 137.0, 141.6, 167.5, 169.3, 170.5, 171.0, 171.5, 171.6, 173.1.

HRMS (ESI) calcd for $C_{55}H_{82}N_{11}O_9^+$ (M+H)$^+$: 1040.6291; found: 1040.6276.

Synthesis of Trp-Nα-methylated dCymC (23)

Methyl *N*-(((2*S*,3*R*)-2-((*S*)-2-((2*S*,4*R*)-2-((*S*)-2-(((allyloxy)carbonyl)(methyl)amino)-*N*-methyl-3-(1-(2-methylbut-3-en-2-yl)-1*H*-indol-3-yl)propanamido)-5-((*tert*-butyldimethylsilyl)oxy)-4-methylpentanamido)propanamido)-3-methoxy-3-phenylpropanoyl)-L-valyl)-*N*-methyl-L-leucinate (SI-53)

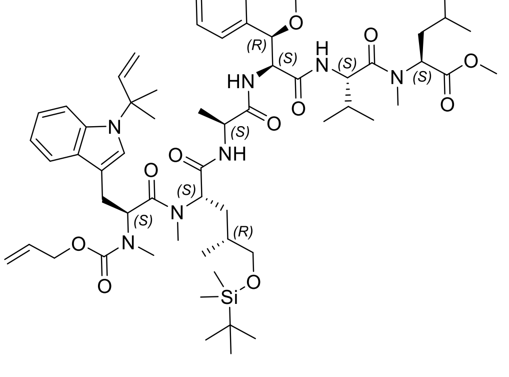

*N*α-((allyloxy)carbonyl)-1-(2-methylbut-3-en-2-yl)-L-tryptophan (203 mg, 0.570 mmol) and methyl iodide (214 µl, 3.42 mmol) were dissolved in THF (5.7 ml) and cooled to 0 °C. NaH (91.0 mg, 2.23 mmol) was added in portions and the reaction mixture was slowly warmed to rt over night. After 17 h, H_2O was added carefully (gas evolution), the resulting mixture was washed with PE (2x) and the PE phase was extracted with 0.5 M NaOH (1x). The aqueous phases were combined, acidified with 1 M HCl and extracted with EtOAc (3x). The combined org phases were washed with half saturated $Na_2S_2O_3$ and sat. NaCl soln., dried (Na_2SO_4) and evaporated in vacuo. The crude methylated Trp-derivative was then used in the next step without further purification.

Pentapeptide **2** (787 mg, 0.876 mmol) was deprotected according to *GP1* and the resulting amine was coupled to the crude *Nα*-methyl-1-(2-methylbut-3-en-2-yl)Trp (prepared as described above) according to *GP5:* DIPEA (100 µl, 570 µmol), $BnNMe_2$ (6.51 µl, 43.8 µmol), 1 M iPrOCOCl soln. (570 µl, 570 µmol), NMI (6.99 µl, 87.7 µmol), 4 M HCl soln. (11.0 µl, 43.8 µmol) (18 h). Flash chromatography (CyH/EtOAc 100:0 – 35:65) followed by lyophilization **SI-53** (402 mg, 0.331 mmol, 76%, 92% purity) as a white, amorphous solid. R_f = 0.27 (PE/EtOAc 1:1).

$[\alpha]_{20}^{D}$ = −80.4 (c 0.5, $CHCl_3$).

^{1}H NMR (500 MHz, $CDCl_3$) *(mixture of rotamers, ratio ~2:2:1)* δ -0.46 – -0.35 (m, 0.3H), -0.07 (s, 0.8H), -0.05 (s, 0.9H), 0.01 – 0.07 (m, 4.9H), 0.31 (d, *J* = 6.6 Hz, 0.8H), 0.81 (s, 3.1H), 0.86 – 0.91 (m, 9.2H), 0.91 – 1.02 (m, 15.0H), 1.05 (d, *J* = 7.1 Hz, 1.0H), 1.18 (d, *J* = 7.1 Hz, 0.7H), 1.22 (d, *J* = 7.1 Hz, 0.8H), 1.23 – 1.35 (m, 2.2H), 1.44 – 1.56 (m, 2.7H), 1.66 – 1.71 (m, 6.6H), 1.72 – 1.78 (m, 1.7H), 1.88 – 1.97 (m, 0.9H), 2.06 – 2.19 (m, 1.3H), 2.62 – 2.70 (m, 0.3H), 2.74 – 2.80 (m, 1.3H), 2.82 – 2.91 (m, 4.1H), 2.95 – 3.02 (m, 3.7H), 3.08 – 3.16 (m, 1.0H), 3.18 (s, 0.9H), 3.28 (s, 1.0H), 3.31 – 3.34 (m, 2.6H), 3.34 – 3.44 (m, 1.5H), 3.50 (dd, *J* = 9.7, 4.3 Hz, 0.9H), 3.66 – 3.71 (m, 3.3H), 3.93 – 4.00 (m, 0.2H), 4.19 (t, *J* = 6.9 Hz, 0.3H), 4.23 – 4.29 (m, 0.7H), 4.31 – 4.38 (m, 0.3H), 4.52 – 4.59 (m, 1.1H), 4.60 – 4.73 (m, 1.6H), 4.75 – 4.87 (m, 2.5H), 5.01 – 5.13 (m, 1.7H), 5.13 – 5.33 (m, 3.7H), 5.32 – 5.38 (m, 1.1H), 5.40 – 5.53 (m, 0.6H), 5.79 – 5.97 (m, 0.7H), 6.03 – 6.14 (m, 1.0H), 6.39 (d, *J* = 6.6 Hz, 0.3H), 6.49 (d, *J* = 6.6 Hz, 0.2H), 6.65 (d, *J* = 7.6 Hz, 0.3H), 6.67 – 6.72 (m, 0.6H), 6.81 (d, *J* = 7.6 Hz, 0.1H), 7.03 – 7.13 (m, 2.7H), 7.14 – 7.25 (m, 3.9H),7.25 – 7.32 (m, 3.2H), 7.33 – 7.39 (m, 1.0H), 7.42 – 7.50 (m, 1.1H), 7.55 – 7.60 (m, 0.6H), 7.62 (d, *J* = 6.6 Hz, 0.3H), 7.66 – 7.71 (m, 0.3H).

^{13}C NMR (126 MHz, $CDCl_3$) *(mixture of rotamers)* δ -5.3, -5.2, 17.4, 17.6, 17.7, 18.4, 18.5, 19.66, 19.70, 21.5, 23.4, 24.9, 26.1, 28.0, 29.0, 29.6, 29.9, 30.1, 30.6, 31.0, 31.4, 31.5, 32.3, 37.0, 49.2, 49.6, 52.3, 53.8, 54.2, 54.6, 55.7, 57.6, 57.7, 57.8, 59.0, 66.4, 66.6, 66.8, 67.2, 68.1, 81.3, 108.0, 108.8, 109.0, 113.5, 113.6, 113.9, 117.3, 117.9, 118.4, 118.6, 118.8, 119.0, 119.4, 120.8, 121.3, 124.0, 124.3, 127.0, 128.2, 128.3, 128.5, 129.1, 129.4, 129.6, 132.3, 132.6, 132.9, 135.5, 136.8, 144.3, 155.6, 156.4, 157.4, 168.5, 170.5, 171.5, 171.8, 172.1, 172.2.

Selected diagnostic peaks:

Major rotamers: **^{1}H NMR** (500 MHz, $CDCl_3$) δ 0.03 (s, 3H), 0.03 (s, 3H), 0.05 (s, 3H), 0.88 (s, 9H), 0.89 (s, 9H), 1.69 (s, 3H), 2.87 (s, 3H), 2.87 (s, 3H), 2.97 (s, 3H), 2.98 (s, 3H), 3.69 (s, 3H), 4.26 (t, *J* = 6.8 Hz, 1H), 5.35 (dd, *J* = 10.6, 5.4 Hz, 3H), 6.69 (d, *J* = 7.4 Hz, 1H). **^{13}C NMR** (126 MHz, $CDCl_3$) δ 26.1, 52.3, 81.3, 127.0, 128.5, 156.4.

Minor rotamer: **^{1}H NMR** (500 MHz, $CDCl_3$) δ -0.44 – -0.37 (m, 1H), -0.07 (s, 3H), -0.05 (s, 3H), 0.31 (d, *J* = 6.6 Hz, 3H), 0.81 (s, 9H), 1.05 (d, *J* = 7.1 Hz, 3H), 3.18 (s, 3H), 3.28 (s, 3H), 3.50 (dd, *J* = 9.7, 4.3 Hz, 2H), 3.97 (dd, *J* = 12.9, 5.8 Hz, 1H), 4.19 (t, *J* = 6.9 Hz, 1H), 6.49 (d, *J* = 6.6 Hz, 1H), 6.65 (d, *J* = 7.6 Hz, 1H).

HRMS (ESI): calcd for $C_{60}H_{97}N_7O_{11}Si^+$ $(M+H)^+$: 1116.6775; found:1116.6776.

methyl *N*-(((2*S*,3*R*)-2-((*S*)-2-((2*S*,4*R*)-2-((*S*)-2-((2*S*,3*R*)-2-(((allyloxy)carbonyl)amino)-*N*,3,5-trimethylhex-4-enamido)-*N*-methyl-3-(1-(2-methylbut-3-en-2-yl)-1*H*-indol-3-yl)propanamido)-5-((*tert*-butyldimethylsilyl)oxy)-4-methylpentanamido)propanamido)-3-methoxy-3-phenylpropanoyl)-L-valyl)-*N*-methyl-L-leucinate (SI-54)

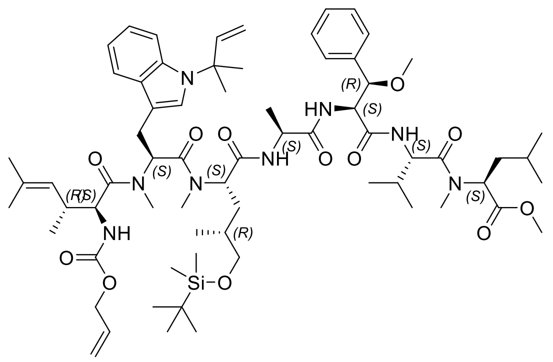

Prepared according to *GP2* and *GP5*: **SI-53** (368 mg, 330 µmol), DMBA (154 mg, 989 µmol), $Pd(PPh_3)_4$ (11.4 mg, 9.89 µmol) (1 h); (2*S*,3*R*)-2-(((allyloxy)carbonyl)amino)-3,5-dimethylhex-4-enoic acid (108 mg, 448 µmol), DIPEA (74.9 µl, 429 µmol), $BnNMe_2$ (4.90 µl, 33.0 µmol), 1 M iPrOCOCl (429 µl, 429 µmol), NMI (5.26 µl, 6.60 µmol), 4 M HCl (8.25 µl, 2.06 µmol) (17 h). Flash chromatography (CyH/EtOAc 100:0 – 35:65) followed by lyophilization yielded **SI-54** (323 mg, 255 µmol, 77%) as a yellow amorphous solid. R_f = 0.33 (PE/EtOAc 1:1).

$[\alpha]^{D}_{20}$ = −75.8 (c 0.5, $CHCl_3$).

^{1}H NMR (500 MHz, $CDCl_3$) *(mixture of rotamers, ratio ~2:2:1)* δ -0.56 – -0.4 (m, 0.2H), -0.07 (s, 0.9H), -0.05 (s, 0.9H), 0.00 – 0.07 (m, 4.3H), 0.30 (d, *J* = 6.6 Hz, 0.7H), 0.80 – 0.82 (m, 2.7H), 0.88 (s, 5.1H), 0.90 – 1.01 (m, 17.2H), 1.13 – 1.23 (m, 0.8H), 1.23 – 1.30 (m, 2.2H), 1.45 – 1.53 (m, 3.0H), 1.57 – 1.60 (m, 1.6H), 1.65 – 1.71 (m, 8.1H), 1.71 – 1.78 (m, 1.6H), 1.80 – 1.87 (m, 1.3H), 1.89 – 1.97 (m, 0.7H), 2.06 – 2.17 (m, 1.1H), 2.60 – 2.67 (m, 0.4H), 2.72 (s, 1.0H), 2.75 – 2.79 (m, 0.9H), 2.80 (s, 1.3H), 2.82 – 2.88 (m, 1.0H), 2.95 – 3.00 (m, 3.2H), 3.00 – 3.06 (m, 0.7H), 3.08 (s, 1.2H), 3.10 – 3.21 (m, 0.7H), 3.27 – 3.34 (m, 3.9H), 3.37 – 3.45 (m, 1.4H), 3.50 (dd, *J* = 9.7, 4.0 Hz, 0.6H), 3.66 – 3.73 (m, 3.0H), 4.14 – 4.19 (m, 0.4H), 4.23 – 4.34 (m, 0.4H), 4.44 – 4.50 (m, 0.5H), 4.51 – 4.62 (m, 1.6H), 4.62 – 4.70 (m, 1.1H), 4.73 (dd, *J* = 7.1, 4.1 Hz, 0.3H), 4.77 – 4.90 (m, 2.4.H), 4.99 – 5.04 (m, 0.7H), 5.05 – 5.09 (m, 0.4H), 5.09 – 5.24 (m, 2.9H), 5.26 – 5.43 (m, 2.4H), 5.82 (t, *J* = 7.3 Hz, 0.4H), 5.85 – 6.01 (m, 0.8H), 6.03 – 6.13 (m, 1.1H), 6.32 (d, *J* = 6.8 Hz, 0.4H), 6.52 (d, *J* = 7.1 Hz, 0.2H), 6.61 – 6.73 (m, 0.6H), 7.01 (s, 0.2H); 7.04 – 7.12 (m, 2.0H), 7.12 – 7.24 (m, 3.7H), 7.25 – 7.30 (m, 2.7H), 7.33 – 7.37 (m, 0.6H), 7.40 – 7.46 (m, 0.8H), 7.47 – 7.41 (m, 0.4H), 7.52 – 7.55 (m, 0.3H), 7.67 – 7.72 (m, 0.5H), 8.30 (d, *J* = 6.3 Hz, 0.2H).

^{13}C NMR (126 MHz, $CDCl_3$) δ -5.30, -5.27, 15.6, 16.3, 17.3, 17.7, 18.2, 18.4, 18.5, 19.7, 21.5, 23.4, 24.9, 26.0, 26.1, 26.2, 27.9, 28.9, 30.6, 31.1, 31.3, 31.5, 32.3, 35.8, 37.0, 49.2, 49.9, 52.3, 54.2, 54.6, 54.8, 57.56, 57.65, 57.8, 59.0, 59.1, 65.9, 67.2, 68.3, 81.3, 107.8, 108.8, 113.4, 113.6, 113.8, 114.0, 117.8, 118.8, 119.0, 119.4, 120.9, 124.0, 125.5, 127.0, 127.1, 128.4, 128.5, 129.4, 132.8, 133.1, 133.4, 135.6, 136.8, 144.4, 156.0, 168.4, 168.5, 170.3, 171.4, 171.7, 171.8, 172.0, 172.1, 172.2, 173.3.

Selected diagnostic peaks:

Major rotamers: **^{1}H NMR** (500 MHz, $CDCl_3$) δ 0.03 (s, 3H), 0.04 (s, 3H), 0.88 (s, 9H), 2.98 (s, 3H), 3.32 (s, 3H), 3.50 (dd, *J* = 9.7, 4.0 Hz, 1H), 3.69 (s, 3H), 4.16 (t, *J* = 6.9 Hz, 1H), 5.82 (t, *J* = 7.3 Hz, 1H), 6.32 (d, *J* = 6.8 Hz, 1H), 6.64 (d, *J* = 7.6 Hz, 1H). **^{13}C NMR** (126 MHz, $CDCl_3$) δ 26.1, 81.3, 156.0.

Minor rotamer: **^{1}H NMR** (500 MHz, $CDCl_3$) δ -0.49 (s, 1H), -0.07 (s, 3H), -0.05 (s, 3H), 0.30 (d, *J* = 6.6 Hz, 3H), 0.81 (s, 9H), 2.72 (s, 3H), 2.80 (s, 3H), 3.08 (s, 3H), 3.30 (s, 3H), 3.50 (dd, *J* = 9.7, 4.0 Hz, 1H), 4.31 (t, *J* = 6.8 Hz, 1H), 6.52 (d, *J* = 7.1 Hz, 1H), 8.30 (d, *J* = 6.3 Hz, 1H). **^{13}C NMR** (126 MHz, $CDCl_3$) δ 26.0.

HRMS (ESI): calcd for $C_{68}H_{107}N_8O_{12}Si^+$ $(M+H)^+$: 1025.6434; found:1025.6429.

(3*S*,6*S*,9*S*,12*S*,15*S*,18*S*,21*S*)-21-((*R*)-3-hydroxy-2-methylpropyl)-9-isobutyl-12-isopropyl-15-((*R*)-methoxy(phenyl)methyl)-1,4,10,18-tetramethyl-3-((1-(2-methylbut-3-en-2-yl)-1*H*-indol-3-yl)methyl)-6-((*R*)-4-methylpent-3-en-2-yl)-1,4,7,10,13,16,19-heptaazacyclohenicosane-2,5,8,11,14,17,20-heptaone (23)

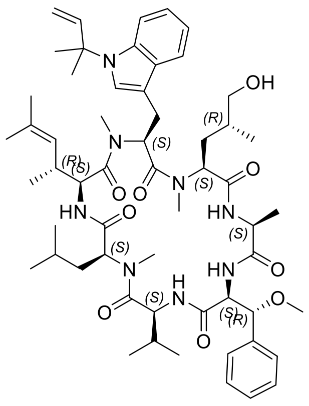

Prepared according to *GP6c*: **SI-54** (180 mg, 143 µmol), 1 M LiOH (229 µl, 229 µmol) (5.5 h); $Pd(OAc)_2$ (1.3 mg, 5.7 µmol), TPPTS (6.5 mg, 11 µmol), Et_2NH (74.7 µl, 715 µmol) (2.5 h); HATU (217 mg, 572 µmol), DIPEA (125 µl, 715 µmol) (addition over 4 h, additional 18 h); NH_4F (132 mg, 3.58 mmol) (16 h). RP flash chromatography (H_2O/MeCN 90:10 - 5:95) followed by prep HPLC (H_2O/MeCN 70:30 – 5:95) and lyophilizaiton afforded **23** (42.4 mg, 36.4 µmol, 25%, purity 88%) as a white, amorphous solid.

$[\alpha]_{20}^{D}$ = −76.5 (c 0.5, $CHCl_3$).

^{1}H NMR (500 MHz, $CDCl_3$) δ -0.46 – -0.36 (m, 2H), 0.17 (d, *J* = 6.9 Hz, 3H), 0.46 (d, *J* = 6.7 Hz, 3H), 0.93 – 1.04 (m, 10H), 1.09 – 1.13 (m, 4H), 1.23 (d, *J* = 7.3 Hz, 3H), 1.31 (d, *J* = 1.5 Hz, 3H), 1.48 – 1.58 (m, 1H), 1.65 – 1.69 (m, 6H), 1.69 (d, *J* = 1.4 Hz, 3H), 1.69 – 1.75 (m, 1H), 1.98 (d, *J* = 7.5 Hz, 2H), 2.20 – 2.28 (m, 1H), 2.32 (ddd, *J* = 13.3, 10.7, 4.5 Hz, 1H), 2.66 (s, 3H), 2.86 (s, 3H), 2.89 (dd, *J* = 11.0, 6.1 Hz, 1H), 2.97 (dd, *J* = 10.8, 4.9 Hz, 1H), 3.16 (dd, *J* = 14.1, 6.2 Hz, 1H), 3.32 (s, 3H), 3.35 – 3.40 (m, 4H), 4.37 – 4.46 (m, 1H), 4.70 – 4.78 (m, 2H), 4.82 – 4.92 (m, 3H), 4.94 (dd, *J* = 10.7, 3.5 Hz, 1H), 5.10 (d, *J* = 5.3 Hz, 1H), 5.13 (dd, *J* = 9.5, 6.2 Hz, 1H), 5.18 (d, *J* = 17.5 Hz, 1H), 5.21 (d, *J* = 10.8 Hz, 1H), 6.06 (dd, *J* = 17.5, 10.7 Hz, 1H), 6.94 (d, *J* = 4.7 Hz, 1H), 6.99 (s, 1H), 7.02 – 7.14 (m, 2H), 7.19 – 7.28 (m, 5H), 7.43 (d, *J* = 7.0 Hz, 0H), 7.49 (d, *J* = 8.1 Hz, 1H), 7.58 (d, *J* = 10.1 Hz, 1H), 7.99 (d, *J* = 7.6 Hz, 1H), 8.40 (d, *J* = 9.6 Hz, 1H).

^{13}C NMR (126 MHz, $CDCl_3$) δ 16.7, 18.0, 18.9, 19.5, 20.2, 20.7, 22.6, 23.8, 25.3, 25.8, 26.6, 28.0, 28.1, 29.0, 29.6, 31.1, 32.2, 33.0, 35.9, 39.2, 50.8, 53.4, 53.7, 55.6, 55.9, 57.9, 58.7, 58.8, 59.1, 66.8, 80.1, 107.4, 113.8, 114.3, 118.4, 119.5, 121.5, 124.0, 125.1, 128.1, 128.6, 129.0, 129.1, 133.6, 135.2, 135.6, 144.0, 168.5, 169.5, 170.8, 171.5, 172.3, 174.3.

HRMS (ESI): calcd for $C_{57}H_{85}N_8O_9^+$ $(M+H)^+$: 1025.6434; found: 1025.6429.

Synthesis of Trp-Nα-methylated exit vector 6 Homo-BacPROTACs (SI-57, 27, 28, SI-61)

methyl *N*-(((2*R*,3*S*)-2-((*S*)-2-((2*S*,4*R*)-2-((*S*)-2-(((allyloxy)carbonyl)(methyl)amino)-*N*-methyl-3-(1-(prop-2-yn-1-yl)-1*H*-indol-3-yl)propanamido)-5-((*tert*-butyldimethylsilyl)oxy)-4-methylpentanamido)propanamido)-3-methoxy-3-phenylpropanoyl)-D-valyl)-*N*-methyl-D-leucinate (SI-55)

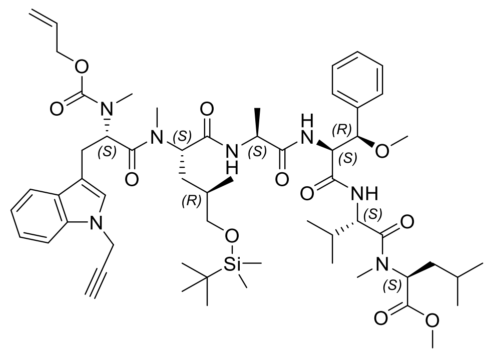

*N*α-((allyloxy)carbonyl)-1-propargyl-L-tryptophan (180 mg, 0.552 mmol) and methyl iodide (207 µl, 3.31 mmol) were dissolved in THF (5.5 ml) and the mixture was cooled to 0 °C. NaH (66.2 mg, 1.66 mmol) was added in one portion and the reaction mixture was slowly warmed to rt. After 17 h, H_2O was added carefully (gas evolution), the resulting mixture was washed with PE (2x) and the PE phase was extracted with 0.5 M NaOH (1x). The aqueous phases were combined, acidified with 1 M HCl and extracted with EtOAc (3x). The combined org phases were washed with 1 M Na_2SO_3 and sat. NaCl soln., dried (Na_2SO_4) and evaporated in vacuo. The crude methylated Trp-derivative was used without further purification.

Pentapeptide **2** was deprotected according to *GP1* and the resulting amine (298 mg, 0.390 mmol) was coupled to crude *N*α-methyl-*N*1-propargyl-Trp (prepared as described above) according to *GP5:* DIPEA (89.0 µl, 507 µmol), $BnNMe_2$ (5.79 µl, 39.0 µmol), 1 M iPrOCOCl soln. (546 µl, 546 µmol), NMI (6.22 µl, 77.9 µmol), 4 M HCl soln. (9.75 µl, 39.0 µmol) (18 h). Flash chromatography (CyH/EtOAc 100:0 – 30:70) followed by lyophilization afforded **SI-55** (352 mg, 0.324 mmol, 83%) as a white, amorphous solid. R_f = 0.30 (PE/EtOAc 4:6).

$[\alpha]^{D}_{20}$ = −91.2 (c 0.5, $CHCl_3$).

^{1}H NMR (500 MHz, $CDCl_3$) *(mixture of rotamers, ratio ~2:2:1)* δ -0.12 – -0.05 (m, 0.8H), -0.04 (s, 0.8H), 0.01 – 0.08 (m, 5.4H), 0.38 (d, *J* = 6.7 Hz, 0.5H), 0.82 (s, 2.0H), 0.83 – 0.89 (m, 10.2H), 0.89 – 1.02 (m, 17.3H), 1.06 (d, *J* = 7.0 Hz, 0.8H), 1.21 – 1.34 (m, 3.4H), 1.45 – 1.54 (m, 2.7H), 1.65 – 1.80 (m, 2.4H), 1.89 – 1.95 (m, 0.8H), 2.06 – 2.17 (m, 1.2H), 2.32 – 2.42 (m, 1.0H), 2.76 – 2.81 (m, 3.0H), 2.89 (s, 1.2H), 2.91 (s, 1.1H), 2.95 – 3.04 (m, 4.3H), 3.15 (s, 0.8H), 3.28 (s, 0.8H), 3.30 – 3.42 (m, 3.8H), 3.42 – 3.53 (m, 1.9H), 3.65 – 3.72 (m, 3.3H), 4.11 – 4.19 (m, 0.5H), 4.20 – 4.32 (m, 0.9H), 4.41 – 4.49 (m, 0.4H), 4.52 – 4.70 (m, 2.9H), 4.70 – 4.88 (m, 4.7H), 5.04 – 5.09 (m, 0.4H), 5.09 – 5.15 (m, 0.8H), 5.15 – 5.31 (m, 2.1H), 5.31 – 5.39 (m, 1.3H), 5.51 (dd, *J* = 9.2, 5.8 Hz, 0.5H), 5.57 – 5.69 (m, 0.3H), 5.81 – 5.98 (m, 0.7H), 6.17 (d, *J* = 7.0 Hz, 0.1H), 6.22 (d, *J* = 6.9 Hz, 0.3H), 6.39 (d, *J* = 6.9 Hz, 0.3H), 6.61 – 6.72 (m, 1.0H), 6.97 – 7.04 (m, 0.6H), 7.04 – 7.09 (m, 0.5H), 7.10 – 7.15 (m, 1.0H), 7.15 – 7.25 (m, 4.4H), 7.26 – 7.39 (m, 4.6H), 7.55 – 7.63 (m, 0.7H), 7.72 (d, *J* = 7.9 Hz, 1H).

^{13}C NMR (126 MHz, $CDCl_3$) *(mixture of rotamers)* δ -5.31, -5.28, -5.2, 15.8, 16.1, 17.3, 17.4, 17.58, 17.65, 17.7, 17.9, 18.41, 18.45, 19.7, 20.0, 21.5, 22.29, 22.32, 22.78, 22.81, 22.83, 23.42, 23.43, 24.9, 25.0, 25.3, 25.4, 26.0, 26.05, 26.07, 26.3, 29.1, 29.6, 29.8, 30.0, 30.4, 30.7, 30.8, 31.0, 31.3, 31.4, 31.46, 31.50, 31.8, 32.2, 32.3, 32.4, 35.7, 35.78, 35.80, 37.1, 49.1, 49.2, 49.6, 52.2, 52.3, 52.7, 53.9, 54.2, 54.6, 54.7, 54.8, 55.6, 56.7, 57.6, 57.7, 57.8, 57.87, 57.95, 66.5, 66.8, 67.0, 67.1, 68.1, 73.61, 73.65, 73.7, 77.8, 77.97, 78.03, 81.26, 81.35, 109.5, 109.6, 109.7, 110.2, 110.7, 110.8, 117.4, 117.8, 118.7, 118.8, 119.1, 119.7, 119.9, 120.1, 122.1, 122.5, 125.9, 126.4, 126.6, 126.97, 127.01, 128.2, 128.3, 128.4, 128.5, 132.4, 132.6, 132.9, 136.10, 136.14, 136.8, 136.9, 155.6, 156.3, 157.4, 168.4, 168.5, 169.1, 170.4, 171.4, 171.9, 172.0, 172.1, 172.2.

Selected diagnostic peaks:

Major rotamers: **^{1}H NMR** (500 MHz, $CDCl_3$) δ 0.03 (s, 3H), 0.03 (s, 3H), 0.04 (s, 3H), 0.04 (s, 3H), 0.88 (s, 9H), 2.78 (s, 3H), 2.99 (s, 3H), 3.33 (s, 3H), 3.69 (s, 3H), 4.16 (t, *J* = 6.9 Hz, 1H), 5.51 (dd, *J* = 9.2, 5.8 Hz, 1H), 6.22 (d, *J* = 6.9 Hz, 1H), 7.07 (s, 1H). **^{13}C NMR** (126 MHz, $CDCl_3$) δ 23.42, 26.05, 81.4, 155.6, 156.3.

Minor rotamer: **^{1}H NMR** (500 MHz, $CDCl_3$) δ -0.06 (s, 3H), -0.04 (s, 3H), 0.38 (d, *J* = 6.7 Hz, 3H), 0.82 (s, 9H), 1.06 (d, *J* = 7.0 Hz, 3H), 2.97 (s, 3H), 3.15 (s, 3H), 3.28 (s, 3H), 4.22 (t, *J* = 6.9 Hz, 1H), 6.39 (d, *J* = 6.9 Hz, 1H).

HRMS (ESI): calcd for $C_{58}H_{88}N_7O_{11}Si^+$ $(M+H)^+$: 1086.6306; found: 1086.6273.

methyl *N*-(((2*S*,3*R*)-2-((*S*)-2-((2*S*,4*R*)-2-((*S*)-2-((*S*)-2-(((allyloxy)carbonyl)amino)-*N*,3-dimethylbutanamido)-*N*-methyl-3-(1-(prop-2-yn-1-yl)-1*H*-indol-3-yl)propanamido)-5-((*tert*-butyldimethylsilyl)oxy)-4-methylpentanamido)propanamido)-3-methoxy-3-phenylpropanoyl)-L-valyl)-*N*-methyl-L-leucinate (SI-56)

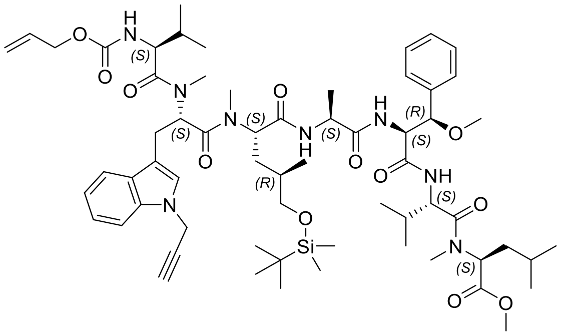

Prepared according to *GP2* and *GP5*: **SI-55** (332 mg, 159 µmol), DMBA (143 mg, 917 µmol), $Pd(PPh_3)_4$ (10.6 mg, 9.17 µmol) (3 h); Alloc-Val-OH (86.0 mg, 428 µmol), 1 M iPrOCOCl (428 µl, 428 µmol), $BnNMe_2$ (4.55 µl, 30.6 µmol), DIPEA (74.8 µl, 428 µmol), NMI (2.44 µl, 30.6 µmol), 4 M HCl (7.65 µl, 30.6 µmol) (17 h). Flash chromatography (CyH/EtOAc 100:0 – 30:70) followed by lyophilization afforded **SI-56** (288 mg, 243 µmol, 79%) as a yellowish, amorphous solid. R_f = 0.36 (PE/EtOAc 3:7).

$[\alpha]_{20}^{D}$ = −93.3 (c 0.5, $CHCl_3$).

^{1}H-NMR (500 MHz, $CDCl_3$) *(mixture of rotamers, ratio ~10:4:1)* δ -0.14 – -0.11 (m, 0.4H), -0.07 (s, 0.8H), -0.05 (s, 0.8H), -0.03 (s, 2.0H), -0.04 (s, 1.9H), 0.38 (d, *J* = 6.6 Hz, 0.6H), 0.44 (d, *J* = 6.8 Hz, 0.2H), 0.81 (s, 2.9H), 0.86 – 0.89 (m, 8.3H), 0.89 – 0.93 (m, 9.0H), 0.94 – 1.01 (m, 8.1H), 1.21 – 1.26 (m, 1.3H), 1.32 – 1.40 (m, 0.9H), 1.44 – 1.56 (m, 1.6H), 1.66 – 1.87 (m, 2.5H), 1.94 – 2.00 (m, 1.0H), 2.07 – 2.15 (m, 0.9H), 2.31 – 2.39 (m, 1H), 2.71 (s, 0.6H), 2.73 (s, 1.4H), 2.79 (s, 0.4H), 2.83 – 2.89 (m, 0.6H), 2.92 – 2.97 (m, 0.7H), 2.97 – 3.00 (m, 2.6H), 3.10 (s, 1.6H), 3.18 – 3.23 (m, 0.4H), 3.3 0 (s, 0.9H), 3.31 – 3.35 (m, 3.2H), 3.47 – 3.54 (m, 1.1H), 3.69 (s, 2.7H), 4.09 – 4.18 (m, 0.5H), 4.33 – 4.41 (m, 0.4H), 4.50 – 4.63 (m, 2.6H), 4.66 (dd, *J* = 7.5, 3.6 Hz, 0.8H), 4.69 – 4.86 (m, 4.2H), 5.07 (dd, *J* = 9.9, 6.0 Hz, 0.6H), 5.23 (d, *J* = 10.3 Hz, 0.9H), 5.29 – 5.39 (m, 1.7H), 5.48 (d, *J* = 9.2 Hz, 0.5H), 5.58 (t, *J* = 7.8 Hz, 0.2H), 5.87 – 6.00 (m, 1.3H), 6.09 (d, *J* = 9.3 Hz, 0.2H), 6.18 (d, *J* = 6.8 Hz, 0.5H), 6.58 (d, *J* = 7.2 Hz, 0.2H), 6.62 (d, *J* = 7.5 Hz, 0.5H), 6.99 (s, 0.3H), 7.06 (s, 0.6H), 7.11 – 7.25 (m, 4.8H), 7.26 – 7.41 (m, 4.2H), 7.43 – 7.49 (m, 1.3H), 7.52 – 7.58 (m, 0.9H), 7.63 – 7.69 (m, 1.2H), 7.73 (d, *J* = 7.9 Hz, 0.6H), 8.21 (d, *J* = 7.0 Hz, 0.2H).

^{13}C NMR (126 MHz, $CDCl_3$) *(mixture of rotamers)* δ -5.31, -5.30, -5.26, 15.9, 16.9, 17.2, 17.3, 17.6, 17.9, 18.40, 18.42, 19.7, 19.9, 21.5, 23.4, 24.9, 25.0, 26.0, 29.0, 30.6, 30.9, 31.1, 31.2, 31.36, 31.40, 31.5, 31.6, 32.0, 32.2, 35.7, 37.0, 38.7, 49.1, 49.8, 52.3, 53.3, 54.2, 54.5, 54.7, 56.0, 56.5, 57.5, 57.7, 57.8, 65.9, 67.0, 68.3, 73.7, 73.8, 78.0, 81.2, 81.3, 109.7, 110.0, 110.4, 117.8, 117.9, 119.0, 119.1, 119.9, 120.1, 122.2, 122.6, 125.7, 126.4, 127.0, 127.1, 128.1, 128.36, 128.44, 128.5, 128.6, 128.7, 132.06, 132.08, 132.19, 132.25, 132.3, 132.8, 133.0, 133.1, 136.05, 136.09, 136.6, 136.8, 156.4, 156.7, 168.3, 168.4, 170.2, 171.4, 171.6, 171.7, 172.11, 172.13, 172.16, 172.22, 173.7.

Selected diagnostic peaks:

Major rotamer: **^{1}H NMR** (500 MHz, $CDCl_3$) δ 0.03 (s, 3H), 0.04 (s, 3H), 0.88 (s, 9H), 2.98 (s, 3H), 3.10 (s, 3H), 3.32 (s, 3H), 3.50 (dd, *J* = 9.7, 4.2 Hz, 1H), 4.13 (t, *J* = 6.9 Hz, 1H), 5.07 (dd, *J* = 9.9, 6.0 Hz, 1H), 5.48 (d, *J* = 9.2 Hz, 1H), 6.18 (d, *J* = 6.8 Hz, 1H), 6.62 (d, *J* = 7.5 Hz, 1H), 7.06 (s, 1H). **^{13}C NMR** (126 MHz, $CDCl_3$) δ 26.0, 73.7, 81.3, 156.4.

Minor rotamer 1: **^{1}H NMR** (500 MHz, $CDCl_3$) δ -0.07 (s, 3H), -0.05 (s, 3H), 0.38 (d, *J* = 6.6 Hz, 3H), 0.81 (s, 9H), 2.71 (s, 3H), 2.98 (s, 3H), 4.37 (t, *J* = 6.9 Hz, 1H), 5.58 (t, *J* = 7.8 Hz, 1H), 6.09 (d, *J* = 9.3 Hz, 1H), 6.58 (d, *J* = 7.2 Hz, 1H), 6.99 (s, 1H). **^{13}C NMR** (126 MHz, $CDCl_3$) δ 73.8, 81.2, 156.7.

Minor rotamer 2: **^{1}H NMR** (500 MHz, $CDCl_3$) δ -0.12 (s, 3H), -0.12 (s, 3H), 0.44 (d, *J* = 6.8 Hz, 3H), 0.79 (s, 9H), 2.66 (s, 3H), 6.95 (s, 1H).

HRMS (ESI): calcd for $C_{63}H_{97}N_8O_{12}Si^+$ $(M+H)^+$: 1185.6990; found: 1185.6991.

(3*S*,6*S*,9*S*,12*S*,15*S*,18*S*,21*S*)-21-((*R*)-3-hydroxy-2-methylpropyl)-9-isobutyl-6,12-diisopropyl-15-((*R*)-methoxy(phenyl)methyl)-1,4,10,18-tetramethyl-3-((1-(prop-2-yn-1-yl)-1*H*-indol-3-yl)methyl)-1,4,7,10,13,16,19-heptaazacyclohenicosane-2,5,8,11,14,17,20-heptaone (26)

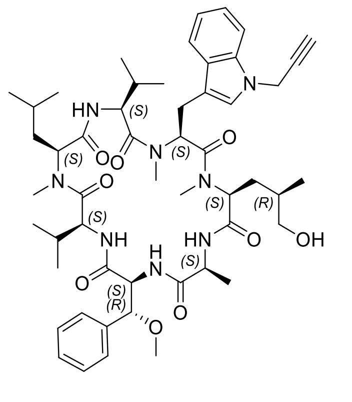

Prepared according to modified *GP6a*: **SI-56** (84.0 mg, 70.8 µmol), 1 M LiOH (92.2 µl, 92.2 µmol) (5 h); $Pd(OAc)_2$ (0.64 mg, 2.86 µmol), TPPTS (1.6 mg, 2.9 µmol), Et_2NH (37.5 µl, 359 µmol) (7 h); HATU (109 mg, 287 µmol), DIPEA (62.6 µl, 359 µmol) (addition over 2.5 h at 40 °C, additional 1 h at 40 °C); NH_4F (53.1 mg, 1.43 mmol) (14.5 h). RP flash chromatography (H_2O/MeCN 80:20 – 5:95) followed by lyophilization afforded **26** (22.6 mg, 23.7 µmol, 33%) as a white, amorphous solid.

$[\boldsymbol{\alpha}]^{\mathbf{D}}_{\mathbf{24}}$ = −106.1 (c 0.2, $CHCl_3$).

^{1}H NMR (500 MHz, $CDCl_3$) δ -0.39 – -0.27 (m, 1H), 0.31 (d, *J* = 6.8 Hz, 3H), 0.44 (d, *J* = 6.6 Hz, 3H), 0.63 (d, *J* = 6.8 Hz, 3H), 0.78 – 0.87 (m, 2H), 0.91 – 1.01 (m, 10H), 1.05 – 1.13 (m, 7H), 1.54 – 1.64 (m, 1H), 1.85 – 1.96 (m, 1H), 2.21 – 2.29 (m, 1H), 2.30 – 2.37 (m, 2H), 2.41 (t, *J* = 2.7 Hz, 1H), 2.61 (s, 3H), 2.85 (s, 3H), 2.92 – 2.99 (m, 1H), 3.01 – 3.08 (m, 1H), 3.15 (dd, *J* = 13.9, 5.8 Hz, 1H), 3.37 (s, 3H), 3.40 (s, 3H), 3.42 – 3.53 (m, 1H), 4.45 – 4.56 (m, 2H), 4.71 – 4.81 (m, 4H), 4.83 – 4.90 (m, 2H), 5.15 (d, *J* = 5.7 Hz, 1H), 5.40 (dd, *J* = 9.7, 5.8 Hz, 1H), 6.93 (d, *J* = 4.7 Hz, 1H), 6.98 (s, 1H), 7.10 – 7.16 (m, 1H), 7.21 – 7.29 (m, 4H), 7.31 – 7.40 (m, 2H), 7.47 (d, *J* = 7.9 Hz, 1H), 8.13 – 8.21 (m, 2H), 8.51 (d, *J* = 9.3 Hz, 1H).

^{13}C NMR (126 MHz, $CDCl_3$) δ 17.4, 18.8, 19.49, 19.53, 20.2, 20.3, 22.7, 23.7, 25.4, 26.7, 29.0, 29.7, 31.0, 31.8, 32.37, 32.44, 33.0, 35.8, 39.1, 50.9, 53.4, 54.4, 55.5, 55.9, 57.9, 59.0, 59.2, 66.0, 74.1, 77.5, 80.1, 109.4, 109.9, 119.0, 120.3, 122.8, 125.9, 128.1, 128.3, 128.4, 129.0, 135.1, 136.0, 168.5, 169.2, 169.8, 170.7, 171.5, 171.9, 174.2.

HRMS (ESI): calcd for $C_{52}H_{75}N_8O_9^+$ $(M+H)^+$: 955.5652; found: 955.5614.

(3*S*,3'*S*,6*S*,6'*S*,9*S*,9'*S*,12*S*,12'*S*,15*S*,15'*S*,18*S*,18'*S*,21*S*,21'*S*)-21,21'-((((pentane-1,5-diylbis(1H-1,2,3-triazole-1,4-diyl))bis(methylene))bis(1*H*-indole-1,3-diyl))bis(methylene))bis(18-((*R*)-3-hydroxy-2-methylpropyl)-6-isobutyl-3,9-diisopropyl-12-((*R*)-methoxy(phenyl)methyl)-1,7,15,19-tetramethyl-1,4,7,10,13,16,19-heptaazacyclohenicosane-2,5,8,11,14,17,20-heptaone) (SI-57)

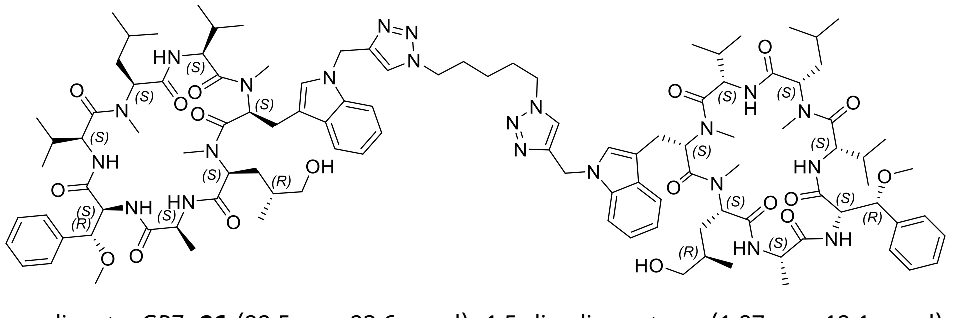

Prepared according to *GP7*: **26** (22.5 mg, 23.6 µmol), 1,5-diazdiopentane (1.87 mg, 12.1 µmol), 1 M $CuSO_4$ (9.72 µl, 9.72 µmol), 1 M sodium ascorbate (15.8 µmol, 15.8 µmol) (17 h). RP flash chromatography (H_2O/MeCN 90:10 – 5:95) followed by prep HPLC (H_2O/MeCN 75:25 – 5:95) and lyophilization afforded **SI-57** (8.2 mg, 3.97 µmol, 33%) as a white, amorphous solid.

$[\boldsymbol{\alpha}]^{\mathbf{D}}_{\mathbf{23}}$ = −103.5 (c 0.2, $CHCl_3$).

^{1}H NMR (500 MHz, $CDCl_3$) δ -0.66 – -0.41 (m, 2H), 0.17 (d, *J* = 6.7 Hz, 6H), 0.41 (d, *J* = 6.7 Hz, 6H), 0.63 (d, *J* = 6.9 Hz, 6H), 0.85 – 1.02 (m, 20H), 1.04 – 1.17 (m, 16H), 1.26 – 1.33 (m, 2H), 1.51 – 1.60 (m, 2H), 1.70 – 1.76 (m, 2H),

1.80 – 1.92 (m, 4H), 2.18 – 2.37 (m, 4H), 2.53 (s, 6H), 2.69 – 2.80 (m, 2H), 2.85 (s, 6H), 2.99 (dd, *J* = 10.0, 4.8 Hz, 2H), 3.15 (dd, *J* = 14.1, 5.7 Hz, 2H), 3.33 – 3.38 (m, 12H), 3.42 (dd, *J* = 14.2, 9.6 Hz, 2H), 4.18 – 4.29 (m, 4H), 4.48 (t, *J* = 8.3 Hz, 2H), 4.55 (dd, *J* = 11.0, 3.2 Hz, 2H), 4.66 – 4.77 (m, 4H), 4.81 – 4.90 (m, 4H), 5.14 (d, *J* = 5.5 Hz, 2H), 5.31 (q, *J* = 15.7 Hz, 5H), 6.91 (d, *J* = 4.6 Hz, 2H), 6.95 – 7.00 (m, 2H), 7.07 – 7.12 (m, 2H), 7.18 – 7.26 (m, 14H), 7.38 – 7.41 (m, 4H), 7.45 (d, *J* = 7.9 Hz, 2H), 8.00 (d, *J* = 10.1 Hz, 2H), 8.13 (d, *J* = 7.6 Hz, 3H), 8.47 (d, *J* = 9.5 Hz, 2H).

^{13}C NMR (126 MHz, $CDCl_3$) δ 16.7, 18.9, 19.4, 19.5, 20.21, 20.23, 22.6, 23.5, 23.7, 25.4, 26.7, 28.9, 29.5, 29.6, 31.0, 31.8, 32.27, 32.29, 32.6, 39.1, 41.6, 50.0, 50.8, 53.6, 54.5, 55.6, 55.9, 57.9, 58.9, 59.0, 66.6, 80.1, 109.3, 110.0, 118.8, 120.0, 122.2, 122.6, 126.5, 127.9, 128.3, 128.5, 129.0, 135.1, 136.2, 144.0, 168.5, 169.0, 169.7, 170.7, 171.7, 172.1, 174.2.

HRMS (ESI): calcd for $C_{109}H_{159}N_{22}O_{18}^+$ $(M+H)^+$: 2064.2197; found:2064.2219.

(3*S*,3'*S*,6*S*,6'*S*,9*S*,9'*S*,12*S*,12'*S*,15*S*,15'*S*,18*S*,18'*S*,21*S*,21'*S*)-21,21'-(((((3,6,9,12-tetraoxatetradecane-1,14-diyl)bis(1*H*-1,2,3-triazole-1,4-diyl))bis(methylene))bis(1*H*-indole-1,3-diyl))bis(methylene))bis(18-((*R*)-3-hydroxy-2-methylpropyl)-6-isobutyl-3,9-diisopropyl-12-((*R*)-methoxy(phenyl)methyl)-1,7,15,19-tetramethyl-1,4,7,10,13,16,19-heptaazacyclohenicosane-2,5,8,11,14,17,20-heptaone) (27)

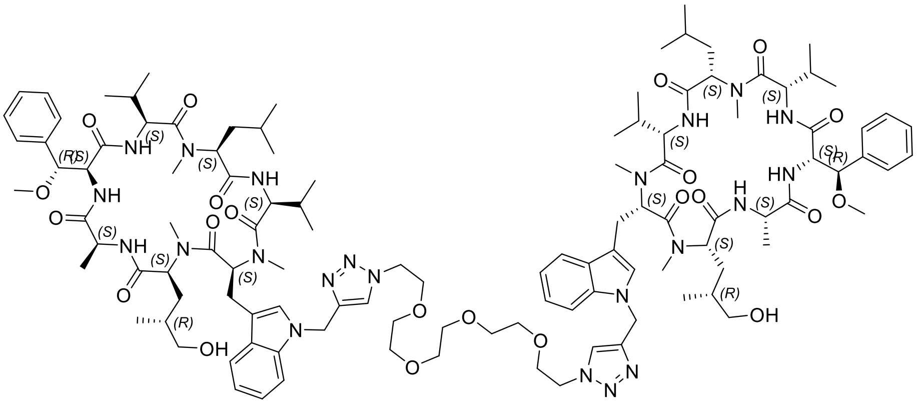

Prepared according to *GP7*: **26** (11.1 mg, 11.6 µmol), 1,14-diazido-3,6,9,12-tetraoxatetradecane (1.68 mg, 5.81 µmol), 1 M $CuSO_4$ (5.81 µl, 5.81 µmol), 1 M sodium ascorbate (6.97 µl, 6.97 µmol). RP flash chromatography (H_2O/MeCN 90:10 – 5:95) followed by prep HPLC (H_2O/MeCN 60:40 – 5:95) and lyophilization afforded **27** (6.3 mg, 2.87 µmol, 49%) as a white, amorphous solid.

$[\alpha]^{D}_{23}$ = −69.1 (c 0.5, $CHCl_3$).

^{1}H NMR (500 MHz, $CDCl_3$) δ -0.52 – -0.40 (m, 2H), 0.21 (d, *J* = 6.7 Hz, 6H), 0.42 (d, *J* = 6.6 Hz, 6H), 0.62 (d, *J* = 6.9 Hz, 6H), 0.85 – 0.92 (m, 4H), 0.93 (d, *J* = 6.7 Hz, 6H), 0.96 (d, *J* = 6.6 Hz, 6H), 0.99 (d, *J* = 6.7 Hz, 6H), 0.99 – 1.06 (m, 2H), 1.07 – 1.17 (m, 14H), 1.49 – 1.60 (m, 2H), 1.73 – 1.81 (m, 2H), 2.21 – 2.27 (m, 2H), 2.31 (ddd, *J* = 13.4, 10.5, 4.7 Hz, 2H), 2.56 (s, 6H), 2.81 – 2.86 (m, 8H), 3.01 (dd, *J* = 10.8, 4.7 Hz, 2H), 3.13 (dd, *J* = 14.1, 5.9 Hz, 2H), 3.34 – 3.40 (m, 12H), 3.41 – 3.47 (m, 2H), 3.47 – 3.55 (m, 12H), 3.76 – 3.82 (m, 4H), 4.41 – 4.46 (m, 4H), 4.45 – 4.55 (m, 2H), 4.70 – 4.79 (m, 4H), 4.82 – 4.91 (m, 4H), 5.14 (d, *J* = 5.6 Hz, 2H), 5.25 – 5.38 (m, 6H), 6.92 (d, *J* = 4.7 Hz, 2H), 6.98 (s, 2H), 7.05 – 7.11 (m, 2H), 7.15 – 7.27 (m, 14H), 7.38 (d, *J* = 8.2 Hz, 2H), 7.43 – 7.47 (m, 2H), 7.55 (s, 2H), 8.05 (d, *J* = 10.1 Hz, 2H), 8.14 (d, *J* = 7.6 Hz, 2H), 8.50 (d, *J* = 9.5 Hz, 2H).

^{13}C NMR (126 MHz, $CDCl_3$) δ 16.9, 18.8, 19.45, 19.50, 20.2, 20.3, 22.7, 23.7, 25.4, 26.8, 29.0, 29.6, 31.0, 31.8, 32.3, 32.4, 32.7, 39.1, 41.6, 50.38, 50.42, 50.8, 53.5, 54.4, 55.6, 55.9, 57.9, 59.0, 59.1, 66.4, 69.4, 70.49, 70.54, 80.1, 109.2, 110.1, 118.9, 120.0, 122.6, 123.4, 126.5, 128.0, 128.3, 128.5, 129.0, 135.1, 136.2, 143.6, 168.5, 169.0, 169.7, 170.7, 171.6, 172.1, 174.2.

HRMS (ESI): calcd for $C_{114}H_{169}N_{22}O_{22}^+$ $(M+H)^+$: 2198.2776; found:2198.2672.

Methyl *N*-(((2*S*,3*R*)-2-((*S*)-2-((2*S*,4*R*)-2-((*S*)-3-(1-allyl-1*H*-indol-3-yl)-2-(((allyloxy)carbonyl)(methyl)amino)-*N*-methylpropanamido)-5-((*tert*-butyldimethylsilyl)oxy)-4-methylpentanamido)propanamido)-3-methoxy-3-phenylpropanoyl)-L-valyl)-*N*-methyl-L-leucinate (SI-58)

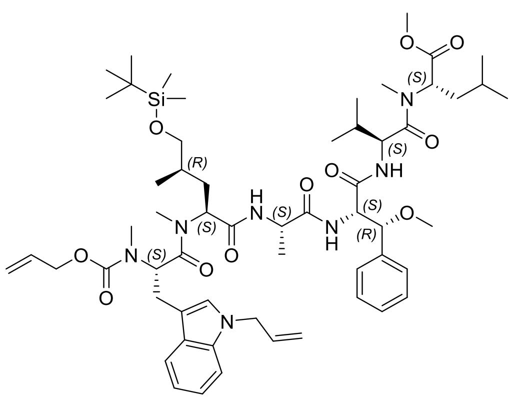

1-Allyl-*N*α-((allyloxy)carbonyl)-L-tryptophan (**SI-41**) (146 mg, 417 µmol) and methyl iodide (167 µl, 2.67 mmol) were dissolved in THF (4.5 ml) and cooled to 0 °C. NaH (71.1 mg, 1.78 mmol) was added in one portion and the reaction mixture was slowly warmed to rt over night. After 16 h, H_2O was added carefully (gas evolution), the resulting mixture was washed with PE, the PE phase was extracted with 0.5 M NaOH (1x). The combined aq. phases were acidified with 1 M HCl and extracted with EtOAc (3x). The combined EtOAc-phases were washed with 1 M Na_2SO_3 and brine, dried (Na_2SO_4) and evaporated in vacuo. The crude methylated Trp-derivative was then used in the peptide coupling without further purification.

Pentapeptide **2** (286 mg, 318 µmol) was deprotected according to *GP1* and the resulting amine was coupled to crude *Nα*-methyl-*N1*-allyl-Trp according to *GP5:* DIPEA (75.2 µl, 413 µmol), $BnNMe_2$ (4.78 µl, 31.8 µmol), 1 M iPrOCOCl (413 µl, 413 µmol), NMI (2.84 µl, 31.8 µmol), 4 M HCl (7.95 µl, 31.8 µmol) (3 h). Flash chromatography (CyH/EtOAc 100:0 – 30:70) followed by lyophilization afforded **SI-58** (300 mg, 276 µmol, 87%) as a white, amorphous solid. R_f = 0.44 (PE/EtOAc 3:7).

$[\boldsymbol{\alpha}]_{20}^{D}$ = −85.2 (c 1.0, $CHCl_3$).

^{1}H NMR (500 MHz, $CDCl_3$) *(mixture of rotamers, ratio ~3:1)* δ -0.06 (s, 0.6H), -0.04 (s, 0.8H), -0.01 – 0.08 (m, 4.7H), 0.40 (d, *J* = 6.6 Hz, 0.5H), 0.82 (s, 1.8H), 0.84 – 1.03 (m, 23.4H), 1.10 (d, *J* = 7.0 Hz, 0.7H), 1.21 – 1.27 (m, 1.5H), 1.32 (d, *J* = 7.2 Hz, 0.7H), 1.43 – 1.55 (m, 2.3H), 1.65 – 1.81 (m, 2.3H), .1.83 – 1.99 (m, 1.8H), 2.05 – 2.18 (m, 1.2H), 2.75 – 2.85 (m, 3.1H), 2.88 (s, 0.9H), 2.90 (s, 0.91H), 2.94 – 3.02 (m, 3.1H), 3.03 – 3.10 (m, 0.8H), 3.14 (s, 0.7H), 3.28 (s, 0.7H), 3.30 – 3.35 (m, 2.4H), 3.35 – 3.52 (m, 2.4H), 3.69 (s, 3.0H), 4.12 – 4.30 (m, 1.3H), 4.37 – 4.45 (m, 0.4H), 4.50 – 4.71 (m, 4.2H), 4.72 – 4.86 (m, 2.2H), 4.97 – 5.30 (m, 4.7H), 5.31 – 5.39 (m, 1.2H), 5.48 – 5.53 (m, 0.4H), 5.82 – 5.97 (m, 1.5H), 6.21 (d, *J* = 7.2 Hz, 0.1H), 6.26 (d, *J* = 7.0 Hz, 0.3H), 6.41 (d, *J* = 6.7 Hz, 0.3H), 6.61 – 6.74 (m, 0.9H), 6.87 – 6.93 (m, 0.5H), 6.97 (s, 0.4H), 7.06 – 7.13 (m, 1.0H), 7.14 – 7.22 (m, 3.0H), 7.22 – 7.40 (m, 5.7H), 7.55 – 7.63 (m, 0.7H), 7.70 (d, *J* = 7.8 Hz, 0.3H).

^{13}C NMR (126 MHz, $CDCl_3$) *(mixture of rotamers)* δ -5.30 -5.27, -5.2, 15.7, 17.3, 17.4, 17.5, 17.6, 17.7, 17.85, 17.95, 18.4, 18.5, 19.66, 19.70, 21.5, 23.4, 24.9, 25.0, 25.2, 25.3, 26.0, 26.1, 29.1, 29.6, 30.0, 30.5, 30.8, 30.9, 31.1, 31.3, 31.4, 31.46, 31.51, 31.8, 32.2, 32.30, 32.35, 37.1, 48.77, 48.81, 48.9, 49.1, 49.3, 49.6, 52.3, 54.2, 54.59, 54.64, 54.7, 54.9, 55.6, 56.6, 57.6, 57.7, 57.8, 57.9, 66.4, 66.5, 66.7, 66.8, 67.1, 67.2, 68.1, 81.27, 81.34, 109.5, 109.8, 109.89, 109.94, 110.0, 117.2, 117.3, 117.7, 117.8, 118.6, 118.7, 118.9, 119.2, 119.4, 119.6, 121.8, 122.1, 126.4, 126.8, 126.98, 127.02, 127.1, 128.1, 128.2, 128.3, 128.37, 128.44, 128.5, 132.4, 132.6, 132.9, 133.5, 133.6, 133.7, 136.4, 136.5, 136.9, 155.6, 156.4, 157.4, 168.5, 168.5, 169.1, 170.5, 171.5, 171.6, 172.06, 172.10, 172.2.

Selected diagnostic peaks:

Major rotamers: **^{1}H NMR** (500 MHz, $CDCl_3$) δ 0.03 (s, 3H), 0.03 (s, 3H), 0.04 (s, 3H), 0.04 (s, 3H), 0.88 (s, 9H), 2.81 (s, 3H), 2.98 (s, 3H), 2.99 (s, 3H), 3.33 (s, 3H), 3.69 (s, 3H), 4.41 (dd, *J* = 13.0, 6.0 Hz, 1H), 5.48 – 5.54 (m, 1H), 6.26 (d, *J* = 7.0 Hz, 1H), 6.89 (s, 1H), 6.97 (s, 1H), 7.70 (d, *J* = 7.8 Hz, 1H). **^{13}C NMR** (126 MHz, $CDCl_3$) δ -5.27, 19.70, 26.1, 81.34, 155.6, 156.4.

Minor rotamer: **^{1}H NMR** (500 MHz, $CDCl_3$) δ -0.06 (s, 3H), -0.04 (s, 3H), 0.40 (d, *J* = 6.6 Hz, 3H), 0.82 (s, 9H), 1.10 (d, *J* = 7.0 Hz, 3H), 2.77 (s, 3H), 3.14 (s, 3H), 3.28 (s, 3H), 6.21 (d, *J* = 7.2 Hz, 0H), 6.91 (s, 1H). **^{13}C NMR** (126 MHz, $CDCl_3$) δ -5.30, 19.66, 26.0, 81.3, 157.4.

HRMS (ESI) calcd for $C_{58}H_{90}N_7O_{11}Si^+$ $(M+H)^+$: 1088.6462; found: 1088.6470.

methyl *N*-(((2*S*,3*R*)-2-((*S*)-2-((2*S*,4*R*)-2-((*S*)-3-(1-allyl-1*H*-indol-3-yl)-2-((*S*)-2-(((allyloxy)carbonyl)amino)-*N*,3-dimethylbutanamido)-*N*-methylpropanamido)-5-((*tert*-butyldimethylsilyl)oxy)-4-methylpentanamido)propanamido)-3-methoxy-3-phenylpropanoyl)-L-valyl)-*N*-methyl-L-leucinate (SI-59)

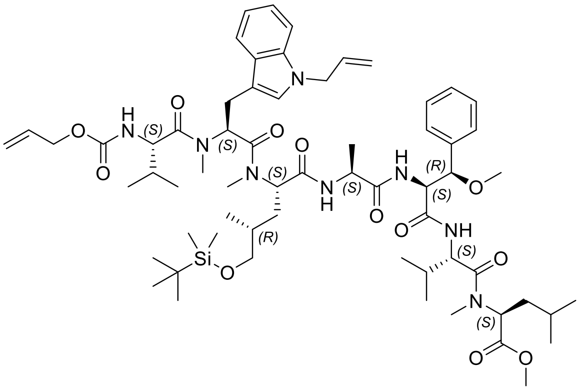

Prepared according to *GP2* and *GP5*: **SI-58** (284 mg, 261 µmol), DMBA (122 mg, 783 µmol), $Pd(PPh_3)_4$ (9.1 mg, 7.8 µmol) (1.5 h); Alloc-L-Val-OH (73.5 mg, 365 µmol), DIPEA (63.8 µl, 365 µmol), $BnNMe_2$ (3.88 µl, 26.1 µmol), 1 M iPrOCOCl (339 µl, 339 µmol), NMI (4.16 µl, 52.2 µmol), 4 M HCl (6.53 µl, 26.1 µmol) (16 h). Flash chromatography (CyH/EtOAc 100:0 – 40:60) followed by lyophilization afforded **SI-59** (250 mg, 179 µmol, 69%, 85% purity) as a white amorphous solid. R_f = 0.26 (PE/EtOAc 4:6).

$[\alpha]^{D}_{24}$ = −88.9 (c 0.5, $CHCl_3$).

^{1}H NMR (500 MHz, $CDCl_3$) *(mixture of rotamers, ratio ~2:1:1)* δ -0.07 (s, 0.7H), -0.05 (s, 0.7H), -0.02 – 0.08 (m, 4.6H), 0.39 (d, *J* = 6.6 Hz, 0.7H), 0.80 – 0.84 (m, 2.9H), 0.84 – 1.02 (m, 27.1H), 1.21 – 1.27 (m, 1.7H), 1.31 – 1.34 (m, 0.5H), 1.34 – 1.41 (m, 0.7H), 1.43 – 1.56 (m, 1.9H), 1.66 – 1.80 (m, 2.2H), 1.81 – 1.89 (m, 1.9H), 1.90 – 2.01 (m, 1.2H), 2.01 – 2.17 (m, 1.4H), 2.25 – 2.30 (m, 0.2H), 2.67 – 2.72 (m, 0.7H), 2.74 – 2.80 (m, 1.6H), 2.80 – 2.89 (m, 1.2H), 2.84 – 3.02 (m, 3.4H), 3.03 – 3.08 (m, 0.5H), 3.10 (s, 1.3H), 3.17 – 3.25 (m, 0.5H), 3.27 – 3.36 (m, 4.1H), 3.36 – 3.44 (m, 0.8H), 3.44 – 3.52 (m, 1.1H), 3.69 (s, 3.0H), 4.10 – 4.20 (m, 0.5H), 4.26 (d, *J* = 5.5 Hz, 0.1H), 4.32 – 4.43 (m, 0.5H), 4.48 – 4.72 (m, 5.0H), 4.72 – 4.76 (m, 0.4H), 4.77 – 4.87 (m, 2.2H), 4.98 – 5.11 (m, 1.3H), 5.11 – 5.27 (m, 2.1H), 5.27 – 5.39 (m, 1.8H), 5.47 (d, *J* = 9.3 Hz, 0.4H), 5.58 (t, *J* = 7.7 Hz, 0.2H), 5.84 – 5.99 (m, 2.0H), 6.09 (d, *J* = 9.3 Hz, 0.2H), 6.17 (d, *J* = 6.9 Hz, 0.4H), 6.59 (d, *J* = 7.3 Hz, 0.2H), 6.63 – 6.72 (m, 0.7H), 6.87 (s, 0.2H), 6.89 – 6.98 (m, 0.7H), 7.07 – 7.12 (m, 0.9H), 7.13 – 7.25 (m, 4.5H), 7.27 – 7.36 (m, 2.8H), 7.43 – 7.48 (m, 0.5H), 7.51 – 7.57 (m, 0.5H), 7.63 – 7.75 (m, 1.0H), 8.22 (d, *J* = 7.2 Hz, 0.2H).

^{13}C NMR (126 MHz, $CDCl_3$) *(mixture of rotamers)* δ -5.30, -5.29, 15.8, 16.9, 17.2, 17.3, 17.5, 17.8, 18.39, 18.44, 19.7, 19.9, 21.5, 23.4, 25.0, 26.0, 26.1, 30.7, 30.8, 30.9, 31.26, 31.33, 31.4, 31.5, 31.6, 32.2, 32.4, 37.1, 48.8, 48.9, 48.96, 49.05, 49.7, 52.3, 53.3, 54.2, 54.61, 54.65, 56.0, 57.5, 57.7, 57.8, 65.9, 67.1, 68.2, 81.27, 81.34, 109.3, 109.7, 109.8, 117.3, 117.75, 117.84, 118.8, 118.9, 119.5, 119.7, 121.8, 122.2, 126.1, 126.8, 127.0, 127.1, 128.1, 128.35, 128.41, 128.44, 128.5, 128.58, 128.60, 128.7, 132.05, 132.09, 132.2, 132.3, 132.8, 133.1, 133.5, 133.6, 136.4, 136.6, 136.9, 156.4, 156.6, 168.4, 168.5, 170.2, 171.4, 171.5, 171.7, 172.08, 172.10, 172.12, 172.17, 172.23, 173.6.

Selected diagnostic peaks:

Major rotamer: **^{1}H NMR** (500 MHz, $CDCl_3$) δ 0.03 (s, 3H), 0.04 (s, 3H), 0.88 (s, 9H), 2.98 (s, 3H), 3.32 (s, 3H), 3.69 (s, 3H), 5.47 (d, *J* = 9.3 Hz, 1H), 6.17 (d, *J* = 6.9 Hz, 1H), 6.66 (d, *J* = 7.5 Hz, 1H), 6.94 (s, 1H). **^{13}C NMR** (126 MHz, $CDCl_3$) δ 26.0, 81.34, 156.4.

Minor rotamers: **^{1}H NMR** (500 MHz, $CDCl_3$) δ -0.07 (s, 3H), -0.05 (s, 3H), 0.02 (s, 3H), 0.03 (s, 3H), 0.39 (d, *J* = 6.6 Hz, 3H), 0.82 (s, 9H), 2.70 (s, 3H), 3.10 (s, 3H), 3.30 (s, 3H), 3.31 (s, 3H), 5.58 (t, *J* = 7.7 Hz, 1H), 6.09 (d, *J* = 9.3 Hz, 1H), 6.59 (d, *J* = 7.3 Hz, 1H), 6.87 (s, 1H), 8.22 (d, *J* = 7.2 Hz, 1H). **^{13}C NMR** (126 MHz, $CDCl_3$) δ 26.1, 81.27, 156.7.

HRMS (ESI) calcd for $C_{63}H_{99}N_8O_{13}Si^+$ $(M+H)^+$: 1187.7146; found: 1187.7156.

(3*S*,6*S*,9*S*,12*S*,15*S*,18*S*,21*S*)-3-((1-allyl-1*H*-indol-3-yl)methyl)-21-((*R*)-3-hydroxy-2-methylpropyl)-9-isobutyl-6,12-diisopropyl-15-((*R*)-methoxy(phenyl)methyl)-1,4,10,18-tetramethyl-1,4,7,10,13,16,19-heptaazacyclohenicosane-2,5,8,11,14,17,20-heptaone (SI-60)

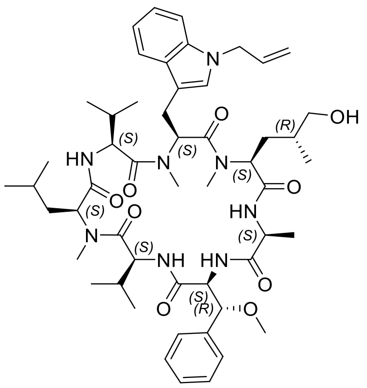

Prepared according to modified *GP6c*: **SI-59** (60.3 mg, 50.8 µmol), 1 M LiOH (76.0 µl, 76.0 µmol) (6 h); $Pd(OAc)_2$ (0.5 mg, 2.0 µmol), TPPTS (2.3 mg, 4.1 µmol), Et_2NH (26.5 µl, 254 µmol) (3 h); HATU (57.9 mg, 152 µmol), DIPEA (35.5 µl, 203 µmol) (addition over 3 h, additional 16 h); NH_4F (56.4 mg, 1.52 mmol) (15 h). RP flash chromatography (H_2O/MeCN 90:10 – 5:95) yielded **SI-60** (18.0 mg, 18-8 µmol, 37%) as an off-white amorphous solid.

$[\boldsymbol{\alpha}]^{\mathbf{D}}_{\mathbf{24}}$ = −90.3 (c 0.5, $CHCl_3$).

^{1}H NMR (500 MHz, $CDCl_3$) δ -0.36 – -0.19 (m, 1H), 0.34 (d, *J* = 6.7 Hz, 3H), 0.43 (d, *J* = 6.5 Hz, 3H), 0.63 (d, *J* = 6.8 Hz, 3H), 0.84 – 0.89 (m, 1H), 0.92 – 0.97 (m, 6H), 0.97 – 1.01 (m, 4H), 1.08 – 1.12 (m, 6H), 1.12 – 1.16 (m, 1H), 1.55 (dt, *J* = 13.2, 6.5 Hz, 1H), 1.94 (ddd, *J* = 13.4, 10.8, 6.9 Hz, 1H), 2.18 – 2.37 (m, 3H), 2.61 (s, 3H), 2.84 (s, 3H), 2.97 (dd, *J* = 11.3, 5.4 Hz, 1H), 3.06 (dd, *J* = 11.2, 4.3 Hz, 1H), 3.16 (dd, *J* = 14.0, 6.4 Hz, 1H), 3.36 (s, 3H), 3.37 (s, 3H), 3.42 (dd, *J* = 14.3, 9.2 Hz, 1H), 4.47 – 4.53 (m, 2H), 4.59 – 4.65 (m, 2H), 4.71 – 4.80 (m, 2H), 4.80 – 4.90 (m, 2H), 5.10 (dd, *J* = 17.1, 1.4 Hz, 1H), 5.14 (d, *J* = 5.6 Hz, 1H), 5.19 (dd, *J* = 10.2, 1.4 Hz, 1H), 5.42 (dd, *J* = 9.2, 6.4 Hz, 1H), 5.91 (ddt, *J* = 16.0, 10.7, 5.6 Hz, 1H), 6.84 (s, 1H), 6.94 (d, *J* = 4.8 Hz, 1H), 7.04 – 7.12 (m, 1H), 7.15 – 7.26 (m, 6H), 7.27 – 7.29 (m, 1H), 7.45 (d, *J* = 7.9 Hz, 1H), 8.14 – 8.21 (m, 2H), 8.49 (d, *J* = 9.3 Hz, 1H).

^{13}C NMR (126 MHz, $CDCl_3$) δ 17.4, 18.8, 19.50, 19.53, 20.2, 20.3, 22.6, 23.7, 25.4, 26.7, 29.0, 29.6, 31.0, 32.0, 32.3, 32.4, 33.1, 39.1, 49.0, 50.9, 53.5, 54.4, 55.5, 55.9, 57.9, 59.0, 59.2, 66.2, 80.1, 108.8, 110.2, 118.0, 118.8, 119.8, 122.4, 126.3, 127.9, 128.3, 128.4, 129.0, 133.2, 135.1, 136.4, 168.5, 169.2, 169.8, 170.7, 171.6, 172.1, 174.3.

HRMS (ESI) calcd for $C_{52}H_{77}N_8O_9^+$ $(M+H)^+$: 957.5808; found: 957.5813.

(3*S*,3'*S*,6*S*,6'*S*,9*S*,9'*S*,12*S*,12'*S*,15*S*,15'*S*,18*S*,18'*S*,21*S*,21'*S*)- 21,21'-((((*E*)-but-2-ene-1,4-diyl)bis(1*H*-indole-1,3-diyl))bis(methylene))bis(18-((*R*)-3-hydroxy-2-methylpropyl)-6-isobutyl-3,9-diisopropyl-12-((*R*)-methoxy(phenyl)methyl)-1,7,15,19-tetramethyl-1',4',7',10',13',16',19'-heptaazacyclohenicosane-2,5,8,11,14,17,20-heptaone) (28)

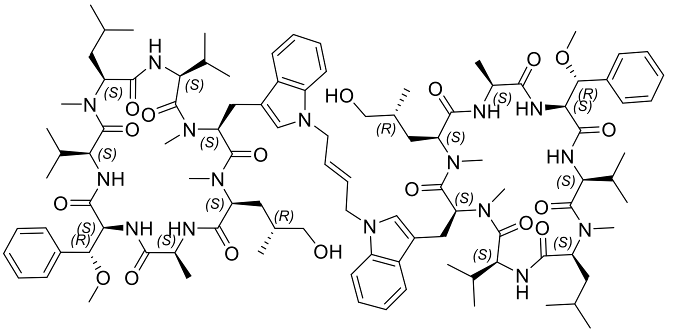

A Schlenk flask was charged with **SI-60** (32.5 mg, 34.0 µmol) and Grubbs catalyst 2nd Gen (3.5 mg, 4.1 µmol). The flask was evacuated and back-filled with argon three times. Freshly degassed (3x freeze-pump-thaw) DCM (0.27 ml) was added and the mixture was stirred at rt. After 64 h, LC/MS indicated ~60% conversion. The mixture was evaporated in vacuo and the residue was purified by RP flash chromatography (H_2O/MeCN 90:10 – 5:95) and prep HPLC (H_2O/MeCN 65:35 – 5:95), which after lyophilization yielded **28** (9.1 mg, 4.8 µmol, 28%) as a white, amorphous solid.

$[\boldsymbol{\alpha}]^{\mathbf{D}}_{\mathbf{20}}$ = −112.4 (c 0.2, $CHCl_3$).

^{1}H NMR (500 MHz, $CDCl_3$) δ -0.48 – -0.29 (m, 2H), 0.26 (d, *J* = 6.7 Hz, 6H), 0.43 (d, *J* = 6.6 Hz, 6H), 0.62 (d, *J* = 6.8 Hz, 6H), 0.83 – 0.87 (m, 2H), 0.92 – 0.96 (m, 12H), 0.98 (d, *J* = 6.8 Hz, 6H), 1.00 – 1.04 (m, 2H), 1.08 – 1.12 (m, 12H), 1.12 – 1.15 (m, 2H), 1.51 – 1.58 (m, 2H), 1.81 – 1.87 (m, 4H), 2.21 – 2.27 (m, 2H), 2.28 – 2.35 (m, 2H), 2.55 (s, 6H), 2.85 (s, 6H), 2.89 (dd, *J* = 11.2, 5.5 Hz, 2H), 3.01 (dd, *J* = 11.2, 4.5 Hz, 2H), 3.13 (dd, *J* = 14.2, 5.9 Hz, 2H), 3.36 – 3.38 (m, 12H), 3.39 – 3.44 (m, 2H), 4.46 – 4.52 (m, 4H), 4.59 – 4.63 (m, 4H), 4.70 – 4.79 (m, 4H), 4.82 – 4.87 (m, 4H), 5.14 (d, *J* = 5.6 Hz, 2H), 5.36 (dd, *J* = 9.5, 6.1 Hz, 2H), 5.66 (t, *J* = 2.8 Hz, 2H), 6.80 (s, 2H), 6.91 (d, *J* = 4.7 Hz, 2H), 7.05 – 7.12 (m, 2H), 7.14 – 7.19 (m, 2H), 7.20 – 7.24 (m, 8H), 7.25 – 7.26 (m, 4H), 7.44 (d, *J* = 7.9 Hz, 2H), 8.09 (d, *J* = 10.1 Hz, 2H), 8.16 (d, *J* = 7.5 Hz, 2H), 8.48 (d, *J* = 9.4 Hz, 2H).

^{13}C NMR (126 MHz, $CDCl_3$) δ 17.2, 18.8, 19.49, 19.53, 20.2, 20.3, 22.7, 23.7, 25.4, 26.7, 28.9, 29.6, 31.0, 31.9, 32.3, 32.4, 32.9, 39.1, 47.6, 50.9, 53.5, 54.4, 55.5, 55.9, 57.9, 59.0, 59.1, 66.3, 80.1, 109.1, 110.1, 118.9, 120.0, 122.6, 126.4, 128.0, 128.3, 128.5, 128.6, 129.0, 135.1, 136.2, 168.5, 169.0, 169.8, 170.7, 171.5, 172.0, 174.2.

HRMS (ESI) calcd for $C_{102}H_{150}N_{16}O_{18}^{2+}$ $(M+2H)^{2+}$: 944.0669; found: 944.0667.

(3*S*,3'*S*,6*S*,6'*S*,9*S*,9'*S*,12*S*,12'*S*,15*S*,15'*S*,18*S*,18'*S*,21*S*,21'*S*)- 21,21'-((-butane-1,4-diylbis(1*H*-indole-1,3-diyl))bis(methylene))bis(18-((*R*)-3-hydroxy-2-methylpropyl)-6-isobutyl-3,9-diisopropyl-12-((*R*)-methoxy(phenyl)methyl)-1,7,15,19-tetramethyl-1',4',7',10',13',16',19'-heptaazacyclohenicosane-2,5,8,11,14,17,20-heptaone) (SI-61)

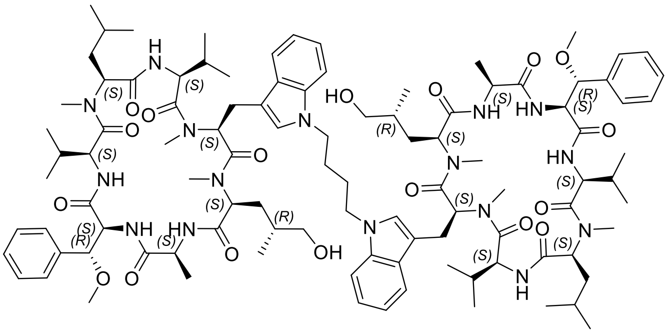

A Schlenk flask was charged with **SI-60** (25.4 mg, 26.5 µmol) and Grubbs catalyst 2nd Gen (2.7 mg, 3.2 µmol). The flask was evacuated and back-filled with argon three times. Freshly degassed (3x freeze-pump-thaw) DCM (0.21 ml) was added and the mixture was stirred at rt. After 68 h, LC/MS indicated ~50% conversion. The mixture was evaporated in vacuo and the residue was dissolved in MeOH (0.7 ml). Pd/C (10 wt%) (1.4 mg) was added and the mixture was stirred under H_2 atmosphere for 3.5 h, after which LC/MS indicated full conversion. The mixture was filtered over a syringe filter and evaporated in vacuo. RP flash chromatography (H_2O/MeCN 90:10 – 5:95) followed by prep HPLC (H_2O/MeCN 90:10 – 5:95) and lyophilization yielded **SI-61** (10.0 mg, 5.3 µmol, 40%) as a white, amorphous solid.

$[\alpha]^{D}_{20}$ = −90.7 (c 0.2, $CHCl_3$).

^{1}H NMR (500 MHz, $CDCl_3$) δ -0.48 – -0.37 (m, 2H), 0.25 (d, *J* = 6.7 Hz, 6H), 0.42 (d, *J* = 6.6 Hz, 6H), 0.62 (d, *J* = 6.6 Hz, 6H), 0.83 – 0.86 (m, 2H), 0.92 – 0.96 (m, 12H), 0.97 – 1.01 (m, 8H), 1.08 – 1.12 (m, 12H), 1.13 – 1.15 (m, 2H), 1.52 – 1.60 (m, 2H), 1.69 – 1.78 (m, 6H), 1.82 – 1.89 (m, 2H), 2.20 – 2.28 (m, 2H), 2.26 – 2.36 (m, 2H), 2.53 (s, 6H), 2.84 (s, 6H), 2.90 (dd, *J* = 11.2, 5.5 Hz, 2H), 3.00 (dd, *J* = 11.1, 4.4 Hz, 2H), 3.13 (dd, *J* = 14.3, 5.9 Hz, 2H), 3.34 – 3.42 (m, 14H), 3.98 (t, *J* = 6.4 Hz, 4H), 4.43 – 4.52 (m, 4H), 4.70 – 4.79 (m, 4H), 4.80 – 4.86 (m, 4H), 5.14 (d, *J* = 5.6 Hz, 2H), 5.36 (dd, *J* = 9.4, 6.1 Hz, 2H), 6.75 (s, 2H), 6.89 (d, *J* = 4.8 Hz, 2H), 7.04 – 7.11 (m, 2H), 7.17 – 7.19 (m, 4H), 7.21 – 7.26 (m, 10H), 7.44 (d, *J* = 7.9 Hz, 2H), 8.09 (d, *J* = 10.1 Hz, 2H), 8.16 (d, *J* = 7.6 Hz, 2H), 8.47 (d, *J* = 9.3 Hz, 2H).

^{13}C NMR (126 MHz, $CDCl_3$) δ 17.2, 18.8, 19.48, 19.51, 20.20, 20.25, 22.7, 23.7, 25.4, 26.7, 28.0, 28.9, 29.6, 31.0, 31.8, 32.3, 32.4, 32.9, 39.1, 45.9, 50.8, 53.4, 54.4, 55.5, 55.9, 57.9, 59.0, 59.1, 66.2, 80.1, 108.8, 109.7, 119.0, 119.8, 122.5, 126.2, 127.8, 128.3, 128.5, 129.0, 135.1, 136.2, 168.5, 169.0, 169.8, 170.7, 171.5, 172.0, 174.2.

HRMS (ESI) calcd for $C_{102}H_{151}N_{16}O_{18}^{+}$ $(M+H)^{+}$: 1888.1387; found:1888.1466.

Synthesis of Trp-Nα methylated exit vector 3 Homo-BacPROTACs (SI-65, SI-66)

Methyl *N*-(((2*S*,3*R*)-2-((2*S*)-2-((2*S*)-2-(2-(((allyloxy)carbonyl)(methyl)amino)-*N*-methyl-3-(1-methyl-1*H*-indol-3-yl)propanamido)-5-((tert-butyldimethylsilyl)oxy)-4-methylpentanamido)propanamido)-3-methoxy-3-(4-(prop-2-yn-1-yloxy)phenyl)propanoyl)-L-valyl)-*N*-methyl-L-leucinate (SI-62)

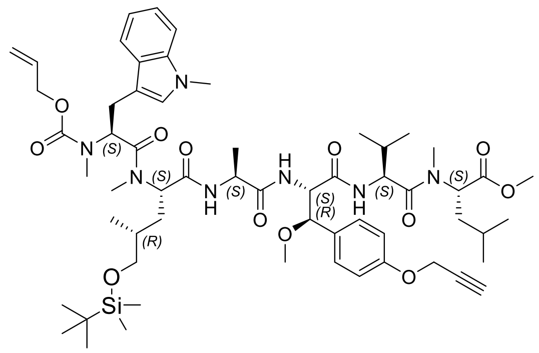

Pentapeptide **SI-35** (500 mg, 481 µmol) was dissolved in DCM (4.8 ml), tris(2-aminoethyl)amine (720 µl, 4.81 mmol) was added and the reaction mixture was stirred vigorously. After 30 min, full conversion was determined by TLC. The reaction mixture was washed with saturated NaCl solution (20 ml×3) and phosphate buffer (67 mM, pH 5.5) (30 ml×3). The aqueous phase was back–extracted with DCM (1x). The organic phase was dried (Na_2SO_4) and evaporated in vacuo. The crude peptide was dissolved in DMF (4.8 ml), cooled to 0°C and *N*α-((allyloxy)carbonyl)-*N*α,1-dimethyl-L-tryptophan (prepared as described above, cf. compound **18**) (152 mg, 480 µmol), HATU (366 mg, 962 µmol), HOAt (65.5 mg, 481 µmol) and DIPEA (420 µl, 2.41 mmol) were added. The reaction temperature was allowed to raise up to rt over the course of 18 h and the reaction was quenched by the addition of 1 M $KHSO_4$. The mixture was diluted with EtOAc, transferred to a separatory funnel, and shaken vigorously. The phases were separated, and the organic phase was subsequently washed with H_2O, sat. $NaHCO_3$ soln. and brine. The organic phase was dried (Na_2SO_4) and evaporated. Flash chromatography (CyH/EtOAc 100:0 – 45:55) followed by lyophilization afforded **SI-62** (319mg, 286 µmol, 60%) as a white amorphous solid. R_f = 0.39 (PE/EtOAc 4:6).

$[\boldsymbol{\alpha}]_{20}^{D}$ = –72.0 (c 0.5, $CHCl_3$).

^{1}H NMR (500 MHz, $CDCl_3$) *(mixture of rotamers)* δ -0.09 – -0.03 (m, 1.7H), 0.00 – 0.06 (m, 5.3H), 0.08 (s, 0.7H), 0.41 (d, *J* = 6.6 Hz, 0.8H), 0.81 (s, 2.5H), 0.86 – 0.89 (m, 7.6H), 0.90 – 1.09 (m, 22.7 H), 1.24 (d, *J* = 6.8 Hz, 1.2H), 1.43 – 1.56 (m, 3.2H), 1.64–1.84 (m, 2.6H), 1.83–1.97 (m, 1.1H), 2.00 – 2.18 (m, 1.6H), 2.41 – 2.64 (m, 1.2H), 2.78 (s, 2.8H), 2.85–2.94 (m, 2.5H), 2.94 – 3.02 (m, 3.5H), 3.14 (s, 0.7H), 3.26 (s, 0.9H), 3.38 – 3.32 (m, 3.0H), 3.36 – 3.53 (m, 2.3H), 3.62 – 3.75 (m, 8.4H), 4.08 – 4.19 (m, 0.4H), 4.19 – 4.33 (m, 0.8H), 4.40–4.50 (m, 0.4H), 4.50–4.58 (m, 0.3H), 4.58 – 4.71 (m, 4.5H), 4.71 – 4.85 (m, 2.5H), 5.05 – 5.10 (m, 0.4H), 5.10 – 5.15 (m, 0.7H), 5.16 – 5.30 (m, 2.1H), 5.32–5.39 (m, 1.5H), 5.52 (m, 0.5H), 5.56 – 5.69 (m, 0.3H), 5.84 – 5.98 (m, 0.9H), 6.22 (d, *J* = 6.9 Hz, 0.5H), 6.40 (d, *J* = 6.7 Hz, 0.4H), 6.57 – 6.68 (m, 1.1H), 6.81–6.88 (m, 1.0H), 6.87 – 6.96 (m, 2.6H), 7.05 – 7.15 (m, 4.0H), 7.17 – 7.26 (m, 2.5H), 7.33 – 7.43 (m, 1.2H), 7.59 (q, *J* = 7.5 Hz, 1.0H), 7.71 (d, *J* = 7.8 Hz, 0.5H).

^{13}C NMR (126 MHz, $CDCl_3$) *(mixture of rotamers)* δ -5.32, -5.30, -5.2, -3.4, 15.7, 17.3, 17.4, 17.6, 17.7, 17.8, 17.9, 18.39, 18.41, 19.6, 19.7, 21.5, 23.4, 25.0, 26.0, 29.1, 29.6, 30.0, 30.4, 30.7, 30.8, 31.0, 31.44, 31.38, 31.8, 32.2, 32.3, 32.75, 32.68, 37.0, 49.1, 49.2, 49.7, 52.2, 54.0, 54.18, 54.20, 54.61, 54.64, 54.7, 54.8, 55.5, 55.80, 55.83, 56.6, 57.4, 57.5, 57.7, 57.8, 66.4, 66.8, 67.0, 67.1, 68.0, 75.7, 75.8, 78.62, 78.64, 80.9, 109.2, 109.3, 109.4, 109.5, 114.8, 114.9, 117.3, 117.7, 118.57, 118.63, 118.8, 119.0, 119.2, 119.4, 121.7, 122.0, 127.3, 127.8, 127.88, 127.95, 128.1, 128.2, 129.62, 129.65, 129.8, 132.4, 132.6, 132.9, 136.9, 137.0, 155.6, 156.3, 157.4, 157.58, 157.65, 168.42, 168.44, 168.5, 170.5, 171.4, 171.5, 171.7, 171.97, 172.03, 172.08, 172.14.

Selected diagnostic peaks:

Major rotamer: **^{1}H NMR** (500 MHz, $CDCl_3$) δ 0.87 (s, 9H), 1.43 – 1.56 (m, 3H), 2.50 – 2.52 (m, 1H), 2.78 (s, 3H), 2.91 (s, 3H), 3.30 (s, 3H), 3.69 (s, 3H), 5.84 – 5.98 (m, 1H), 6.57 – 6.68 (m, 1H), 7.59 (q, *J* = 7.5 Hz, 1H). **^{13}C-NMR** (126 MHz, $CDCl_3$) δ 19.65, 26.0, 37.0, 55.83, 81.0, 170.5.

Minor rotamer: **^{1}H NMR** (500 MHz, $CDCl_3$) δ -0.09 – -0.03 (m, 6H), 0.81 (s, 9H), 2.48 – 2.49 (m, 1H), 2.76 (s, 3H), 2.89 (s, 3H), 3.26 (s, 3H), 3.68 (s, 3H), 5.56 – 5.69 (m, 1H), 6.40 (d, *J* = 6.7 Hz, 1H) **^{13}C-NMR** (126 MHz, $CDCl_3$) δ 19.69, 55.80.

HRMS (ESI): calcd for $C_{59}H_{90}N_7O_{12}Si^+$ (M+H)$^+$: 1049.1101; found: 1049.1048.

Methyl *N*-(((2*S*,3*R*)-2-((2*S*)-2-((2*S*)-2-(2-(2-(((allyloxy)carbonyl)amino)-*N*-methyl-3-(1-methyl -1*H*-indol-3-yl)propanamido)-5-((tert-butyldimethylsilyl)oxy)-4-methylpentanamido) propanamido)-3-methoxy-3-(4-(prop-2-yn-1-yloxy)phenyl)propanoyl)-L-valyl)-*N*-methyl-L-leucinate (SI-63)

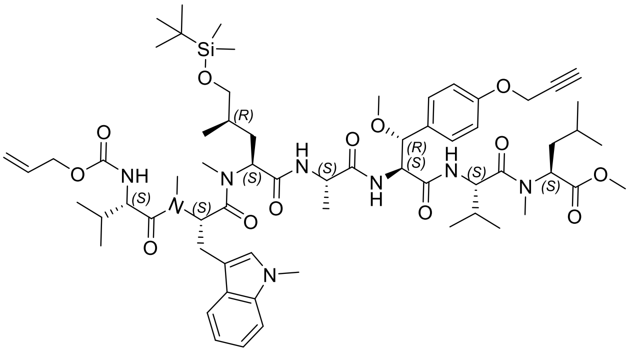

Deprotection according to *GP2*: **SI-62** (322 mg, 288 mmol), DMBA (135 mg, 865 µmol), $Pd(PPh_3)_4$ (10.0 mg, 8.65 µmol). The crude deprotected peptide was dissolved in DMF (3 ml), cooled to 0 °C and Alloc-Val-OH (122 mg, 605 µmol), HATU (219 mg, 576 µmol), HOAt (29.2 mg, 288 µmol) and DIPEA (250 µl, 1.40 mmol) were added. The reaction temperature was allowed to raise up to rt over the course of 18 h and the reaction was quenched by the addition of 1 M $KHSO_4$. The mixture was diluted with EtOAc, transferred to a separatory funnel, and shaken vigorously. The phases were separated, and the organic phase was subsequently washed with H_2O, sat. $NaHCO_3$ soln. and brine. The organic phase was dried (Na_2SO_4) and evaporated. Flash chromatography (CyH/EtOAc 100:0 – 45:55) followed by lyophilization afforded **SI-63** (268 mg, 220 µmol, 77%) as a white amorphous solid. R_f = 0.34 (PE/EtOAc 4:6).

$[\alpha]_{20}^{D}$ = –64.8 (c 1.0, $CHCl_3$).

^{1}H NMR (500 MHz, $CDCl_3$) *(mixture of rotamers)* δ -0.09 – -0.03 (m, 1.1H), 0.03 (d, *J* = 6.0 Hz, 3.3H), 0.09 (s, 0.7H), 0.17 (d, *J* = 7.6 Hz, 0.3H), 0.34 – 0.37 (m, 0.2H), 0.42 (d, *J* = 6.6 Hz, 0.5H), 0.81 (s, 2.1H), 0.88 (s, 5.0 H), 0.90 – 1.00 (m, 17.2H), 1.19 – 1.29 (m, 1.9H), 1.33 – 1.55 (m, 3.4H), 1.92 – 2.16 (m, 1.6H), 2.47 – 2.51 (m, 0.2H), 2.53 (t, *J* = 2.4 Hz, 0.4H), 2.62 – 2.79 (m, 2.4H), 2.85 (s, 3.0H), 2.97 – 3.01 (m, 1.5H), 3.05 – 3.18 (m, 1.5H), 3.20 – 3.29 (m, 0.8H), 3.30 (s, 1.3H), 3.32 – 3.42 (m, 0.6H), 3.44 – 3.57 (m, 1.0H), 3.59 – 3.67 (m, 1.8H), 3.69 (s, 4.2H), 4.09 – 4.20 (m, 0.7H), 4.47 – 4.87 (m, 6.0H), 5.01 – 5.14 (m, 0.5H), 5.19 – 5.25 (m, 0.9H), 5.27 – 5.38 (m, 2.0H), 5.46 – 5.50 (m, 0.6H), 5.84 – 5.98 (m, 1.5H), 6.13 – 6.26 (m, 0.5H), 6.59 – 6.72 (m, 0.6H), 6.80 – 6.88 (m, 0.7H), 6.88 – 6.94 (m, 0.6H), 7.08 – 7.15 (m, 2.6H), 7.16 – 7.25 (m, 1.3H), 7.39 (d, *J* = 8.8 Hz, 0.42H), 7.44 – 7.50 (m, 0.4H), 7.52 – 7.59(m, 0.5H), 7.61 – 7.74 (m, 1.1H).

^{13}C NMR (126 MHz, $CDCl_3$) *(mixture of rotamers)* δ -5.3, -4.6, -4.4, -3.4, 15.9, 16.3, 17.0, 17.33, 17.35, 17.4, 17.7, 17.8, 18.0, 18.1, 18.4, 18.7, 19.5, 19.60, 19.64, 19.8, 19.9, 21.5, 23.3, 23.4, 25.0, 25.1, 25.3, 25.8, 26.0, 26.1, 28.7, 29.0, 29.1, 29.8, 30.7, 30.9, 31.2, 31.47, 31.55, 31.7, 31.8, 32.0, 32.2, 32.67, 32.72, 32.79, 32.83, 33.0, 33.1, 33.2, 37.0, 38.8, 49.1, 49.3, 50.0, 52.3, 52.30, 53.32, 54.2, 54.29, 54.35, 54.8, 55.3, 55.8, 55.9, 56.0, 56.5, 57.4, 57.47, 57.53, 57.6, 57.8, 58.2, 58.9, 65.9, 67.0, 67.2, 68.2, 75.0, 75.78, 75.80, 75.84, 78.6, 81.0, 81.1, 109.2, 109.4, 109.5, 114.8, 114.9, 117.8, 118.79, 118.85, 119.29, 119.34, 119.55, 121.62, 121.78, 121.89, 122.19, 127.05, 127.48, 127.76, 127.84, 128.24, 128.39, 128.6, 128.7, 129.6, 129.6, 132.2, 132.3, 132.79, 132.84, 132.9, 133.1, 137.0, 156.5, 156.6, 157.7, 168.4, 168.5, 168.6, 170.3, 171.7, 172.08, 172.12, 172.2, 172.6, 173.8.

Selected diagnostic peaks:

Major rotamer: **^{1}H NMR** (500 MHz, $CDCl_3$) δ 0.02 (s, 3H), 0.04 (s, 3H), 0.88 (s, 9H), 1.19 – 1.29 (m, 3H), 2.53 (t, *J* = 2.4 Hz, 1H), 2.73 (s, 3H), 2.99, (s, 3H), 3.29 (s, 3H), 3.69 (s, 3H), 4.09 – 4.20 (m, 1H), 5.27 – 5.38 (m, 1H), 5.84 – 5.97 (m, 1H), 6.88 – 6.94 (m, 1H), 7.70 – 7.74 (m, 2H). **^{13}C-NMR** (126 MHz, $CDCl_3$) δ 26.08, 37.0, 55.85, 81.0.

Minor rotamer: **^{1}H NMR** (500 MHz, $CDCl_3$) δ -0.07 (s, 3H), -0.05 (s, 3H), 0.81 (s, 9H), 2.48 – 2.50 (m, 1H), 2.70 (s, 3H), 2.98 (s, 3H), 3.27 (s, 3H), 3. 63 (s, 3H), 6.82 – 6.87 (m, 1H). 8.24 – 8.31 (m, 1H). **^{13}C-NMR** (126 MHz, $CDCl_3$) δ 26.04, 55.89, 81.1.

HRMS (ESI): calcd for $C_{64}H_{99}N_8O_{13}Si^+$ $(M+H)^+$: 1215.7095; found: 1215.7045.

(9*S*,12*R*,15*S*,18*R*,21*S*)-21-(3-Hydroxy-2-methylpropyl)-9-isobutyl-6,12-diisopropyl-15-((*R*)-methoxy(4-(prop-2-yn-1-yloxy)phenyl)methyl)-1,4,10,18-tetramethyl-3-((1-methyl-1*H*-indol-3-yl)methyl)-1,4,7,10,13,16,19-heptaazacyclohenicosane-2,5,8,11,14,17,20-heptaone (SI-64)

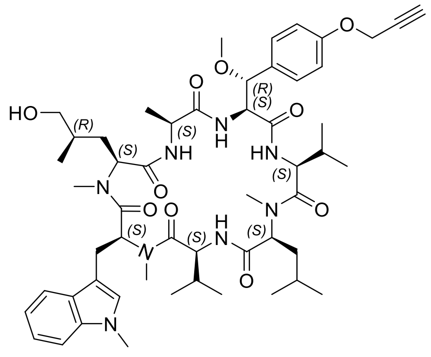

Prepared according to *GP6a*: **SI-63** (143 mg, 118 µmol), 1 M LiOH (141 µl, 141 µmol) (3.5 h); $Pd(OAc)_2$ (800 µg, 3.54 µmol), TPPTS (4.02 mg, 7.08 µmol), Et_2NH (62.0 µl, 590 µmol) (1 h); HATU (179 mg, 220 µmol), HOAt (32.0 mg, 236 µmol) DIPEA (202 µl, 1.18 mmol) (addition over 1.5 h, 17 h); NH_4F (87.0 mg, 2.40 mmol) (16 h). RP flash chromatography (H_2O/MeCN 90:10 - 5:95) followed by lyophilization afforded **SI-64** (33.0 mg, 33.0 µmol, 28%) as an off-white, amorphous solid.

$[\alpha]_{20}^{D}$ = –63.4 (c 0.3, $CHCl_3$).

^{1}H NMR (500 MHz, $CDCl_3$) δ -0.24 – -0.15 (m, 1H), 0.39 (d, *J* = 6.7 Hz, 3H), 0.48 (d, *J* = 6.6 Hz, 3H), 0.66 (d, *J* = 6.8 Hz, 3H), 0.86 – 1.02 (m, 10H), 1.07–1.17 (m, 6H), 1.48 – 1.63 (m, 1H), 1.81 – 2.00 (m, 6H), 2.18 – 2.39 (m, 2H), 2.47 (t, *J* = 2.4 Hz, 1H), 2.61 (s, 3H), 2.85 (s, 3H), 2.96–3.03 (m, 1H), 3.05–3.13 (m, 1H), 3.14 – 3.23 (m, 1H), 3.32–3.39 (m, 6H), 3.71 (s, 3H), 4.46 – 4.54 (m, 2H), 4.62 (d, *J* = 2.4 Hz, 2H), 4.71 – 4.82 (m, 2H), 4.83 – 4.93 (m, 2H), 5.10 (d, *J* = 5.4 Hz, 1H), 5.44 (dd, *J* = 8.9, 6.6 Hz, 1H), 6.80 (s, 1H), 6.83 – 6.92 (m, 2H), 6.96–7.04 (m, 1H), 7.06 – 7.15 (m, 1H), 7.15 – 7.19 (m, 2H), 7.19 – 7.24 (m, 1H), 7.26–7.31, 7.45 (d, *J* = 7.9 Hz, 1H), 8.16–8.27 (m, 2H), 8.50 (d, *J* = 9.4 Hz, 1H).

^{13}C NMR (126 MHz, $CDCl_3$) δ 17.4, 18.9, 19.0, 19.5, 20.2, 20.3, 22.6, 23.6, 25.4, 26.6, 29.0, 29.7, 31.0, 31.9, 32.3, 32.5, 32.9, 33.1, 39.1, 51.0, 53.6, 54.5, 55.5, 55.8, 56.0, 57.8, 59.0, 59.3, 66.2, 75.8, 78.5, 79.6, 108.5, 109.7, 114.7, 118.7, 119.6, 122.4, 127.1, 127.7, 127.9, 129.6, 137.0, 158.1, 168.4, 169.2, 169.9, 170.7, 171.6, 172.1, 174.3.

HRMS (ESI): calcd for $C_{53}H_{77}N_8O_{10}^+$ $(M+H)^+$: 985.5757; found: 985.5713.

(3*S*,6*S*,9*S*,12*S*,15*S*,18*S*,21*S*)-21-((*R*)-3-Hydroxy-2-methylpropyl)-15-((*R*)-(4-((1-(14-(4-((4-((*S*)-((2*R*,5*R*,8*R*,11*R*,14*R*,17*R*,20*R*)-17-((*S*)-3-hydroxy-2-methylpropyl)-8-isobutyl-5,11-diisopropyl-7,13,16,20-tetramethyl-14-((1-methyl-1*H*-indol-3-yl)methyl)-3,6,9,12,15,18,21-heptaoxo-1,4,7,10,13,16,19-heptaazacyclohenicosan-2-yl)(methoxy)methyl)phenoxy)methyl)-1*H*-1,2,3-triazol-1-yl)-3,6,9,12-tetraoxatetradecyl)-1*H*-1,2,3-triazol-4-yl)methoxy)phenyl)(methoxy)methyl)-9-isobutyl-6,12-diisopropyl-1,4,10,18-tetramethyl-3-((1-methyl-1*H*-indol-3-yl)methyl)-1,4,7,10,13,16,19-heptaazacyclohenicosane-2,5,8,11,14,17,20-heptaone (SI-65)

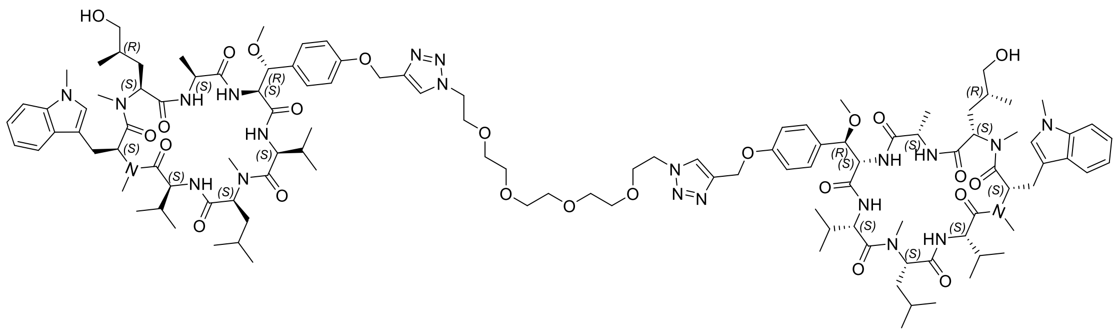

Prepared according to *GP7*: **SI-64** (22.0 mg, 22.0 µmol), 1,14-diazido-3,6,9,12-tetraoxatetradecane (3.22 mg, 11.0 µmol), 1 M $CuSO_4$ (11.2 µl, 11.2 µmol), 1 M sodium ascorbate (13.4 µl, 13.4 µmol) (17 h). RP flash chromatography (H_2O/MeCN 70:30 – 5:95) followed by lyophilization afforded **SI-65** (16.3 mg, 7.22 µmol, 64%) as a white amorphous solid.

$[\alpha]_{20}^{D}$ = –55.6 (c 0.4, $CHCl_3$).

^{1}H NMR (500 MHz, $CDCl_3$**)** δ -0.21– -0.13 (m, 2H), 0.40 (d, *J* = 6.7 Hz, 6H), 0.48 (d, *J* = 6.5 Hz, 6H), 0.65 (d, *J* = 6.8 Hz, 6H), 0.91 – 1.00 (m, 18H), 1.08–1.11 (m, 8H), 1.13–1.17 (m, 7H), 1.46 – 1.60 (m, 2H), 1.93 – 2.07 (m, 8H), 2.17 – 2.35 (m, 4H), 2.62 (s, 6H), 2.85 (s, 6H), 2.97–3.04 (m, 2H), 307–3.13 (m, 2H), 3.14 – 3.22 (m, 2H), 3.33–3.37 m, 14H), 3.38–3.43 (m, 2H), 3.56 – 3.60 (m, 12H), 3.71 (s, 6H), 3.87 (t, *J* = 5.1 Hz, 4H), 4.46 – 4.54 (m, 8H), 4.69 – 4.80 (m, 4H), 4.82 – 4.90 (m, 4H), 5.08 (d, *J* = 5.4 Hz, 2H), 5.10 (m, 4H), 5.44 (dd, *J* = 8.8, 6.8 Hz, 2H), 6.80 (s, 2H), 6.89 (d, *J* = 2.2 Hz, 2H), 7.00 (d, *J* = 4.8 Hz, 2H), 7.09 (t, *J* = 7.7 Hz, 2H), 7.16 (d, *J* = 8.7 Hz, 4H), 7.19 – 7.24 (m, 2H), 7.45 (d, *J* = 7.9 Hz, 2H), 7.77 (s, 2H), 8.17 (d, *J* = 7.7 Hz, 2H), 8.26 (d, *J* = 10.1 Hz, 2H), 8.48 (d, *J* = 9.3 Hz, 2H).

^{13}C NMR (126 MHz, $CDCl_3$) δ 17.4, 18.9, 19.0, 19.5, 20.2, 20.4, 22.6, 23.6, 25.42, 26.6, 29.0, 29.7, 31.0, 31.9, 32.3, 32.4, 32.9, 33.1, 39.1, 50.5, 51.0, 53.6, 54.5, 55.5, 56.0, 57.8, 59.0, 59.2, 62.0, 66.1, 69.5, 70.6, 70.6, 70.7, 79.7, 108.5, 109.7, 114.5, 118.7, 119.6, 122.4, 124.1, 127.1, 127.66, 127.68, 129.6, 137.0, 143.6, 158.8, 168.4, 169.2, 171.0, 170.7, 171.6, 172.0, 174.2.

HRMS (ESI): calcd for $C_{116}H_{173}N_{22}O_{24}^+$ $(M+H)^+$: 1130.1547; found: 1130.1516.

(3*S*,3'*S*,6*S*,6'*S*,9*S*,9'*S*,12*S*,12'*S*,15*S*,15'*S*,18*S*,18'*S*,21*S*,21'*S*)-15,15'-((1*R*,1'*R*)-((((pentane-1,5-diylbis(1*H*-1,2,3-triazole-1,4-diyl))bis(methylene))bis(oxy))bis(4,1-phenylene))bis(methoxymethylene))bis(21-((*R*)-3-hydroxy-2-methylpropyl)-9-isobutyl-6,12-diisopropyl-1,4,10,18-tetramethyl-3-((1-methyl-1*H*-indol-3-yl)methyl)-1,4,7,10,13,16,19-heptaazacyclohenicosane-2,5,8,11,14,17,20-heptaone) (SI-66)

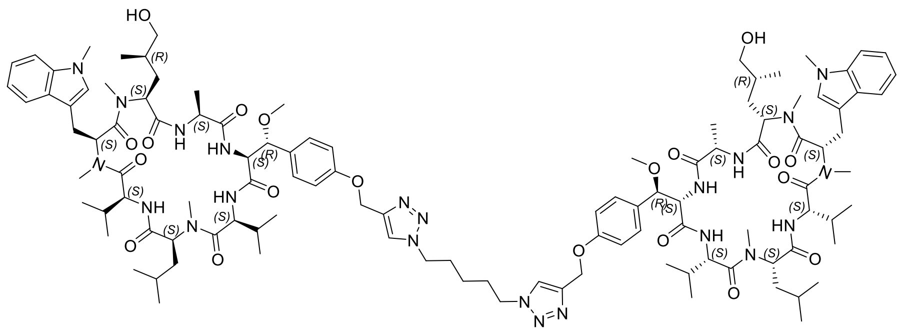

Prepared according to *GP7*: **SI-64** (20.0 mg, 20.0 µmol), 1,5-diazidopentane (1.56 mg, 10.1 µmol), 1 M $CuSO_4$ (10.2 µl, 10.2 µmol), 1 M sodium ascorbate (12.2 µl, 12.2 µmol) (18 h). RP flash chromatography (H_2O/MeCN 70:30 – 5:95) followed by lyophilization afforded **SI-66** (16.7 mg, 3.53 µmol, 79%) as a white amorphous solid.

$[\alpha]_{20}^{D}$ = –49.0 (c 0.5, $CHCl_3$).

^{1}H NMR (500 MHz, $CDCl_3$) δ -0.21– -0.12 (m, 2H), 0.40 (d, *J* = 6.7 Hz, 6H), 0.48 (d, *J* = 6.5 Hz, 6H), 0.65 (d, *J* = 6.7 Hz, 6H), 0.91 – 1.03 (m, 20H), 1.08 – 1.17 (m, 14H), 1.22 – 1.29 (m, 2H), 1.35 – 1.44 (m, 2H), 1.50 – 1.64 (m, 2H), 1.91 – 2.03 (m, 8H), 2.19 – 2.41 (m, 4H), 2.62 (s, 6H), 2.85 (s, 6H), 2.95 – 3.04 (m, 2H), 3.09 (dd, *J* = 11.2, 4.3 Hz, 2H), 3.12 – 3.24 (m, 2H), 3.33 – 3.44 (m, 12H), 3.71 (s, 6H), 4.35 (q, *J* = 6.6, 6.2 Hz, 6H), 4.41 – 4.60 (m, 4H), 4.68 – 4.81 (m, 4H), 4.83 – 4.92 (m, 4H), 5.06 – 5.14 (m, 6H), 5.44 (dd, *J* = 8.7, 6.7 Hz, 2H), 6.80 (s, 2H), 6.88 (d, *J* = 8.2

Hz, 4H), 7.01 (d, *J* = 4.9 Hz, 2H), 7.06 – 7.13 (m, 2H), 7.17 (d, *J* = 8.4 Hz, 4H), 7.20 – 7.24 (m, 2H), 7.23 – 7.29 (m, 2H), 7.45 (d, *J* = 7.9 Hz, 2H), 7.57 (s, 2H), 8.17 (d, *J* = 7.6 Hz, 2H), 8.26 (d, *J* = 10.0 Hz, 2H), 8.48 (d, *J* = 9.3 Hz, 2H).

^{13}C NMR (126 MHz, $CDCl_3$) δ 17.4, 18.9, 19.0, 19.5, 20.2, 20.4, 22.6, 23.59, 23.63, 25.4, 26.6, 29.0, 29.7, 29.7, 31.0, 32.0, 32.3, 32.4, 32.9, 33.1, 39.1, 50.1, 51.0, 53.6, 54.5, 55.5, 56.1, 57.8, 59.0, 59.3, 62.0, 66.2, 79.7, 108.5, 109.7, 114.6, 118.7, 119.6, 122.4, 122.7, 127.1, 127.68, 127.76, 129.7, 137.0, 144.0, 158.7, 168.4, 169.2, 170.0, 170.7, 171.6, 172.0, 174.2.

HRMS (ESI): calcd for $C_{111}H_{163}N_{22}O_{20}^+$ $(M+H)^+$: 1063.1258; found: 1063.1224.

Synthesis of a Phenylalanine-containing dCym derivative (SI-72)

Methyl (2*S*)-2-[(2*S*)-2-[(2*S*)-2-{[(*tert*-butoxy)carbonyl]amino}-3-phenylpropanamido]-*N*,3-dimethylbutanamido]-4-methylpentanoate (SI-67)

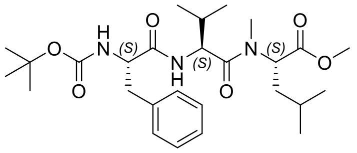

To a solution of methyl *N*-(((benzyloxy)carbonyl)-L-valyl)-N-methyl-L-leucinate (500 mg, 1.27 mmol) in DCM (3.0 ml, 0.42 M) was added HBr (33% in AcOH) (3.0 ml) slowly at 0°C and the mixture was allowed to reach rt. The solvents were evaporated in vacuo and the residue was triturated with diethyl ether. The crude amine was used in the peptide coupling without further purification.

To a solution of Boc-Phe-OH (200 mg, 754 μmol) in DMF (1.0 ml, 0.75 M) was added DIPEA (526 μl, 3.02 mmol), HOAt (51 mg, 404 μmol) and HATU (315 mg, 828 μmol). Then, the deprotected dipeptide (292 mg, 1.13 mmol) was added and the mixture was stirred at rt. The reaction was quenched with water and extracted with EtOAc. The organic layer was washed with brine, dried over Na_2SO_4 and concentrated in vacuo. Silica gel column chromatography (50% EtOAc in petroleum ether) yielded **SI-67** (300 mg, 593 μmol, 79%).

Major rotamer: **^{1}H NMR** (500 MHz, $CDCl_3$) δ 0.85 (d, *J* = 6.8 Hz, 3H), 0.89 (d, *J* = 6.5 Hz, 3H), 0.94 (d, *J* = 6.7 Hz, 3H), 0.97 (d, *J* = 6.8 Hz, 3H), 1.39 (s, 10H), 1.63 – 1.71 (m, 1H), 1.71 – 1.78 (m, 1H), 2.01 – 2.09 (m, 1H), 2.96 – 3.03 (m, 4H), 3.10 (dd, *J* = 14.0, 6.1 Hz, 1H), 3.68 (s, 3H), 4.37 (d, *J* = 7.4 Hz, 1H), 4.79 (dd, *J* = 8.8, 6.1 Hz, 1H), 4.95 (d, *J* = 8.1 Hz, 1H), 5.30 (dd, *J* = 10.6, 5.1 Hz, 1H), 6.67 (d, *J* = 8.8 Hz, 1H), 7.14 – 7.19 (m, 2H), 7.20 – 7.23 (m, 1H), 7.24 – 7.31 (m, 2H). **^{13}C NMR** (126 MHz, CDCl3) δ 17.4, 19.6, 21.5, 23.4, 24.9, 28.4, 31.4, 31.5, 37.0, 38.1, 52.3, 54.0, 54.6, 55.8, 80.2, 127.0, 128.7, 129.5, 136.6, 155.3, 171.1, 172.2, 172.3.

Minor rotamer (ratio *~10:1, selected signals)*: **^{1}H NMR** (500 MHz, $CDCl_3$) δ 0.82 (d, *J* = 6.7 Hz, 3H), 1.00 (d, *J* = 6.1 Hz, 3H), 1.93 – 2.00 (m, 1H), 2.82 (s, 3H), 3.70 (s, 3H), 4.66 (t, *J* = 7.3 Hz, 1H), 4.90 (d, *J* = 8.6 Hz, 1H), 6.55 (d, *J* = 9.3 Hz, 1H).

HRMS (ESI) calcd for $C_{27}H_{44}N_3O_6^+$ $(M+H)^+$: 506.3225; found: 506.3227.

Methyl (2*S*)-2-[(2*S*)-2-[(2*S*)-2-[(2*S*)-2-{[(*tert*-butoxy)carbonyl]amino}propanamido]-3-phenylpropanamido]-*N*,3-dimethylbutanamido]-4-methylpentanoate (SI-68)

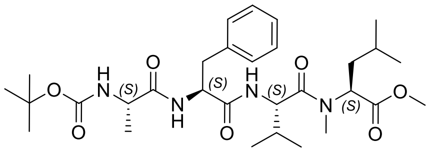

To a solution of **SI-67** (500 mg, 989 μmol) in DCM (4.0 ml, 0.25 M) was added HCl (4 M in dioxane) (2.0 ml) slowly at 0 °C and the mixture was allowed to reach rt and stirred for 3 h. The solvents were evaporated in vacuo and the crude amine was used in the peptide coupling without further purification.

To a solution of the deprotected tripeptide (1.20 g, 2.96 mmol) in DMF (120 ml, 0.025 M) was added DIPEA (1.55 ml, 8.87 mmol) and HATU (1.69 g, 4.44 mmol) at 0 °C. After 20 minutes, Boc-Ala-OH (616 mg, 3.26 mmol) was added, the mixture was allowed to reach rt and stirred for 16 h. The reaction was quenched with water and extracted with EtOAc. The organic layer was washed with water, dried over Na_2SO_4 and concentrated in vacuo. Silica gel column chromatography (30% EtOAc in hexane) yielded **SI-68** (1.10 g, 1.91 mmol, 64%) as a white solid.

$[\alpha]_{20}^{D}$ = −50.4 (c 1.0, $CHCl_3$).

Major rotamer: **^{1}H NMR** (500 MHz, $CDCl_3$) δ 0.84 (d, *J* = 6.8 Hz, 3H), 0.90 (d, *J* = 6.5 Hz, 3H), 0.95 (d, *J* = 6.8 Hz, 3H), 0.95 (d, *J* = 6.9 Hz, 3H), 1.28 (d, *J* = 7.0 Hz, 3H), 1.41 (s, 9H), 1.46 (dd, *J* = 9.6, 4.4 Hz, 1H), 1.68 (ddd, *J* = 14.6, 10.7, 4.6 Hz, 1H), 1.75 (ddd, *J* = 14.6, 9.6, 5.1 Hz, 1H), 2.01 – 2.09 (m, 1H), 2.98 (s, 3H), 3.00 – 3.02 (m, 1H), 3.09 (dd, *J* = 14.0, 6.4 Hz, 1H), 3.68 (s, 3H), 4.08 – 4.18 (m, 1H), 4.65 – 4.72 (m, 1H), 4.76 (dd, *J* = 8.8, 6.2 Hz, 1H), 4.91 – 4.99 (m, 1H), 5.31 (dd, *J* = 10.7, 5.1 Hz, 1H), 6.59 – 6.69 (m, 2H), 7.13 – 7.17 (m, 2H), 7.16 – 7.23 (m, 1H), 7.23 – 7.26 (m, 2H). **^{13}C NMR** (126 MHz, $CDCl_3$) δ 17.5, 18.5, 19.6, 21.6, 23.4, 25.0, 28.4, 31.4, 31.4, 37.0, 38.1, 50.3, 52.3, 54.2, 54.3, 54.6, 80.2, 127.1, 128.7, 129.4, 136.3, 155.5, 170.4, 172.1, 172.2, 172.5.

Minor rotamer (ratio ~10:1, selected signals) **^{1}H NMR** (500 MHz, $CDCl_3$) δ 0.80 (dd, *J* = 6.8, 1.5 Hz, 3H), 0.98 – 1.01 (m, 6H), 1.95 – 2.00 (m, 1H), 2.83 (s, 3H), 3.70 (s, 3H). **^{13}C NMR** (126 MHz, $CDCl_3$) δ 20.0, 22.7, 22.8, 29.2, 38.7, 52.7, 53.9, 57.8.

HRMS (ESI) calcd for $C_{30}H_{49}N_4O_7^+$ $(M+H)^+$: 577.3596; found: 577.3603.

Methyl *N*-((2*S*,4*R*)-2-(((benzyloxy)carbonyl)(methyl)amino)-5-((*tert*-butyldimethylsilyl)oxy)-4-methylpentanoyl)-L-alanyl-L-phenylalanyl-L-valyl-*N*-methyl-L-leucinate (SI-69)

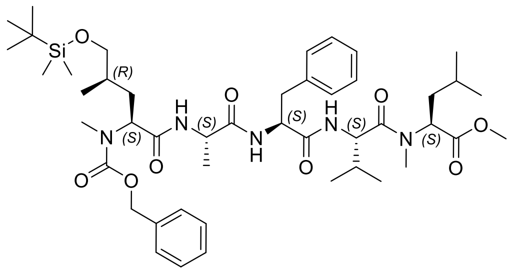

To **SI-68** (500 mg, 867 µmol) was added 4 M HCl in dioxane (2.17 ml, 8.67 mmol) at rt. After 1.5 h, the mixture was evaporated in vacuo and azeotroped with DCM three times. The resulting crude peptide was reacted according to *GP4*: (2S,4R)-2-(((benzyloxy)-carbonyl)(methyl)amino)-5-((tertbutyldimethylsilyl)oxy)-4-methylpentanoic acid (391 mg, 954 µmol), HOBt (146 mg, 954 µmol), EDC (183 mg, 954 µmol), NMM (200 µl, 1.82 mmol) (15 h). Flash chromatography (CyH/EtOAc 100:0 – 4:6) followed by lyophilization yielded **SI-69** (612 mg, 705 µmol, 81%) as a colorless amorphous solid. R_f = 0.36 (PE/EtOAc 4:6).

$[\alpha]_{20}^{D}$ = −57.6 (c 1.0, $CHCl_3$).

^{1}H NMR (500 MHz, $CDCl_3$) *(mixture of rotamers, ratio ca. 10:3:1)* δ -0.07 – 0.08 (m, 6.0H), 0.84 (d, *J* = 6.8 Hz,3.0H), 0.86 – 0.89 (s, 9.0H), 0.89 – 0.92 (m, 4.2H), 0.92 – 0.98 (m, 7.7H), 0.98 – 1.04 (m, 1.1H), 1.18 – 1.26 (m, 2.8H), 1.41 – 1.50 (m, 1.3H), 1.50 – 1.59 (m, 1.8H), 1.64 – 1.79 (m, 2.1H), 1.87 – 1.97 (m, 1.2H), 2.01 – 2.10 (m, 1.2H), 2.10 – 2.16 (m, 0.4H), 2.18 – 2.28 (m, 0.3H), 2.85 (s, 3.0H), 2.95 – 3.02 (m, 4.4H), 3.05 – 3.17 (m, 1.0H), 3.33 – 3.55 (m, 2.1H), 3.68 (s, 2.7H), 3.70 (s, 0.3H), 3.98 (t, *J* = 10.7 Hz, 0.2H),4.13 – 4.19 (m, 0.1H), 4.27 – 4.38 (m, 1.2H), 4.45 – 4.59 (m, 1.1H), 4.64 (q, *J* = 7.0 Hz, 1.1H), 4.70 – 4.80 (m, 1.1H), 5.05 – 5.23 (m, 2.6H), 5.30 (dd, *J* = 10.7, 5.1 Hz, 1.0H), 6.10 – 6.24 (m, 0.3H), 6.36 – 6.49 (m, 0.6H), 6.53 – 6.82 (m, 2.0H), 7.08 – 7.25 (m, 4.8H), 7.28 – 7.39 (m, 6.1H).

^{13}C NMR (126 MHz, $CDCl_3$) *(mixture of rotamers)* δ -5.3, -3.4, 16.8, 17.5, 17.6, 17.8, 18.5, 19.6, 21.5, 23.4, 24.9, 25.8, 26.1, 28.8, 30.7, 31.3, 31.4, 32.4, 33.3, 37.0, 37.8, 49.1, 52.3, 54.2, 54.4, 54.6, 57.4, 67.3, 67.76, 67.82, 75.0, 127.1, 128.0, 128.2, 128.6, 128.67, 128.72, 129.4, 136.5, 156.5, 157.3, 169.3, 170.4, 171.0, 171.6, 172.15, 172.23.

Selected diagnostic peaks:

Major rotamer: **¹H NMR** (500 MHz, $CDCl_3$) δ -0.05 – 0.06 (m, 6H), 0.84 (d, *J* = 6.8 Hz, 3H), 0.88 (s, 9H), 0.90 (d, *J* = 6.6 Hz, 3H), 2.85 (s, 3H), 2.98 (s, 3H), 3.68 (s, 3H), 3.98 (t, *J* = 10.7 Hz, 0H), 4.64 (q, *J* = 7.0 Hz, 1H), 4.73 – 4.78 (m, 1H), 5.30 (dd, *J* = 10.7, 5.1 Hz, 1H). **¹³C NMR** (126 MHz, $CDCl_3$) δ -5.3, 26.1, 157.3.

Minor rotamer: **¹H NMR** (500 MHz, $CDCl_3$) δ 1.02 (d, *J* = 6.7 Hz, 3H), 2.96 (s, 3H), 3.70 (s, 3H), 3.98 (t, *J* = 10.7 Hz, 1H). **¹³C NMR** (126 MHz, $CDCl_3$) δ -3.4, 25.8, 156.5.

HRMS (ESI) calcd for $C_{46}H_{74}N_5O_9Si^+$ $(M+H)^+$: 868.525; found: 868.5202.

Methyl *N*-((2*S*,4*R*)-2-((*S*)-2-(((allyloxy)carbonyl)amino)-*N*-methyl-3-(1-(prop-2-yn-1-yl)-1*H*-indol-3-yl)propanamido)-5-((*tert*-butyldimethylsilyl)oxy)-4-methylpentanoyl)-L-alanyl-L-phenylalanyl-L-valyl-*N*-methyl-L-leucinate (SI-70)

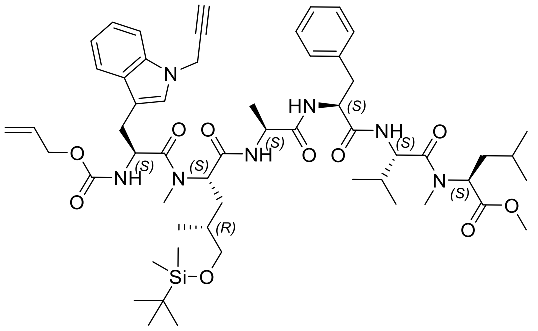

Prepared according to *GP1* and *GP5*: **SI-69** (300 mg, 346 µmol), Pd/C (30.0 mg) (1 h); Alloc-Trp(propargyl)-OH (151 mg, 463 µmol), 1 M iPrOCOCl (450 µl, 450 µmol), DIPEA (79.0 µl, 450 µmol), $BnNMe_2$ (5.14 µl, 34.6 µmol), NMI (5.52 µl, 69.2 µmol), 4 M HCl (8.65 µl, 34.6 µmol) (16 h). Flash chromatography (CyH/EtOAc 100:0 – 4:6) followed by lyophilization yielded **SI-70** (263 mg, 252 µmol, 73%) as a white amorphous solid. R_f = 0.23 (PE/EtOAc 4:6).

$[\alpha]_{20}^{D}$ = –62.7 (c 1.0, $CHCl_3$).

¹H NMR (500 MHz, $CDCl_3$) *(mixture of rotamers, ratio ca. 2:1)* δ -0.06 (d, *J* = 9.0 Hz, 1.3H), -0.02 – 0.07 (m, 6.6H), 0.37 (d, *J* = 6.7 Hz, 0.6H), 0.74 – 0.82 (m, 5.2H), 0.82 – 0.92 (m, 17.9H), 0.92 – 1.00 (m, 8.3H), 1.18 – 1.33 (m, 5.5H), 1.34 – 1.48 (m, 2.8H), 1.50 – 1.61 (m, 0.8H), 1.62 – 1.79 (m, 2.5H), 1.81 – 2.14 (m, 3.6H), 2.31 – 2.45 (m, 1.0H), 2.75 (s, 0.7H), 2.77 – 2.87 (m, 3.2H), 2.92 – 3.02 (m, 4.5H), 3.02 – 3.23 (m, 3.3H), 3.25 – 3.44 (m, 2.6H), 3.47 – 3.53 (m, 0.3H), 3.63 – 3.73 (m, 3.9H), 4.20 – 4.42 (m, 1.8H), 4.42 – 4.67 (m, 3.5H), 4.67 – 4.88 (m, 4.7H), 4.88 – 5.05 (m, 1.0H), 5.12 – 5.37 (m, 3.4H), 5.66 (d, *J* = 7.6 Hz, 0.2H), 5.79 – 5.95 (m, 1.0H), 6.38 (d, *J* = 7.3 Hz, 0.5H), 6.42 (d, *J* = 8.6 Hz, 0.5H), 6.52 (d, *J* = 8.7 Hz, 0.3H), 6.75 (d, *J* = 7.6 Hz, 0.3H), 6.90 (d, *J* = 9.1 Hz, 0.5H), 7.04 (s, 1.0H), 7.10 – 7.31 (m, 9.8H), 7.33 – 7.39 (m, 1.0H), 7.42 – 7.49 (m, 0.3H), 7.51 – 7.56 (m, 0.2H), 7.58 (d, *J* = 7.9 Hz, 0.2H), 7.63 – 7.68 (m, 0.4H), 7.70 (d, *J* = 8.6 Hz, 0.6H), 7.84 (d, *J* = 7.1 Hz, 0.2H).

¹³C NMR (126 MHz, $CDCl_3$) *(mixture of rotamers)* δ -5.3, -5.2, -3.4, 15.9, 17.2, 17.37 17.40, 17.5, 17.6, 17.7, 18.4, 18.48, 18.51, 19.4, 19.6, 21.6, 22.3, 23.4, 24.9, 25.8, 26.0, 26.08, 26.11, 28.5, 28.8, 29.3, 31.29, 31.31, 31.35, 31.38, 31.42, 31.6, 31.9, 32.2, 32.4, 34.5, 35.7, 35.8, 37.0, 37.1, 37.6, 37.9, 49.56, 49.61, 51.7, 52.3, 54.1, 54.17, 54.23, 54.3, 54.37, 54.44, 54.57, 54.60, 54.64, 58.2, 58.9, 65.8, 66.4, 67.6, 68.4, 69.7, 73.7, 73.8, 77.7, 78.0, 109.6, 109.7, 110.3, 117.7, 118.4, 119.0, 119.2, 120.0, 120.2, 122.3, 122.6, 126.2, 126.3, 126.9, 127.0, 128.55, 128.58, 128.60, 128.65, 128.68, 128.74, 129.38, 129.42, 129.5, 132.05, 132.08, 132.19, 132.23, 132.3, 133.0, 136.0, 136.2, 136.4, 136.8, 156.3, 156.7, 168.9, 170.3, 170.4, 170.5, 171.7, 171.8, 172.0, 172.15, 172.19, 172.22, 172.5, 172.6, 173.1.

Selected diagnostic peaks:

Major rotamer: **¹H NMR** (500 MHz, $CDCl_3$) δ 0.01 (s, 3H), 0.02 (s, 3H), 0.87 (s, 9H), 2.38 (t, *J* = 2.5 Hz, 1H), 2.85 (s, 3H), 2.99 (s, 3H), 3.68 (s, 3H), 6.38 (d, *J* = 7.3 Hz, 1H), 6.42 (d, *J* = 8.6 Hz, 1H), 6.90 (d, *J* = 9.1 Hz, 1H), 7.04 (s, 1H). **¹³C NMR** (126 MHz, $CDCl_3$) δ 26.1, 73.7, 78.0, 156.3

Minor rotamer: **¹H NMR** (500 MHz, $CDCl_3$) δ -0.07 (s, 3H), -0.04 (s, 3H), 0.37 (d, *J* = 6.7 Hz, 3H), 0.81 (s, 9H), 2.75 (s, 3H), 2.79 (s, 3H), 2.94 (s, 3H), 3.67 (s, 3H), 5.66 (d, *J* = 7.6 Hz, 1H), 6.52 (d, *J* = 8.7 Hz, 1H), 6.75 (d, *J* = 7.6 Hz, 1H), 7.84 (d, *J* = 7.1 Hz, 1H). **¹³C NMR** (126 MHz, $CDCl_3$) δ 73.8, 77.7, 156.7

HRMS (ESI) calcd for $C_{56}H_{84}N_7O_{10}Si^+$ $(M+H)^+$: 1042.6043; found: 1042.6003.

methyl *N*-((2*S*,4*R*)-2-((*S*)-2-((*S*)-2-(((allyloxy)carbonyl)amino)-3-methylbutanamido)-*N*-methyl-3-(1-(prop-2-yn-1-yl)-1*H*-indol-3-yl)propanamido)-5-((*tert*-butyldimethylsilyl)oxy)-4-methylpentanoyl)-L-alanyl-L-phenylalanyl-L-valyl-*N*-methyl-L-leucinate (SI-71)

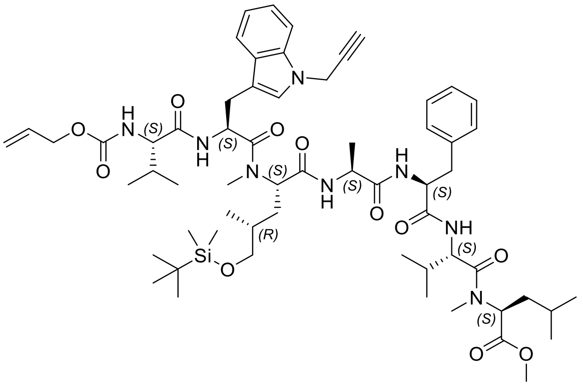

Prepared according to *GP2* and *GP4*: **SI-70** (234 mg, 224 µmol), $Pd(PPh_3)_4$ (2.59 mg, 2.25 µmol), DMBA (105 mg, 673 µmol) (1 h); Alloc-Val-OH (54.1 mg, 269 µmol), HOBt (37.7 mg, 246 µmol), EDC (47.2 mg, 246 µmol), NMM (27.1 µl, 246 µmol) (17 h). Flash chromatography (CyH/EtOAc 100:0 – 3:7) followed by lyophilization yielded **SI-71** (200 mg, 175 µmol, 78%) as a white amorphous solid. R_f = 0.19 (PE/EtOAc 4:6).

$[\alpha]_{20}^{D}$ = −61.0 (c 1.0, $CHCl_3$).

^{1}H NMR (500 MHz, $CDCl_3$) *(mixture of rotamers, ratio ca. 3:2)* δ -0.07 – -0.04 (m, 1.6H), -0.03 – 0.06 (m, 6.2H), 0.36 (d, *J* = 6.6 Hz, 0.7H), 0.74 (d, *J* = 6.4 Hz, 2.0H), 0.79 – 0.83 (m, 4.2H), 0.83 – 1.00 (m, 36.5H), 1.19 – 1.24 (m, 2.8H), 1.24 – 1.31 (m, 3.5H), 1.33 – 1.60 (m, 4.2H), 1.61 – 1.79 (m, 3.1H), 1.81 – 1.93 (m, 0.8H), 1.93 – 2.15 (m, 4.7H), 2.37 (t, *J* = 2.5 Hz, 0.6H), 2.39 (t, *J* = 2.6 Hz, 0.4H), 2.70 (s, 0.8H), 2.79 (s, 0.9H), 2.80 – 2.88 (m, 2.6H), 2.93 – 3.03 (m, 5.1H), 3.03 – 3.21 (m, 4.0H), 3.23 – 3.35 (m, 1.8H), 3.35 – 3.44 (m, 1.2H), 3.51 (dd, *J* = 9.8, 4.6 Hz, 0.4H), 3.64 – 3.71 (m, 4.5H), 4.03 – 4.09 (m, 0.4H), 4.20 – 4.45 (m, 2.7H), 4.47 – 4.63 (m, 3.1H), 4.63 – 4.88 (m, 5.6H), 4.89 – 5.00 (m, 0.7H), 5.15 – 5.23 (m, 1.3H), 5.24 – 5.35 (m, 3.1H), 5.55 (d, *J* = 9.2 Hz, 0.5H), 5.82 – 5.97 (m, 1.3H), 6.28 – 6.54 (m, 0.7H), 6.57 (d, *J* = 8.8 Hz, 0.3H), 6.61 – 6.74 (m, 0.6H), 6.75 – 6.92 (m, 1.3H), 6.99 – 7.08 (m, 1.5H), 7.09 – 7.29 (m, 10.9H), 7.30 – 7.39 (m, 1.6H), 7.43 – 7.49 (m, 0.7H), 7.51 – 7.57 (m, 0.6H), 7.63 – 7.76 (m, 1.8H), 8.07 (d, *J* = 7.8 Hz, 0.3H).

^{13}C NMR (126 MHz, $CDCl_3$) *(mixture of rotamers)* δ -5.3, -5.2, -3.4, 15.9, 17.3, 17.5, 17.8, 17.9, 18.4, 18.46, 18.50, 19.3, 19.4, 19.52, 19.54, 21.5, 21.6, 22.3, 23.3, 23.4, 24.92, 24.94, 25.8, 26.0, 26.07, 26.10, 26.13, 28.8, 29.3, 31.29, 31.34, 31.38, 31.40, 31.5, 31.7, 31.85, 31.89, 32.5, 35.7, 35.8, 37.0, 37.7, 49.1, 49.6, 50.0, 52.2, 52.3, 54.0, 54.1, 54.2, 54.4, 54.5, 54.59, 54.64, 54.8, 57.1, 58.3, 59.9, 60.1, 65.8, 66.1, 67.6, 68.4, 69.7, 73.7, 73.8, 77.8, 78.0, 109.5, 109.6, 109.7, 110.3, 117.7, 118.1, 118.9, 119.3, 120.0, 120.2, 122.3, 122.6, 126.1, 126.3, 127.0, 127.1, 128.2, 128.5, 128.5, 128.6, 128.6, 128.66, 128.68, 129.37, 129.41, 129.44, 129.5, 132.05, 132.08, 132.2, 132.3, 132.9, 133.0, 136.0, 136.1, 136.5, 136.8, 156.1, 156.5, 168.5, 170.1, 170.3, 170.7, 171.4, 171.7, 171.9, 172.0, 172.12, 172.15, 172.2, 172.3, 172.5, 172.7.

Selected diagnostic peaks:

Major rotamer: **^{1}H NMR** (500 MHz, $CDCl_3$) δ -0.01 (s, 3H), 0.00 (s, 3H), 0.74 (d, *J* = 6.4 Hz, 3H), 0.86 (s, 9H), 2.37 (t, *J* = 2.5 Hz, 1H), 2.86 (s, 3H), 2.99 (s, 3H), 3.51 (dd, *J* = 9.8, 4.6 Hz, 0H), 3.67 (s, 3H), 5.55 (d, *J* = 9.2 Hz, 1H), 7.03 (s, 1H). **^{13}C NMR** (126 MHz, $CDCl_3$) δ 24.94, 26.10, 73.7, 78.04, 156.1.

Minor rotamer: **^{1}H NMR** (500 MHz, $CDCl_3$) δ -0.06 (s, 3H), -0.04 (s, 3H), 0.36 (d, *J* = 6.6 Hz, 3H), 0.82 (s, 9H), 2.39 (t, *J* = 2.6 Hz, 1H), 2.70 (s, 3H), 2.79 (s, 3H), 2.95 (s, 3H), 3.51 (dd, *J* = 9.8, 4.6 Hz, 1H), 3.67 (s, 3H), 6.57 (d, *J* = 8.8 Hz, 1H), 7.04 (s, 1H), 8.07 (d, *J* = 7.8 Hz, 1H). **^{13}C NMR** (126 MHz, $CDCl_3$) δ 24.92, 73.8, 77.8, 156.5.

HRMS (ESI) calcd for $C_{61}H_{93}N_8O_{11}Si^+$ $(M+H)^+$: 1141.6728; found: 1141.6744.

(3*S*,6*S*,9*S*,12*S*,15*S*,18*S*,21*S*)-15-benzyl-21-((*R*)-3-hydroxy-2-methylpropyl)-9-isobutyl-6,12-diisopropyl-1,10,18-trimethyl-3-((1-(prop-2-yn-1-yl)-1*H*-indol-3-yl)methyl)-1,4,7,10,13,16,19-heptaazacyclohenicosane-2,5,8,11,14,17,20-heptaone (SI-72)

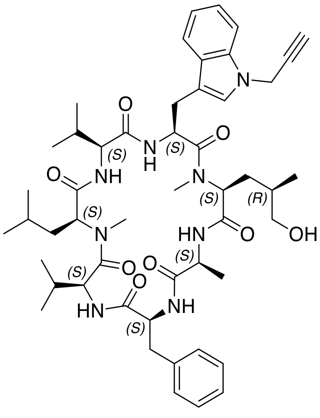

Prepared according to modified *GP6a*: **SI-71** (68.9 mg, 60.4 µmol), 1 M LiOH (96.1 µl, 96.1 µmol) (4.5 h); $Pd(OAc)_2$ (0.7 mg, 3.0 µmol), TPPTS (3.4 mg, 6.0 µmol), Et_2NH (31.6 µl, 302 µmol) (3 h); HATU (80.0 mg, 211 µmol), HOAt (16.4 mg, 121 µmol), DIPEA (42.2 µl, 242 µmol) (17 h); Deprotection: The crude cyclic peptide was dissolved in THF (0.6 ml), 1 M TBAF in THF (133 µl, 133 µmol) was added at 0 °C. After 2.5 h, the mixture was quenched with 1 M $KHSO_4$ soln., extracted with EtOAc (3x). The combined org phases were washed with 1 M $KHSO_4$ and brine, dried (Na_2SO_4) and evaporated. Preparative TLC ($CHCl_3$/MeOH 94:6) followed by RP flash chromatography (H_2O/MeCN 90:10 – 5:95) and lyophilization afforded **SI-72** (5.4 mg, 5.93 µmol, 10%) as a colorless amorphous solid. R_f = 0.26 ($CHCl_3$/MeOH 95:5).

$[\alpha]_{20}^{D}$ = –95.9 (c 1.0, $CHCl_3$).

^{1}H NMR (500 MHz, $CDCl_3$) δ 0.03 (ddd, *J* = 15.3, 8.9, 2.7 Hz, 1H), 0.61 (d, *J* = 6.7 Hz, 3H), 0.87 (d, *J* = 6.2 Hz, 1H), 0.90 (d, *J* = 6.7 Hz, 3H), 0.97 (t, *J* = 6.3 Hz, 6H), 1.03 (t, *J* = 6.8 Hz, 6H), 1.08 (d, *J* = 6.8 Hz, 3H), 1.18 (d, *J* = 7.4 Hz, 3H), 1.31 (ddd, *J* = 13.8, 7.0, 1.6 Hz, 2H), 1.66 – 1.73 (m, 1H), 1.99 – 2.10 (m, 3H), 2.28 (ddd, *J* = 13.9, 8.0, 5.9 Hz, 1H), 2.38 (t, *J* = 2.5 Hz, 1H), 2.79 (s, 3H), 2.80 (s, 3H), 2.94 (dd, *J* = 11.7, 2.7 Hz, 1H), 2.96 – 3.03 (m, 1H), 3.11 (dd, *J* = 13.6, 10.0 Hz, 1H), 3.19 (dd, *J* = 14.4, 4.9 Hz, 1H), 3.43 – 3.53 (m, 3H), 4.18 (pent, *J* = 7.1 Hz, 1H), 4.32 – 4.38 (m, 1H), 4.42 (t, *J* = 9.8 Hz, 1H), 4.53 (dd, *J* = 8.1, 5.5 Hz, 1H), 4.67 (ddd, *J* = 10.9, 9.0, 4.9 Hz, 1H), 4.76 (dd, *J* = 17.7, 2.6 Hz, 1H), 4.83 (dd, *J* = 17.7, 2.5 Hz, 1H), 5.22 (ddd, *J* = 10.0, 6.8, 4.4 Hz, 1H), 7.14 – 7.21 (m, 2H), 7.22 – 7.27 (m, 2H), 7.27 – 7.37 (m, 7H), 7.67 (d, *J* = 6.8 Hz, 1H), 7.84 (d, *J* = 7.9 Hz, 1H), 8.48 (d, *J* = 9.0 Hz, 1H), 8.85 (d, *J* = 10.0 Hz, 1H).

^{13}C NMR (126 MHz, $CDCl_3$) δ 16.8, 16.9, 17.9, 19.4, 19.7, 20.2, 23.0, 23.4, 25.5, 29.0, 29.8, 30.1, 30.9, 31.8, 32.6, 34.0, 35.8, 38.0, 39.2, 50.0, 50.9, 52.6, 55.7, 59.1, 61.8, 64.4, 67.0, 73.8, 78.0, 109.4, 110.4, 119.8, 120.3, 122.3, 126.1, 126.6, 128.4, 129.0, 129.7, 135.9, 137.9, 169.1, 169.3, 171.15, 171.20, 171.7, 172.7, 174.2.

HRMS (ESI) calcd for $C_{50}H_{71}N_8O_8^+$ $(M+H)^+$: 911.5389; found: 911.5349.

Synthesis of Glutamine-containing dCym derivatives (SI-74, SI-76)

Methyl *N*-(((2*S*,3*R*)-2-((*S*)-2-((2*S*,4*R*)-2-((*S*)-2-((*S*)-2-((((9*H*-fluoren-9-)carbonyl)amino)-5-yl)methoxy(dimethylamino)-5-oxopentanamido)-N-methyl-3-(1-methyl-1*H*-indol-3-yl)propanamido)-5-((tert-butyldimethylsilyl)oxy)-4-methylpentanamido)propanamido)-3-methoxy-3-phenylpropanoyl)-L-valyl)-*N*-methyl-L-leucinate (SI-73)

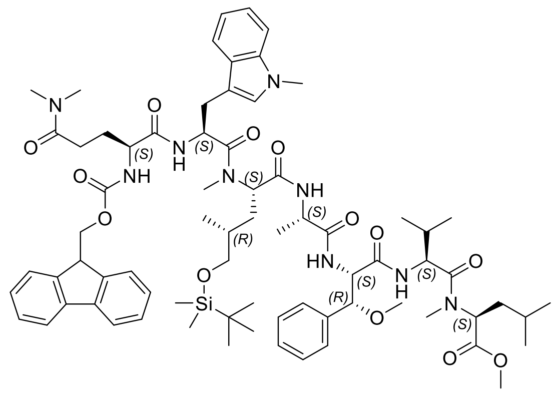

Prepared according to *GP2* and *GP4*: Methyl *N*-(((2S,3R)-2-((S)-2-((2S,4R)-2-((S)-2-(((allyloxy)carbonyl)amino)-N-methyl-3-(1-methyl-1H-indol-3-yl)propanamido)-5-((tert-butyldimethylsilyl)oxy)-4-methylpentanamido)propanamido)-3-methoxy-3-phenylpropanoyl)-L-valyl)-N-methyl-L-leucinate (150 mg, 143 µmol), $Pd(PPh_3)_4$ (4.96 mg, 4.30 µmol), DMBA (67.0 mg, 429 µmol); N^2-(((9*H*-fluoren-9-yl)methoxy)carbonyl)-N^5,N^5-dimethyl-L-glutamine (56.7 mg, 177 µmol), NMM (31.5 µl, 286 µmol), HOBt (23.8 mg, 0.160 µmol), EDC (30.2 mg, 160 µmol) (18 h). Flash chromatography (CyH/EtOAc 100:0 – 60:40) followed by lyophilization afforded **SI-73** (59.0 mg, 44.0 µmol, 31% yield) as a white amorphous solid. R_f = 0.44 (PE/EtOAc 2:3).

$[\alpha]_{20}^{D}$ = –50.8 (c 0.5, $CHCl_3$).

^{1}H NMR (500 MHz, $CDCl_3$) (*mixture of rotamers, ratio ~2:1*) δ -0.06 – 0.05 (m, 8.8H), 0.09 (s, 1.1H), 0.36 (s, 0.19H), 0.47 (d, *J* = 6.7 Hz, 1.2H), 0.82 – 0.86 (m, 3.7H), 0.86 – 0.88 (m, 8.2H), 0.88 – 1.01 (m, 18.6H), 1.14 – 1.21 (m, 2.7H), 1.23 – 1.41 (m, 2.2H), 1.43 – 1.55 (m, 3.3H), 1.63 – 1.86 (m, 2.9H), 1.88 – 2.20 (m, 3.0H), 2.22 – 2.45 (s, 4.0H), 2.71 (s, 1.22H), 2.77 – 2.83 (m, 2.2H), 2.89 – 3.09 (m, 14.4H), 3.11 – 3.29 (m, 3.2H), 3.30 – 3.33 (m, 3.0H), 3.59 – 3.78 (m, 10.6H), 4.14 – 4.39 (m, 6.9H), 4.63 – 4.87 (m, 4.3H), 4.89 – 5.00 (m, 0.5H), 5.07 – 5.21 (m, 0.9H), 5.28 – 5.44 (m, 1.6H), 6.12 – 6.19 (m, 0.8H), 6.35 – 6.40 (m, 0.4H), 6.51 (d, *J* = 6.8 Hz, 0.7H), 6.77 (d, *J* = 7.5 Hz, 0.4H), 6.83 – 6.91 (m, 0.6H), 6.90 – 7.01 (m, 1.5H), 7.09 (t, *J* = 7.4 Hz, 1.2H), 7.13 – 7.16 (m, 0.9H), 7.16 – 7.24 (m, 2.3H), 7.27 – 7.32 (m, 6.1H), 7.35 – 7.43 (m, 5.2H), 7.54 – 7.68 (m, 4.7H), 7.75 (d, *J* = 7.6 Hz, 3.4H), 8.11 (d, *J* = 7.8 Hz, 0.6H), 8.23 (d, *J* = 6.4 Hz, 0.5H), 8.58 (d, *J* = 6.4 Hz, 0.4H).

^{13}C NMR (126 MHz, $CDCl_3$) (*mixture of rotamers*) δ -5.3, -5.2, -3.4, 17.4, 17.5, 17.6, 18.1, 19.6, 21.5, 23.4, 24.90, 24.95, 25.8, 26.1, 28.2, 28.3, 28.6, 29.29, 29.32, 29.7, 29.8, 31.36, 31.39, 31.41, 31.45, 32.76, 32.79, 33.3, 33.4, 35.78, 35.82, 35.85, 37.0, 37.06, 37.09, 37.41, 37.45, 37.50, 37.52, 47.21, 47.25, 50.2, 50.5, 50.6, 52.2, 54.2, 54.26, 54.30, 54.4, 54.6, 54.7, 57.66, 57.68, 57.8, 67.0, 67.1, 67.3, 68.5, 75.0, 81.48, 81.52, 81.7, 108.5, 108.7, 109.3, 109.4, 109.5, 109.6, 118.6, 118.7, 118.9, 119.3, 119.4, 119.5, 120.1, 121.88, 121.92, 122.1, 125.3, 125.4, 127.0, 127.16, 127.19, 127.21, 127.79, 127.82, 127.85, 128.1, 128.31, 128.35, 128.49, 128.52, 136.9, 137.0, 141.37, 141.39, 143.8, 144.1, 156.3, 156.4, 168.60, 168.62, 168.7, 171.6, 171.7, 171.9, 172.1, 172.19, 172.21.

Selected diagnostic peaks:

Major rotamer: **^{1}H NMR** (500 MHz, $CDCl_3$) δ 0.01 (s, 3H), 0.02 (s, 3H), 0.87 (s, 9H), 3.32 (s, 3H), 3.68 (s, 3H), 5.30 – 5.40 (m, 1H), 6.51 (d, *J* = 6.8 Hz, 1H), 6.96 (s, 1H). **^{13}C NMR** (126 MHz, $CDCl_3$) δ 24.95, 47.21, 156.3.

Minor rotamer: **^{1}H NMR** (500 MHz, $CDCl_3$) δ-0.03 (s, 3H), -0.01 (s, 3H), 0.47 (d, *J* = 6.7 Hz, 3H), 0.84 (s, 9H), 2.71 (s, 3H), 2.82 (s, 3H), 3.22 (s, 3H), 6.38 (d, *J* = 7.0 Hz, 1H), 8.58 (d, *J* = 6.5 Hz, 1H). **^{13}C NMR** (126 MHz, $CDCl_3$) δ 24.90, 47.25, 156.4.

HRMS (ESI): calcd for $C_{73}H_{104}N_9O_{13}Si^+$ $(M+H)^+$: 1342.7517; found: 1342.7485.

3-((2*S*,5*S*,8*S*,11*S*,14*S*,17*S*,20*S*)-8-((*R*)-3-Hydroxy-2-methylpropyl)-20-isobutyl-17-isopropyl-14-((*R*)-methoxy(phenyl)methyl)-7,11,19-trimethyl-5-((1-methyl-1*H*-indol-3-yl)methyl)-3,6,9,12,15,18,21-heptaoxo-1,4,7,10,13,16,19-heptaazacyclohenicosan-2-yl)-*N*,*N*-dimethylpropanamide (SI-74)

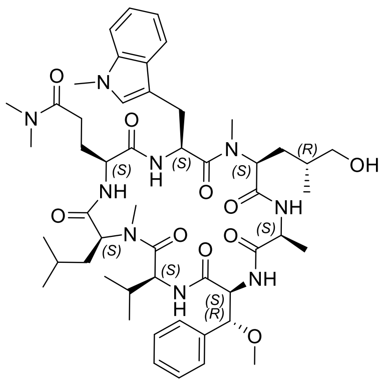

Prepared according to *GP6b*: **SI-73** (106 mg, 80.0 µmol), tris(2-aminoethyl)amine (118 µl, 550 µmol)); LiOH (95.0 µl, 95.0 µmol); HATU (105 mg, 270 µmol), DIPEA (54.0 µl, 310 µmol) (addition over 1.5 h, additional 16 h); NH_4F (58.5 mg, 1.60 mmol). RP flash chromatography (H_2O/MeCN 90:10 - 5:95) followed by lyophilizaiton afforded **SI-74** (18.8 mg, 19.0 µmol, 24%) as an off-white, amorphous solid.

$[\alpha]_{20}^{D}$ = –58.4 (c 0.4, $CHCl_3$).

^{1}H NMR (500 MHz, $CDCl_3$) δ -0.44 – -0.36 (m, 1H), 0.34 (d, *J* = 6.7 Hz, 3H), 0.94 – 0.96 (m, 8H), 1.06 (d, *J* = 6.6 Hz, 3H), 1.23 (d, *J* = 7.2 Hz, 3H), 1.45 – 1.57 (m, 1H), 1.83 – 1.93 (m, 1H), 1.96 – 2.07 (m, 1H), 2.14 – 2.25 (m, 6H), 2.57 (s, 3H), 2.87 (s, 3H), 2.95 (m, 3H), 2.97 – 3.07 (m, 6H), 3.02 – 3.17 (m, 3H), 3.19 – 3.32 (m, 2H), 3.33 (s, 3H), 3.71 (s, 3H), 4.43 – 4.64 (m, 3H), 4.70 – 4.76 (m, 1H), 4.77 – 4.89 (m, 2H), 4.92 – 4.98 (m, 1H), 5.01 (d, *J* = 5.3 Hz, 1H), 6.85 (s, 1H), 7.05 – 7.12 (m, 1H), 7.12 – 7.18 (m, 2H), 7.20 (m, 4H), 7.34 – 7.39 (m, 1H), 7.43 – 7.53 (m, 1H), 7.77 (d, *J* = 5.3 Hz, 1H), 8.02 (d, *J* = 8.5 Hz, 1H), 8.10 (d, *J* = 8.9 Hz, 1H), 8.20 (d, *J* = 10.2 Hz, 1H).

^{13}C NMR (126 MHz, $CDCl_3$) δ 17.3, 19.4, 20.0, 21.4, 22.6, 23.7, 25.2, 28.60, 28.62, 29.1, 29.4, 30.8, 31.7, 32.8, 33.2, 35.8, 37.5, 39.2, 50.8, 51.0, 51.9, 55.2, 56.1, 57.9, 58.8, 59.4, 66.2, 80.3, 108.2, 109.7, 118.8, 119.6, 122.3, 127.7, 127.9, 128.28, 128.33, 128.6, 128.8, 135.6, 136.9, 168.2, 169.7, 169.8, 170.8, 171.7, 171.9, 172.0, 172.2.

HRMS (ESI): calcd for $C_{51}H_{75}N_9O_{10}$ $(M+H)^+$: 974.5710; found: 974.5665.

Methyl *N*-(((2*S*,3*R*)-2-((*S*)-2-((2*S*,4*R*)-2-((*S*)-2-((*S*)-2-(((allyloxy)carbonyl)amino)-5-(dimethylamino)-*N*-methyl-5-oxopentanamido)-*N*-methyl-3-(1-methyl-1*H*-indol-3-yl)propanamido)-5-((*tert*-butyldimethylsilyl)oxy)-4-methylpentanamido)propanamido)-3-methoxy-3-phenylpropanoyl)-L-valyl)-*N*-methyl-L-leucinate (SI-75)

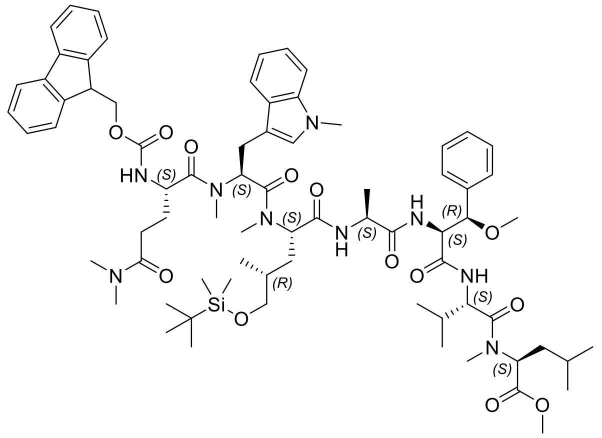

Hexapeptide **18** (98.7 mg, 92.9 µmol) was deprotected accoring to *GP2*: $Pd(PPh_3)_4$ (3.2 mg, 2.8 µmol), DMBA (43.5 mg, 279 µmol) (1 h). The crude deprotected peptide was then dissolved in DMF (0.93 ml), N^2-(((9*H*-fluoren-9-yl)methoxy)carbonyl)-N^5,N^5-dimethyl-L-glutamine (76.5 mg, 193 µmol) was added and the mixture was cooled to 0 °C. HATU (70.7 mg, 186 µmol), HOAt (12.7 mg, 93.3 µmol) and DIPEA (81.2 µl, 465 µmol) were added and the mixture was slowly warmed to rt over the course of 20 h. The mixture was then diluted with EtOAc, washed with 1 M $KHSO_4$, 1 M LiCl (2x), sat. $NaHCO_3$ and sat. NaCl solutions. The organic phase was

dried (Na_2SO_4) and evaporated *in vacuo*. RP flash chromatography (H_2O/MeCN 90:10– 0:100) followed by lyophilization afforded **SI-75** (43.5 mg, 32.1 µmol, 35%) as a white amorphous solid.

$[\alpha]_{20}^{D}$ = –70.6 (c 0.5, $CHCl_3$).

^{1}H NMR (500 MHz, $CDCl_3$) *(mixture of rotamers, ratio ~2:1)* δ -0.17 – -0.08 (m, 0.3H), 0.01 (s, 1.1H), 0.03 (s, 1.1H), 0.06 – 0.14 (m, 3.8H), 0.45 (d, *J* = 6.7 Hz, 0.8H), 0.89 (s, 3.2H), 0.91 – 1.04 (m, 19.2H), 1.05 (d, *J* = 6.8 Hz, 2.2H), 1.27 – 1.43 (m, 2.1H), 1.43 – 1.61 (m, 2.4H), 1.68 – 1.87 (m, 2.7H), 1.92 – 2.10 (m, 2.4H), 2.10 – 2.27 (m, 1.8H), 2.30 – 2.44 (m, 1.0H), 2.51 – 2.63 (m, 0.8H), 2.76 – 2.85 (m, 2.7H), 2.88 – 3.07 (m, 9.1H), 3.07 – 3.12 (m, 0.5H), 3.21 (s, 1.1H), 3.28 (s, 1.4H), 3.35 – 3.42 (m, 2.4H), 3.47 (dd, *J* = 9.7, 5.6 Hz, 0.6H), 3.50 – 3.62 (m, 2.3H), 3.64 – 3.69 (m, 1.7H), 3.70 – 3.79 (m, 4.1H), 4.18 – 4.26 (m, 0.6H), 4.26 – 4.31 (m, 0.5H), 4.32 – 4.48 (m, 2.2H), 4.73 (dd, *J* = 7.5, 3.6 Hz, 0.7H), 4.77 – 4.99 (m, 3.3H), 5.08 – 5.16 (m, 0.5H), 5.38 – 5.45 (m, 0.9H), 5.66 – 5.74 (m, 0.2H), 5.85 – 5.94 (m, 0.9H), 6.34 (d, *J* = 6.9 Hz, 0.5H), 6.45 (d, *J* = 9.1 Hz, 0.2H), 6.68 – 6.78 (m, 0.8H), 6.90 (s, 0.3H), 6.94 – 7.00 (m, 0.5H), 7.12 – 7.19 (m, 1.4H), 7.19 – 7.29 (m, 4.0H), 7.29 – 7.42 (m, 5.3H), 7.42 – 7.50 (m, 2.1H), 7.59 – 7.72 (m, 1.8H), 7.73 – 7.86 (m, 2.6H), 8.12 (d, *J* = 6.7 Hz, 0.2H).

^{13}C NMR (126 MHz, $CDCl_3$) *(mixture of rotamers)* δ -5.3, -5.2, 15.7, 17.2, 17.3, 17.6, 18.4, 18.5, 18.7, 19.7, 21.5, 23.4, 24.9, 25.1, 25.9, 26.0, 26.1, 27.8, 28.5, 28.7, 29.1, 30.7, 30.9, 31.1, 31.29, 31.34, 31.5, 32.0, 32.3, 32.6, 32.7, 35.7, 37.0, 37.2, 47.2, 49.0, 49.8, 51.1, 51.7, 52.3, 53.2, 54.0, 54.2, 54.6, 55.0, 57.3, 57.4, 57.7, 57.9, 67.1, 67.3, 68.1, 81.3, 109.0, 109.4, 118.8, 118.9, 119.2, 119.4, 120.1, 121.7, 122.1, 125.2, 125.3, 126.9, 127.16, 127.20, 127.9, 128.4, 128.5, 136.86, 136.91, 141.4, 143.78, 143.85, 144.1, 156.5, 156.8, 168.2, 168.5, 168.7, 170.3, 171.3, 171.5, 171.6, 171.7, 172.1, 172.2, 172.3, 173.6.

Selected diagnostic peaks:

Major rotamer: **^{1}H NMR** (500 MHz, $CDCl_3$) δ 0.09 (s, 3H), 0.11 (s, 3H), 0.94 (s, 9H), 2.98 (s, 3H), 3.00 (s, 3H), 3.05 (s, 3H), 3.39 (s, 3H), 3.75 (s, 3H), 5.42 (dd, *J* = 10.7, 5.3 Hz, 1H), 6.34 (d, *J* = 6.9 Hz, 1H), 6.73 (d, *J* = 7.7 Hz, 1H), 6.97 (s, 1H). **^{13}C NMR** (126 MHz, $CDCl_3$) δ 26.1, 136.91, 156.5.

Minor rotamer: **^{1}H NMR** (500 MHz, $CDCl_3$) δ 0.01 (s, 3H), 0.03 (s, 3H), 0.45 (d, *J* = 6.7 Hz, 3H), 0.89 (s, 9H), 3.02 (s, 3H), 3.21 (s, 3H), 3.28 (s, 3H), 3.67 (s, 3H), 6.45 (d, *J* = 9.1 Hz, 1H), 6.90 (s, 1H), 8.12 (d, *J* = 6.7 Hz, 1H). **^{13}C NMR** (126 MHz, $CDCl_3$) δ 26.0, 136.86, 156.8.

HRMS (ESI) calcd for $C_{74}H_{106}N_9O_{13}Si^+$ $(M+H)^+$: 1356.7674; found: 1356.7705.

3-((2*S*,5*S*,8*S*,11*S*,14*S*,17*S*,20*S*)-8-((*R*)-3-Hydroxy-2-methylpropyl)-20-isobutyl-17-isopropyl-14-((*R*)-methoxy(phenyl)methyl)-4,7,11,19-tetramethyl-5-((1-methyl-1*H*-indol-3-yl)methyl)-3,6,9,12,15,18,21-heptaoxo-1,4,7,10,13,16,19-heptaazacyclohenicosan-2-yl)-N,N-dimethylpropanamide (SI-76)

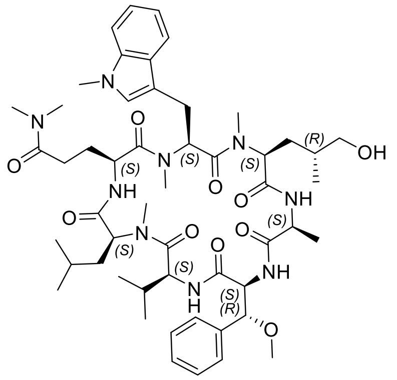

Prepared according to *GP6b*: **SI-75** (74.0 mg, 55.0 µmol), tris(2-aminoethyl)amine (82.0 µl, 550 µmol), LiOH (66.0 µl, 66.0 µmol); HATU (73.0 mg, 200 µmol), DIPEA (38.0 µl, 220 µmol) (addition over 1.5 h, additional 16 h); NH_4F (41.0 mg, 1.10 mmol). RP flash chromatography (H_2O/MeCN 90:10 - 5:95) followed by lyophilizaiton afforded **SI-76** (17.2 mg, 17.0 µmol, 32%) as an off-white, amorphous solid.

$[\alpha]_{20}^{D}$ = –82.6 (c 0.5, $CHCl_3$).

^{1}H NMR (500 MHz, $CDCl_3$) δ -0.25 – -0.11 (m, 1H), 0.38 (d, *J* = 6.7 Hz, 3H), 0.67 – 0.77 (m, 1H), 0.88 – 1.04 (m, 11H), 1.08 (d, *J* = 6.7 Hz, 3H), 1.20 (d, *J* = 7.2 Hz, 3H), 1.34 – 1.43 (m, 1H), 1.49 – 1.57 (m, 1H), 1.77 – 2.17 (m, 7H),

2.18 – 2.32 (m, 2H), 2.62 (s, 3H), 2.87 – 2.99 (m, 9H), 3.06 – 3.21 (m, 2H), 3.27 – 3.31 (m, 1H), 3.31 – 3.42 (m, 6H), 3.70 (s, 3H), 4.55 (t, *J* = 8.9 Hz, 1H), 4.66 (dd, *J* = 10.4, 3.1 Hz, 1H), 4.71 – 4.82 (m, 1H), 4.82 – 4.92 (m, 2H), 4.92 – 5.01 (m, 1H), 5.16 (d, *J* = 5.4 Hz, 1H), 5.41 (dd, *J* = 9.0, 6.4 Hz, 1H), 6.79 (s, 1H), 6.99 – 7.14 (m, 1H), 7.17 – 7.24 (m, 4H), 7.25 (s, 1H), 7.26 – 7.27 (m, 1H), 7.45 (dd, *J* = 8.0, 1.0 Hz, 1H), 8.06 – 8.16 (m, 2H), 8.36 (d, *J* = 9.0 Hz, 1H).

^{13}C NMR (126 MHz, $CDCl_3$) δ 17.4, 19.5, 20.1, 20.6, 22.6, 23.6, 25.2, 26.5, 28.1, 28.9, 29.21, 29.24, 30.9, 31.7, 32.1, 32.8, 33.2, 35.8, 37.1, 39.3, 48.7, 51.0, 53.3, 55.3, 56.1, 58.0, 58.8, 59.3, 66.2, 79.9, 108.7, 109.7, 118.8, 119.6, 122.3, 127.2, 127.7, 128.2, 128.46, 128.53, 135.7, 137.0, 168.1, 169.6, 169.9, 170.8, 171.0, 171.7, 172.1, 173.9.

HRMS (ESI): calcd for $C_{52}H_{78}N_9O_{10}^+$ $(M+H)^+$: 988.5866; found: 988.5861.

Supplementary Fig. 7: SPR Sensorgrams

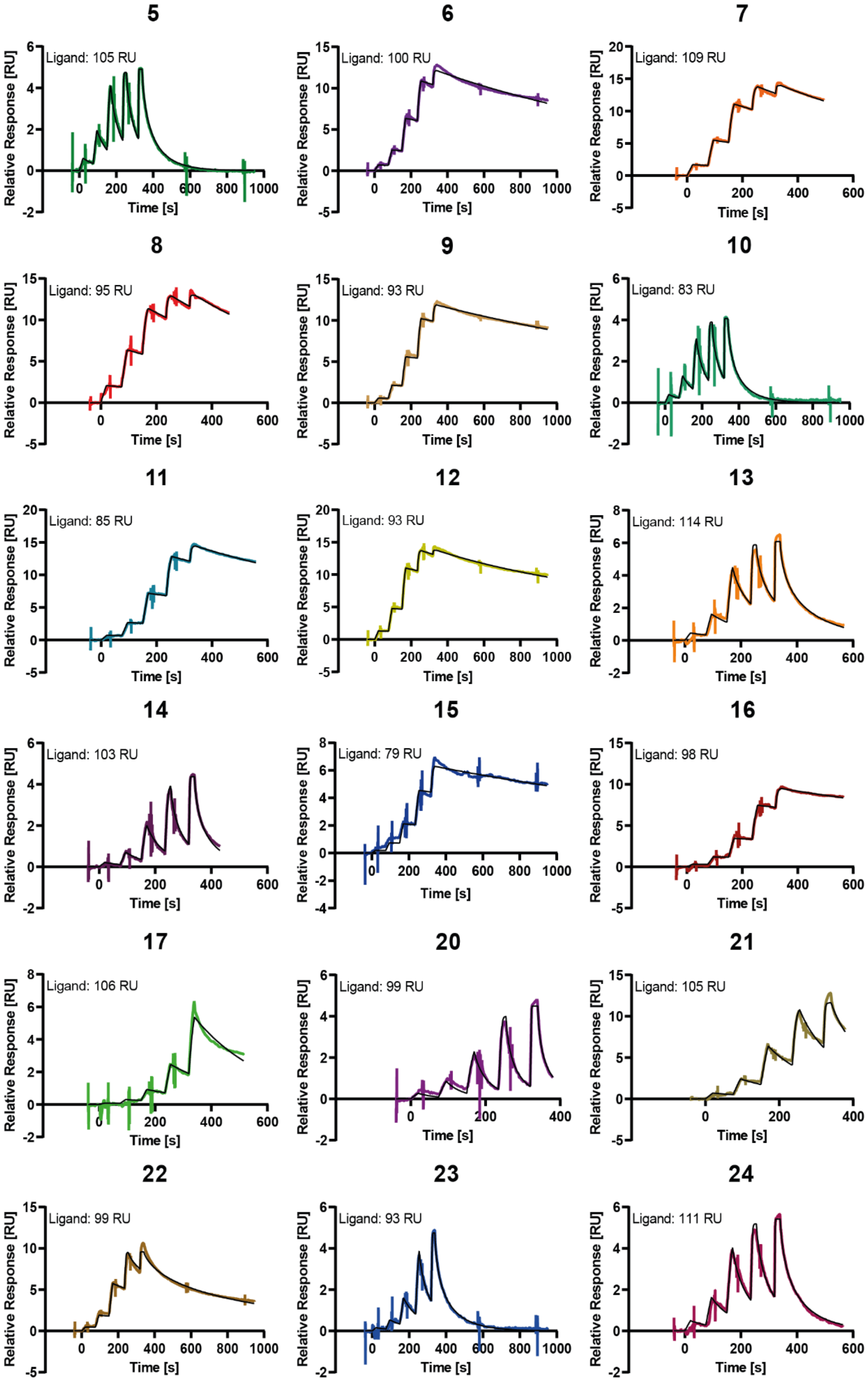

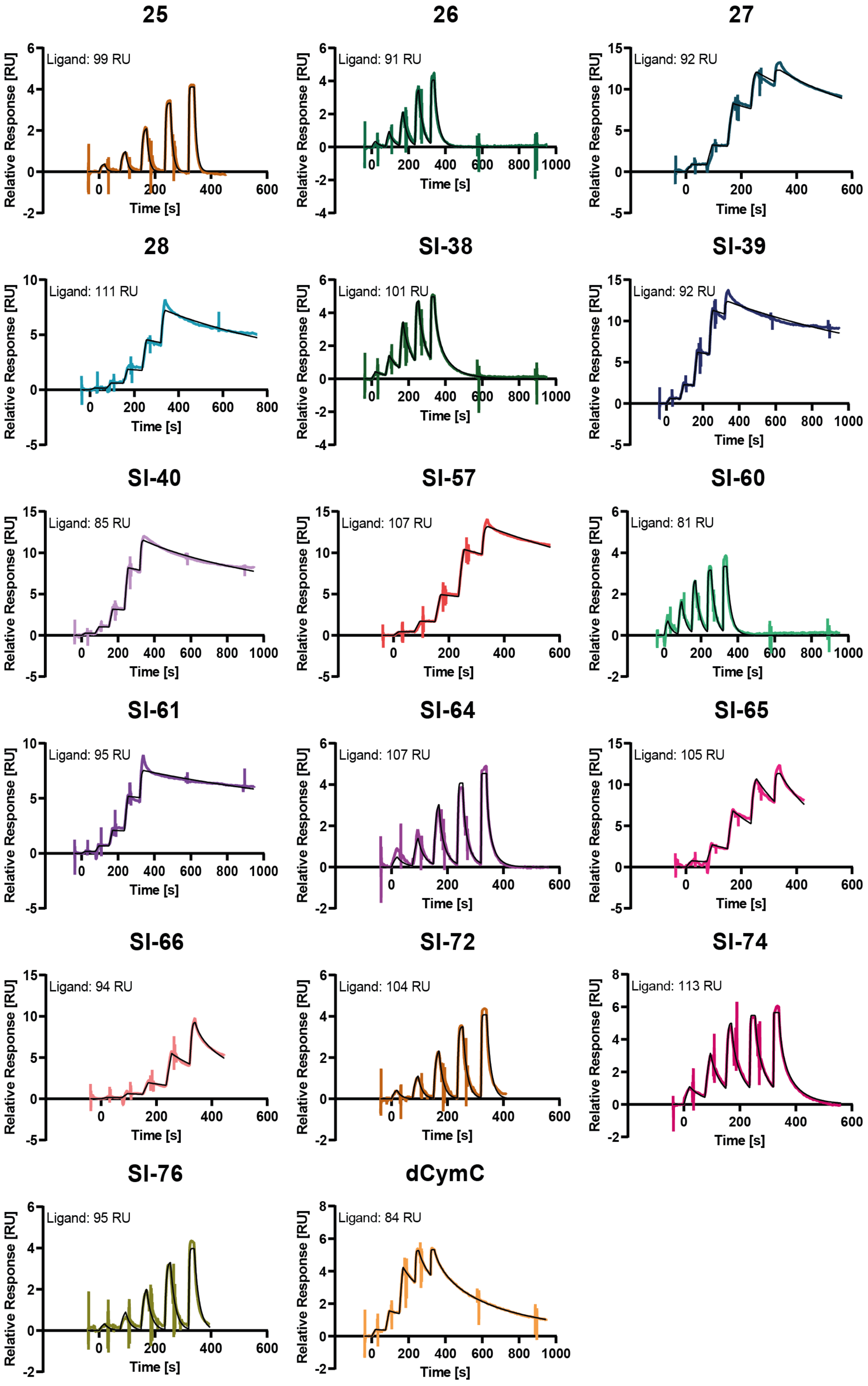

Supplementary Fig. 7. SPR sensorgrams of all compounds introduced in this paper. Each sensorgram is representative of at least two single-cycle measurements per compound. The exact number of independent measurements for each compound can be found in Supplementary Table 1. The biotinylated ligand ClpC1 NTD was captured via streptavidin, which was immobilized to a CM5 chip. The final ligand density used is indicated in each sensorgram. For the single-cycle measurements, five analyte concentrations were injected

consecutively for 20 s each: 2.5 nM, 7.4 nM, 22.2 nM, 66.7 nM, 200 nM. Coloured lines represent the measured raw data, while black lines represent fits calculated by the evaluation software.

Supplementary Fig. 8 – 209: Copies of NMR spectra

Supplementary Fig. 8. ^{1}H NMR spectrum of compound **3**

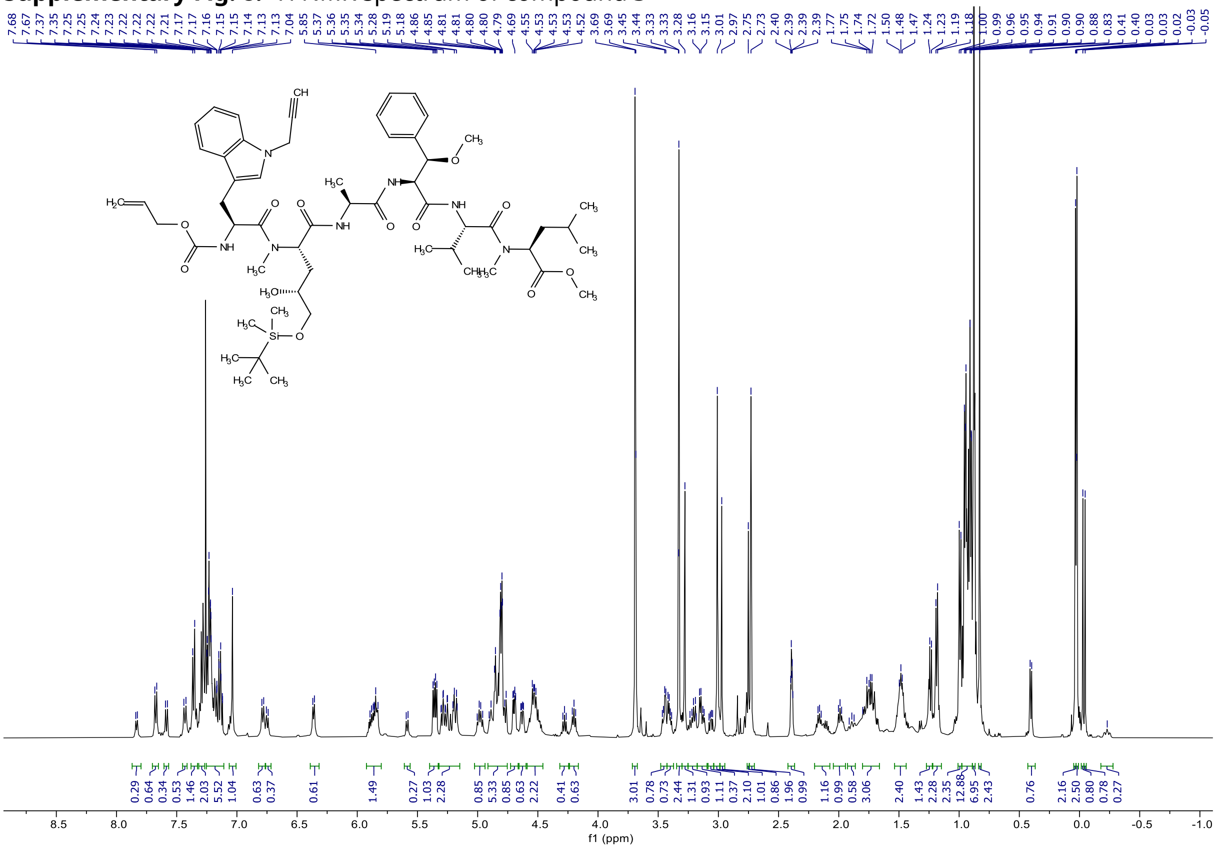

Supplementary Fig. 9. ^{13}C NMR spectrum of compound **3**

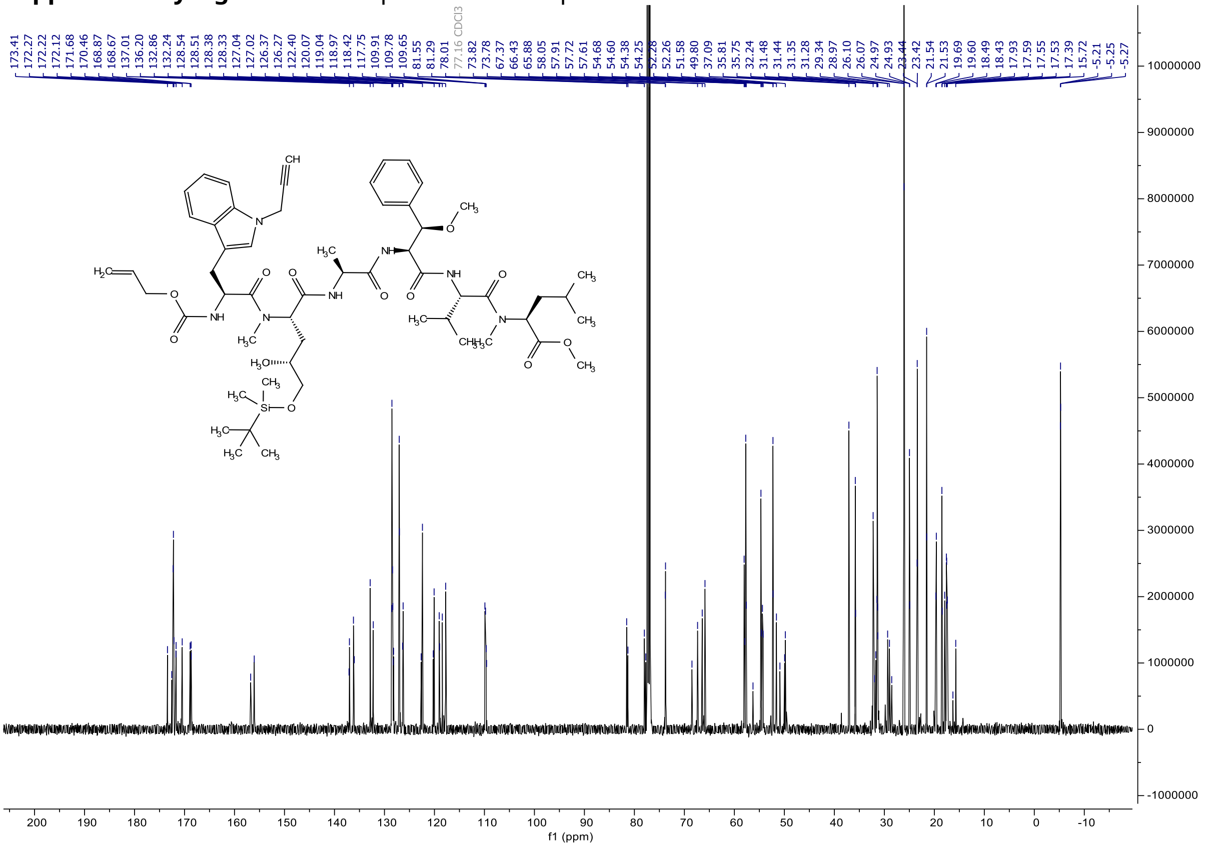

Supplementary Fig. 10. ^{1}H NMR spectrum of compound **4**

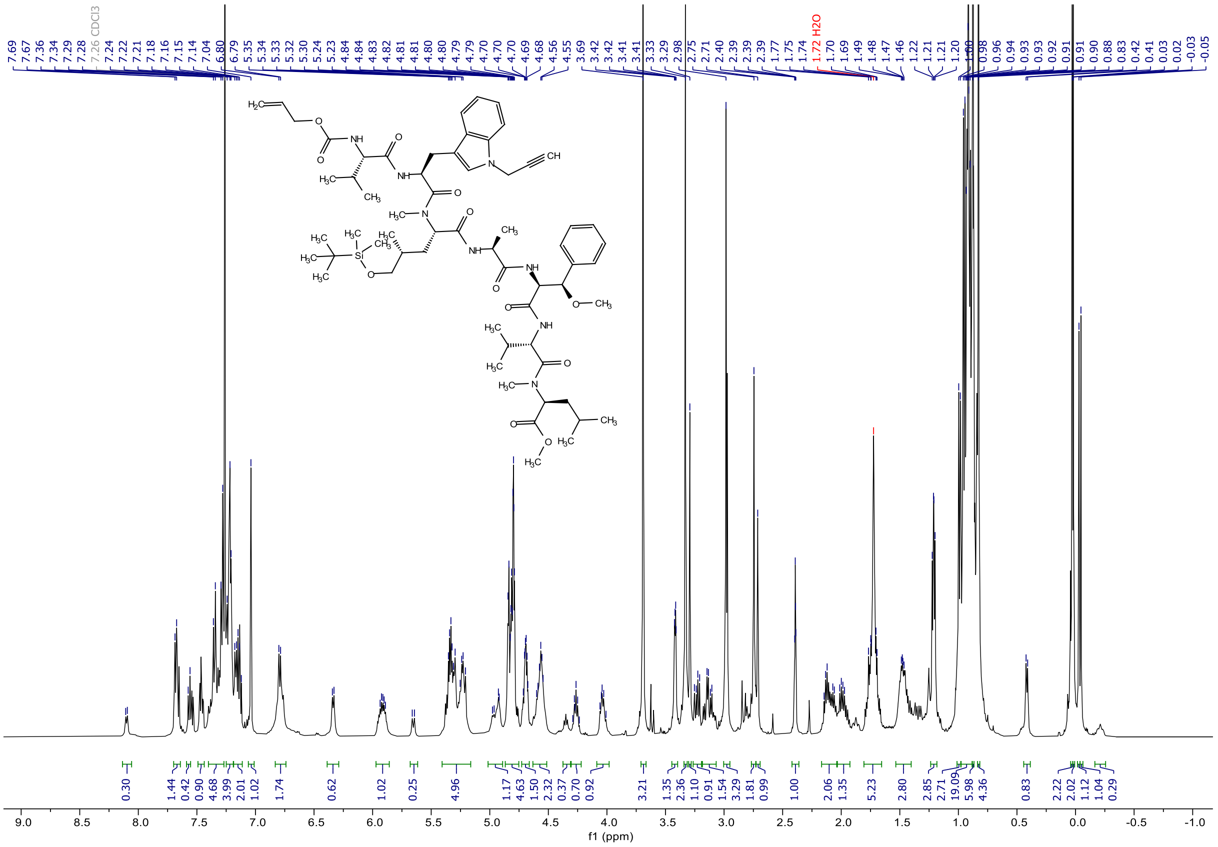

Supplementary Fig. 11. ^{13}C NMR spectrum of compound **4**

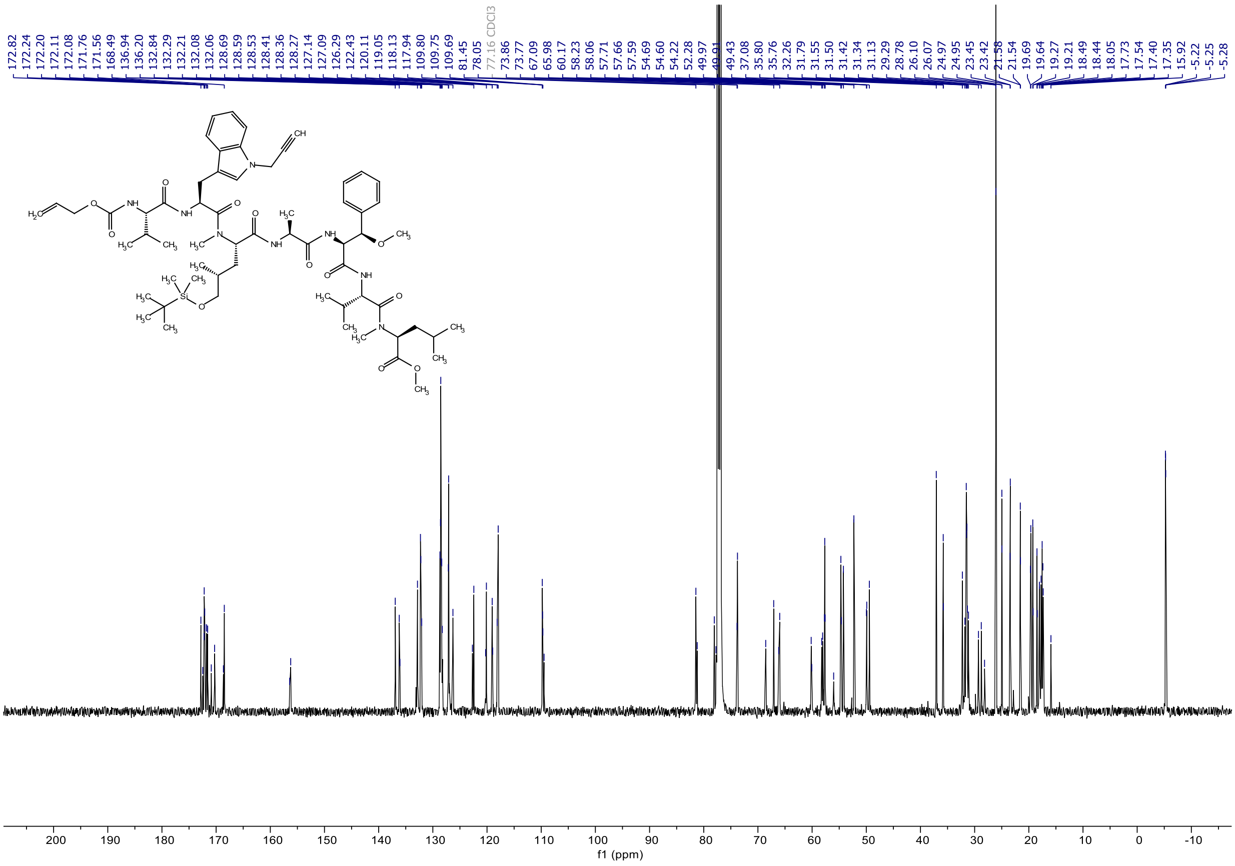

Supplementary Fig. 12. ^{1}H NMR spectrum of compound **5**

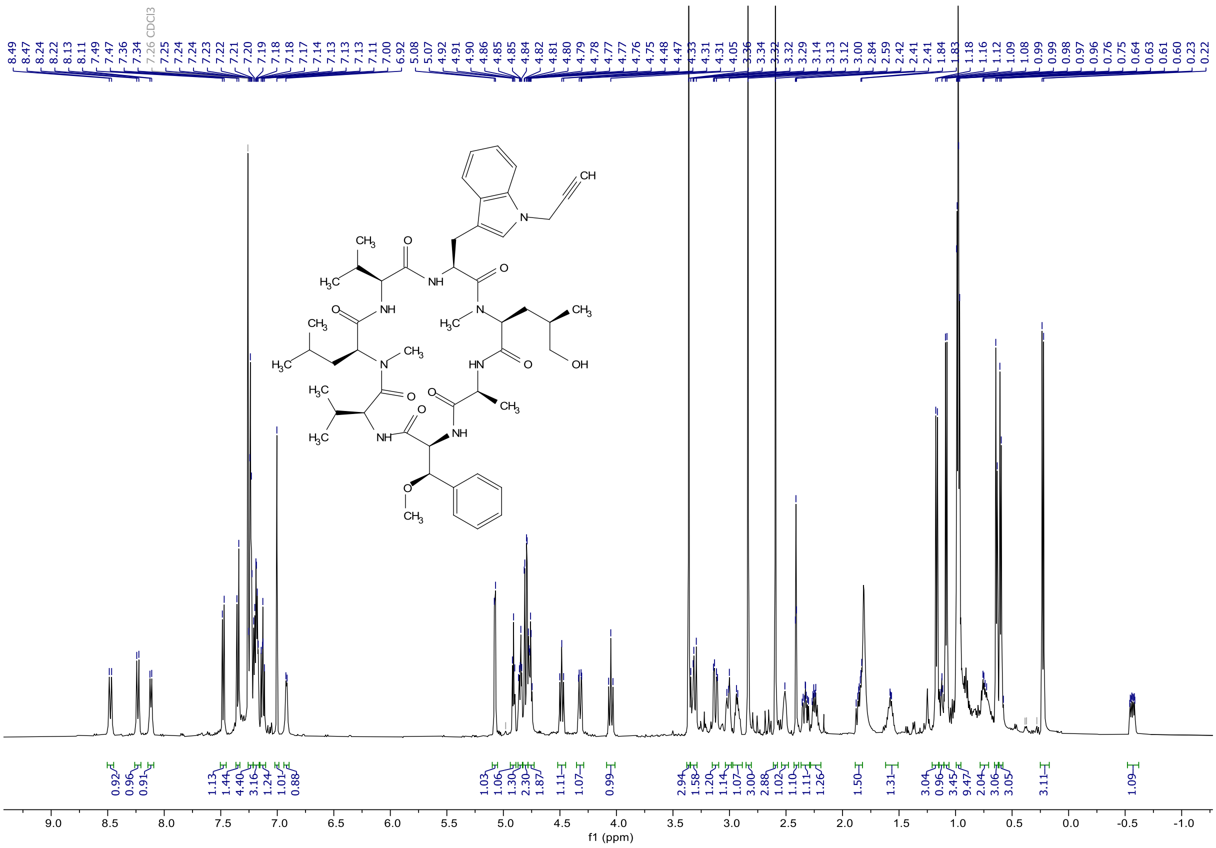

Supplementary Fig. 13. ^{13}C NMR spectrum of compound **5**

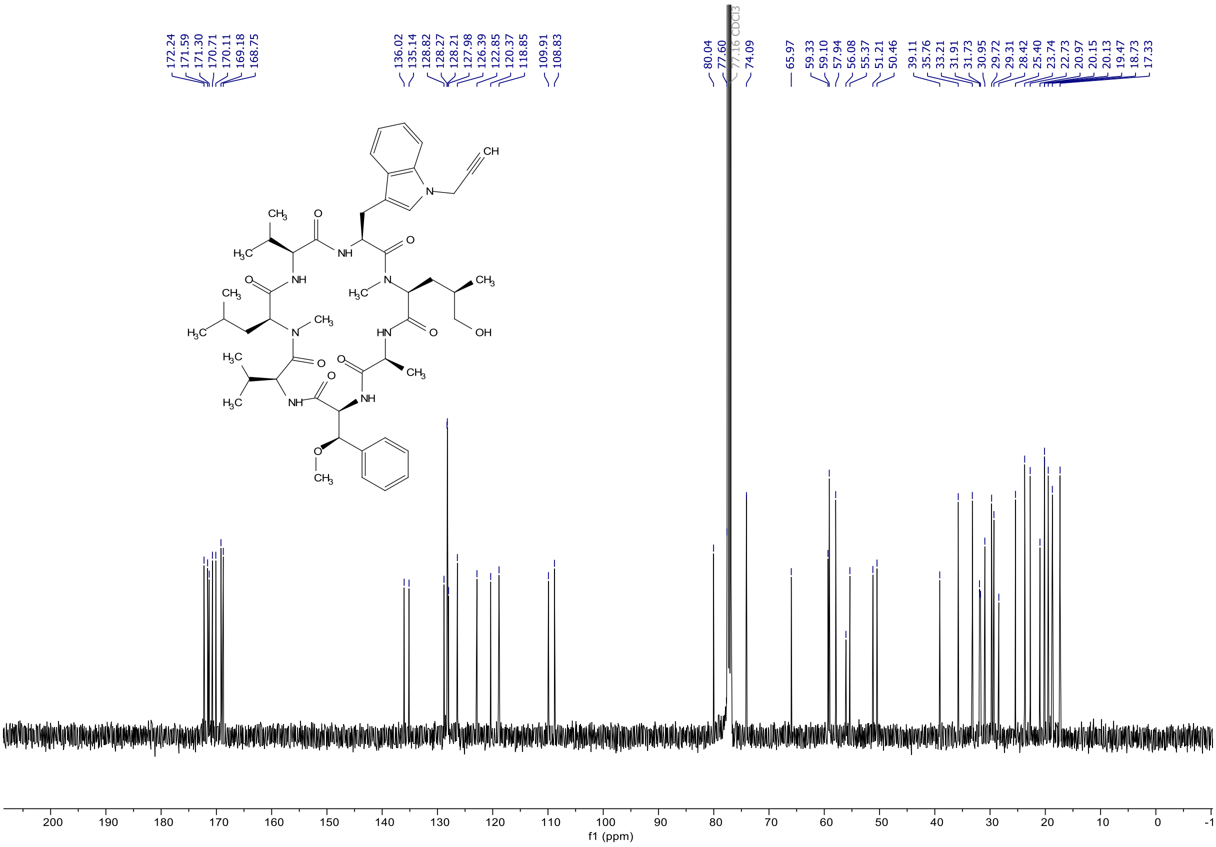

Supplementary Fig. 14. ^{1}H NMR spectrum of compound **6**

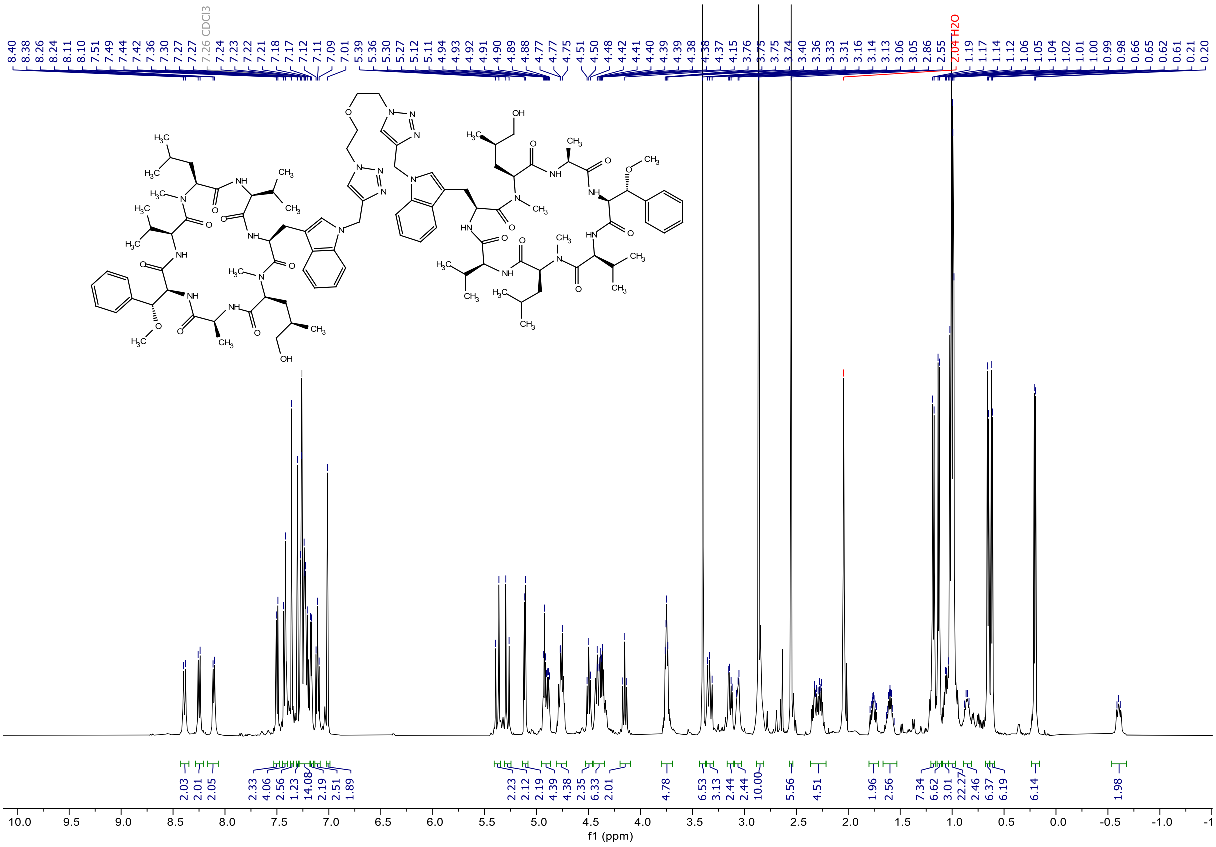

Supplementary Fig. 15. ^{13}C NMR spectrum of compound **6**

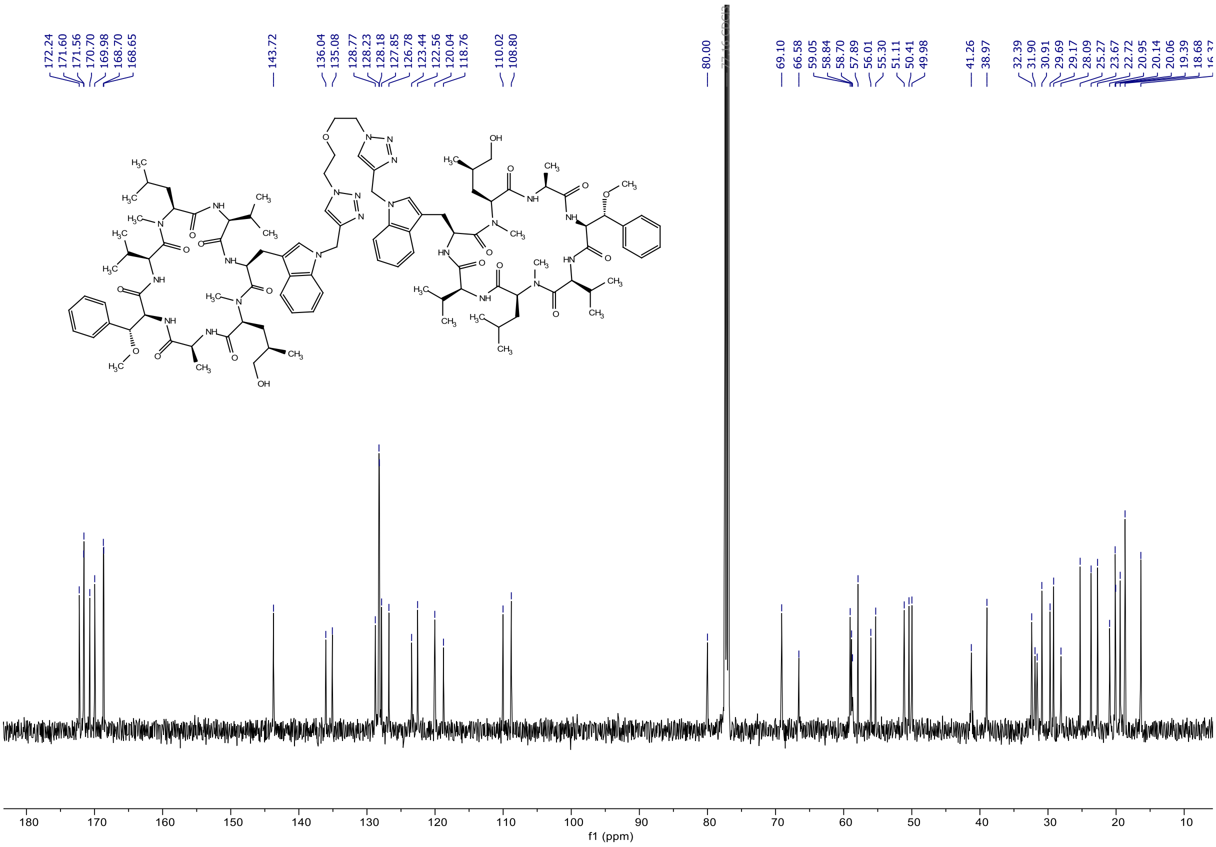

Supplementary Fig. 16. ^{1}H NMR spectrum of compound **7**

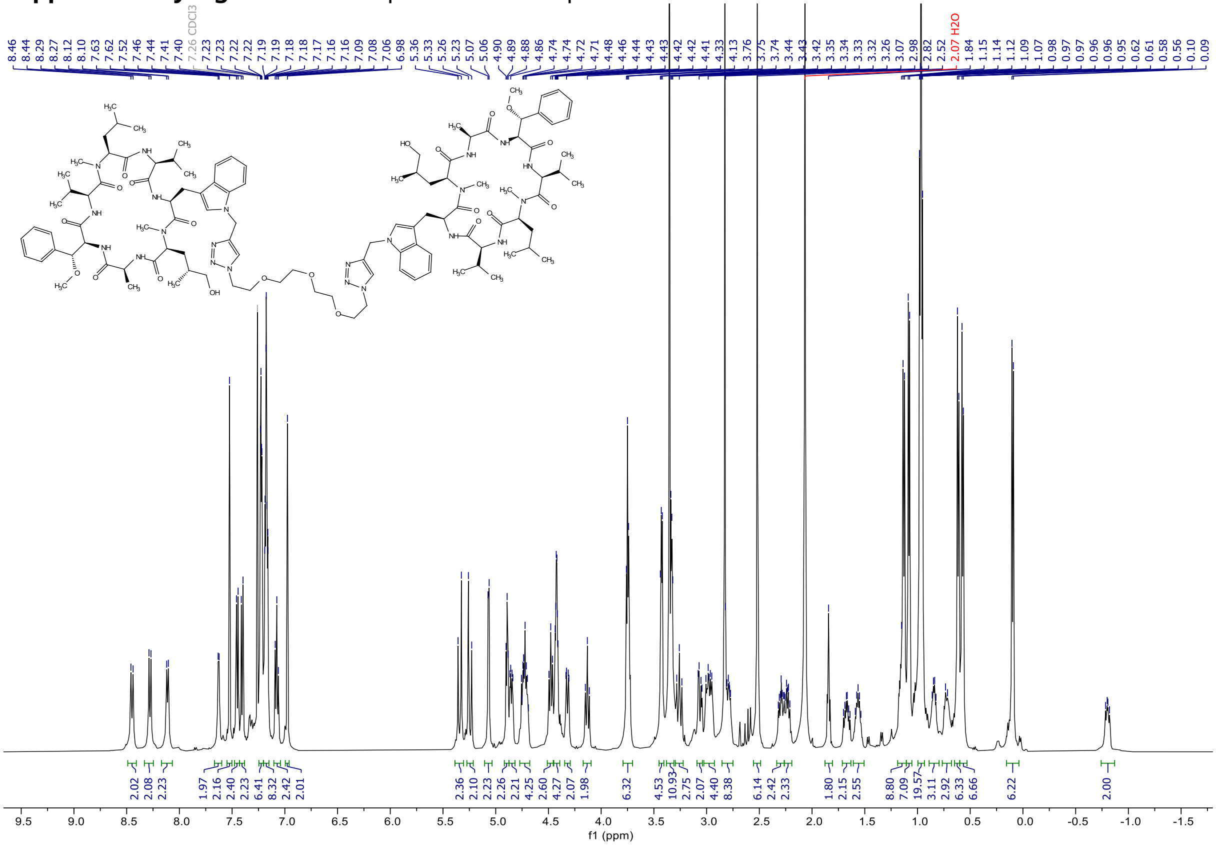

Supplementary Fig. 17. ^{13}C NMR spectrum of compound **7**

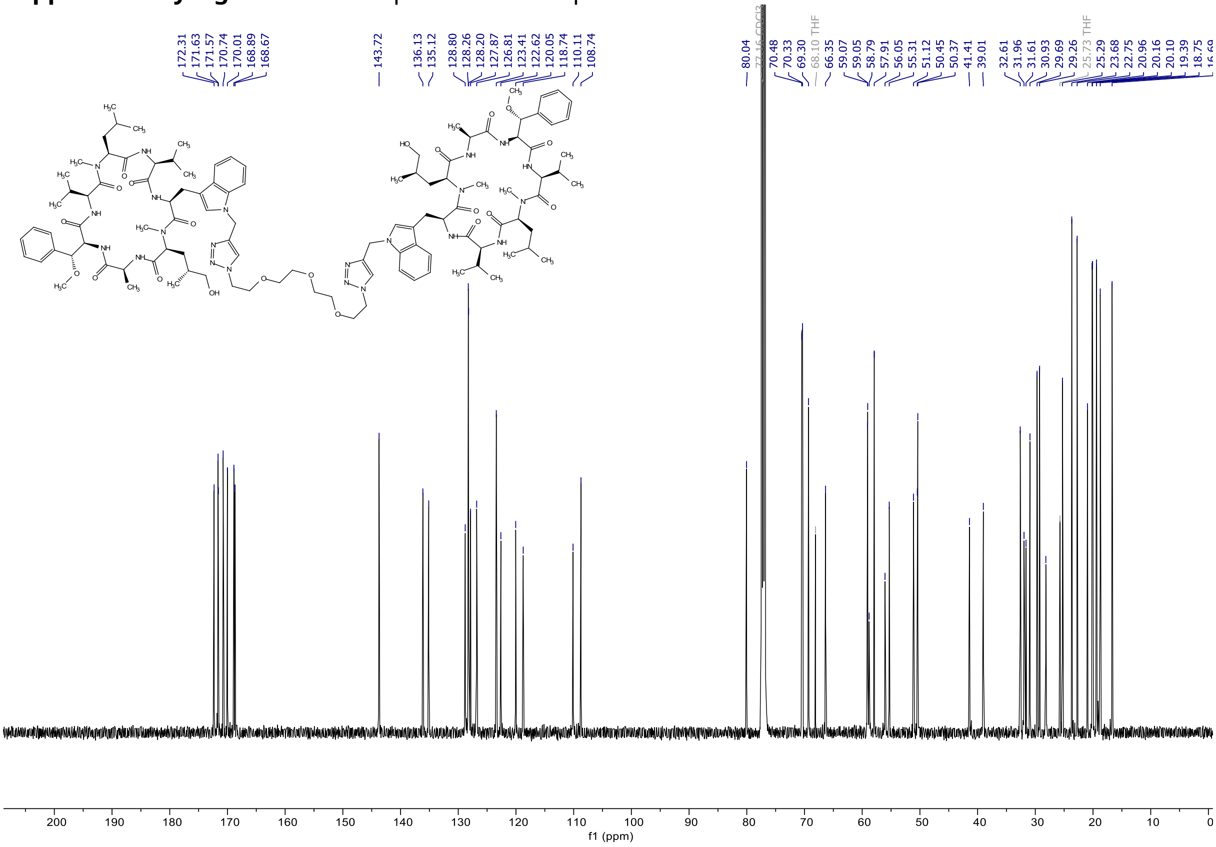

Supplementary Fig. 18. ^{1}H NMR spectrum of compound **8**

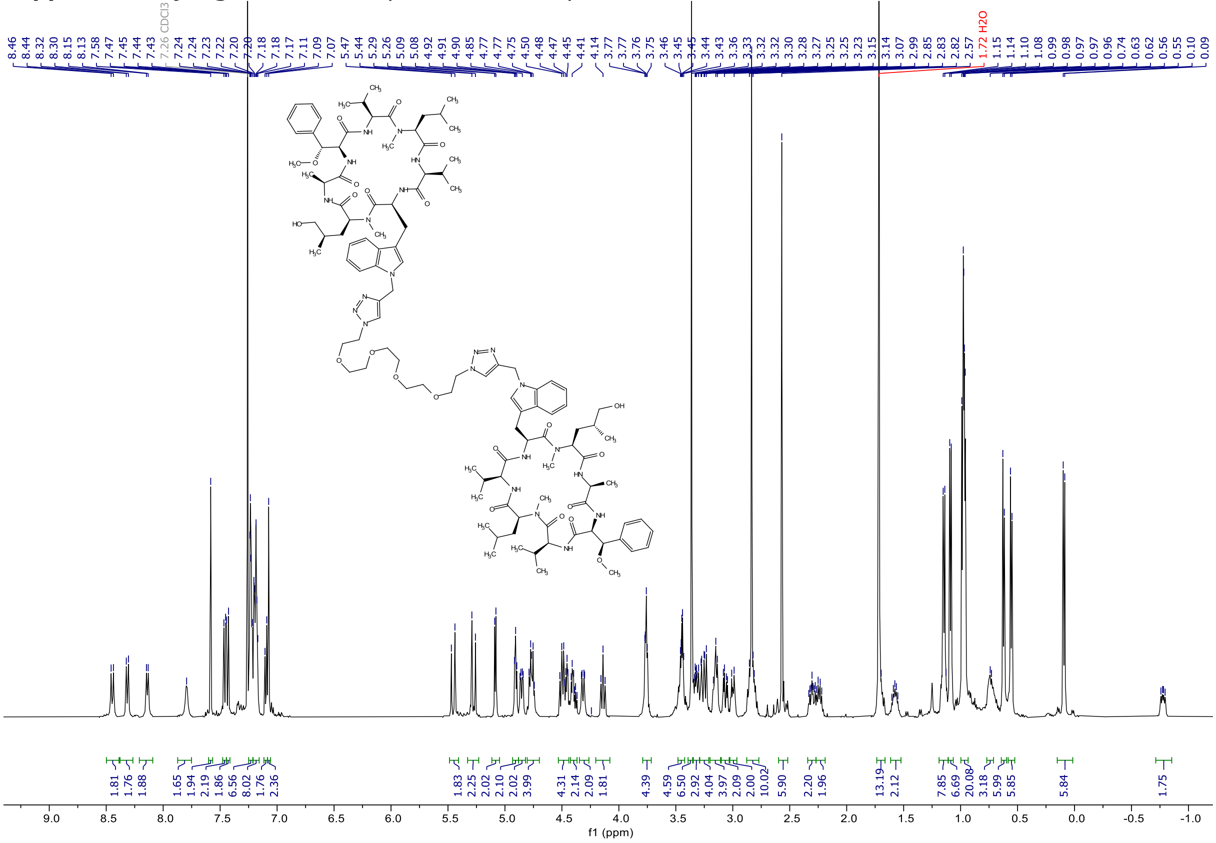

Supplementary Fig. 19. ^{13}C NMR spectrum of compound **8**

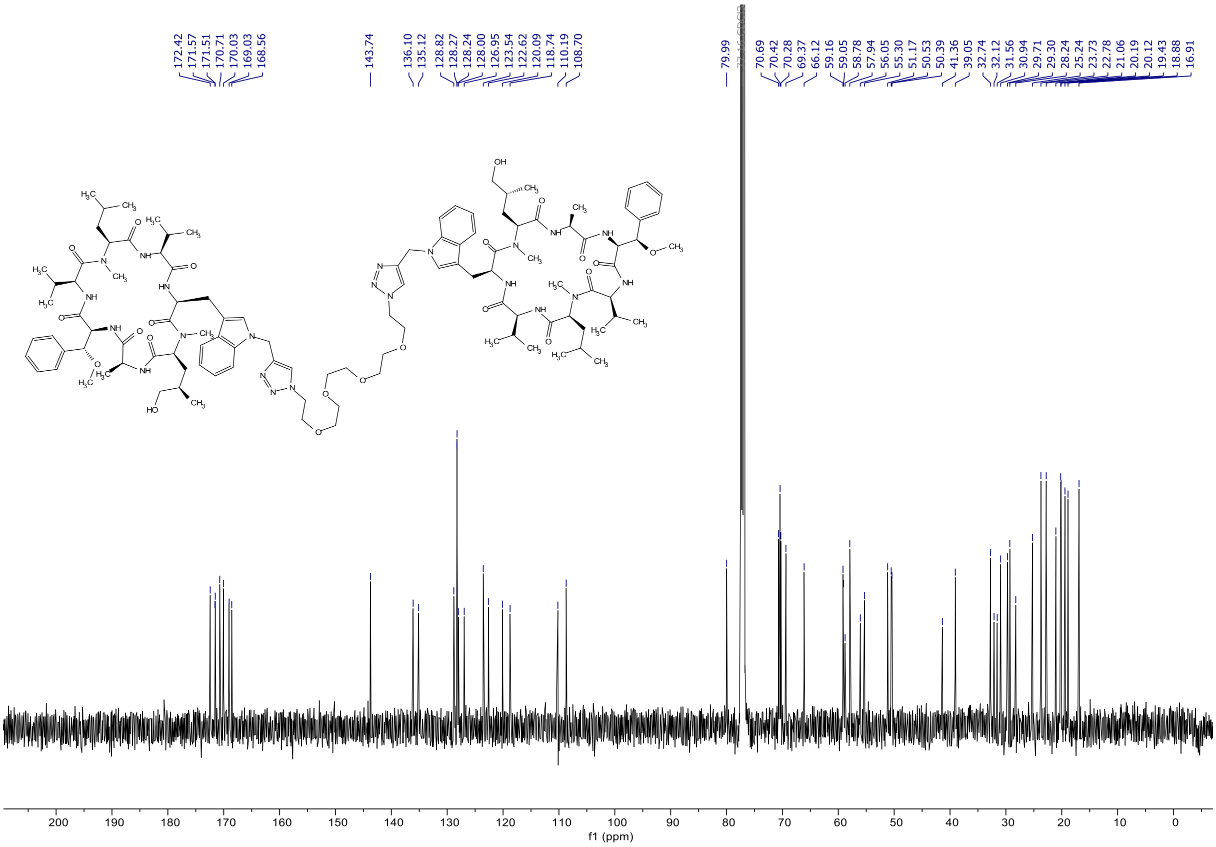

Supplementary Fig. 20. ^{1}H NMR spectrum of compound **9**

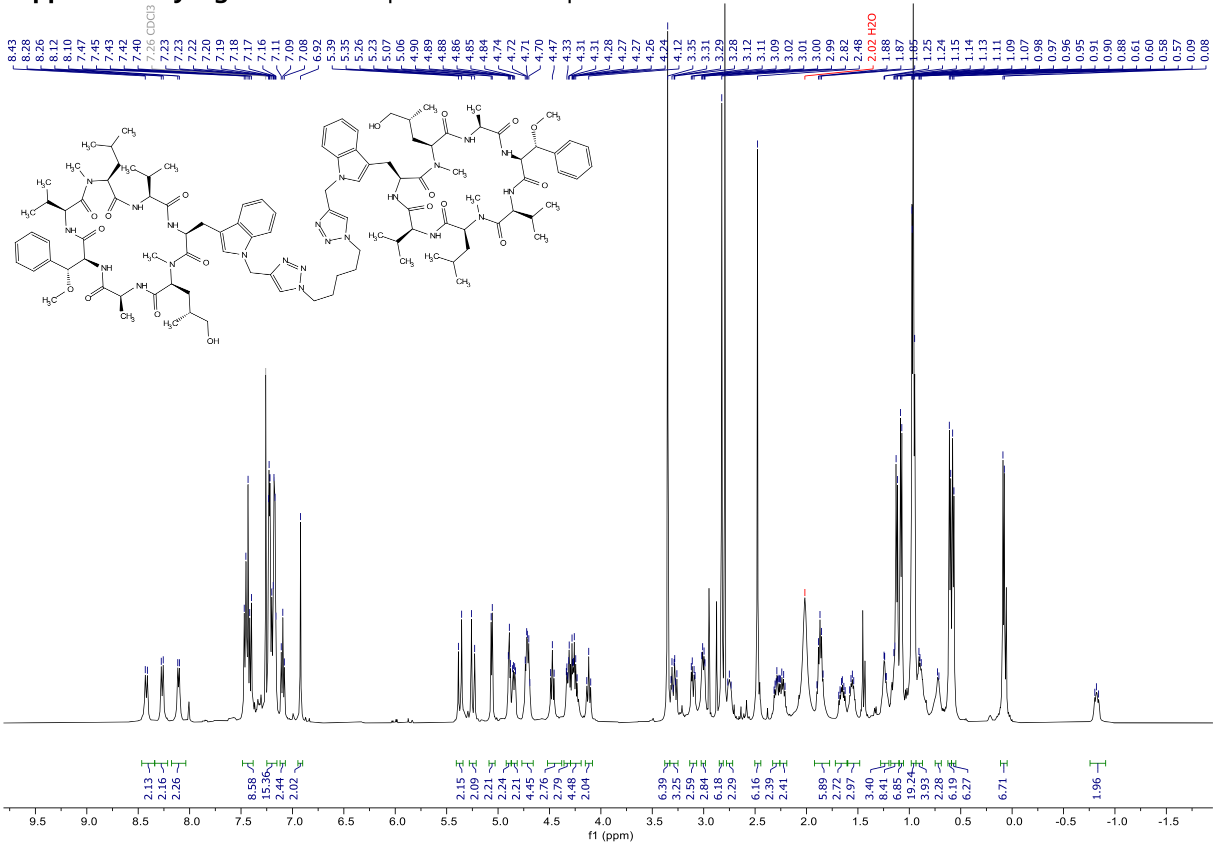

Supplementary Fig. 21. ^{13}C NMR spectrum of compound **9**

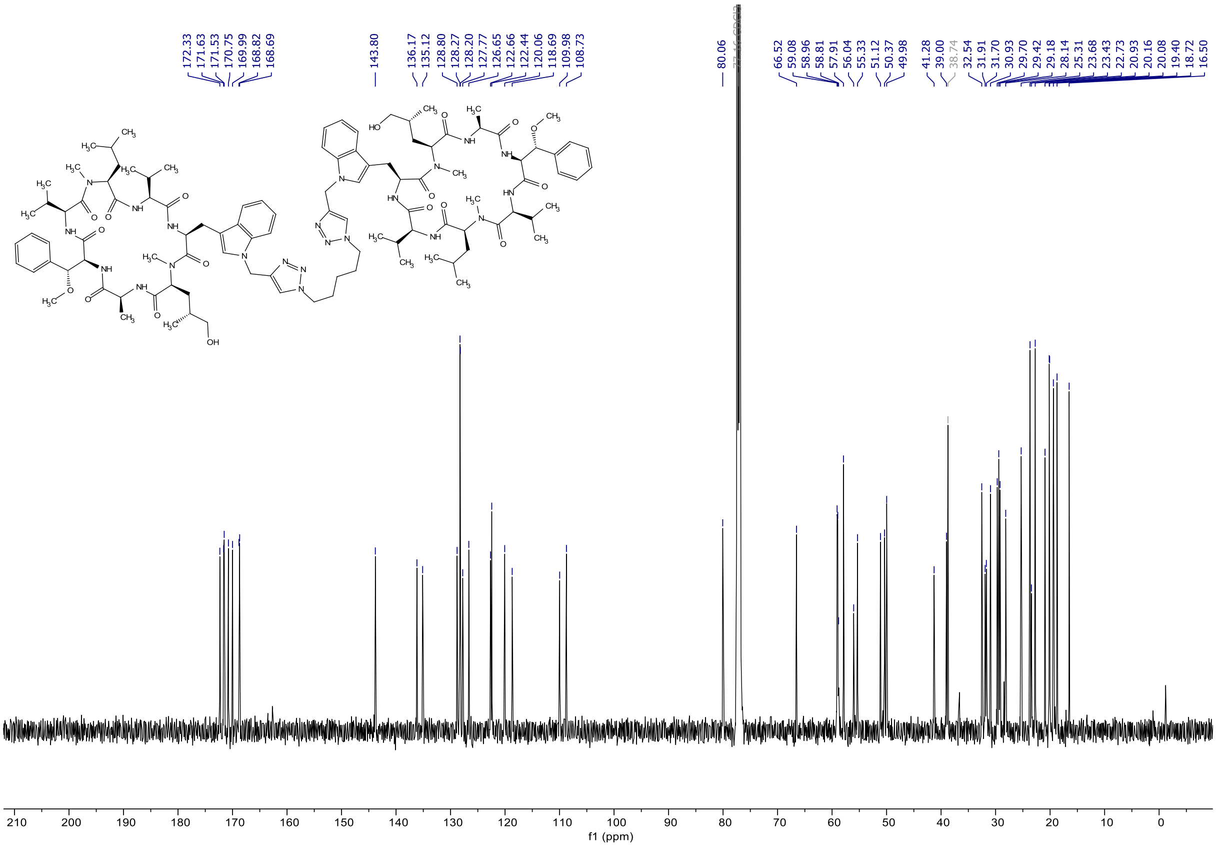

Supplementary Fig. 22. ^{1}H NMR spectrum of compound **SI-1**

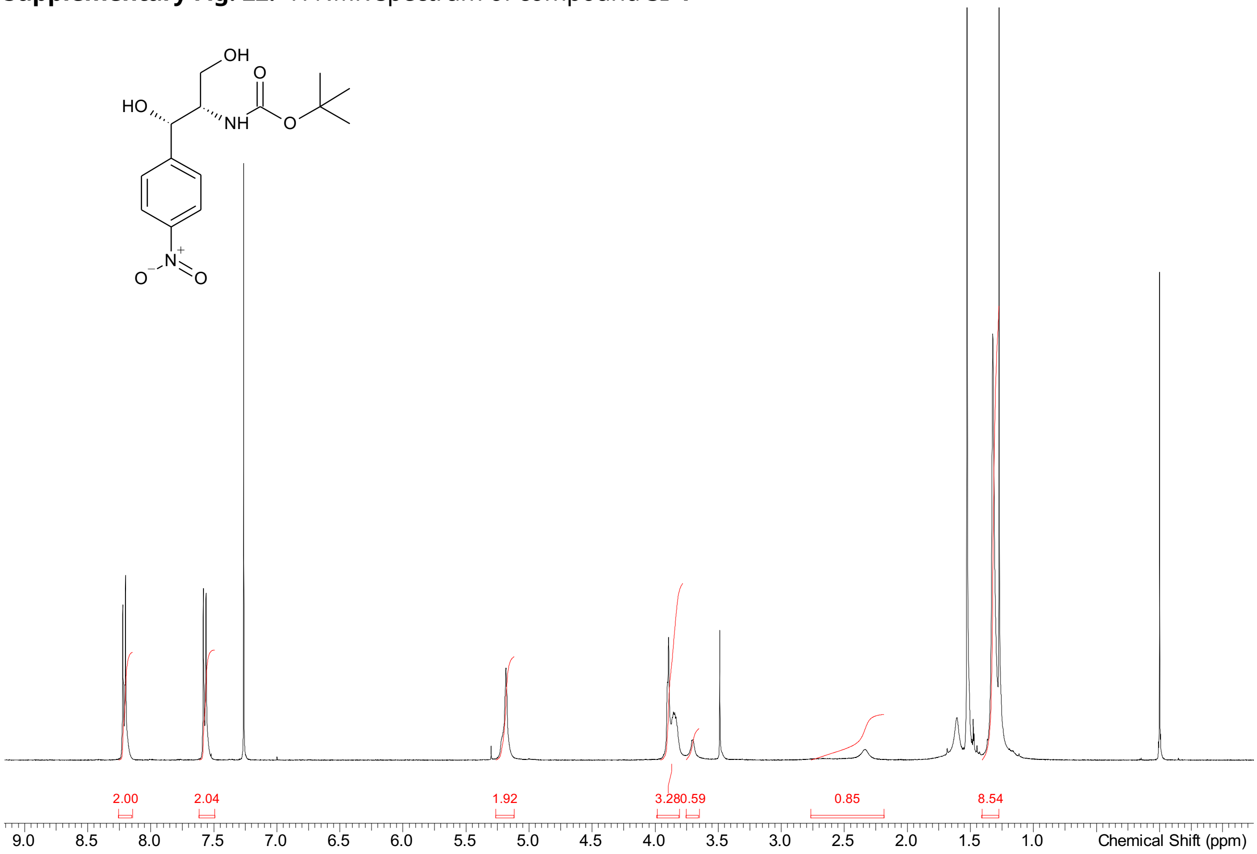

Supplementary Fig. 23. ^{1}H NMR spectrum of compound **SI-2**

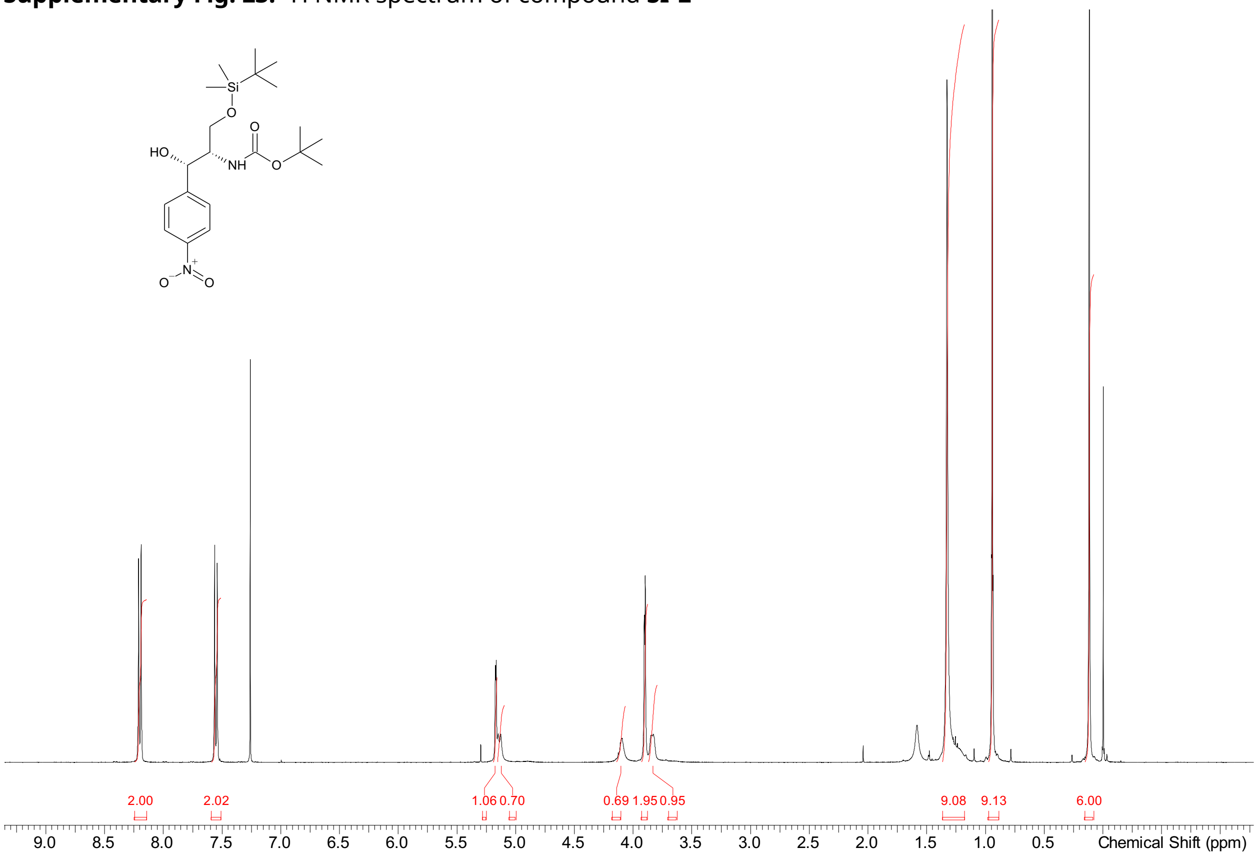

Supplementary Fig. 24. ^{1}H NMR spectrum of compound **SI-3**

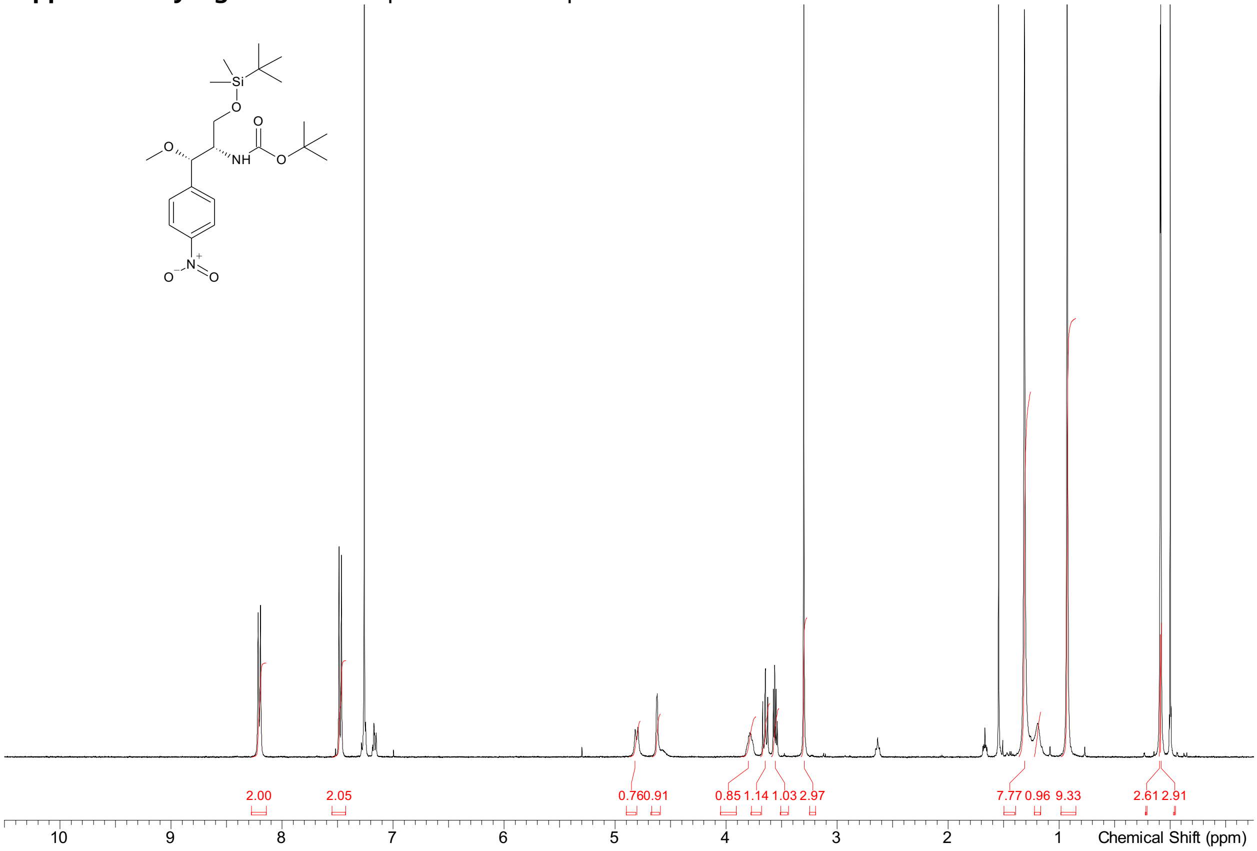

Supplementary Fig. 25. ^{1}H NMR spectrum of compound **SI-4**

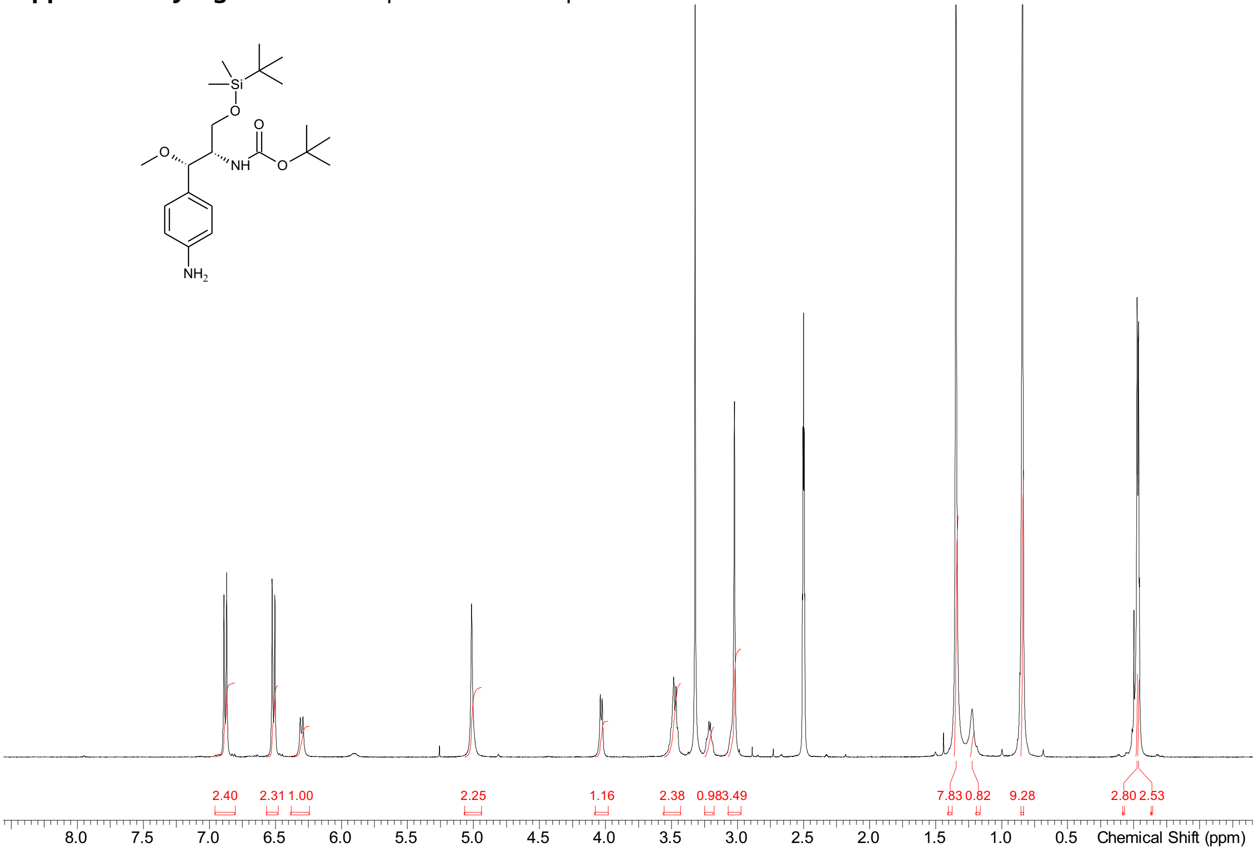

Supplementary Fig. 26. ¹H NMR spectrum of compound **SI-5**

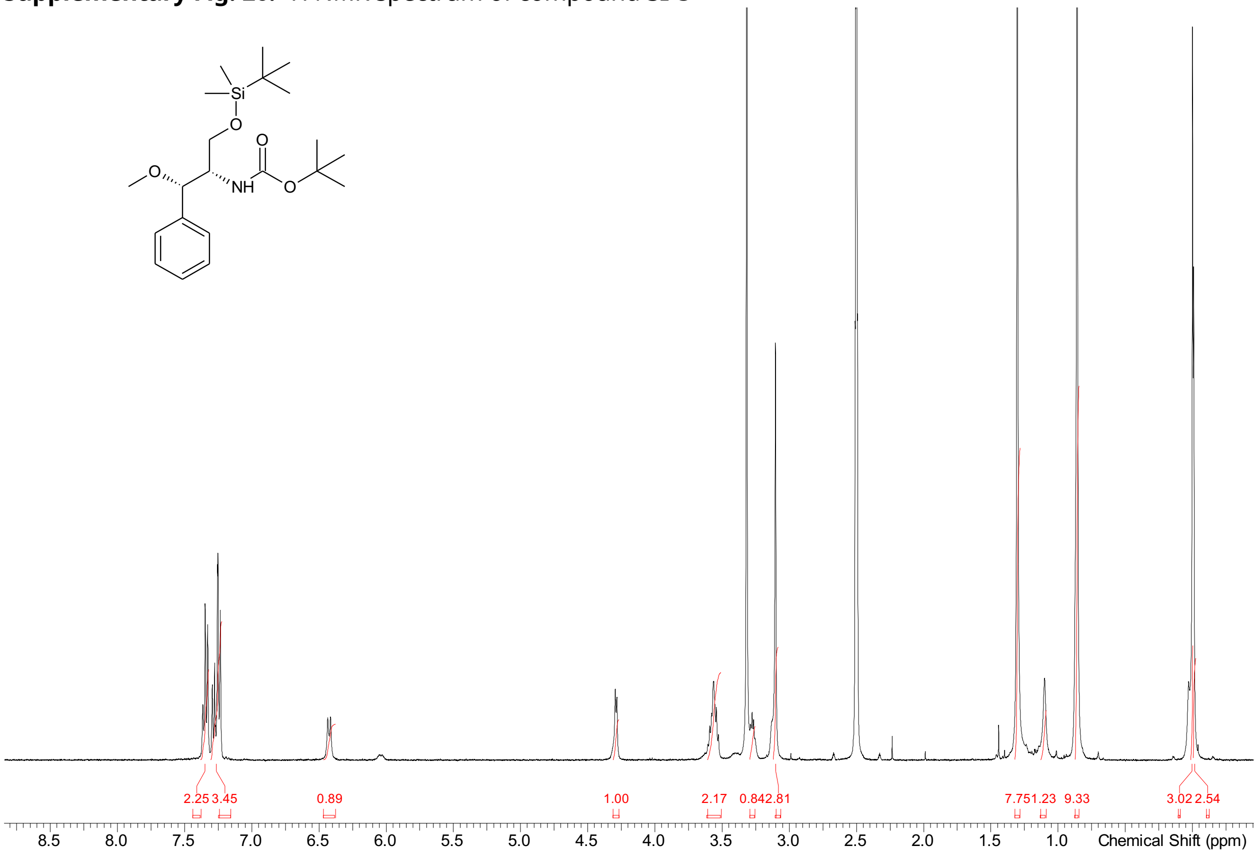

Supplementary Fig. 27. ^{1}H NMR spectrum of compound **SI-6**

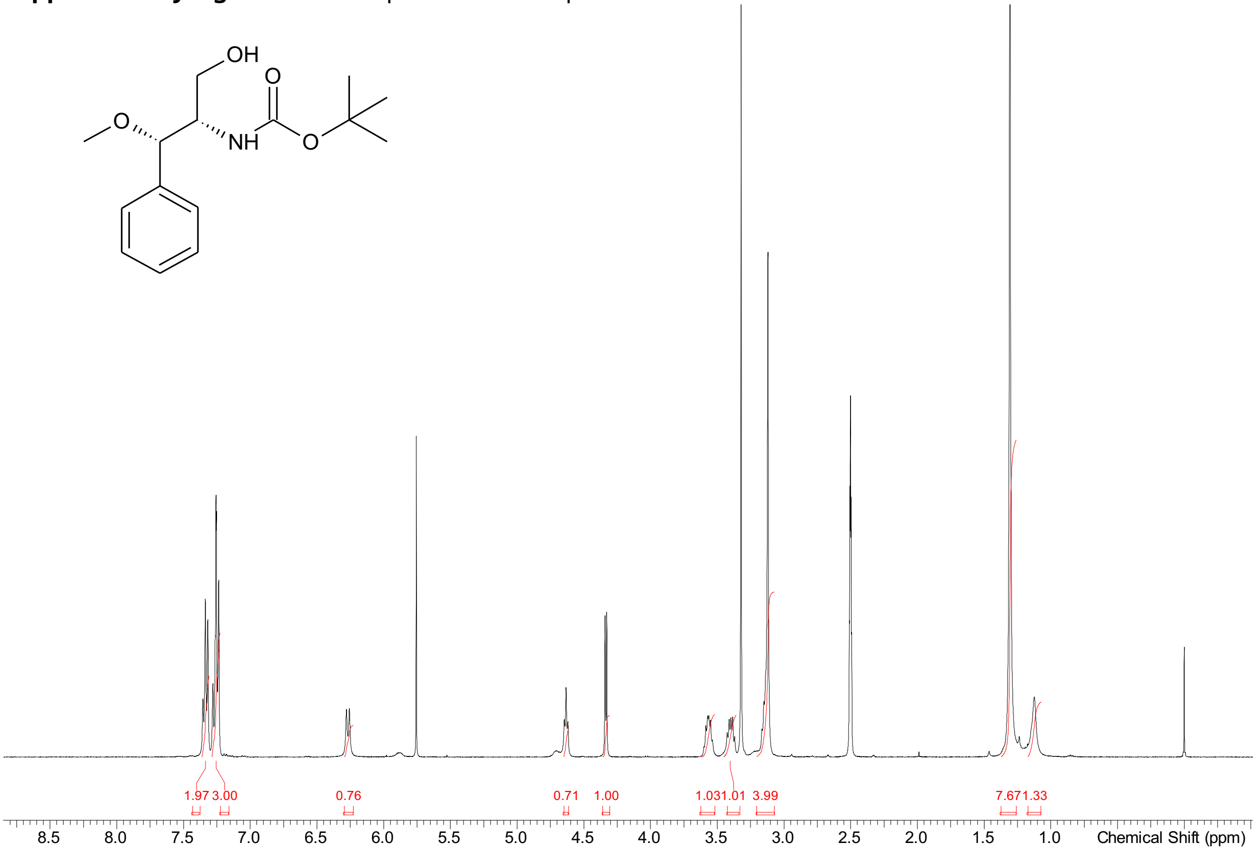

Supplementary Fig. 28. ^{1}H NMR spectrum of compound **SI-7**

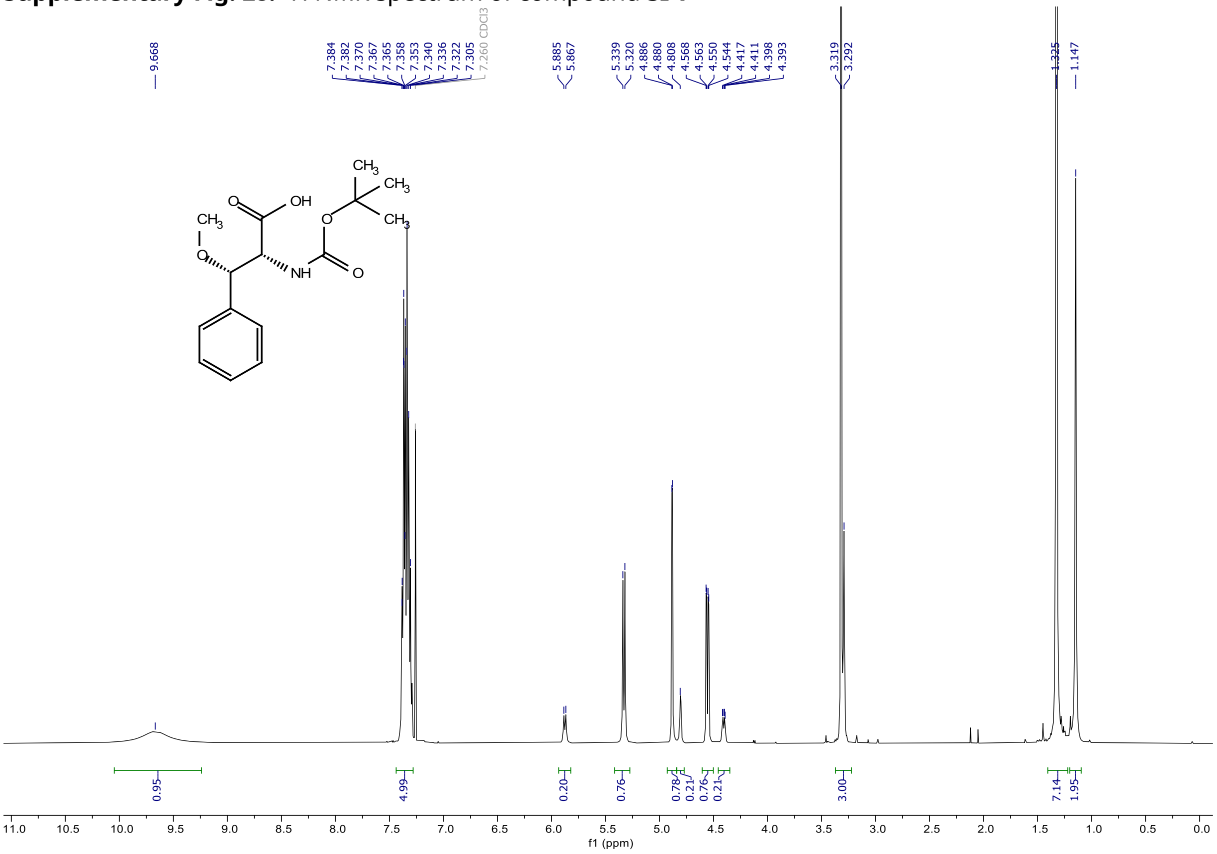

Supplementary Fig. 29. ^{13}C NMR spectrum of compound **SI-7**

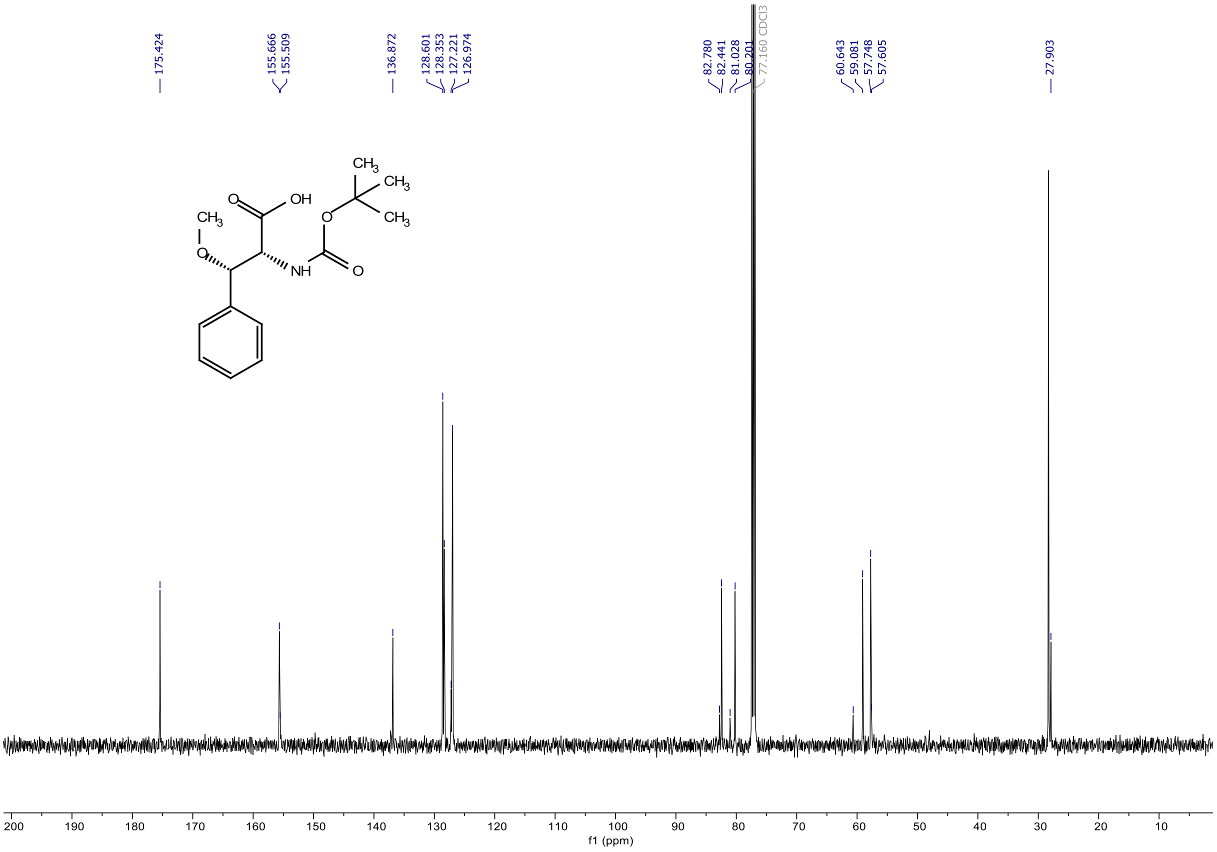

Supplementary Fig. 30. ^{1}H NMR spectrum of compound **SI-8**

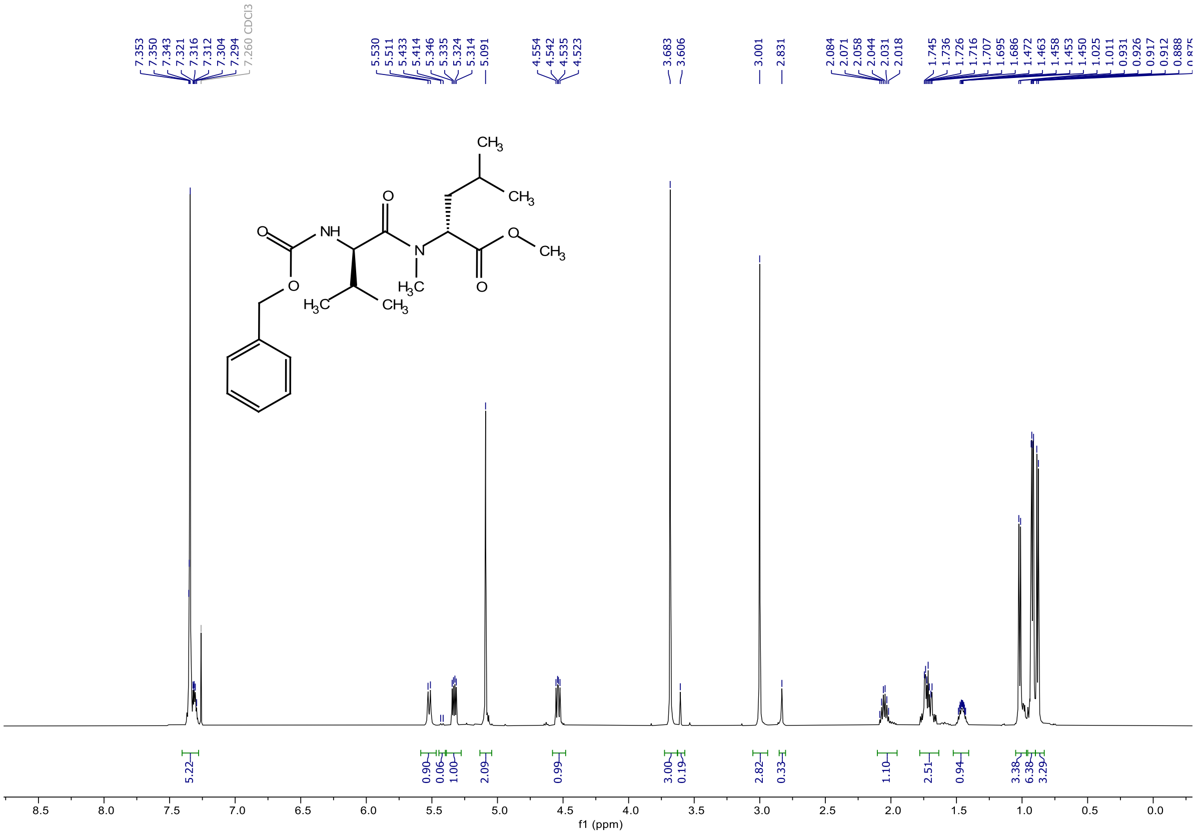

Supplementary Fig. 31. ^{13}C NMR spectrum of compound **SI-8**

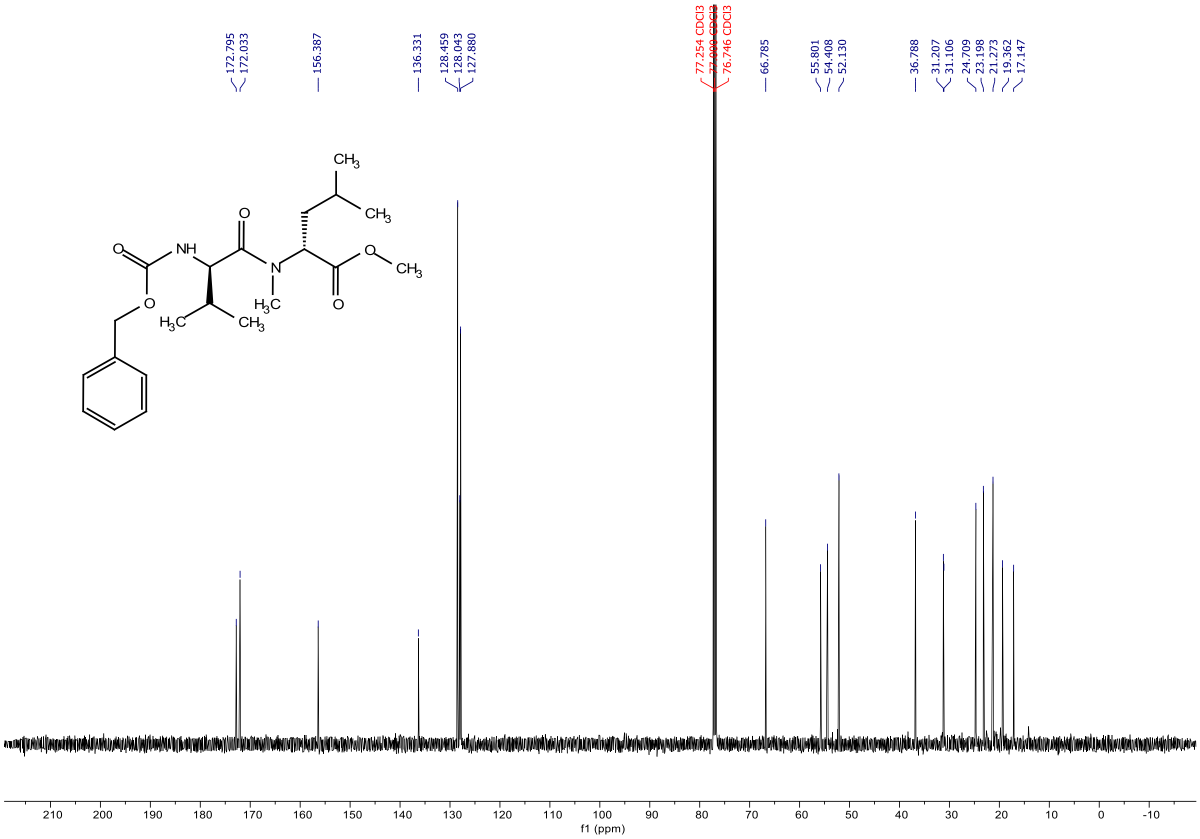

Supplementary Fig. 32. ^{1}H NMR spectrum of compound **SI-9**

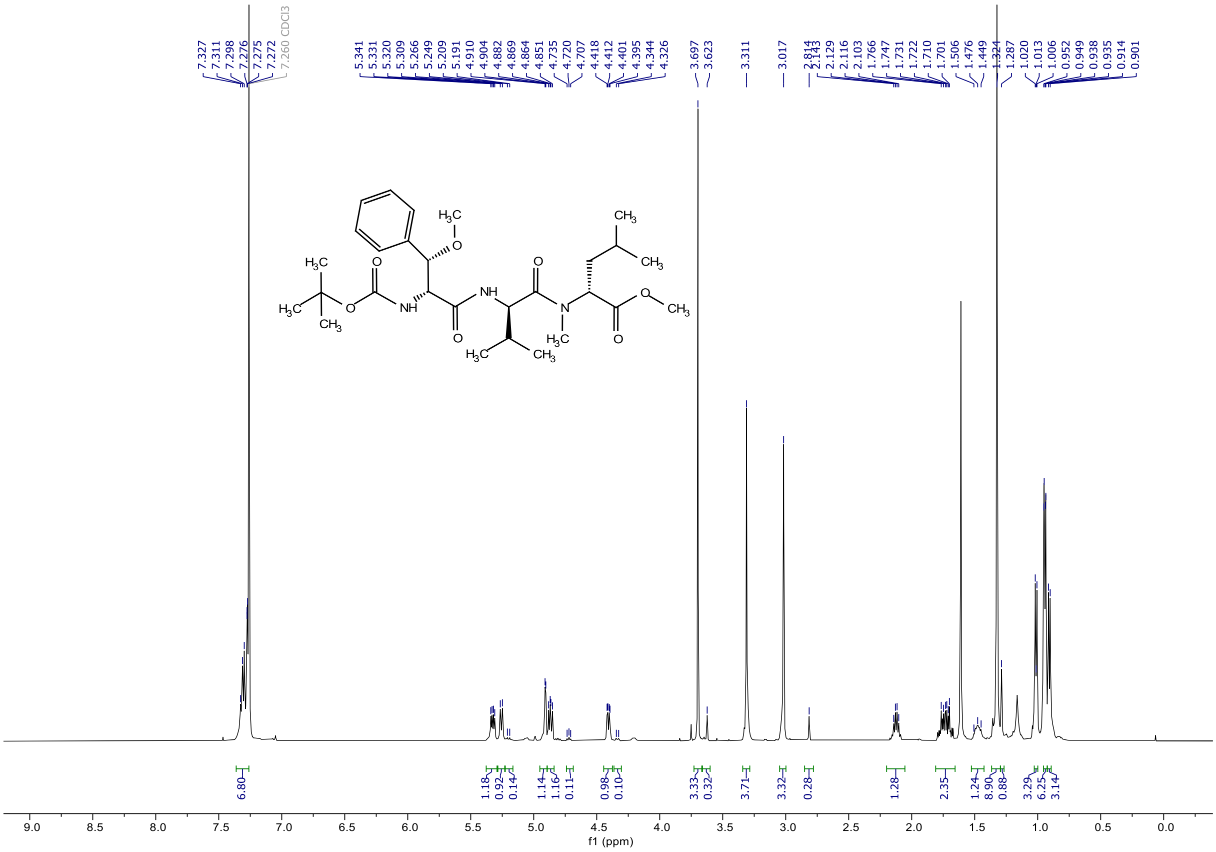

Supplementary Fig. 33. ^{13}C NMR spectrum of compound **SI-9**

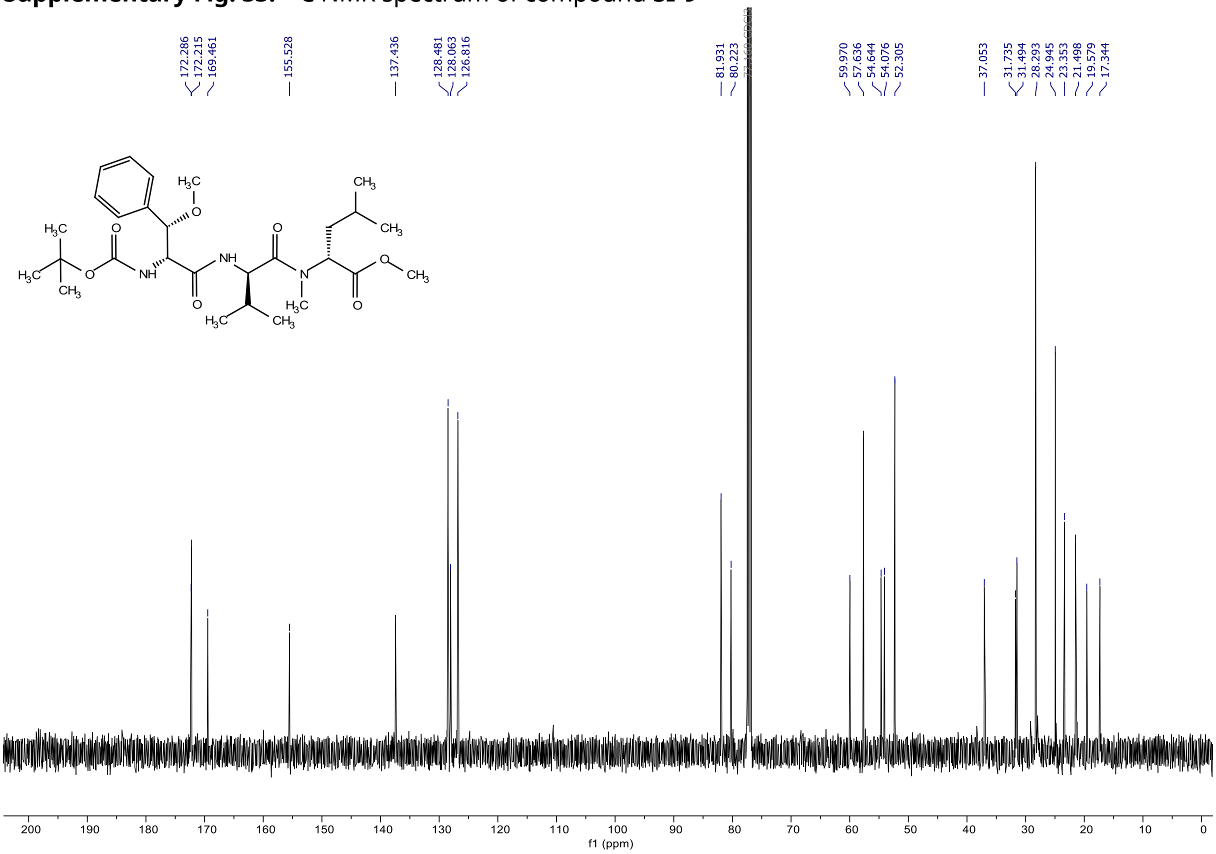

Supplementary Fig. 34. ^{1}H NMR spectrum of compound **SI-10**

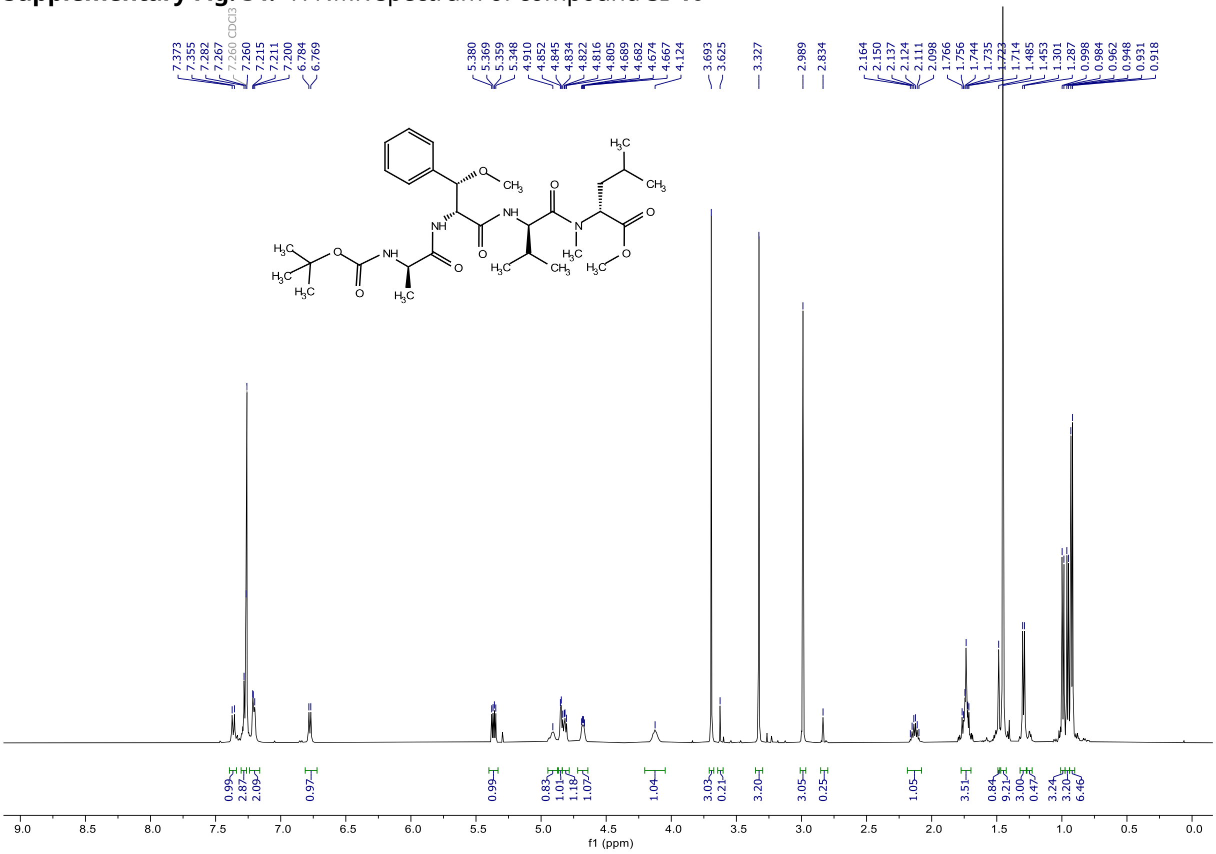

Supplementary Fig. 35. ^{13}C NMR spectrum of compound **SI-10**

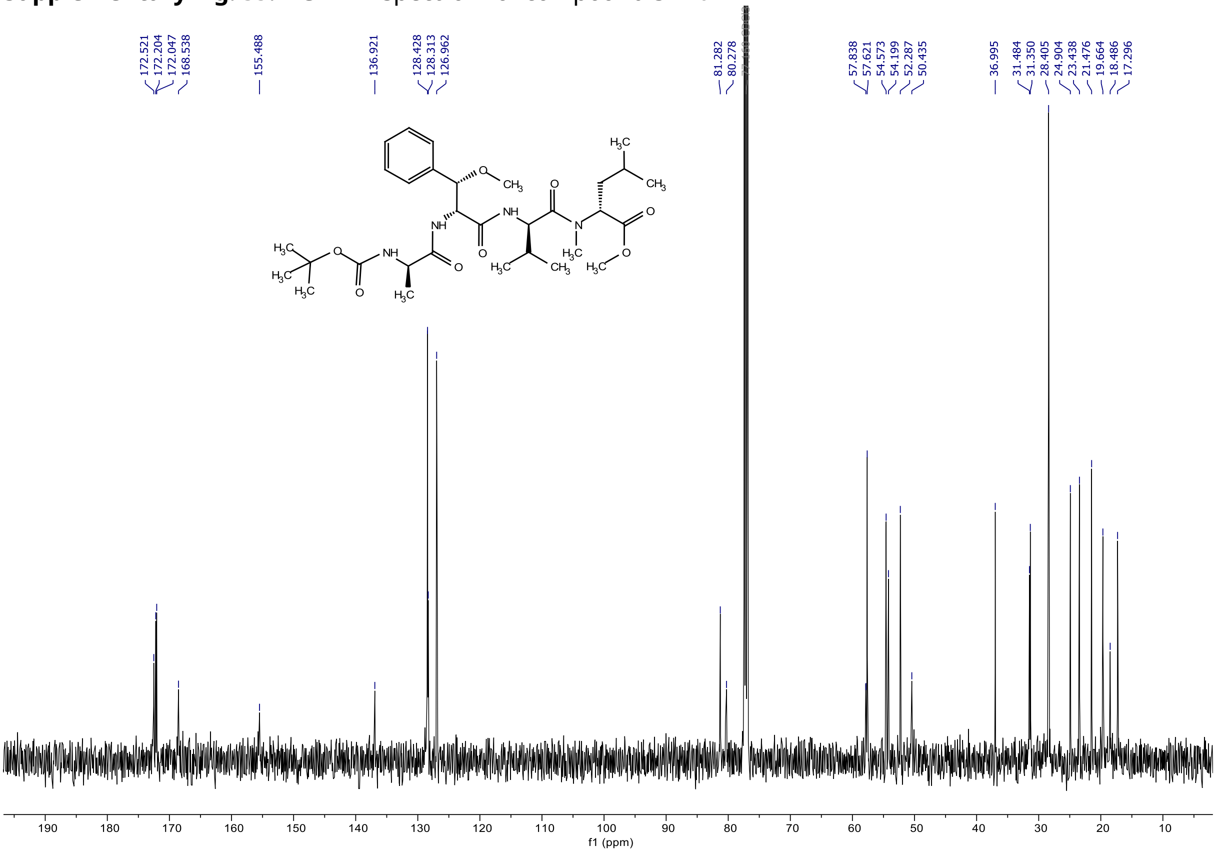

Supplementary Fig. 36. ^{1}H NMR spectrum of compound **SI-11**

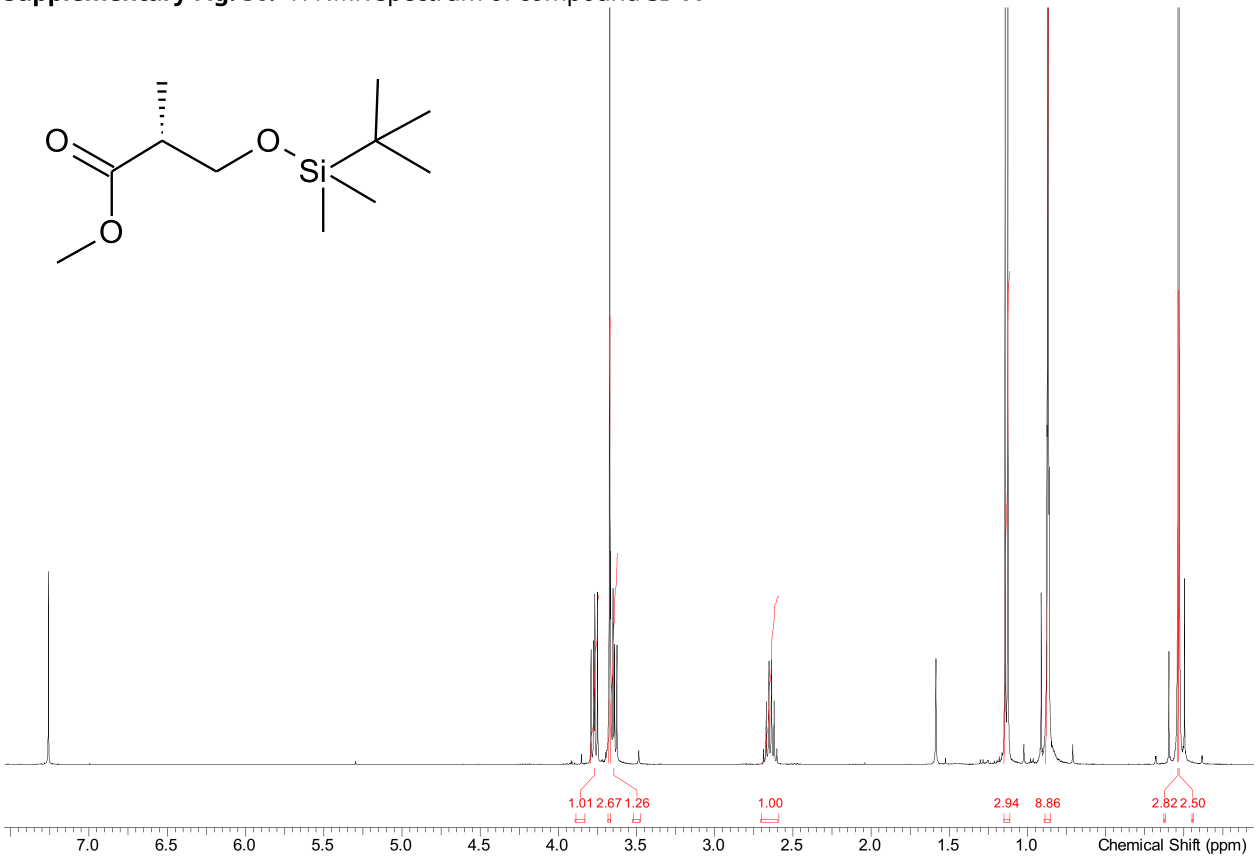

Supplementary Fig. 37. ^{1}H NMR spectrum of compound **SI-12**

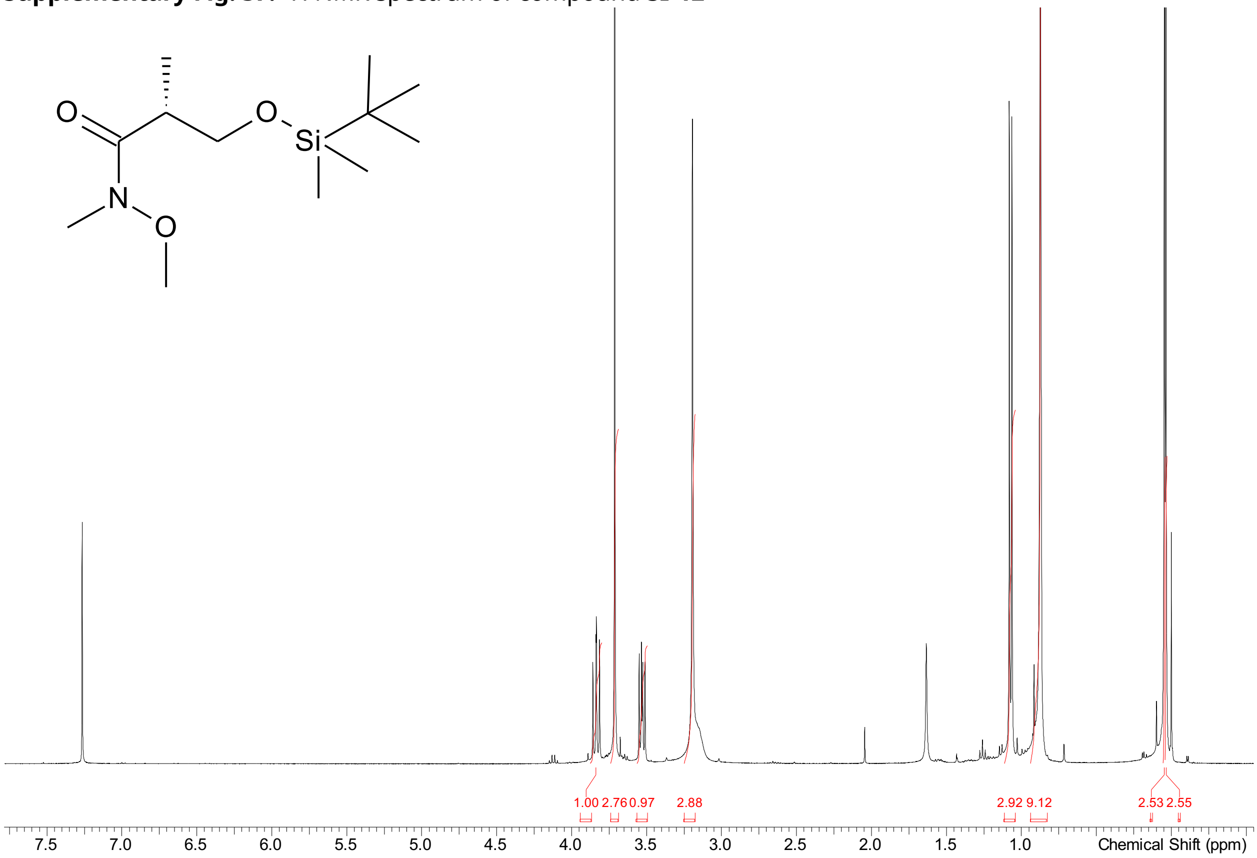

Supplementary Fig. 38. ^{1}H NMR spectrum of compound **SI-13**

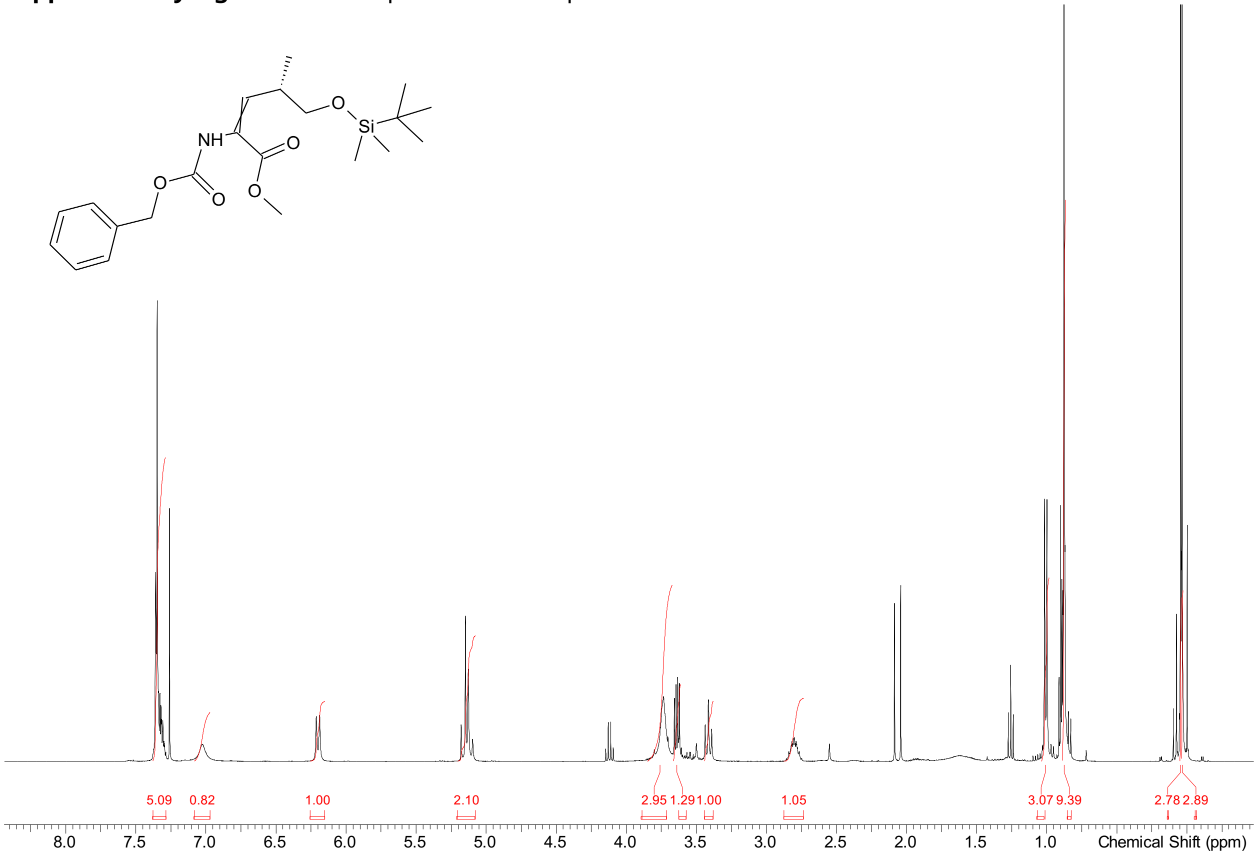

Supplementary Fig. 39. ^{1}H NMR spectrum of compound **SI-14**

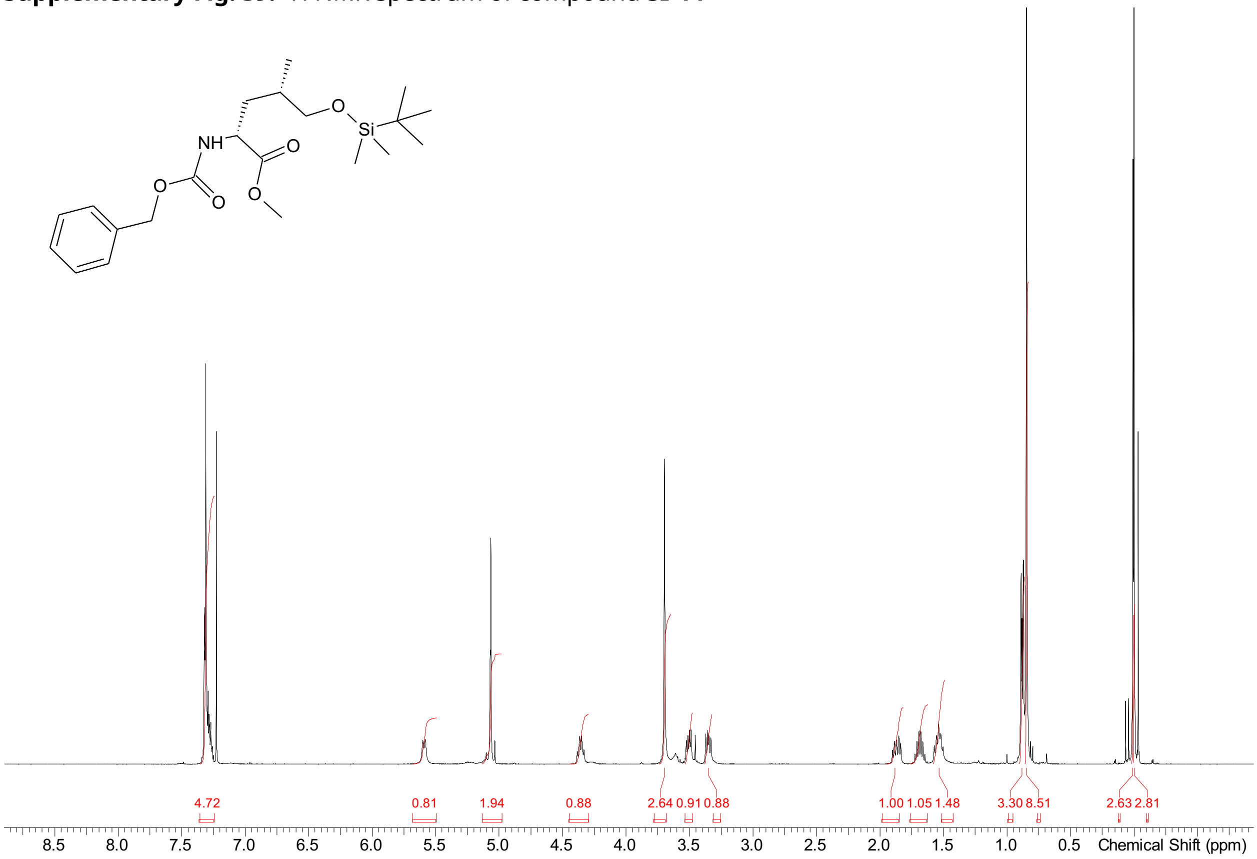

Supplementary Fig. 40. ^{1}H NMR spectrum of compound **SI-15**

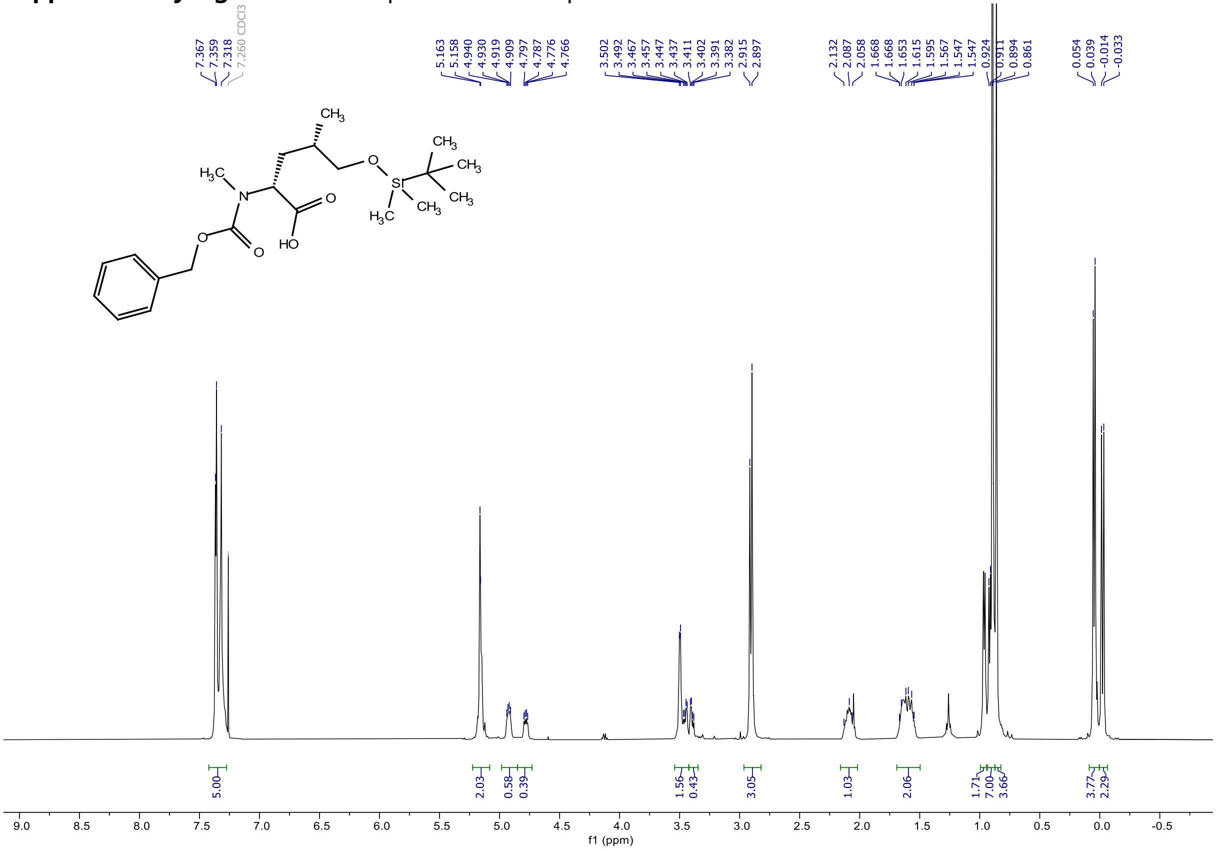

Supplementary Fig. 41. ^{13}C NMR spectrum of compound **SI-15**

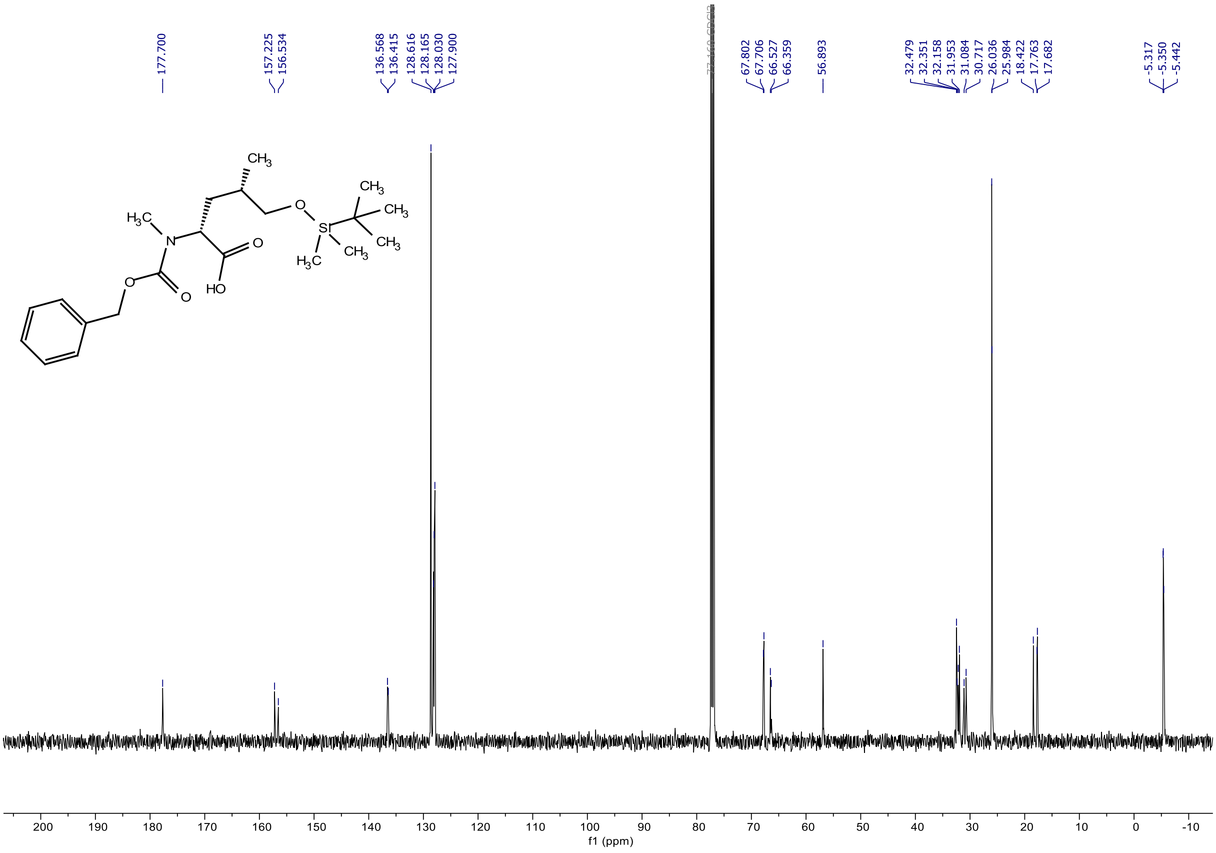

Supplementary Fig. 42. ^{1}H NMR spectrum of compound **2a**

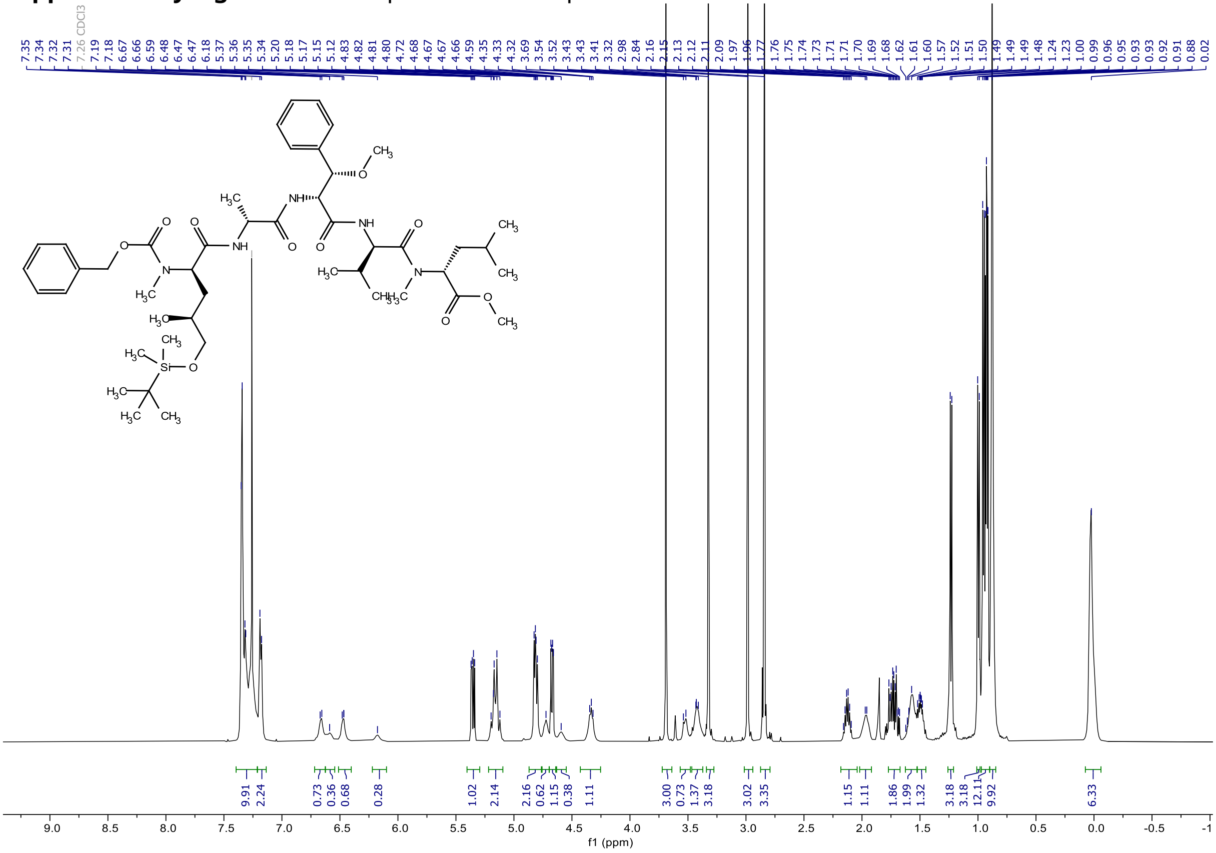

Supplementary Fig. 43. ^{13}C NMR spectrum of compound **2a**

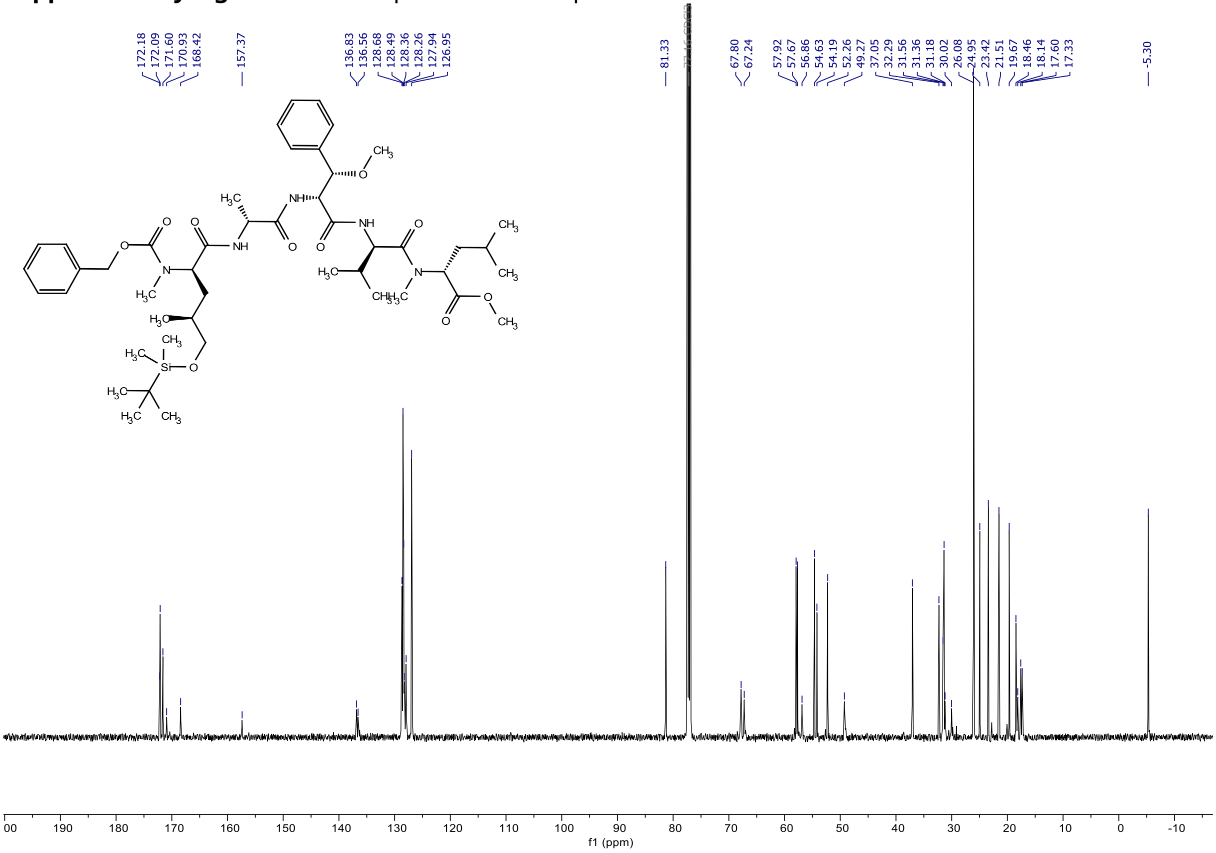

Supplementary Fig. 44. ^{1}H NMR spectrum of compound **SI-16**

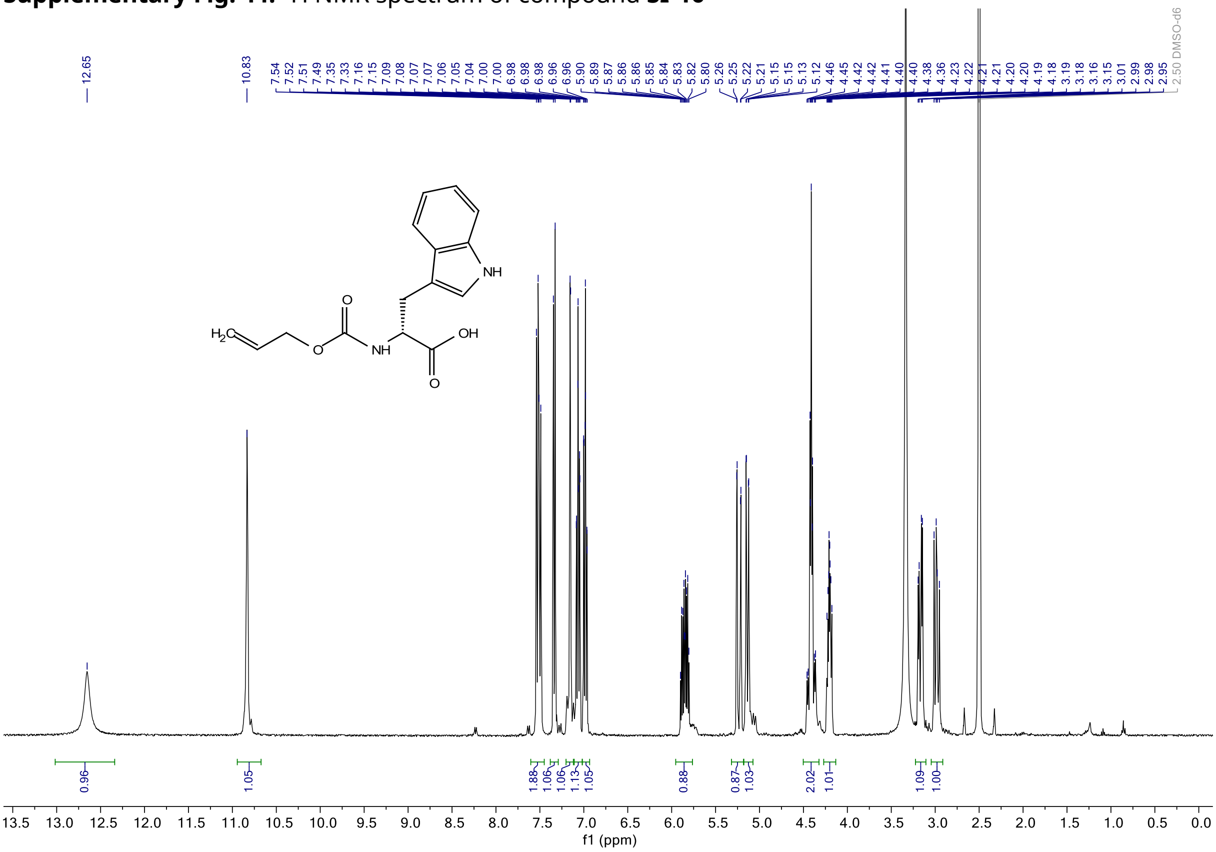

Supplementary Fig. 45. ^{13}C NMR spectrum of compound **SI-16**

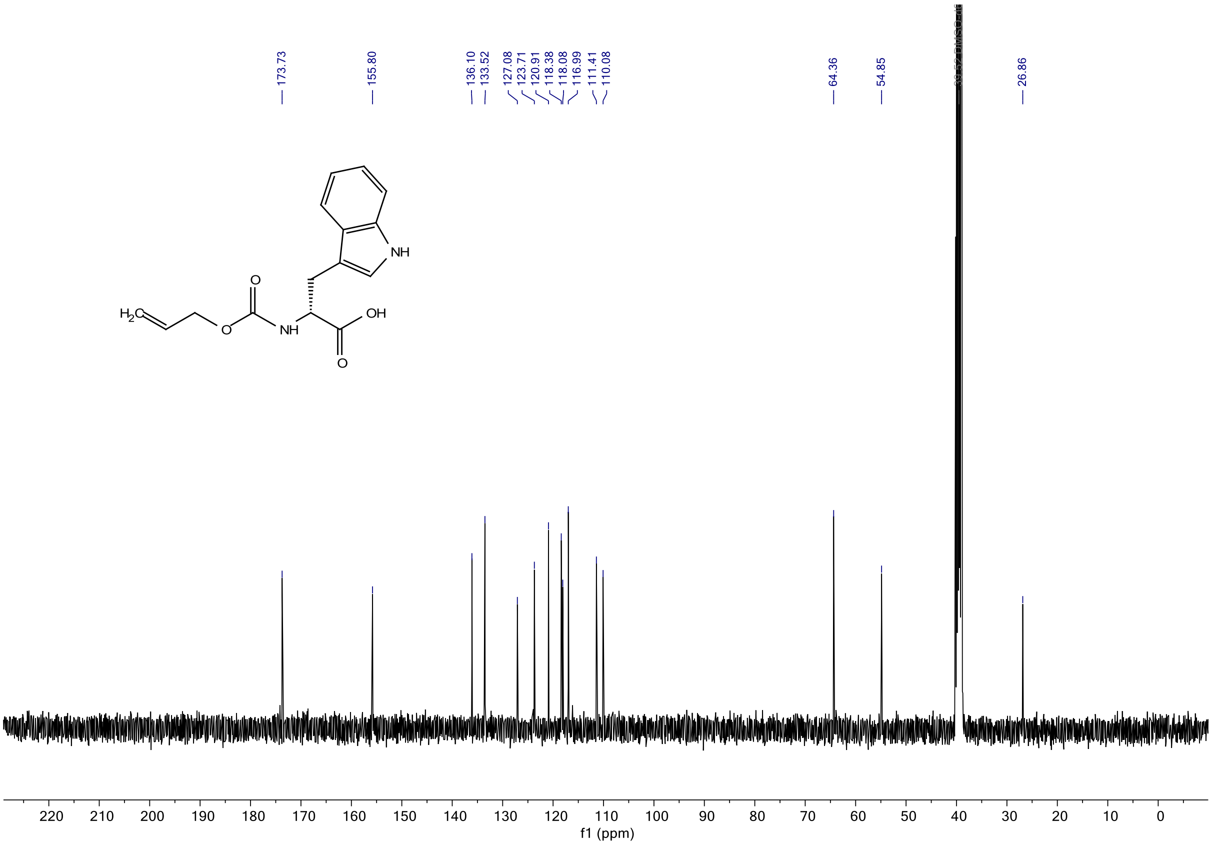

Supplementary Fig. 46. ^{1}H NMR spectrum of compound **SI-17**

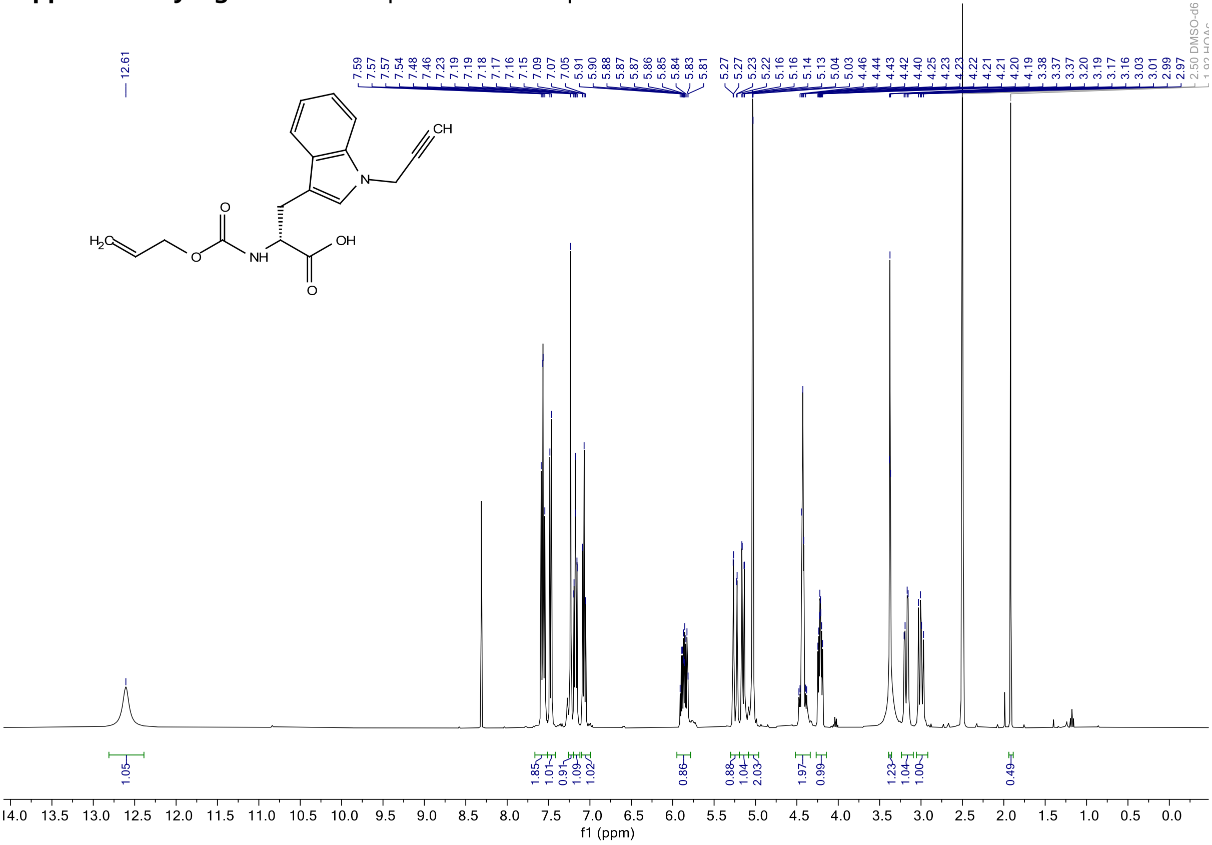

Supplementary Fig. 47. ^{13}C NMR spectrum of compound **SI-17**

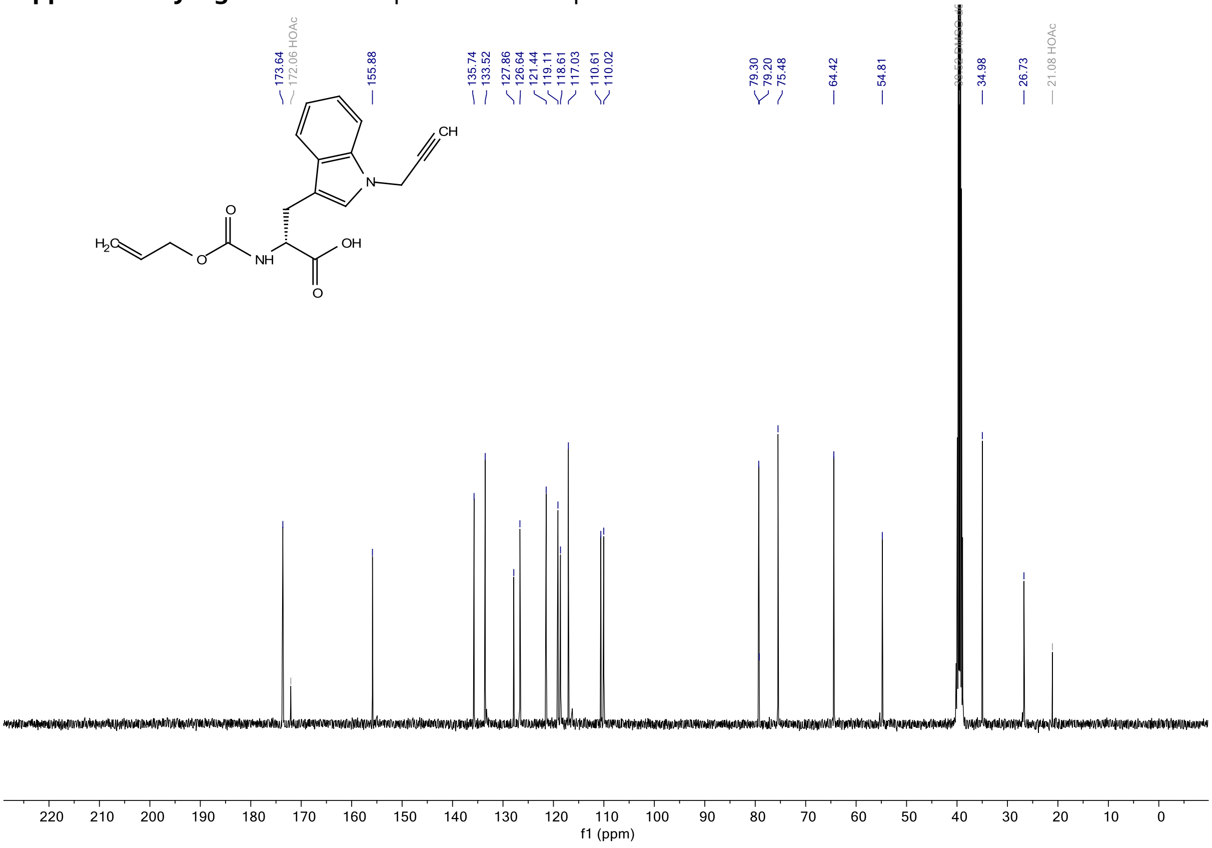

Supplementary Fig. 48. ^{1}H NMR spectrum of compound **3a**

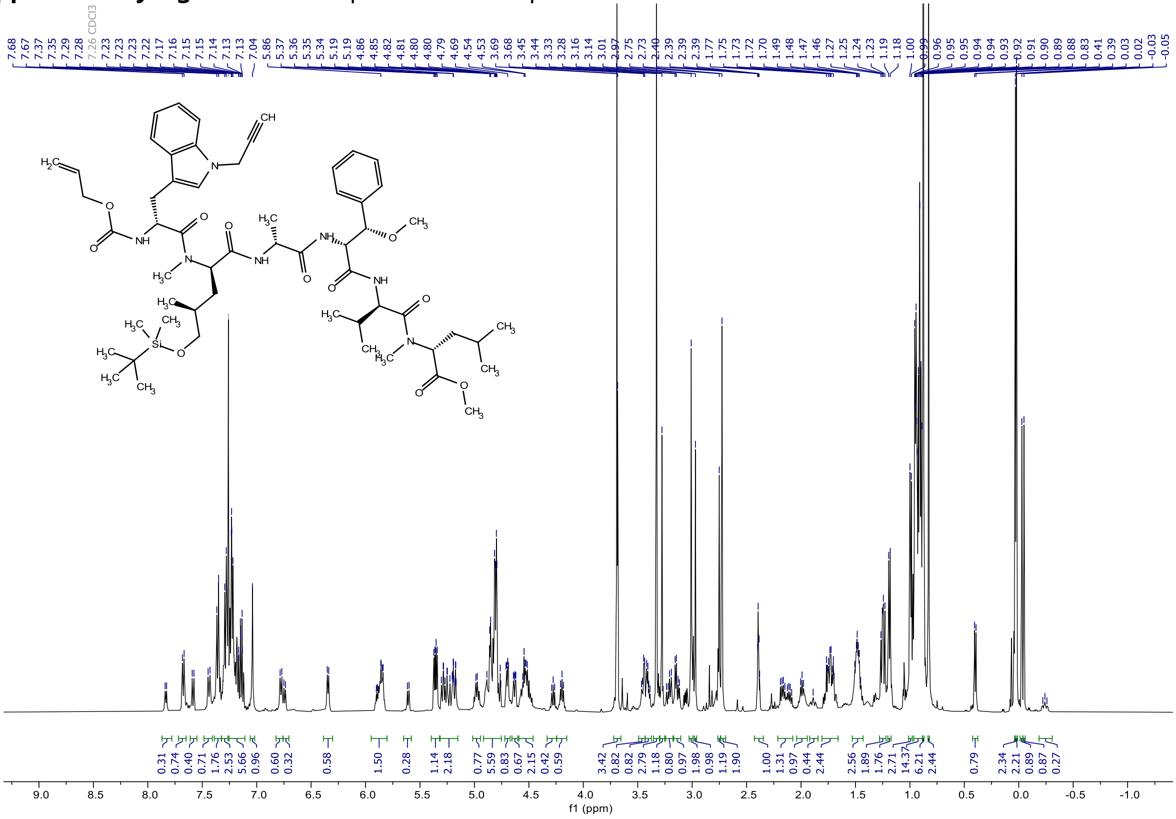

Supplementary Fig. 49. ^{13}C NMR spectrum of compound **3a**

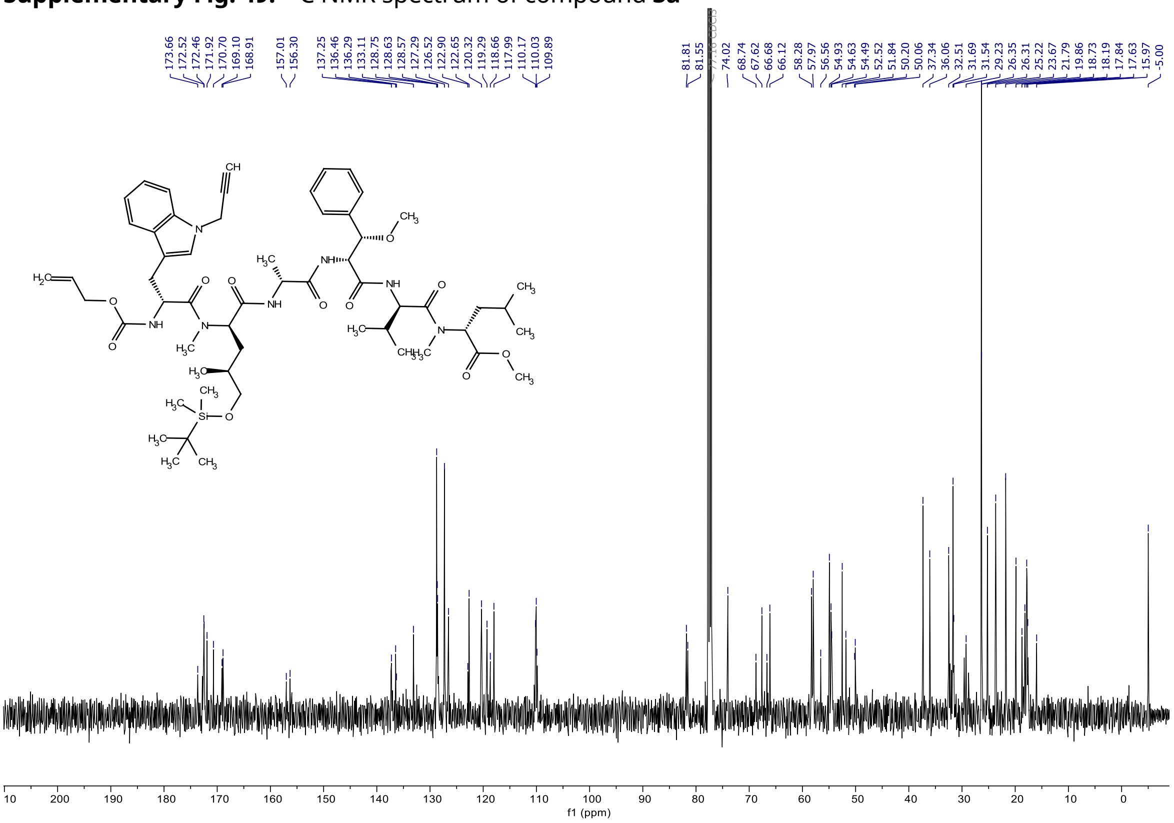

Supplementary Fig. 50. ^{1}H NMR spectrum of compound **4a**

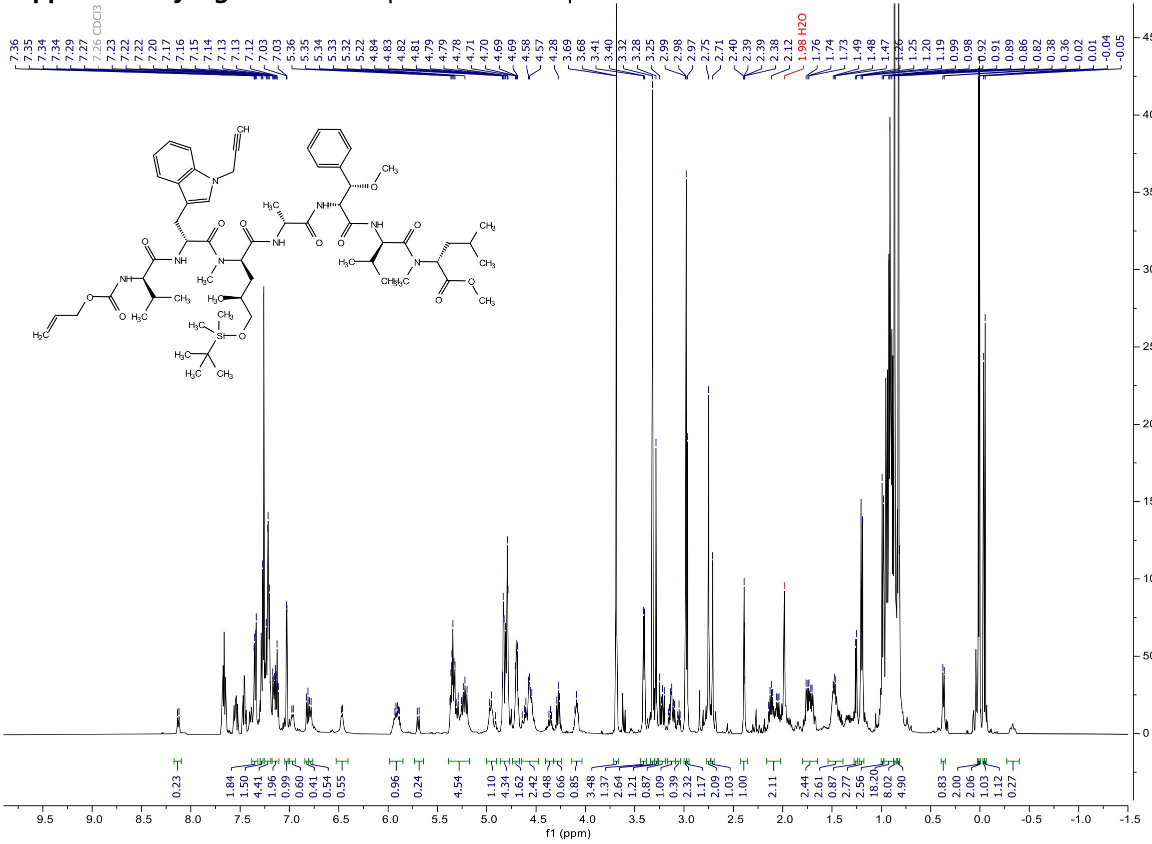

Supplementary Fig. 51. ^{13}C NMR spectrum of compound **4a**

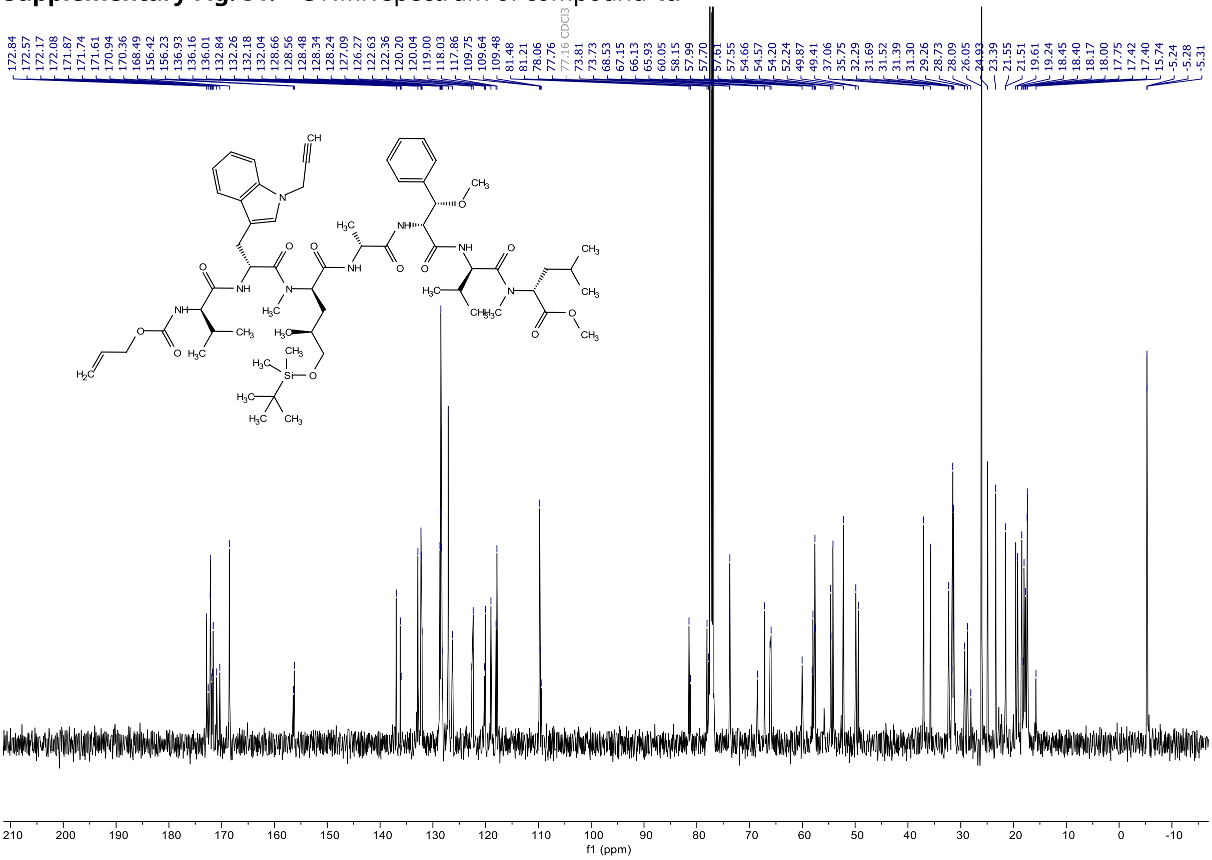

Supplementary Fig. 52. ^{1}H NMR spectrum of compound **5a**

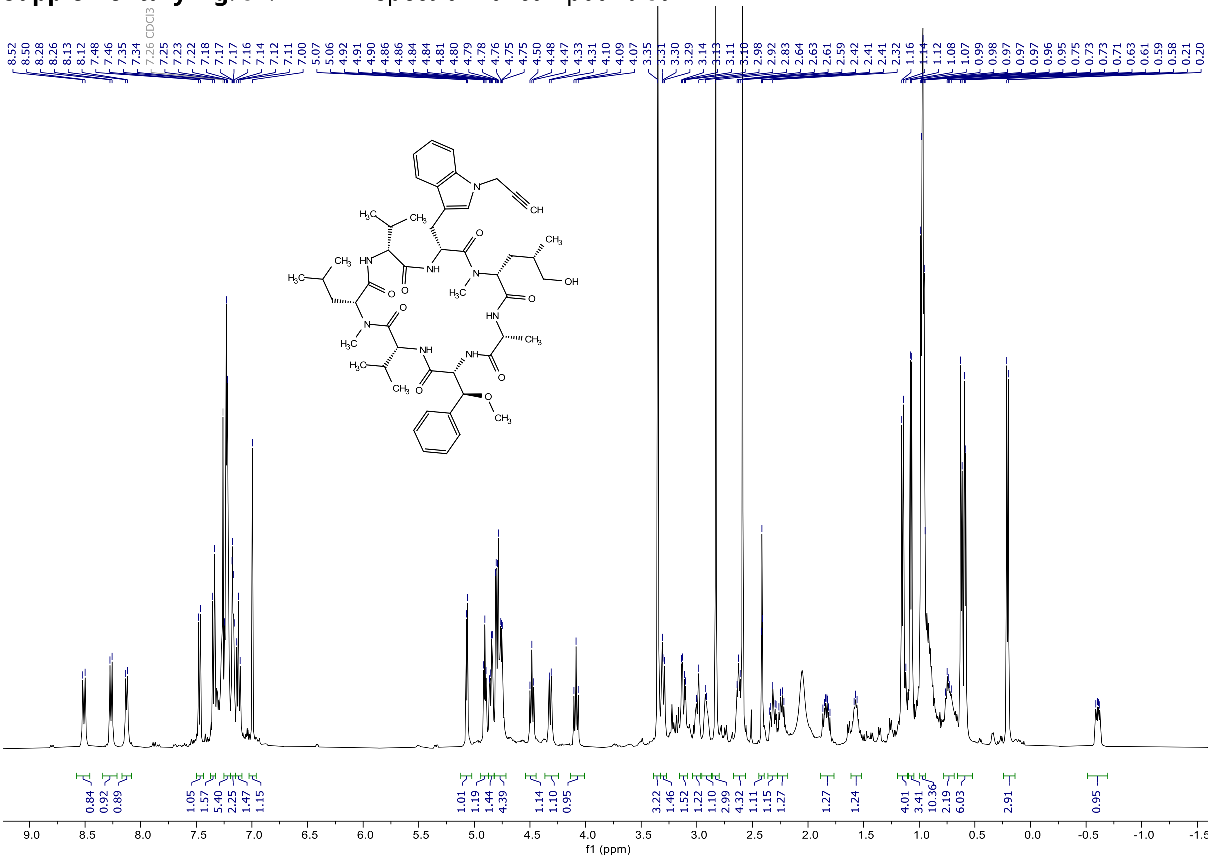

Supplementary Fig. 53. ^{13}C NMR spectrum of compound **5a**

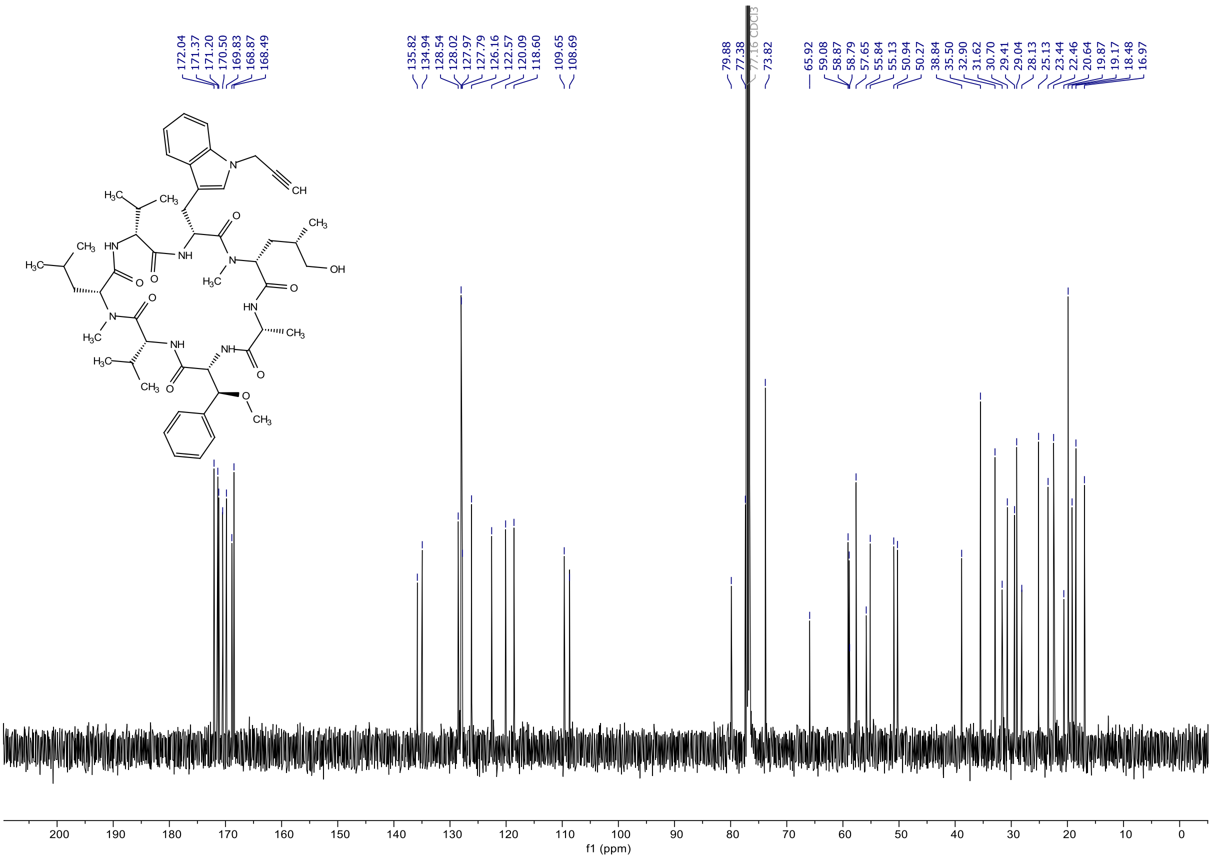

Supplementary Fig. 54. ^{1}H NMR spectrum of compound **8a**

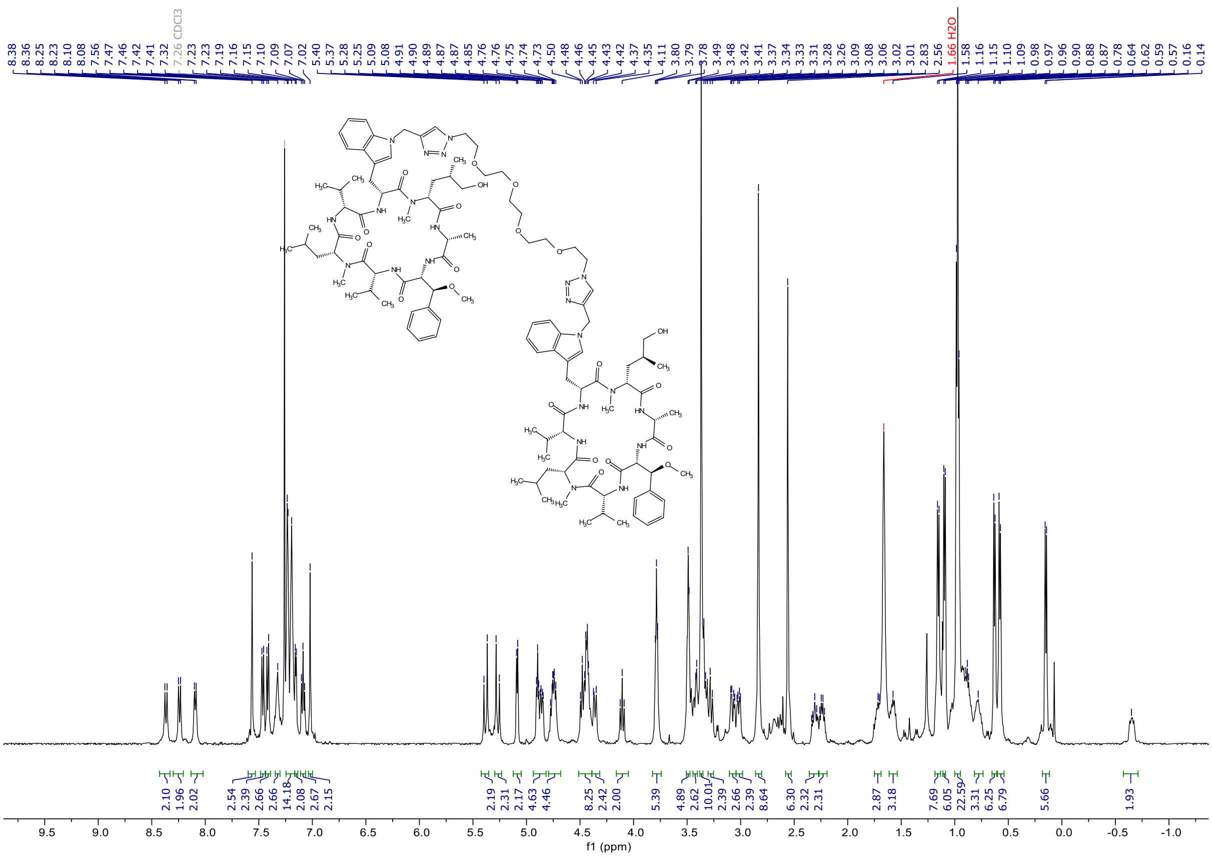

Supplementary Fig. 55. ^{13}C NMR spectrum of compound **8a**

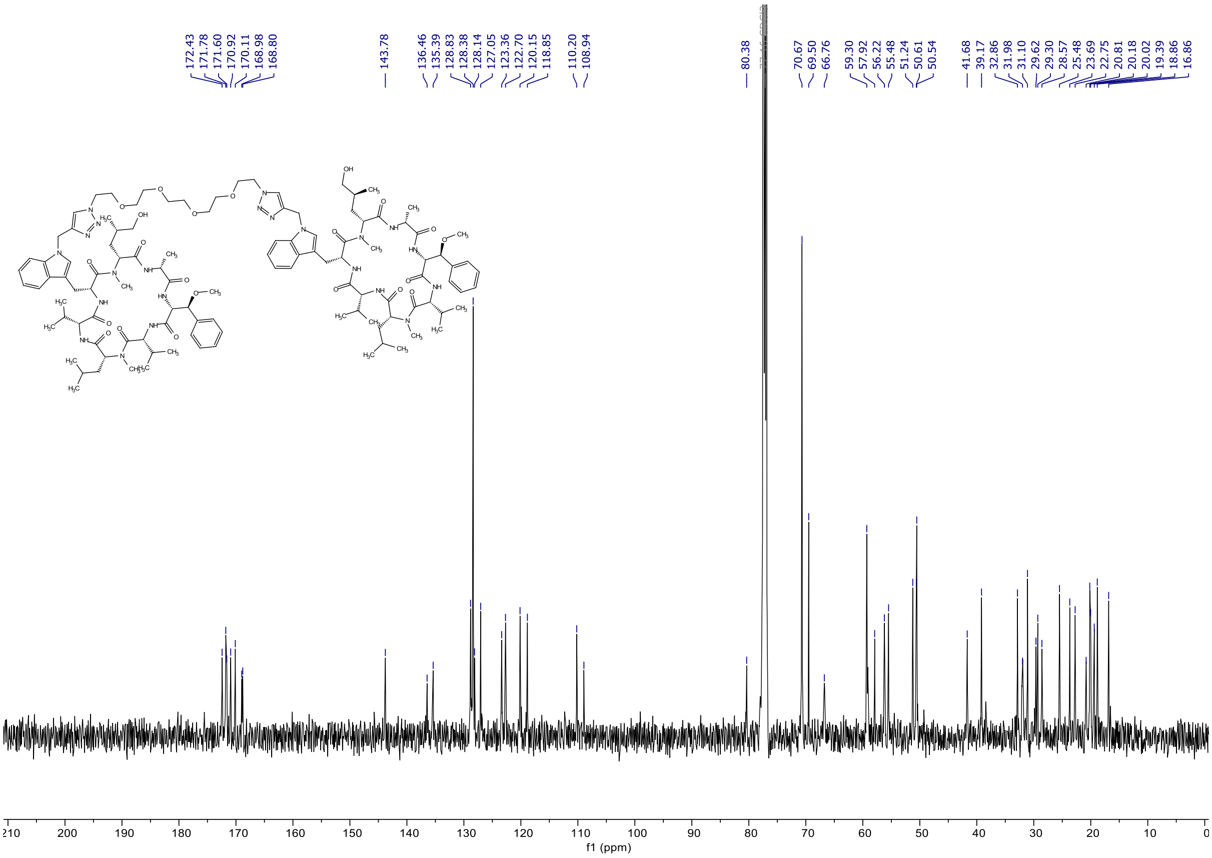

Supplementary Fig. 56. ^{1}H NMR spectrum of compound **SI-18**

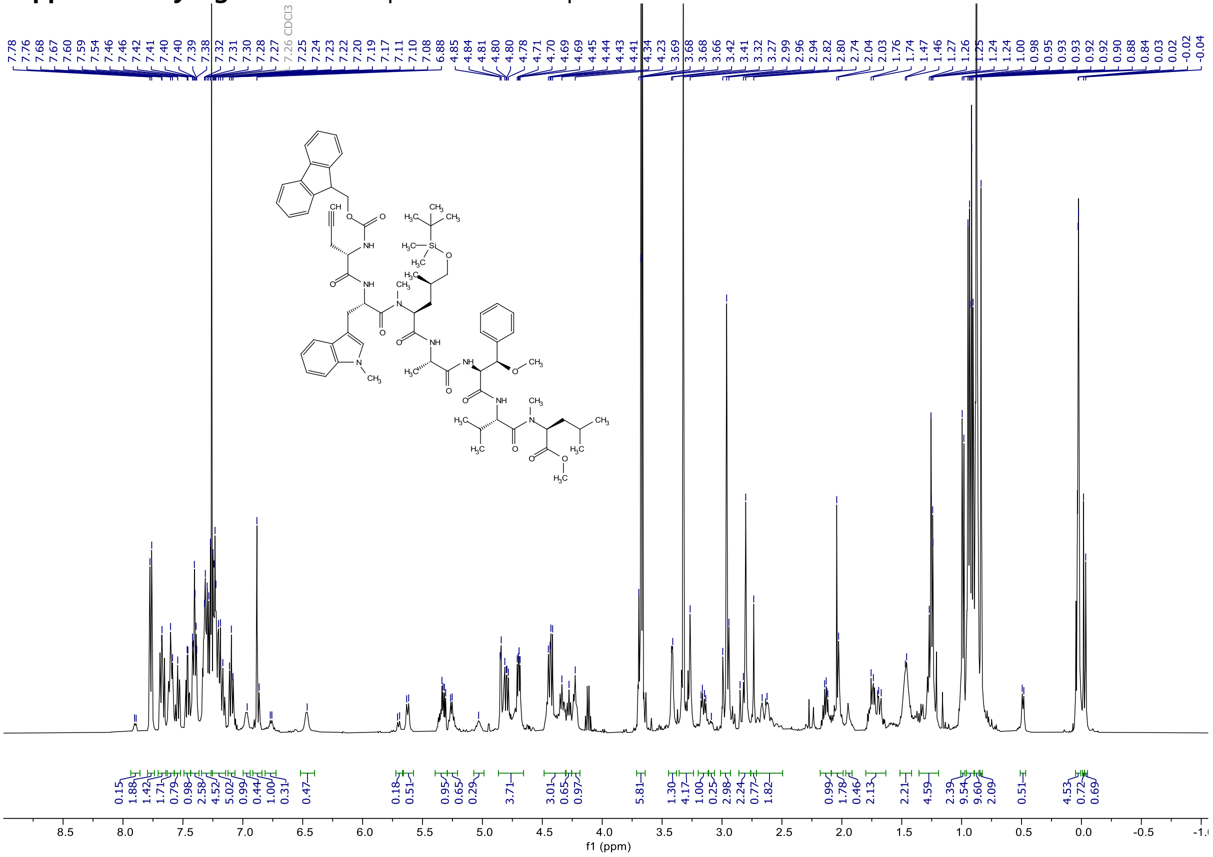

Supplementary Fig. 57. ^{13}C NMR spectrum of compound **SI-18**

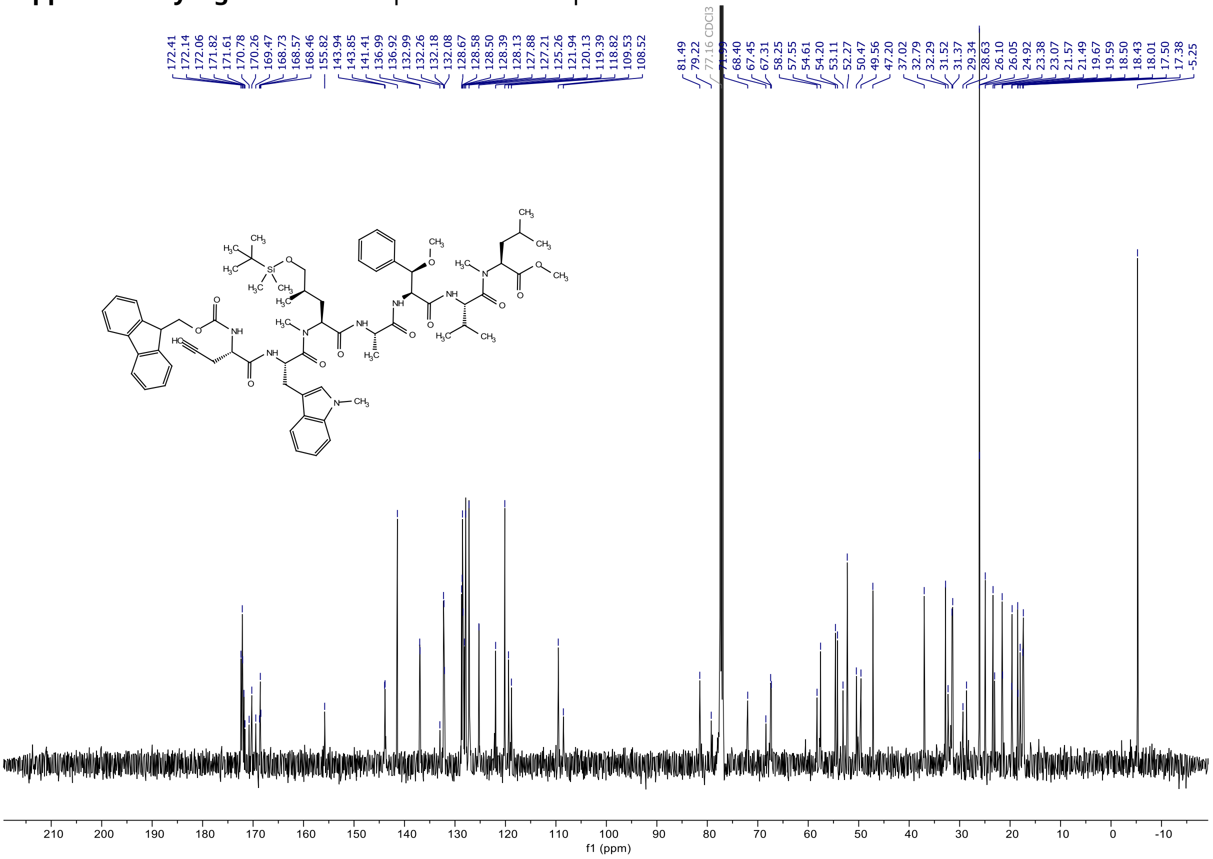

Supplementary Fig. 58. ^{1}H NMR spectrum of compound **10** ($CDCl_3$)

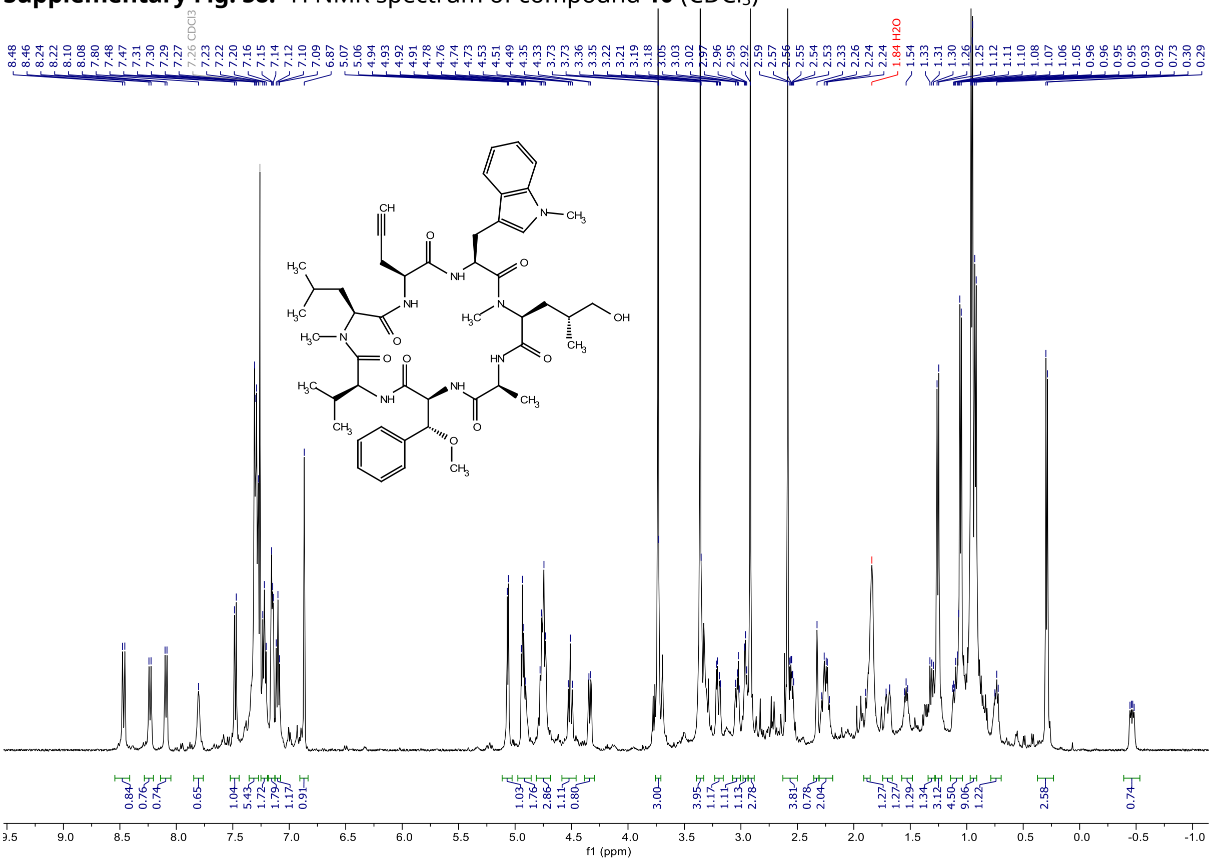

Supplementary Fig. 59. ^{13}C NMR spectrum of compound **10** ($CDCl_3$)

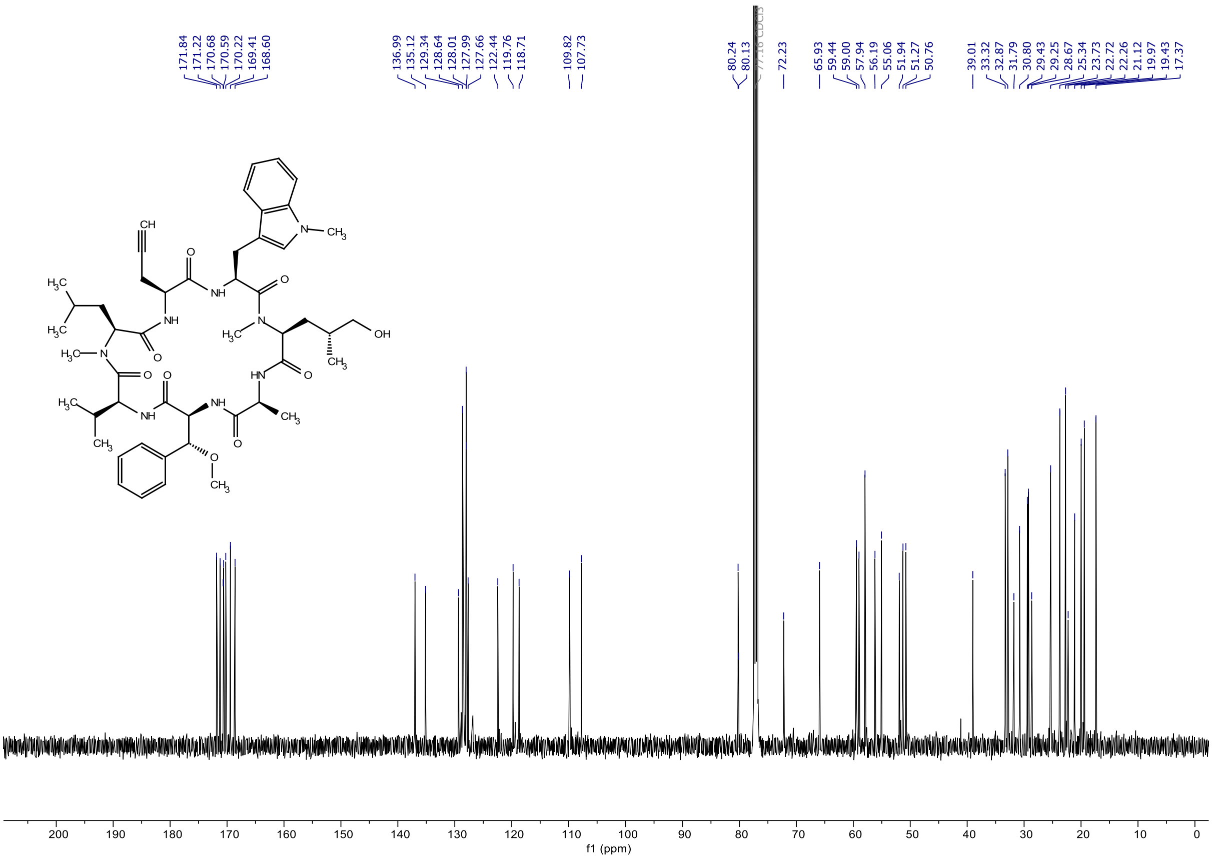

Supplementary Fig. 60. ^{1}H-^{1}H-COSY spectrum of compound **10** ($CDCl_3$)

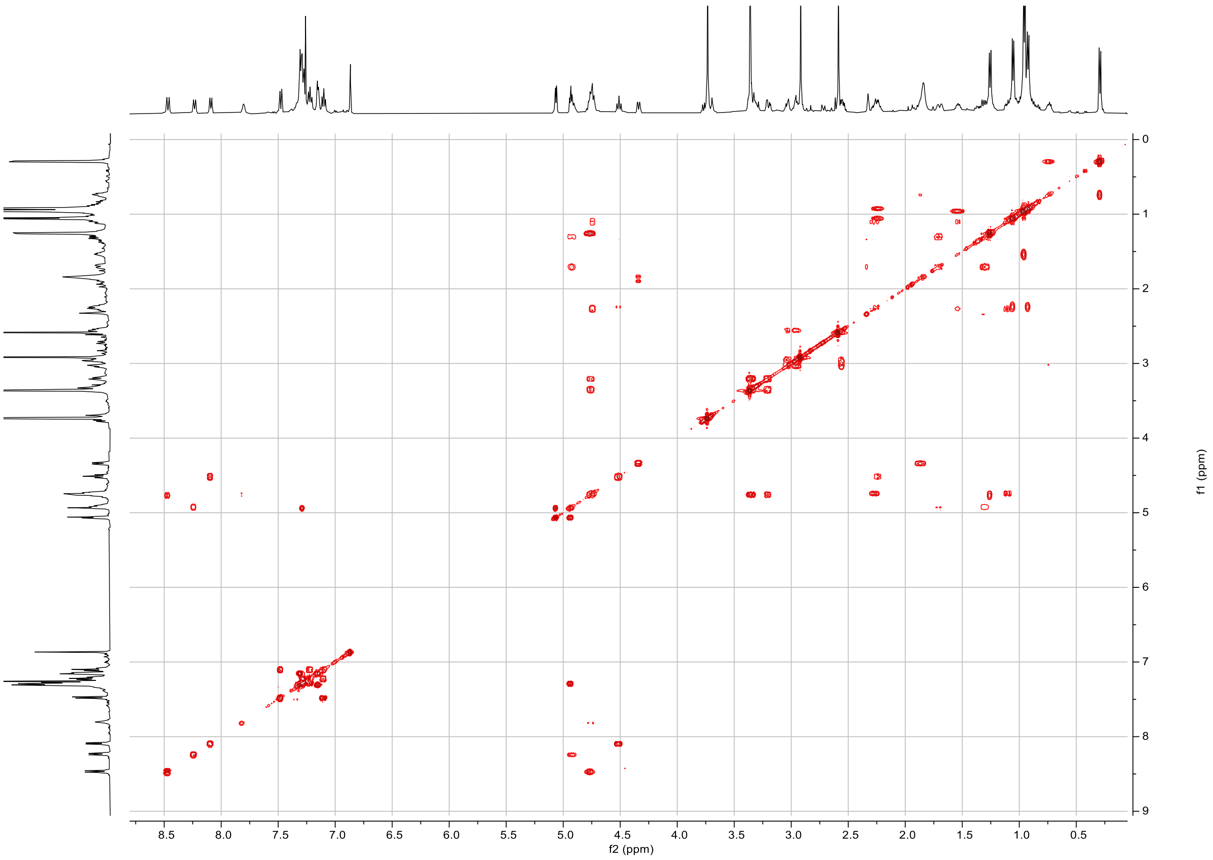

Supplementary Fig. 61. ^{1}H-^{13}C-HSQC(ed) spectrum of compound **10** ($CDCl_3$)

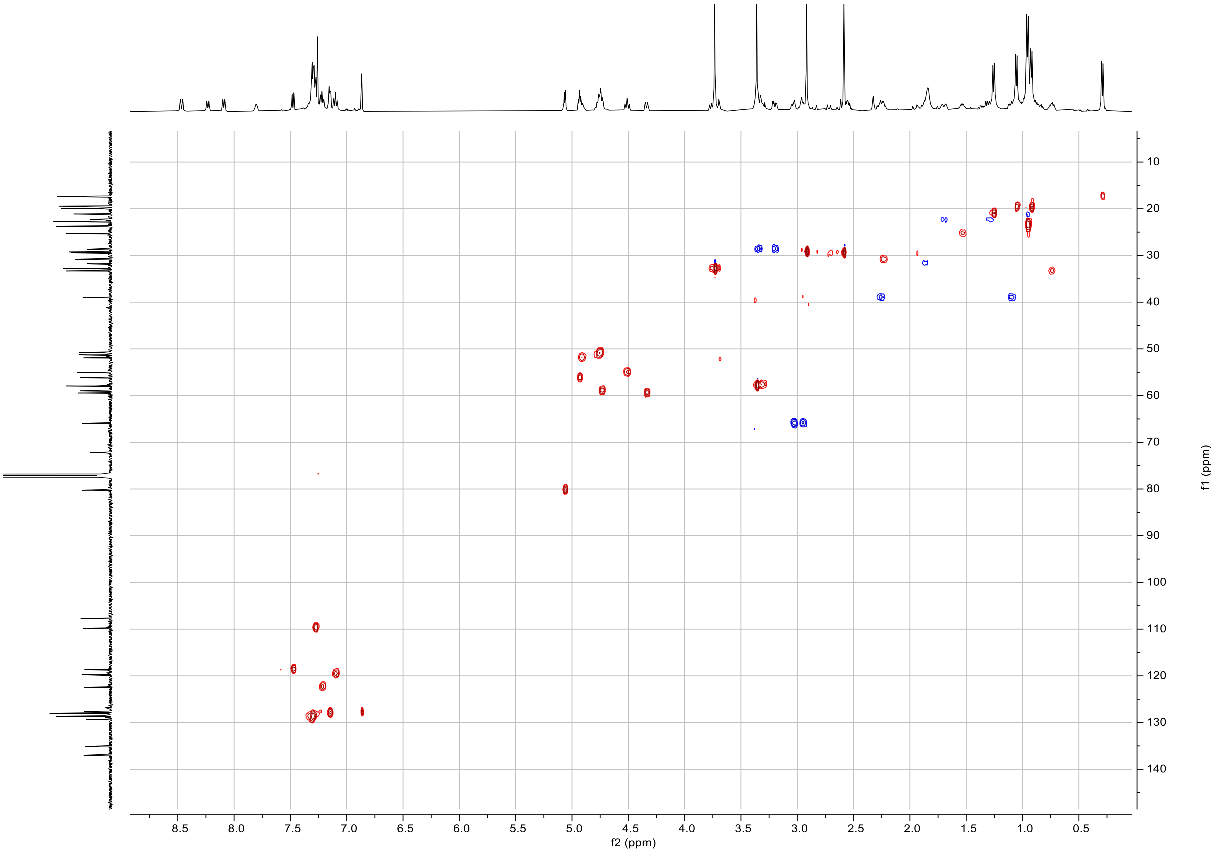

Supplementary Fig. 62. ^1H-^{13}C-HMBC spectrum of compound **10** ($CDCl_3$).

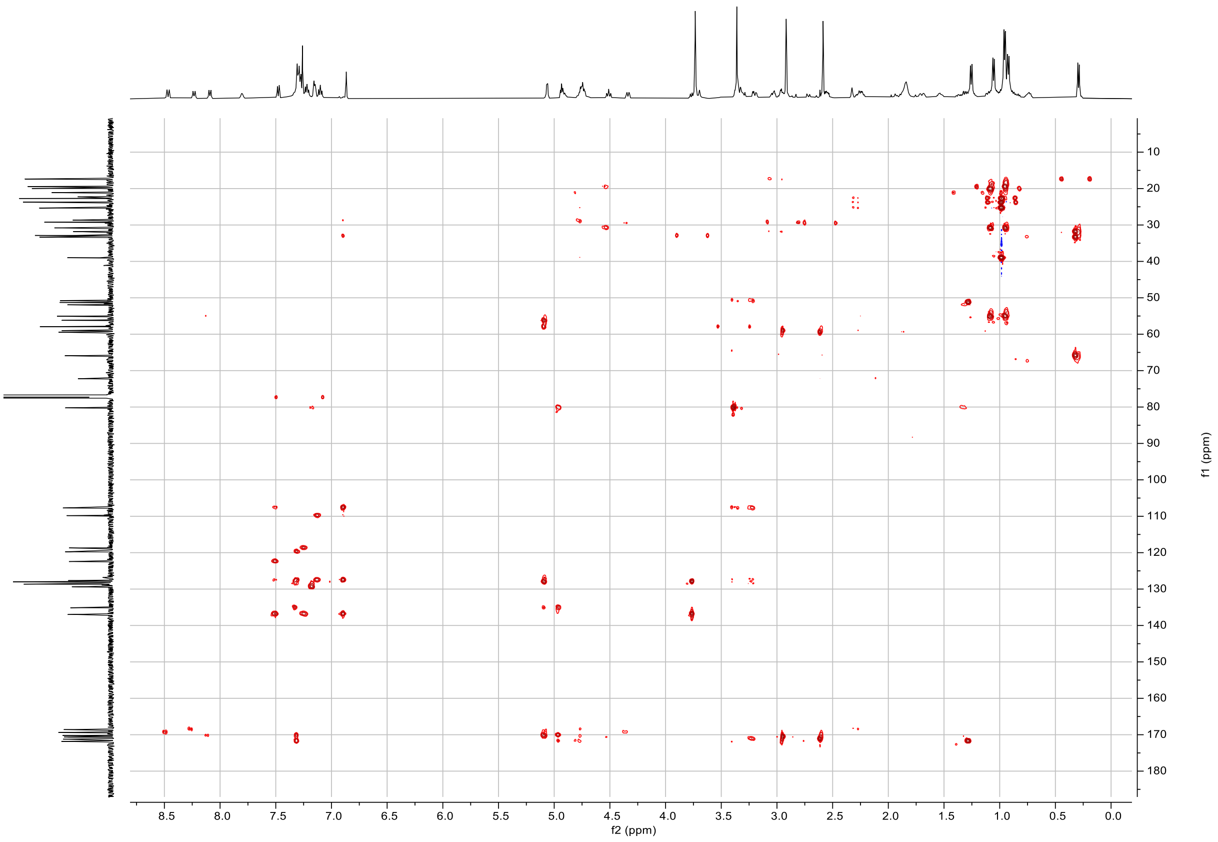

Supplementary Fig. 63. ^1H NMR spectrum of compound **10** (DMSO-d_6)

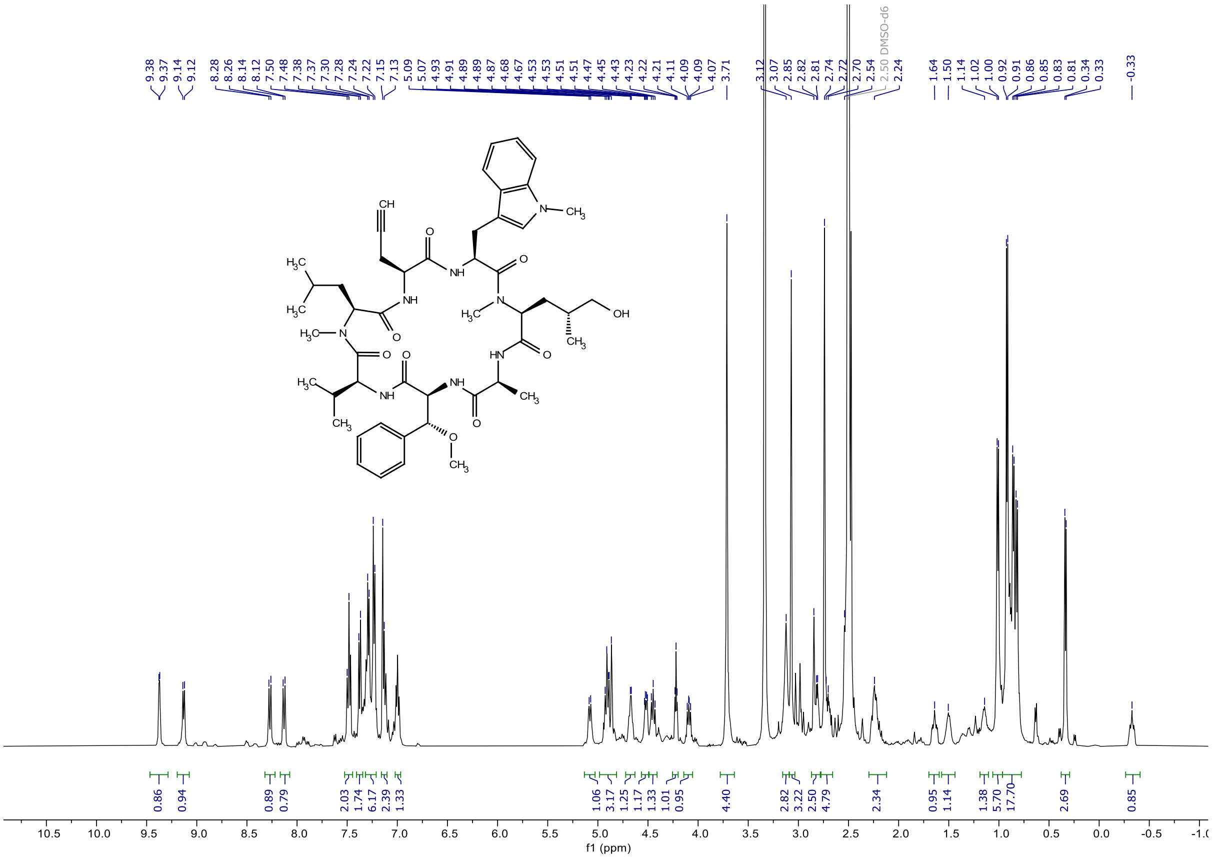

Supplementary Fig. 64. ^{13}C NMR spectrum of compound **10** (DMSO-d$_6$)

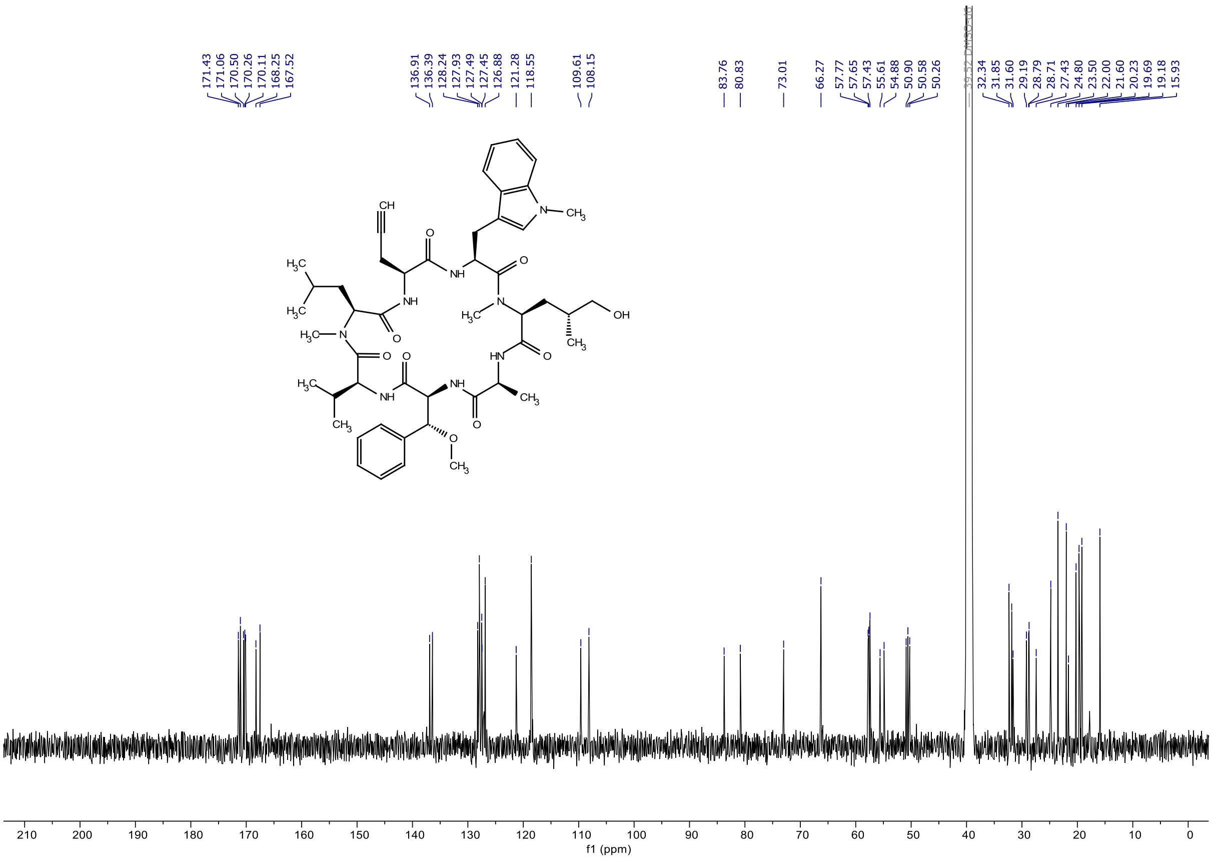

Supplementary Fig. 65. ^{1}H-^{1}H-COSY spectrum of compound **10** (DMSO-d$_6$)

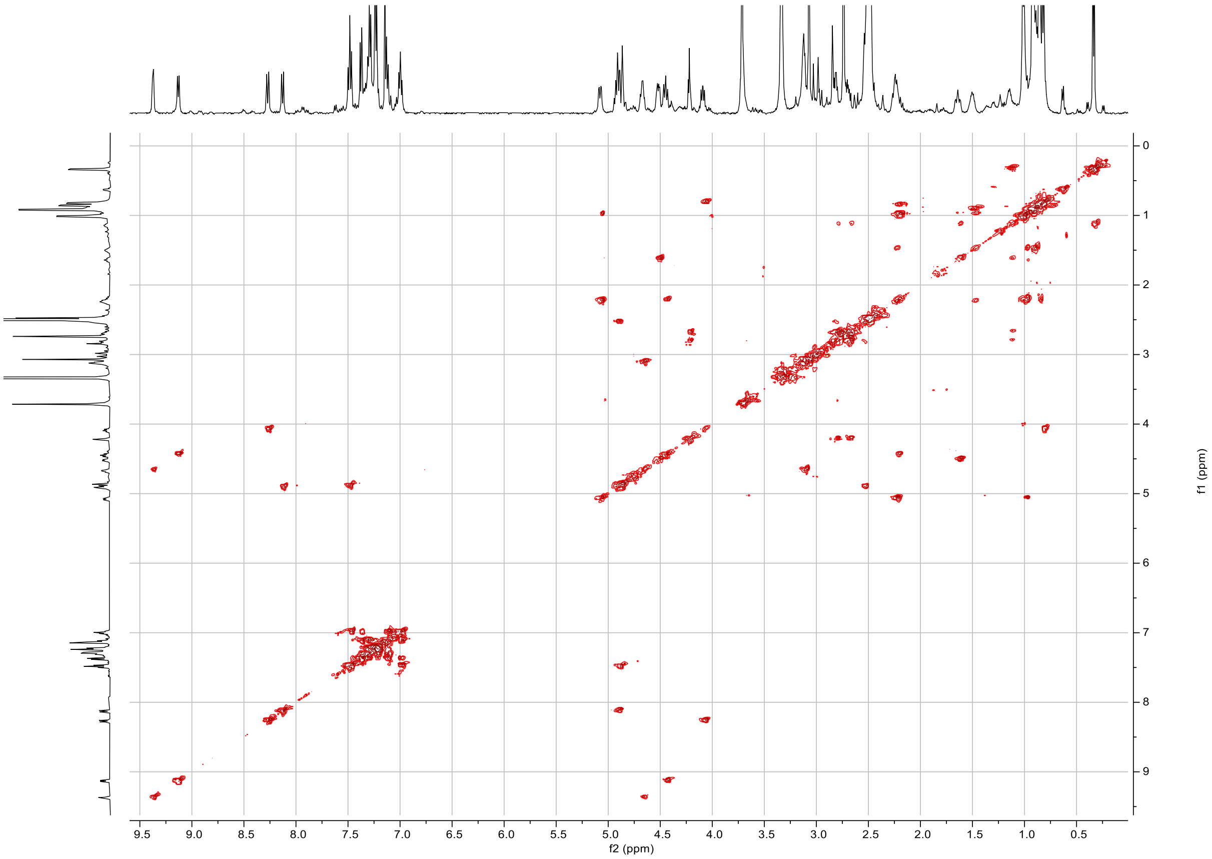

Supplementary Fig. 66. ^{1}H-^{13}C-HSQC(ed) spectrum of compound **10** (DMSO-d_6).

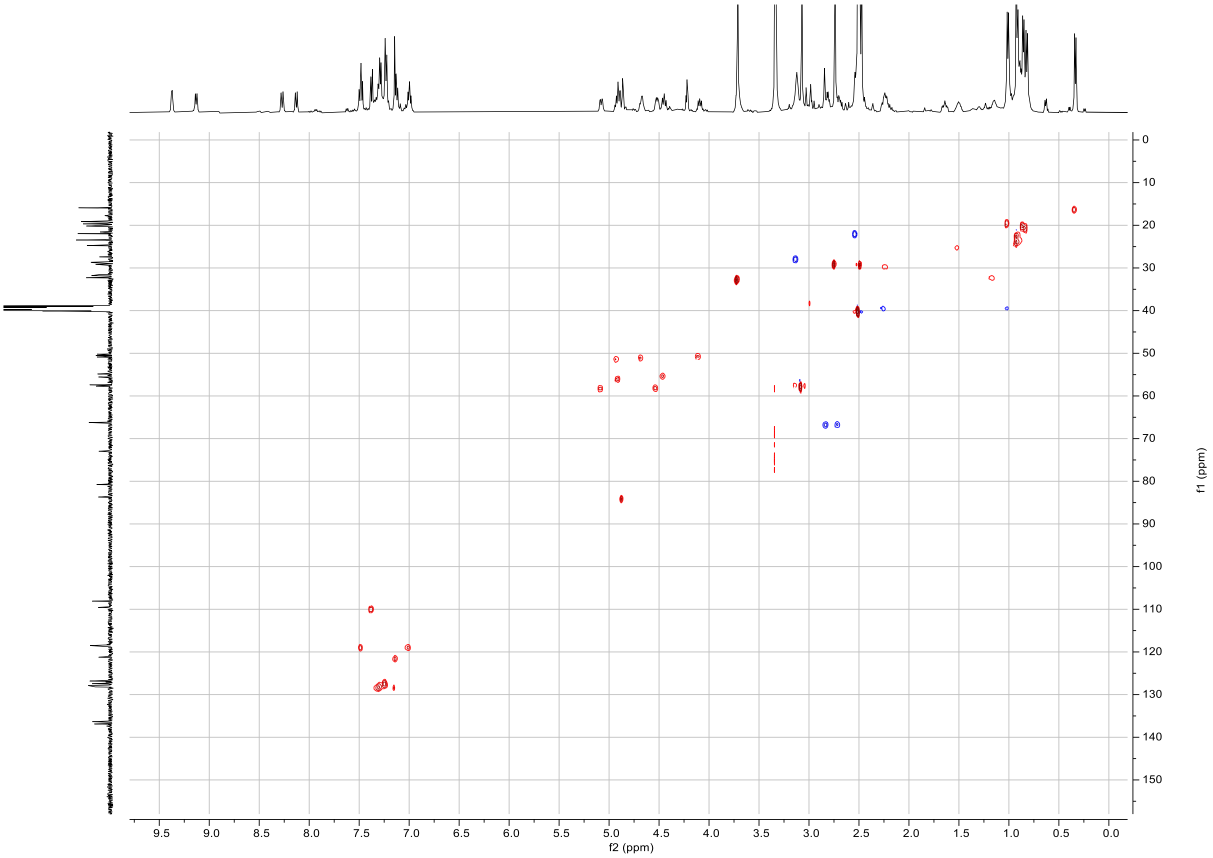

Supplementary Fig. 67. ^{1}H-^{13}C-HMBC spectrum of compound **10** (DMSO-d_6)

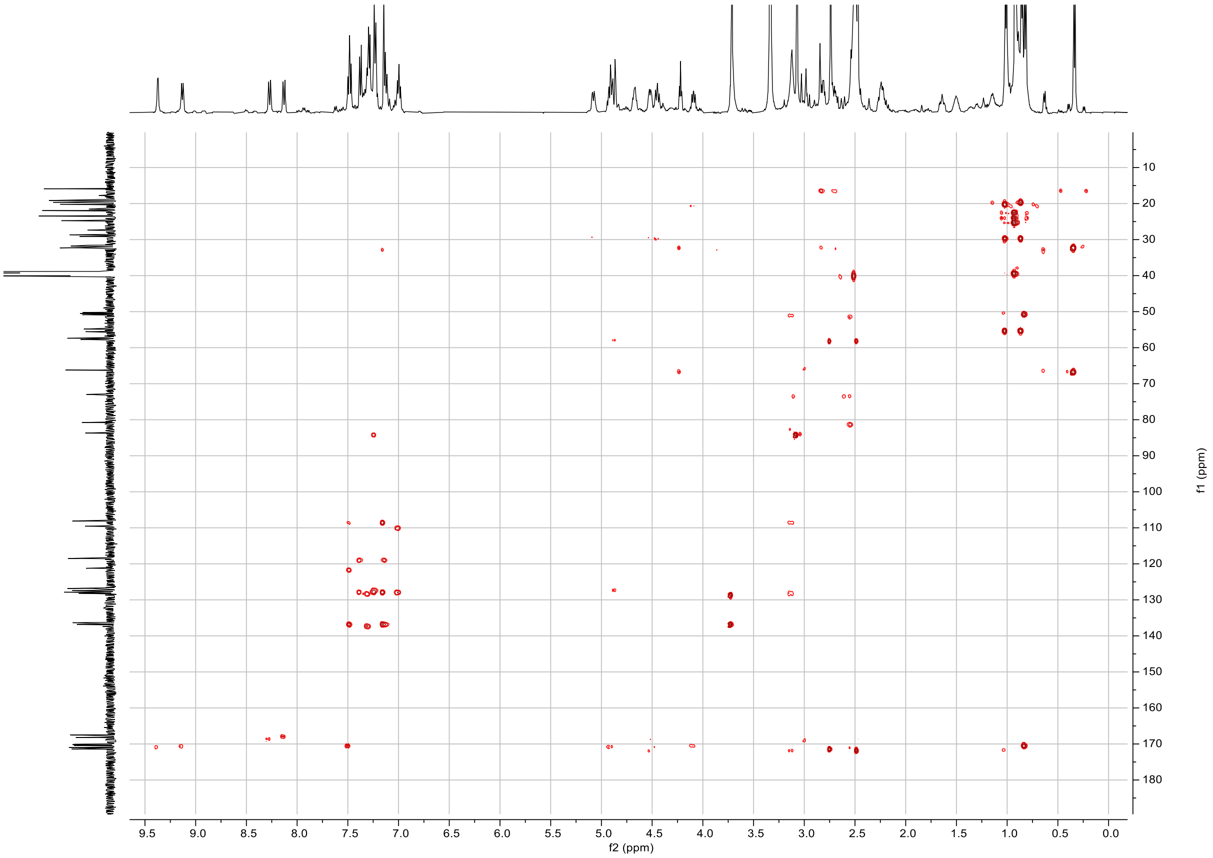

Supplementary Fig. 68. ^{1}H NMR spectrum of compound **11**

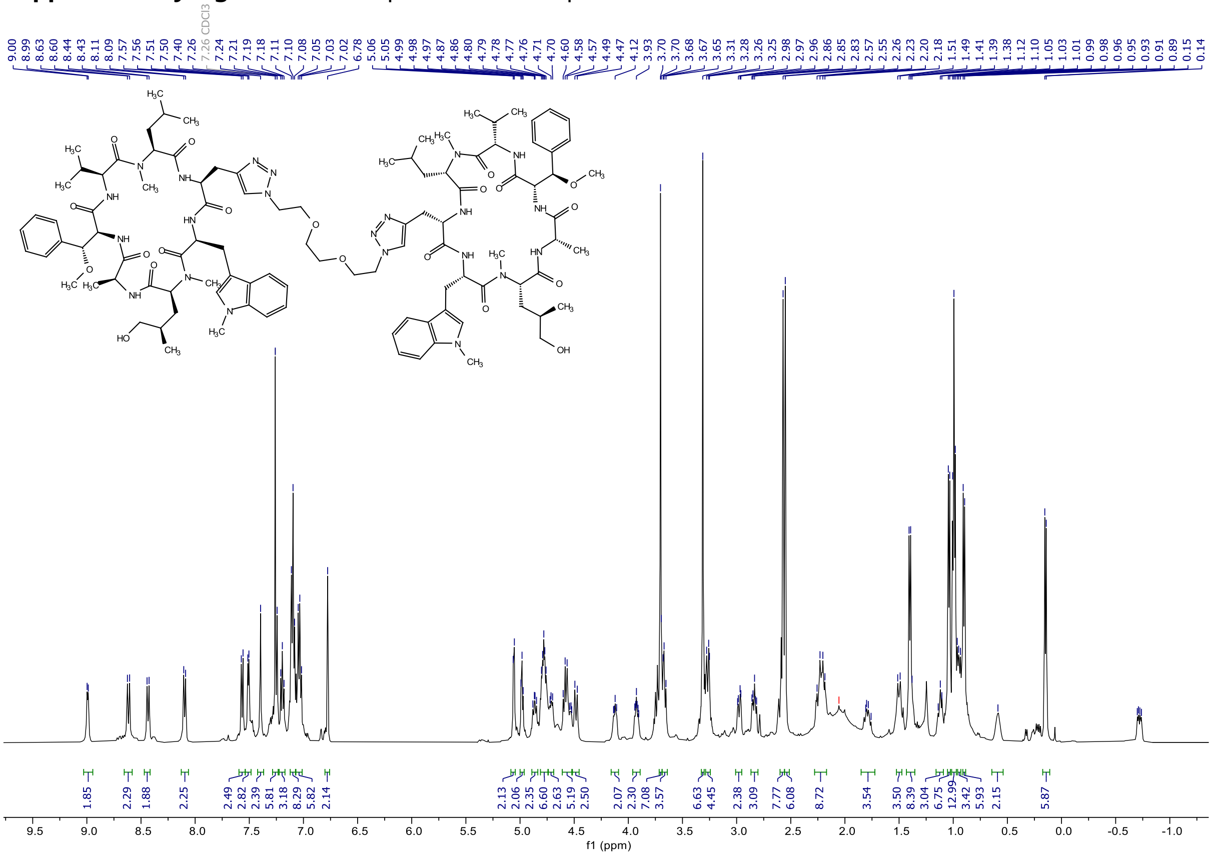

Supplementary Fig. 69. ^{13}C NMR spectrum of compound **11**

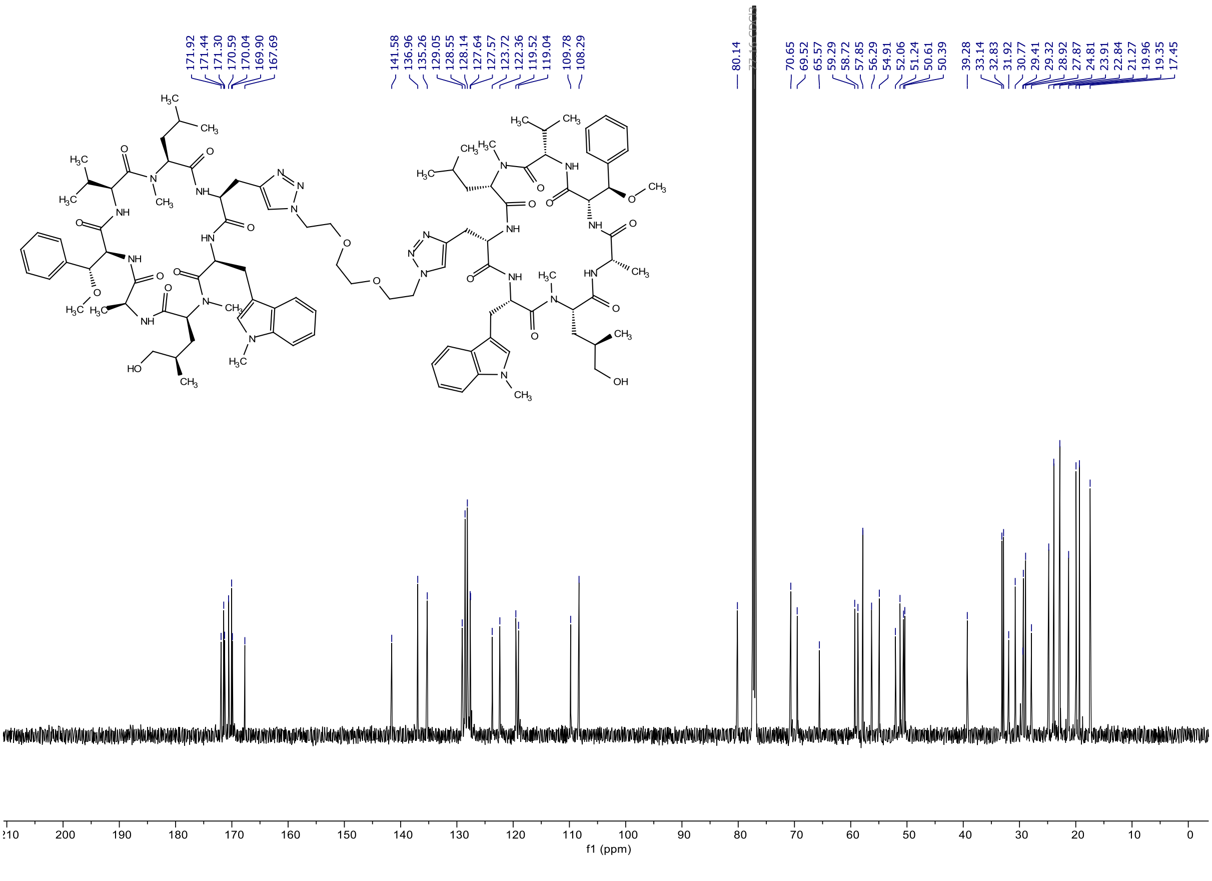

Supplementary Fig. 70. ^{1}H NMR spectrum of compound **12**

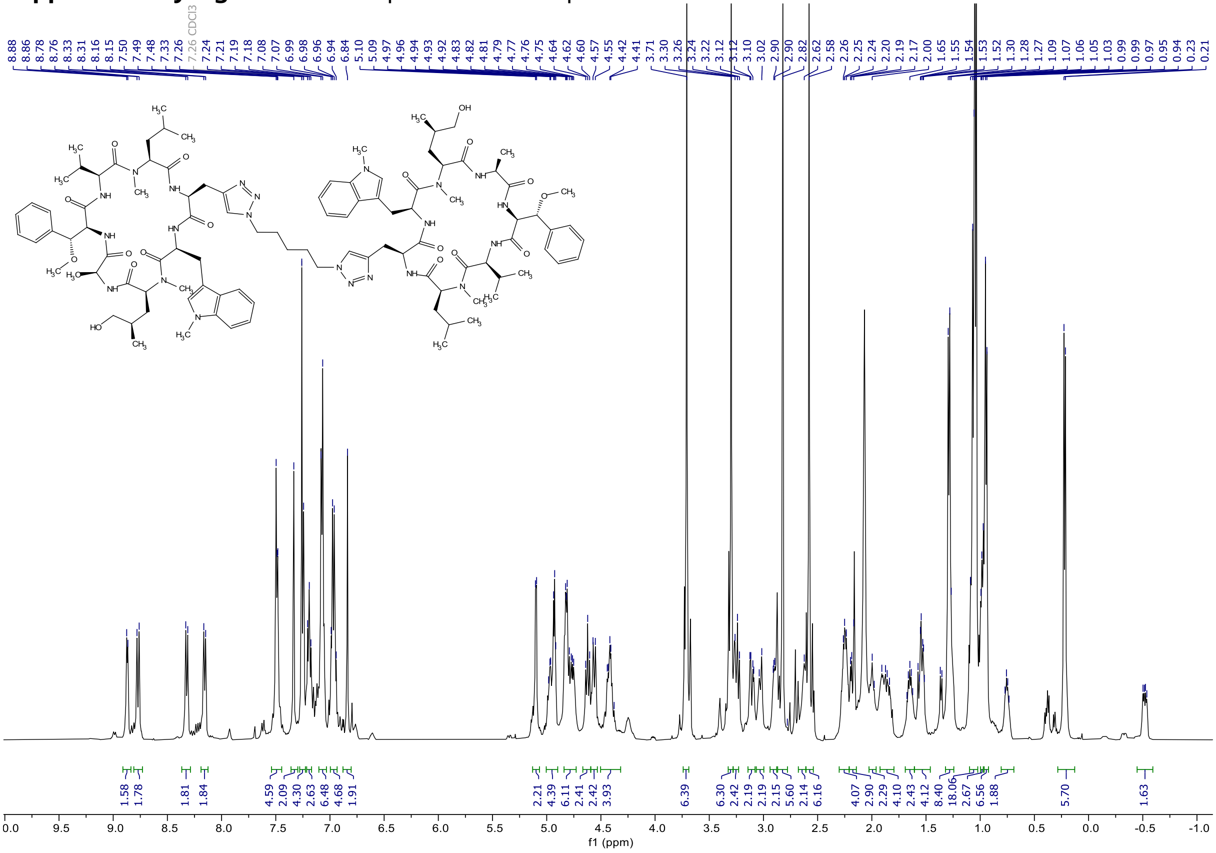

Supplementary Fig. 71. ^{13}C NMR spectrum of compound **12**

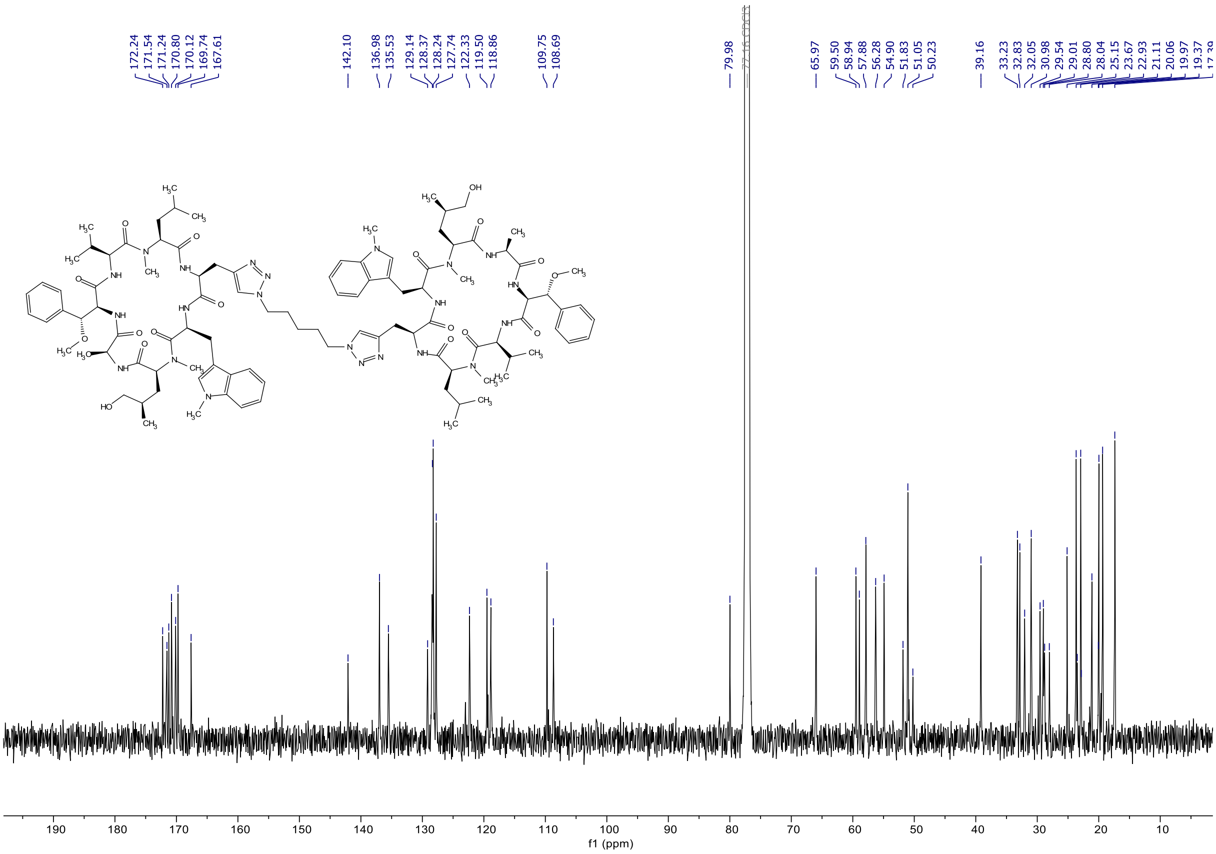

Supplementary Fig. 72. ^{1}H NMR spectrum of compound **24**

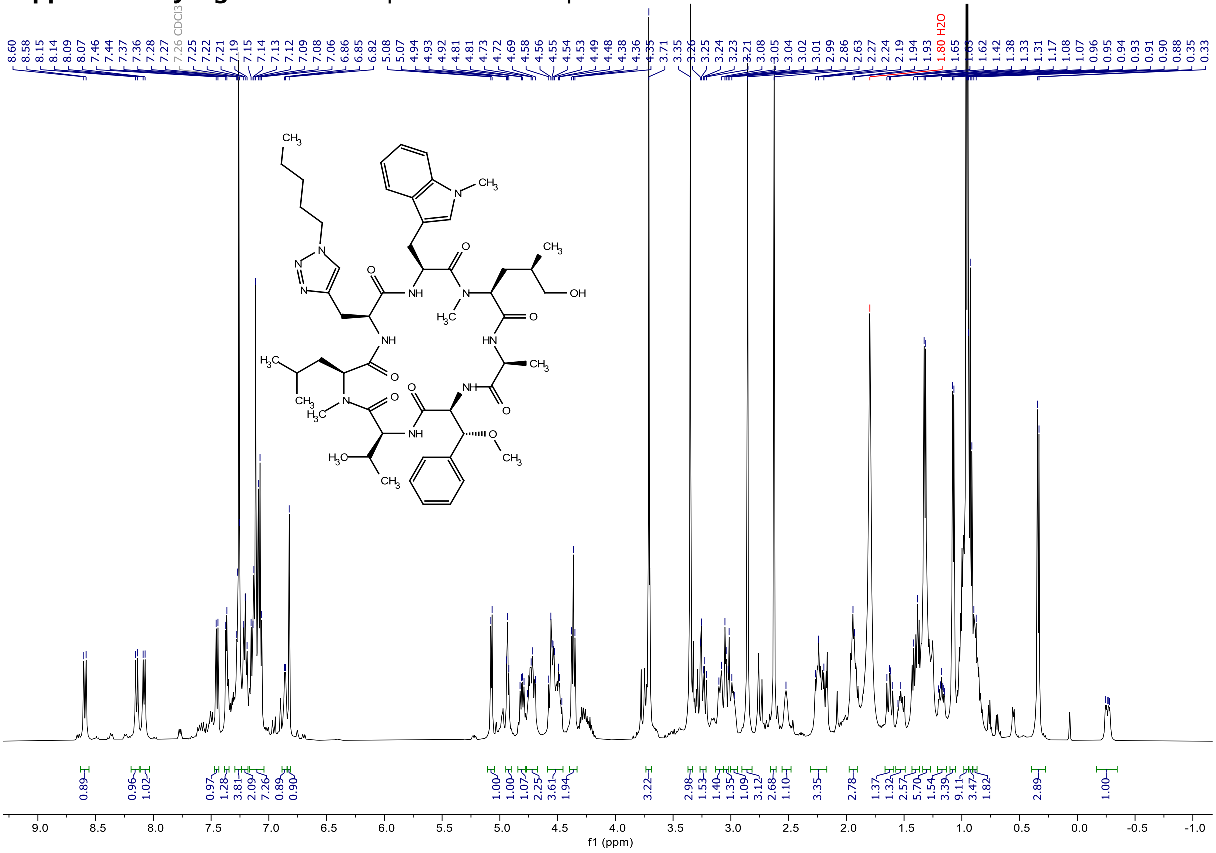

Supplementary Fig. 73. ^{13}C NMR spectrum of compound **24**

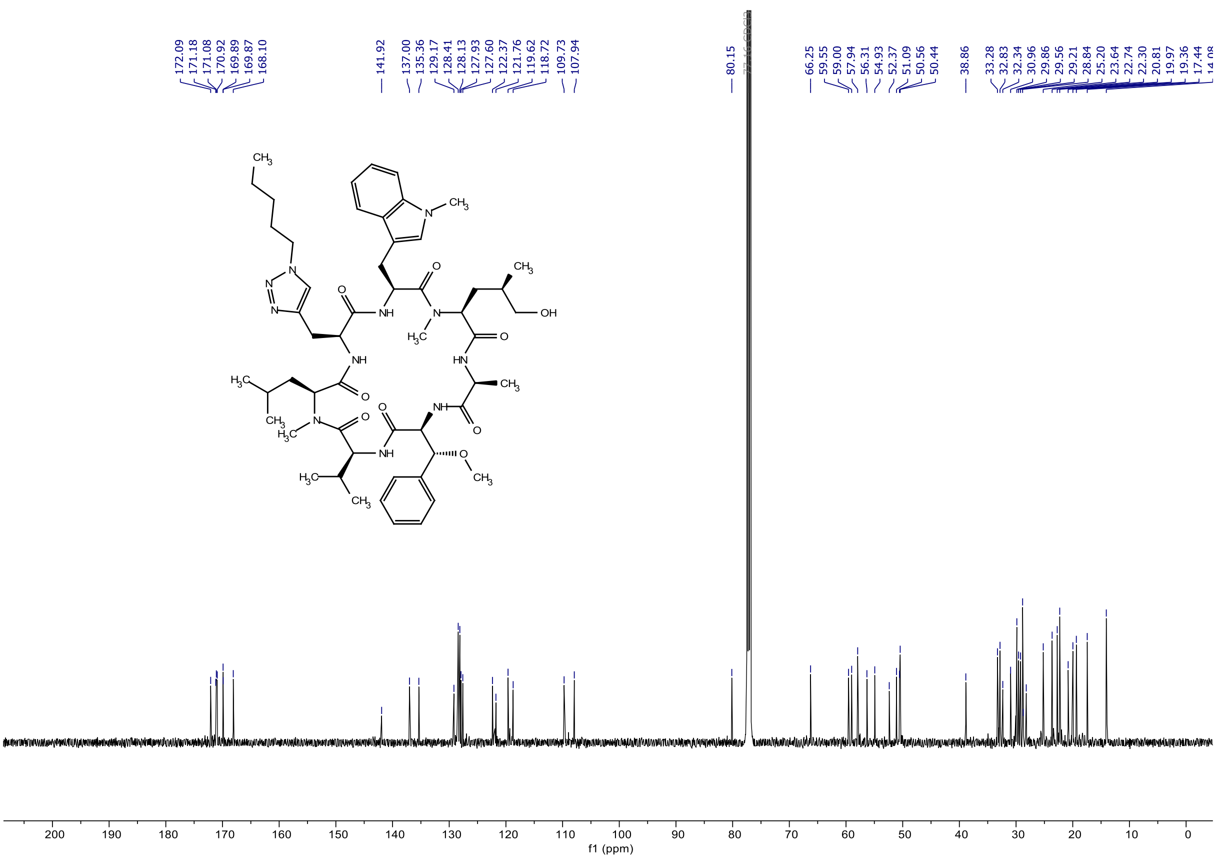

Supplementary Fig. 74. ^{1}H NMR spectrum of compound **SI-19**

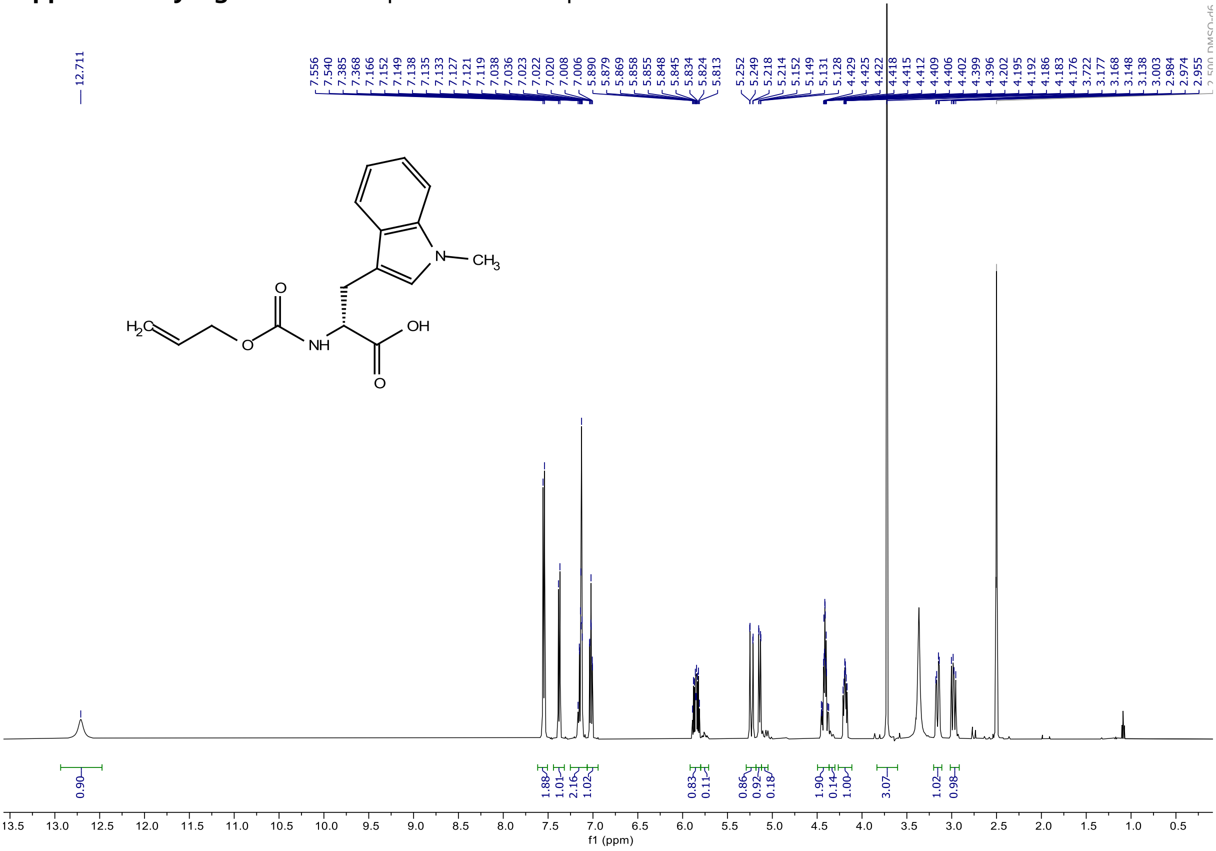

Supplementary Fig. 75. ^{13}C NMR spectrum of compound **SI-19**

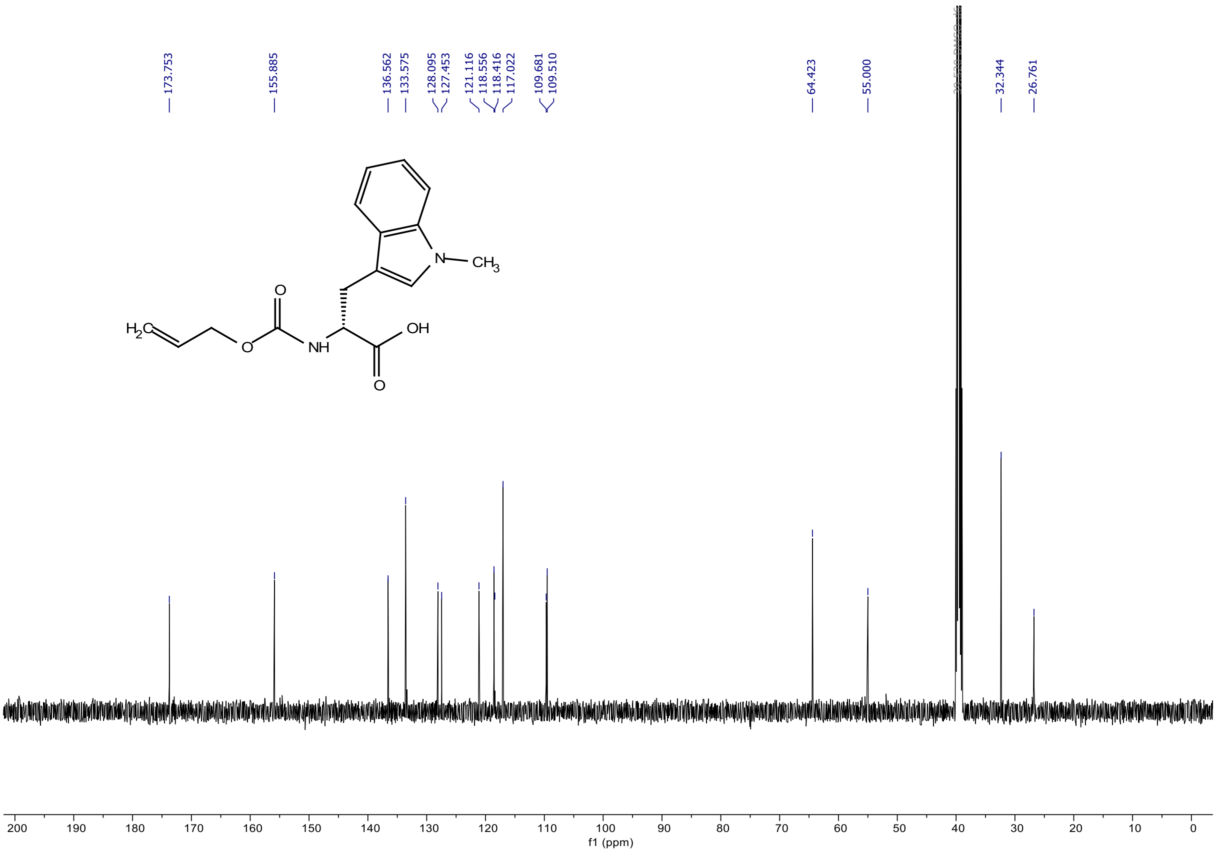

Supplementary Fig. 76. ^{1}H NMR spectrum of compound **SI-20**

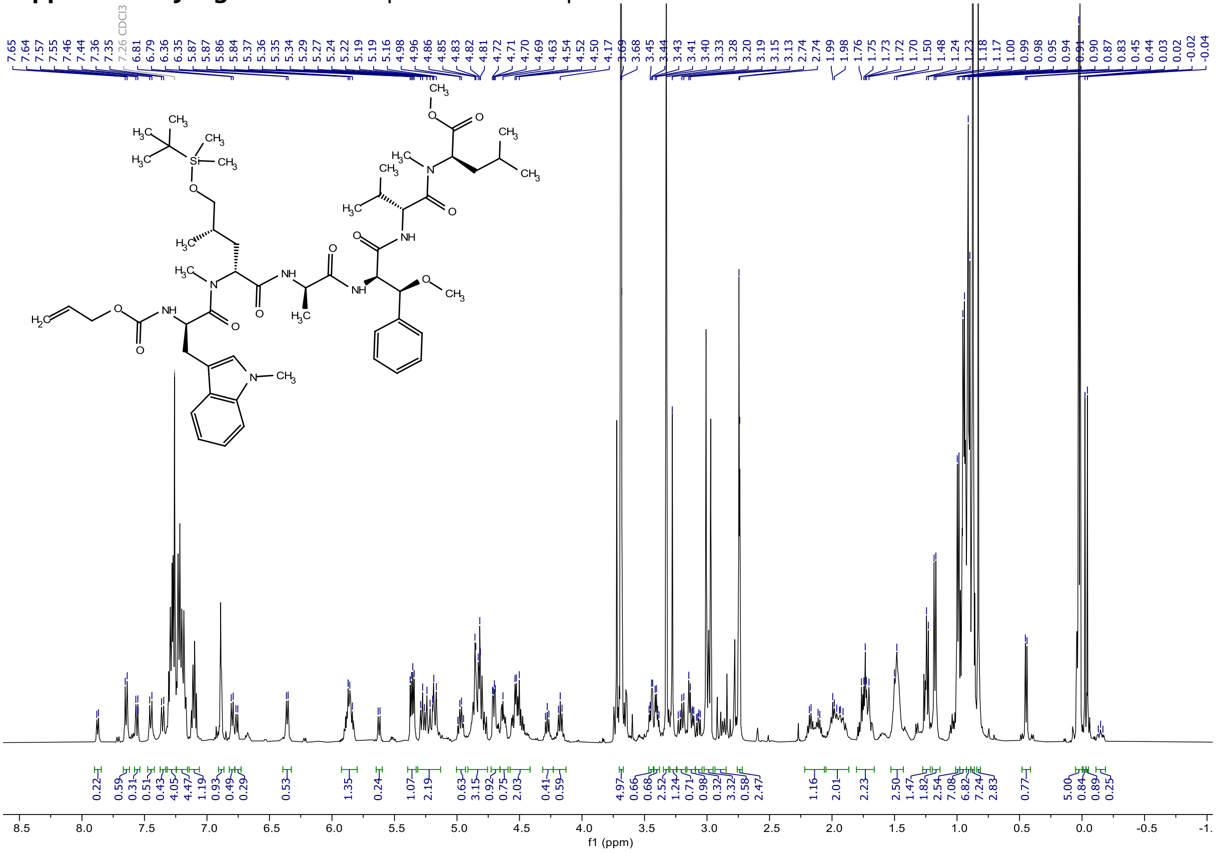

Supplementary Fig. 77. ^{13}C NMR spectrum of compound **SI-20**

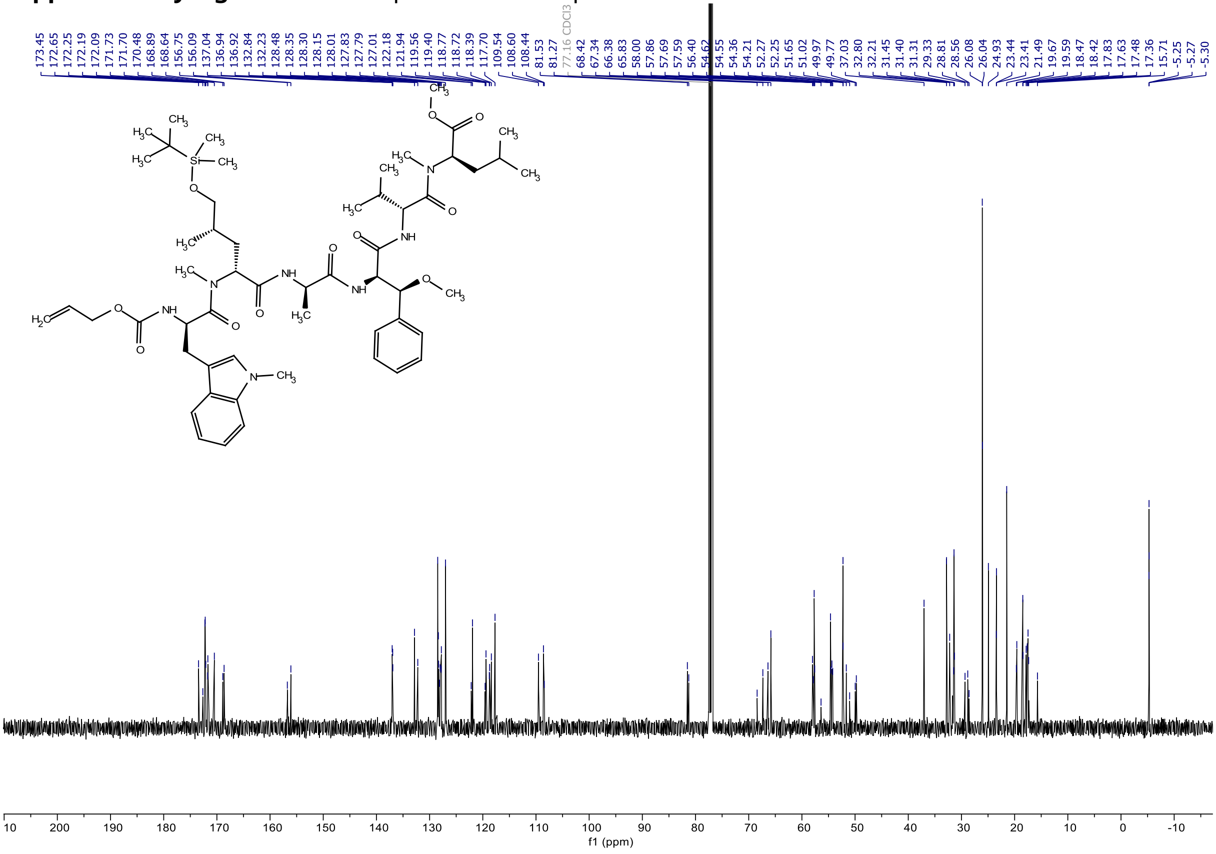

Supplementary Fig. 78. ^{1}H NMR spectrum of compound **SI-18a**

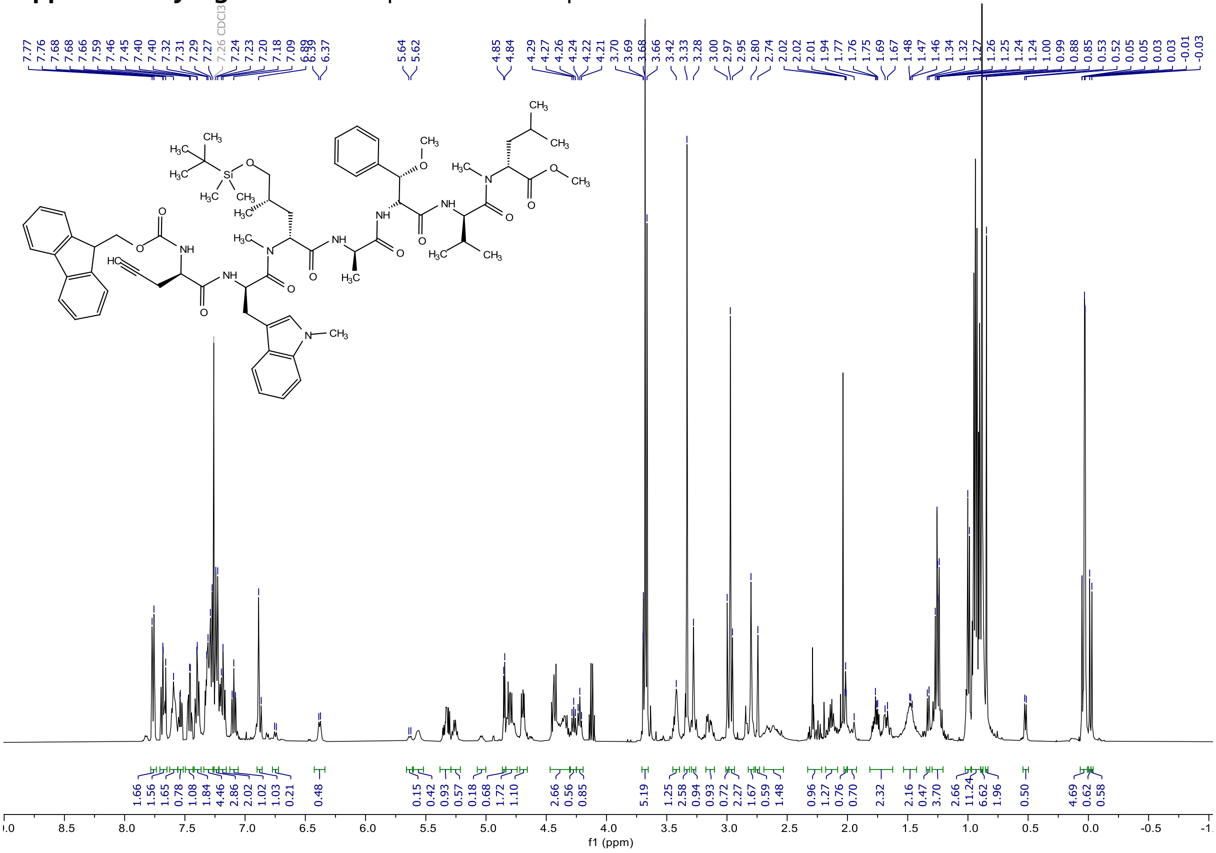

Supplementary Fig. 79. ^{13}C NMR spectrum of compound **SI-18a**

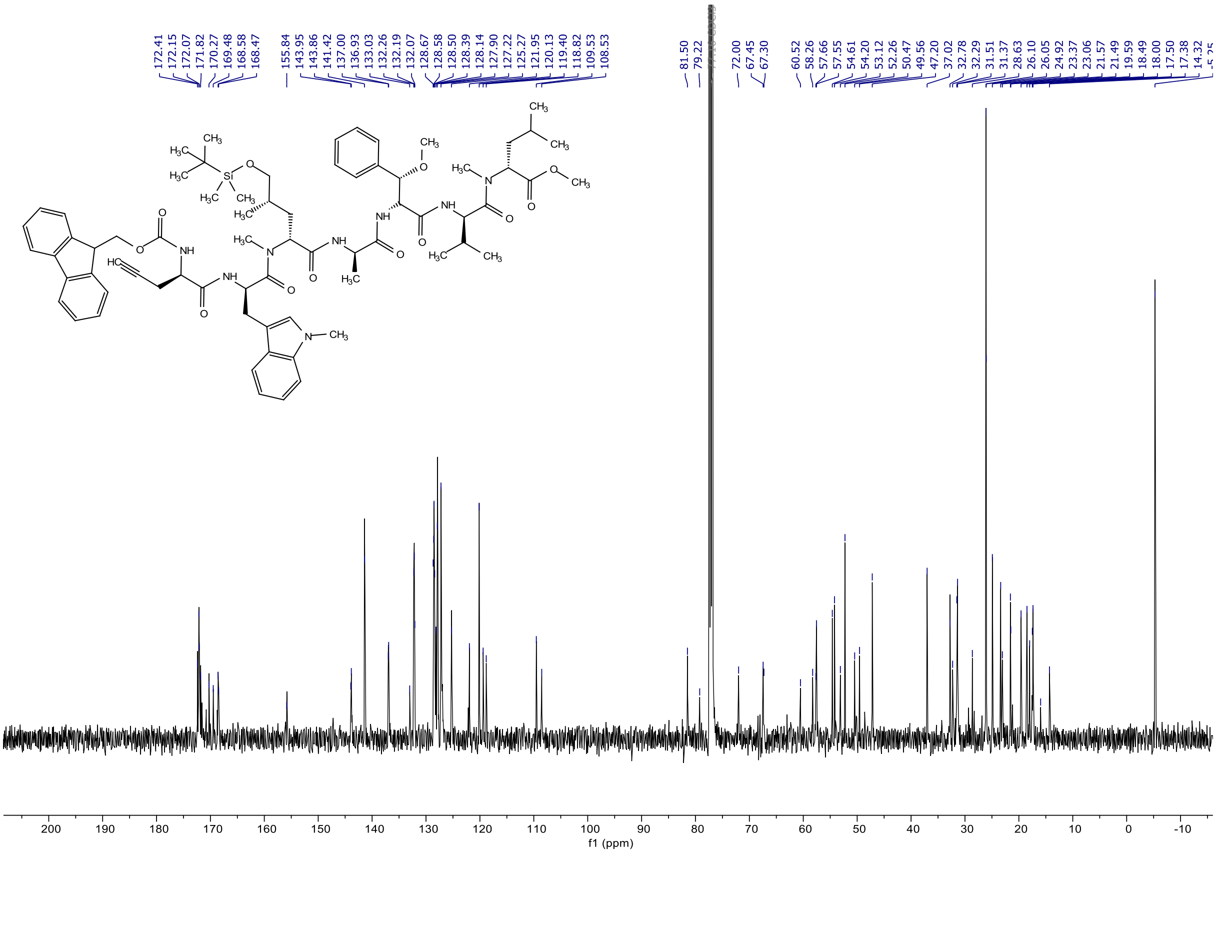

Supplementary Fig. 80. ^{1}H NMR spectrum of compound **10a**

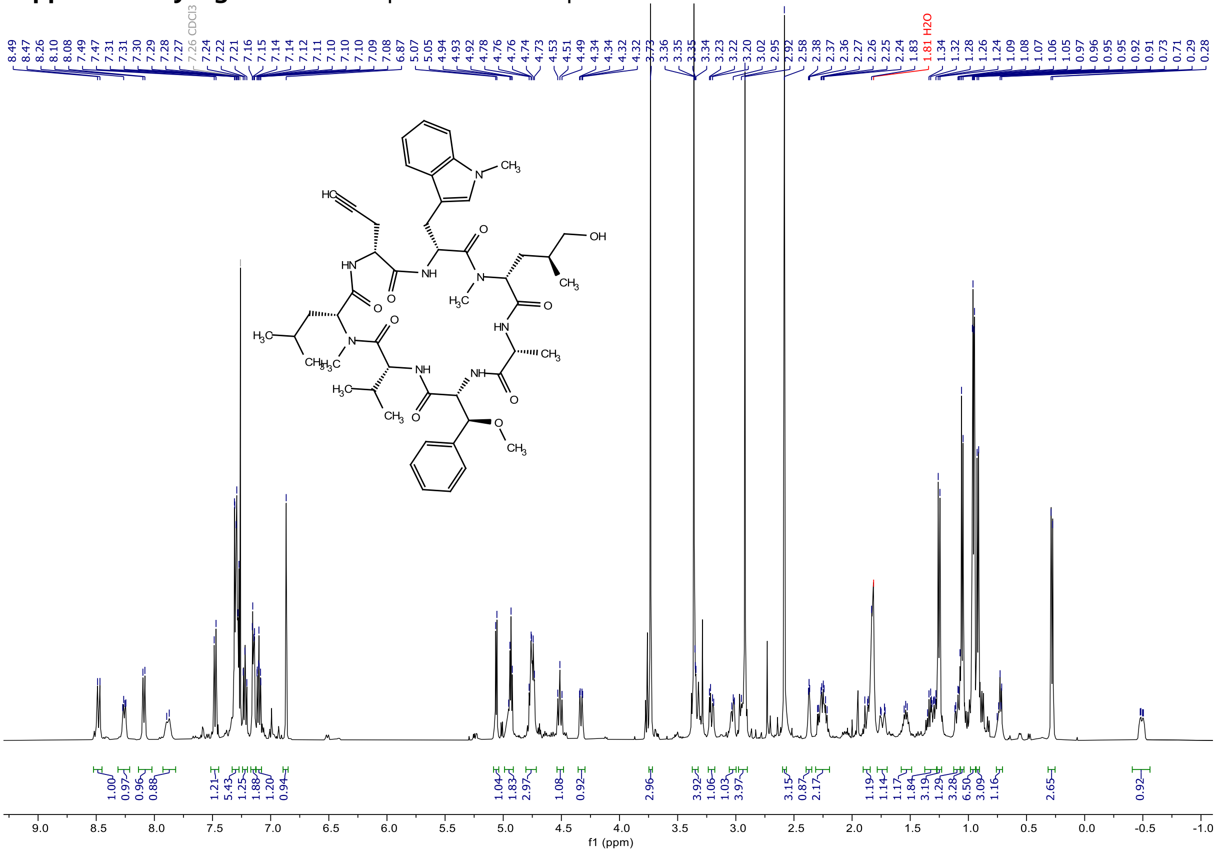

Supplementary Fig. 81. ^{13}C NMR spectrum of compound **10a**

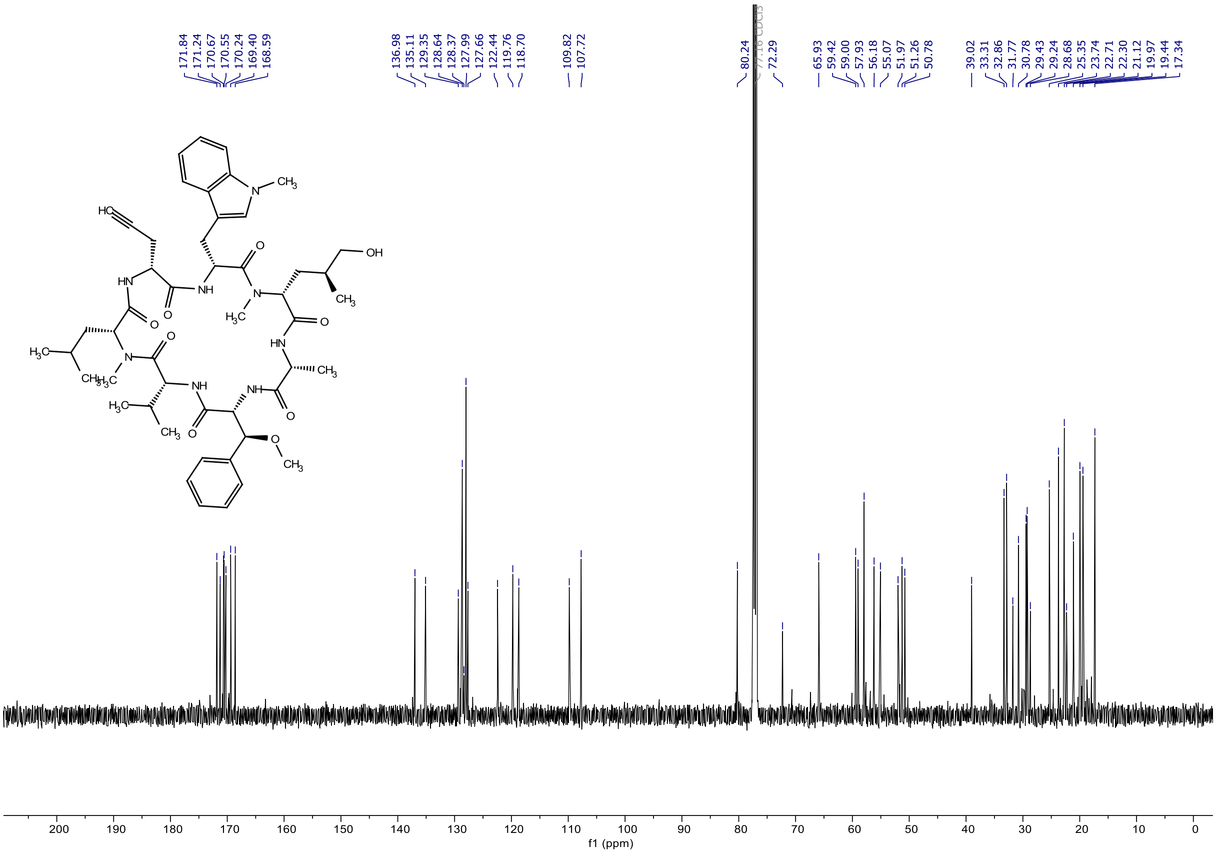

Supplementary Fig. 82. ^{1}H NMR spectrum of compound **12a**

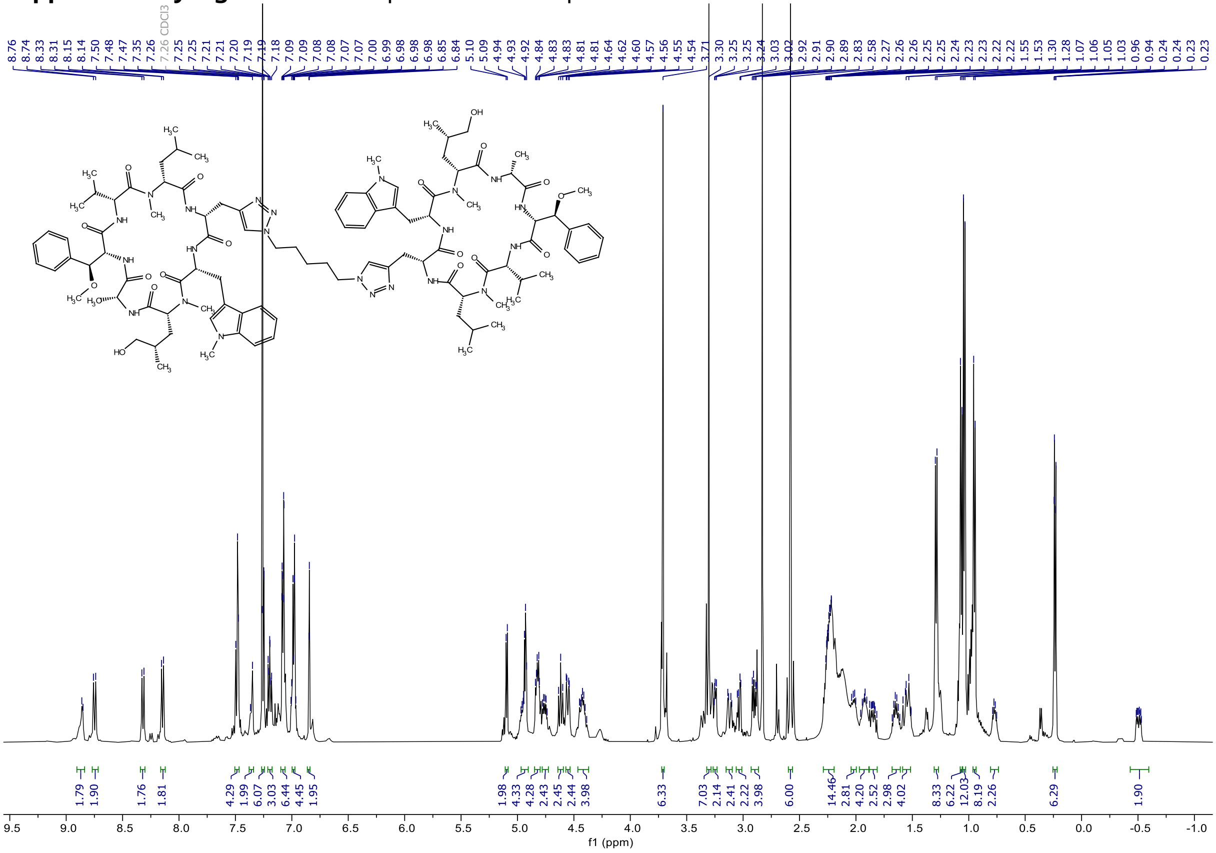

Supplementary Fig. 83. ^{13}C NMR spectrum of compound **12a**

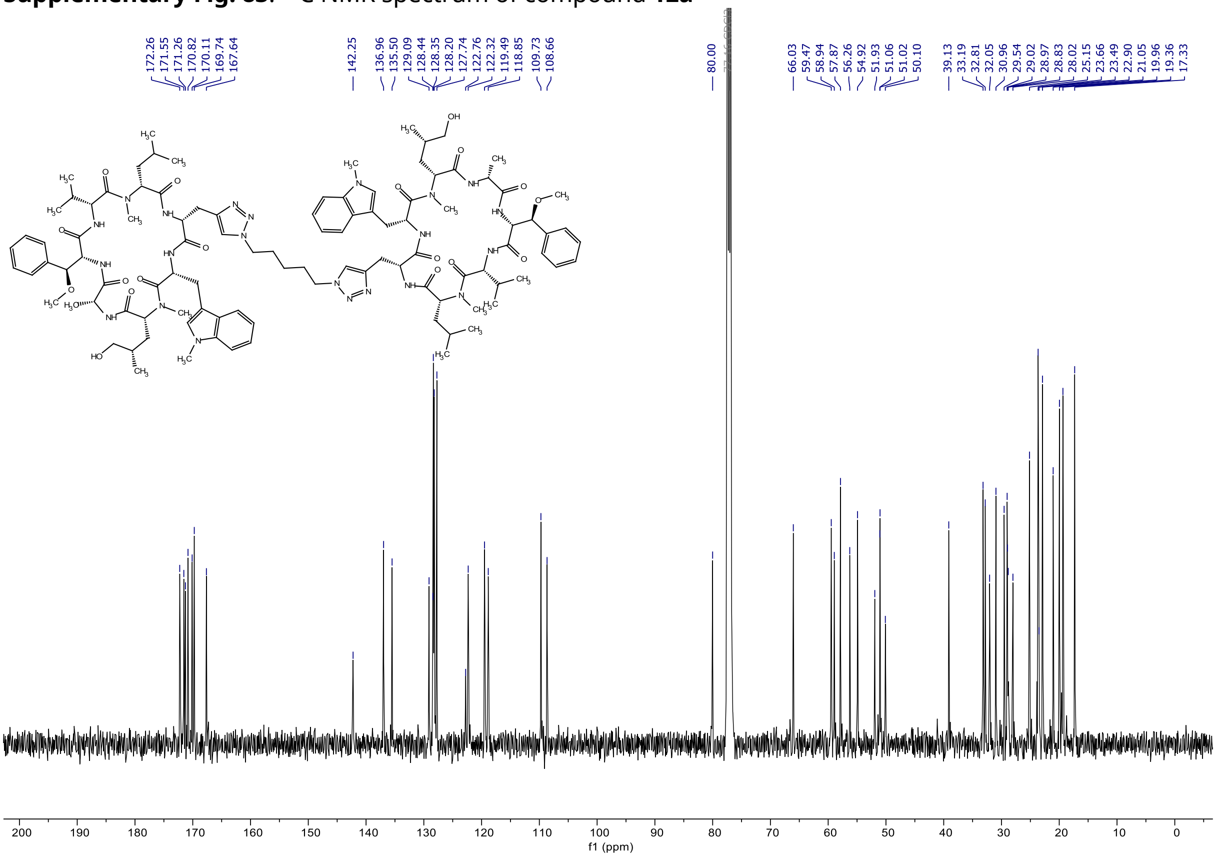

Supplementary Fig. 84. ^{1}H NMR spectrum of compound **SI-21**

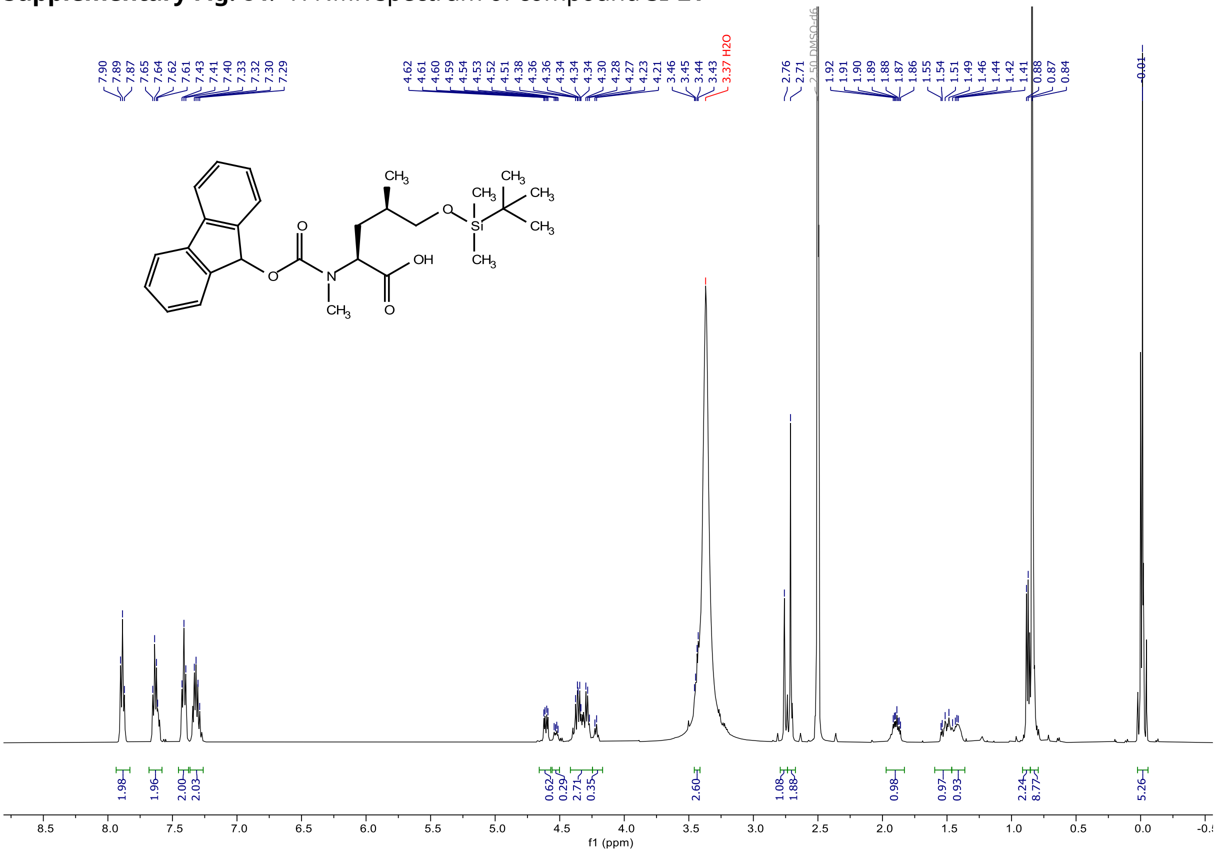

Supplementary Fig. 85. ^{13}C NMR spectrum of compound **SI-21**

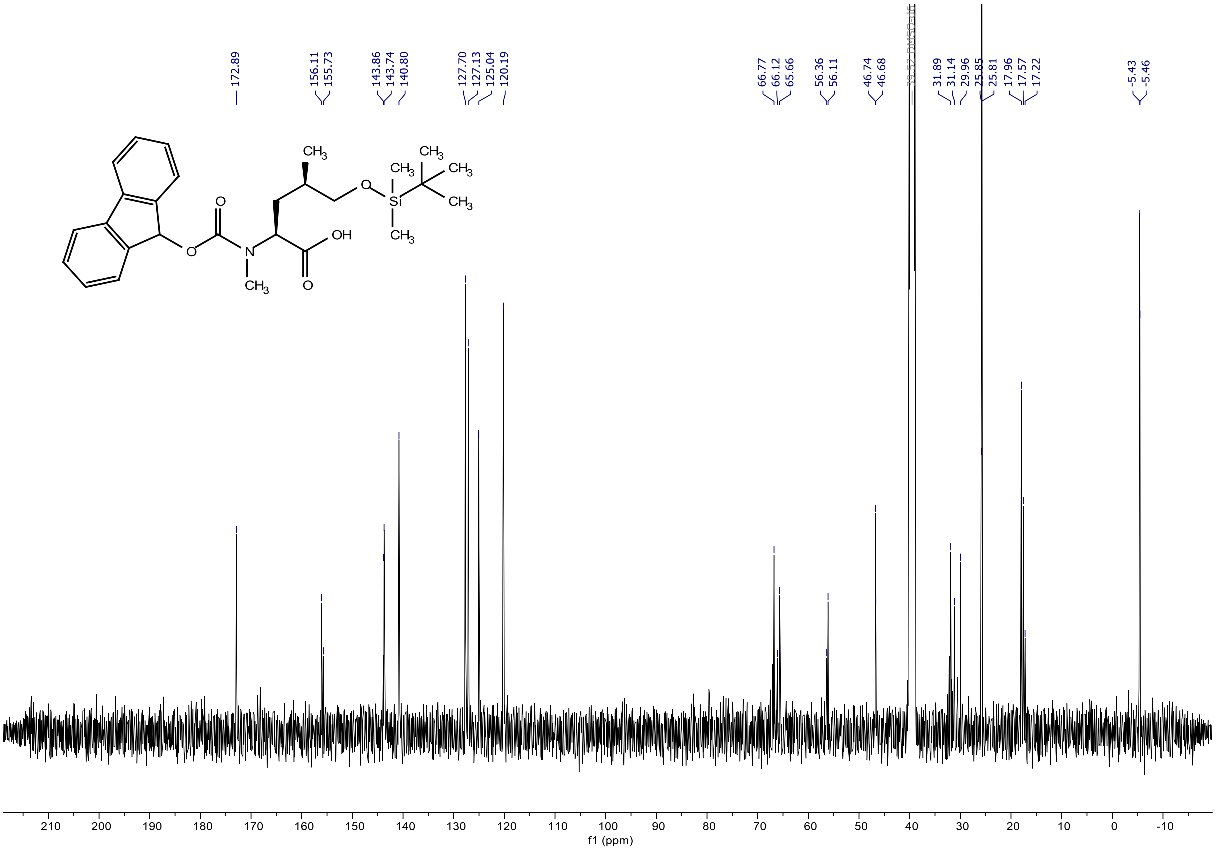

Supplementary Fig. 86. ^{1}H and ^{13}C NMR spectra of compound **SI-22**

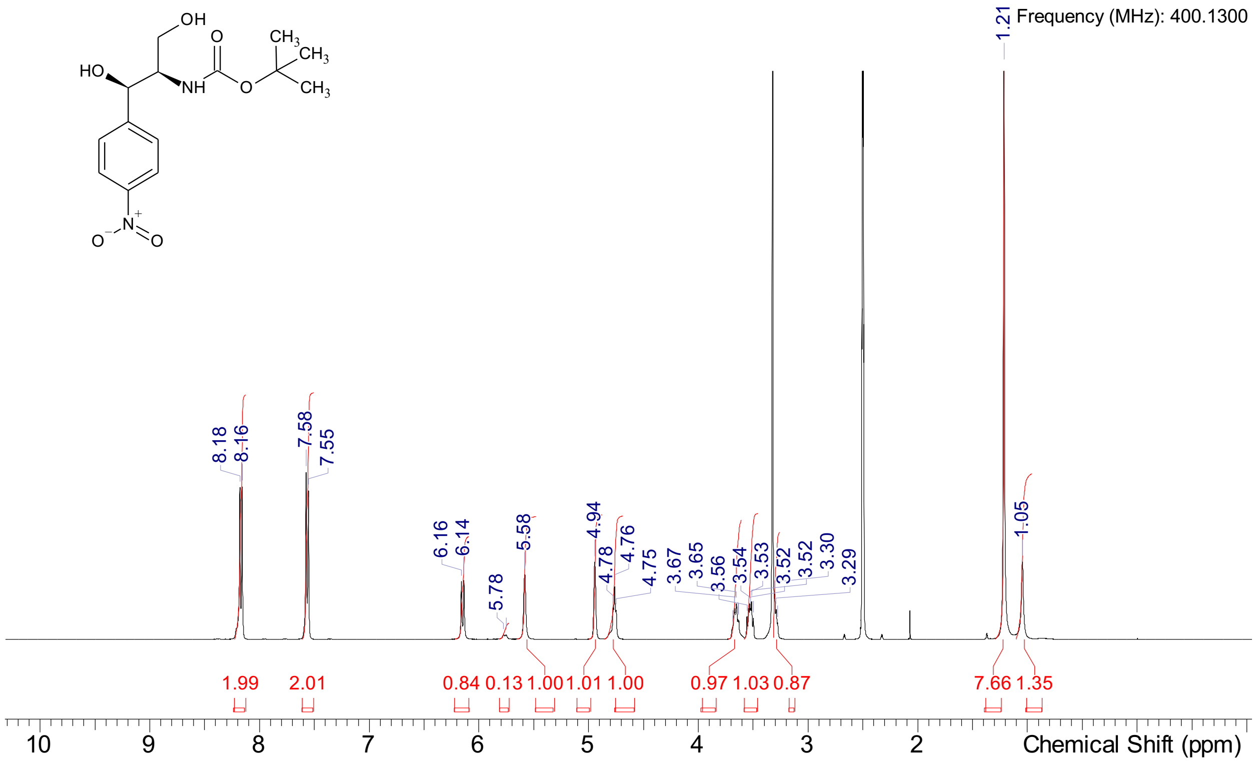

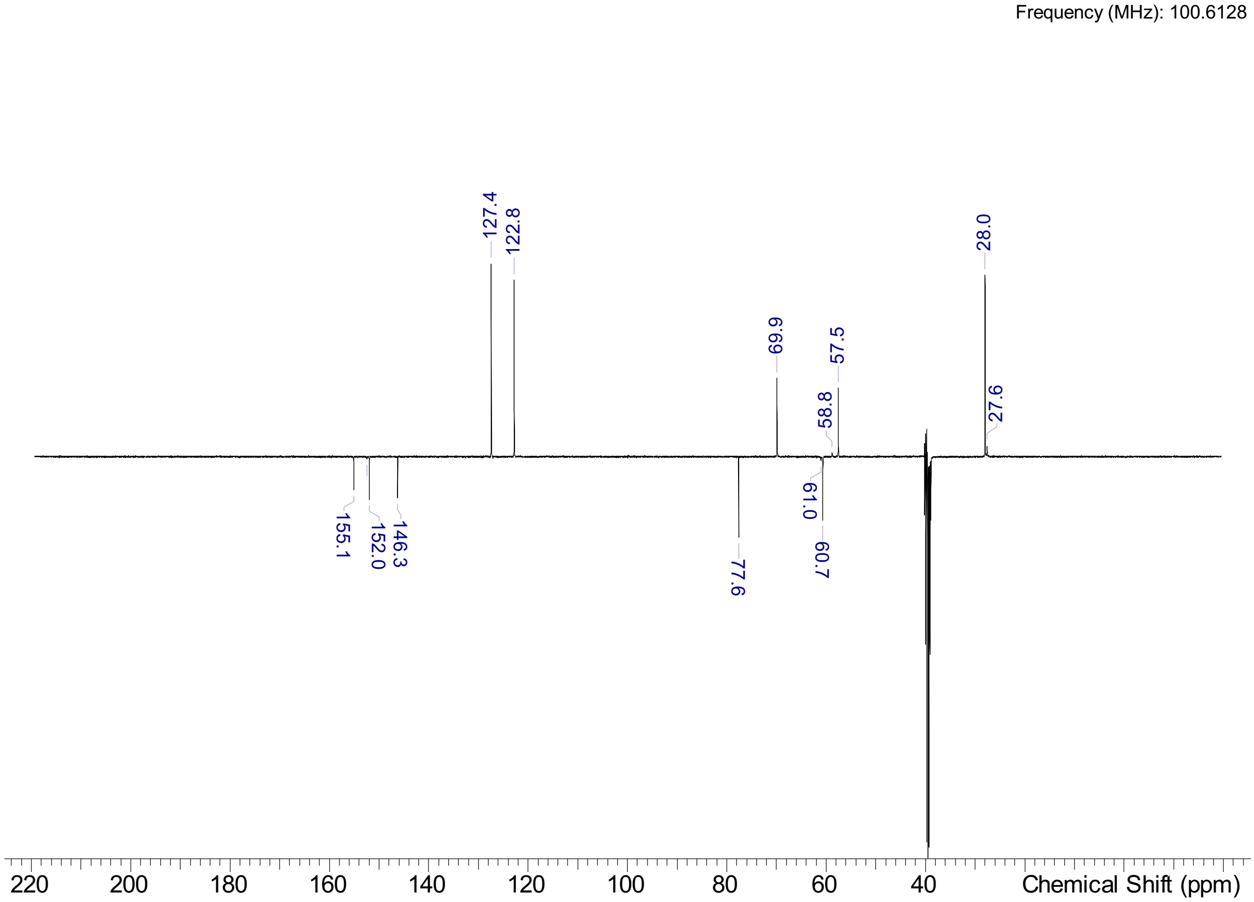

Supplementary Fig. 87. ^{1}H and ^{13}C NMR spectra of compound **SI-23**

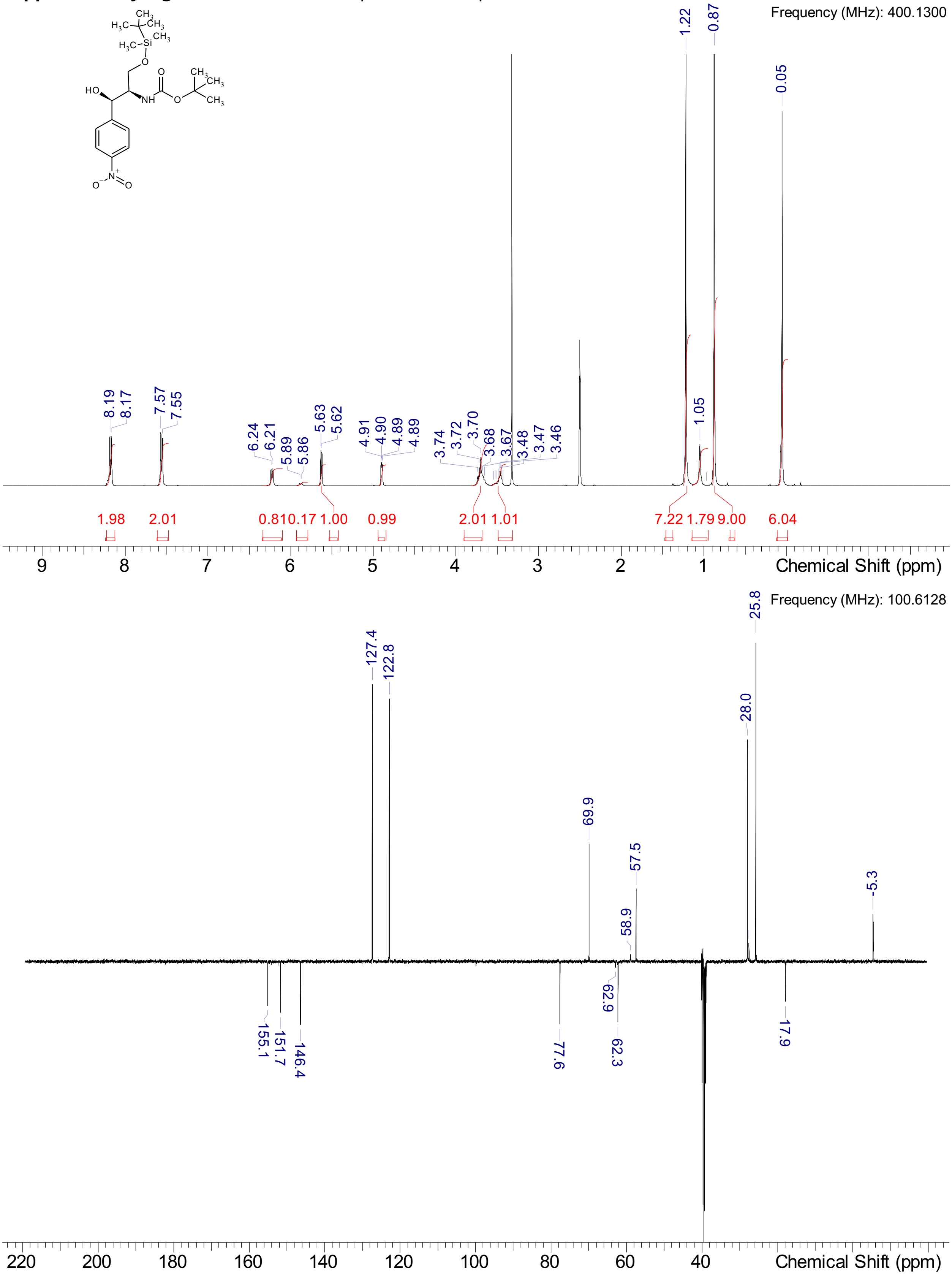

Supplementary Fig. 88. ^{1}H and ^{13}C NMR spectra of compound **SI-24**

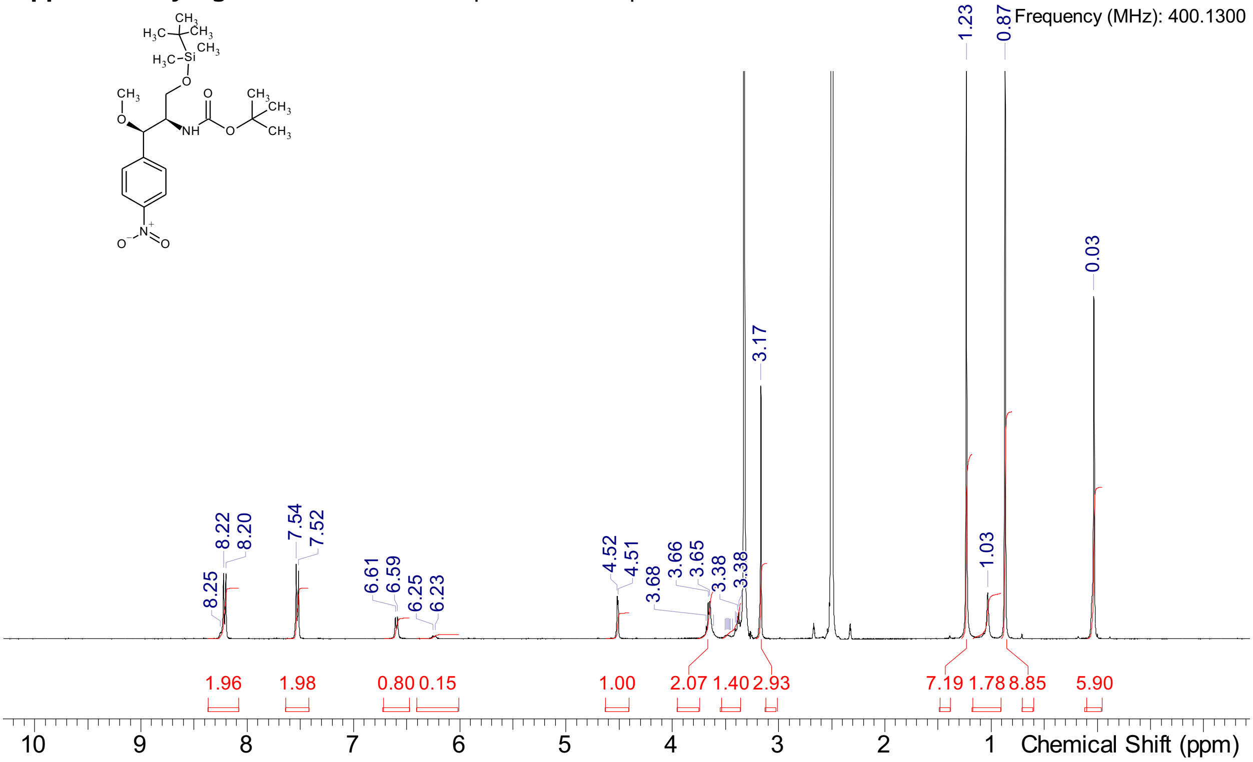

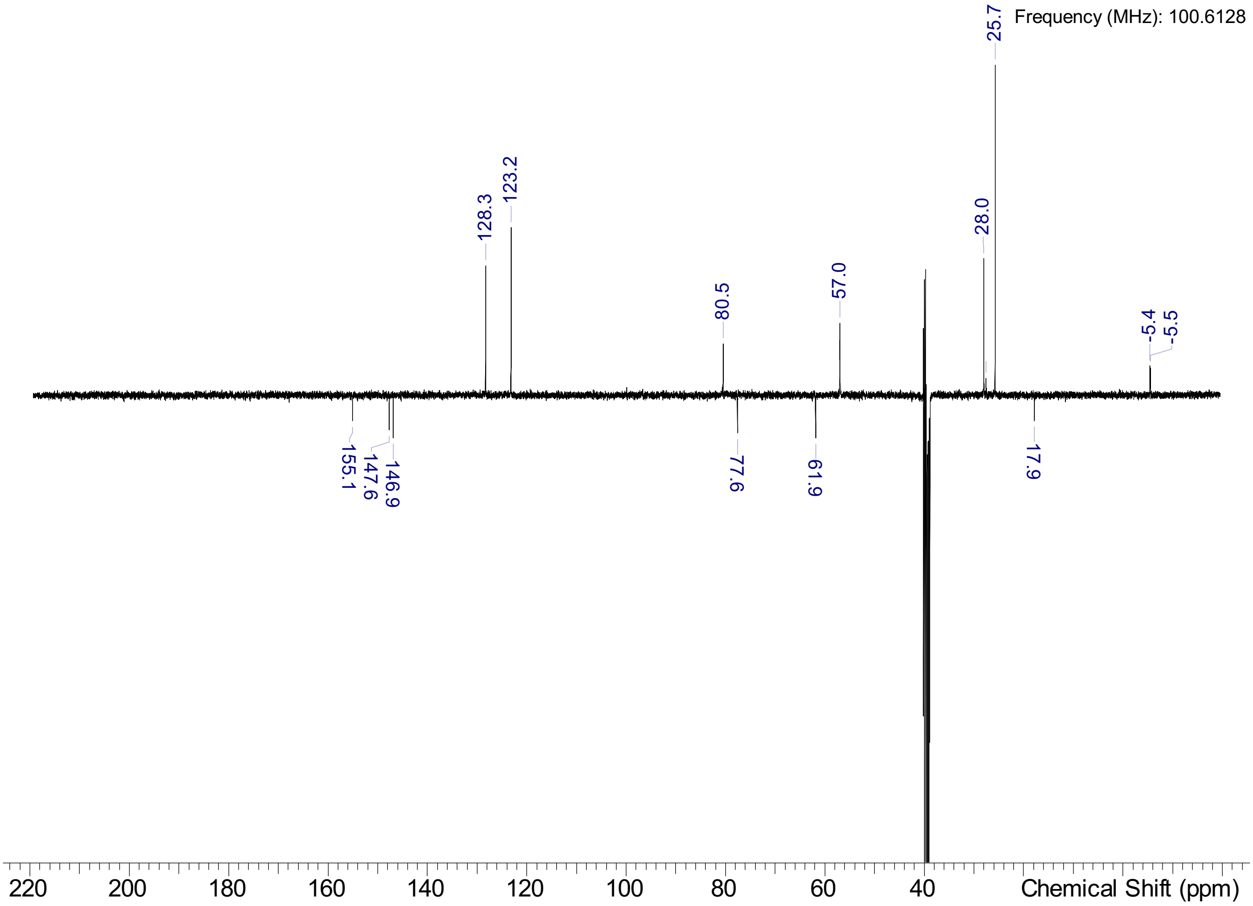

Supplementary Fig. 89. ^{1}H and ^{13}C NMR spectra of compound **SI-25**

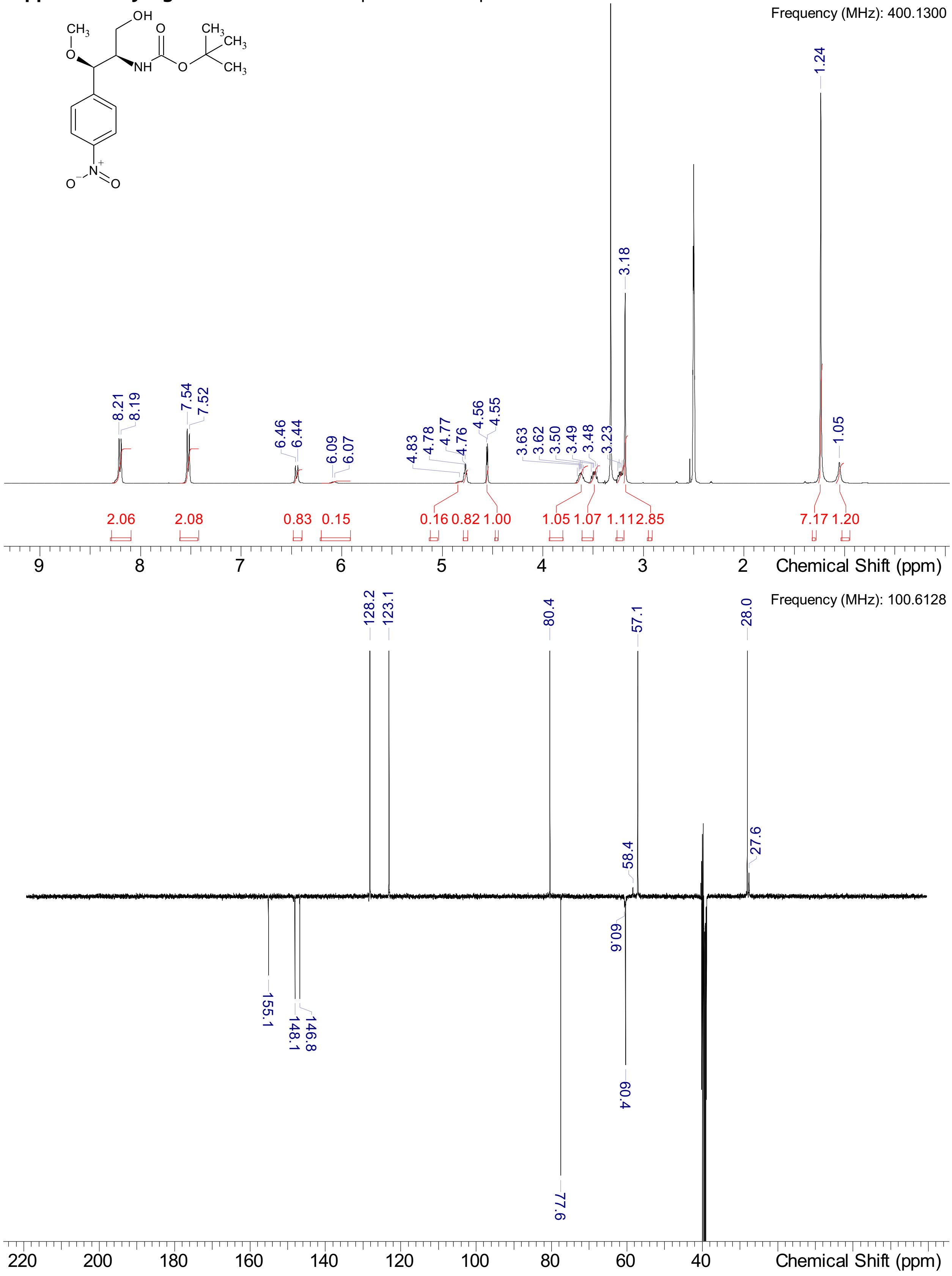

Supplementary Fig. 90. ^{1}H and ^{13}C NMR spectra of compound **SI-26**

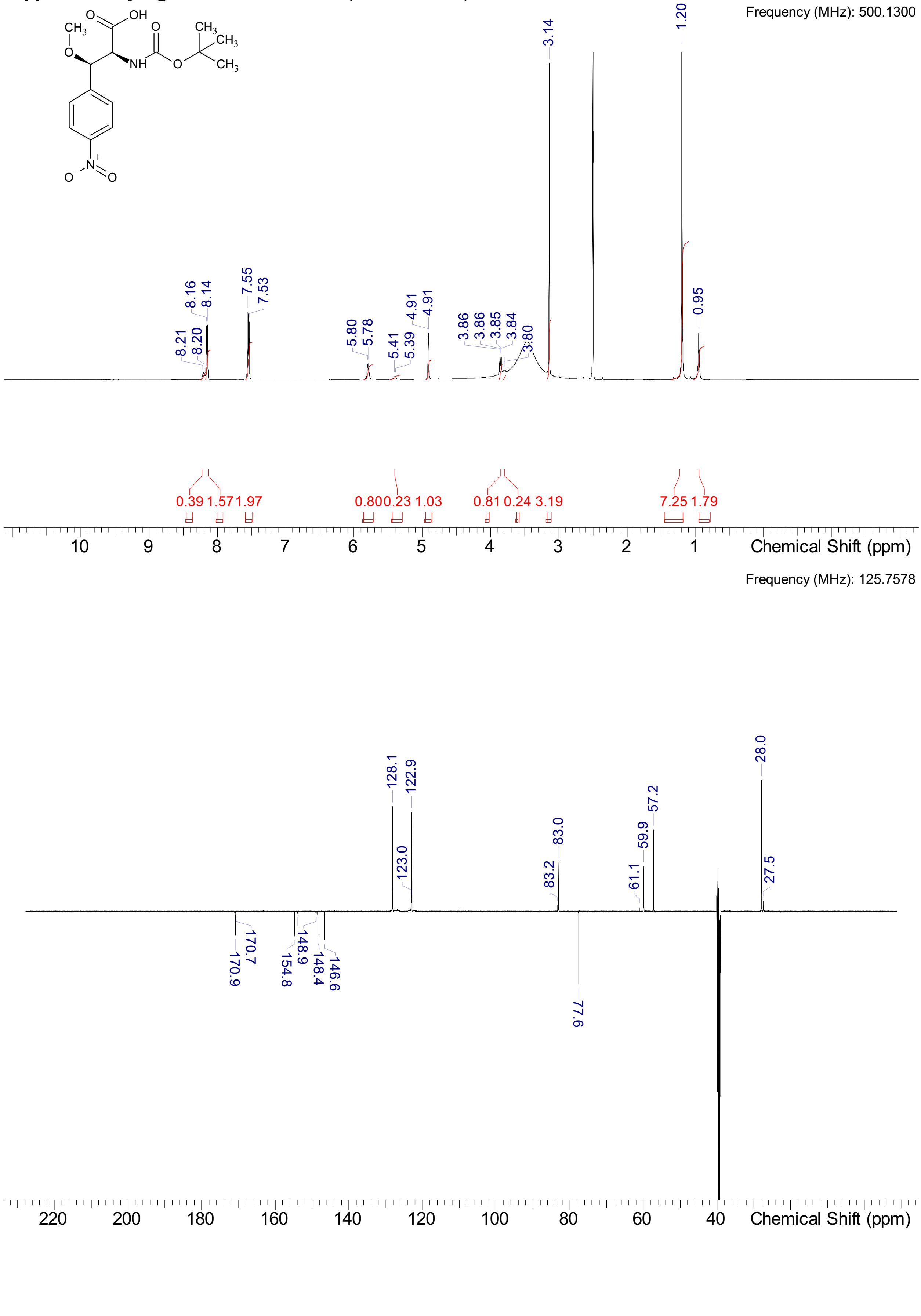

Supplementary Fig. 91. ^{1}H and ^{13}C NMR spectra of compound **SI-28**

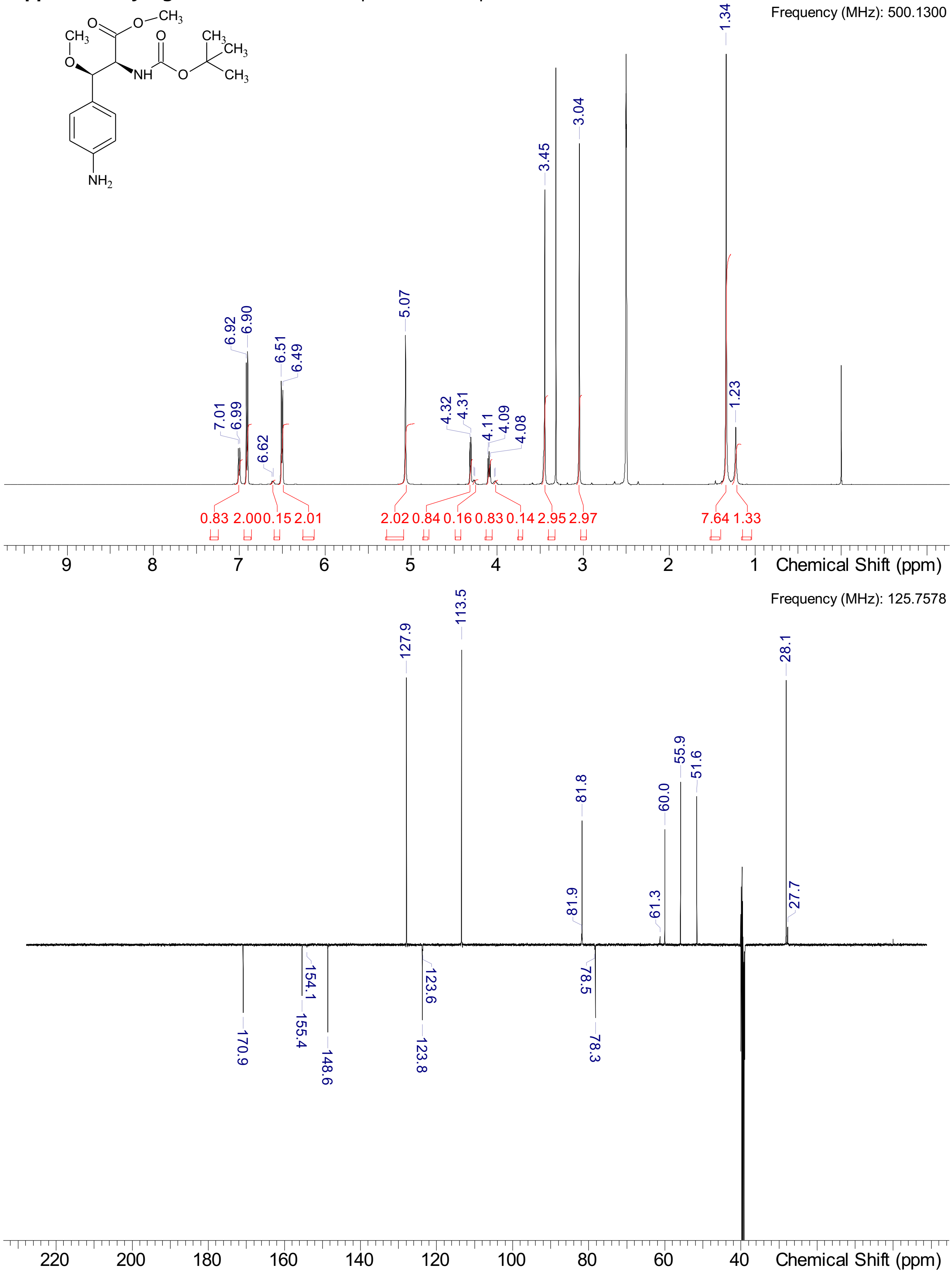

Supplementary Fig. 92. ^{1}H and ^{13}C NMR spectra of compound **SI-29**

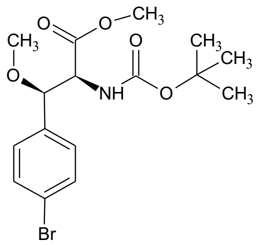

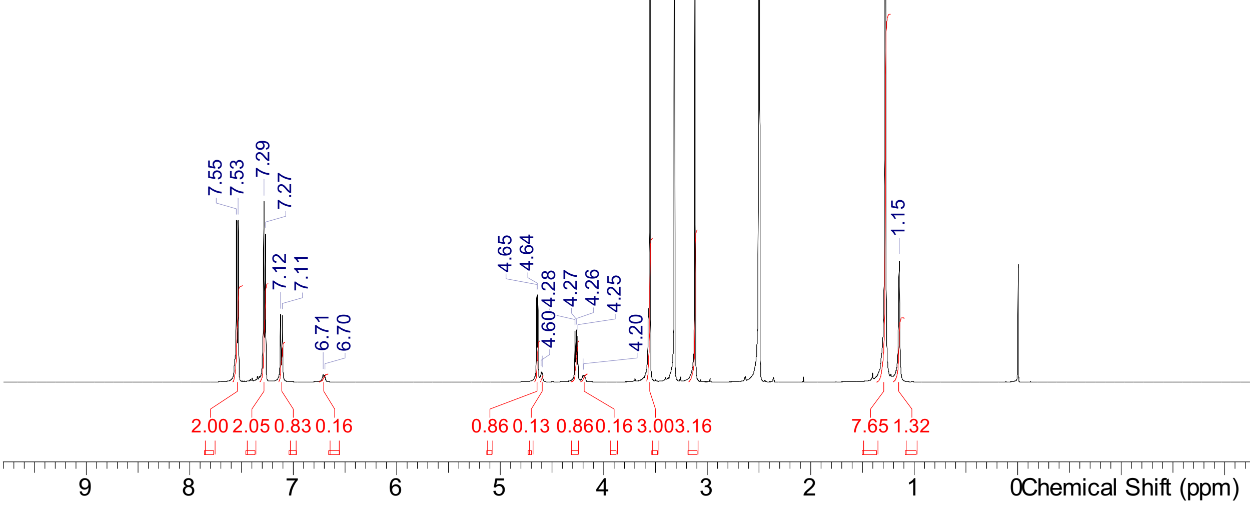

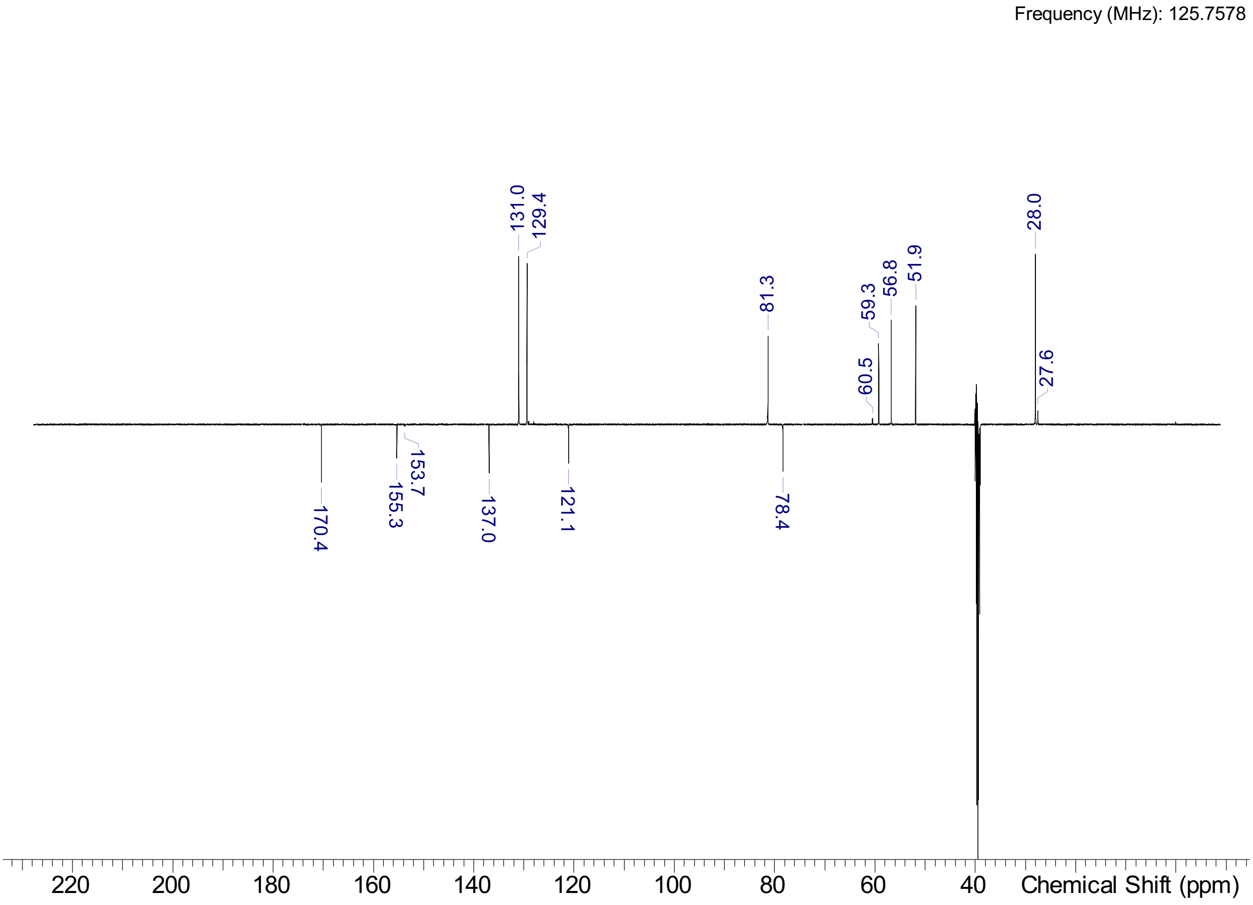

Supplementary Fig. 93. ^{1}H and ^{13}C NMR spectra of compound **SI-30**

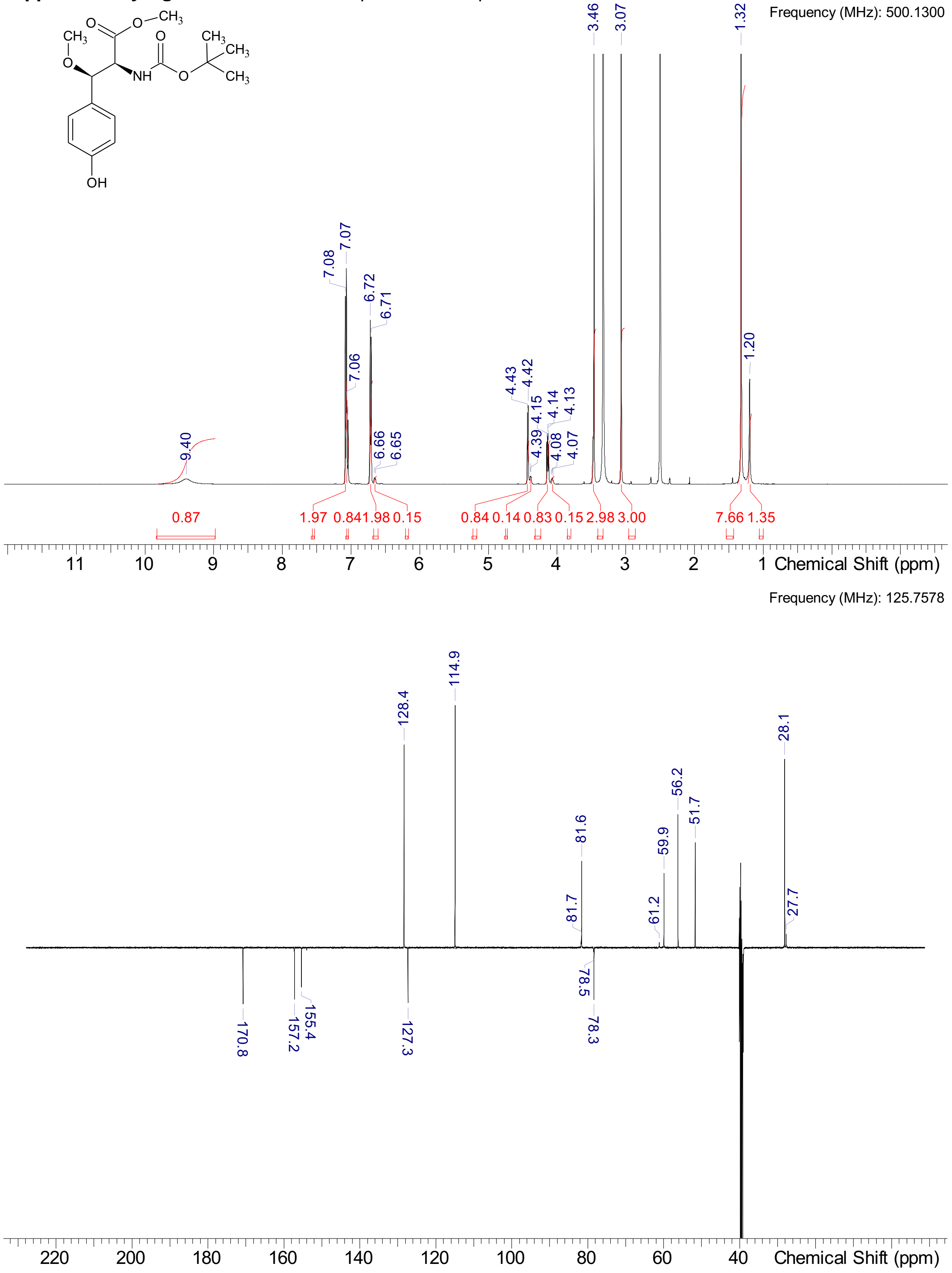

Supplementary Fig. 94. ^{1}H and ^{13}C NMR spectra of compound **SI-31**

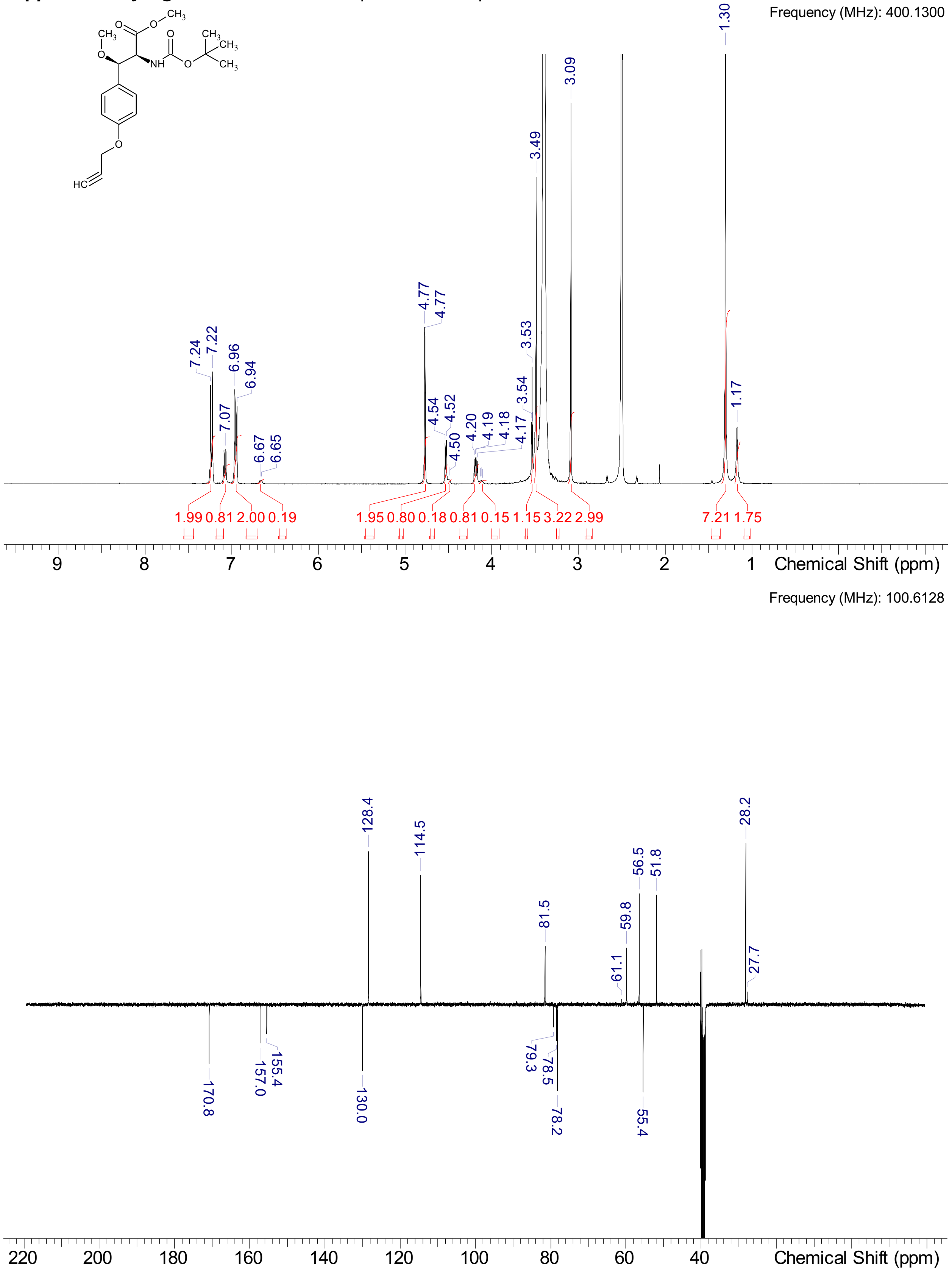

Supplementary Fig. 95. ^{1}H and ^{13}C NMR spectra of compound **SI-32**

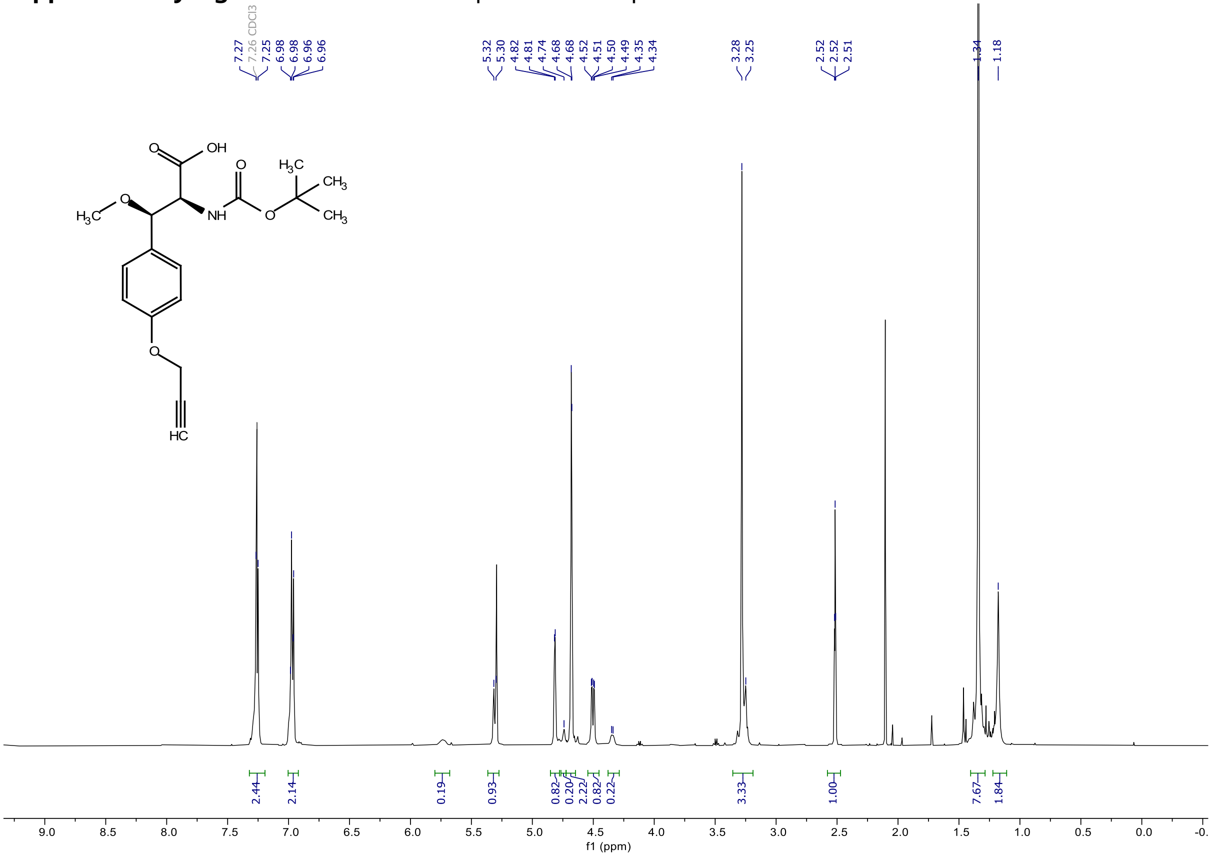

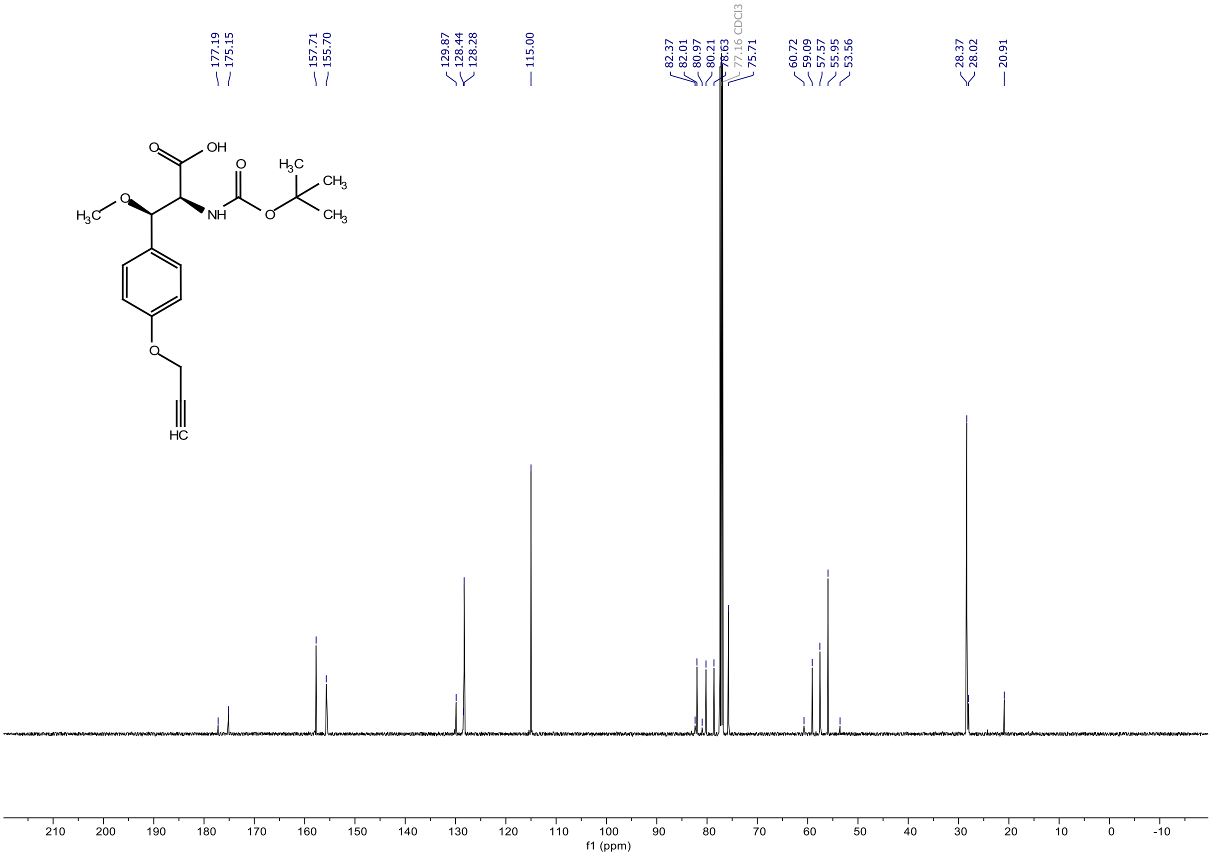

Supplementary Fig. 96. ^{1}H NMR spectrum of compound **SI-33**

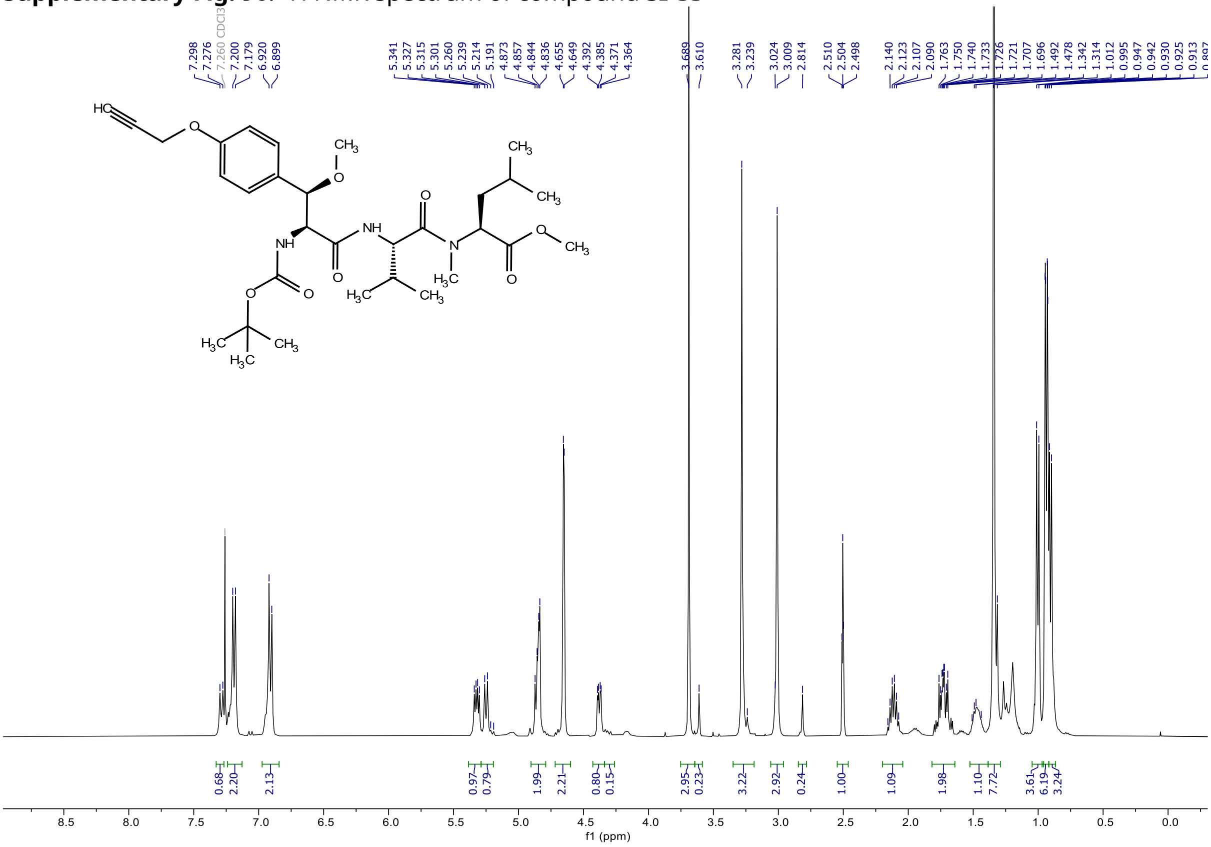

Supplementary Fig. 97. ^{13}C NMR spectrum of compound **SI-33**

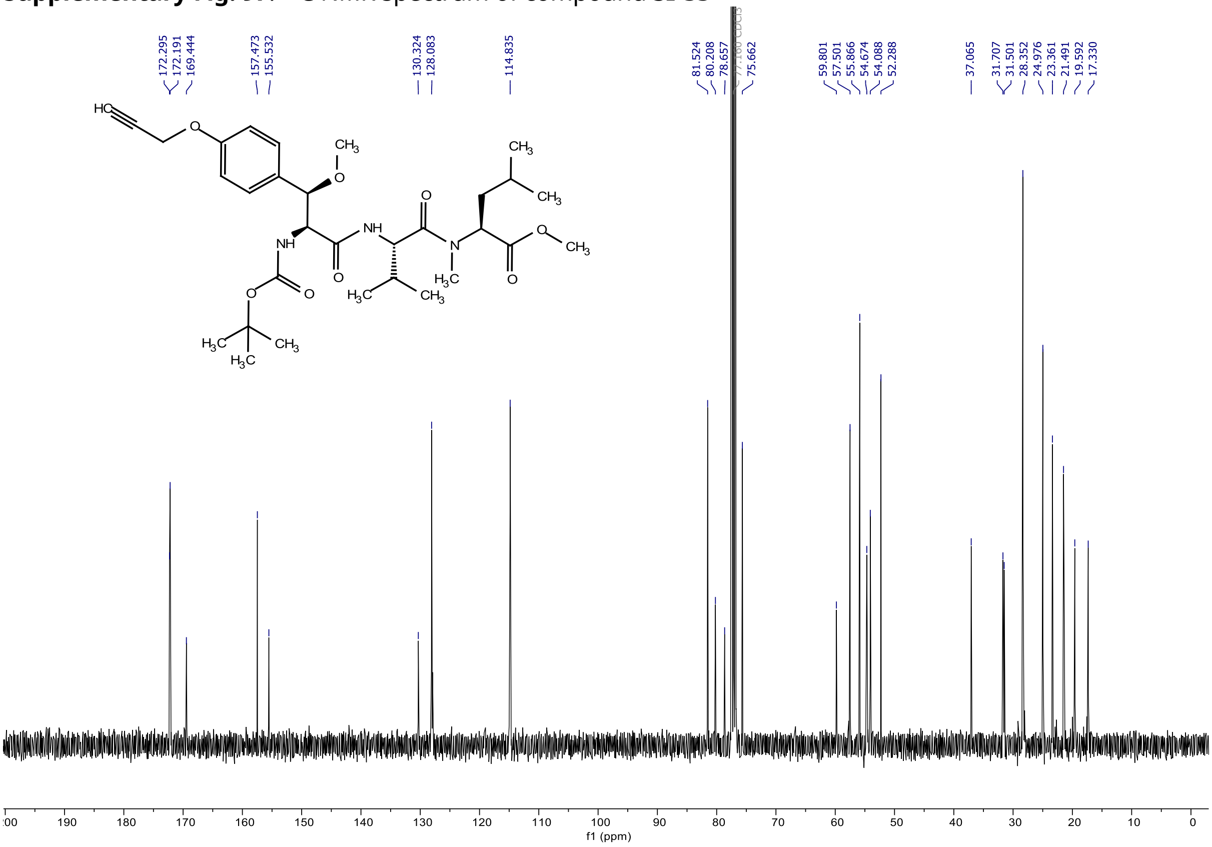

Supplementary Fig. 98. ^{1}H NMR spectrum of compound **SI-34**

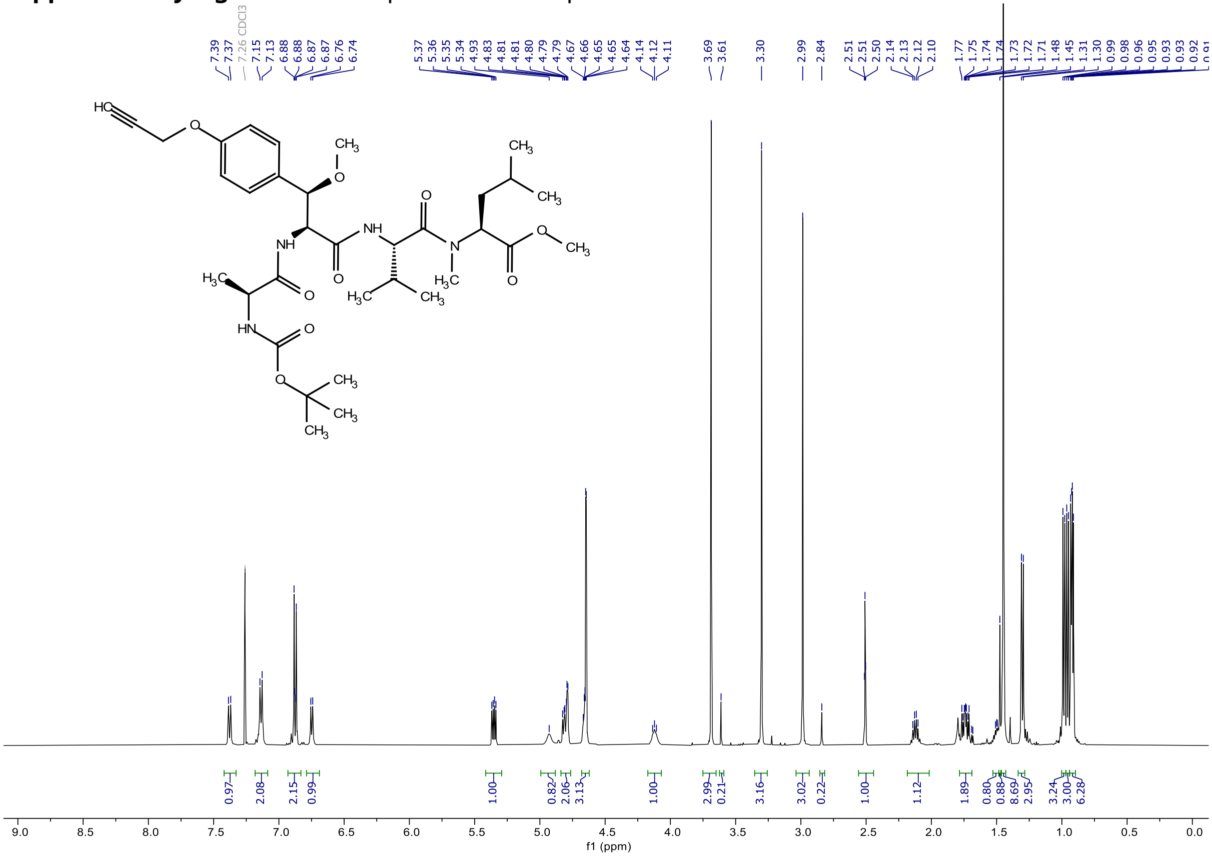

Supplementary Fig. 99. ^{13}C NMR spectrum of compound **SI-34**

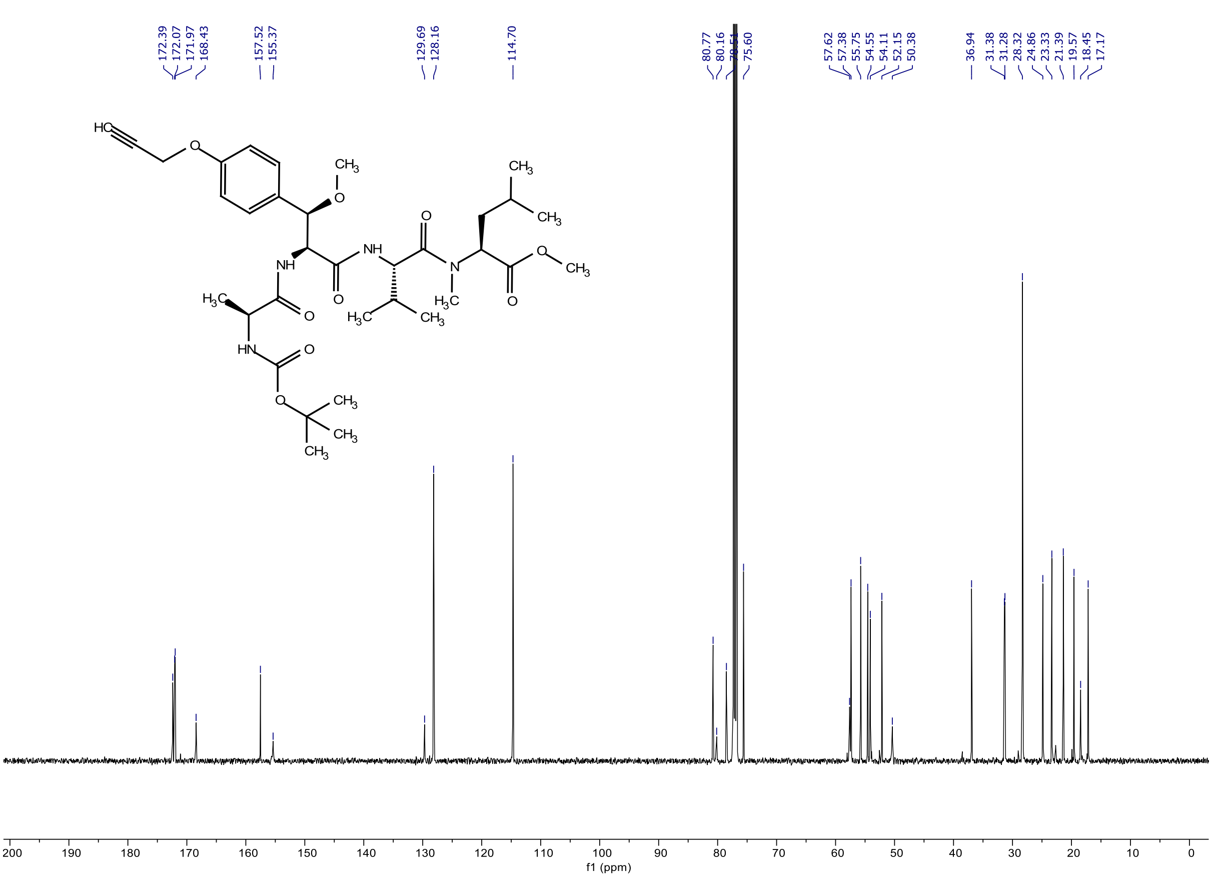

Supplementary Fig. 100. ^{1}H NMR spectrum of compound **SI-35**

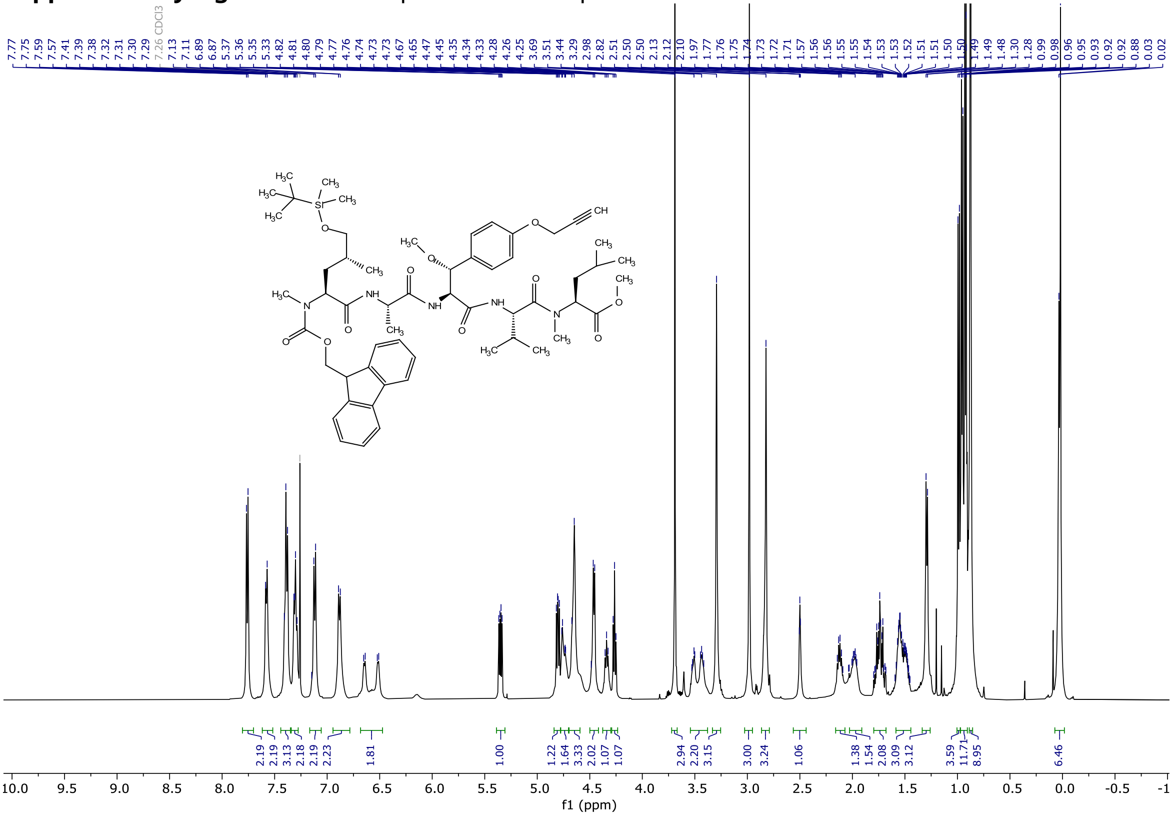

Supplementary Fig. 101. ^{13}C NMR spectrum of compound **SI-35**

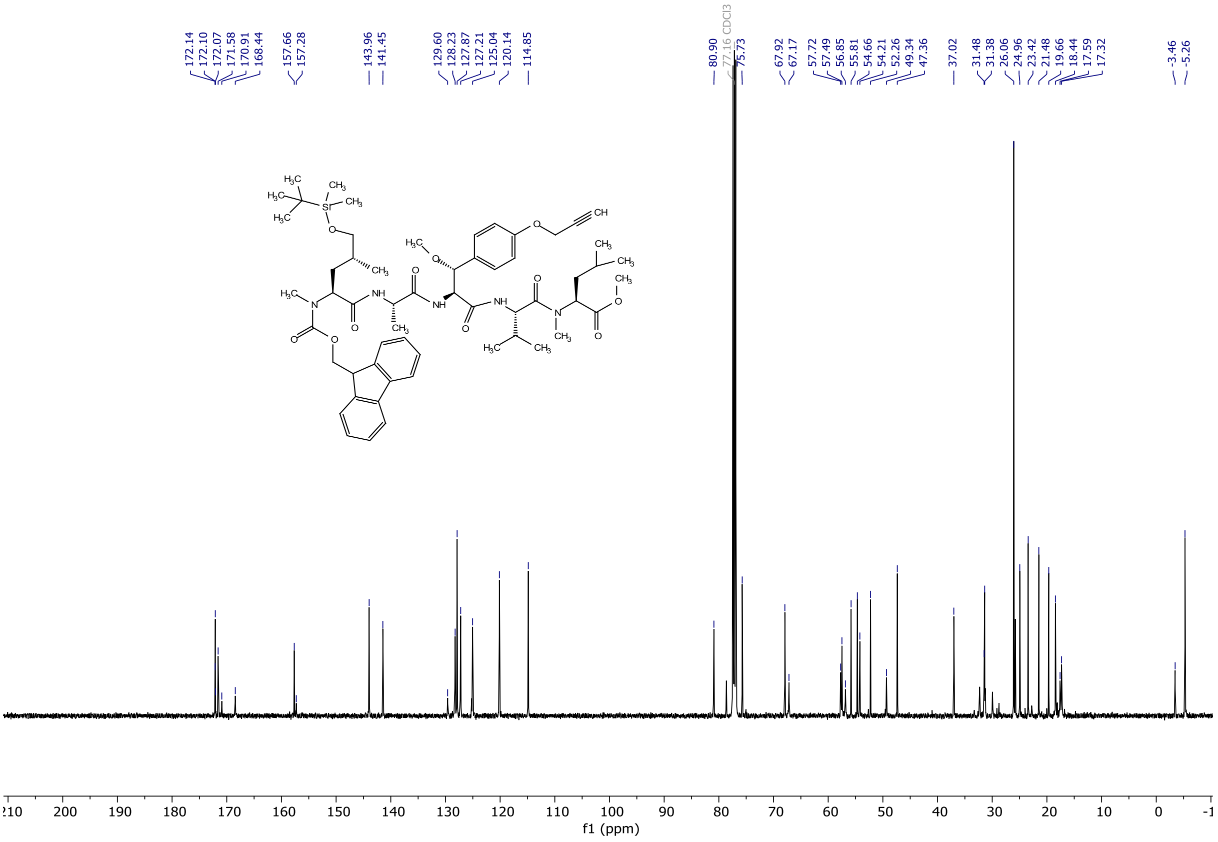

Supplementary Fig. 102. ^{1}H NMR spectrum of compound **SI-36**

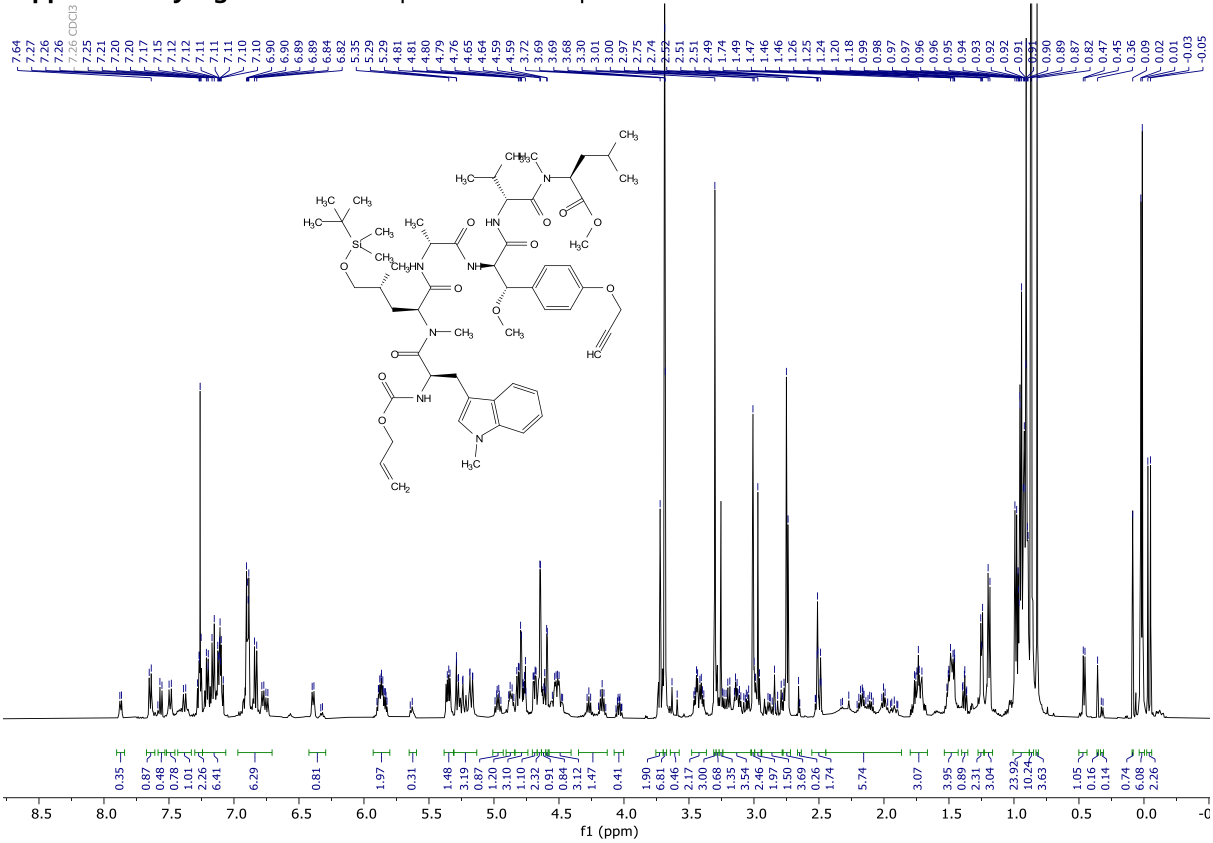

Supplementary Fig. 103. ^{13}C NMR spectrum of compound **SI-36**

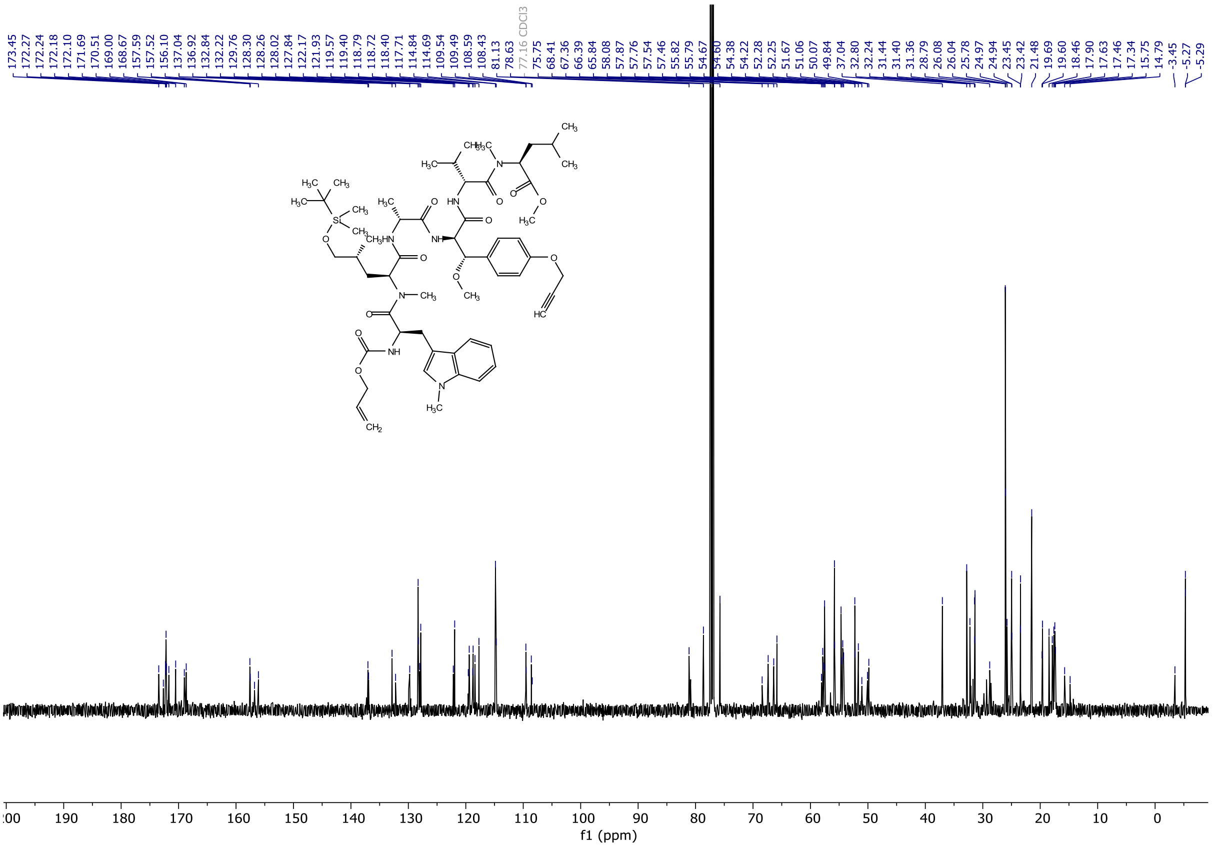

Supplementary Fig. 104. ^{1}H NMR spectrum of compound **SI-37**

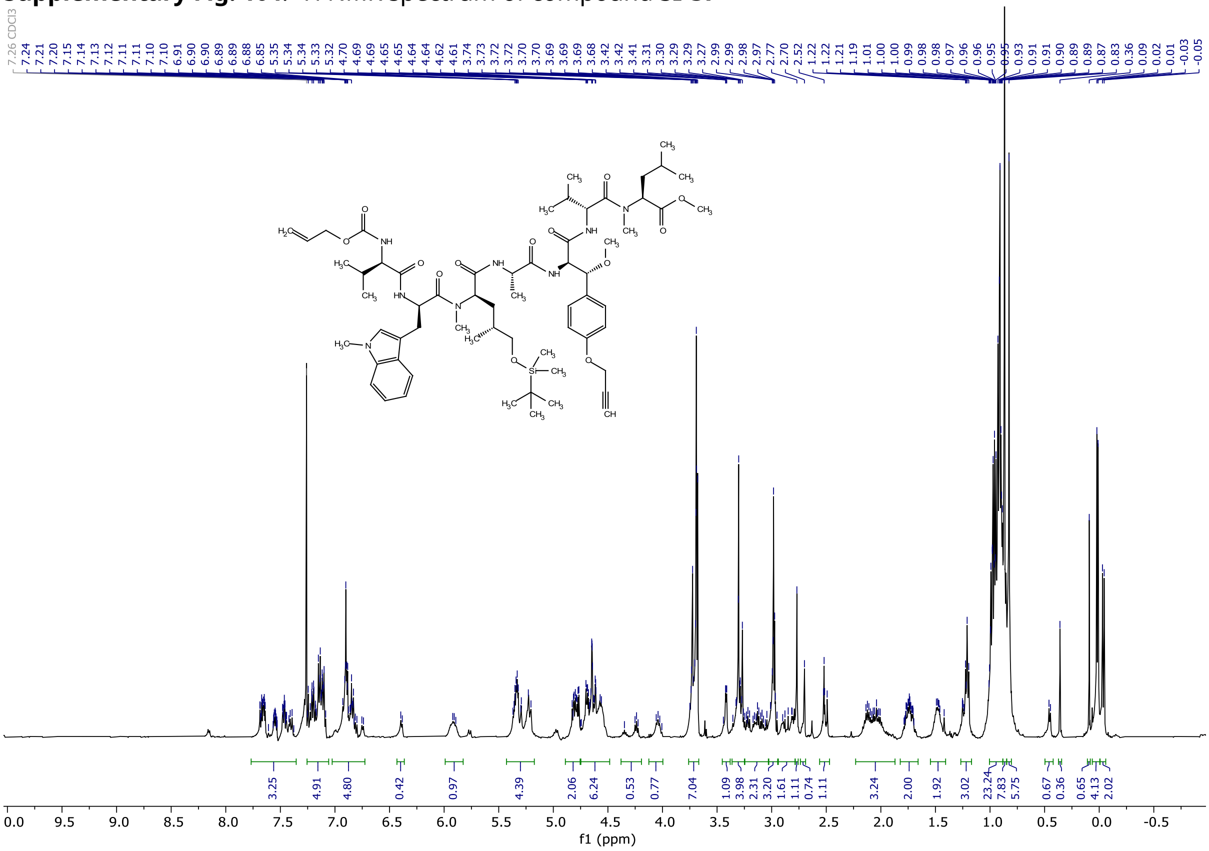

Supplementary Fig. 105. ^{13}C NMR spectrum of compound **SI-37**

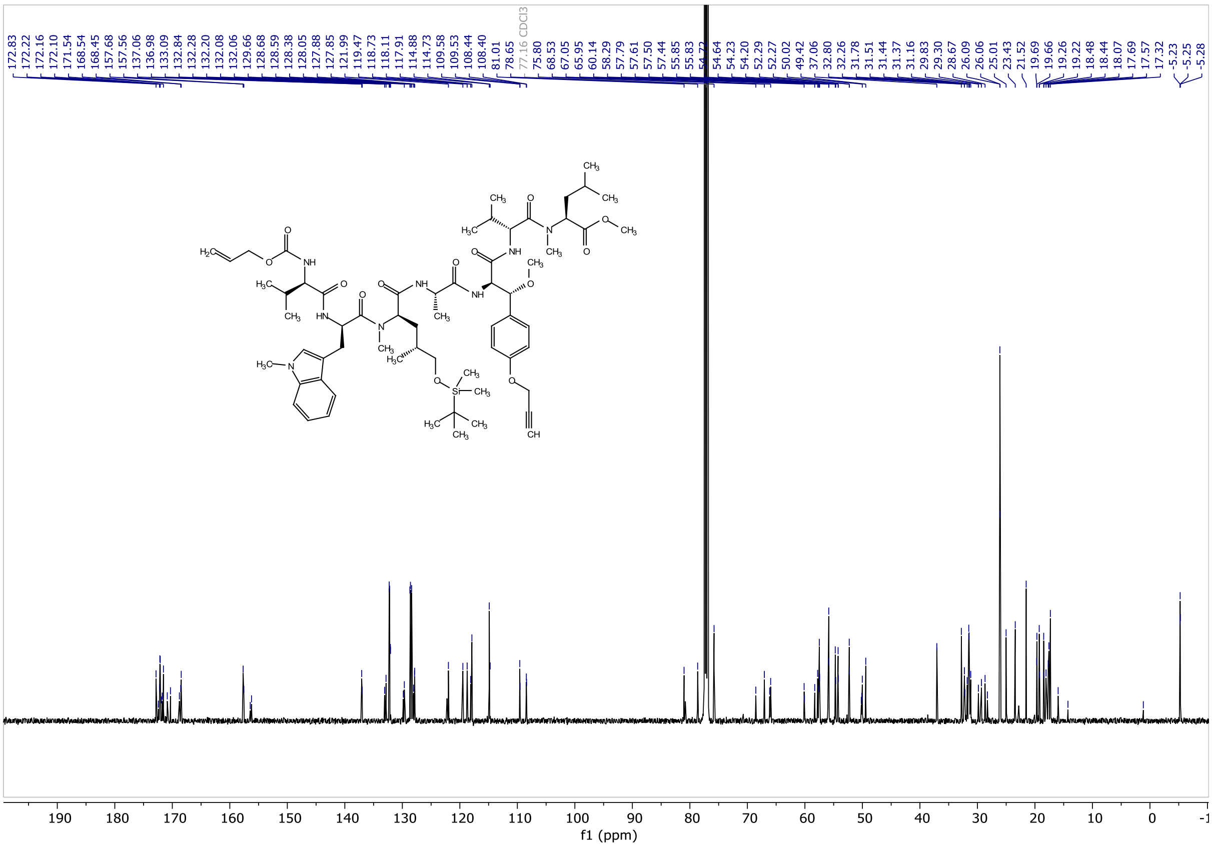

Supplementary Fig. 106. ^{1}H NMR spectrum of compound **SI-38**

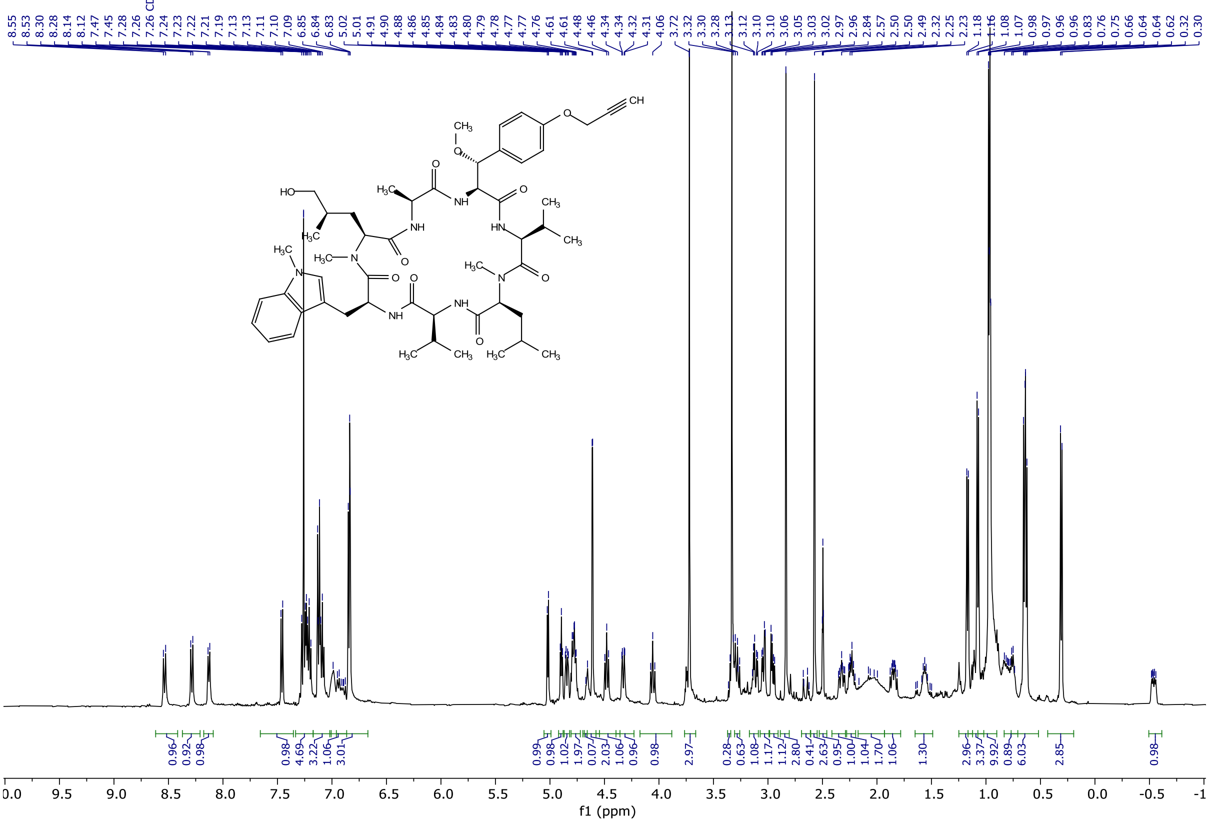

Supplementary Fig. 107. ^{13}C NMR spectrum of compound **SI-38**

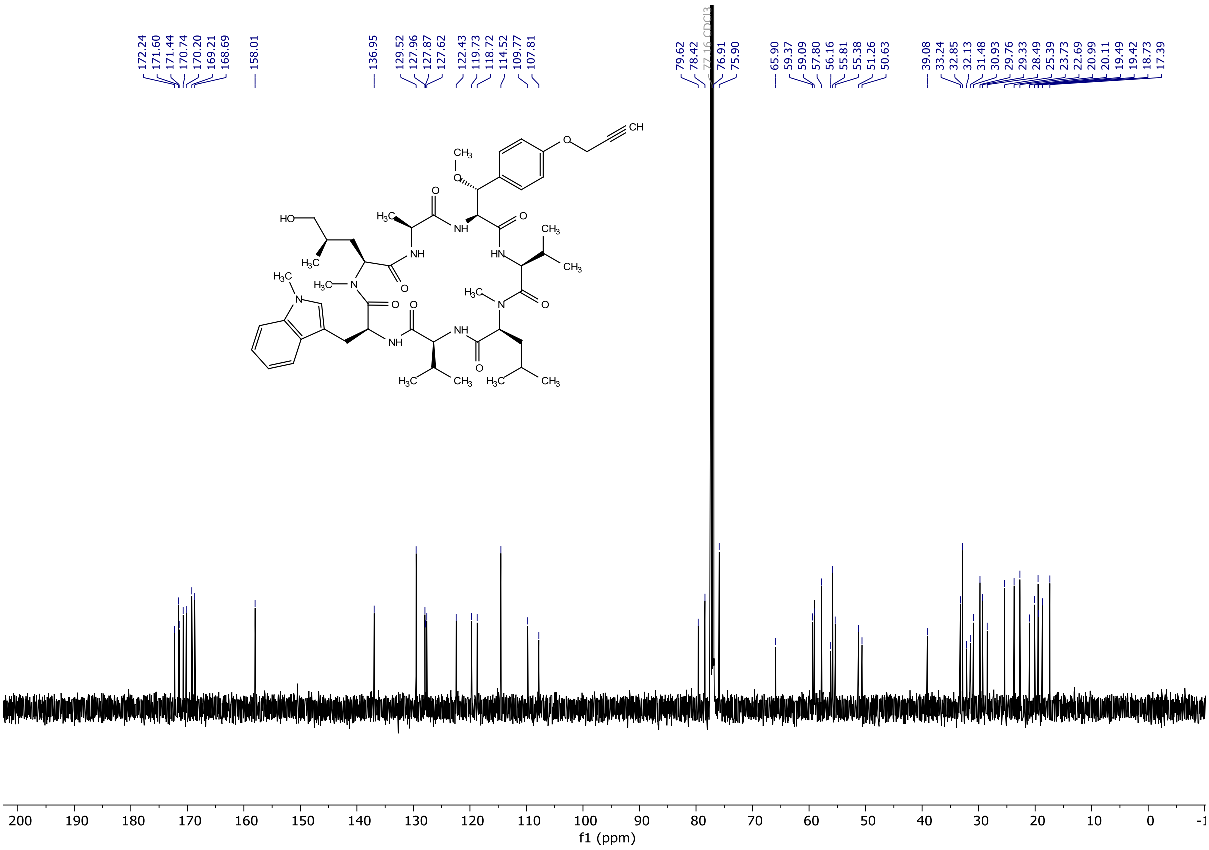

Supplementary Fig. 108. ^{1}H NMR spectrum of compound **SI-39**

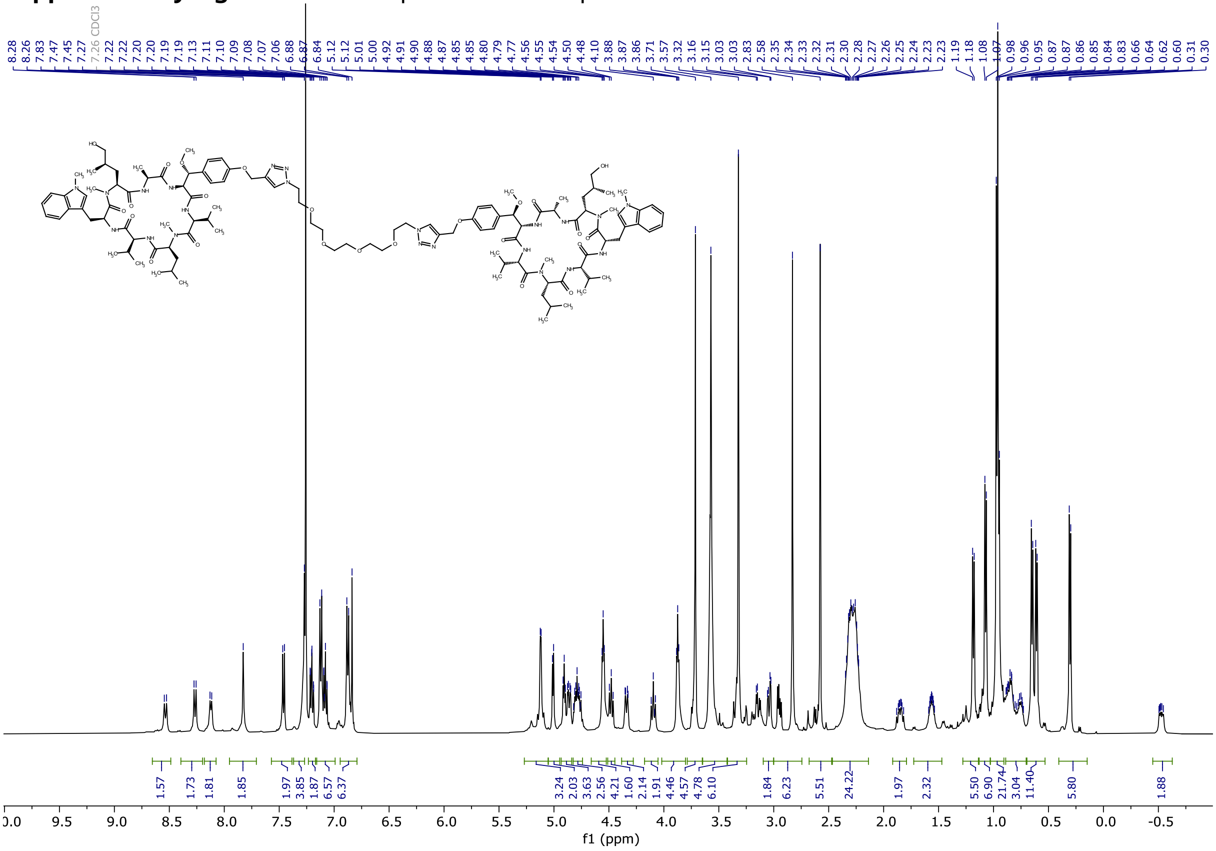

Supplementary Fig. 109. ^{13}C NMR spectrum of compound **SI-39**

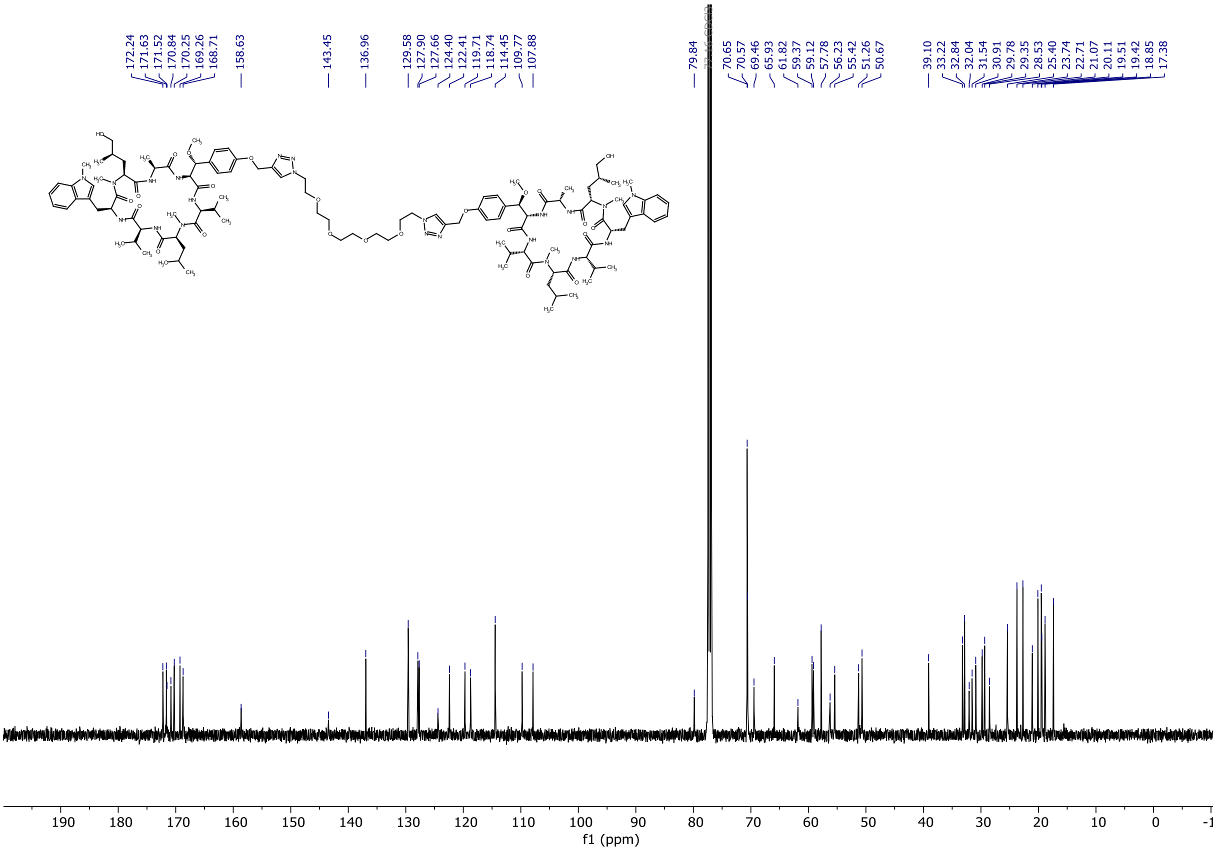

Supplementary Fig. 110. ^{1}H NMR spectrum of compound **SI-40**

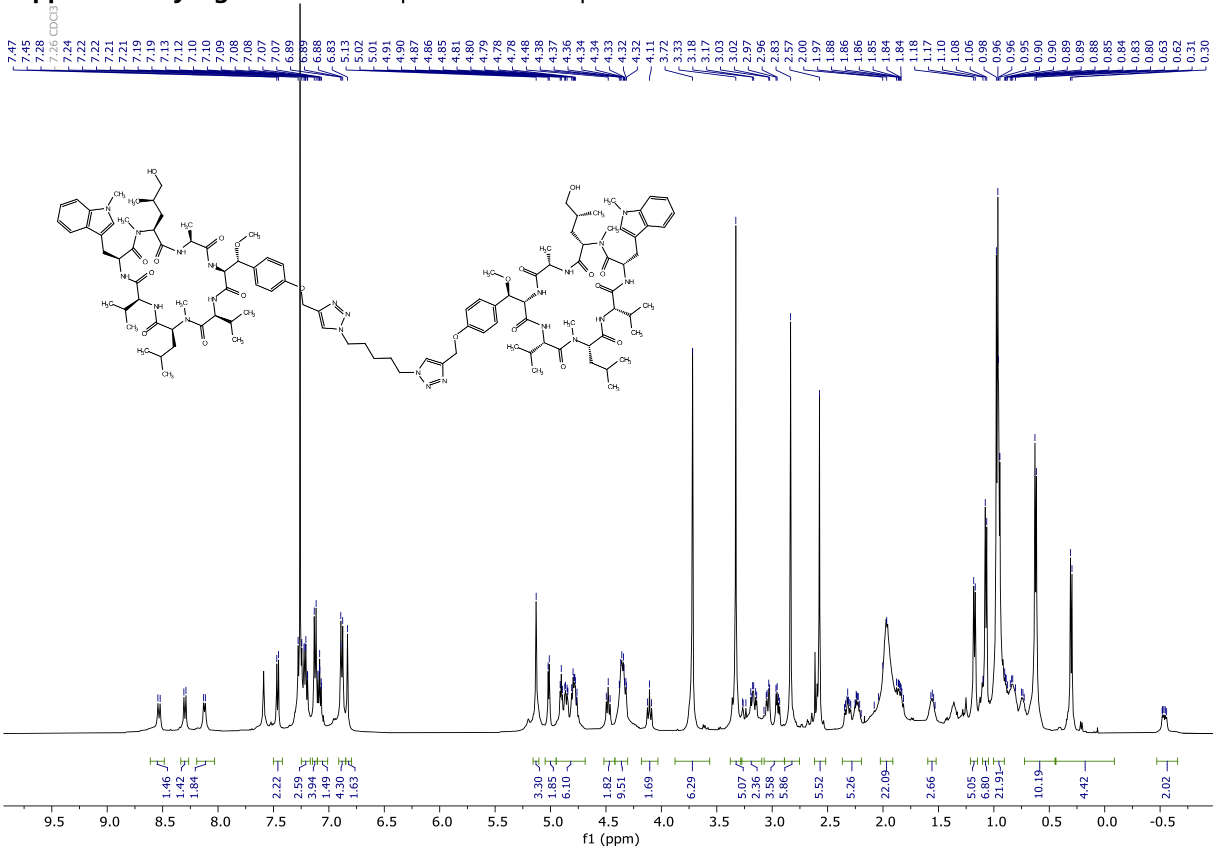

Supplementary Fig. 111. ^{13}C NMR spectrum of compound **SI-40**

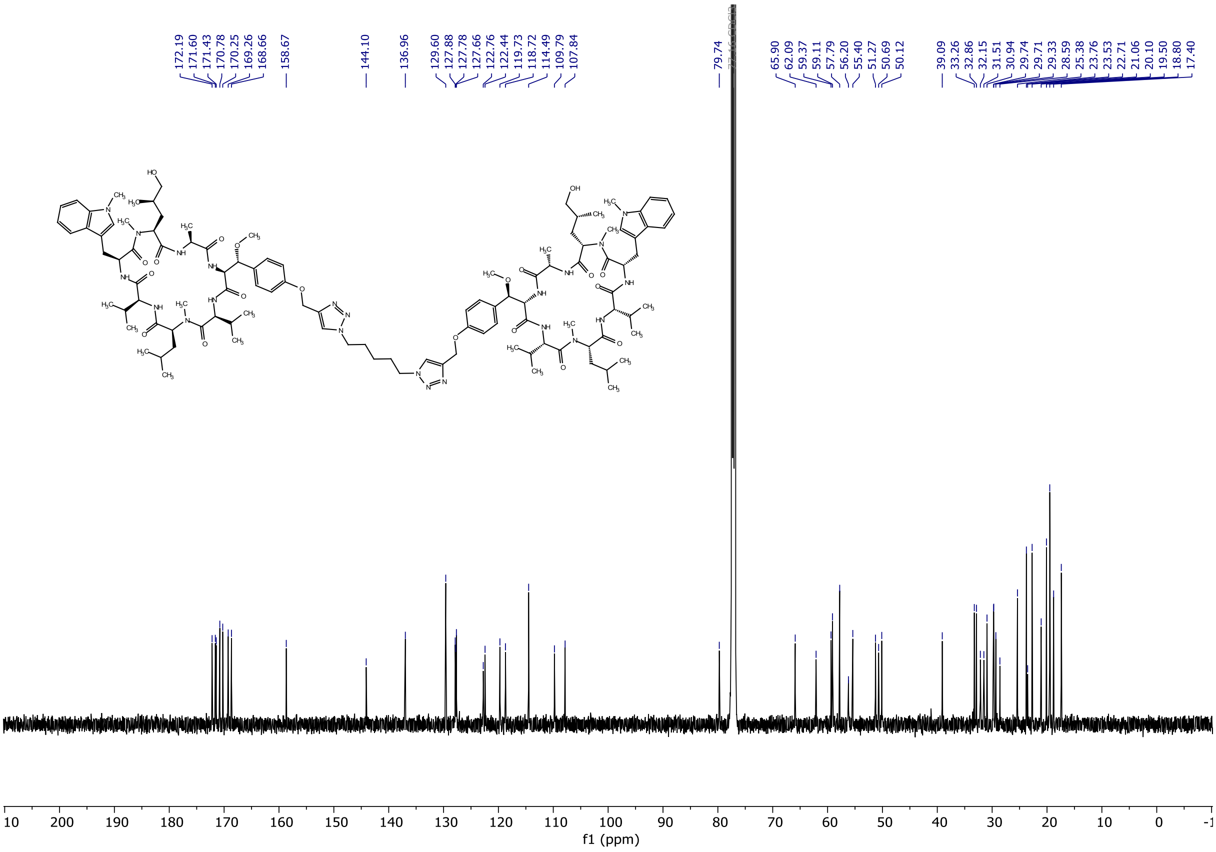

Supplementary Fig. 112. ^{1}H NMR spectrum of compound **SI-41**

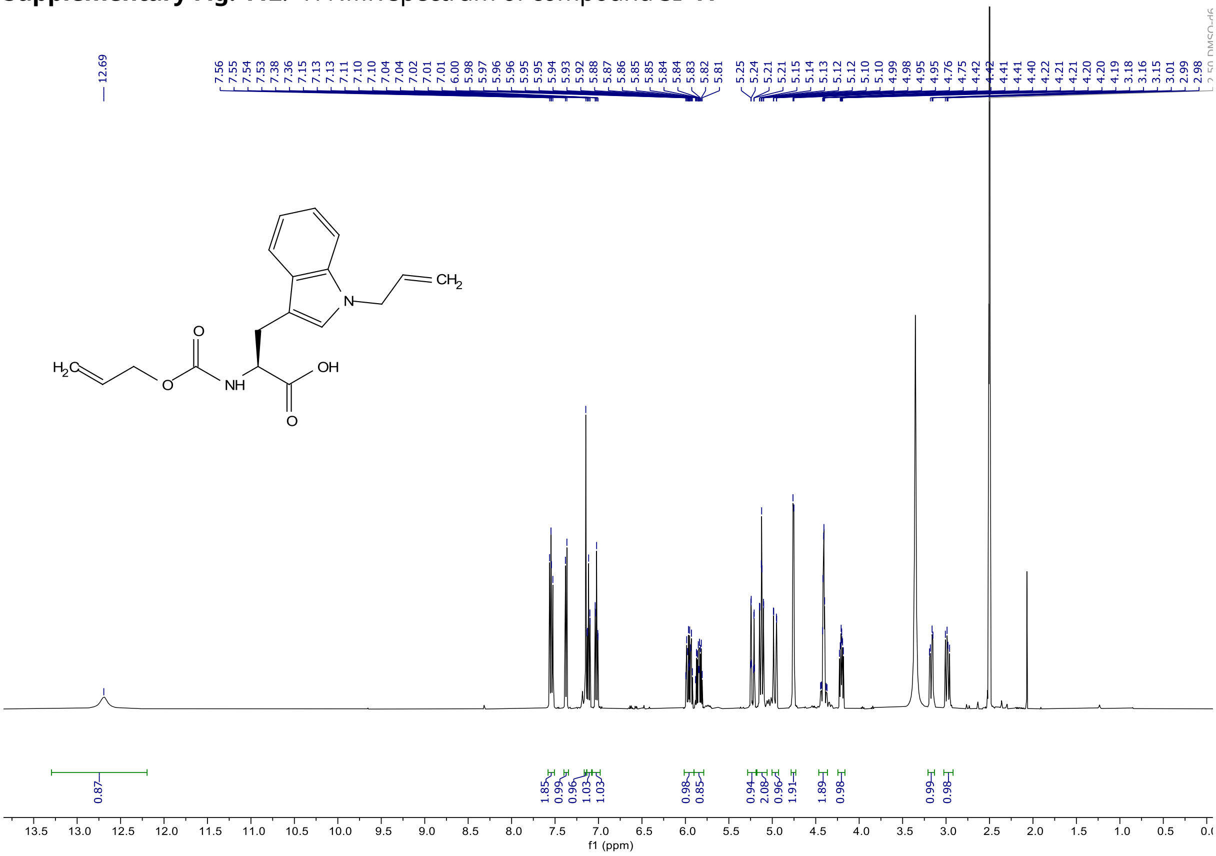

Supplementary Fig. 113. ^{13}C NMR spectrum of compound **SI-41**

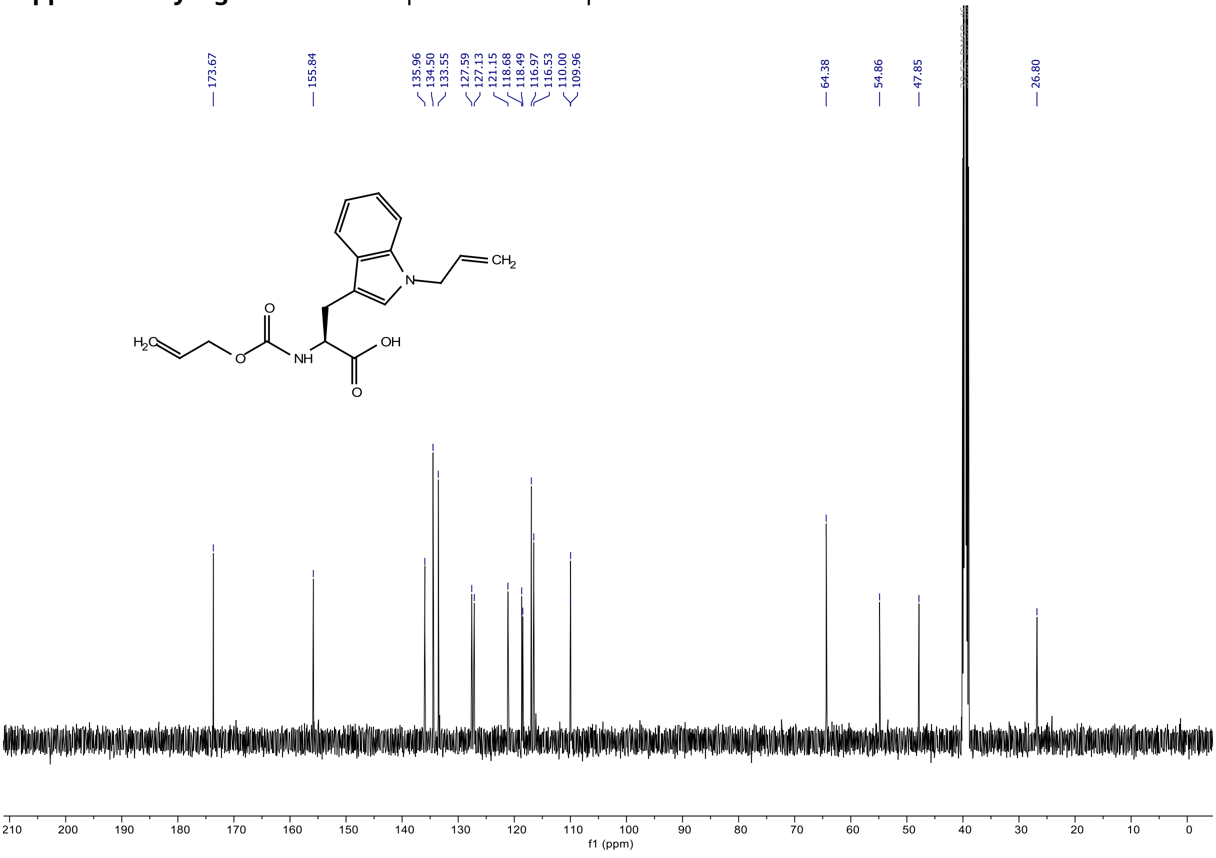

Supplementary Fig. 114. ^{1}H NMR spectrum of compound **SI-42**

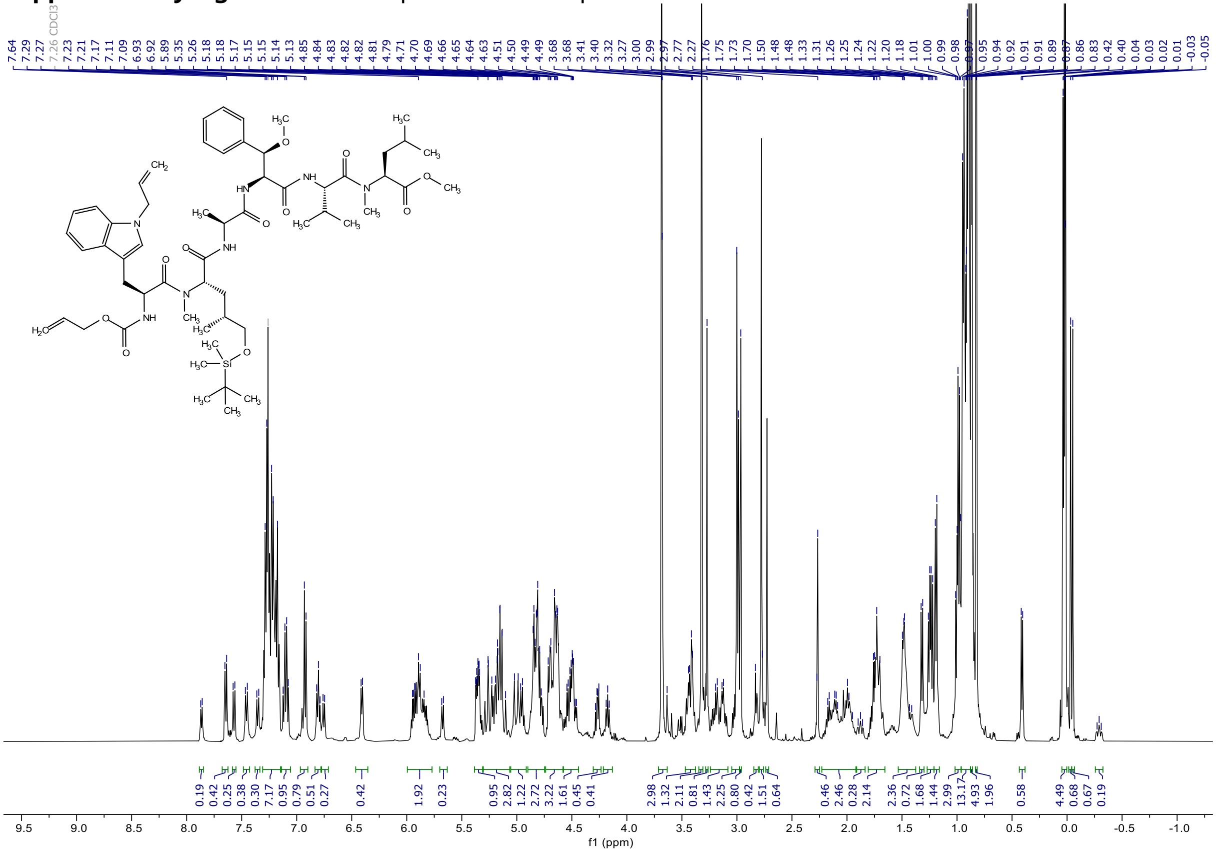

Supplementary Fig. 114. ^{13}C NMR spectrum of compound **SI-42**

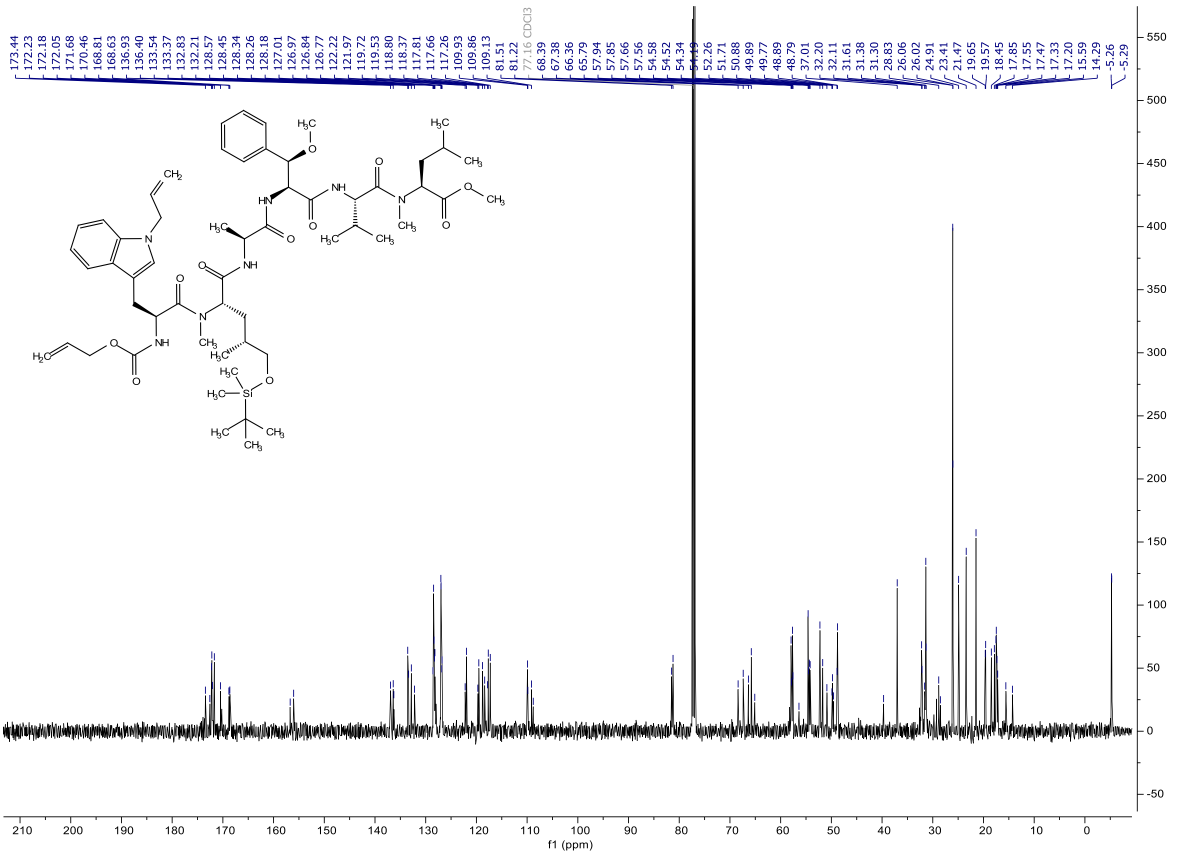

Supplementary Fig. 115. ^{1}H NMR spectrum of compound **SI-43**

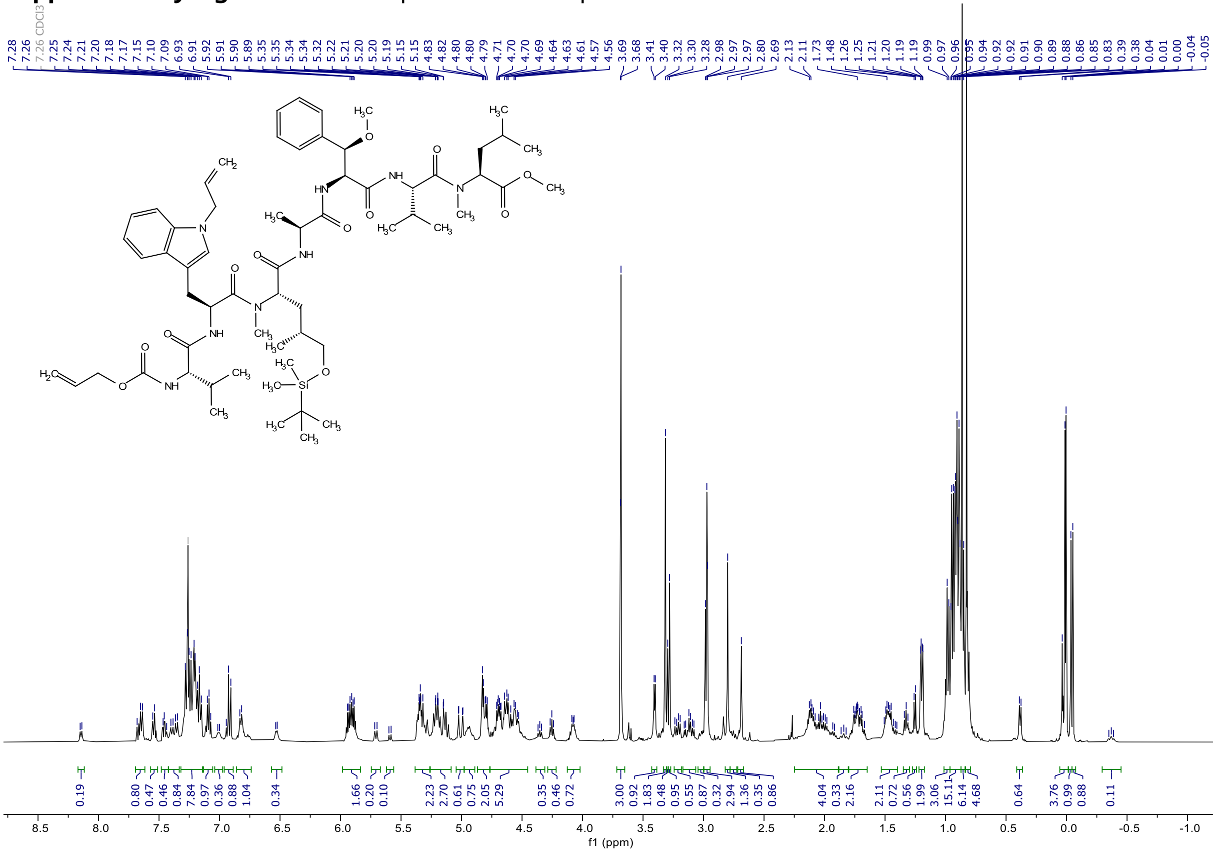

Supplementary Fig. 116. ^{13}C NMR spectrum of compound **SI-43**

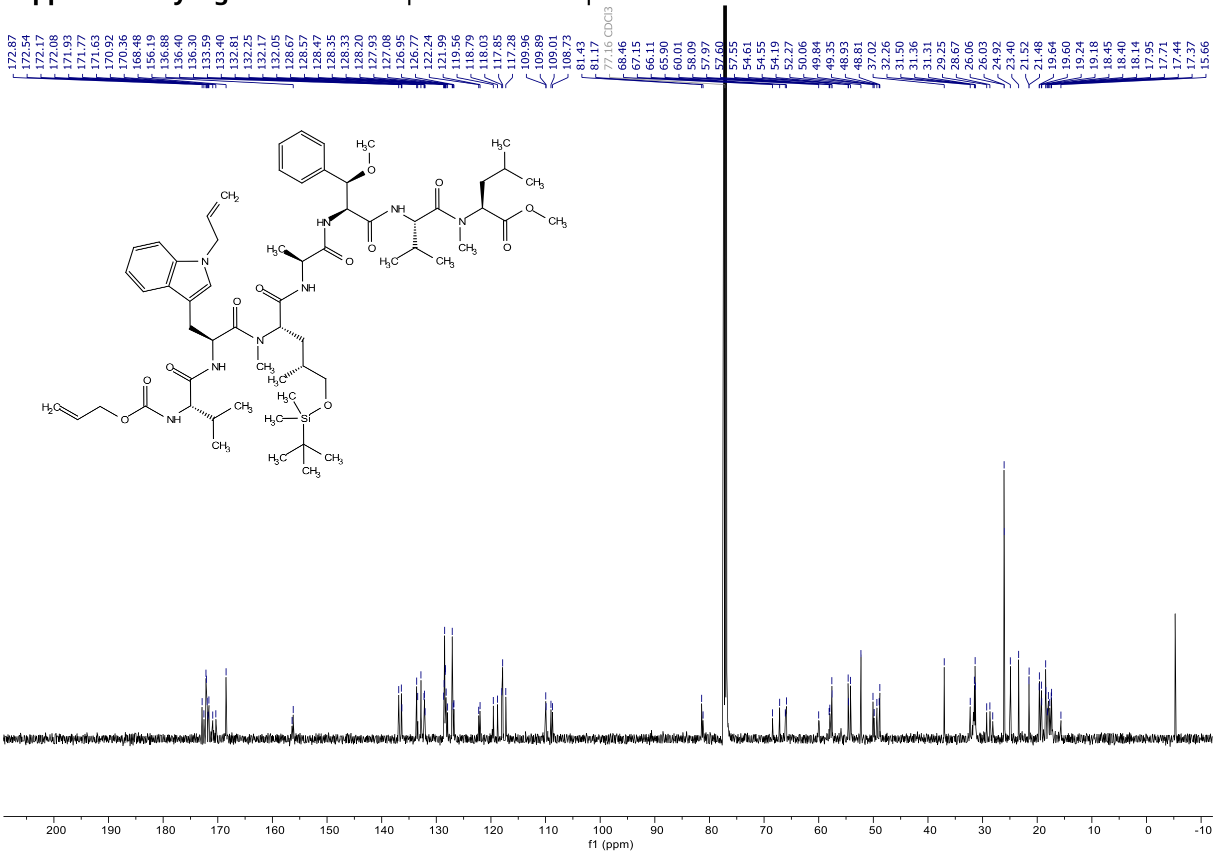

Supplementary Fig. 117. ^{1}H NMR spectrum of compound **13**

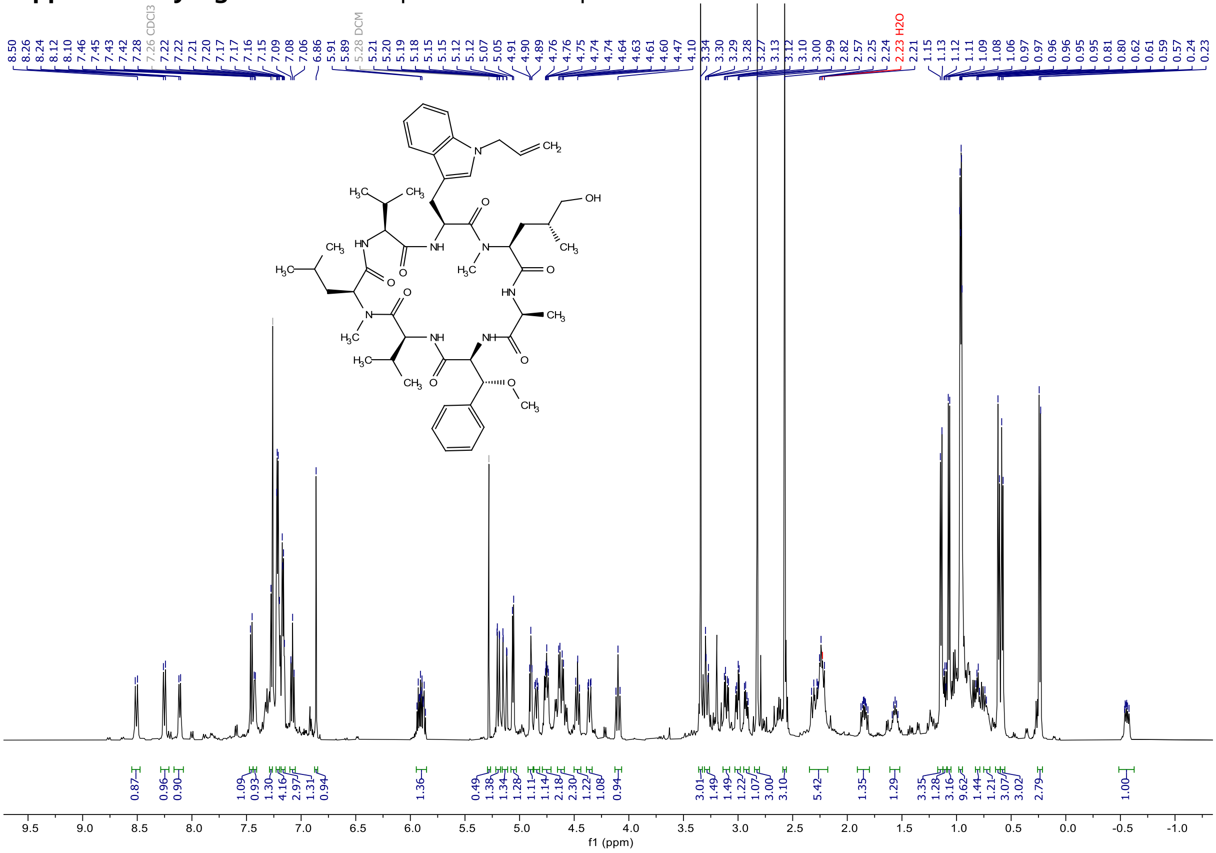

Supplementary Fig. 118. ^{13}C NMR spectrum of compound **13**

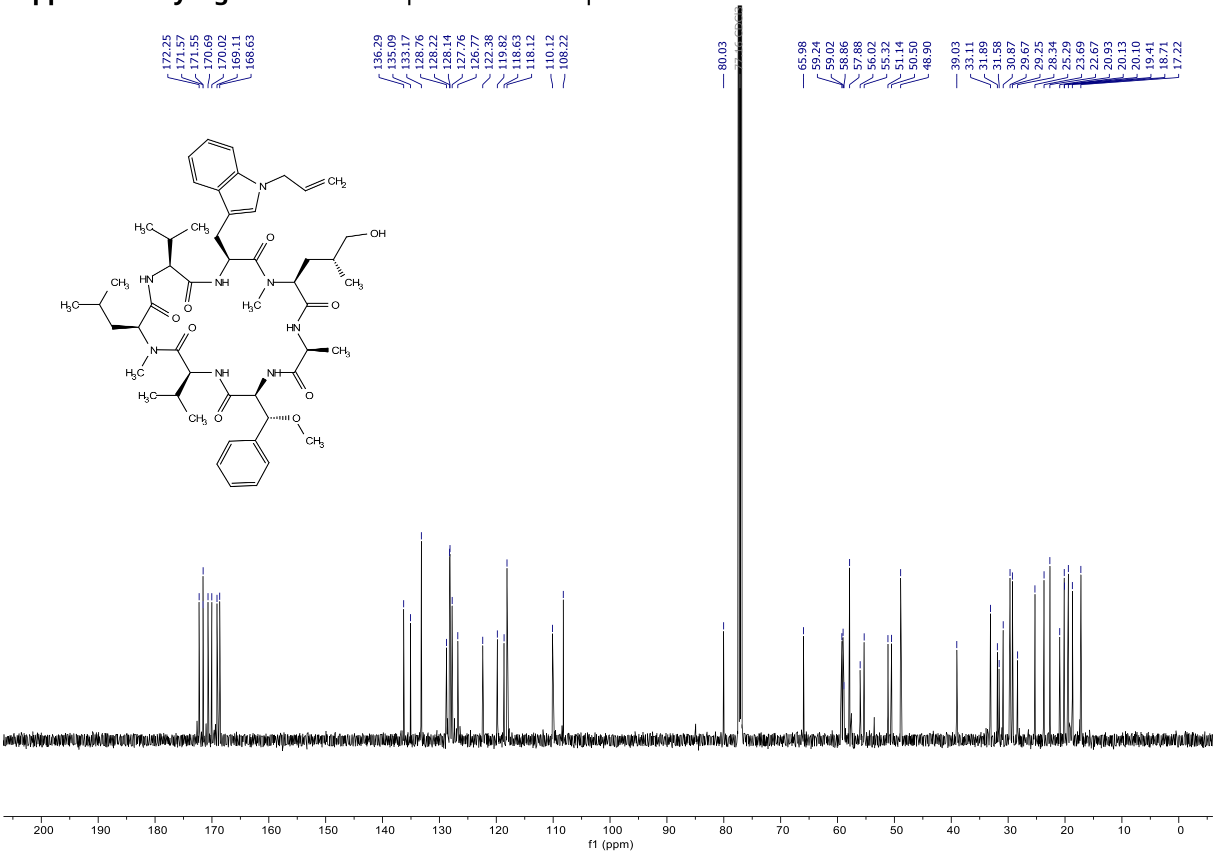

Supplementary Fig. 119. ^{1}H NMR spectrum of compound **15**

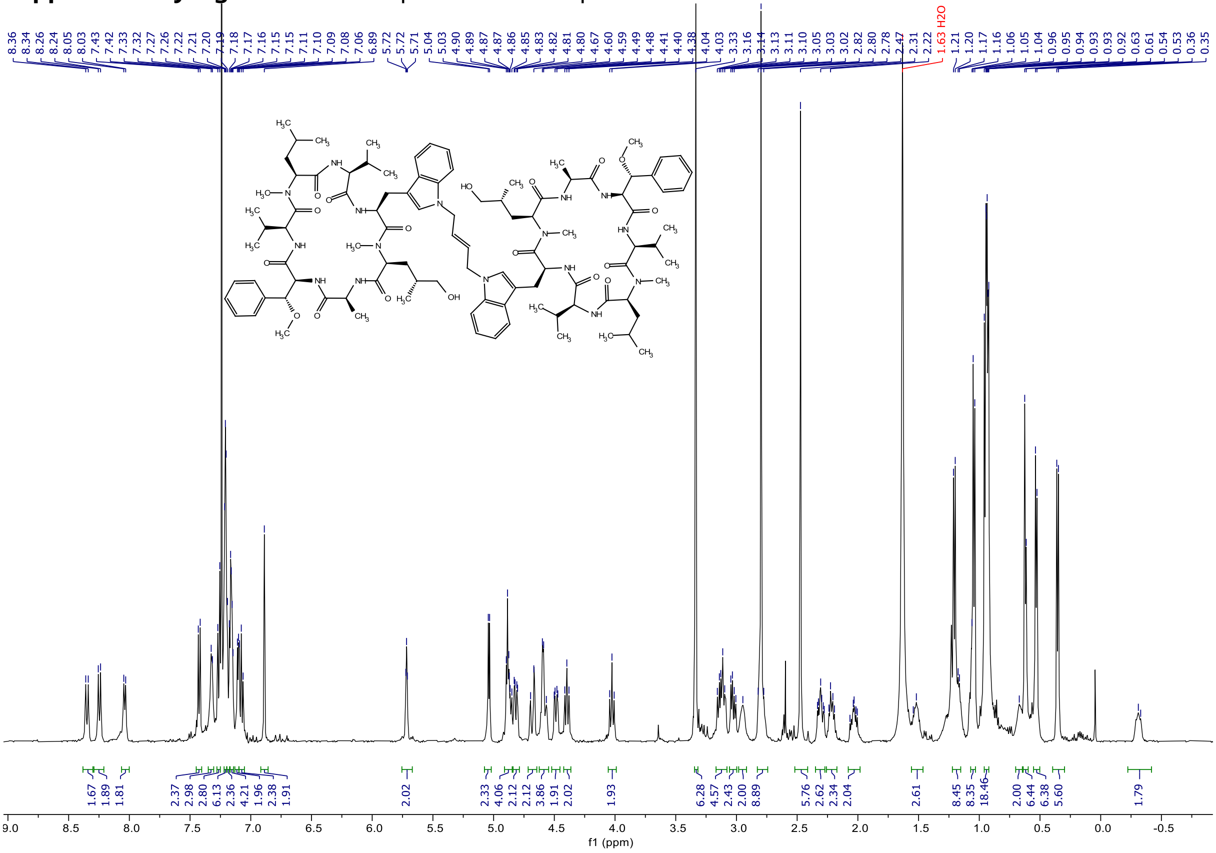

Supplementary Fig. 120. ^{13}C NMR spectrum of compound **15**

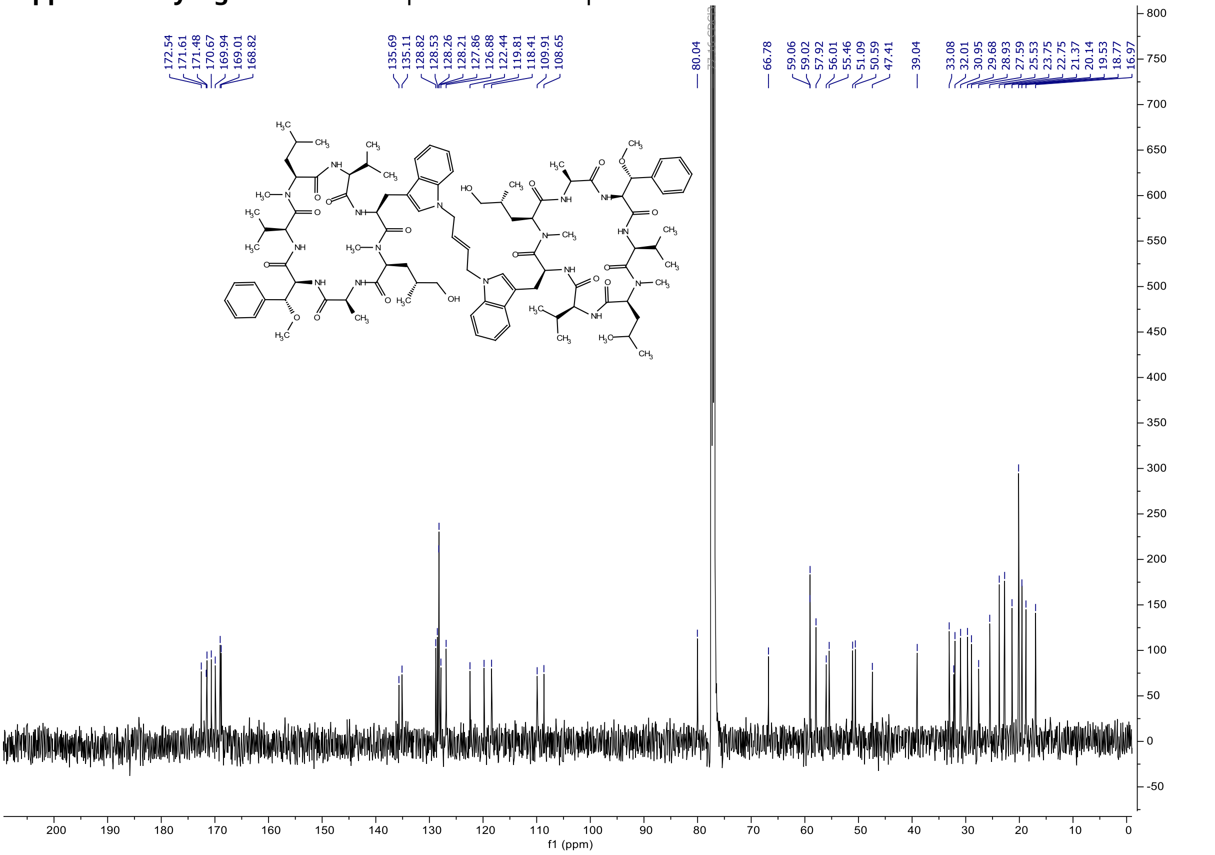

Supplementary Fig. 121. ^{1}H NMR spectrum of compound **16**

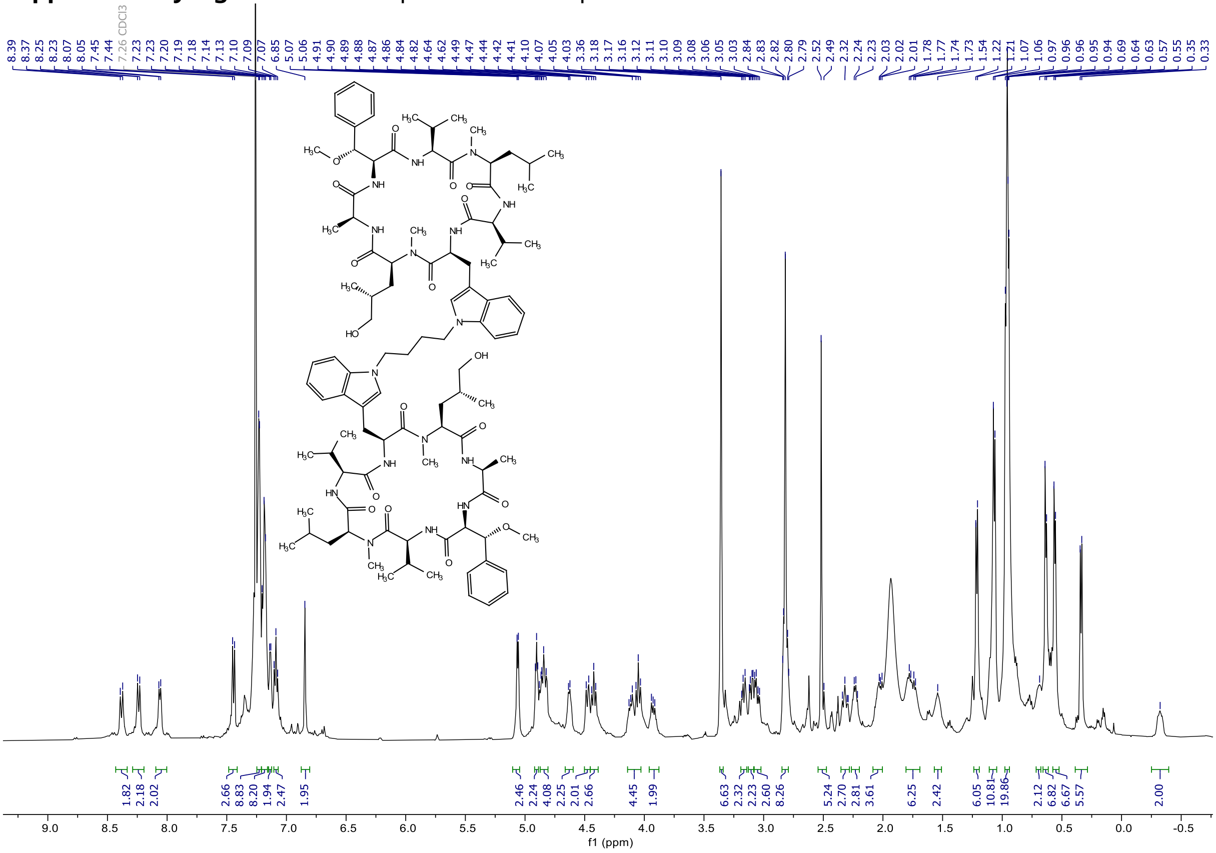

Supplementary Fig. 122. ^{13}C NMR spectrum of compound **16**

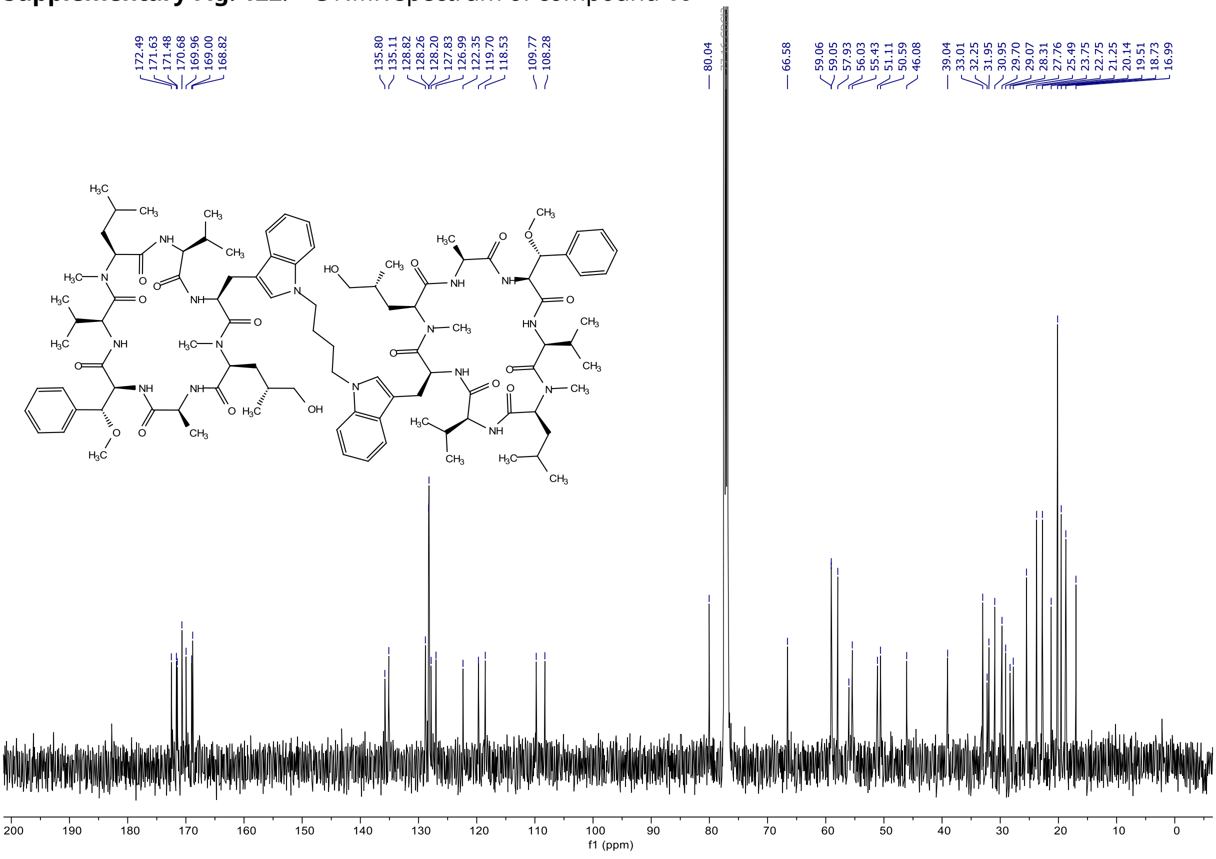

Supplementary Fig. 123. ^{1}H and ^{13}C NMR spectra of compound **SI-44**

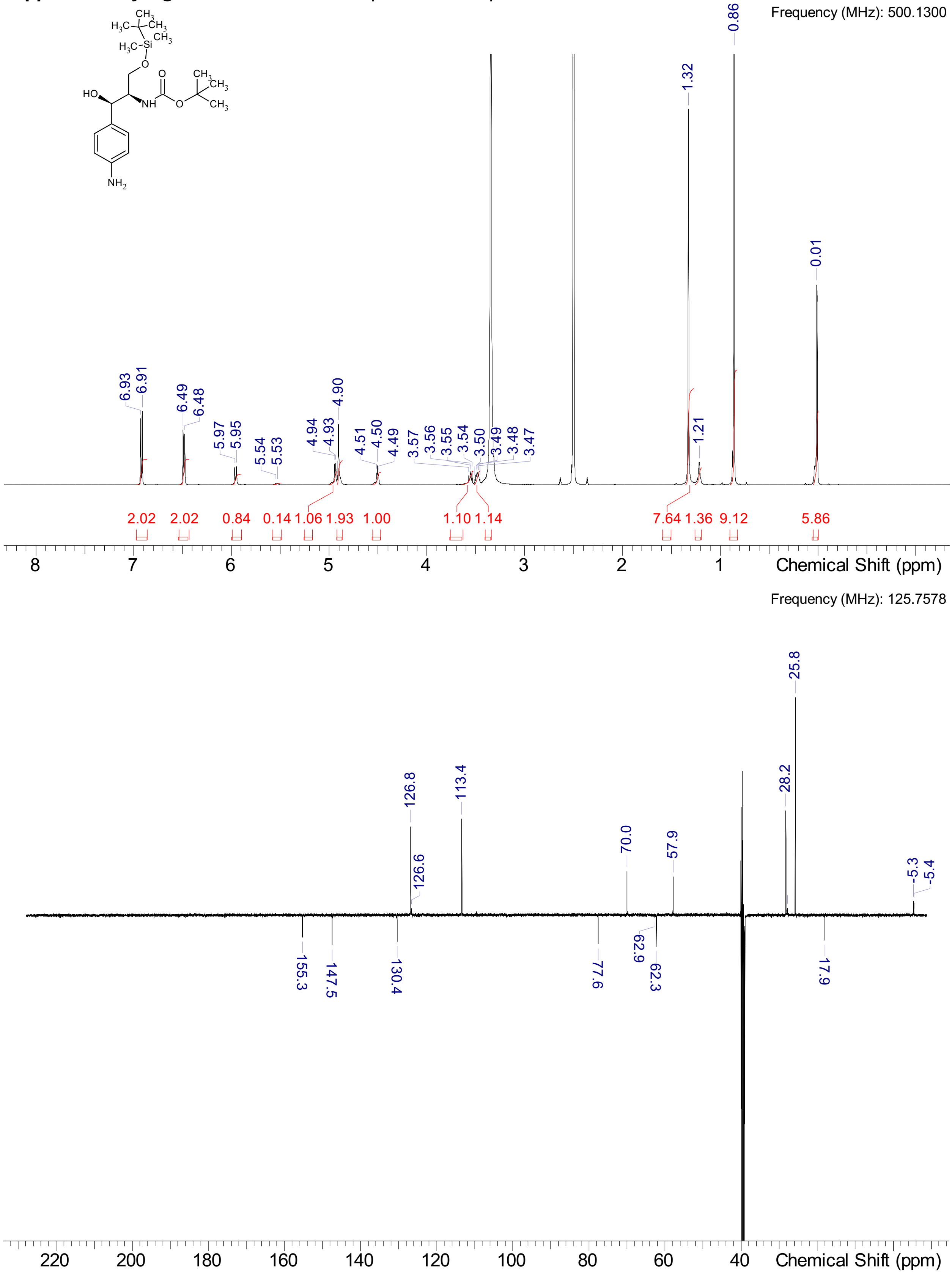

Supplementary Fig. 124. ^{1}H and ^{13}C NMR spectra of compound **SI-45**

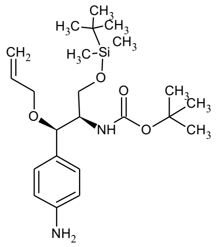

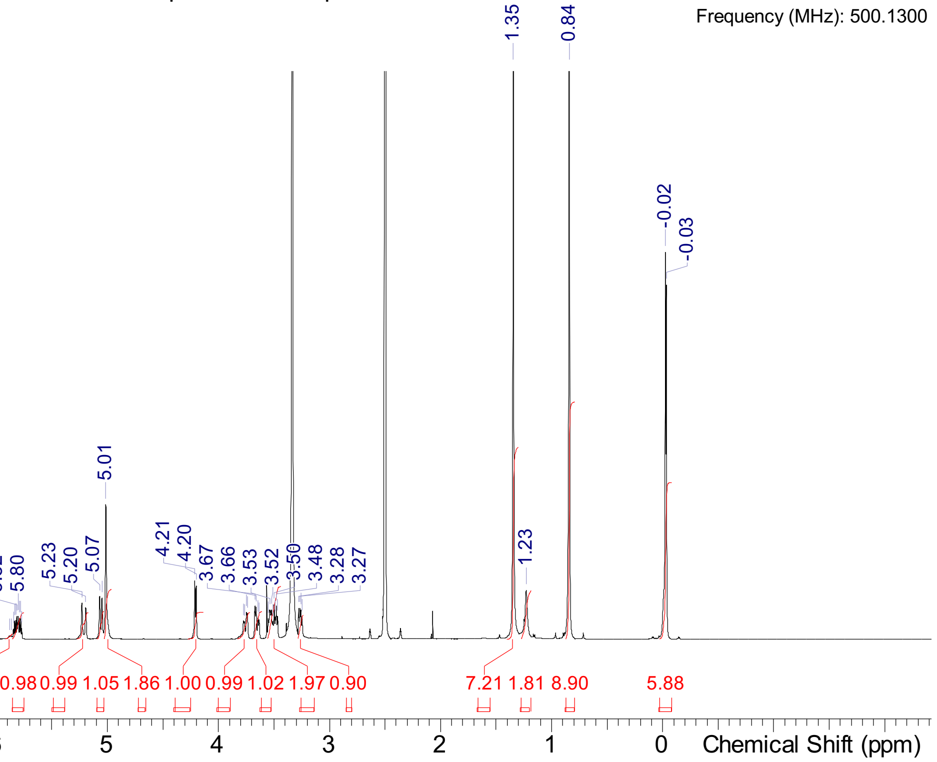

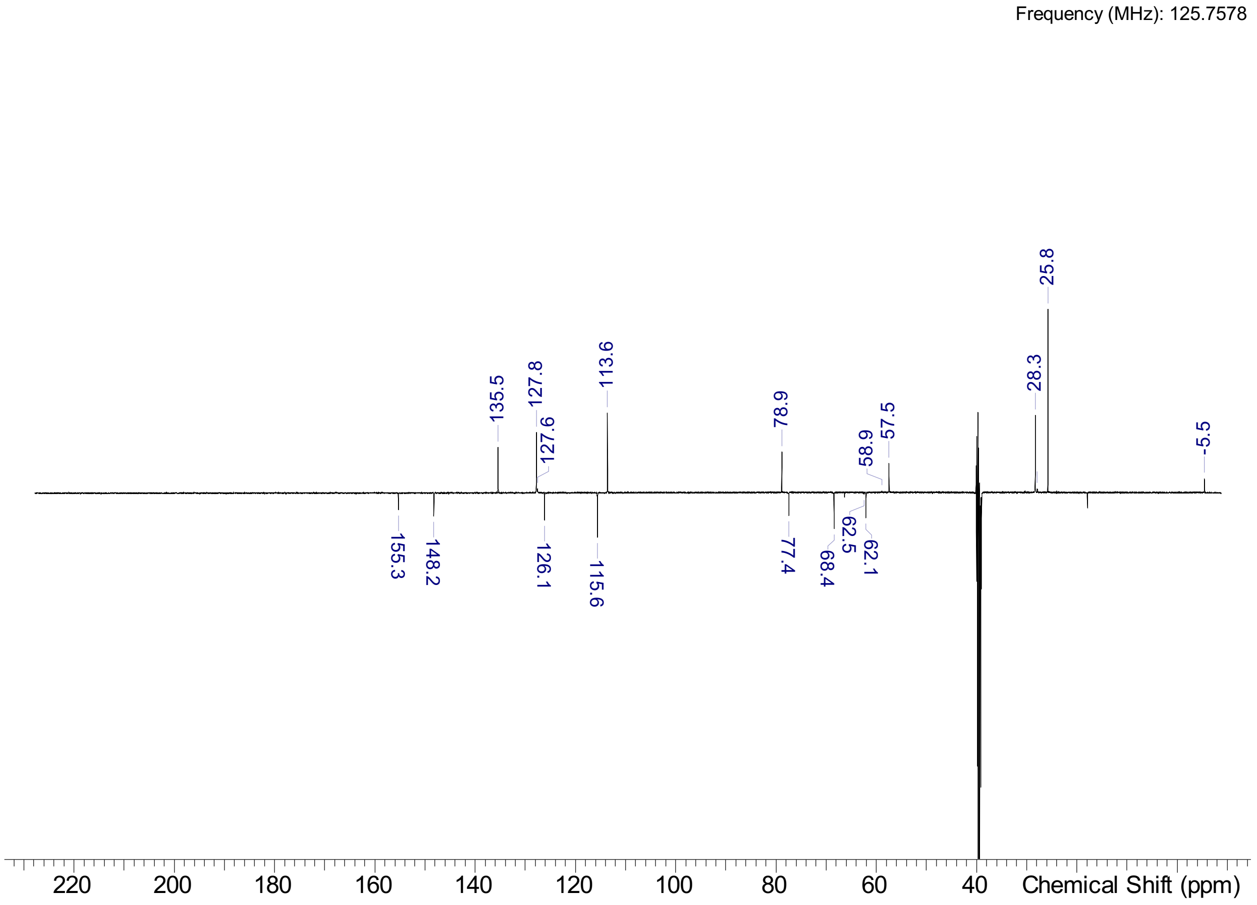

Supplementary Fig. 125. ^{1}H and ^{13}C NMR spectra of compound **SI-46**

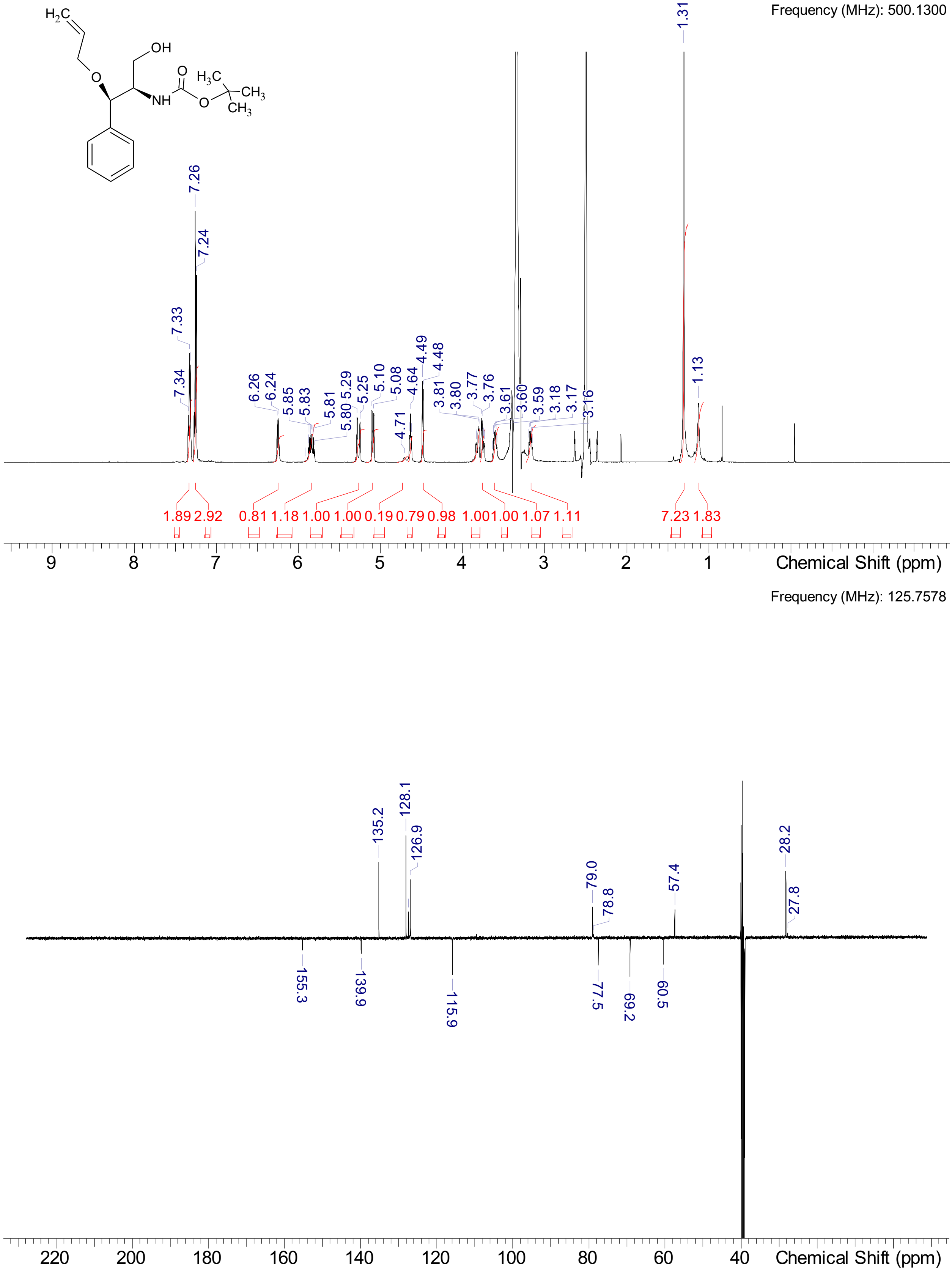

Supplementary Fig. 126. ^{1}H NMR spectrum of compound **SI-47**

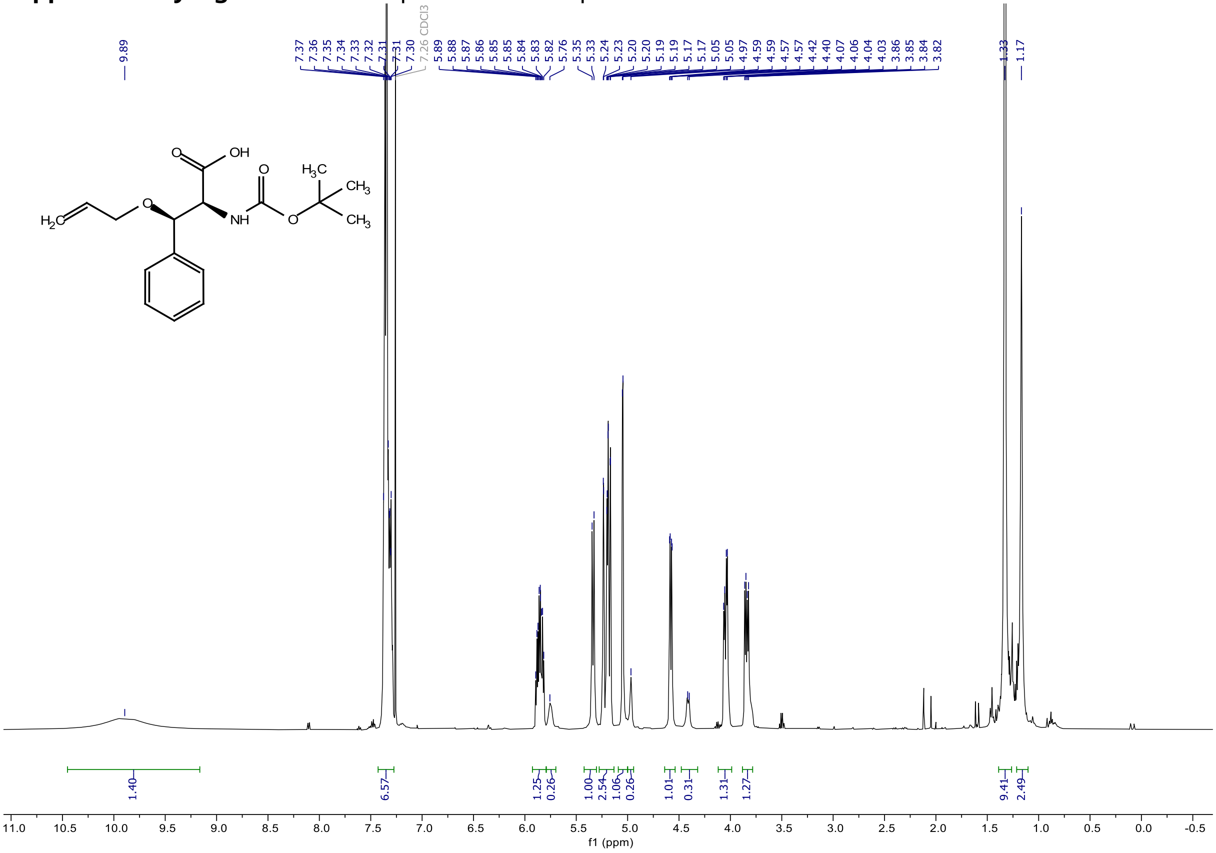

Supplementary Fig. 127. ^{13}C NMR spectrum of compound **SI-47**

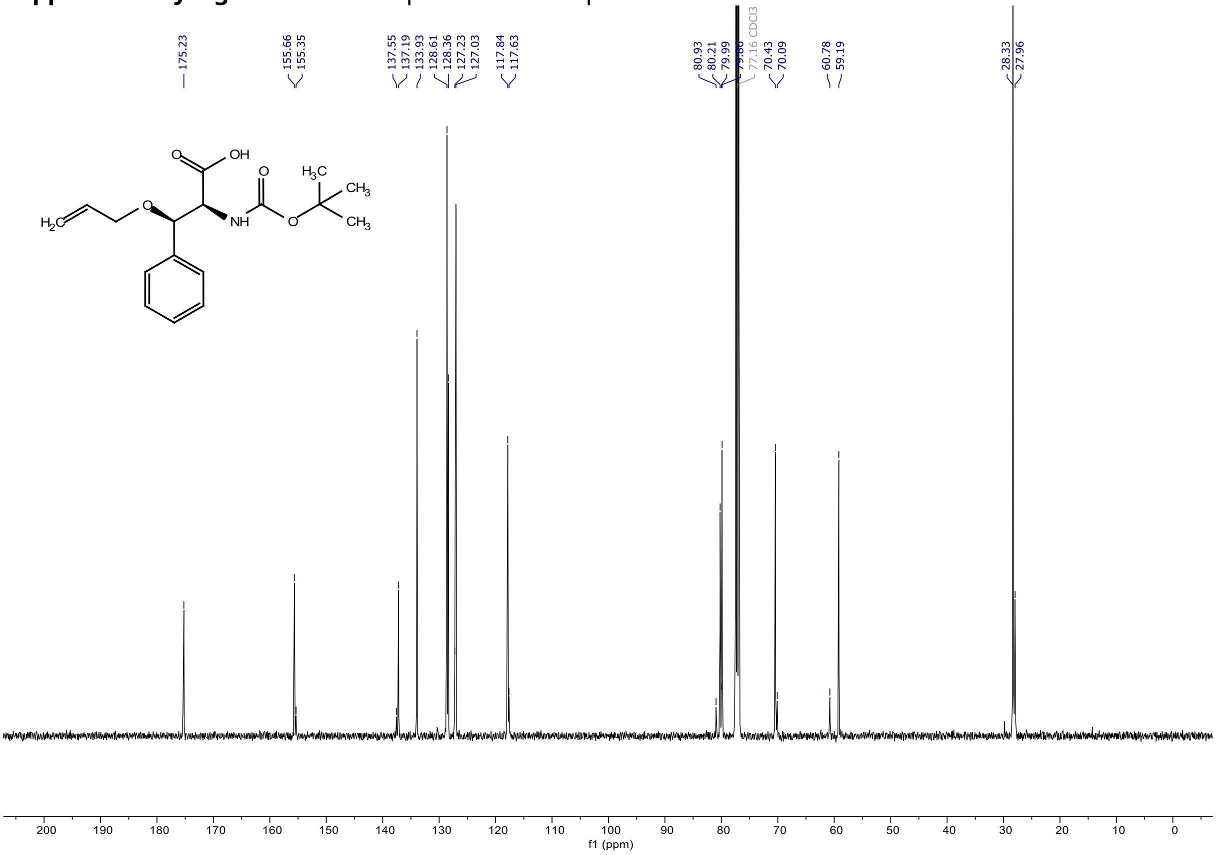

Supplementary Fig. 128. ^{1}H NMR spectrum of compound **SI-48**

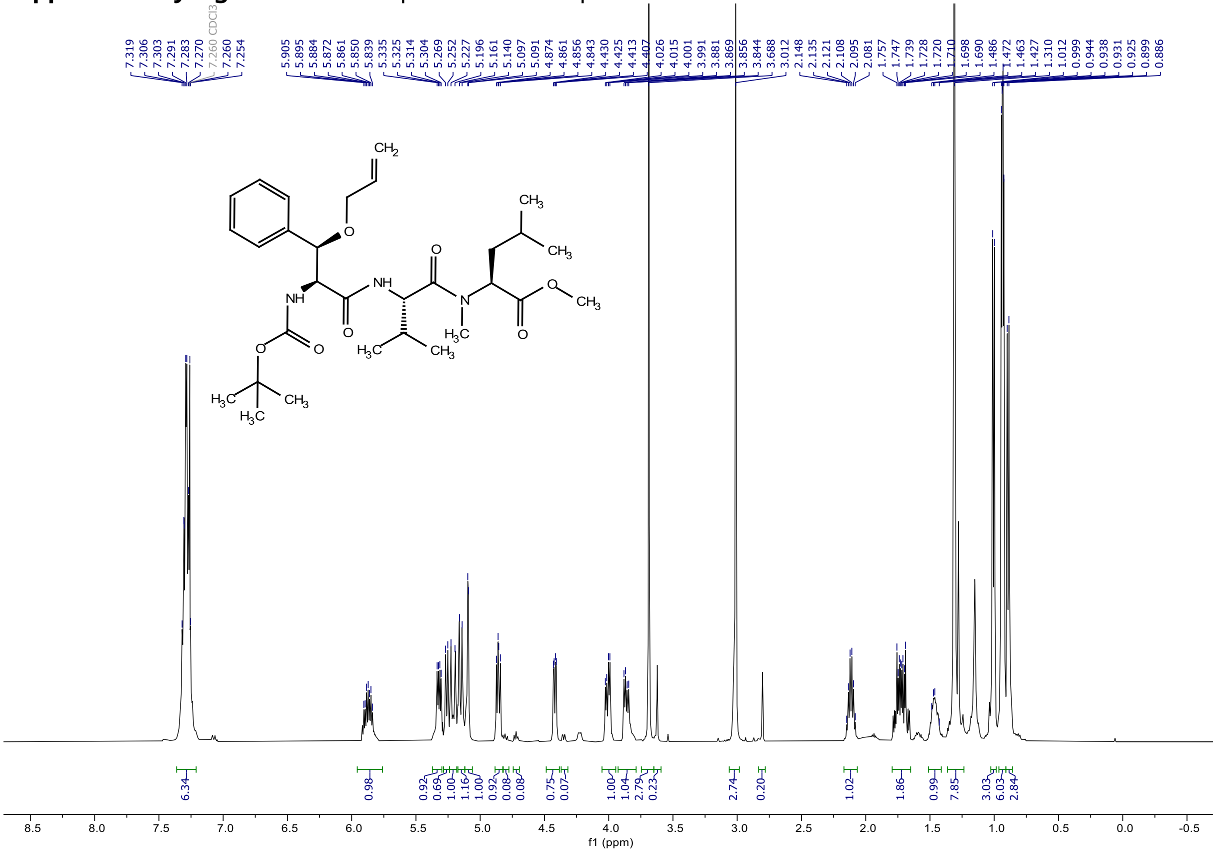

Supplementary Fig. 129. ^{13}C NMR spectrum of compound **SI-48**

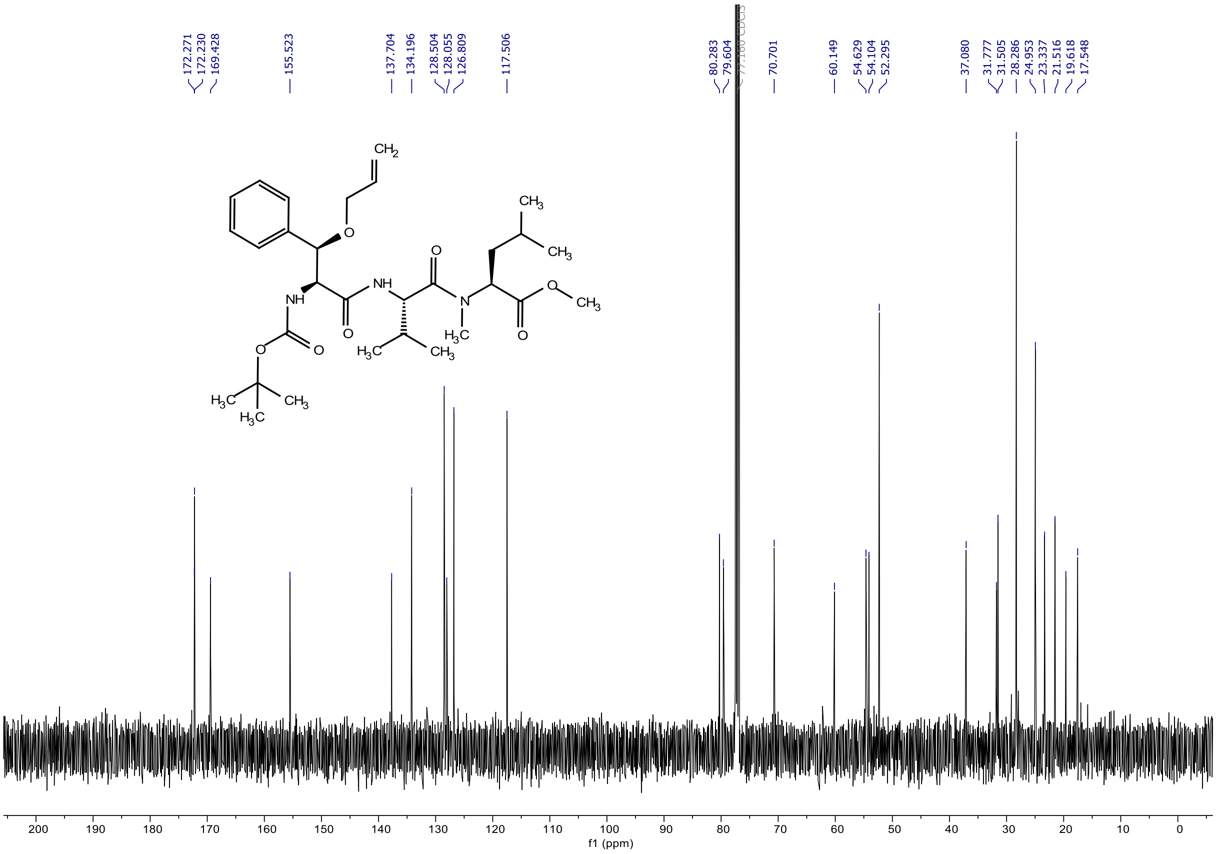

Supplementary Fig. 130. ^{1}H NMR spectrum of compound **SI-49**

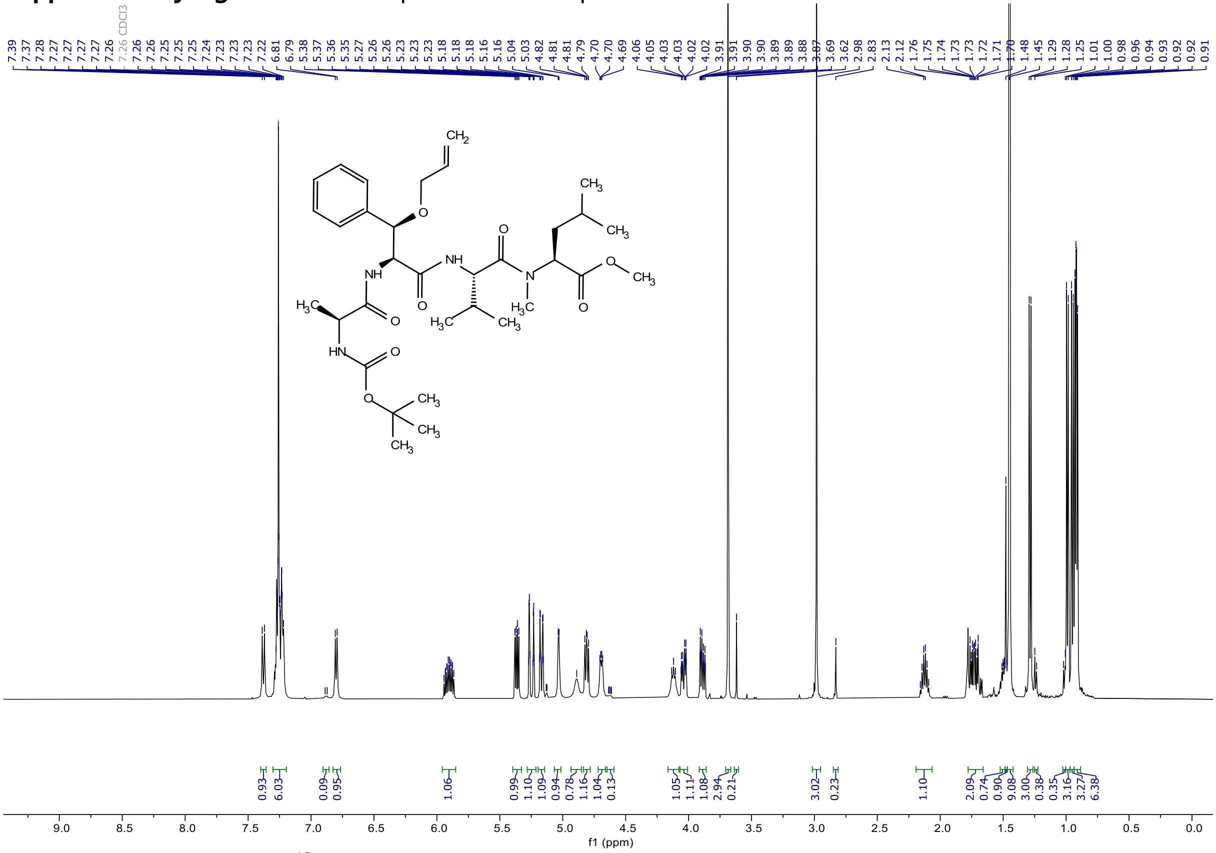

Supplementary Fig. 131. ^{13}C NMR spectrum of compound **SI-49**

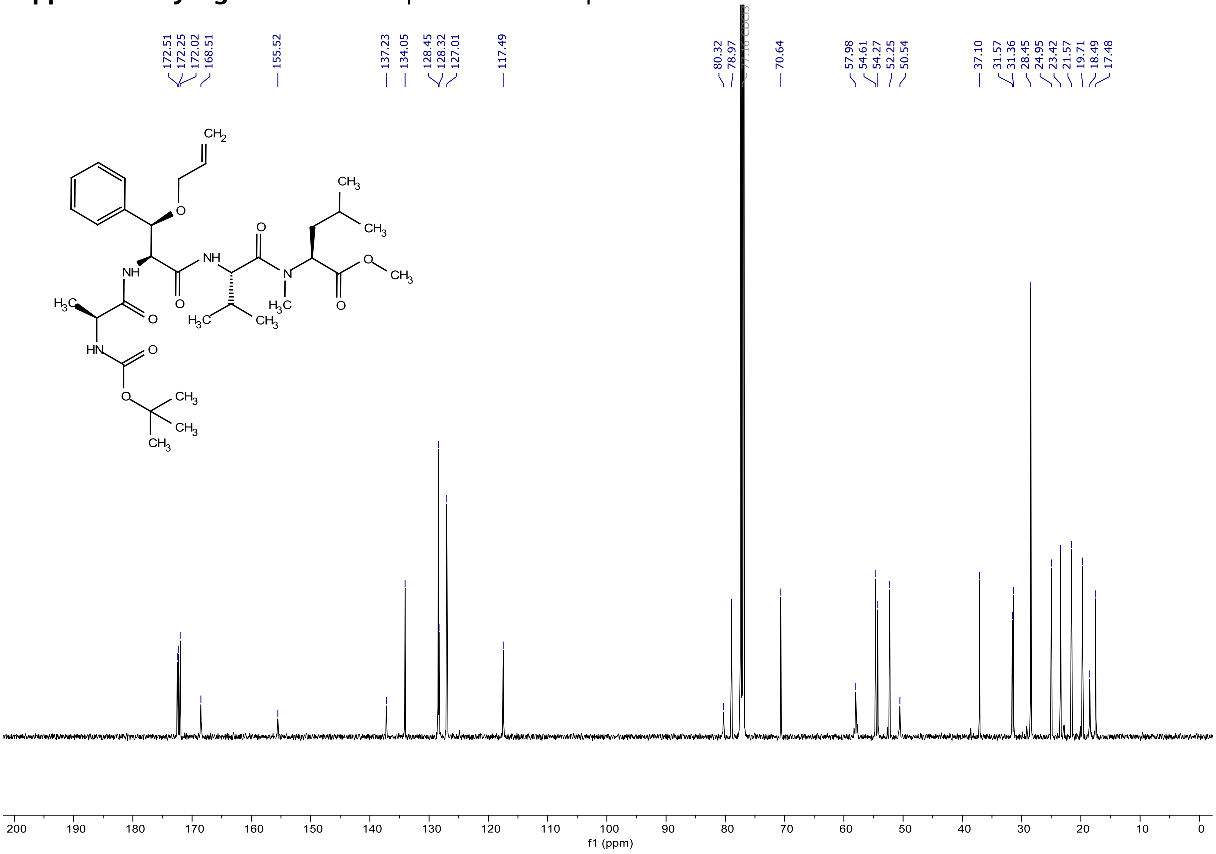

Supplementary Fig. 132. ^{1}H NMR spectrum of compound **SI-50**

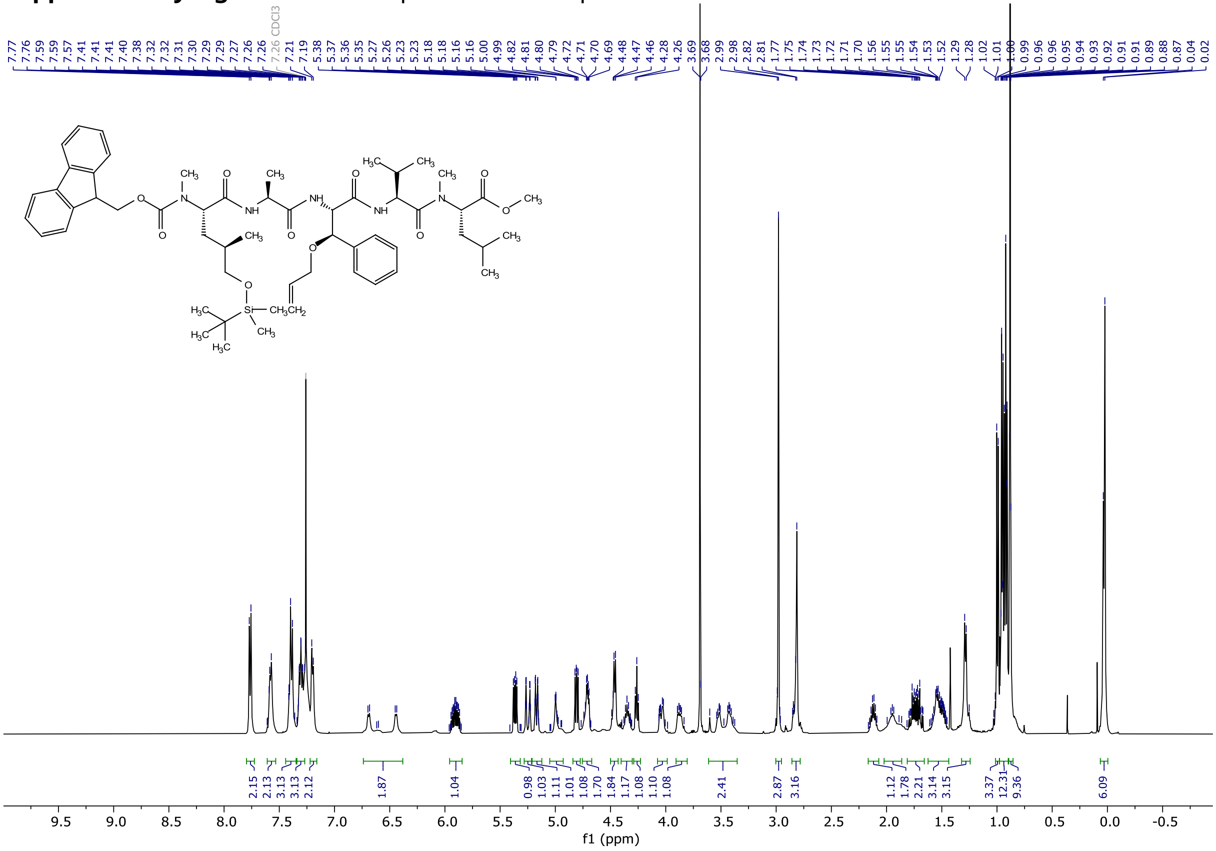

Supplementary Fig. 133. ^{13}C NMR spectrum of compound **SI-50**

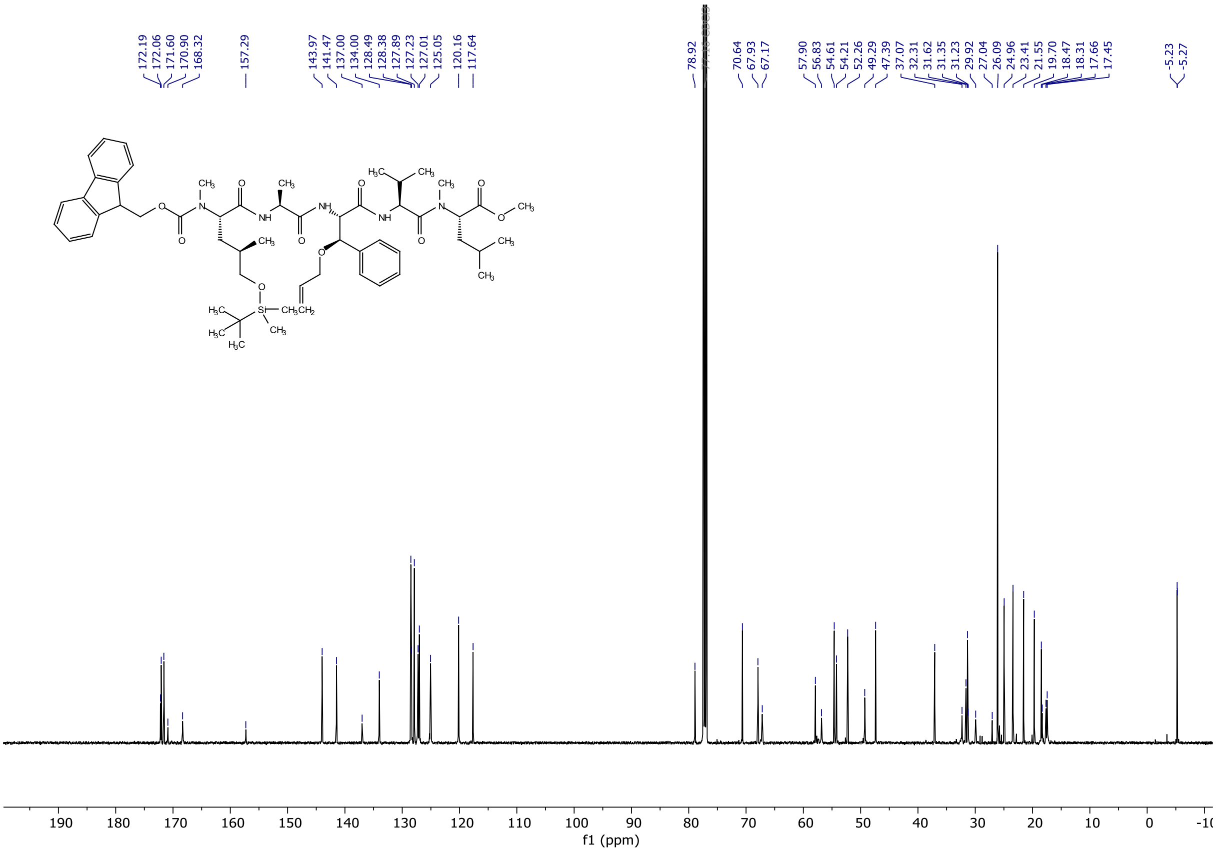

Supplementary Fig. 134. ^{1}H NMR spectrum of compound **SI-51**

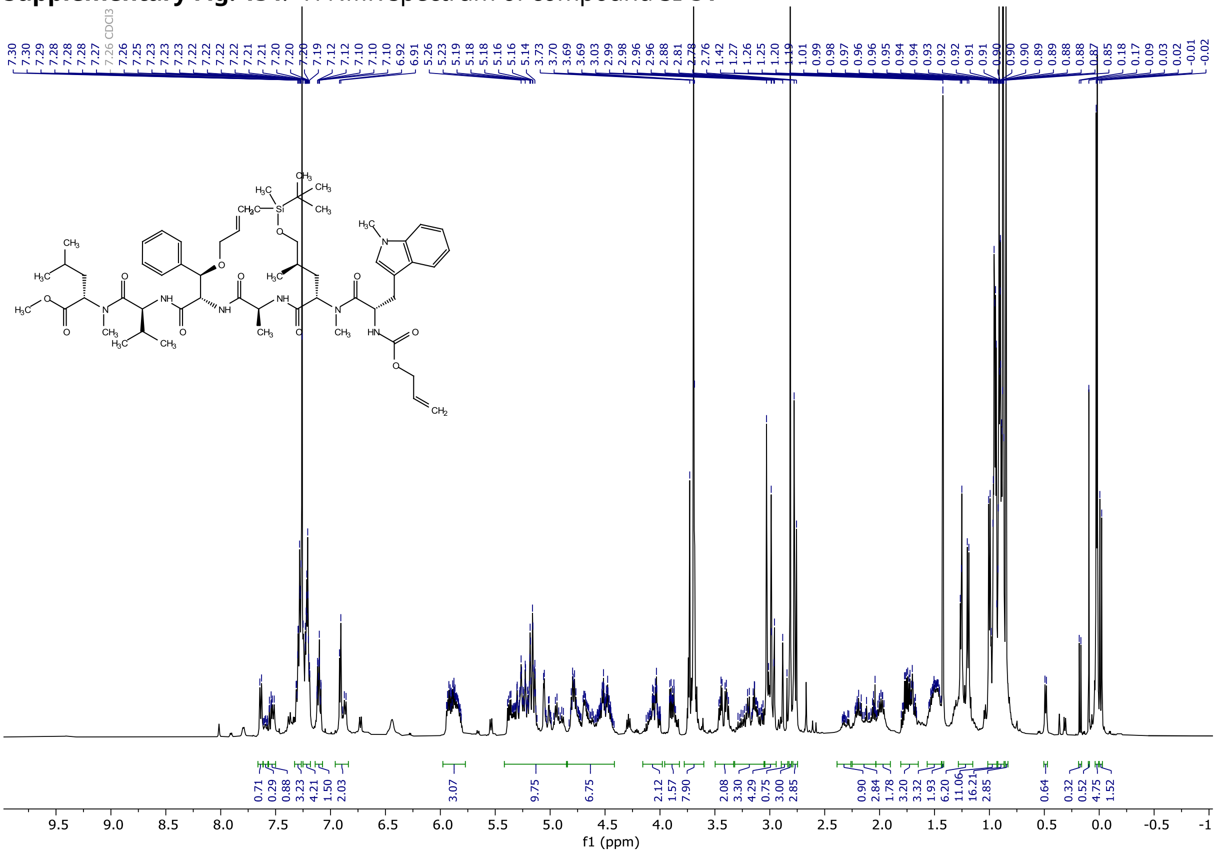

Supplementary Fig. 135. ^{13}C NMR spectrum of compound **SI-51**

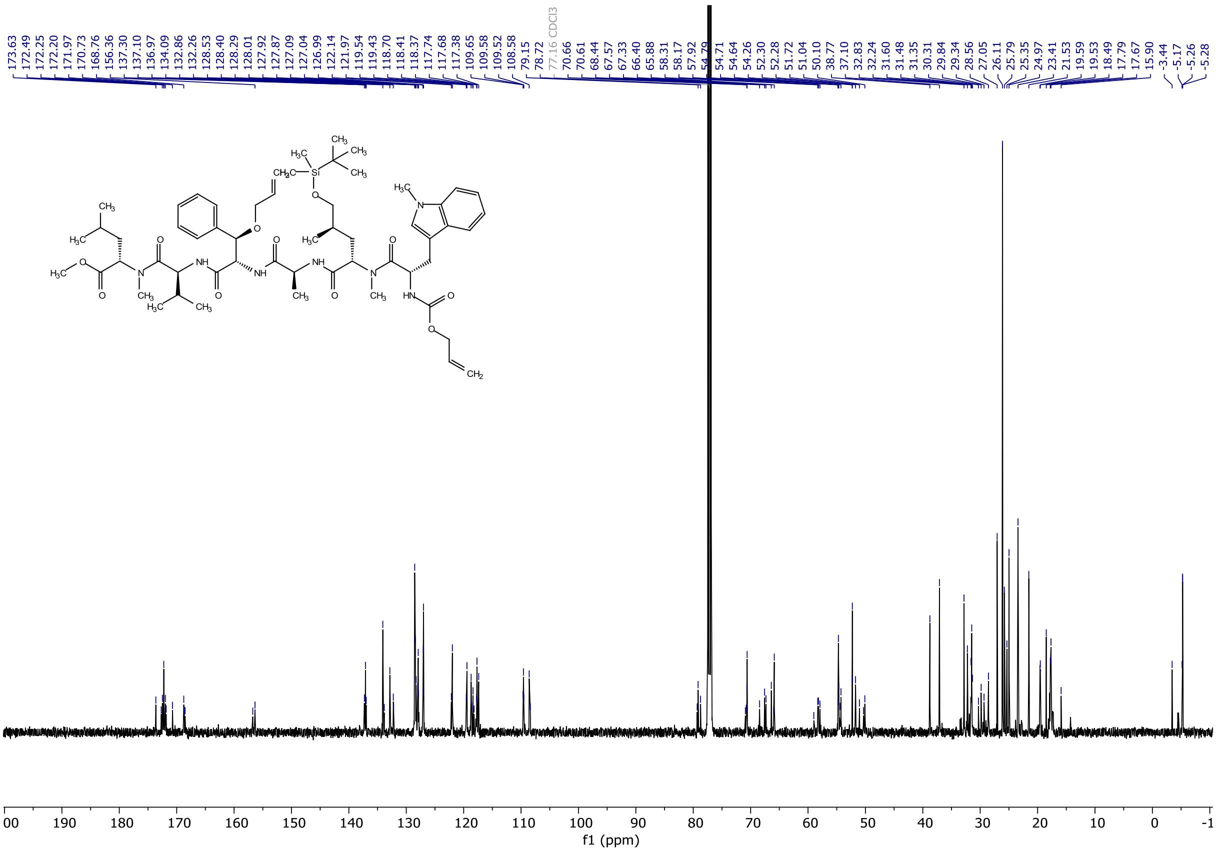

Supplementary Fig. 136. ^{1}H NMR spectrum of compound **SI-52**

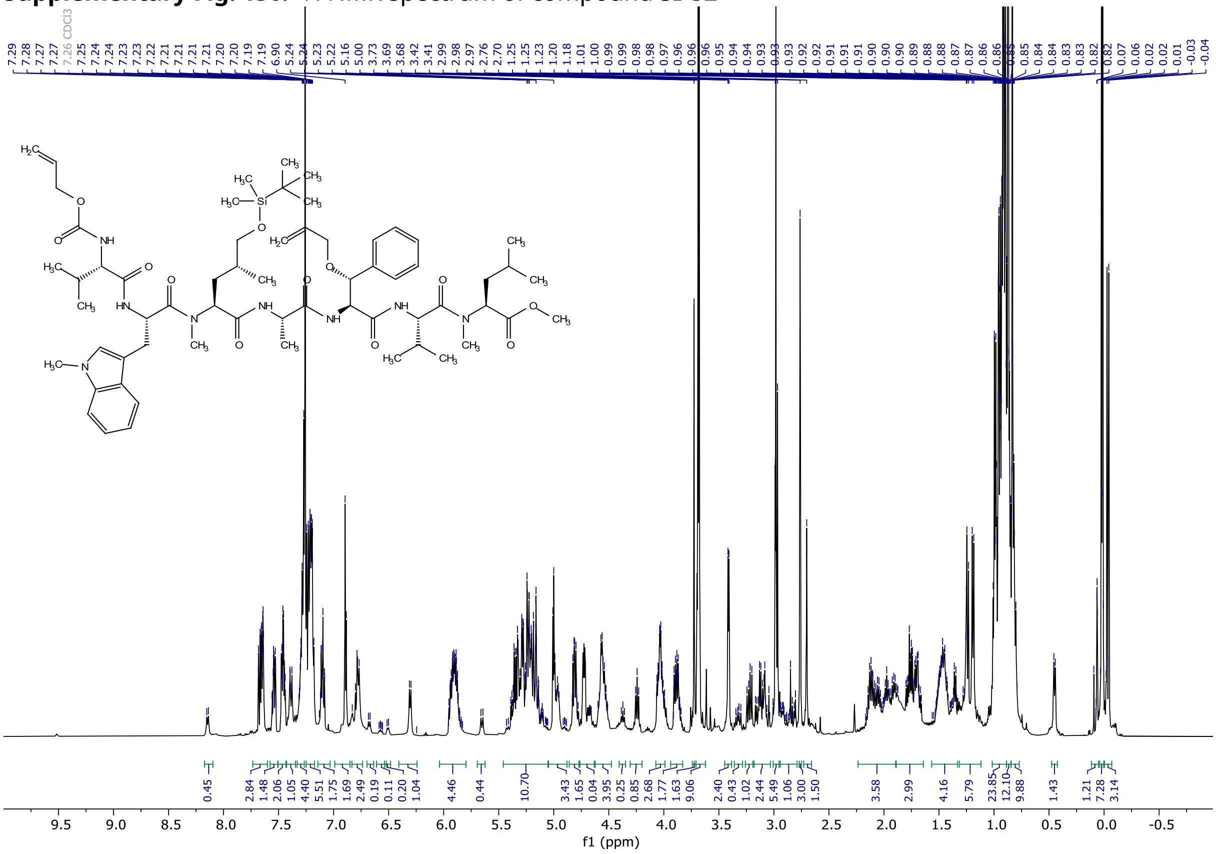

Supplementary Fig. 137. ^{13}C NMR spectrum of compound **SI-52**

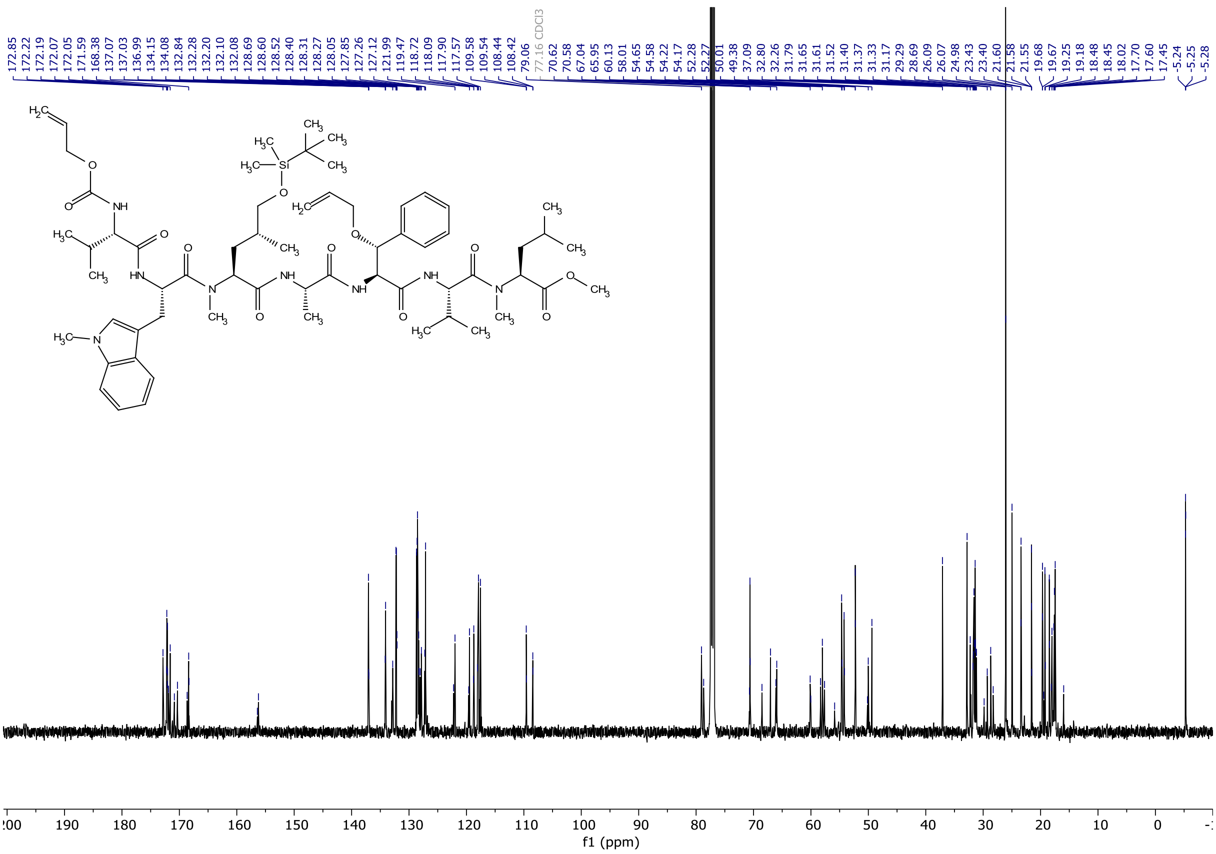

Supplementary Fig. 138. ^{1}H NMR spectrum of compound **14**

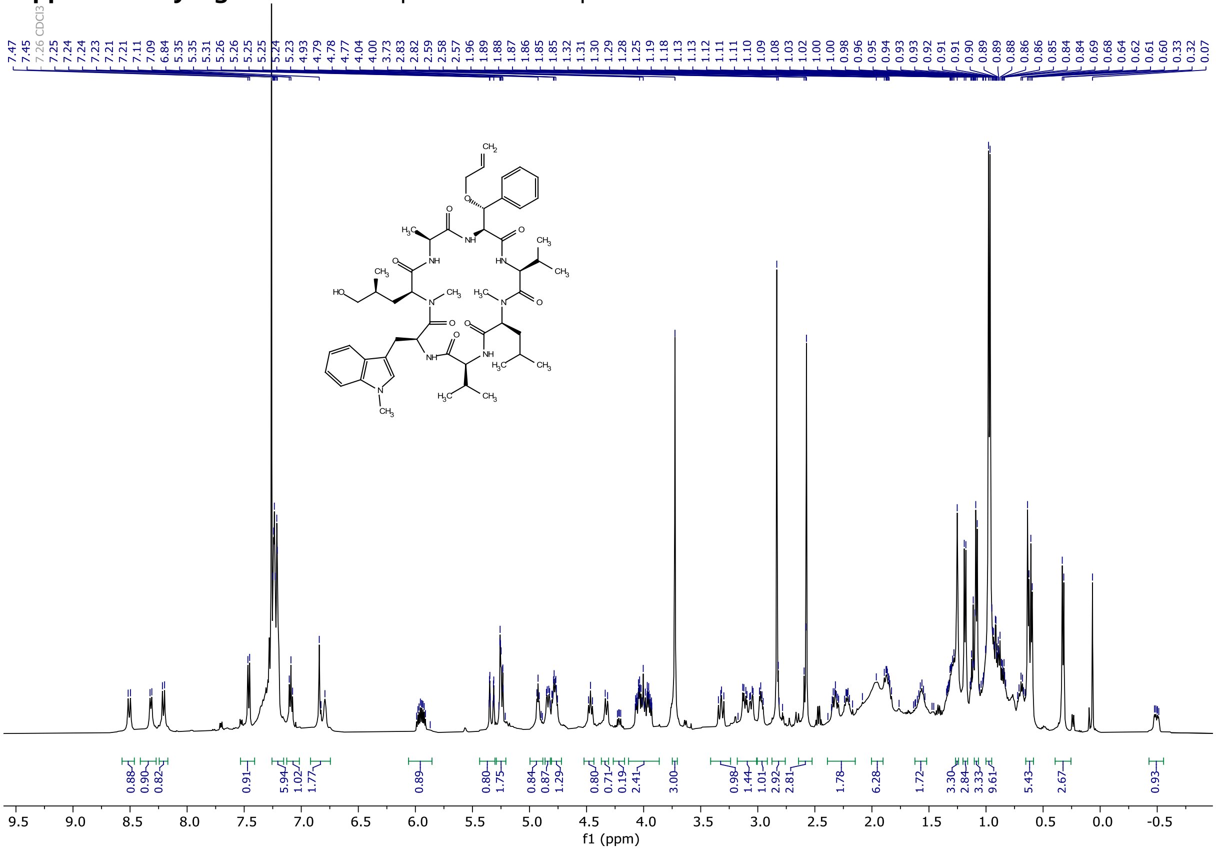

Supplementary Fig. 139. ^{13}C NMR spectrum of compound **14**

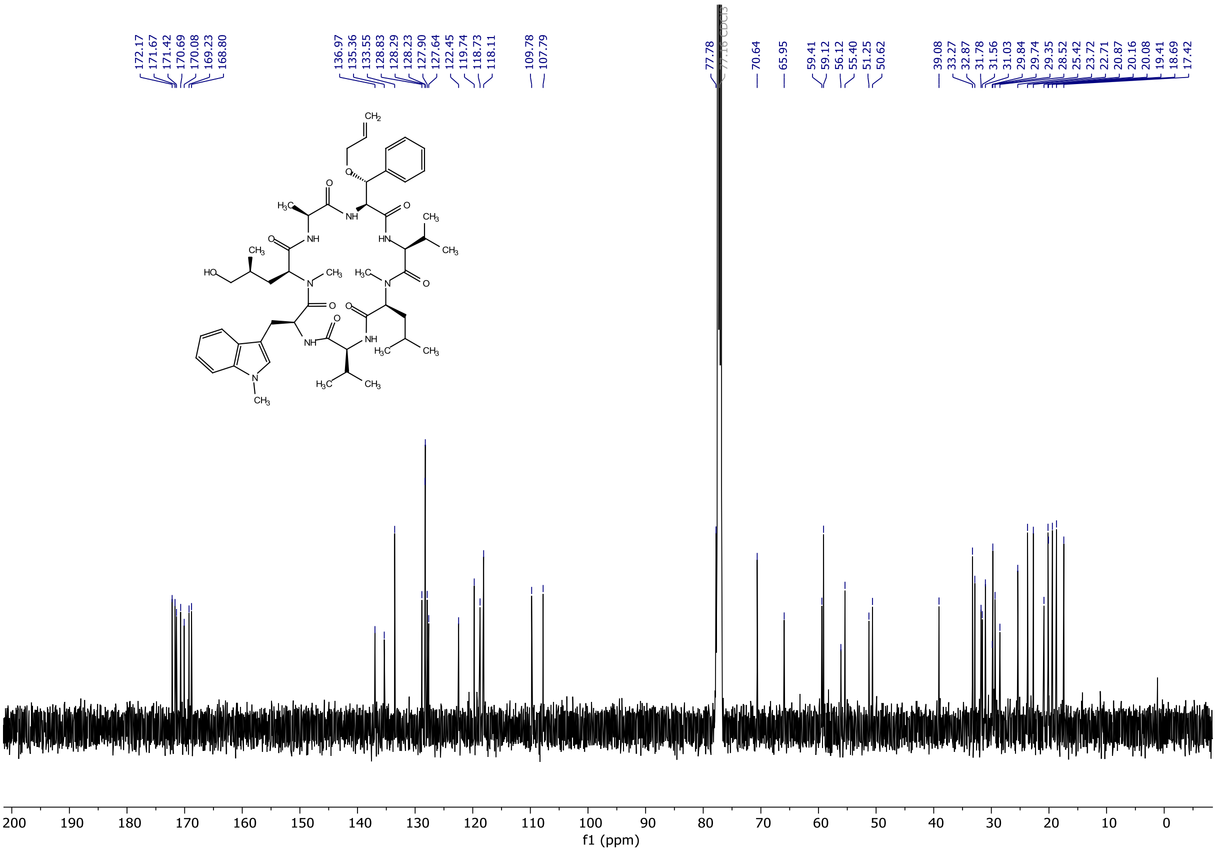

Supplementary Fig. 140. ^{1}H NMR spectrum of compound **17**

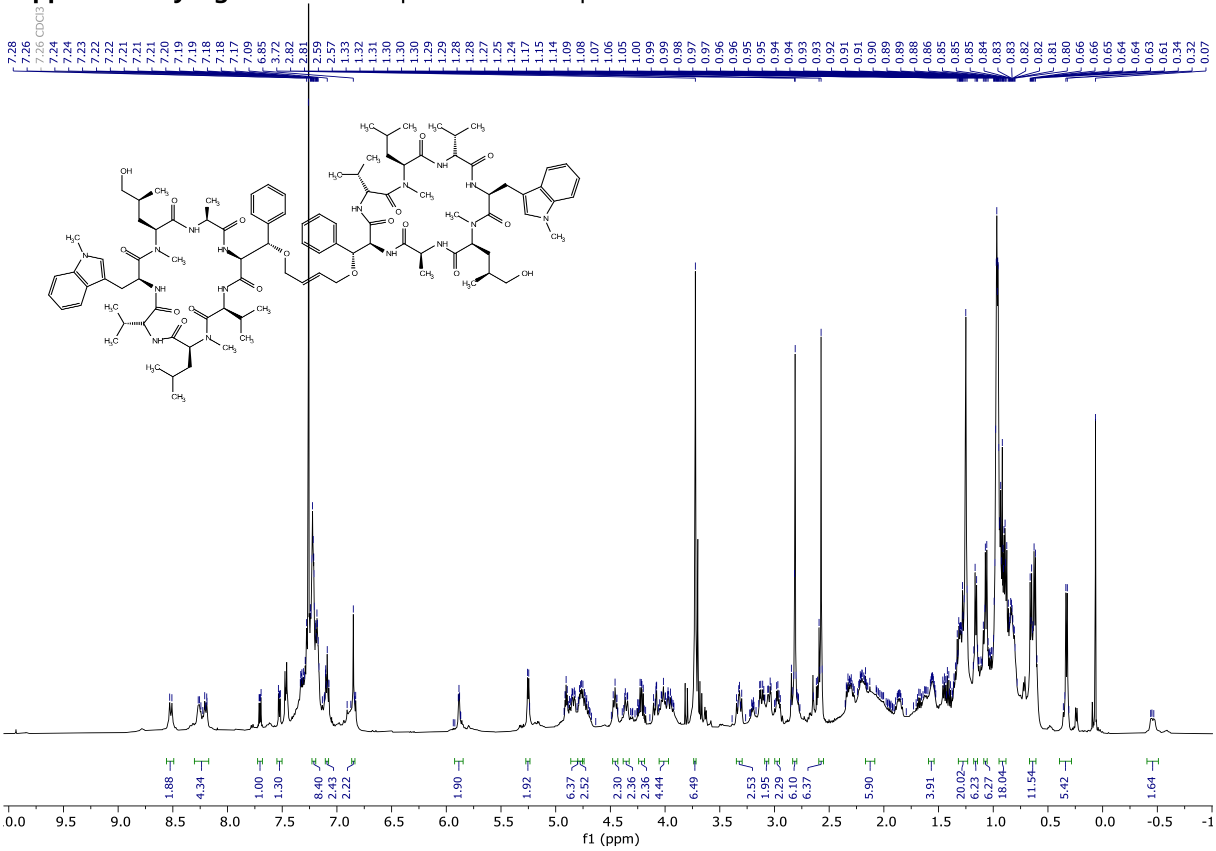

Supplementary Fig. 141. ^{13}C NMR spectrum of compound **17**

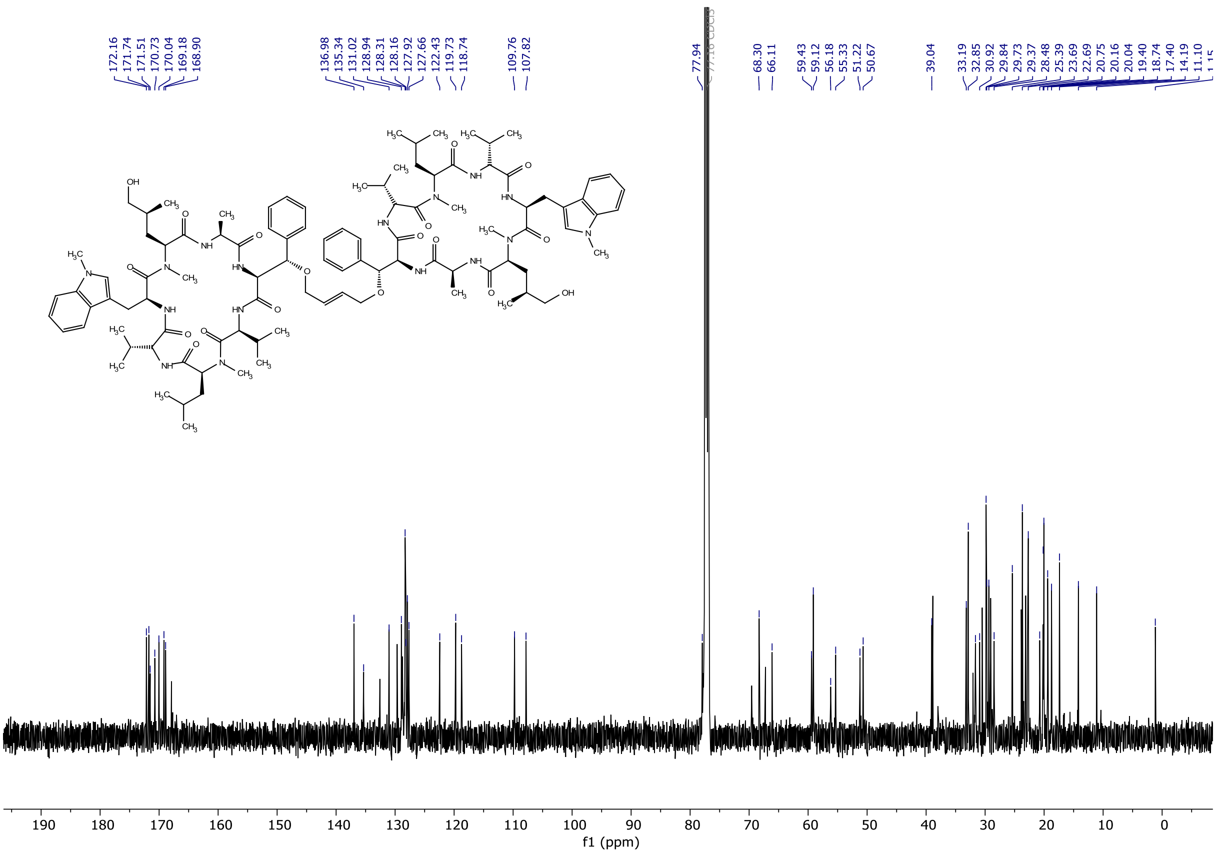

Supplementary Fig. 142. ^{1}H NMR spectrum of compound **18**

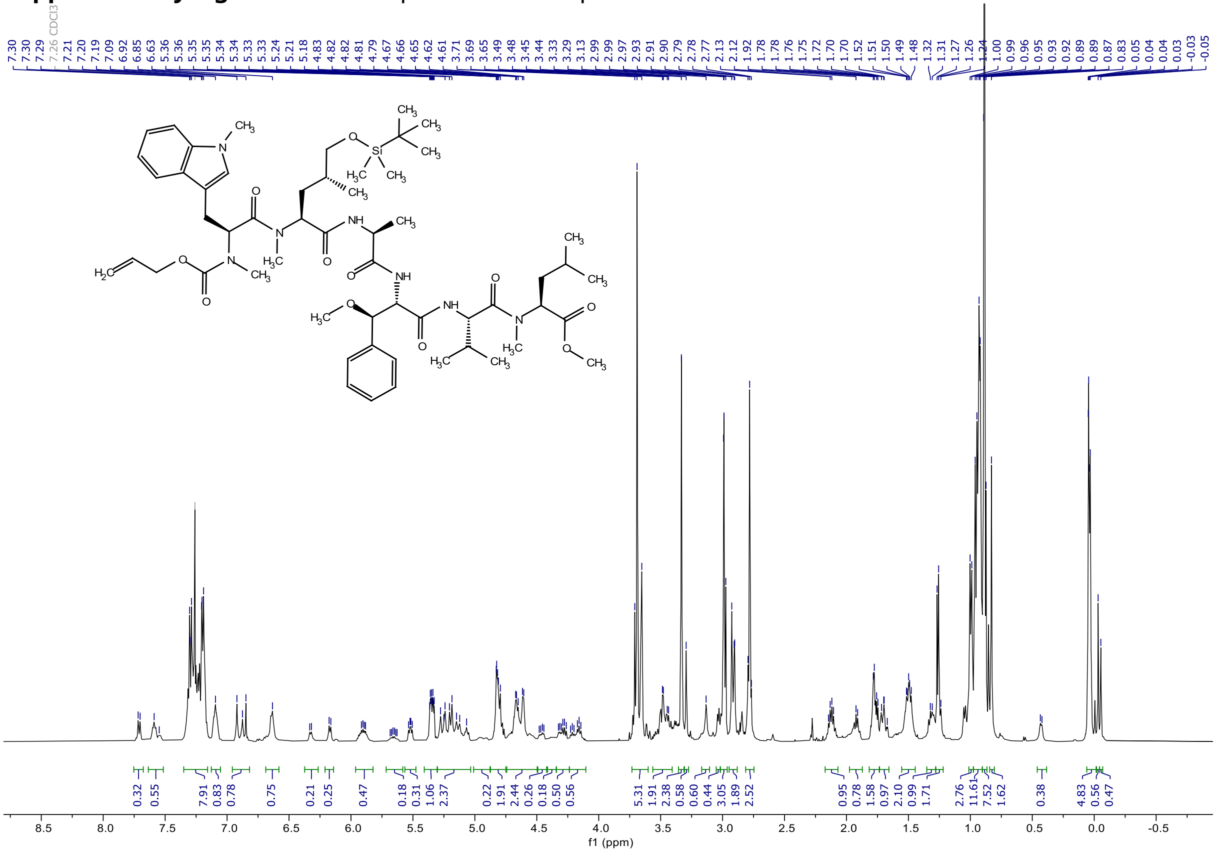

Supplementary Fig. 143. ^{13}C NMR spectrum of compound **18**

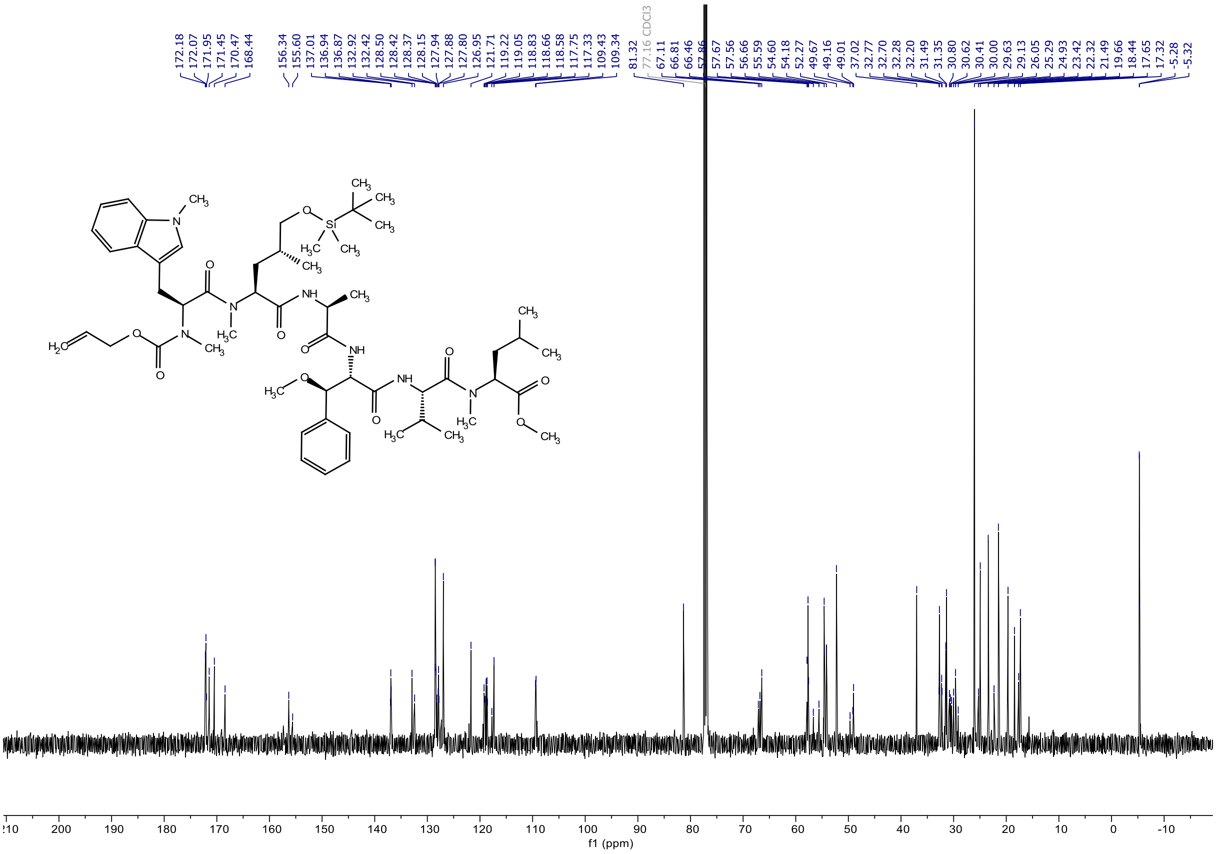

Supplementary Fig. 144. ^{1}H NMR spectrum of compound **19**

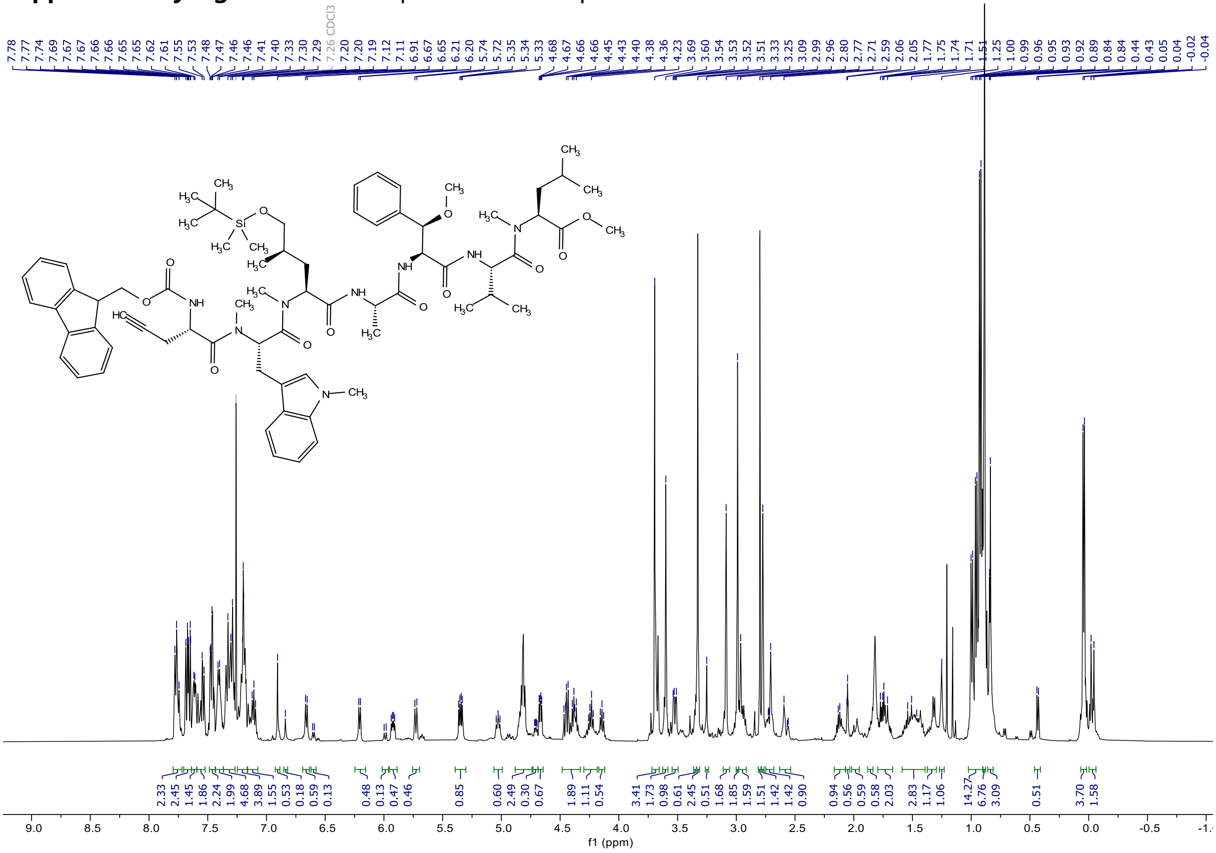

Supplementary Fig. 145. ^{13}C NMR spectrum of compound **19**

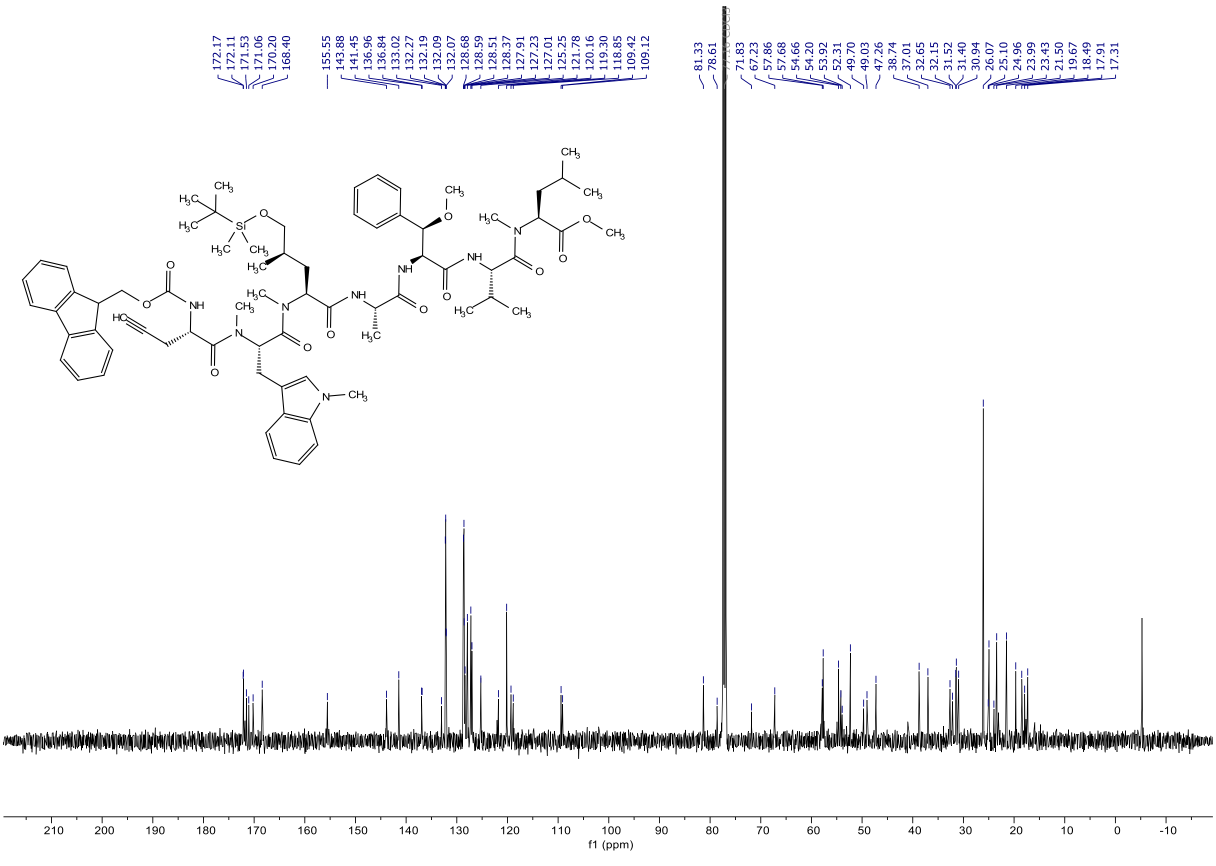

Supplementary Fig. 146. ^{1}H NMR spectrum of compound **20**

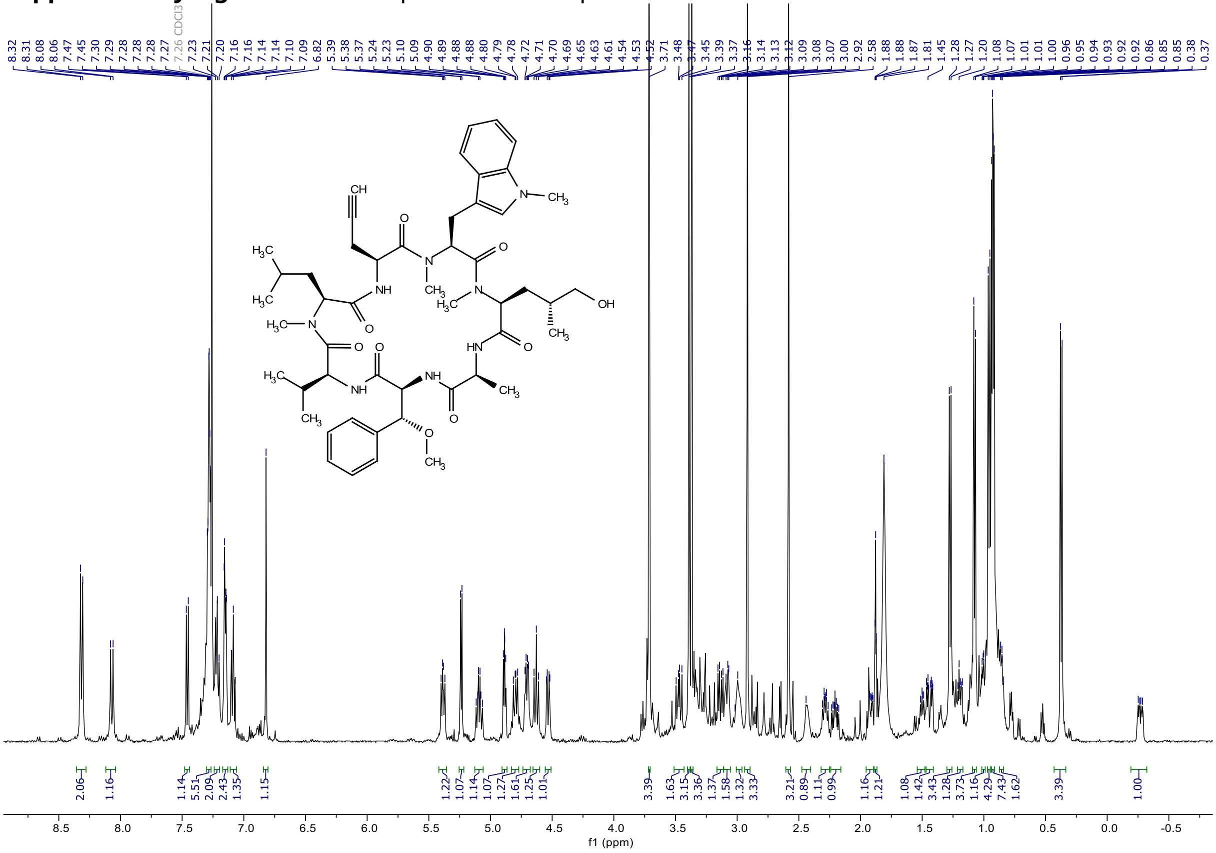

Supplementary Fig. 147. ^{13}C NMR spectrum of compound **20**

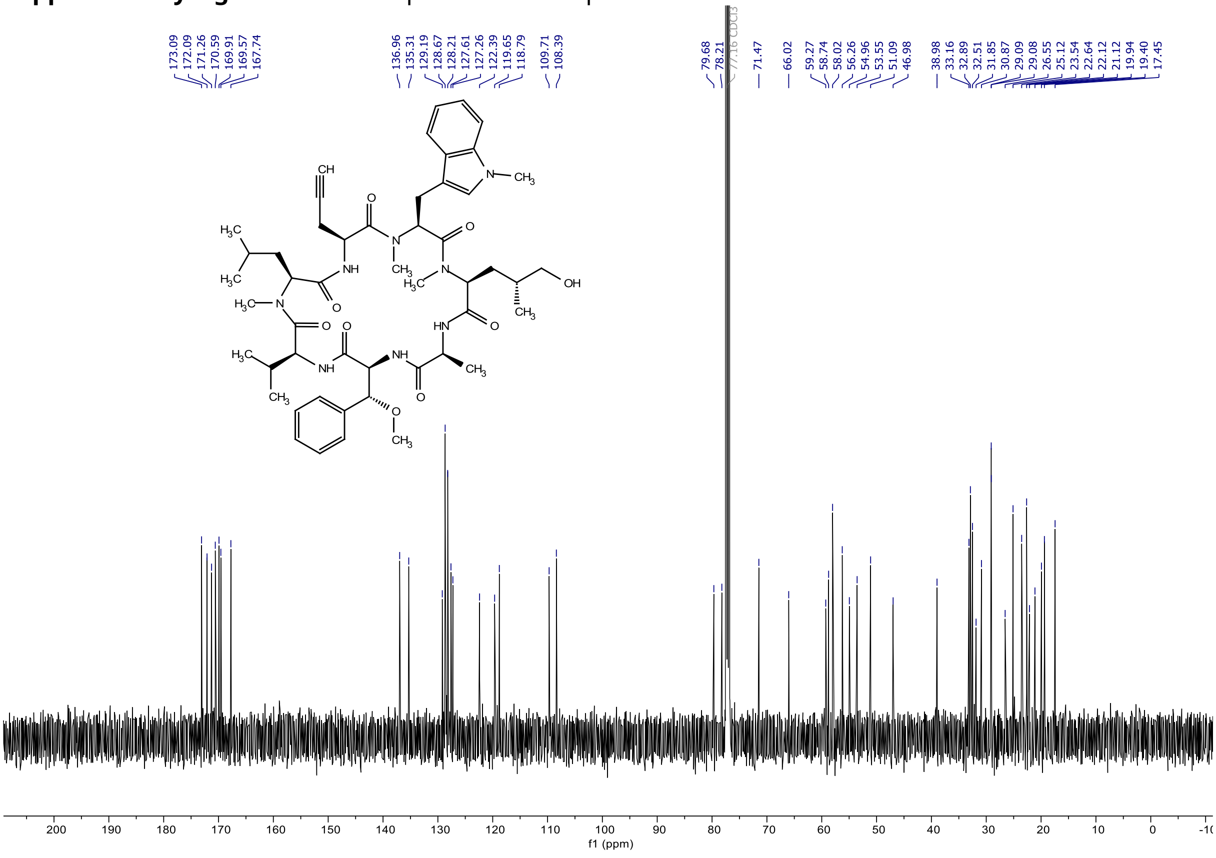

Supplementary Fig. 148. ^{1}H NMR spectrum of compound **21**

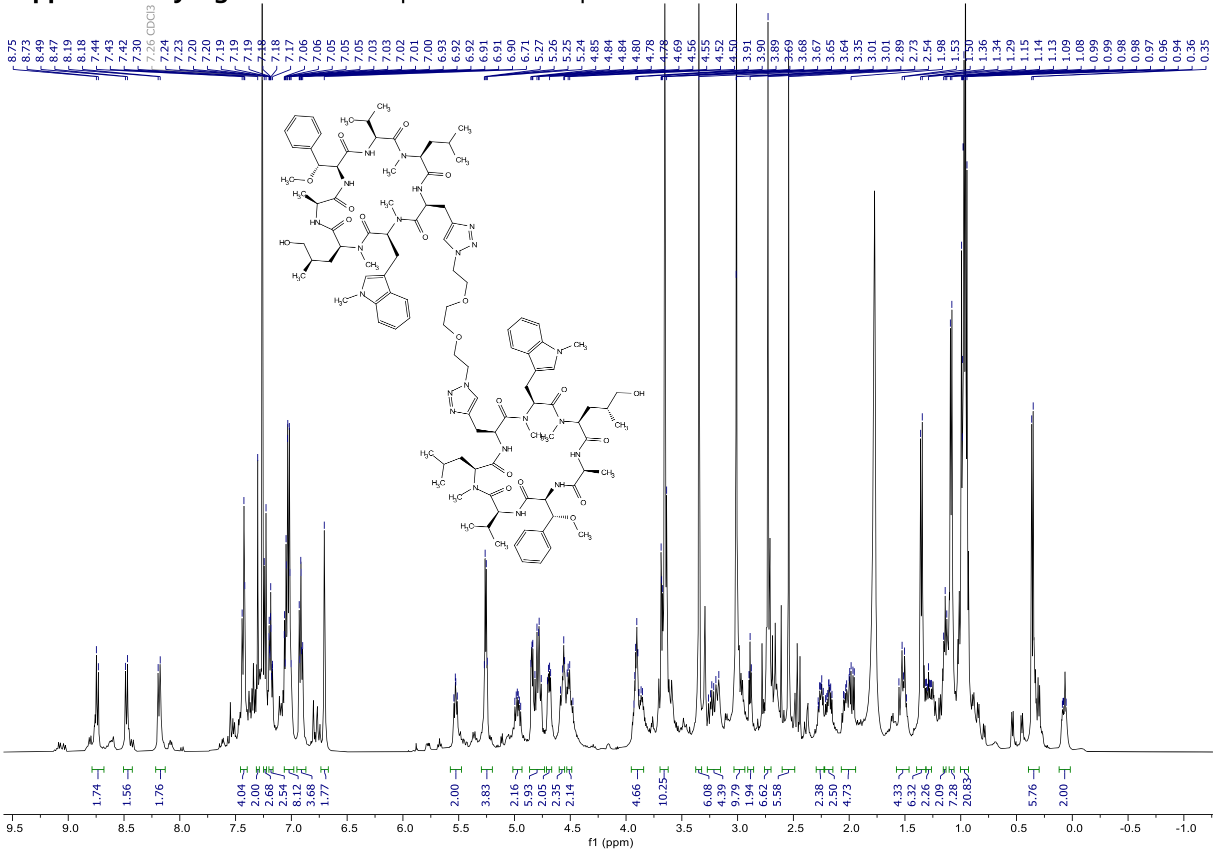

Supplementary Fig. 149. ^{13}C NMR spectrum of compound **21**

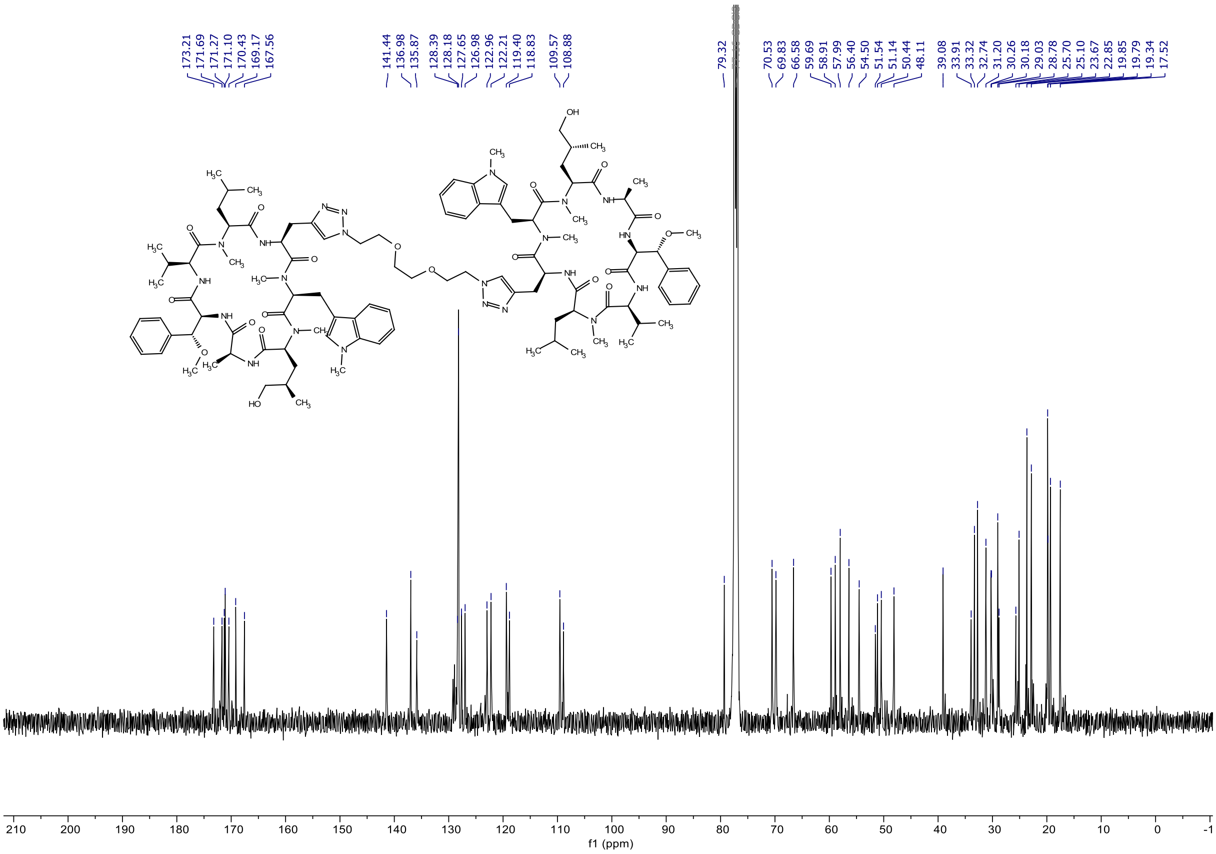

Supplementary Fig. 150. ^{1}H NMR spectrum of compound **22**

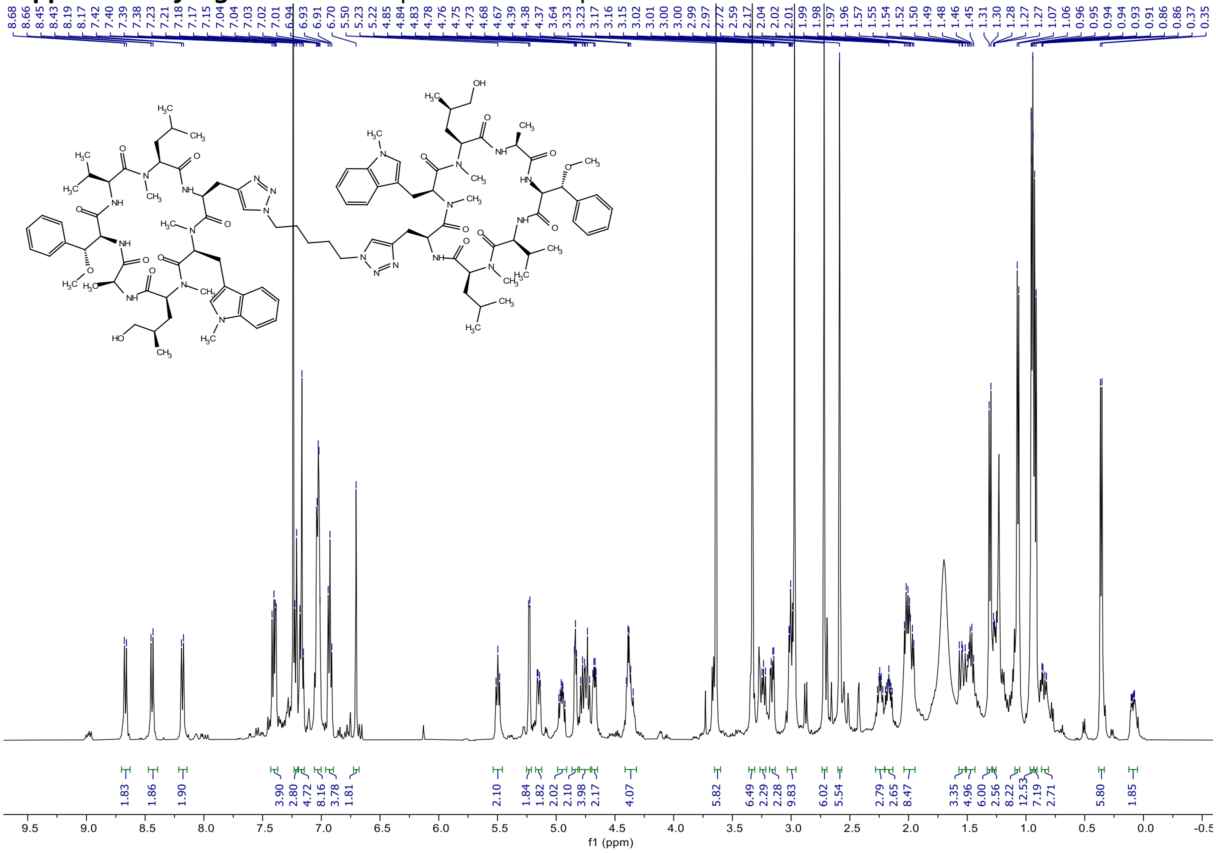

Supplementary Fig. 151. ^{13}C NMR spectrum of compound **22**

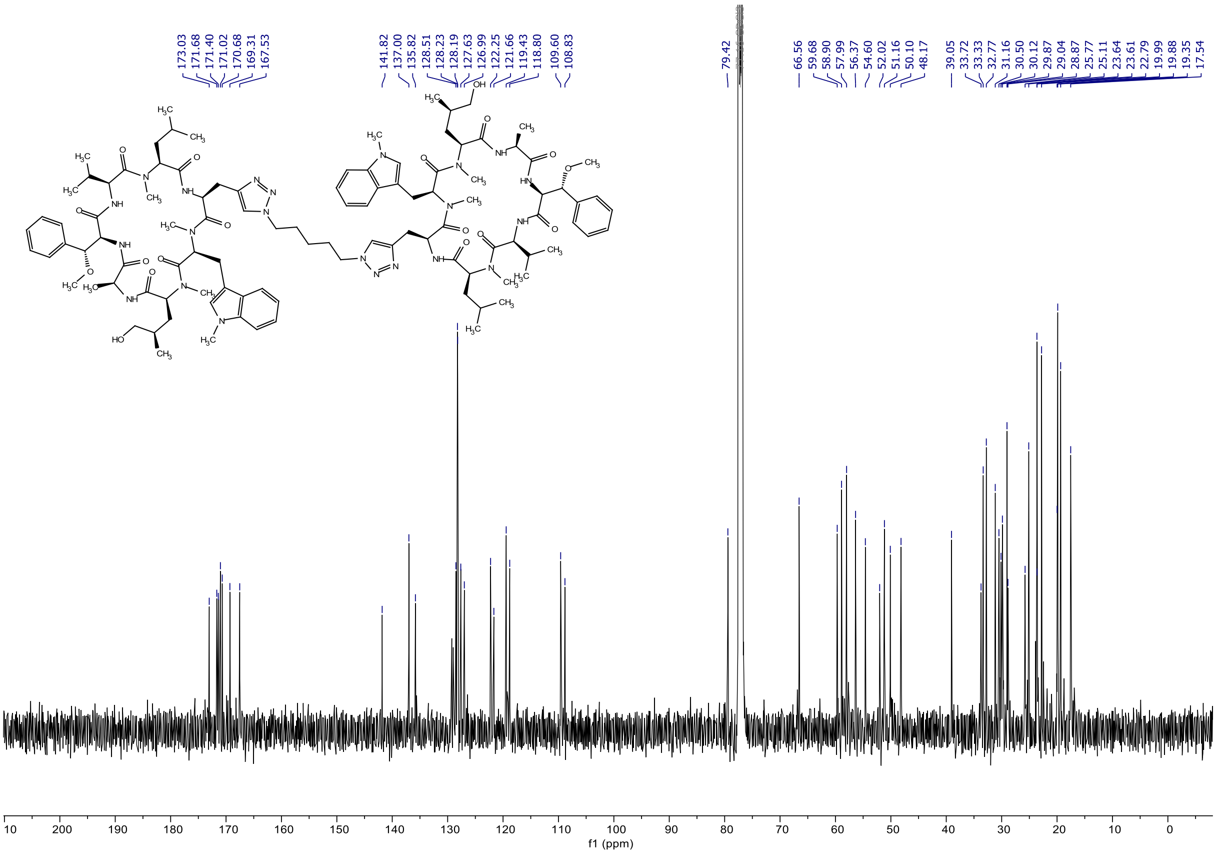

Supplementary Fig. 152. ^{1}H NMR spectrum of compound **25**

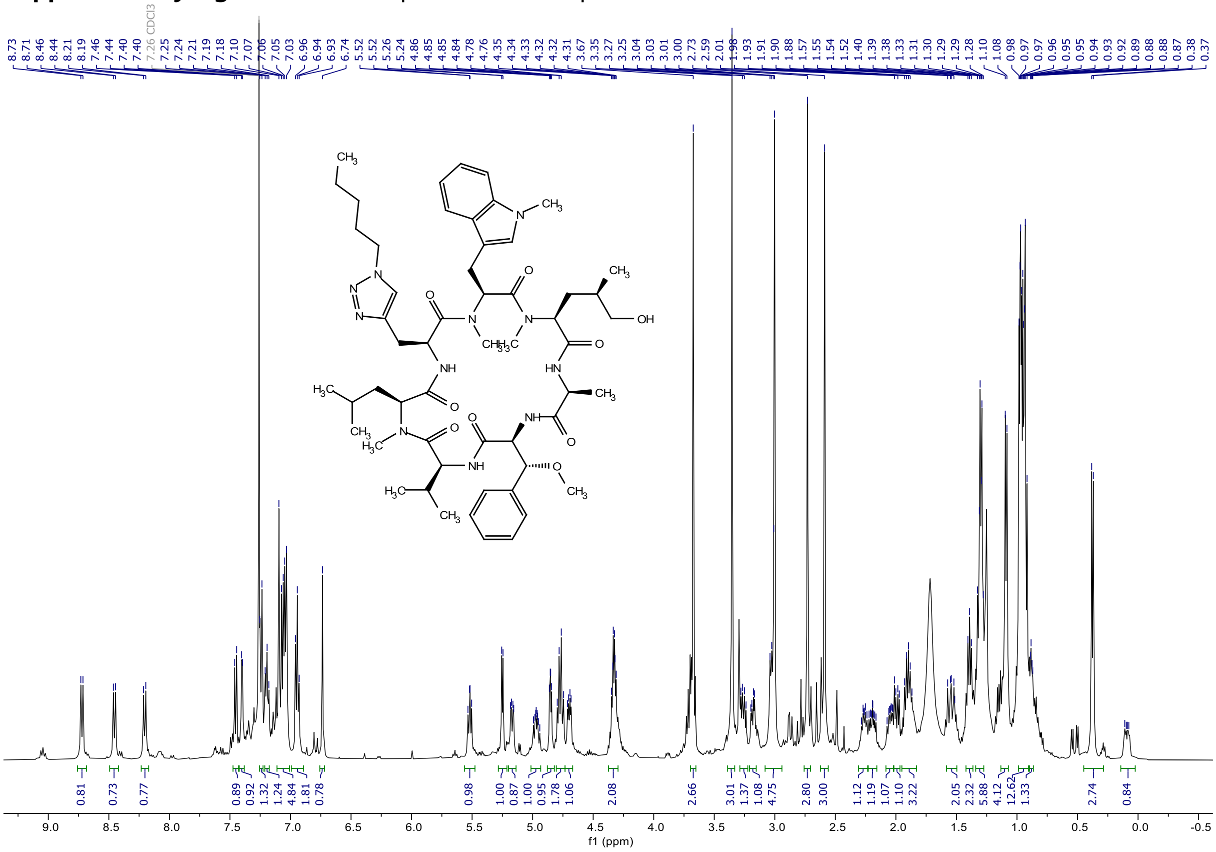

Supplementary Fig. 153. ^{13}C NMR spectrum of compound **25**

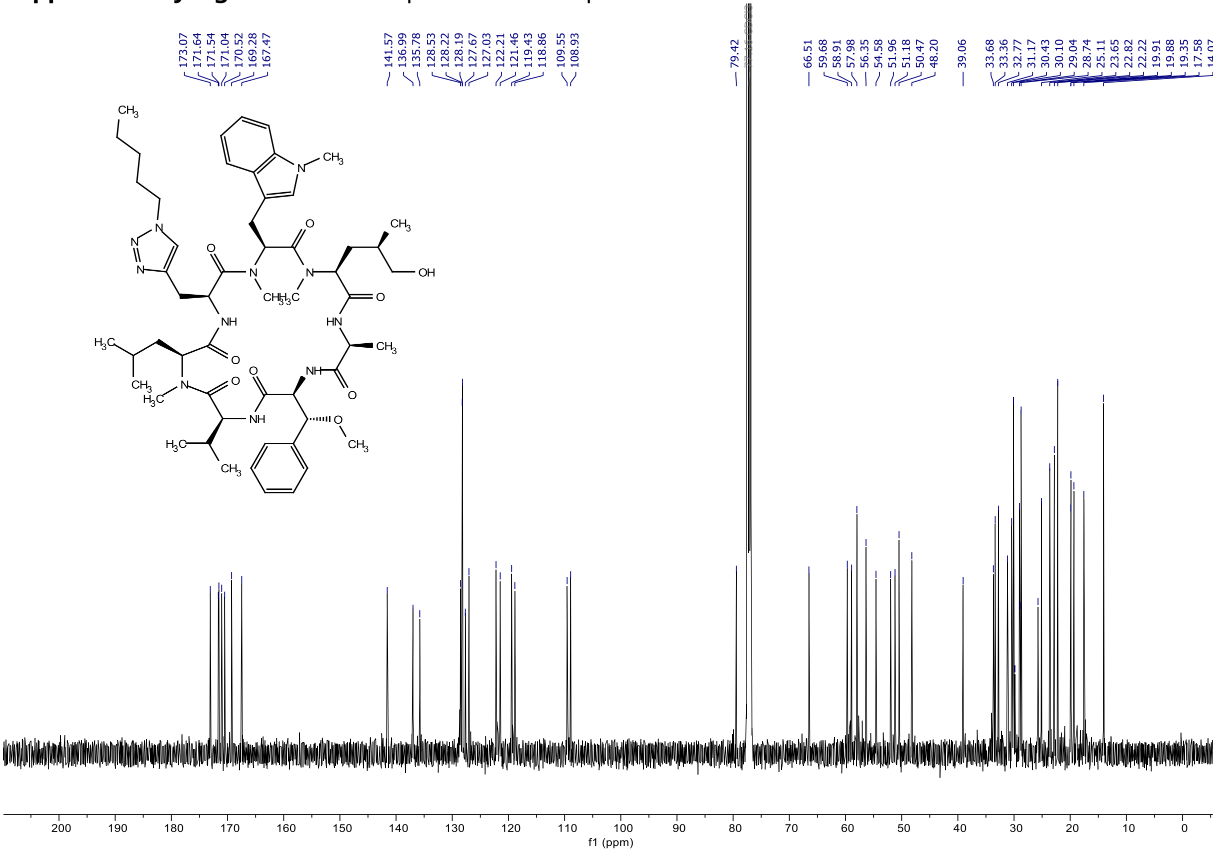

Supplementary Fig. 154. ^{1}H NMR spectrum of compound **SI-53**

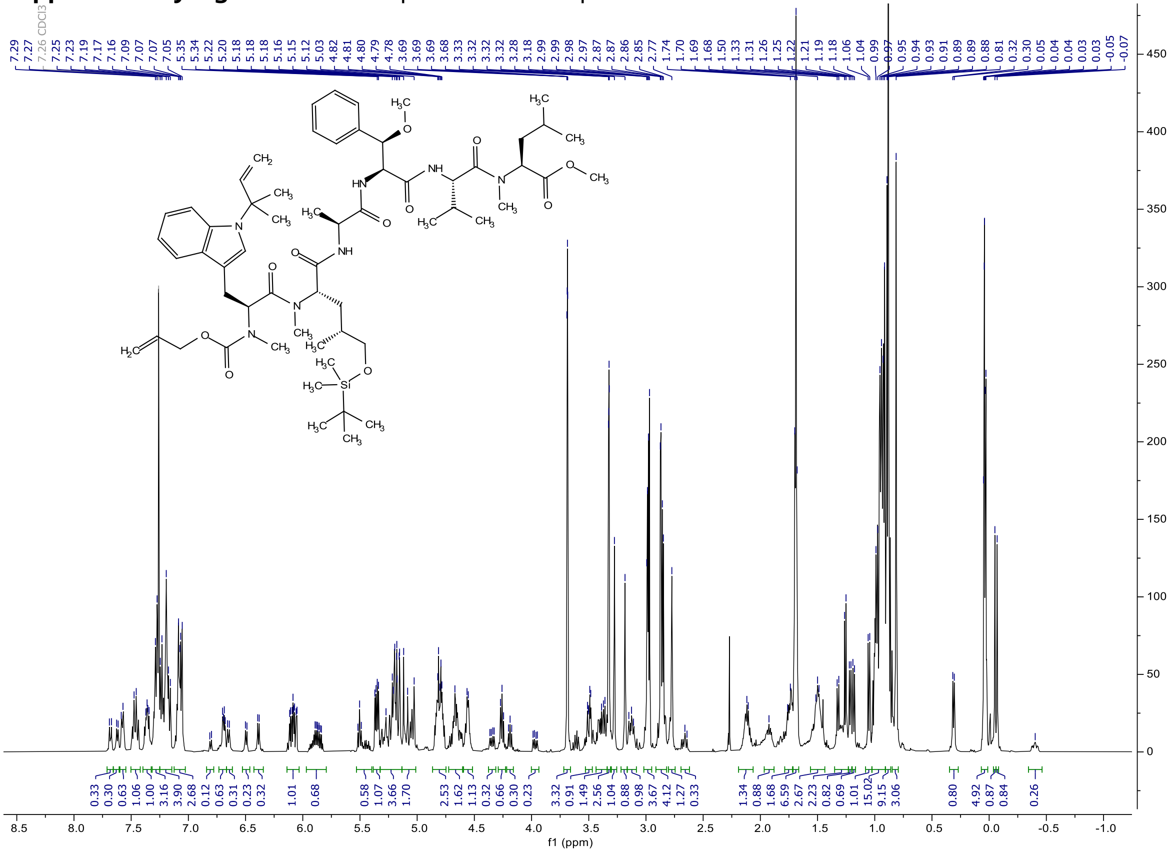

Supplementary Fig. 155. ^{13}C NMR spectrum of compound **SI-53**

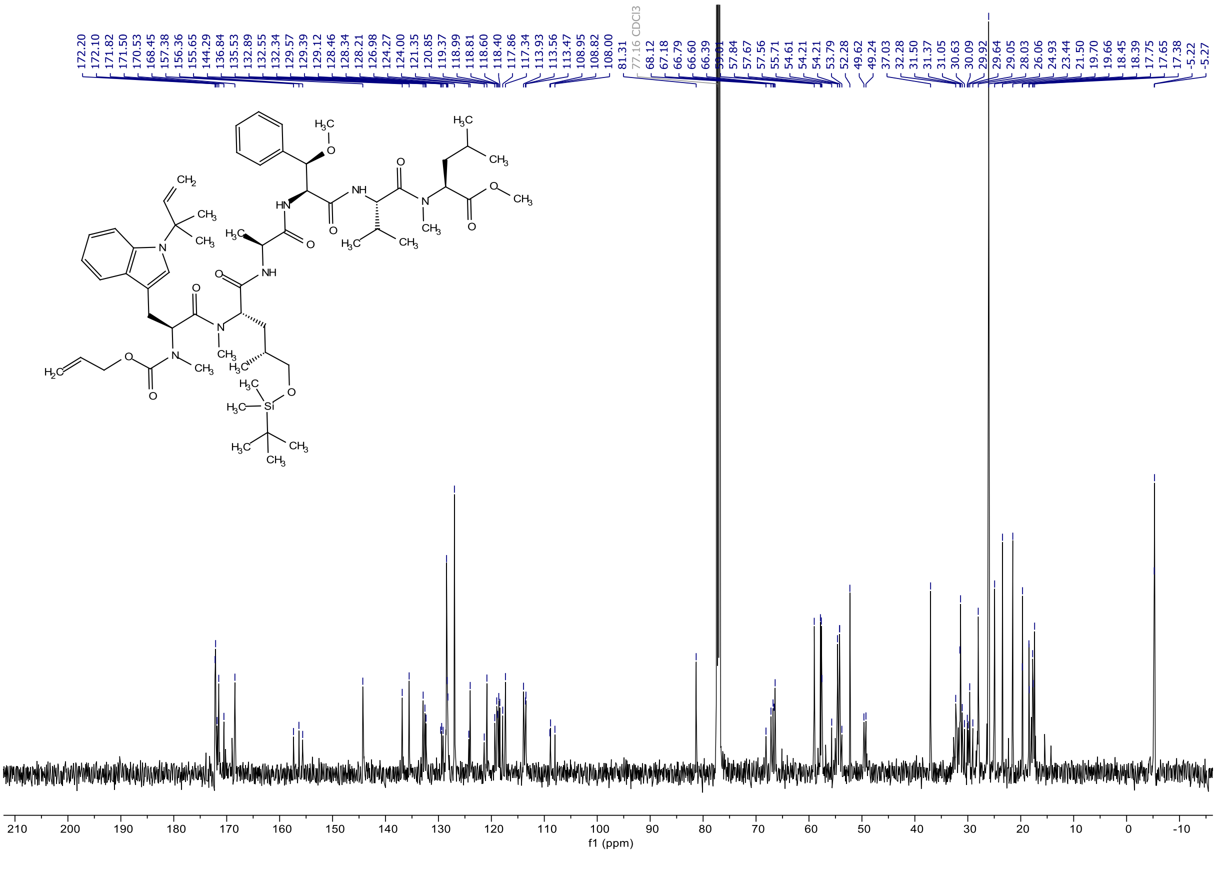

Supplementary Fig. 156. ^{1}H NMR spectrum of compound **SI-54**

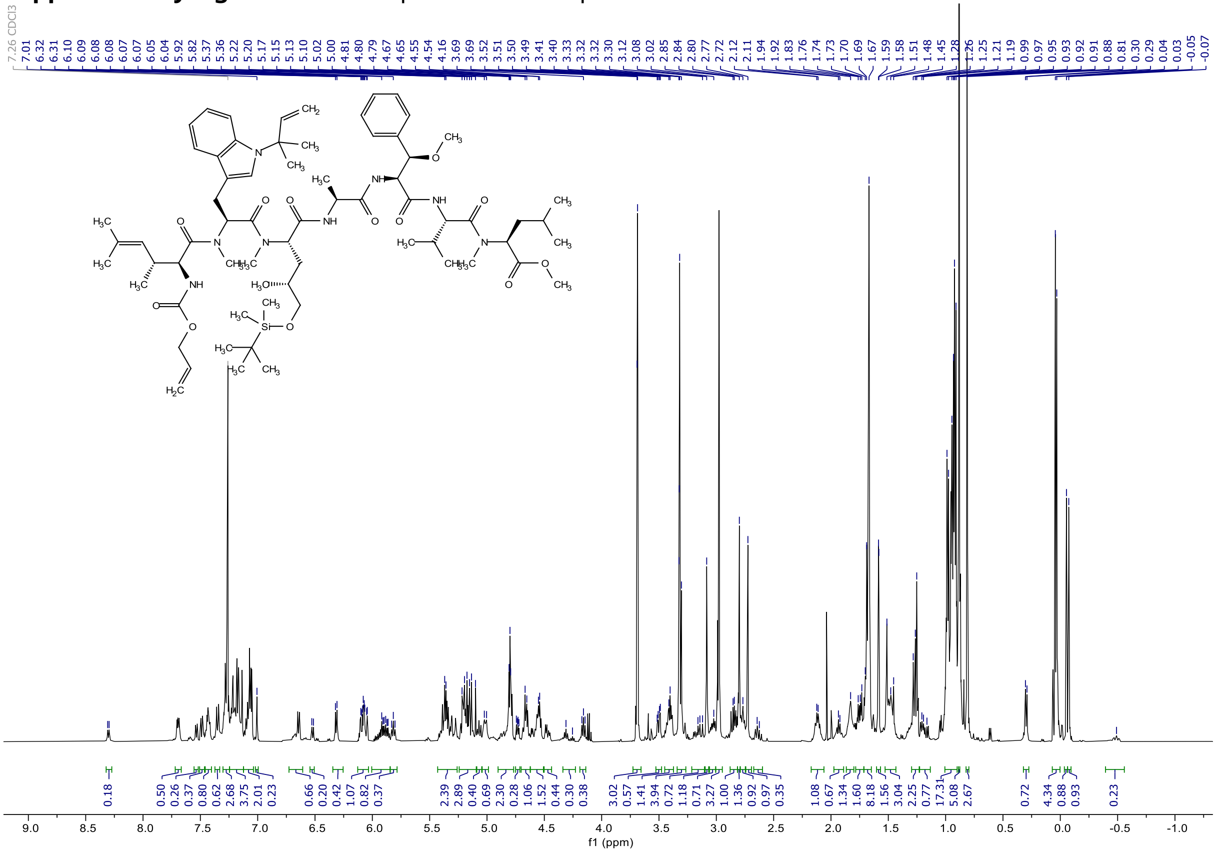

Supplementary Fig. 157. ^{13}C NMR spectrum of compound **SI-54**

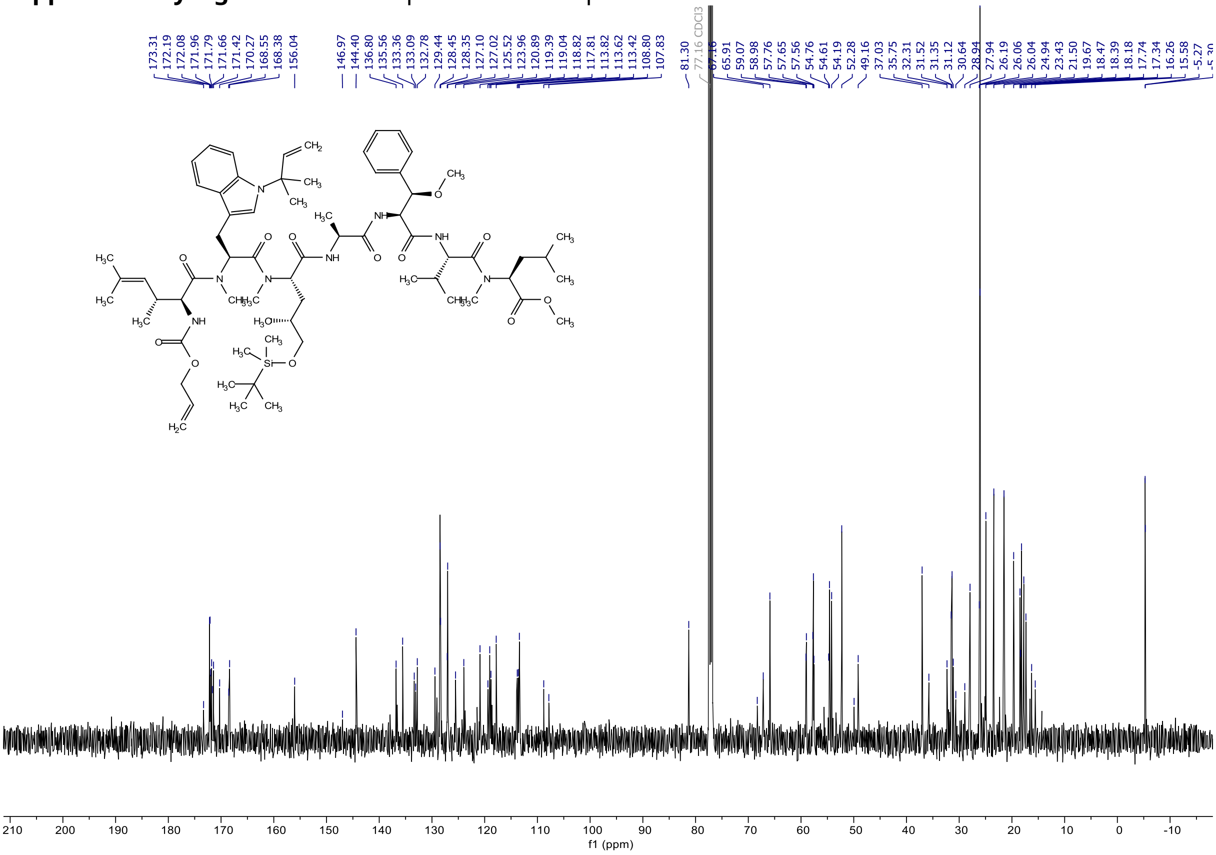

Supplementary Fig. 158. ^{1}H NMR spectrum of compound **23**

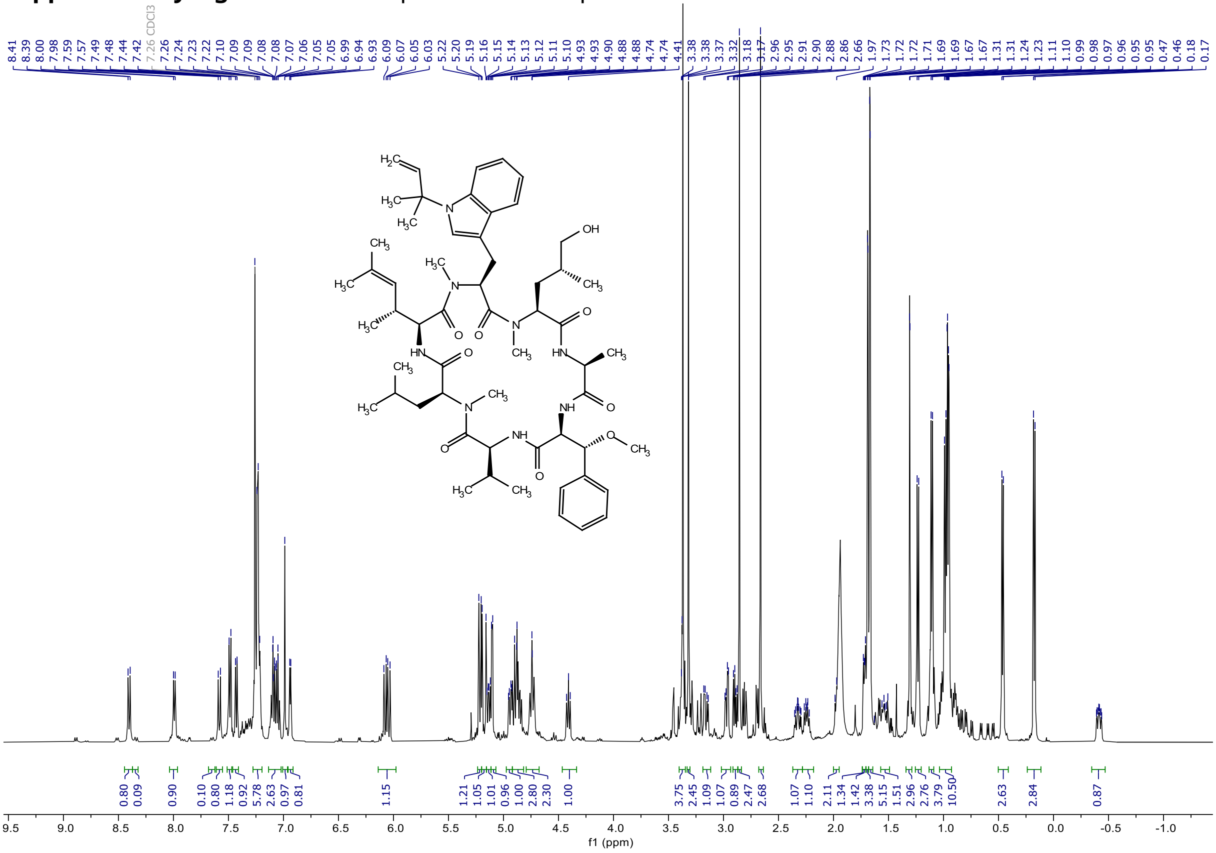

Supplementary Fig. 159. ^{13}C NMR spectrum of compound **23**

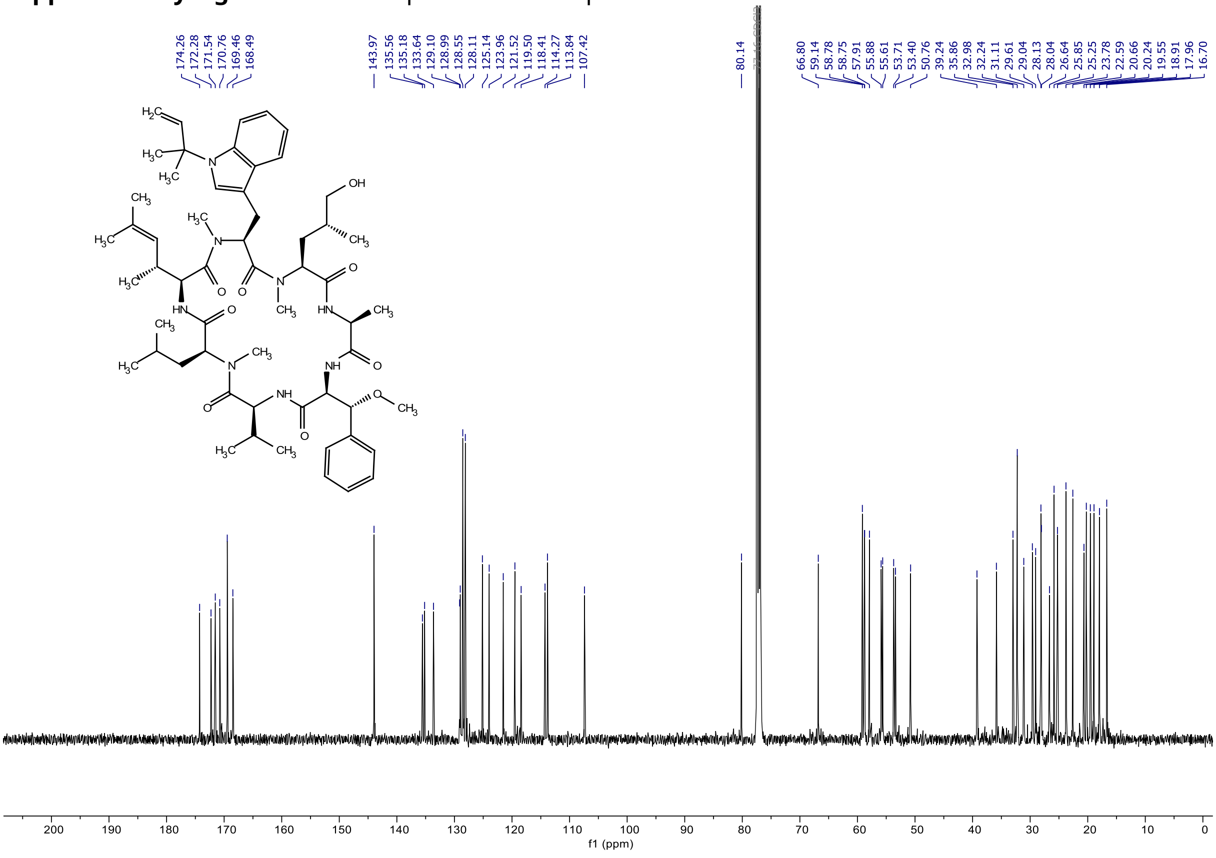

Supplementary Fig. 160. ^{1}H NMR spectrum of compound **SI-55**

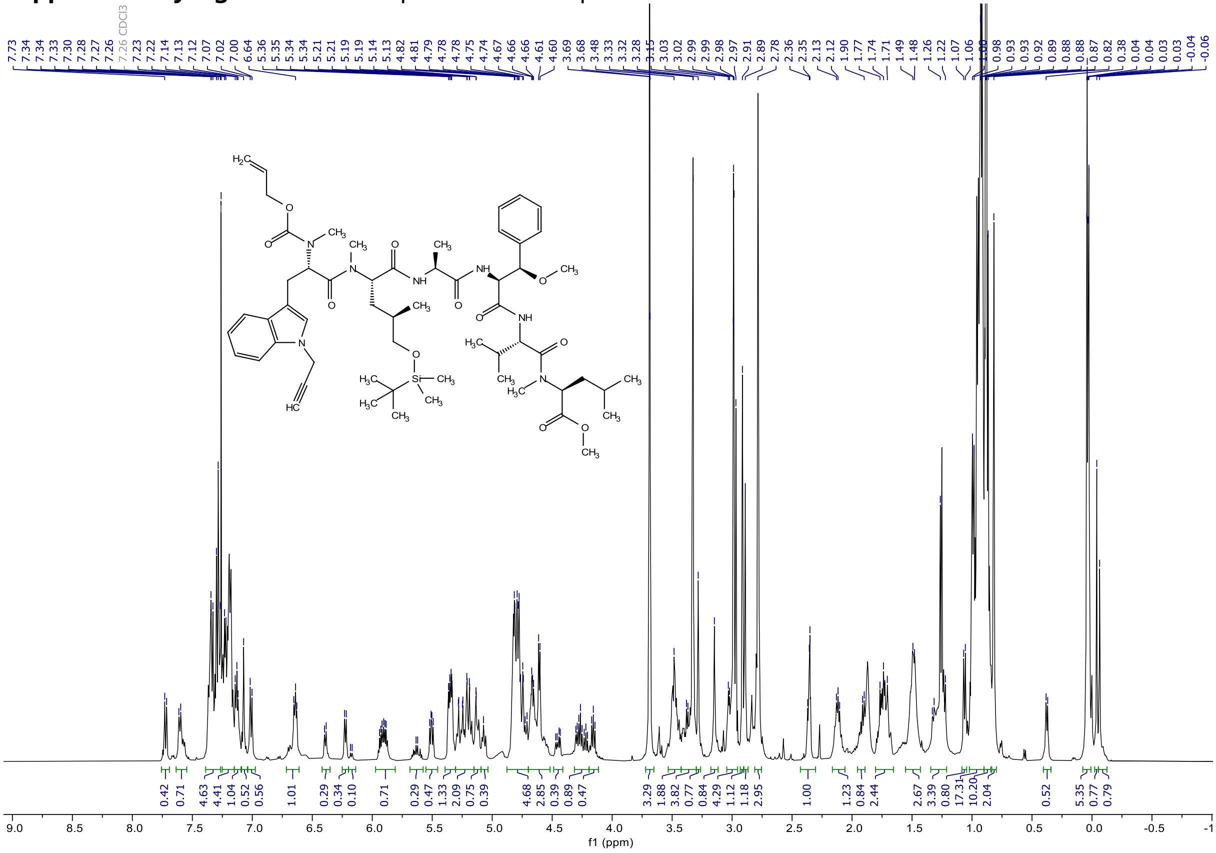

Supplementary Fig. 161. ^{13}C NMR spectrum of compound **SI-55**

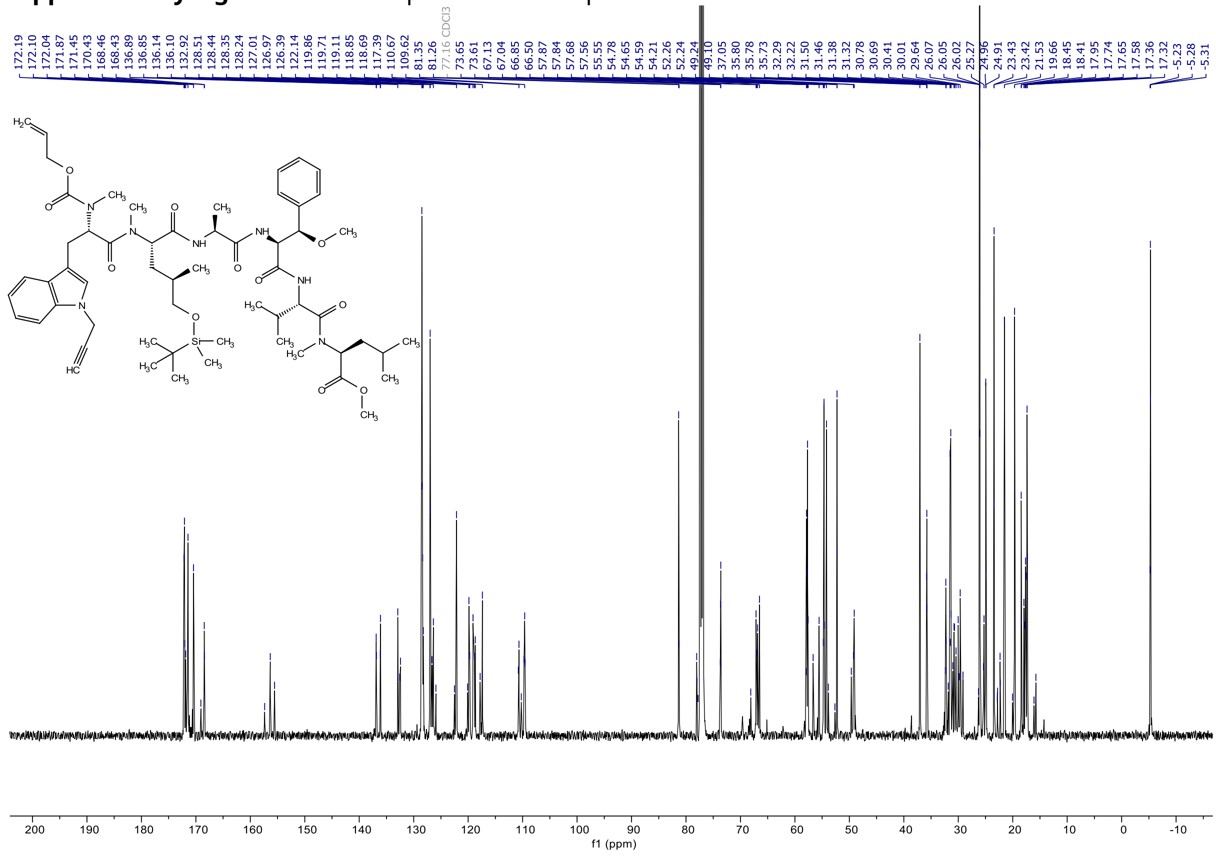

Supplementary Fig. 162. ^{1}H NMR spectrum of compound **SI-56**

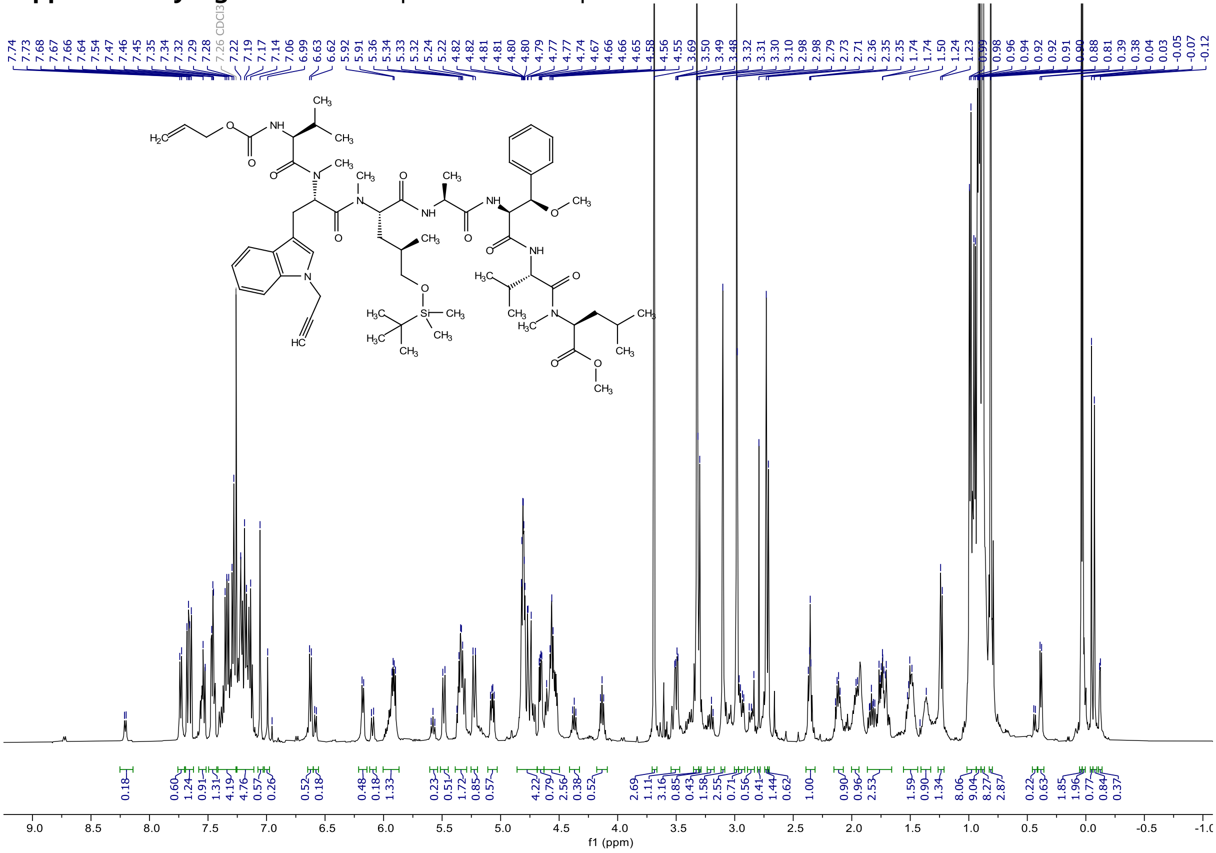

Supplementary Fig. 163. ^{13}C NMR spectrum of compound **SI-56**

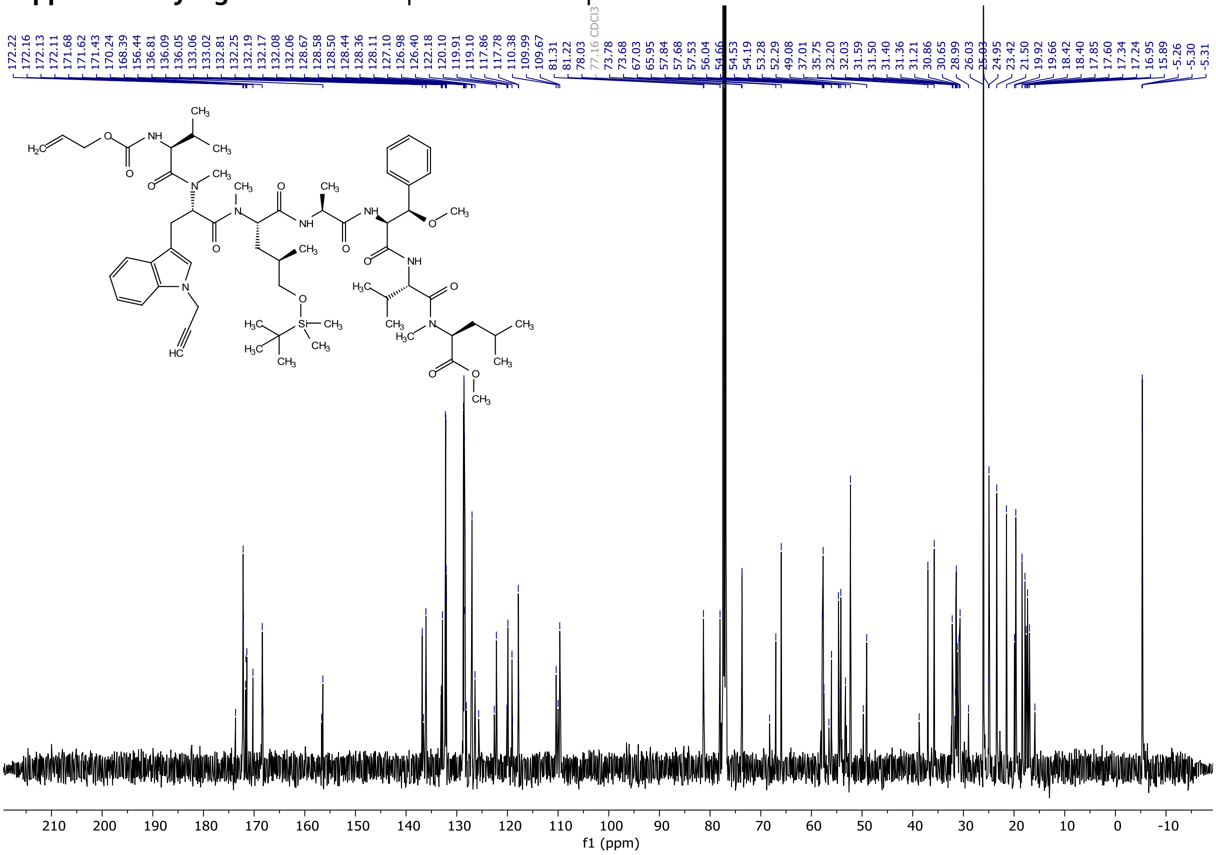

Supplementary Fig. 164. ^{1}H NMR spectrum of compound **26**

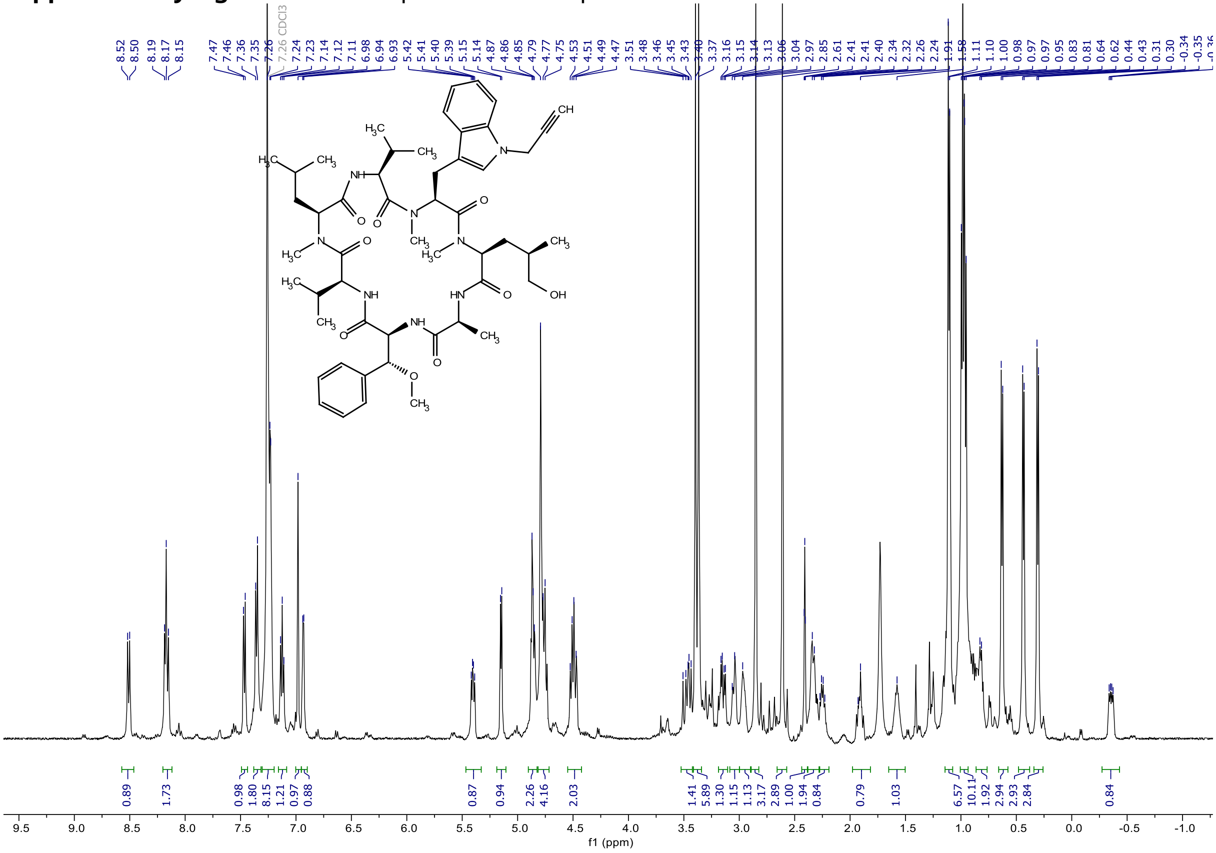

Supplementary Fig. 165. ^{13}C NMR spectrum of compound **26**

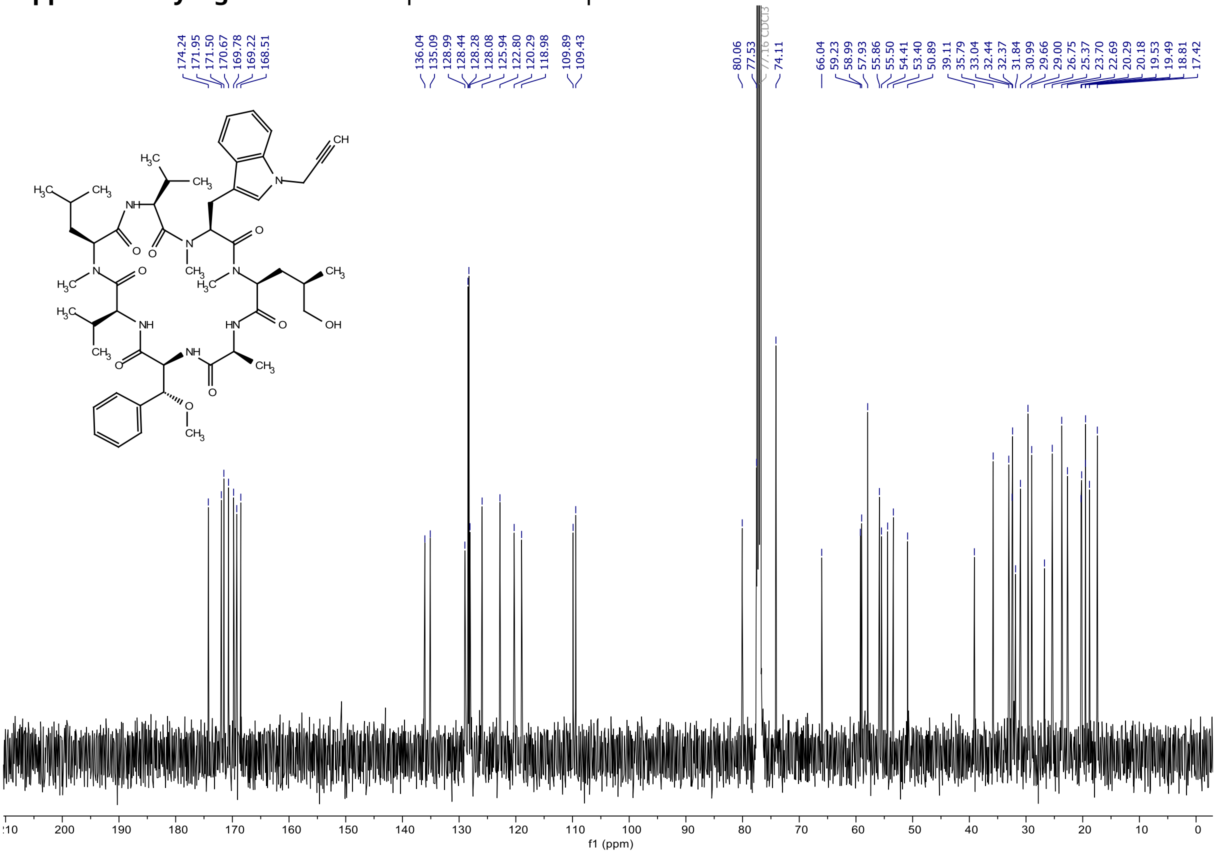

Supplementary Fig. 166. ^{1}H NMR spectrum of compound **SI-57**

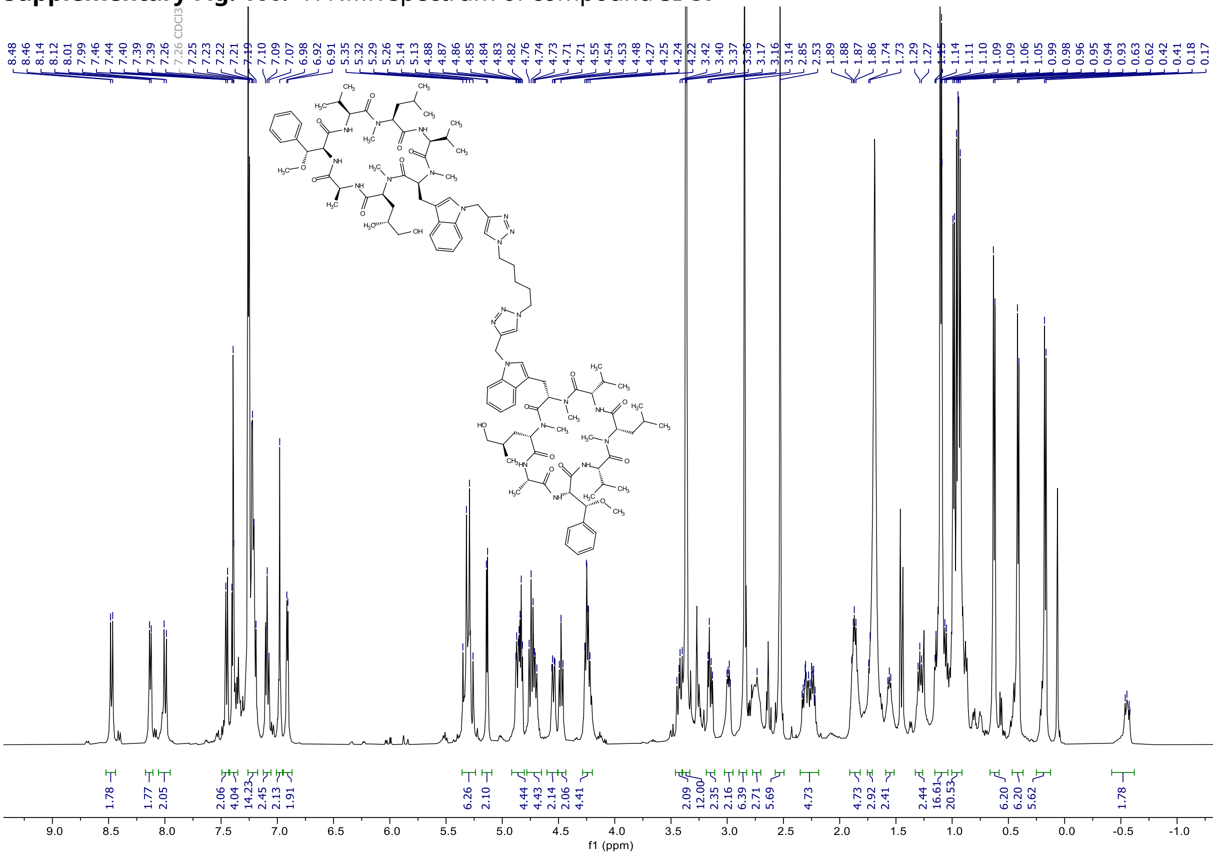

Supplementary Fig. 167. ^{13}C NMR spectrum of compound **SI-57**

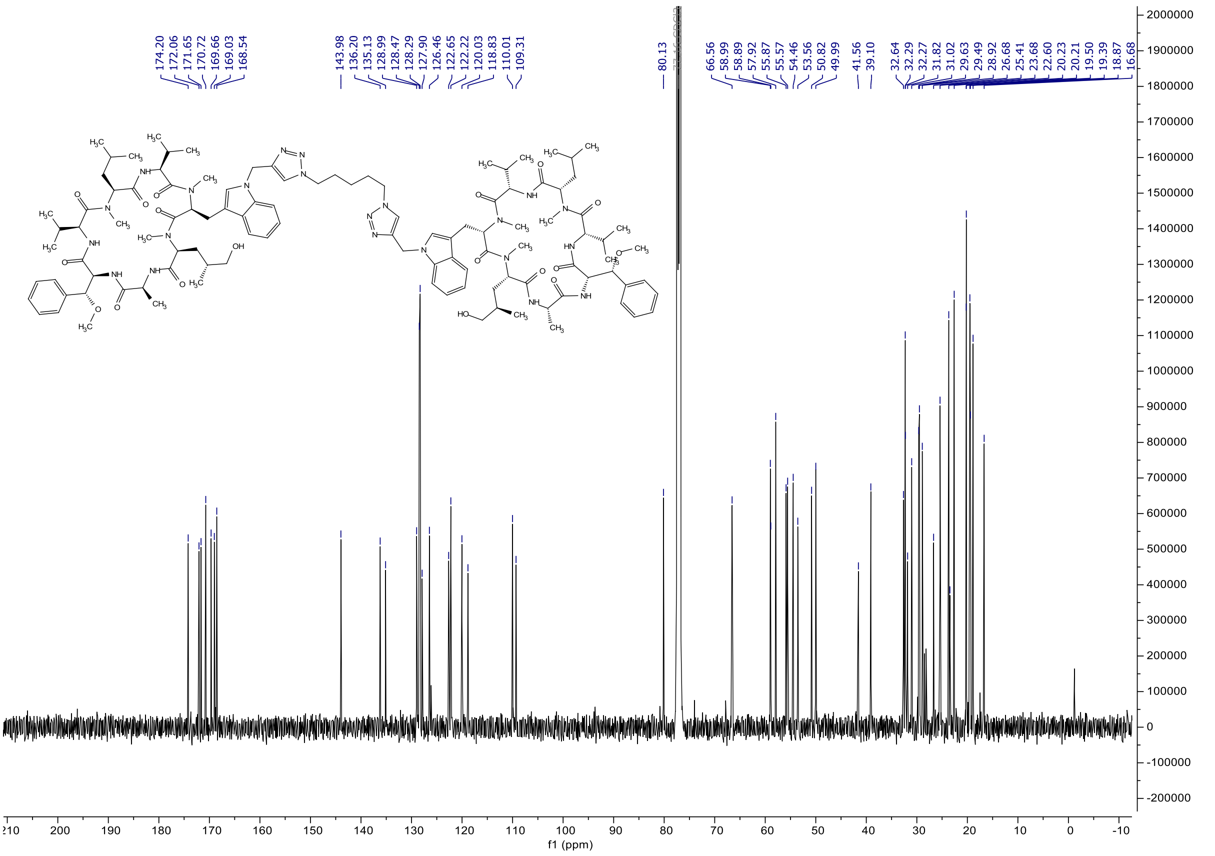

Supplementary Fig. 168. ^{1}H NMR spectrum of compound **27**

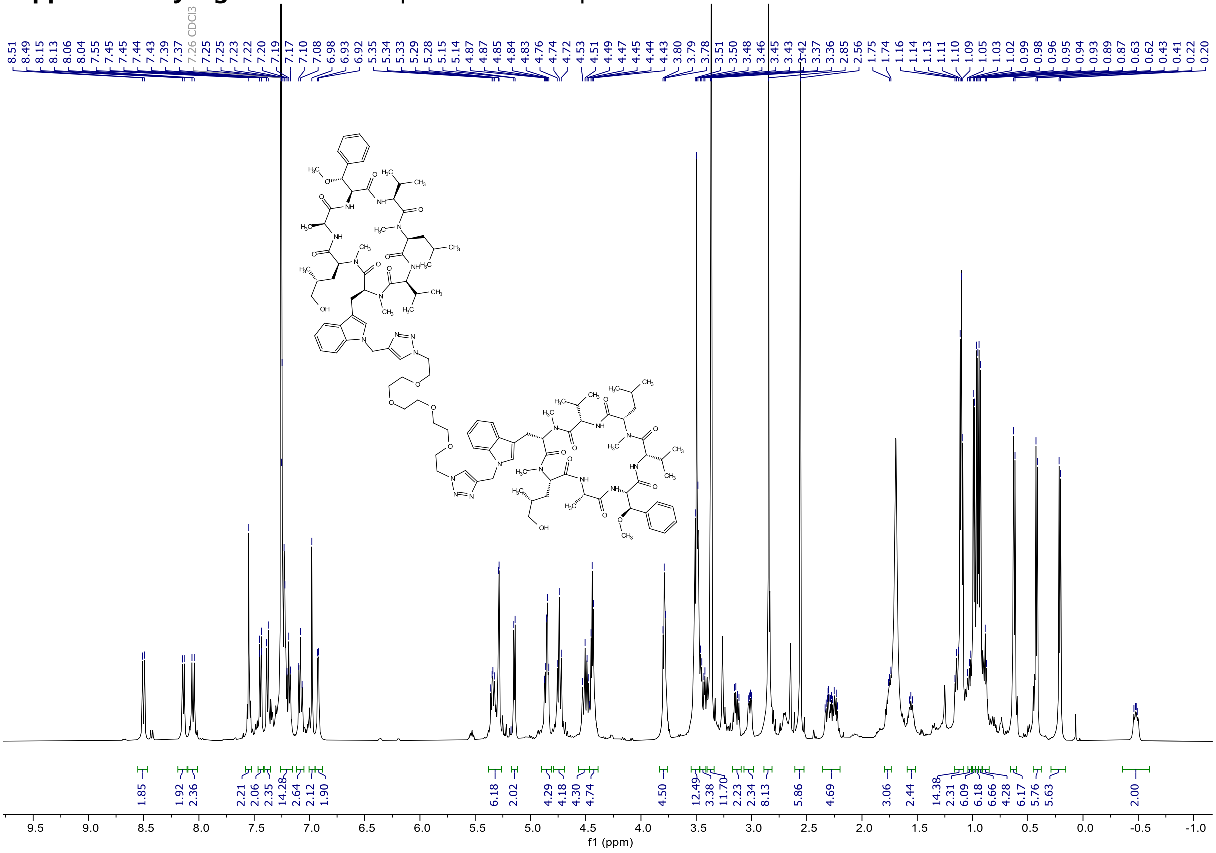

Supplementary Fig. 169. ^{13}C NMR spectrum of compound **27**

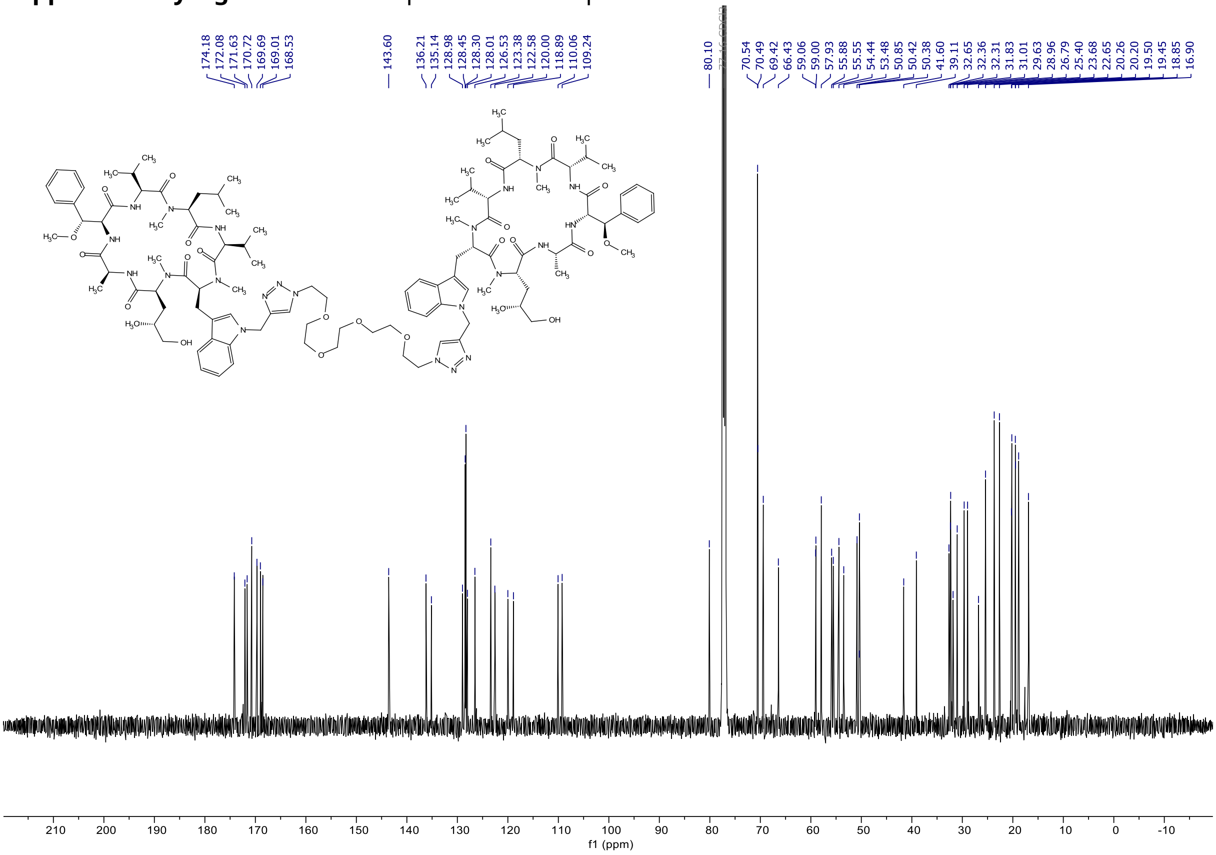

Supplementary Fig. 170. ^{1}H NMR spectrum of compound **SI-58**

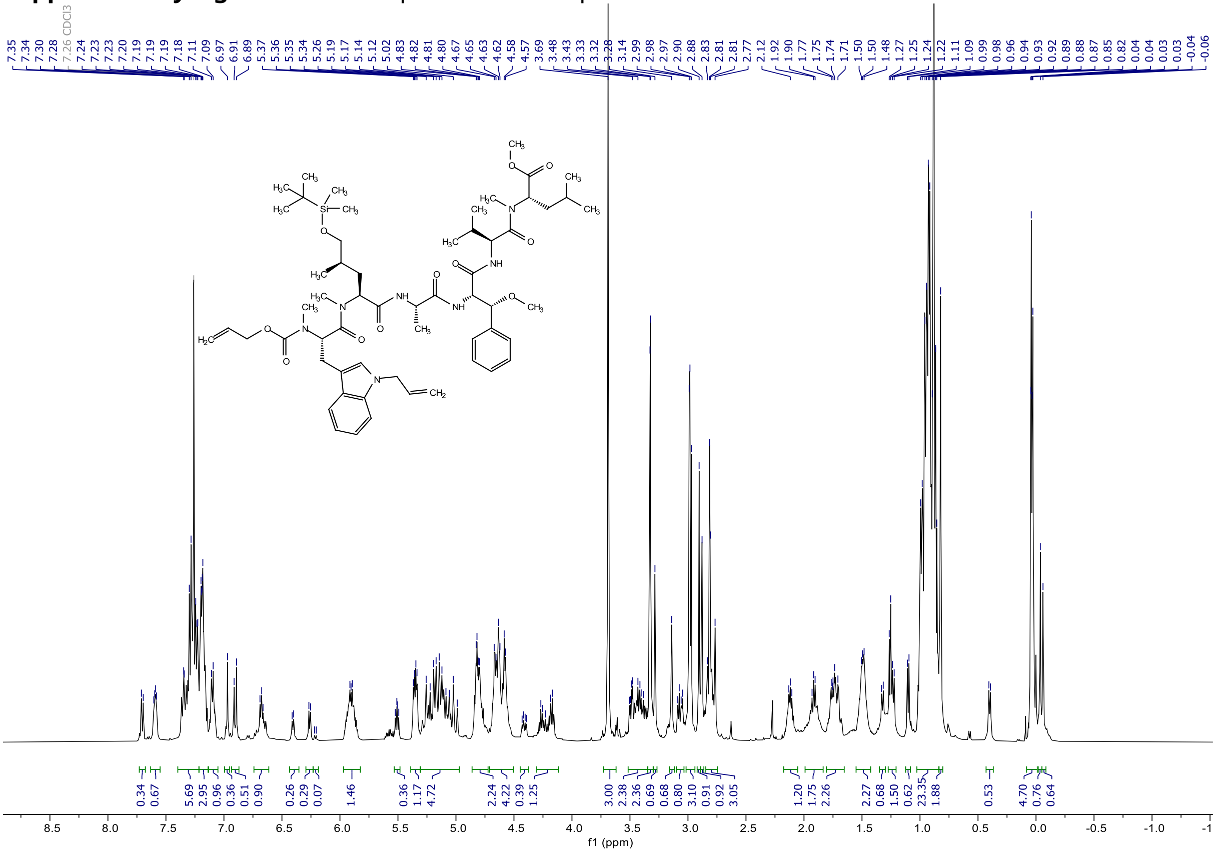

Supplementary Fig. 171. ^{13}C NMR spectrum of compound **SI-58**

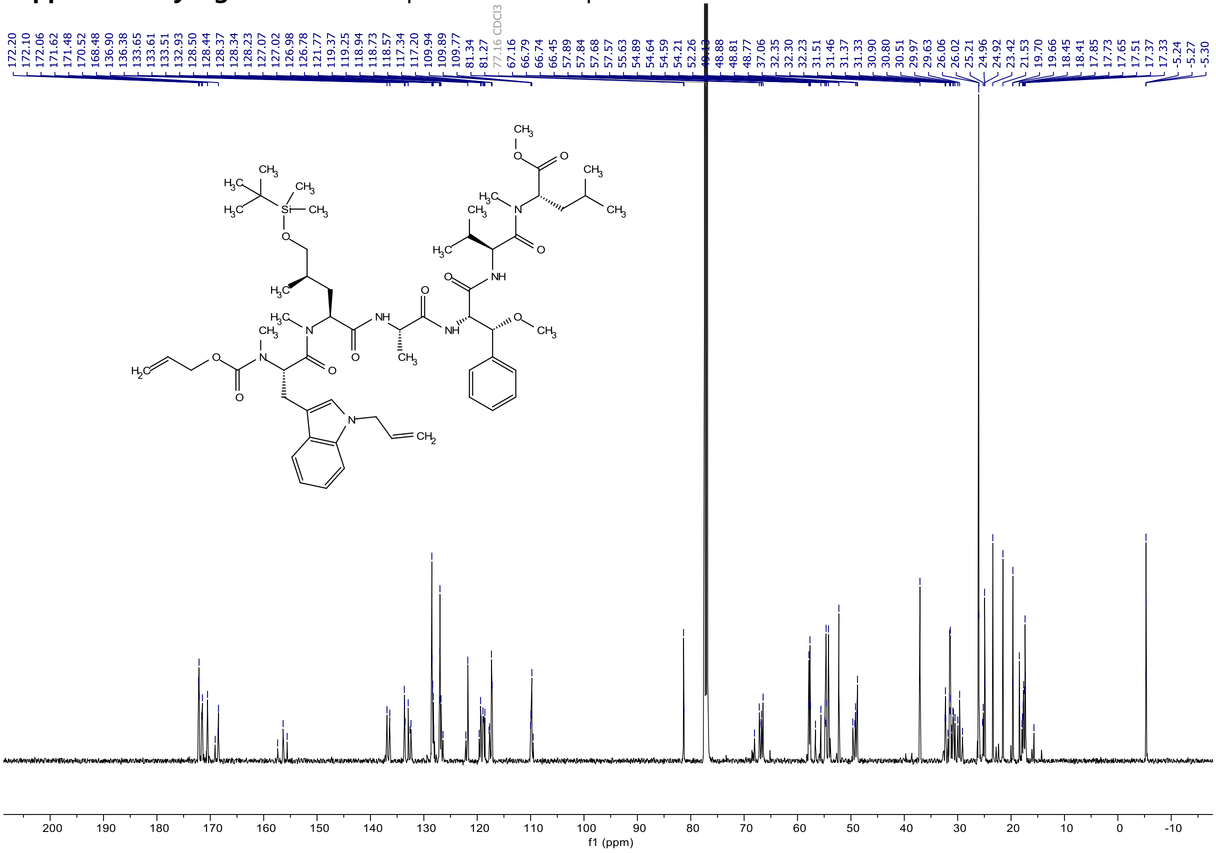

Supplementary Fig. 172. ^{1}H NMR spectrum of compound **SI-59**

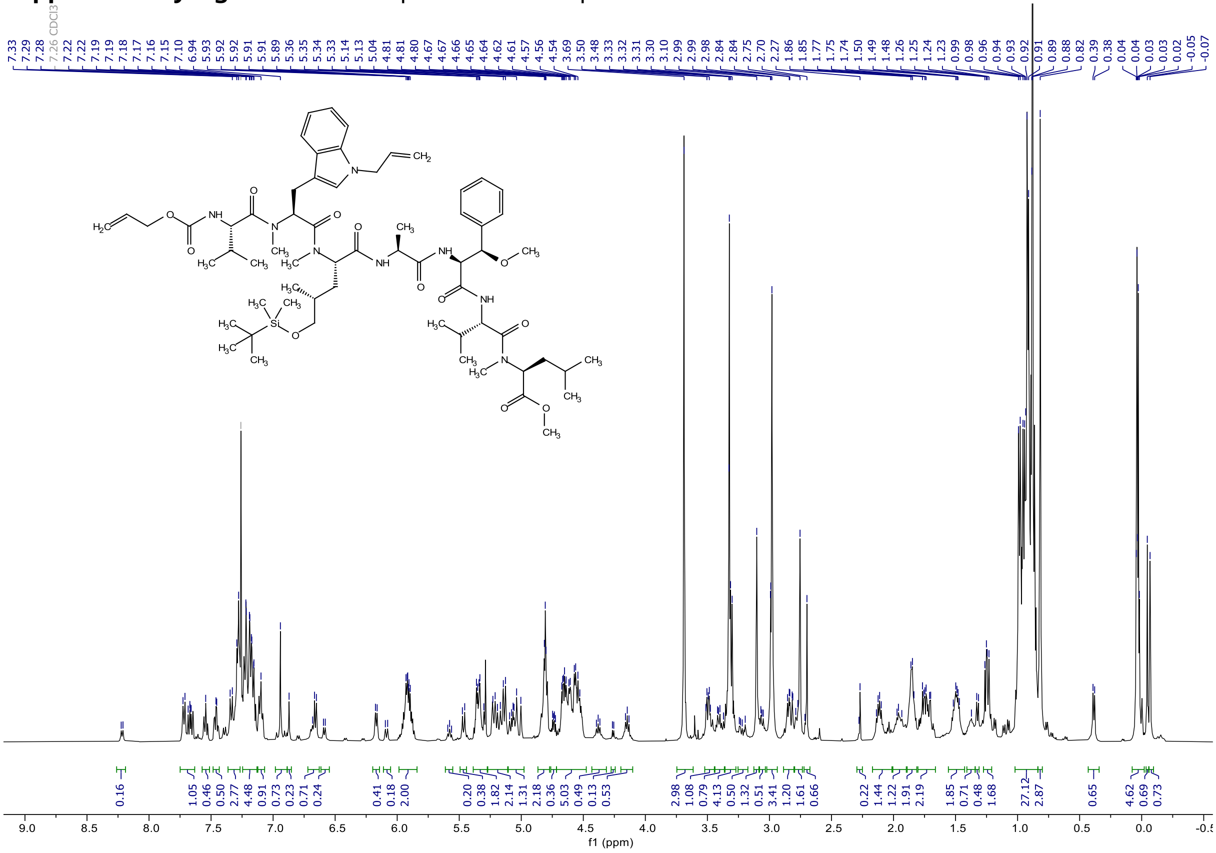

Supplementary Fig. 173. ^{13}C NMR spectrum of compound **SI-59**

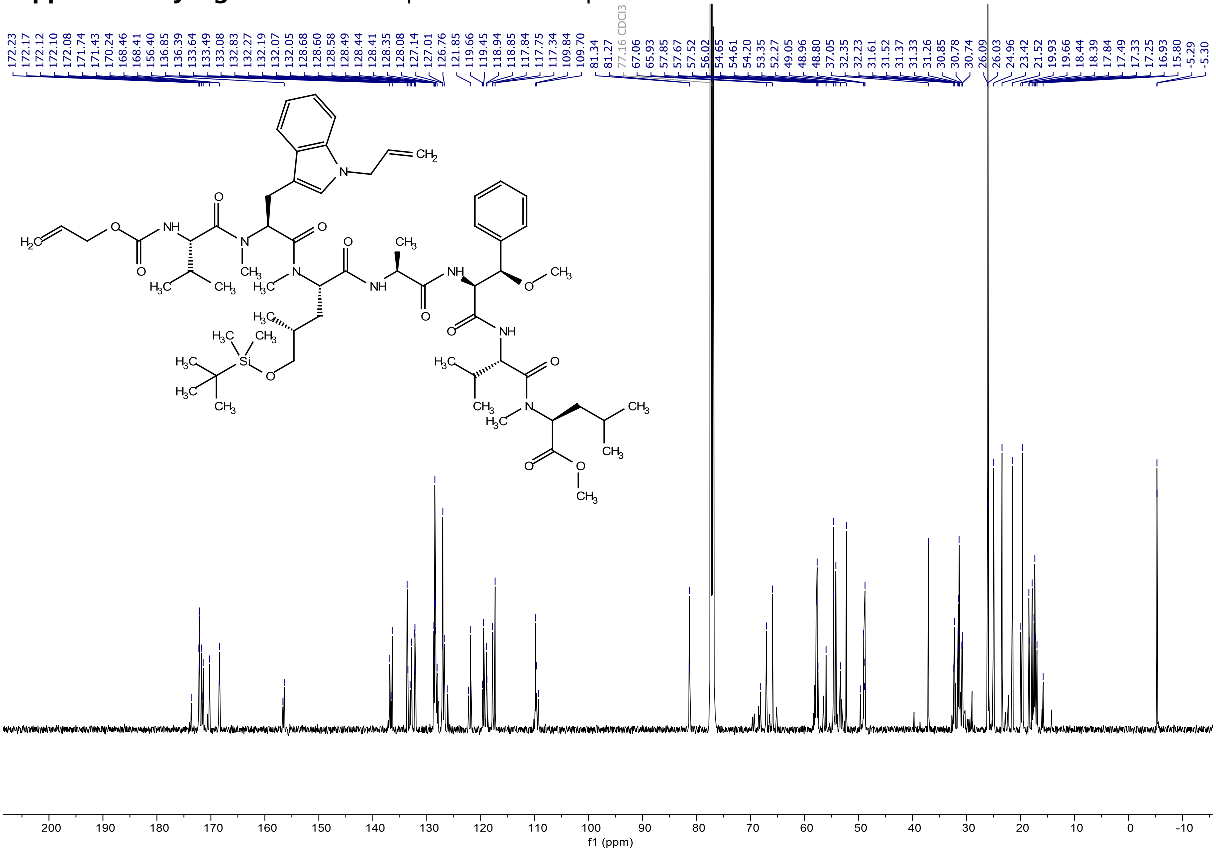

Supplementary Fig. 174. ^{1}H NMR spectrum of compound **SI-60**

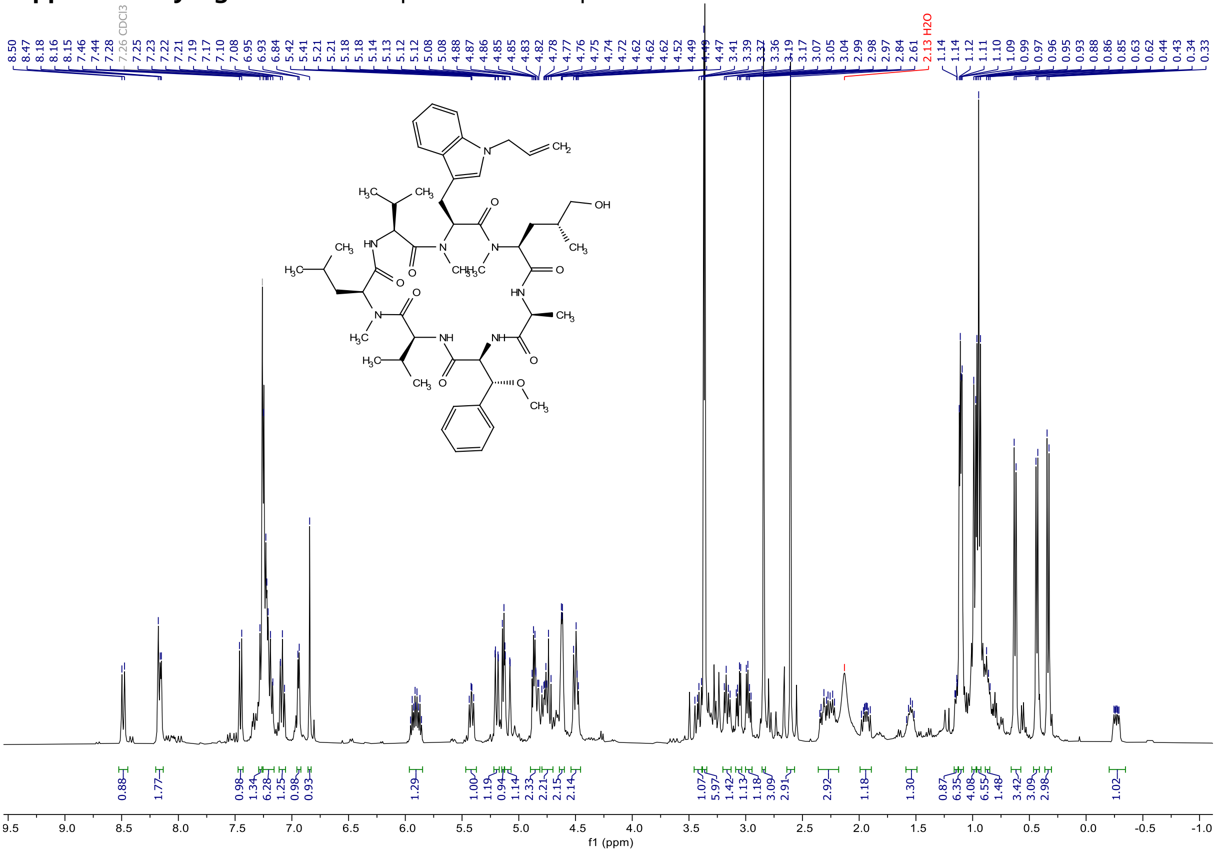

Supplementary Fig. 175. ^{13}C NMR spectrum of compound **SI-60**

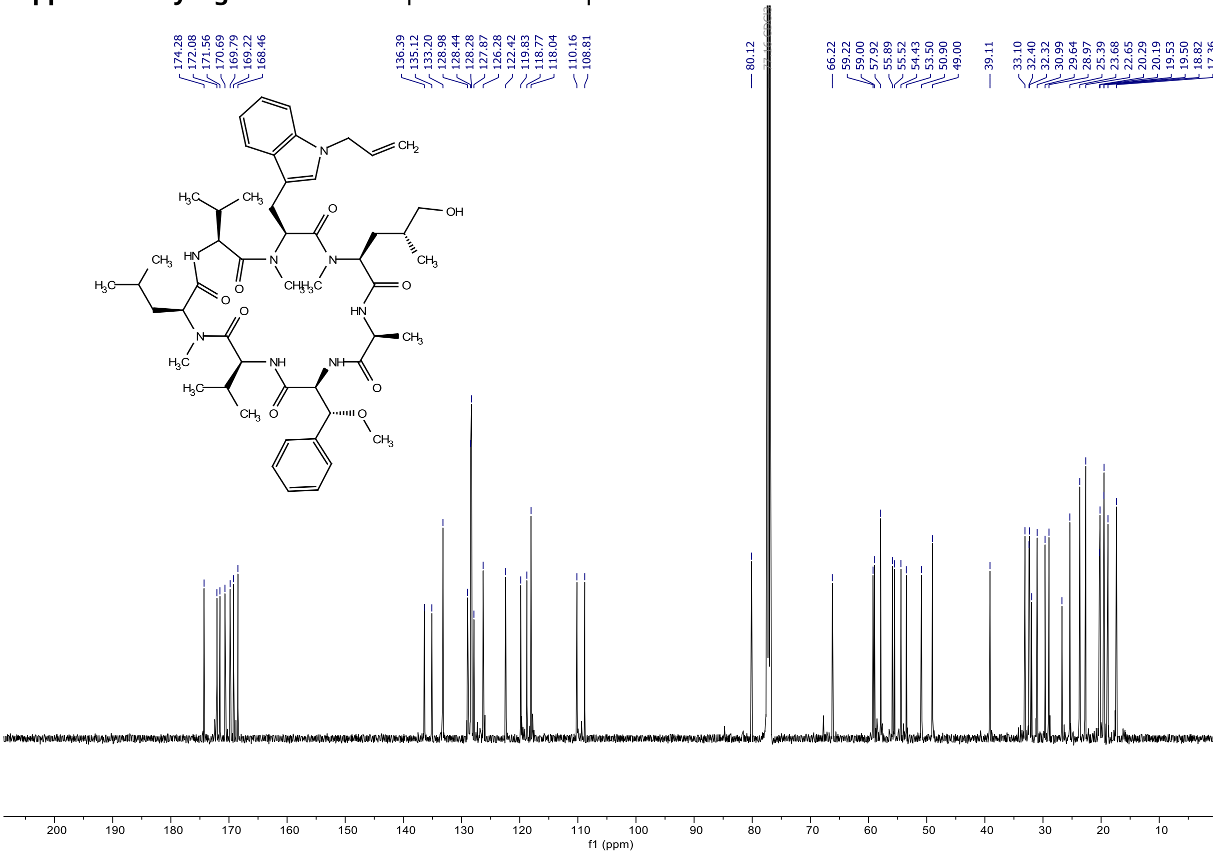

Supplementary Fig. 176. ^{1}H NMR spectrum of compound **28**

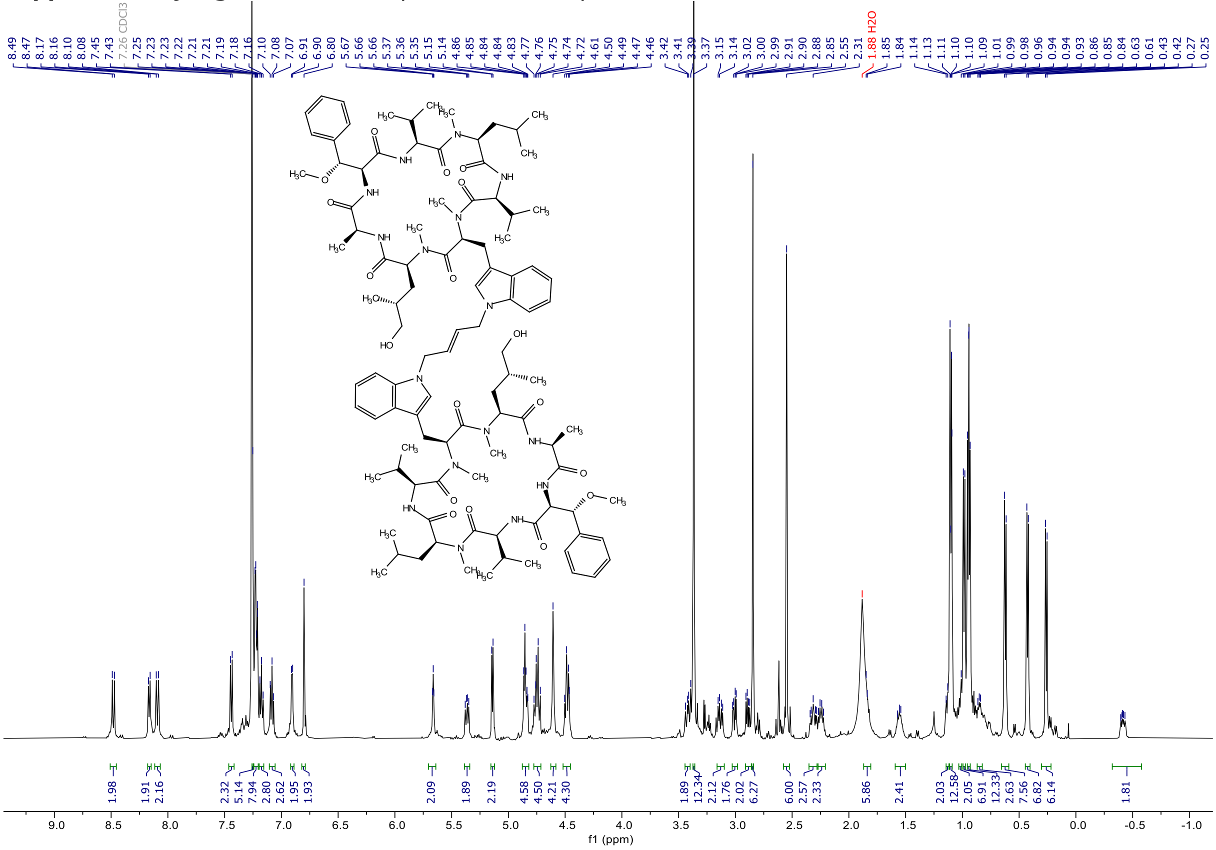

Supplementary Fig. 177. ^{13}C NMR spectrum of compound **28**

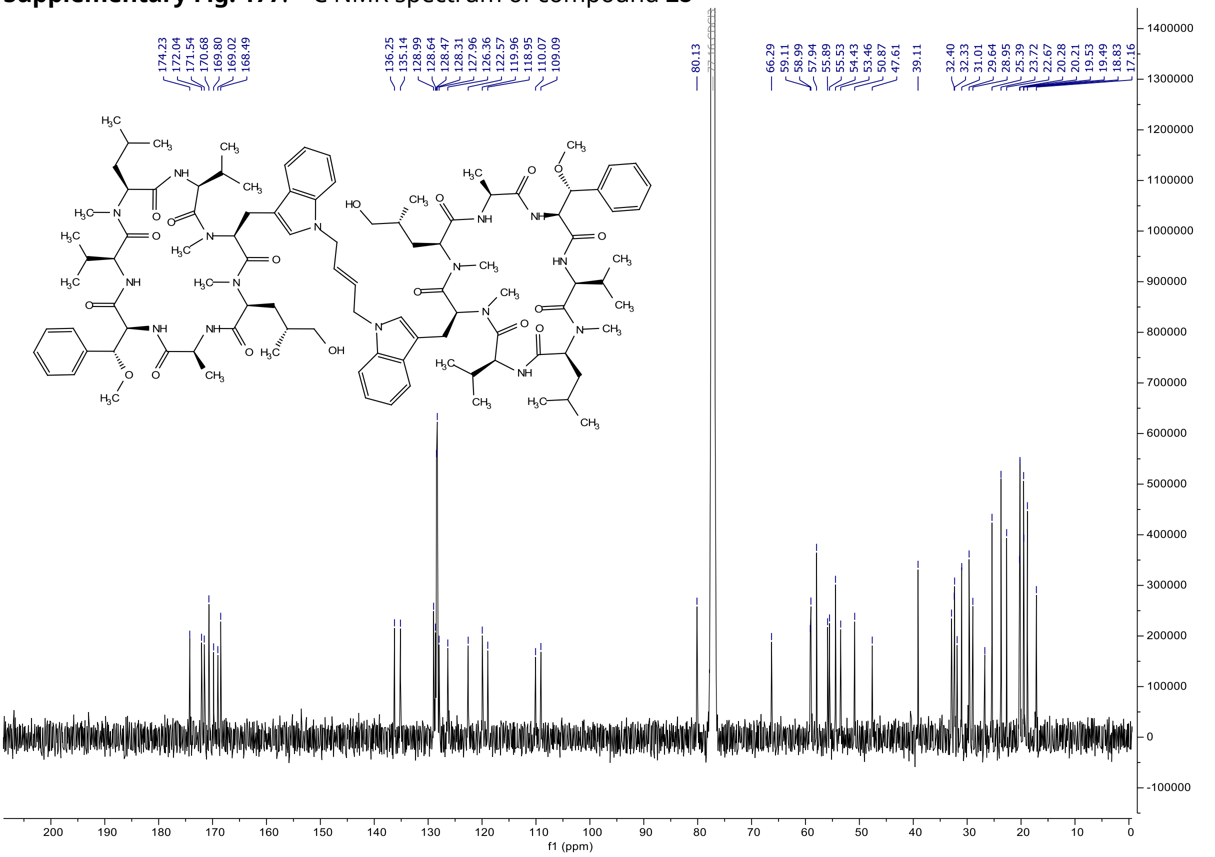

Supplementary Fig. 178. ^{1}H NMR spectrum of compound **SI-61**

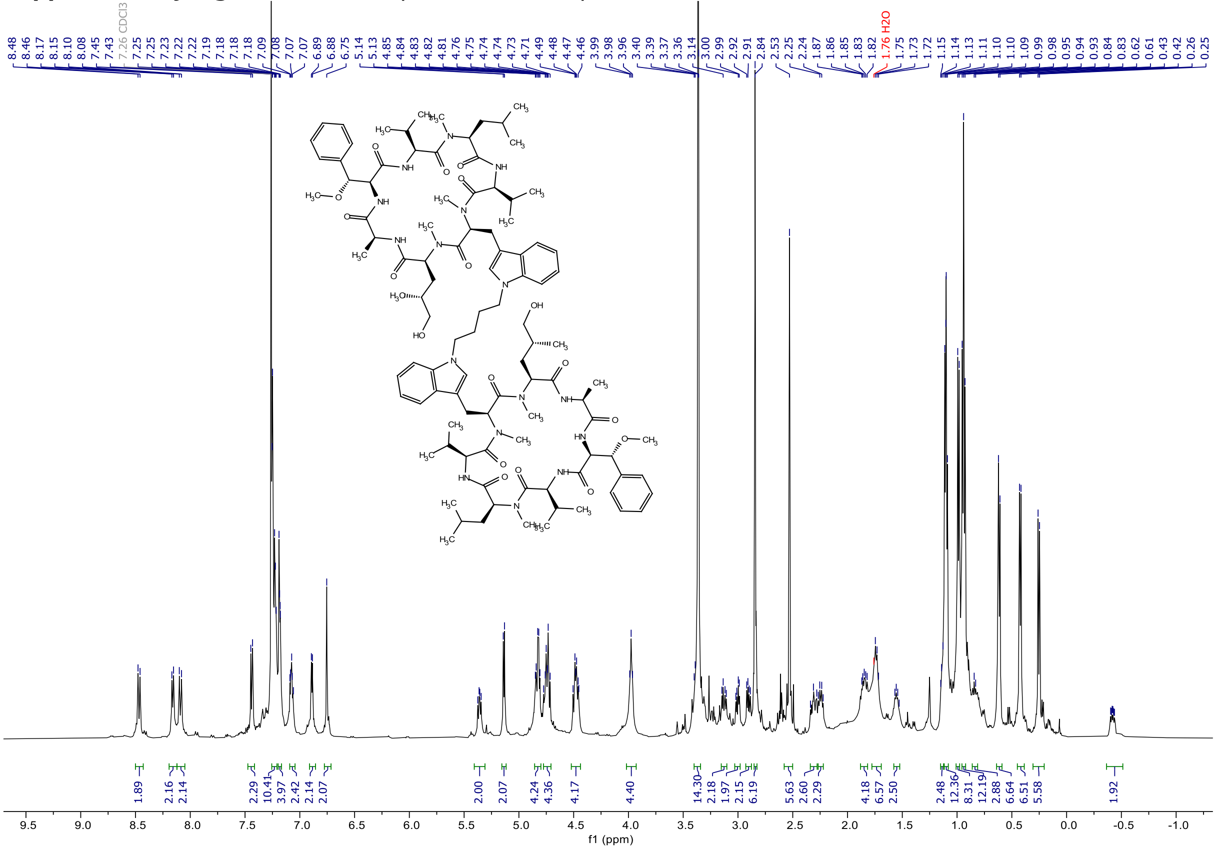

Supplementary Fig. 179. ^{13}C NMR spectrum of compound **SI-61**

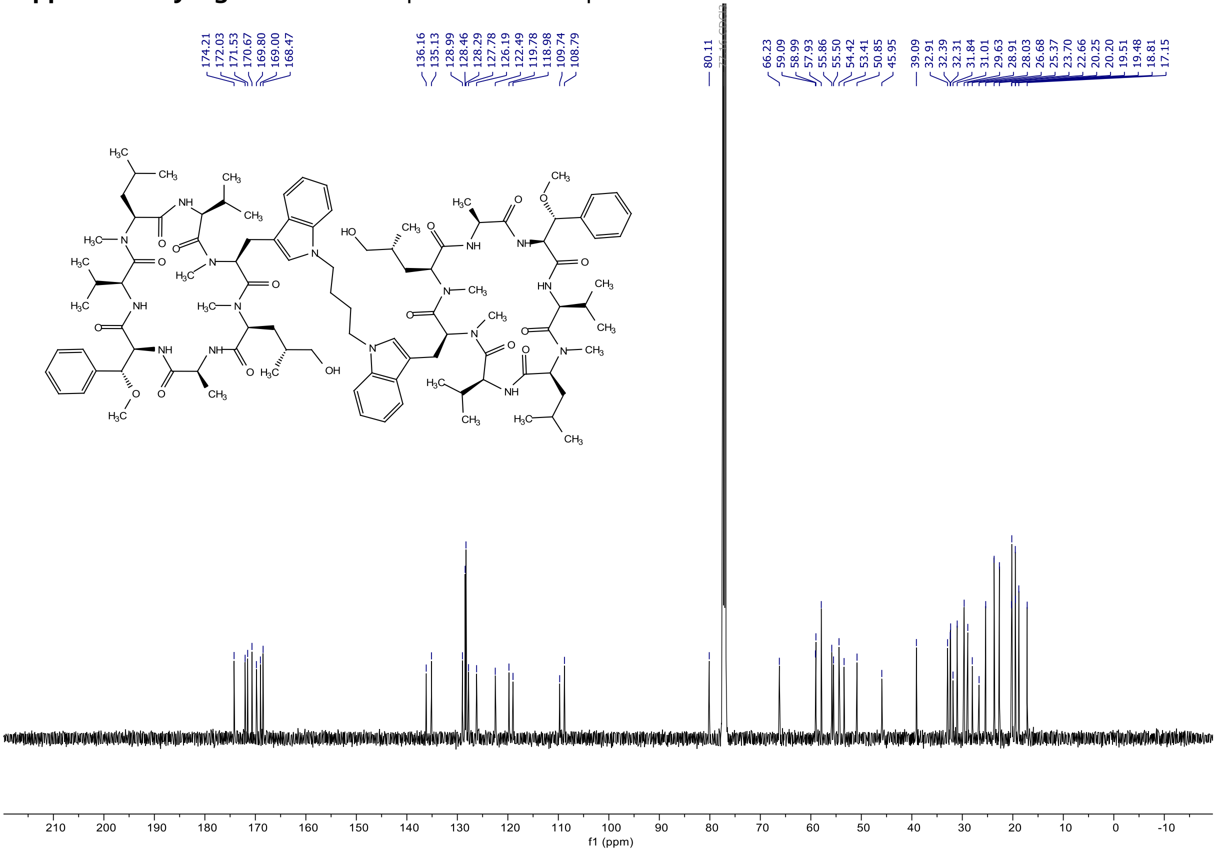

Supplementary Fig. 180. ^{1}H NMR spectrum of compound **SI-62**

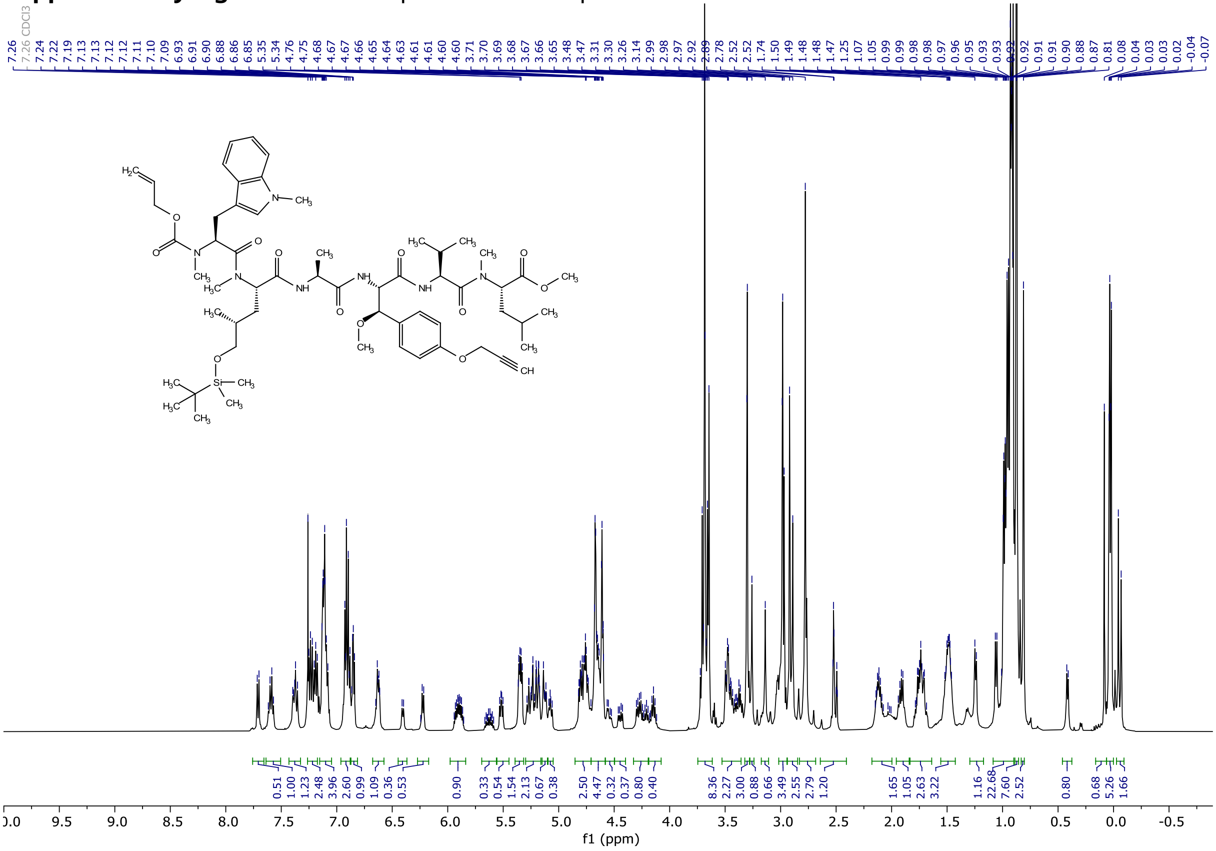

Supplementary Fig. 181. ^{13}C NMR spectrum of compound **SI-62**

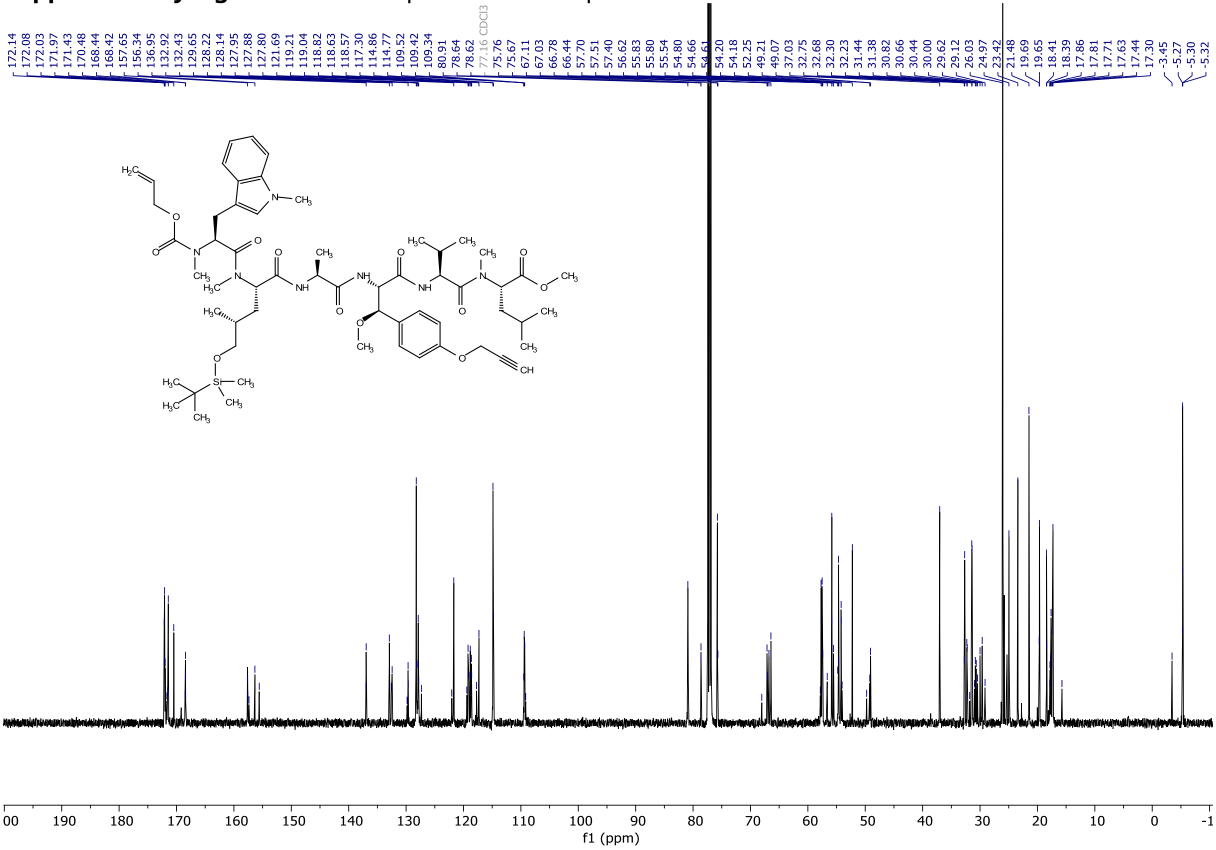

Supplementary Fig. 182. ^{1}H NMR spectrum of compound **SI-63**

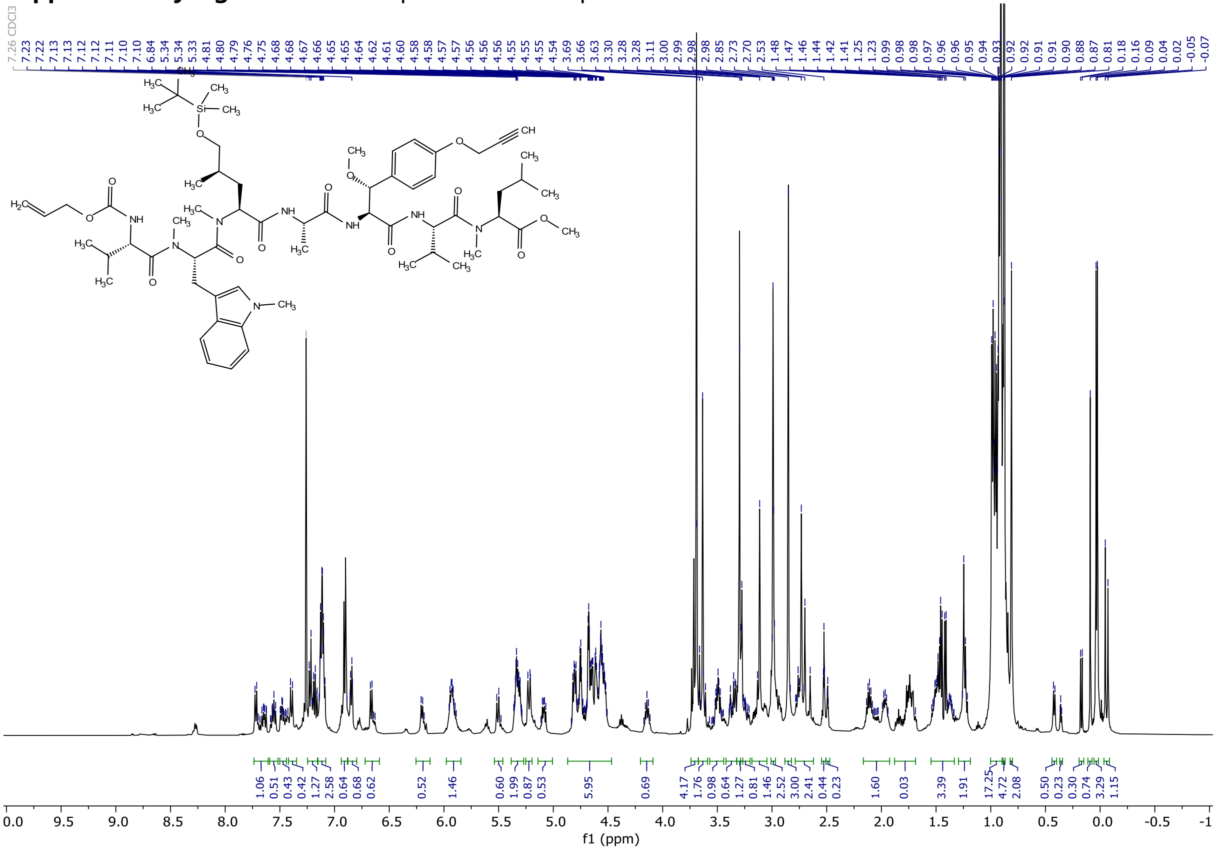

Supplementary Fig. 183. ^{13}C NMR spectrum of compound **SI-63**

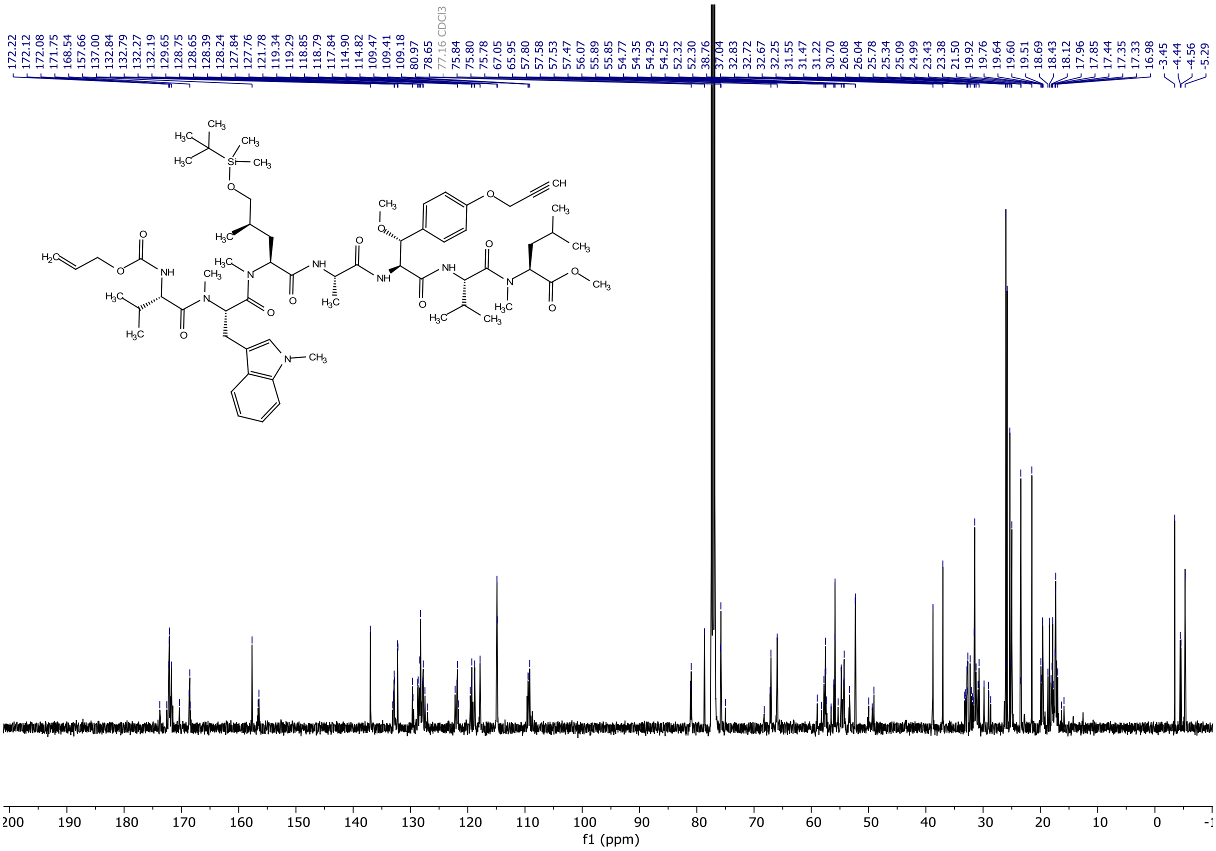

Supplementary Fig. 184. ^{1}H NMR spectrum of compound **SI-64**

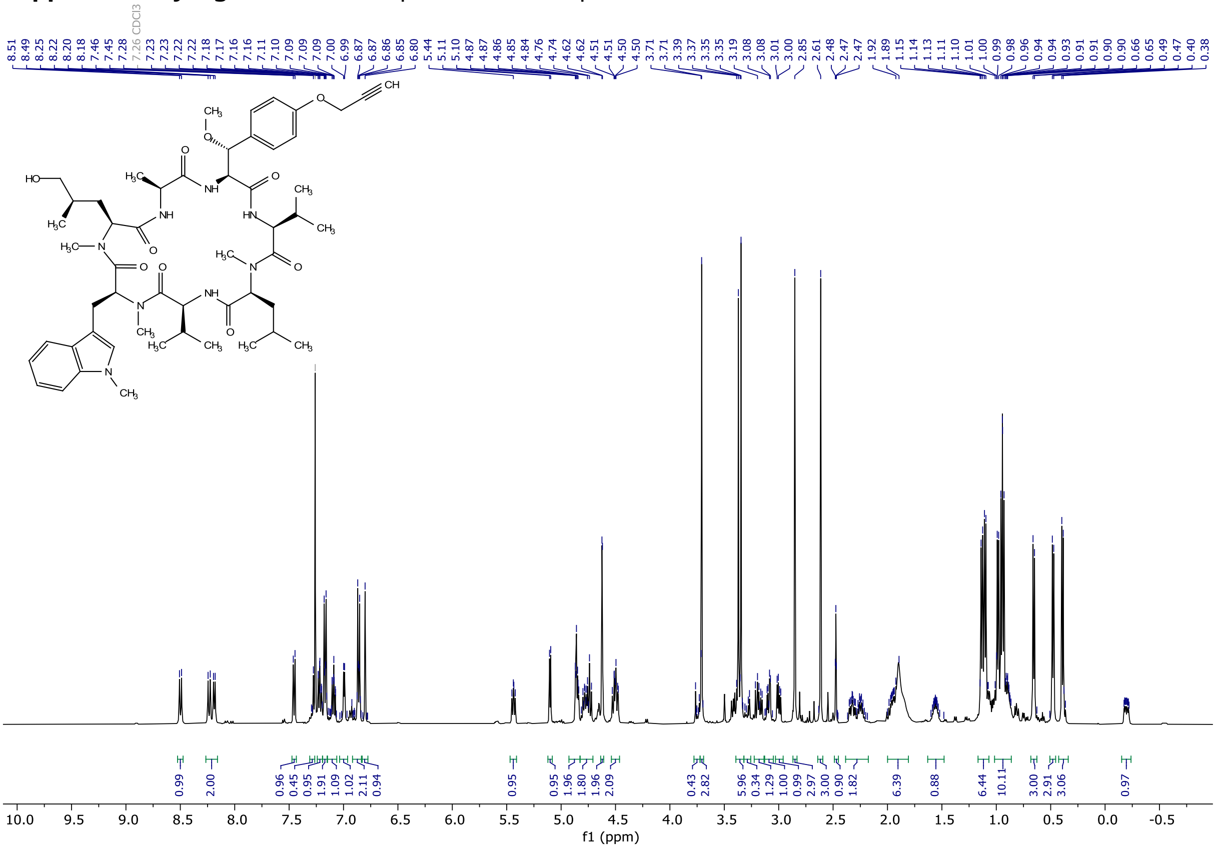

Supplementary Fig. 185. ^{13}C NMR spectrum of compound **SI-64**

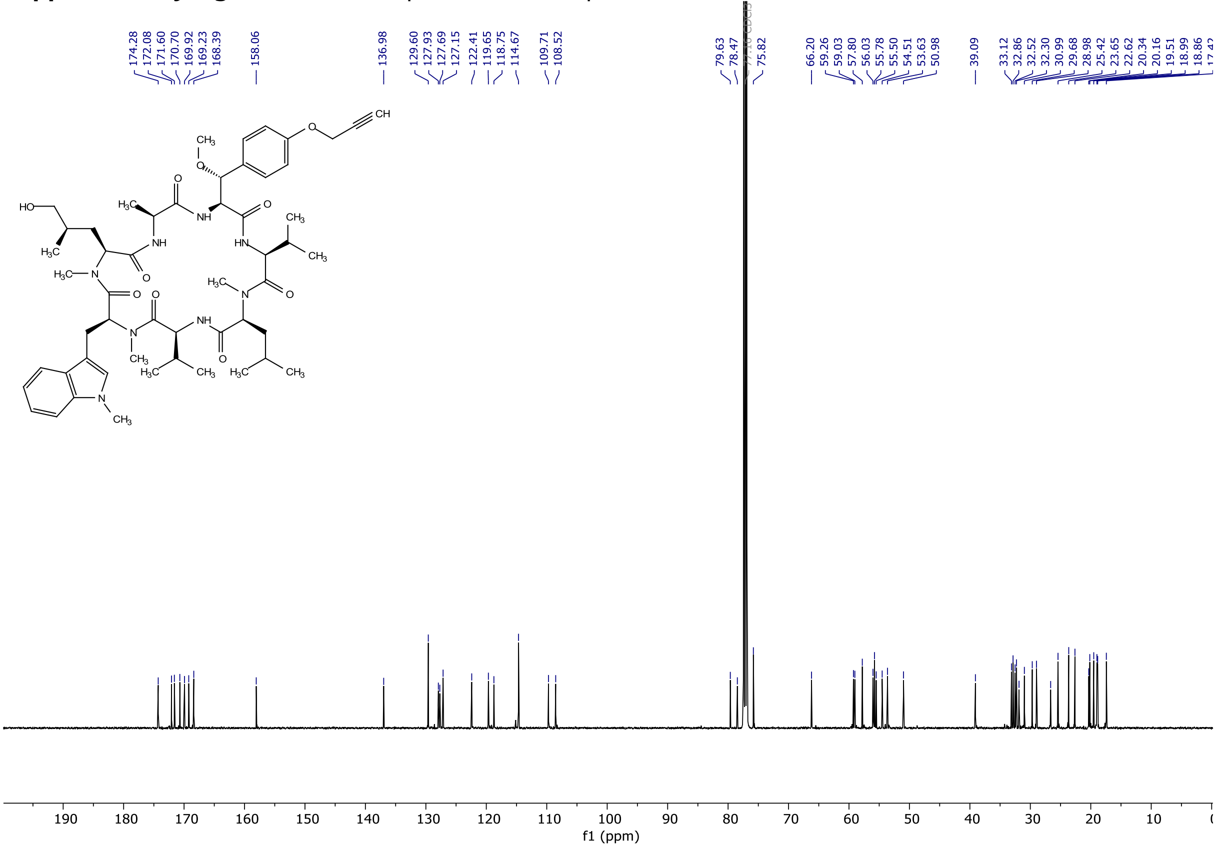

Supplementary Fig. 186. ^{1}H NMR spectrum of compound **SI-65**

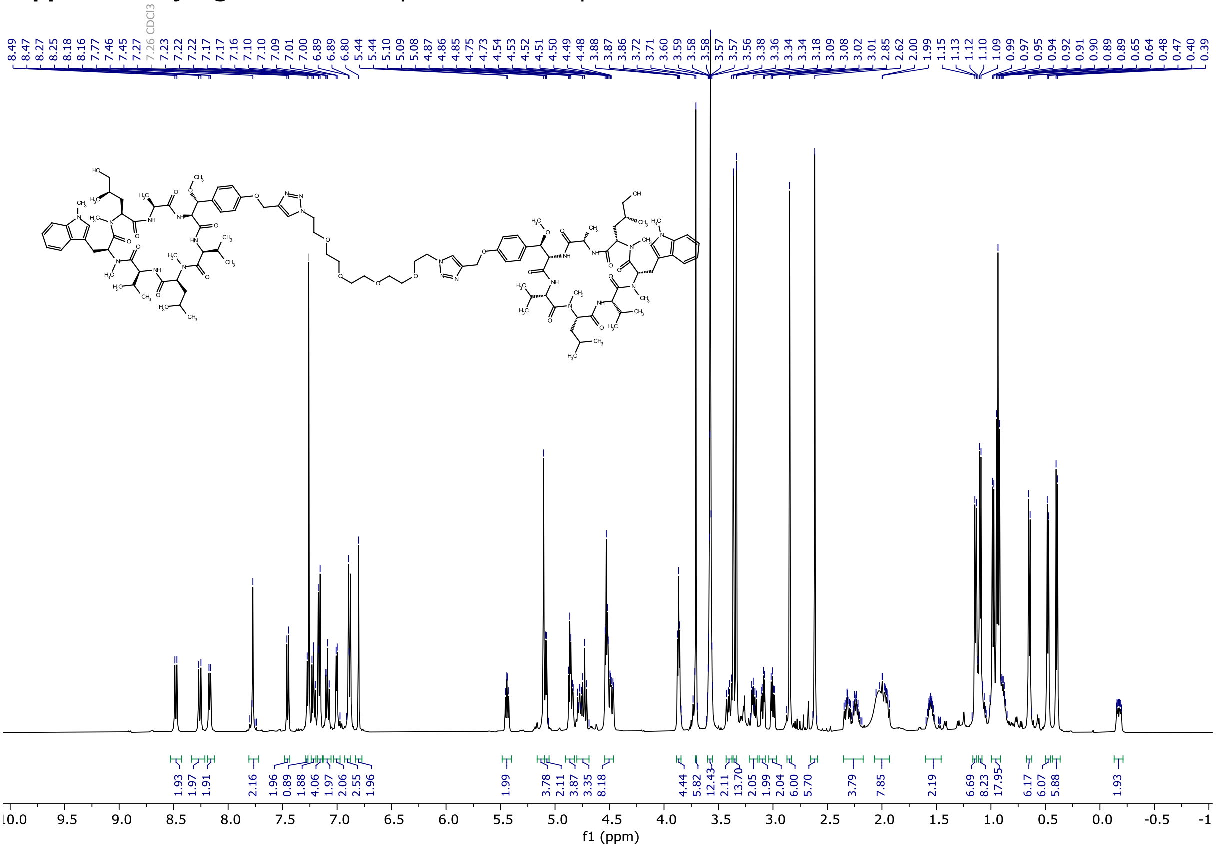

Supplementary Fig. 187. ^{13}C NMR spectrum of compound **SI-65**

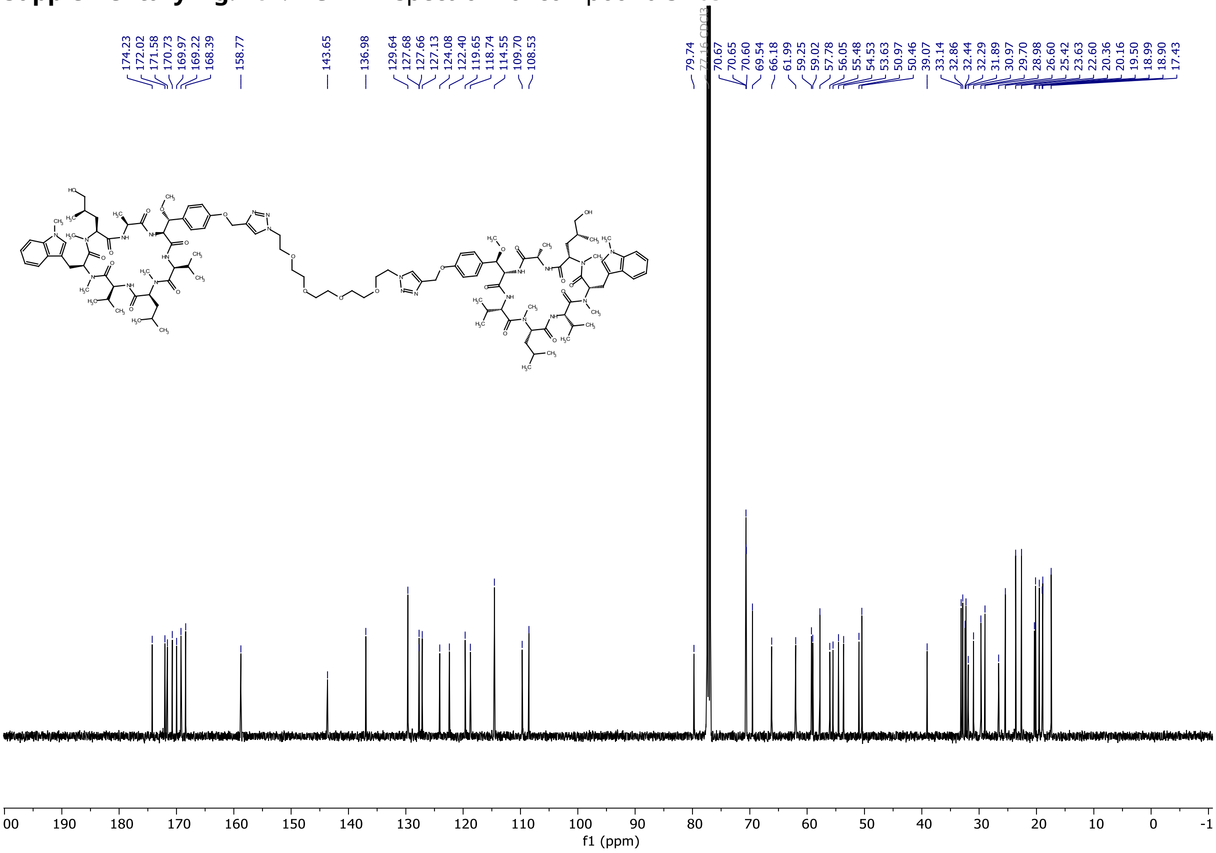

Supplementary Fig. 188. ^{1}H NMR spectrum of compound **SI-66**

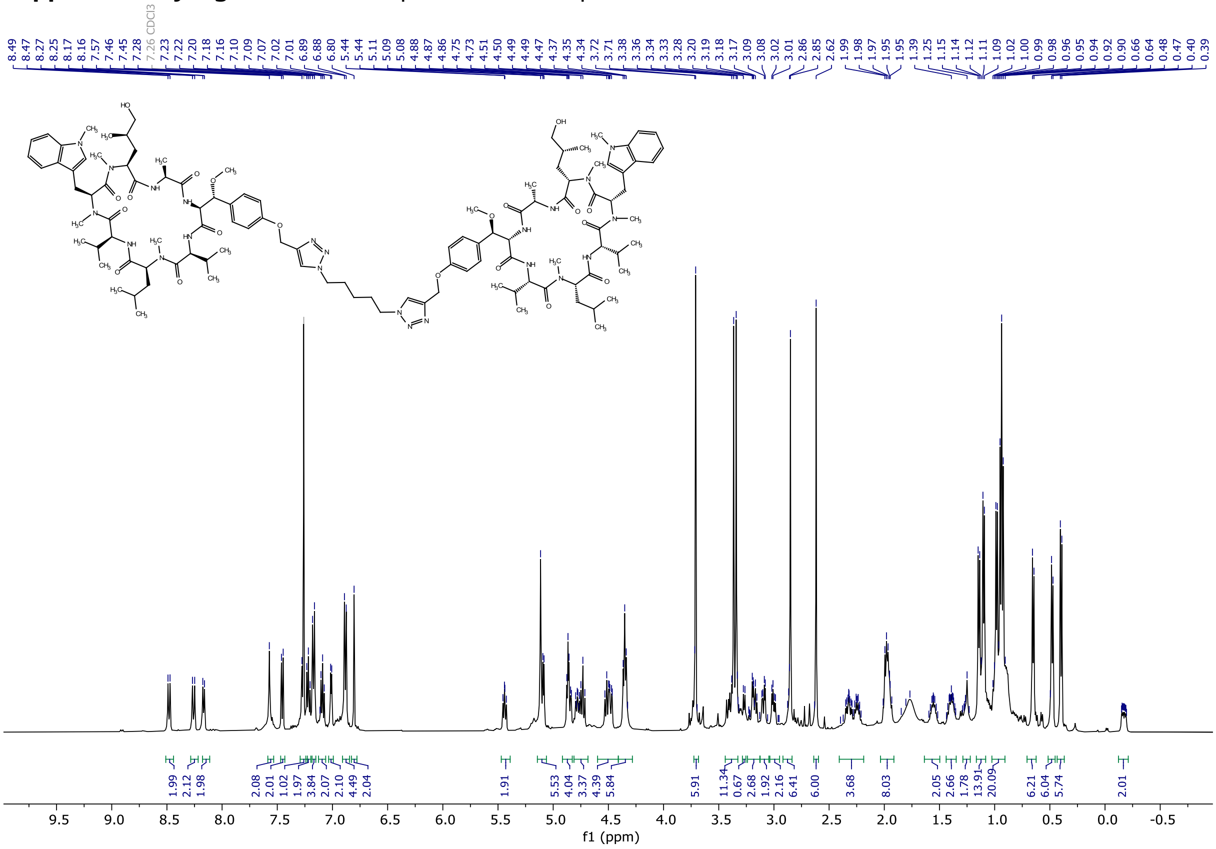

Supplementary Fig. 189. ^{13}C NMR spectrum of compound **SI-66**

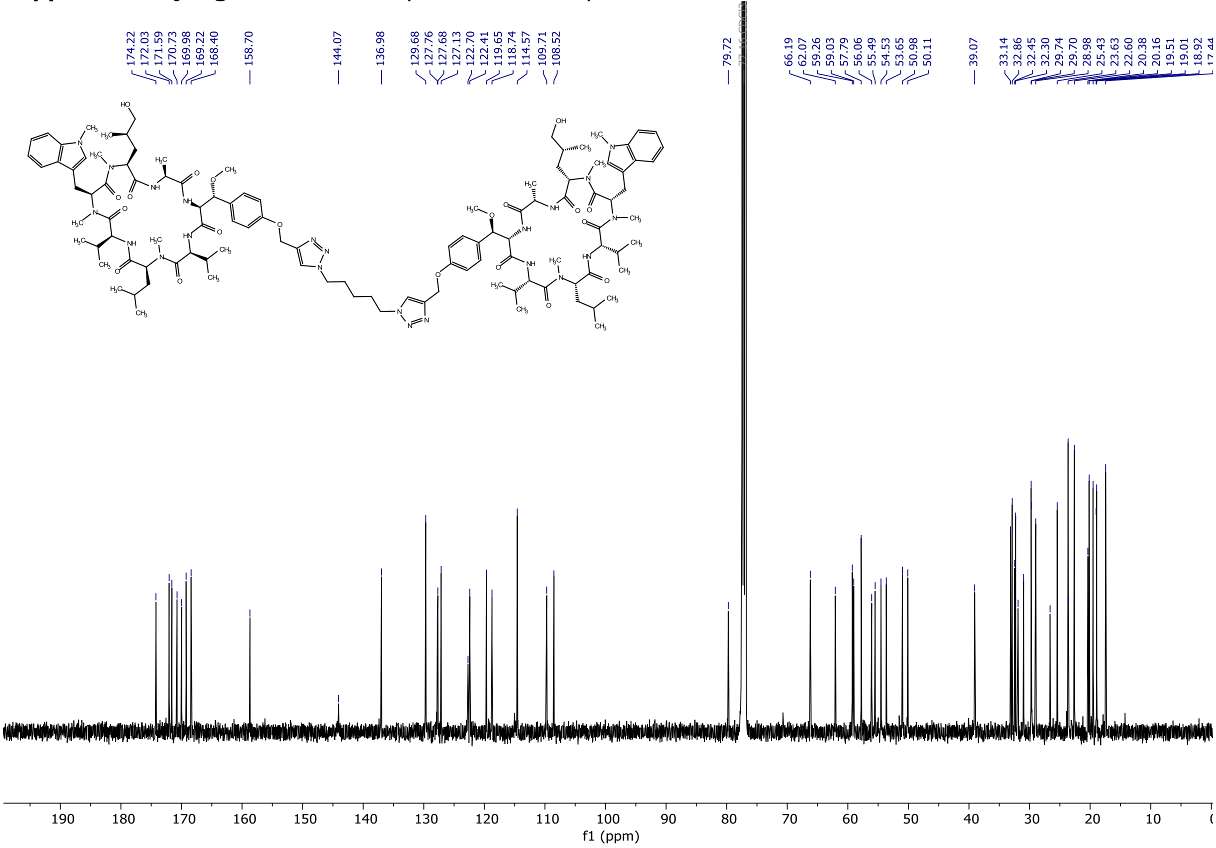

Supplementary Fig. 190. ^{1}H NMR spectrum of compound **SI-67**

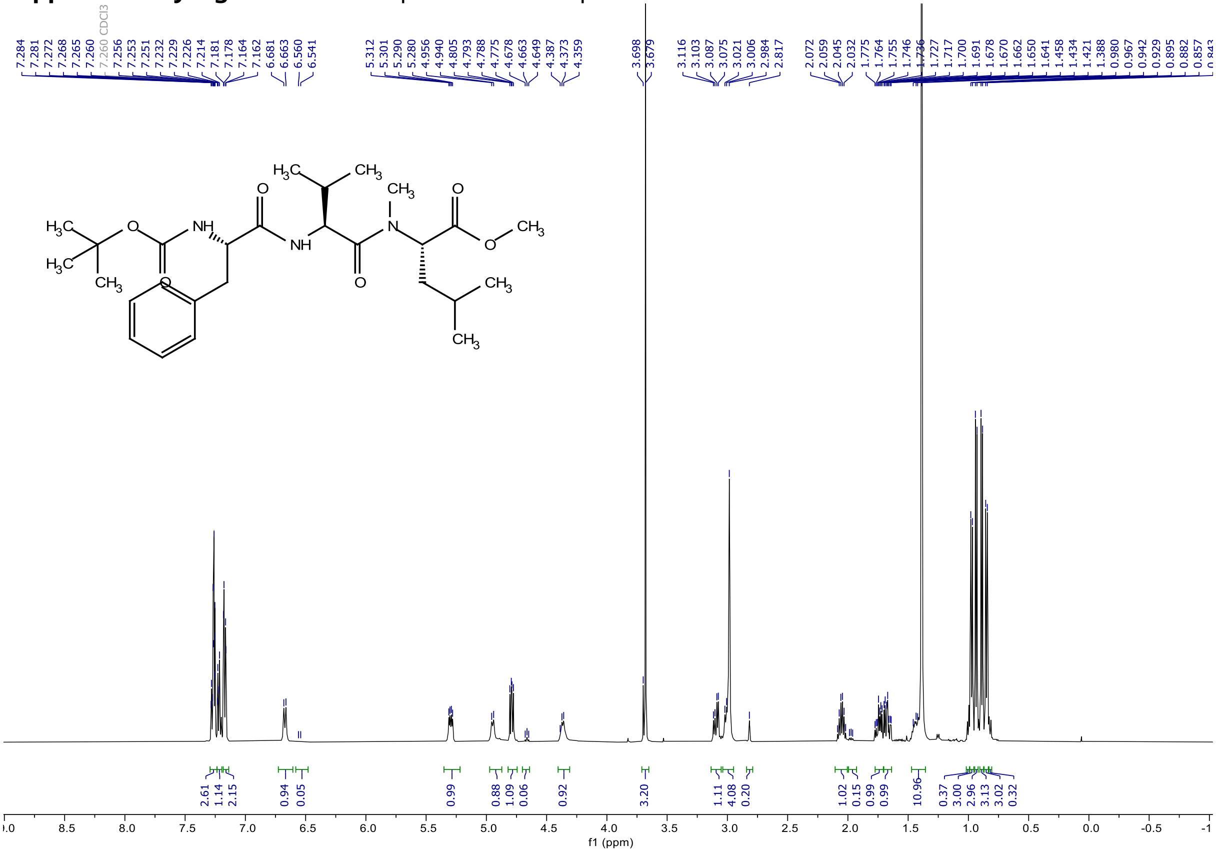

Supplementary Fig. 191. ^{13}C NMR spectrum of compound **SI-67**

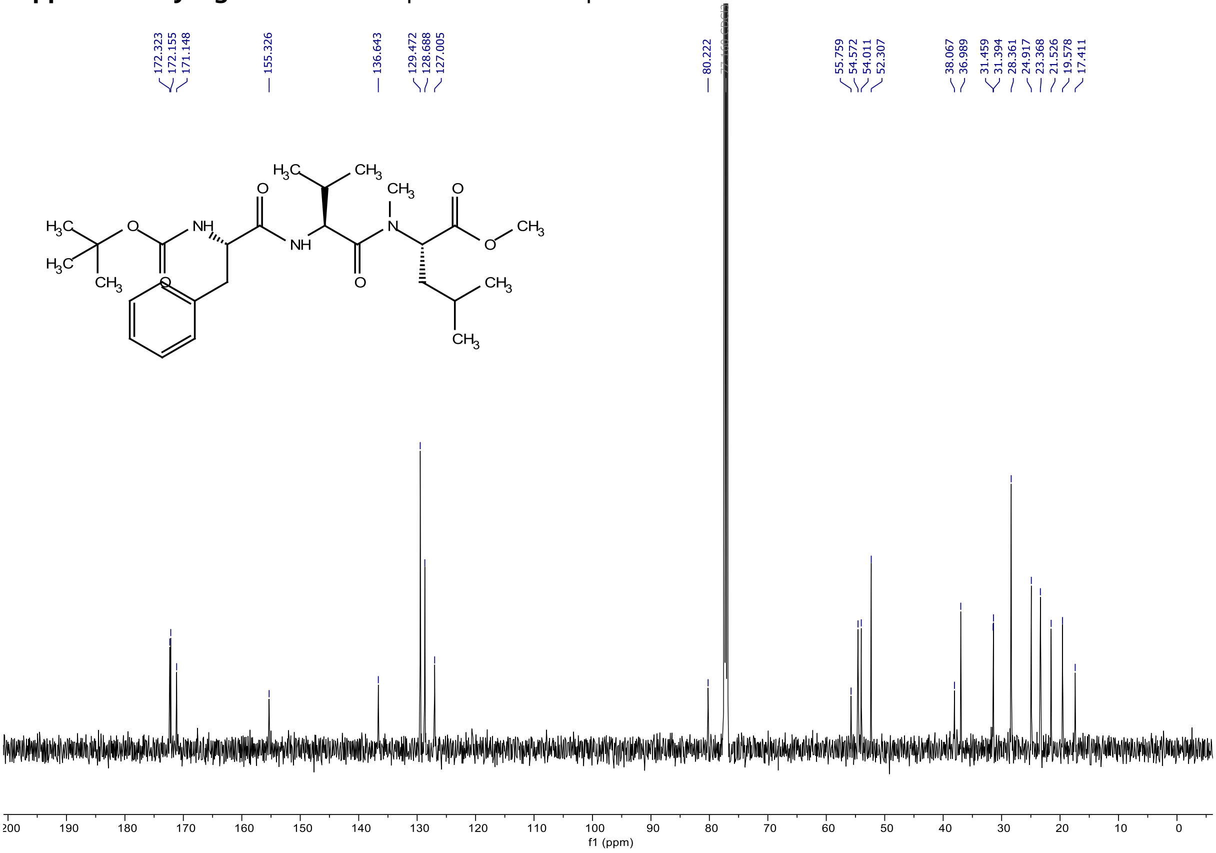

Supplementary Fig. 192. ^{1}H NMR spectrum of compound **SI-68**

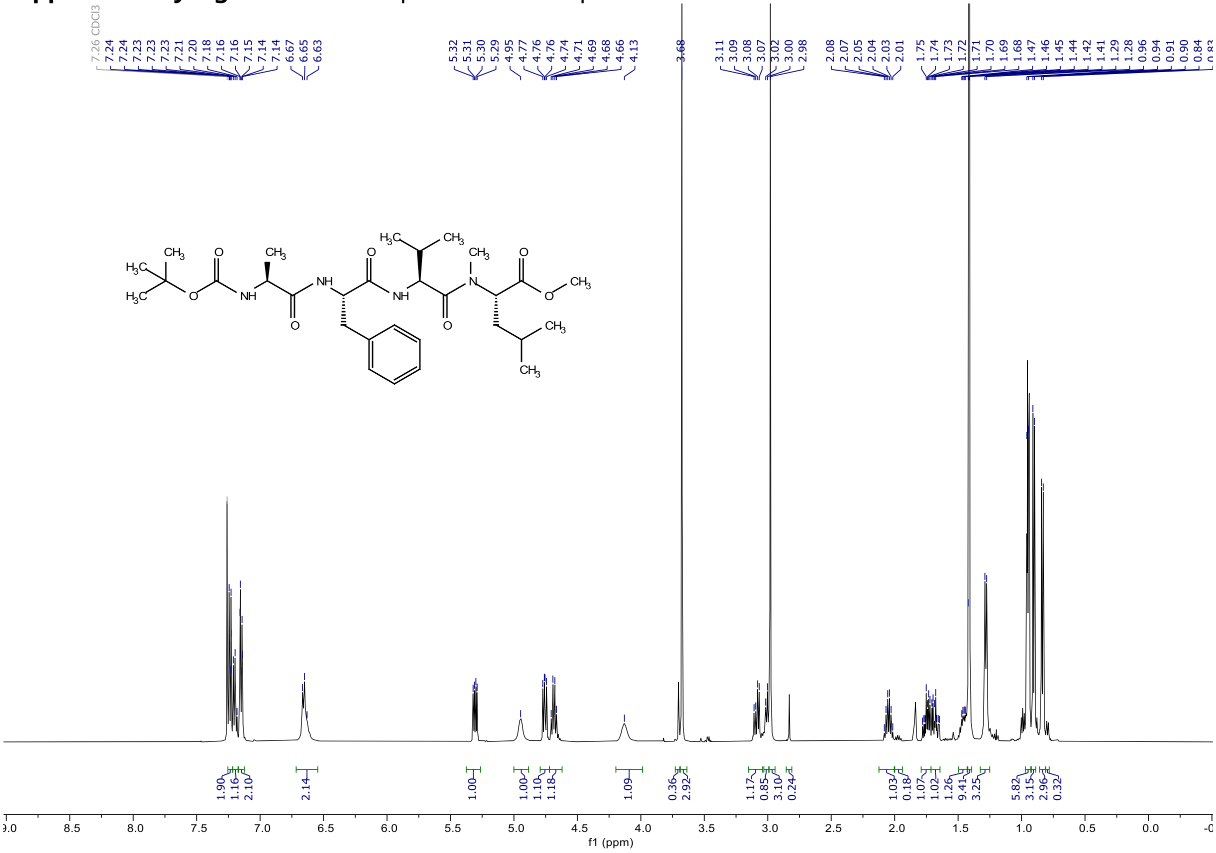

Supplementary Fig. 193. ^{13}C NMR spectrum of compound **SI-68**

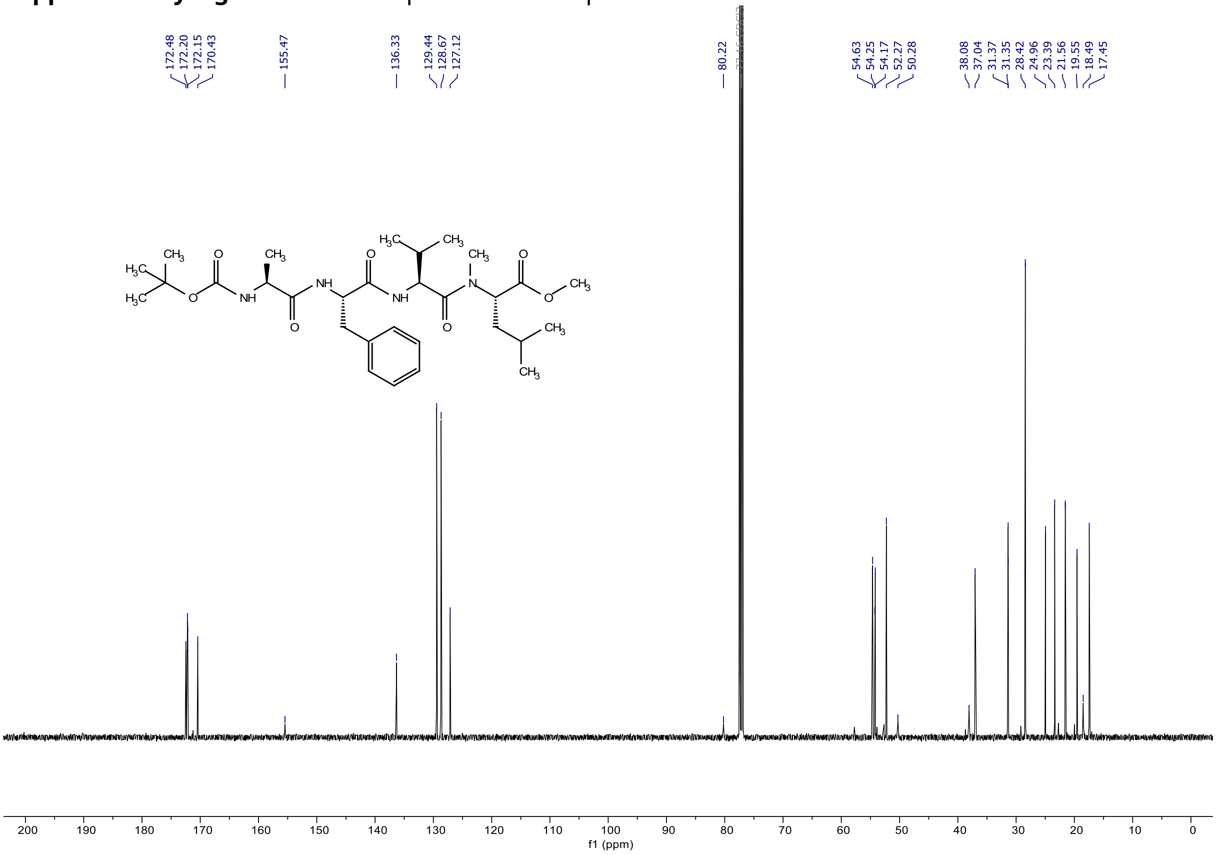

Supplementary Fig. 194. ^{1}H NMR spectrum of compound **SI-69**

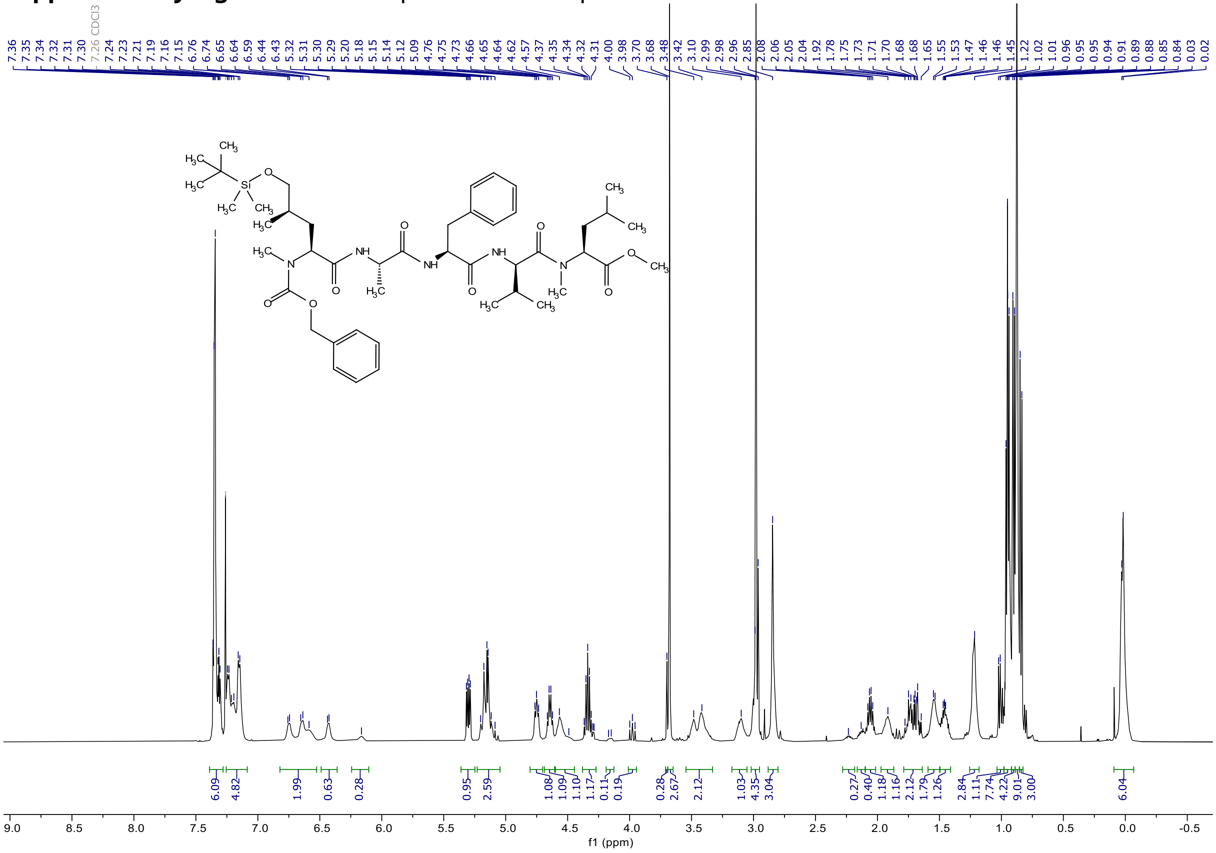

Supplementary Fig. 195. ^{13}C NMR spectrum of compound **SI-69**

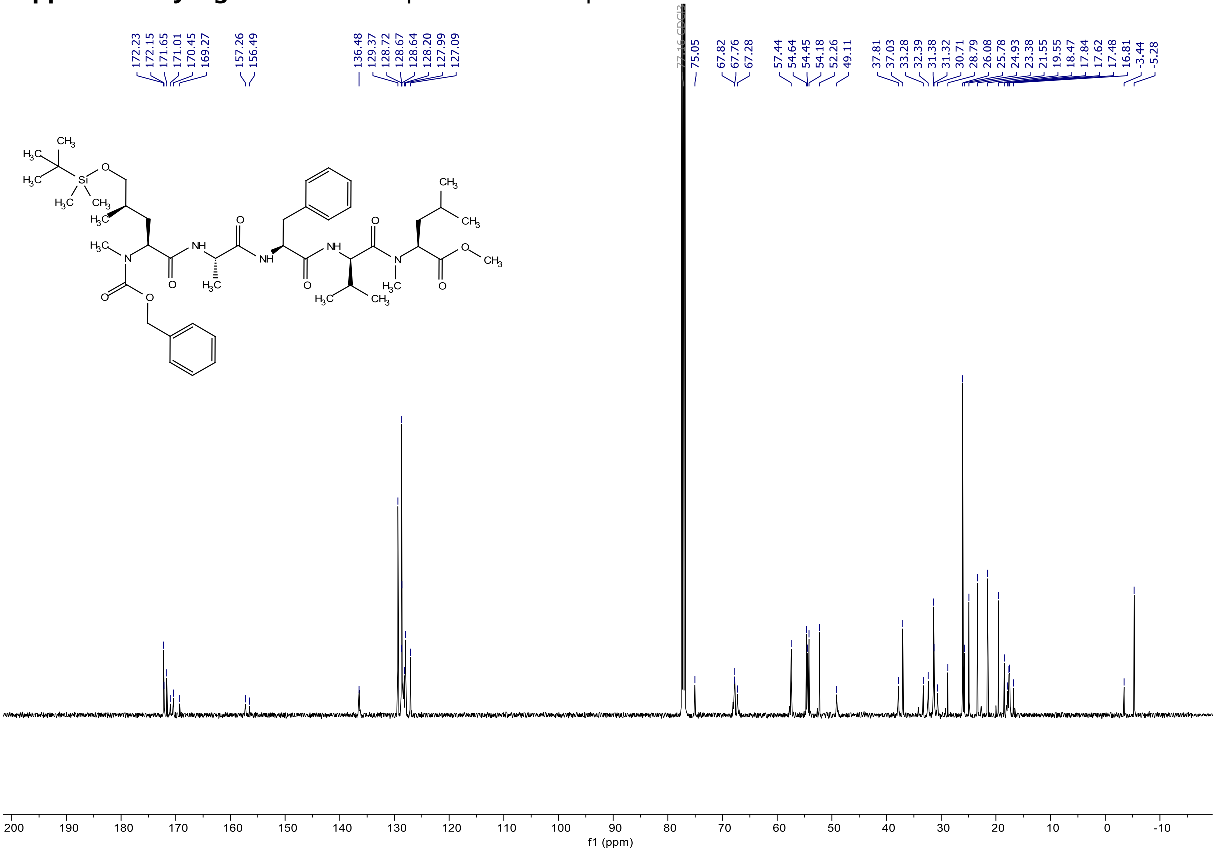

Supplementary Fig. 196. ^{1}H NMR spectrum of compound **SI-70**

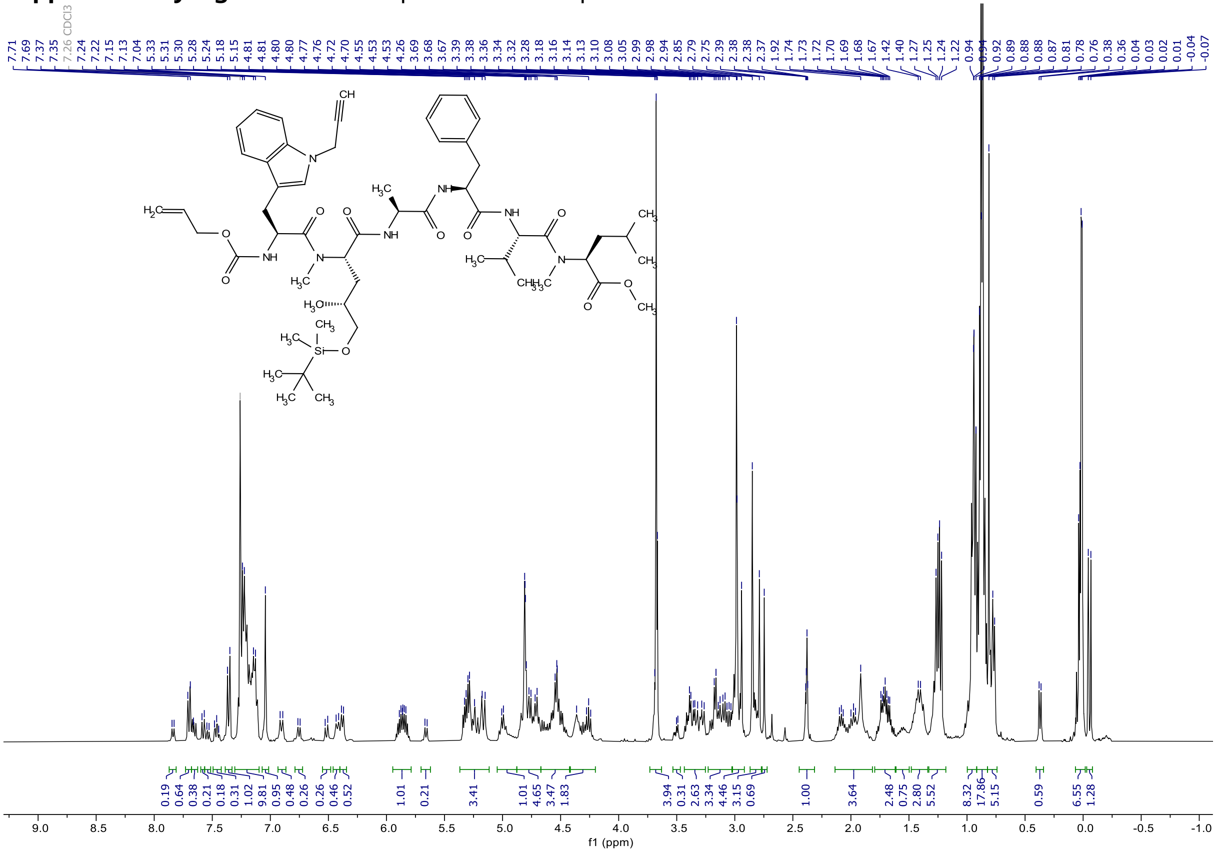

Supplementary Fig. 197. ^{13}C NMR spectrum of compound **SI-70**

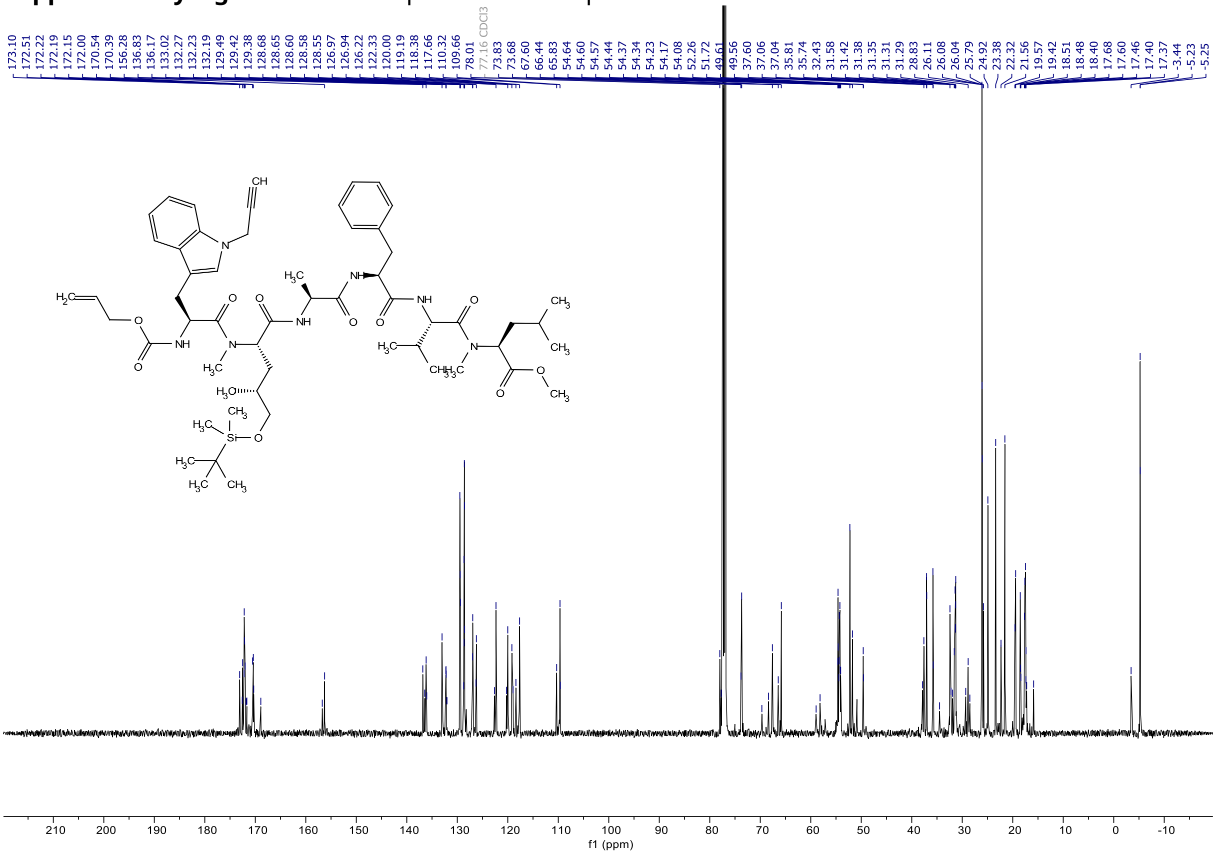

Supplementary Fig. 198. ^{1}H NMR spectrum of compound **SI-71**

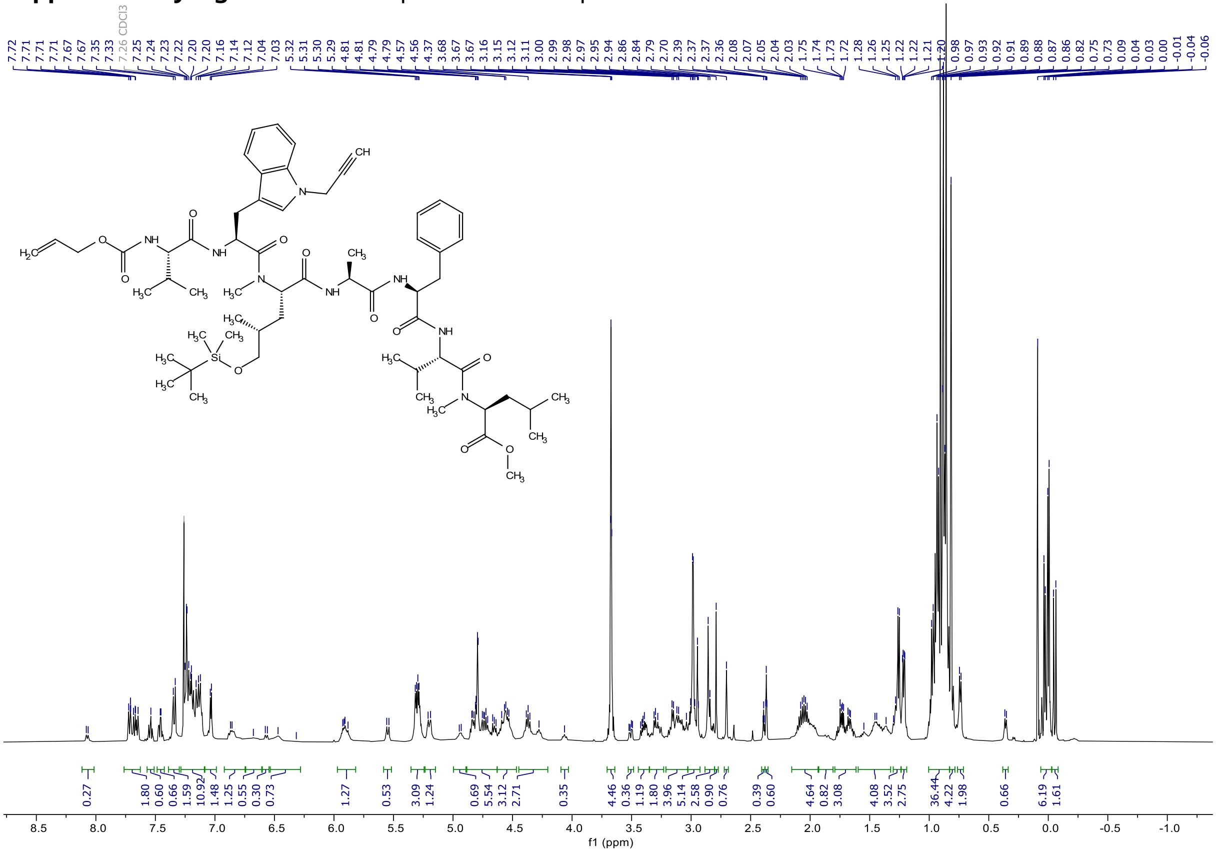

Supplementary Fig. 199. ^{13}C NMR spectrum of compound **SI-71**

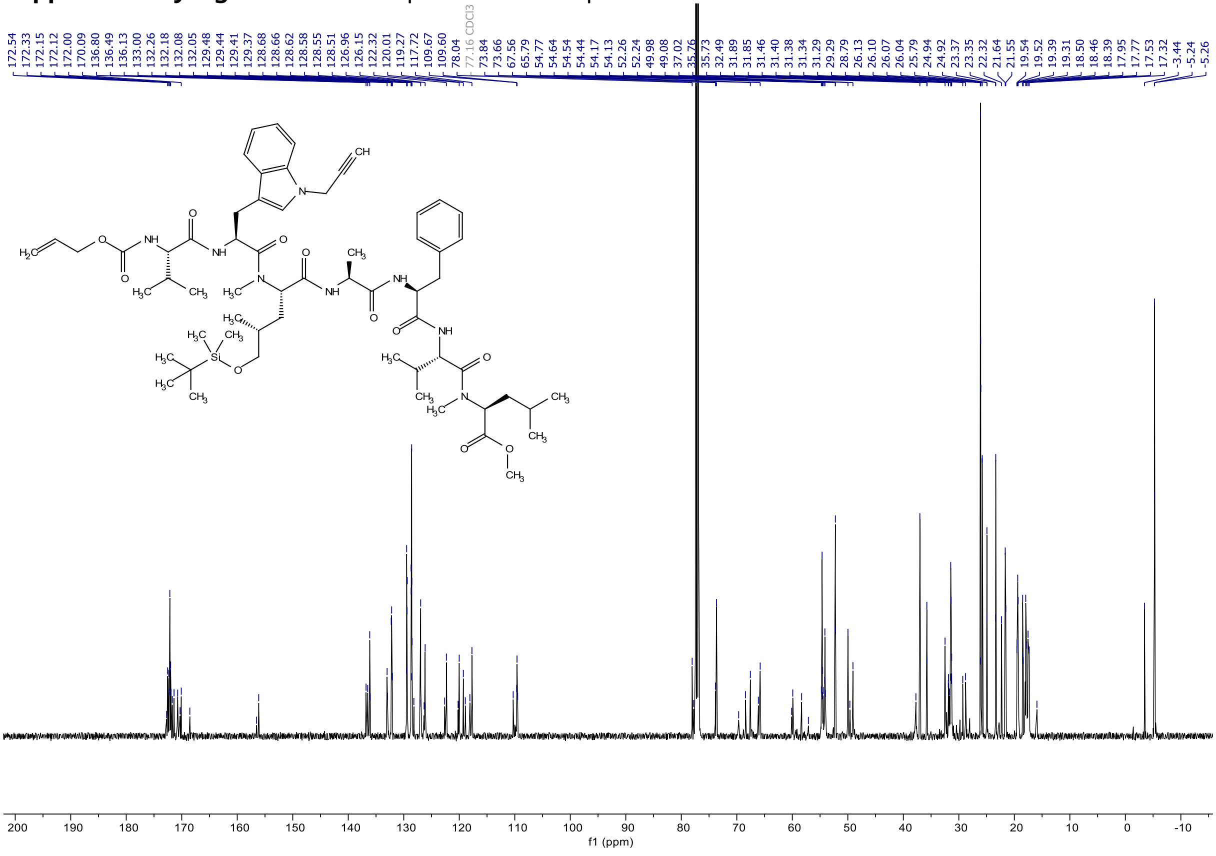

Supplementary Fig. 200. ^{1}H NMR spectrum of compound **SI-72**

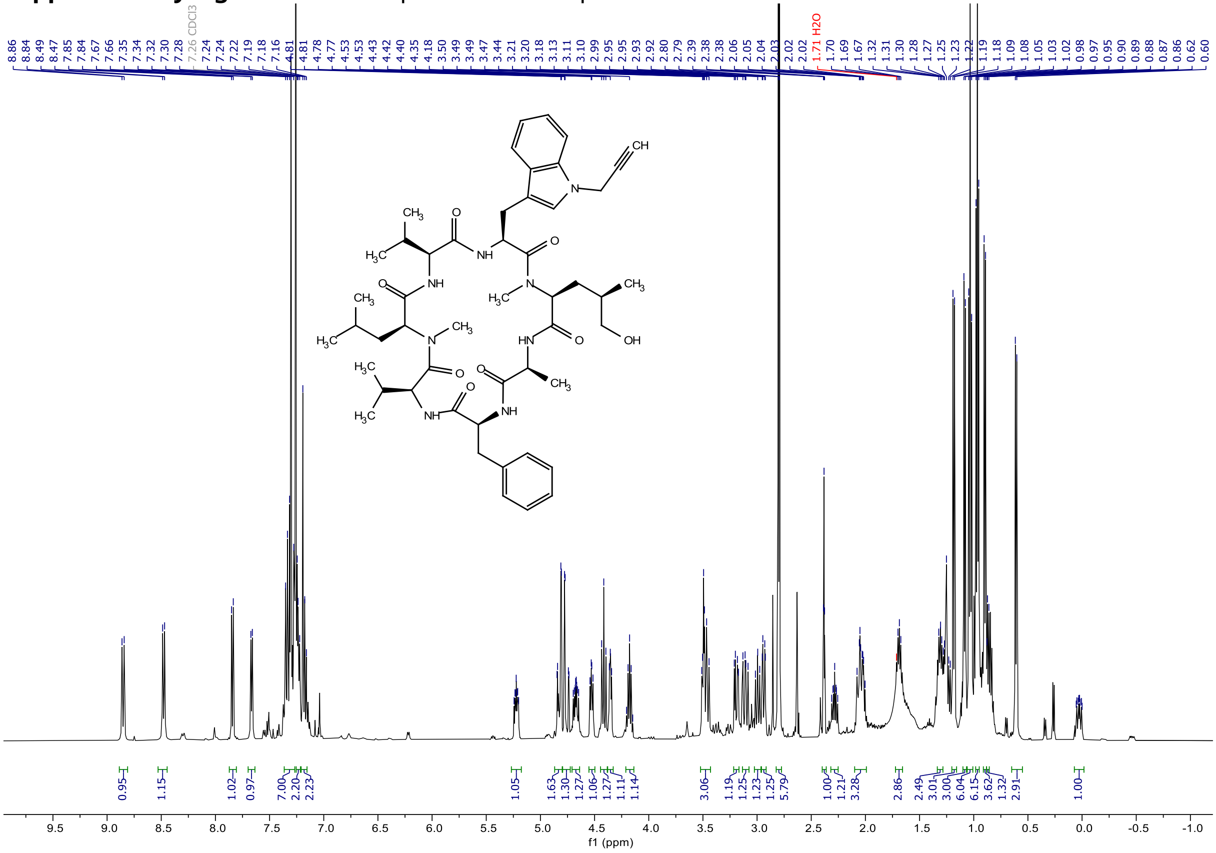

Supplementary Fig. 201. ^{13}C NMR spectrum of compound **SI-72**

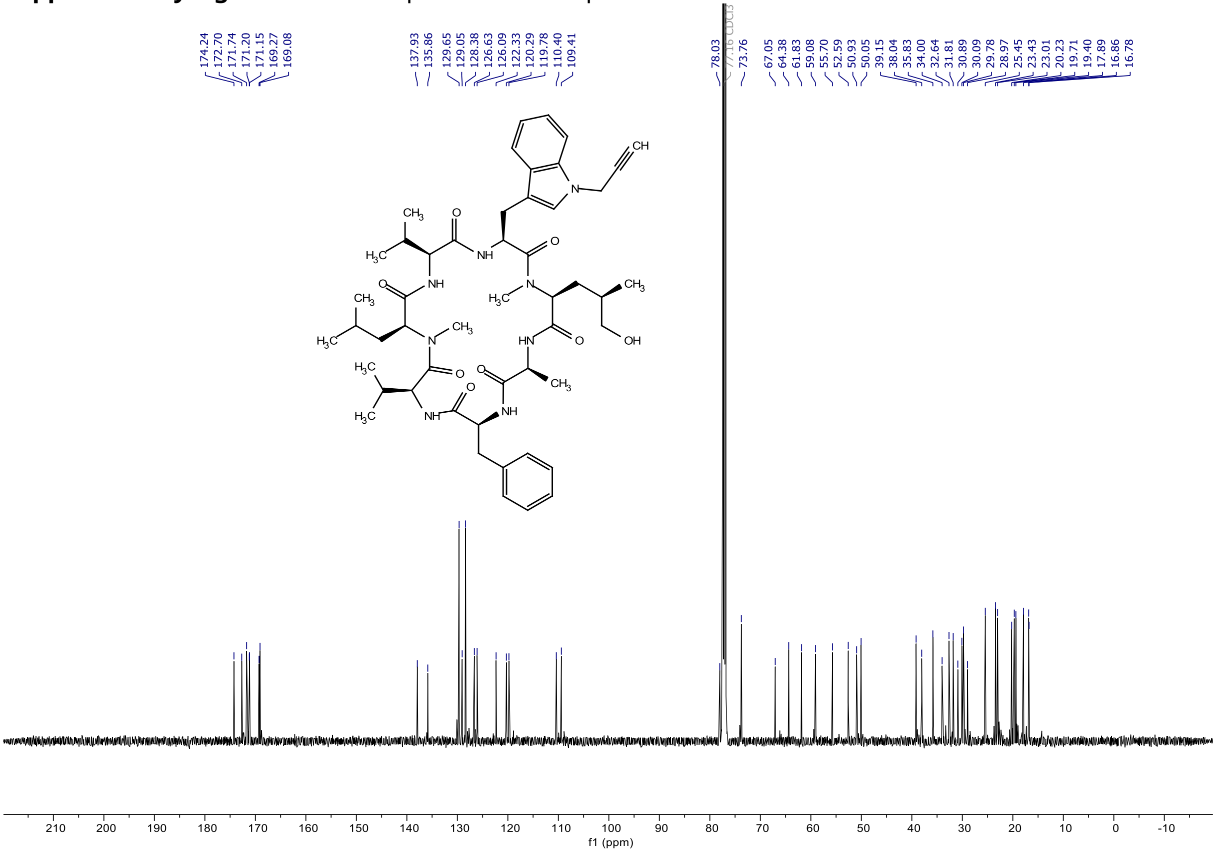

Supplementary Fig. 202. ^{1}H NMR spectrum of compound **SI-73**

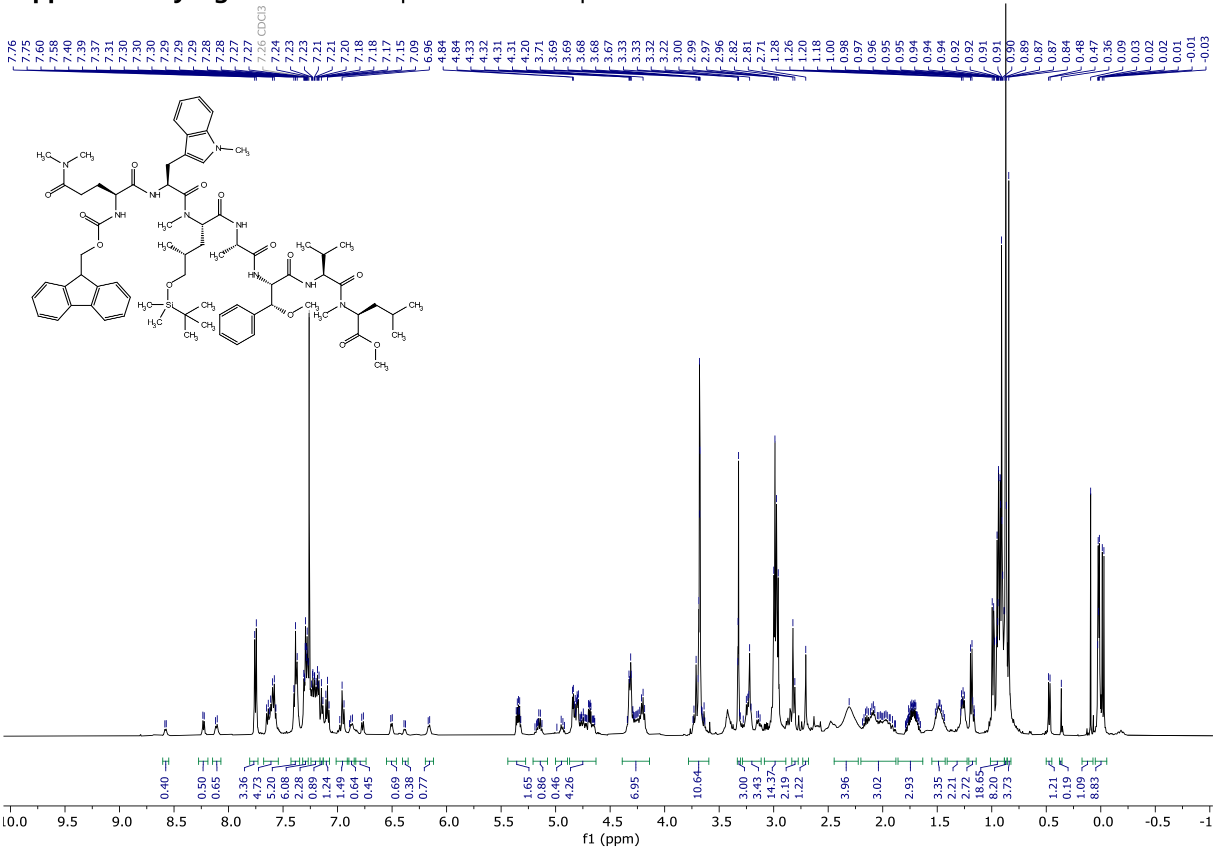

Supplementary Fig. 203. ^{13}C NMR spectrum of compound **SI-73**

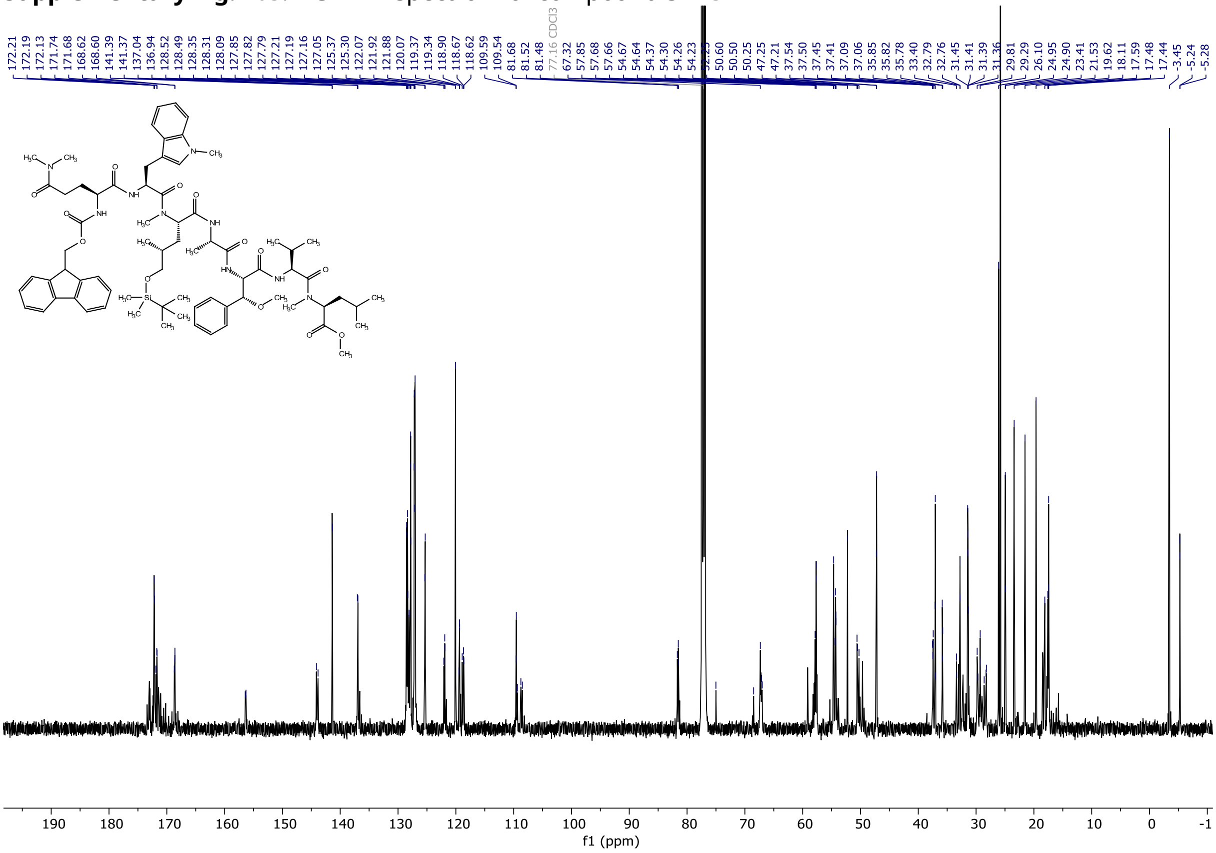

Supplementary Fig. 204. ^{1}H NMR spectrum of compound **SI-74**

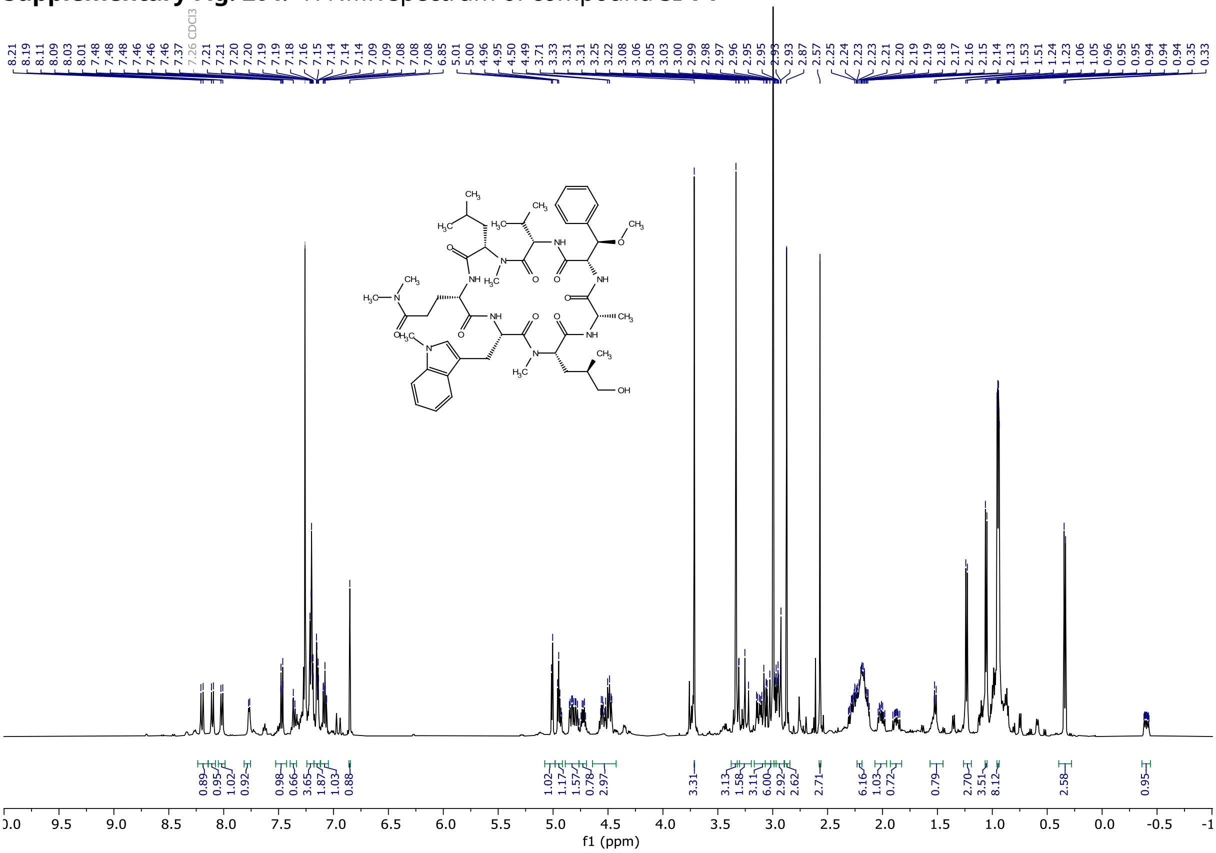

Supplementary Fig. 205. ^{13}C NMR spectrum of compound **SI-74**

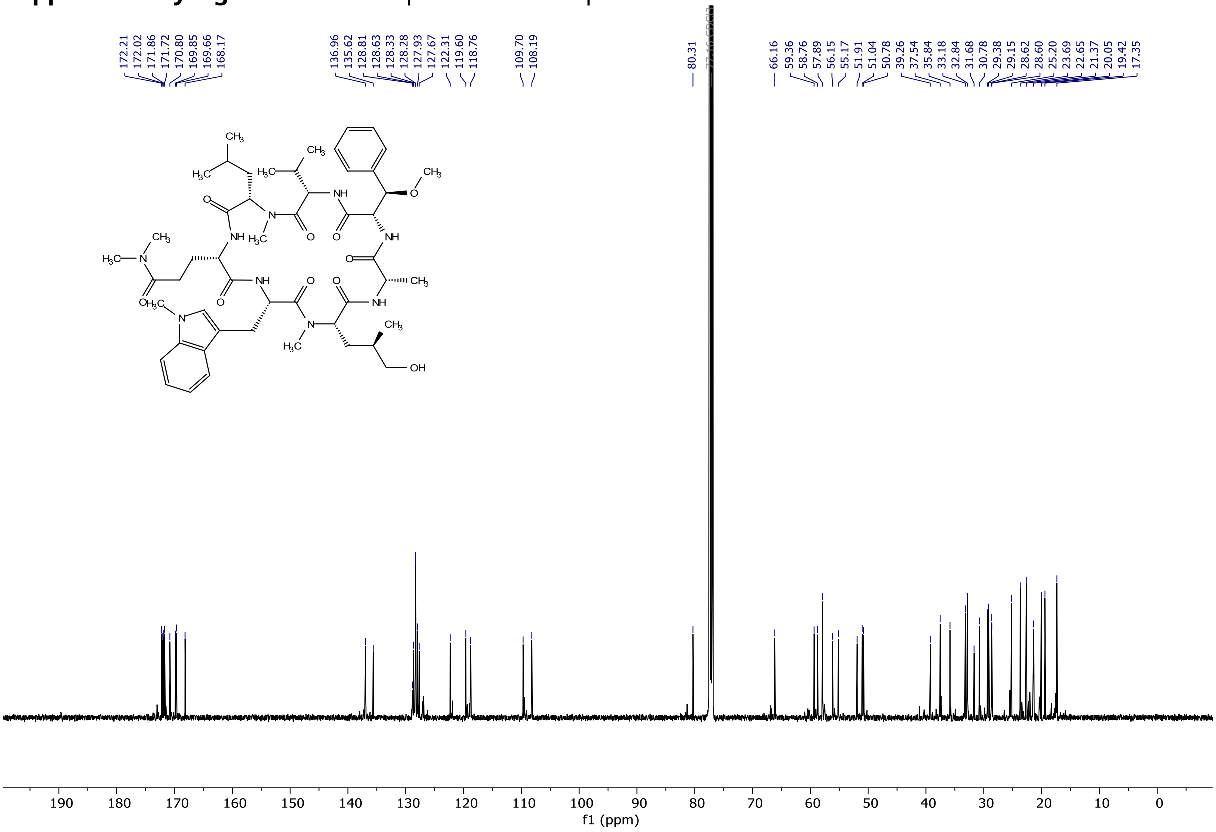

Supplementary Fig. 206. ^{1}H NMR spectrum of compound **SI-75**

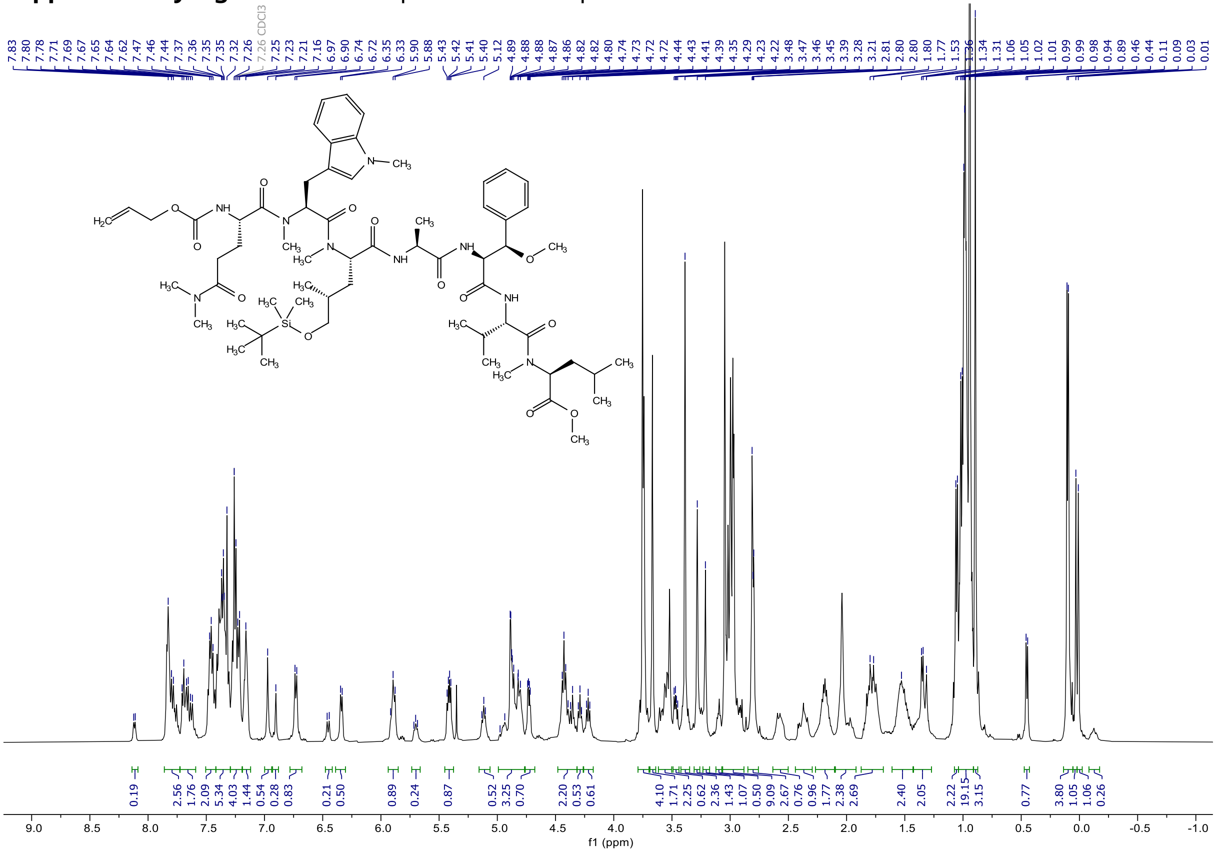

Supplementary Fig. 207. ^{13}C NMR spectrum of compound **SI-75**

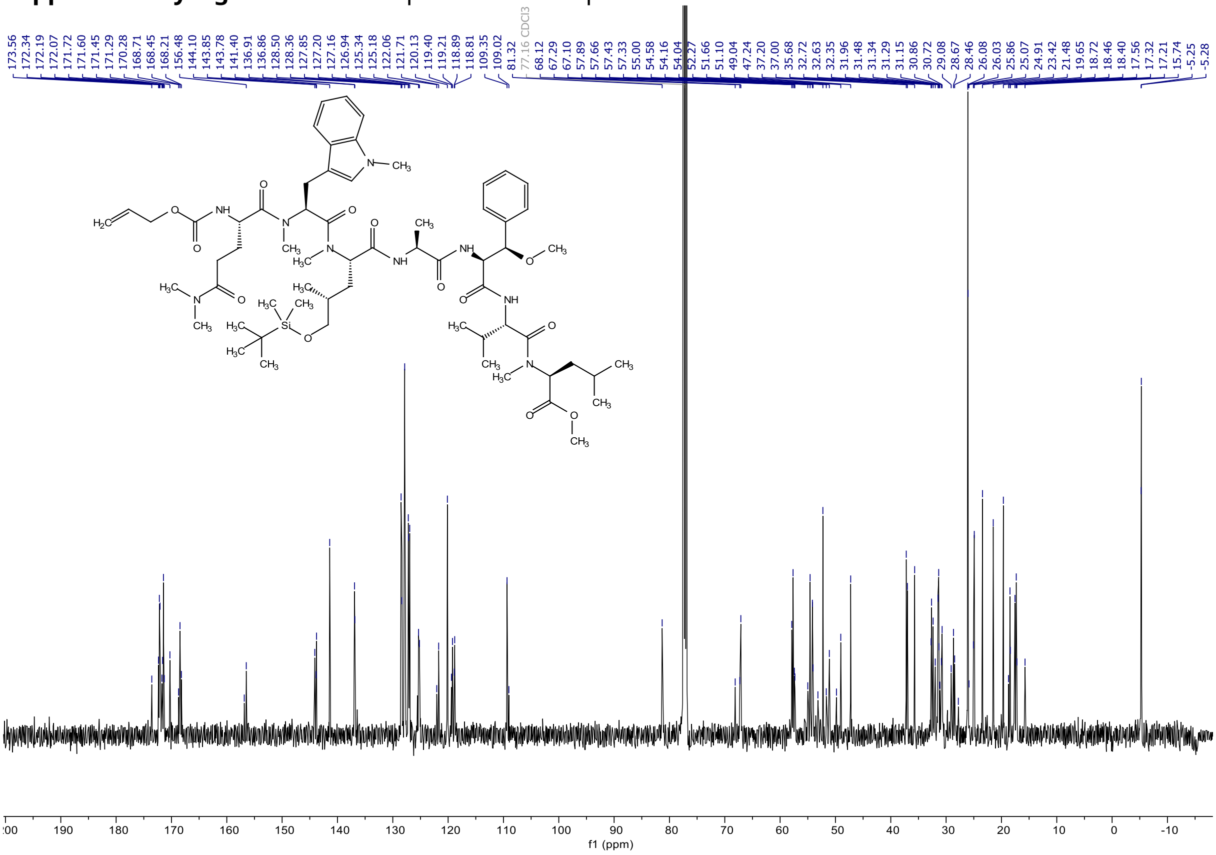

Supplementary Fig. 208. ^{1}H NMR spectrum of compound **SI-76**

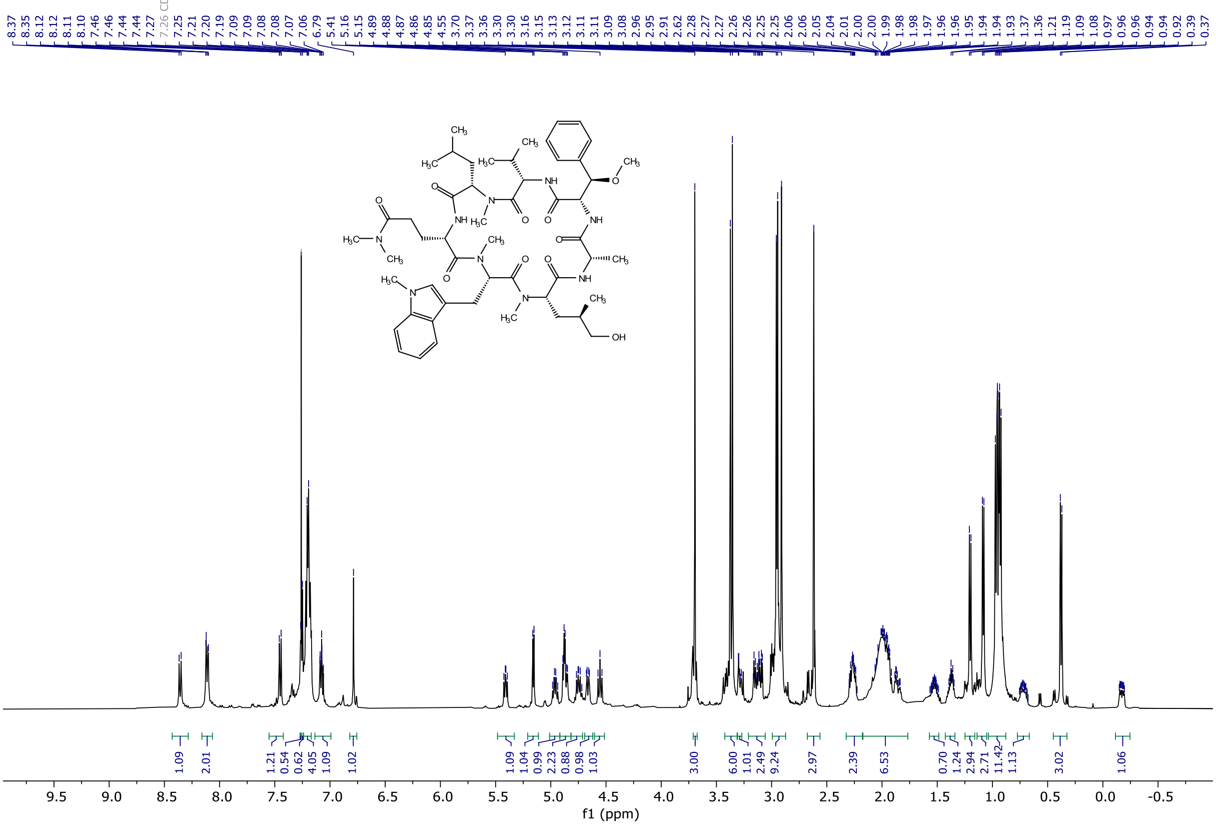

Supplementary Fig. 209. ^{13}C NMR spectrum of compound **SI-76**

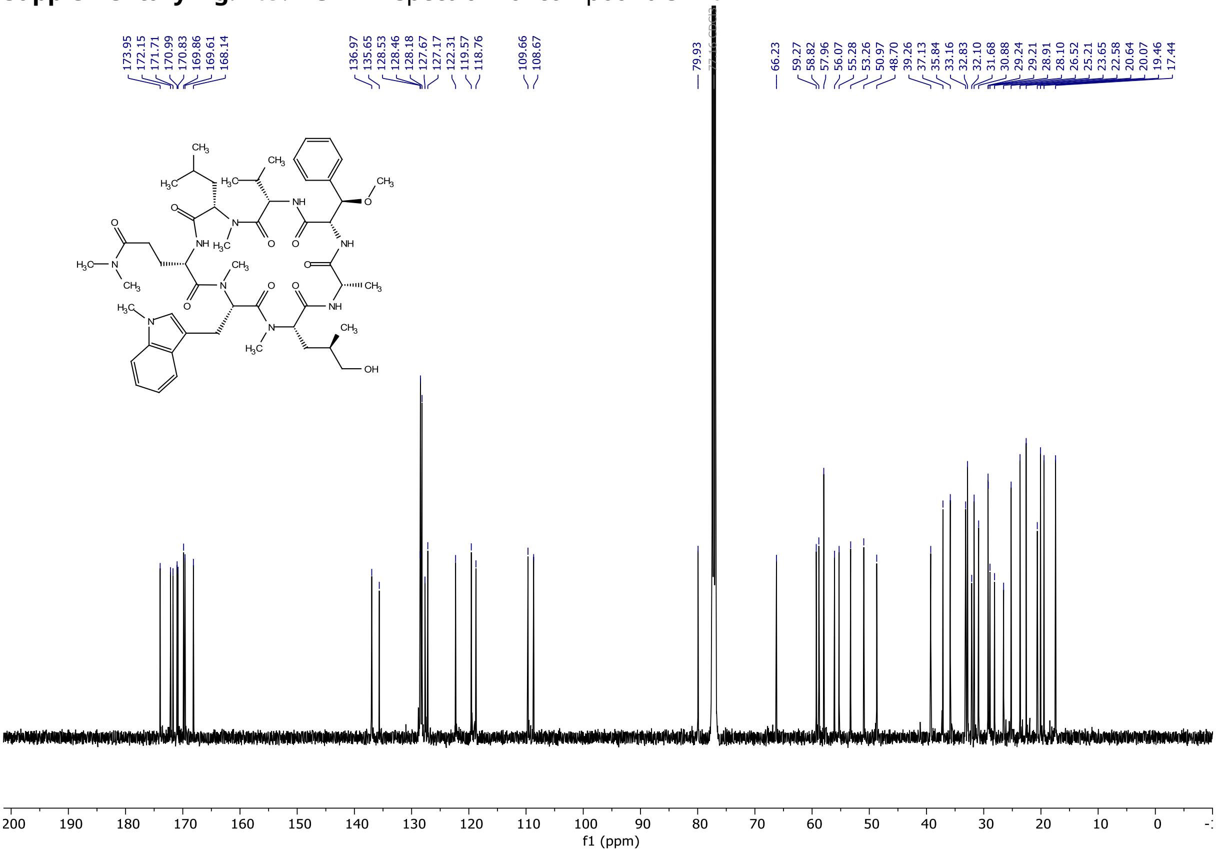

Supplementary Fig. 210: SPR stacking experiment

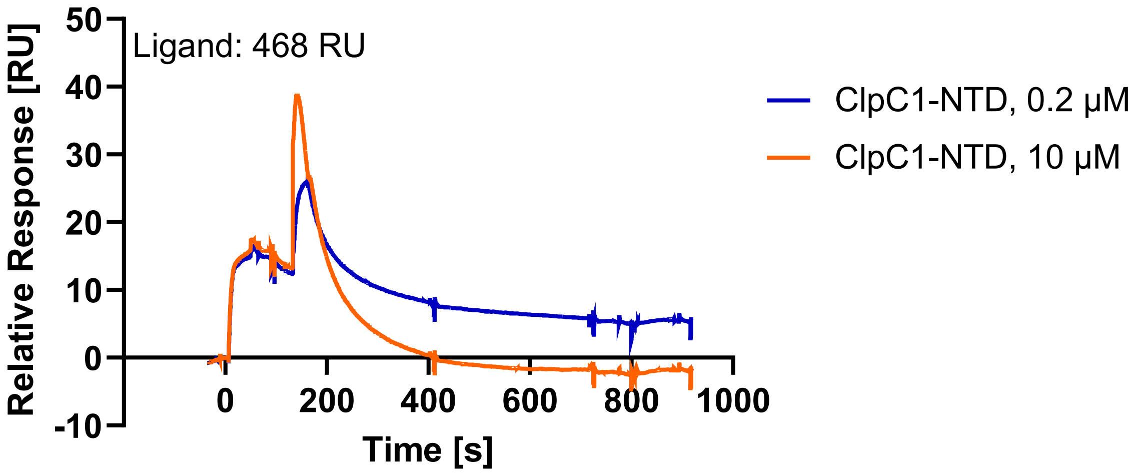

Supplementary Fig. 210. SPR stacking experiment**.** ClpC1-NTD was immobilized on a CM5 chip. After injection of 0.2 µM compound **8 (UdSBI-0545)**, ClpC1-NTD was injected at two concentrations. As can be seen in these sensorgrams, when injecting an equimolar concentration of ClpC1-NTD (0.2 µM ClpC1-NTD) the signal returned to the level observed at the end of the first injection. However, when injecting a 50-fold higher concentration of ClpC1-NTD (10 µM ClpC1-NTD), we still observe low binding of the protein, but the protein seems to completely remove the prebound compound from the chip.

References

1. Gengenbacher, M., Rao, S. P. S., Pethe, K. & Dick, T. Nutrient-starved, non-replicating Mycobacterium tuberculosis requires respiration, ATP synthase and isocitrate lyase for maintenance of ATP homeostasis and viability. *Microbiology* **156**, 81–87 (2010).

2. Abraham, M. H. *et al.* An NMR method for the quantitative assessment of intramolecular hydrogen bonding; Application to physicochemical, environmental, and biochemical properties. *Journal of Organic Chemistry* **79**, 11075–11083 (2014).

3. Cierpicki, T. & Otlewski, J. Amide proton temperature coefficients as hydrogen bond indicators in proteins. *Journal of Biomolecular NMR* **21**, 249–261 (2001).

4. Barbie, P. & Kazmaier, U. Total Synthesis of Cyclomarin A, a Marine Cycloheptapeptide with Anti-Tuberculosis and Anti-Malaria Activity. *Organic Letters* **18**, 204–207 (2016).

5. Junk, L., Papadopoulos, E. & Kazmaier, U. Tryptophan N 1-Alkylation: Quick and Simple Access to Diversely Substituted Tryptophans. *Synthesis* **53**, 2503–2511 (2021).

6. Kiefer, A. *et al.* Synthesis of New Cyclomarin Derivatives and Their Biological Evaluation towards Mycobacterium Tuberculosis and Plasmodium Falciparum. *Chemistry - A European Journal* **25**, 8894–8902 (2019).

7. Gille, F. & Kirschning, A. Studies on the synthesis of peptides containing dehydrovaline and dehydroisoleucine based on copper-mediated enamide formation. *Beilstein Journal of Organic Chemistry* **12**, 564–570 (2016).

8. van de Plassche, M. A. T., Barniol-Xicota, M. & Verhelst, S. H. L. Peptidyl Acyloxymethyl Ketones as Activity-Based Probes for the Main Protease of SARS-CoV-2**. *ChemBioChem* **21**, 3383–3388 (2020).

9. Otake, Y. *et al.* N-Methylated Peptide Synthesis via Generation of an Acyl N-Methylimidazolium Cation Accelerated by a Brønsted Acid. *Angewandte Chemie* **132**, 13025–13030 (2020).

10. Kwit, M., Rozwadowska, M. D., Gawroński, J. & Grajewska, A. Density functional theory calculations of the optical rotation and electronic circular dichroism: The absolute configuration of the highly flexible trans-isocytoxazone revised. *Journal of Organic Chemistry* **74**, 8051–8063 (2009).

11. Yan, L. J. *et al.* Development of Bifunctional Thiourea Organocatalysts Derived from a Chloramphenicol Base Scaffold and their Use in the Enantioselective Alcoholysis of meso Cyclic Anhydrides. *ChemCatChem* **8**, 2249–2253 (2016).

12. Kiefer, A. & Kazmaier, U. Synthesis of modified β-methoxyphenylalanines via diazonium chemistry and their incorporation in desoxycyclomarin analogues. *Organic and Biomolecular Chemistry* **17**, 88–102 (2019).

13. Mulzer, J., Mantoulidis, A. & Öhler, E. Total syntheses of epothilones B and D. *Journal of Organic Chemistry* **65**, 7456–7467 (2000).

14. O'Sullivan, P. T. *et al.* A Concise Synthesis of the Octalactins. *Journal of the American Chemical Society* **126**, 2194–2207 (2004).

15. Jia, M. *et al.* Novel aminopeptidase N (APN/CD13) inhibitors derived from chloramphenicol amine. *Bioorganic and Medicinal Chemistry* **19**, 5190–5198 (2011).
