# Supplementary material for: Homo-BacPROTAC-induced degradation of ClpC1 as a strategy against drug-resistant mycobacteria
Source: Nat Commun. 2024 Mar 5;15:2005. doi: 10.1038/s41467-024-46218-7 (PMC10914731; doi:10.1038/s41467-024-46218-7)
Supplement: Supplementary file 2 — Reporting Summary [file 41467_2024_46218_MOESM2_ESM.pdf]

nature portfolio

Corresponding author(s): Lukas Junk, Guido Boehmelt, Harald Weinstabl

Last updated by author(s): Jan 15, 2024

Reporting Summary

Nature Portfolio wishes to improve the reproducibility of the work that we publish. This form provides structure for consistency and transparency in reporting. For further information on Nature Portfolio policies, see our Editorial Policies and the Editorial Policy Checklist.

Statistics

For all statistical analyses, confirm that the following items are present in the figure legend, table legend, main text, or Methods section.

n/a	Confirmed	
☐	☒	The exact sample size (n) for each experimental group/condition, given as a discrete number and unit of measurement
☐	☒	A statement on whether measurements were taken from distinct samples or whether the same sample was measured repeatedly
☒	☐	The statistical test(s) used AND whether they are one- or two-sided<br>*Only common tests should be described solely by name; describe more complex techniques in the Methods section.*
☒	☐	A description of all covariates tested
☐	☒	A description of any assumptions or corrections, such as tests of normality and adjustment for multiple comparisons
☐	☒	A full description of the statistical parameters including central tendency (e.g. means) or other basic estimates (e.g. regression coefficient) AND variation (e.g. standard deviation) or associated estimates of uncertainty (e.g. confidence intervals)
☒	☐	For null hypothesis testing, the test statistic (e.g. F, t, r) with confidence intervals, effect sizes, degrees of freedom and P value noted<br>*Give P values as exact values whenever suitable.*
☒	☐	For Bayesian analysis, information on the choice of priors and Markov chain Monte Carlo settings
☒	☐	For hierarchical and complex designs, identification of the appropriate level for tests and full reporting of outcomes
☒	☐	Estimates of effect sizes (e.g. Cohen's d, Pearson's r), indicating how they were calculated

Our web collection on statistics for biologists contains articles on many of the points above.

Software and code

Policy information about availability of computer code

Data collection	Compass for Simple Western Software (ProteinSimple, multiple versions)<br>Biacore 8K Control Software V3.0.12.15655<br>Biacore T200 Control Software V3.2<br>Bruker TOPSPIN (multiple versions)<br>Image Quant TL Control Center
Data analysis	Compass for Simple Western Software (ProteinSimple, multiple versions)<br>Biacore Insight Evaluation Software V3.0.12.15655<br>Boehringer Ingelheim's MEGALAB<br>Amersham Imager 680 Analysis software V2.0.0<br>GraphPad Prism V9<br>Mestrenova v14.2<br>Bruker Compass DataAnalysis 4.4

For manuscripts utilizing custom algorithms or software that are central to the research but not yet described in published literature, software must be made available to editors and reviewers. We strongly encourage code deposition in a community repository (e.g. GitHub). See the Nature Portfolio guidelines for submitting code & software for further information.

Data

Policy information about availability of data

All manuscripts must include a data availability statement. This statement should provide the following information, where applicable:

- Accession codes, unique identifiers, or web links for publicly available datasets
- A description of any restrictions on data availability
- For clinical datasets or third party data, please ensure that the statement adheres to our policy

The authors declare that the data supporting the findings of this study are available within the paper, the Source Data file and the Supplementary Information. The SPR data generated in this study are provided in the Source Data file as a separate ZIP folder. The NMR data generated in this study are provided in the Supplementary Information. Should any raw data files be needed in another format they are available from the corresponding authors upon request.

Research involving human participants, their data, or biological material

Policy information about studies with human participants or human data. See also policy information about sex, gender (identity/presentation), and sexual orientation and race, ethnicity and racism.

Reporting on sex and gender	n/a
Reporting on race, ethnicity, or other socially relevant groupings	n/a
Population characteristics	n/a
Recruitment	n/a
Ethics oversight	n/a

Note that full information on the approval of the study protocol must also be provided in the manuscript.

Field-specific reporting

Please select the one below that is the best fit for your research. If you are not sure, read the appropriate sections before making your selection.

☒ Life sciences ☐ Behavioural & social sciences ☐ Ecological, evolutionary & environmental sciences

For a reference copy of the document with all sections, see nature.com/documents/nr-reporting-summary-flat.pdf

Life sciences study design

All studies must disclose on these points even when the disclosure is negative.

Sample size	For in vitro experiments, no dedicated sample size calculations were performed; typically, experiments were repeated at least 3 times independently (sufficient for calculation of mean and SD). In cases where n=3 or larger was not possible for technical reasons, assays were highly optimized with least 2 technical replicates.
Data exclusions	No data exclusions were performed unless indicated and explained otherwise for individual points in capillary Western (WES) where measurement quality standards were not matched (see Source Data for Figures 4 and 5).
Replication	All experiments were replicated independently at least twice, each typically with technical replicates, except were indicated otherwise in the figure legends.
Randomization	No defined randomization was performed for in vitro experiments but well allocation in cell-based and biochemical assays was arbitrary and varied over independent experimental replicates. Edge wells were left unused to avoid plate effects, where indicated.
Blinding	Blinding was neither possible nor relevant for in vitro or in vivo experiments due to experimental setup to ensure correct sample labeling. No clinical experiments were performed.

Reporting for specific materials, systems and methods

We require information from authors about some types of materials, experimental systems and methods used in many studies. Here, indicate whether each material, system or method listed is relevant to your study. If you are not sure if a list item applies to your research, read the appropriate section before selecting a response.

Materials & experimental systems

n/a	Involved in the study
☐	☒ Antibodies
☐	☒ Eukaryotic cell lines
☒	☐ Palaeontology and archaeology
☐	☒ Animals and other organisms
☒	☐ Clinical data
☒	☐ Dual use research of concern
☒	☐ Plants

Methods

n/a	Involved in the study
☒	☐ ChIP-seq
☒	☐ Flow cytometry
☒	☐ MRI-based neuroimaging

Antibodies

Antibodies used	# anti-His tag (R&D Systems MAB050)<br># 6x His tag (Abcam, ab252883, or ab206500)<br># anti-sheep secondary HRP-conjugated antibody (R&D anti-sheep IgG #HAF016)<br># antibodies used for detection of ClpC1 from M. smegmatis and M. tuberculosis were generated at MRCPPU Reagents and Services, University of Dundee, UK, by immunizing sheep with the following peptides: MFERFTDRARRVVVLAQEEAR (derived from the N-terminus of ClpC1, corresponding to amino acids 1 –21, 100% conserved between M. smegmatis and M. tuberculosis) and RRTIQREIEDQLSEKILFEEV (derived from the C-terminus of ClpC1, corresponding to amino acids 774–794, 100% conserved between M. smegmatis and M. tuberculosis). Antibody dilutions are indicated in the text.
Validation	All antibodies were obtained from reputable vendors. Refer to their websites for validation data and relevant citations for the species and application used in this study. Further, all antibodies are profiled in public repositories (RRID). Data provided in this manuscript (pharmacological manipulation of antibody targets) further validates the antibodies used. Custom-made anti-ClpC1 antibodies were validated across different bleedings using different assay conditions and including positive (e.g recombinant ClpC1) and negative (e.g. target knockdown) controls.

Eukaryotic cell lines

Policy information about cell lines and Sex and Gender in Research

Cell line source(s)	Eukaryotic cell lines were obtained from DSMZ (Caco-2), and ATCC (THP-1)
Authentication	The CaCo-2 cell line was authenticated by STR profiling (Eurofins Genomics).
Mycoplasma contamination	All cell lines used in the study were free of mycoplasma contamination in regular checks.
Commonly misidentified lines<br>(See ICLAC register)	None.

Animals and other research organisms

Policy information about studies involving animals; ARRIVE guidelines recommended for reporting animal research, and Sex and Gender in Research

Laboratory animals	Health status and Animal husbandry: The in-life experimental procedures were conducted in strict compliance with German and European animal welfare legislation in an AAALAC accredited facility. Male BALB/cAnNCrl mice (Charles River Laboratories Research Models and Services, 97633 Sulzfeld, Germany) were used. Animals were 9 weeks old at dosing with a body weight of 21.6 – 24.3 g. Animals were delivered free of pathogens according to FELASA recommendations with a health certificate provided by the breeder. Upon receipt, the state of health and sex was checked. Animals acclimatized for at least 5 days before commencement of the in-life phase. During acclimation, animals were group-housed in individually ventilated cages, on birch wood granulate bedding with species-specific enrichment (nesting material, mouse igloo, gnawing wood). During experimental conduct, animals were housed in conventional cages with elevated grid floor. Grid floor was used to avoid contamination with test item material excreted via urine and feces and subsequent reuptake during fur grooming. No bedding and enrichment were provided except a cage divider, which can be used as a shelter. Animals always had unlimited access to food and water and received a pelleted, total pathogen free maintenance diet and tap drinking water, sterilized by filter. Animals were euthanized by exsanguination in deep isoflurane anesthesia. For more details see Methods section.
Wild animals	No wild animals were used in the study.
Reporting on sex	Only male mice were used.
Field-collected samples	No field-collected samples were used in the study.
Ethics oversight	The authors confirm that the research in this study complies with all relevant ethical regulations. All animal studies were approved by the District Government of Upper Bavaria (Regierung von Oberbayern, Az.: 55.2-2532.Vet_03-17-101). All in-life experimental

procedures were conducted in strict accordance with the protocol and in compliance with German and European animal welfare legislation.

Note that full information on the approval of the study protocol must also be provided in the manuscript.

Plants

Seed stocks	n/a
Novel plant genotypes	n/a
Authentication	n/a
